# Isothiourea-catalysed enantioselective synthesis of phosphonate-functionalised β-lactones†

**DOI:** 10.1039/d5sc00322a

**Published:** 2025-03-06

**Authors:** Ffion M. Platt, Yihong Wang, David B. Cordes, Aidan P. McKay, Alexandra M. Z. Slawin, Heena Panchal, Andrew D. Smith

**Affiliations:** a EaStCHEM, School of Chemistry, University of St Andrews St Andrews Fife KY16 9ST UK ads10@st-andrews.ac.uk; b Chemical Development, PT&D, AstraZeneca Etherow Building, Silk Road Business Park, Charter Way Macclesfield Cheshire SK10 2NA UK

## Abstract

Despite growing interest in the reactivity and biological activity of phosphonate-containing molecules, the application of α-ketophosphonates in enantioselective formal [2 + 2] cycloadditions to generate β-lactones bearing a pendant phosphonate group remains unreported. In this manuscript, a highly diastereo- and enantioselective isothiourea-catalysed formal [2 + 2] cycloaddition of both alkyl- and aryl substituted C(1)-ammonium enolates and α-ketophosphonates is established. This strategy allows a mild, practical and scalable approach to highly enantioenriched C(3)-unsubstituted and C(3)-alkyl β-lactones bearing a phosphonate motif from their corresponding α-silyl acids, *via* a desilylative pathway (30 examples, up to 98%, >95 : 5 dr, >99 : 1 er). Alternatively, the use of (hetero)arylacetic acids allows the preparation of C(3)-(hetero)aryl β-lactones to be accessed in high yields and stereocontrol (19 examples, up to 98%, >95 : 5 dr, 99 : 1 er).

## Abstract 2

An isothiourea-catalysed formal [2 + 2] cycloaddition of alkyl- and aryl substituted C(1)-ammonium enolates and α-ketophosphonates gives phosphonate functionalised β-lactones with high enantioselectivity.

## Graphical abstract

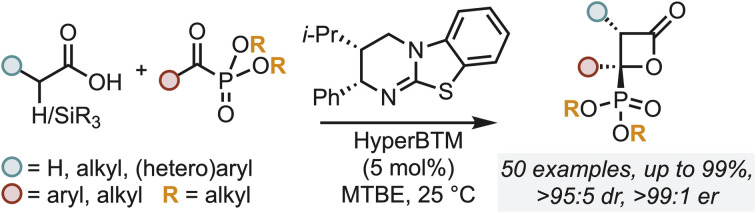

## Introduction

**Fig. 1:**
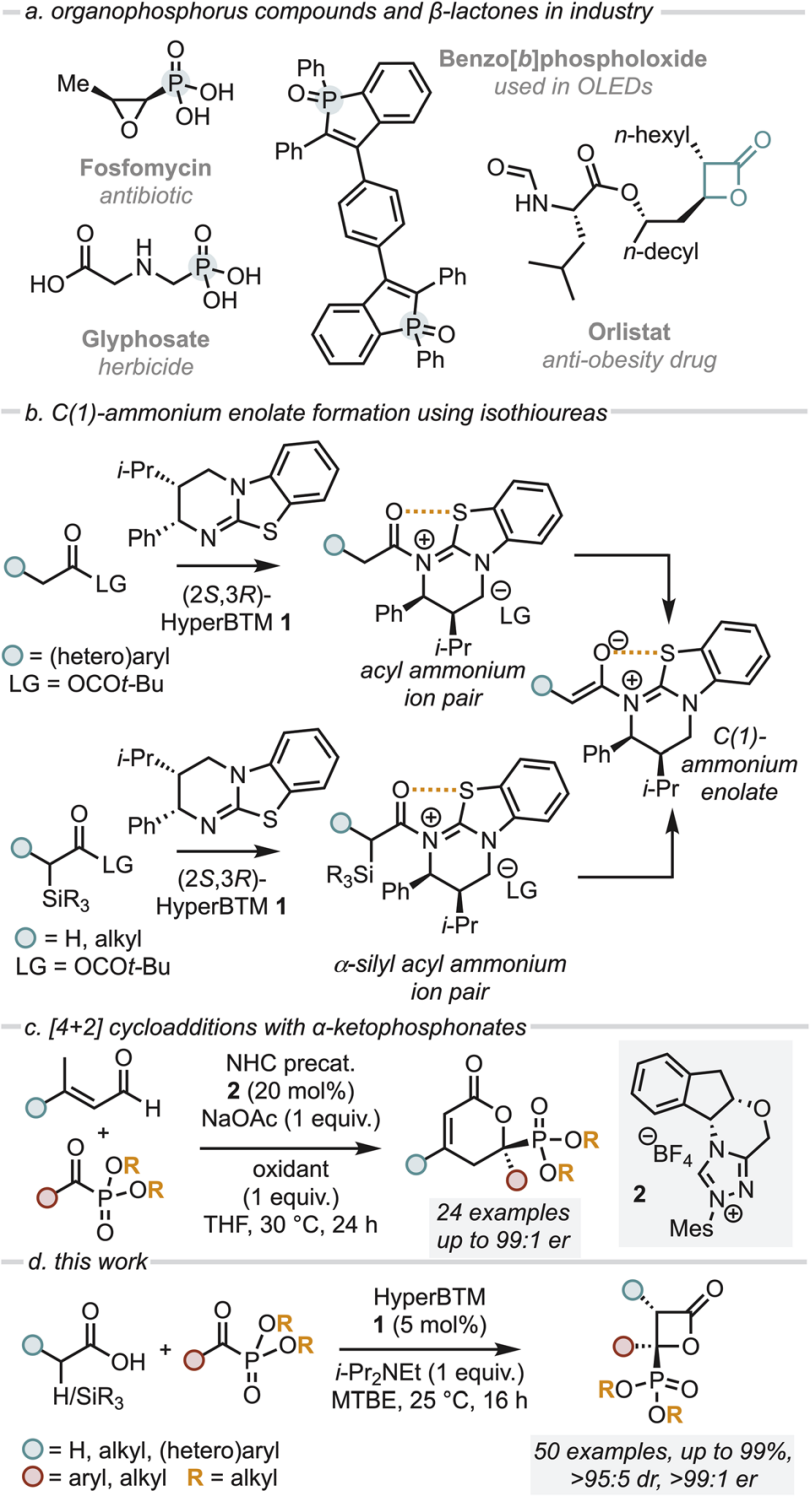
(a) Importance of organophosphorus compounds; (b) previous methods of generating C(1)-ammonium enolates using isothiourea catalysts; (c) relevant background formal [4 + 2] cycloaddition with α-ketophosphonates; (d) and this work.

Over the last two decades, heterocyclic molecules containing a phosphonic acid or related organophosphorus derivative have drawn broad interest due to their versatile properties and widespread biological activities, finding use as medicines, herbicides, optoelectronic materials and flame retardants.^1–4^ Representative examples include the broad-spectrum antibiotic fosfomycin, the herbicide glyphosate and optoelectronic material benzo[*b*]phospholoxide (Fig. 1a).^1–3^ Given the significant interest in these functionalities, a number of catalytic, enantioselective processes have been developed to generate stereodefined products bearing the phosphonate functionality *via* the use of α-ketophosphonates.^5–11^ In the same vein, β-lactones are versatile motifs which are prevalent in medicinal chemistry both as synthetic building blocks and as warheads in several biologically active compounds (Fig. 1a).^12^ Consequently, many enantioselective protocols exist for their synthesis,^13–16^ among which the use of C(1)-ammonium enolates generated from chiral tertiary amines (including the use of cinchona alkaloids, planar chiral DMAP derivatives and isothioureas) have been widely reported.^17^

C(1)-ammonium enolates are powerful reactive intermediates that have been harnessed for enantioselective C–C and C–X bond formation with a broad range of electrophiles. Classically, catalyst turnover occurred through intramolecular cyclisation to afford lactone or lactam products, however intermolecular turnover can be provided using electron-poor phenoxides, which has significantly broadened the scope of electrophiles compatible with this approach.^18–20^ Despite being widely applied, until recently intermolecular reactions with electrophiles involving C(1)-ammonium enolate formation from carboxylic acid starting materials using isothiourea catalysts (such as HyperBTM 1) was limited to (hetero)aryl-acetic acid derivatives. This presumably reflects the acidity of the α-C–H bond within an intermediate acyl ammonium ion pair for deprotonation to occur (Fig. 1b). To broaden the utility of C(1)-ammonium enolate reactivity to alkyl-substituted C(1)-ammonium enolates, we recently harnessed a desilylative approach from α-silyl carboxylic acids, expanding the scope of substitution within C(1)-ammonium enolates to include benzyl, alkyl and even acetic acid-derivatives (Fig. 1b).^21^

In previous work, Chi *et al.* demonstrated the enantioselective, NHC-catalysed formal [4 + 2] cycloaddition of α,β-unsaturated aldehydes with α-ketophosphonates in the presence of a stoichiometric oxidant to give 2-pyranylphosphonates (Fig. 1c).^22^ To the best of our knowledge enantioselective formal [2 + 2] cycloadditions using α-ketophosphonates to access β-lactones with a pendant phosphonate group remain unexplored to date. Building on these precedents, we describe a mild and highly enantioselective organocatalytic formal [2 + 2] cycloaddition of both alkyl C(1)-ammonium enolates (formed *via* a desilylative pathway) and (hetero)aryl C(1)-ammonium enolates (formed by a deprotonation pathway) with a range of α-ketophosphonates using the isothiourea (2*S*,3*R*)-HyperBTM 1 (Fig. 1d). This methodology gives access to a range of stereodefined β-lactones bearing two stereogenic centres, one of which bears a pendant phosphonate group, in excellent stereoselectivity.

## Results and discussion

### Investigation of optimal reaction conditions: reactivity of alkyl substituted C(1)-ammonium enolates by desilylation

Using α-trimethylsilyl propionic acid 3 and dimethyl benzoylphosphonate 4 as model substrates, several parameters were screened to find optimal conditions, including acid, catalyst, temperature and solvent (Table 1). Initially, using racemic acid 3 (2 equiv.), *t*-BuCOCl (3 equiv.) and i-Pr_2_NEt (3 equiv.) in MTBE to form the mixed anhydride *in situ*, followed by i-Pr_2_NEt (1 equiv.), (2*S*,3*R*)-HyperBTM 1 (5 mol%) and α-ketophosphonate 4 (1 equiv.) at room temperature gave the desired β-lactone 5 in 80% yield with exceptional diastereo- and enantioselectivity (>95 : 5 dr, >99 : 1 er) (entry 1). The use of alternative racemic acid 6 led to reduced yield, albeit with retention of high diastereo- and enantioselectivity (72%, >95 : 5 dr, >99 : 1 er, entry 2), however further variation of the silyl group using racemic acid 7 led to significantly reduced yield (<5%, entry 3). Control reactions showed that the α-silyl-substituent is necessary for reactivity in this process, as the use of propionic acid 8 (for subsequent mixed anhydride formation, entry 4) or propionic anhydride as starting material (entry 5) led to no formation of β-lactone 5. Changing to alternative isothiourea catalysts 9 and 10 led to no reaction (entries 6 and 7) and reducing the stoichiometry of acid 3 led to a reduction in yield, although maintained high stereoselectivity (35%, >95 : 5 dr, entry 8). When the temperature was increased to 25 °C (entry 9) β-lactone 5 was obtained in 98% yield, with exceptional stereocontrol (>95 : 5 dr, >99 : 1 er). Variation of the solvent at 25 °C saw no improvement upon these conditions (entries 10–13). Hence, entry 9 was chosen as the optimal reaction conditions.

**Table 1:** Variation of reaction conditions^a^

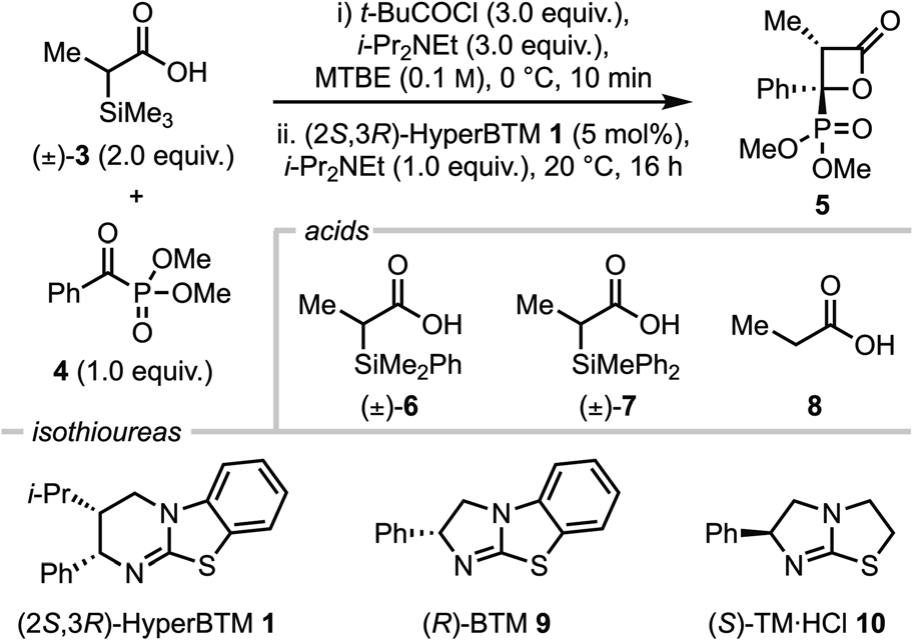				
Entry	Variation from standard	Yield^b^/%	dr^c^	er^d^
1	None	80	>95 : 5	>99 : 1
2	Acid 6	72	>95 : 5	>99 : 1
3	Acid 7	—	—	—
4	Acid 8	—	—	—
5	Propionic anhydride^e^	—	—	—
6	(*R*)-BTM 9	—	—	—
7	(*S*)-TM·HCl 10	—	—	—
8	Acid 3 (1 equiv.)	35	>95 : 5	>99 : 1
**9**	**25°C**	**98**	**>95 : 5**	**>99 : 1**
10	CH_2_Cl_2_, 25 °C	75	89 : 11	94 : 6
11	CH_3_CN, 25 °C	78	81 19	99 : 1
12	Toluene, 25 °C	80	>95 : 5	>99 : 1
13	THF, 25 °C	25	>95 : 5	79 : 21

a *t*-BuCOCl (1.2 mmol), i-Pr_2_NEt (1.2 mmol) and acid 3 (0.8 mmol) in MTBE (4 mL, 0.1 M) was stirred at 0 °C for 10 min before the addition of i-Pr_2_NEt (0.4 mmol), α-ketophosphonate 4 (0.4 mmol) and (2*S*,3*R*)-HyperBTM 1 (5 mol%) at 20 °C for 16 h. Temperatures of 25 °C were maintained using an oil bath. MTBE = methyl *tert*-butyl ether. BTM = benzotetramisole. TM = tetramisole.

b Isolated yield.

c Determined by ^1^H NMR of crude reaction mixture.

d Determined by HPLC analysis on a chiral stationary phase.

e propionic anhydride (1.0 mmol), i-Pr_2_NEt (0.5 mmol), α-ketophosphonate 4 (0.4 mmol) and (2*S*,3*R*)-HyperBTM 1 (5 mol%) in MTBE (4 mL) at rt for 16 h.

### Scope and limitations

**Fig. 2:**
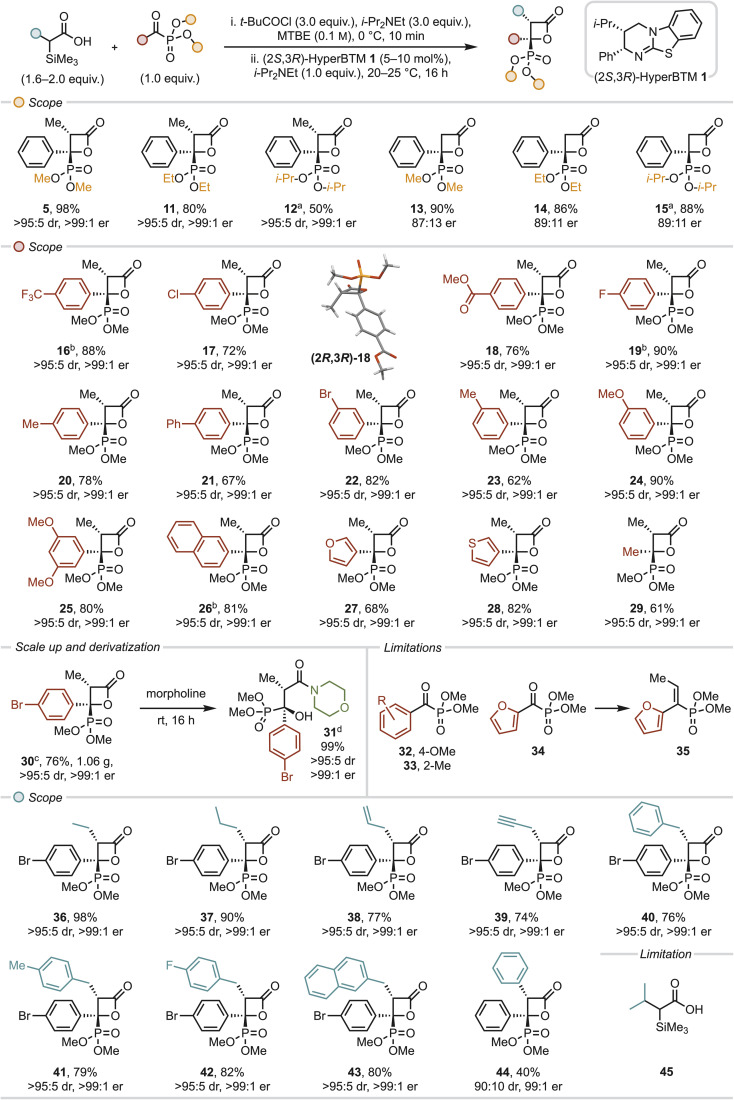
Scope and limitations of the formal [2 + 2] cycloaddition of α-silyl acids with α-ketophosphonates. All yields are isolated yields, dr determined by ^1^H NMR of crude reaction mixture, er determined by HPLC analysis on a chiral stationary phase, reaction scale 0.4 mmol, 25 °C maintained using an oil bath. ^*a*^10 mol% HyperBTM 1 used; ^*b*^1.6 equiv. of acid used, performed at 20 °C; ^*c*^initial reaction carried out on 0.4 mmol scale, scale up reaction carried out on 4.0 mmol scale, both gave 31 in 76% yield, >95 : 5 dr, >99 : 1 er; ^*d*^morpholine (5.0 equiv.) added after full conversion to β-lactone 31, 20 °C, 16 h.

With optimised conditions in hand, the scope and limitations of the developed process were investigated through variation of the phosphonate alkyl group under the standard reaction conditions (Fig. 2). Increasing substitution of the phosphonate group from methyl to ethyl and iso-propyl gave β-lactones 11 and 12 in reduced yield (80% and 50% respectively) compared to 5 but with exceptional stereocontrol (both >95 : 5 dr, >99 : 1 er). Conversely, taking unsubstituted α-silyl acetic acid with α-ketophosphonates of increasing substitution gave C(3)-unsubstituted β-lactones 13–15 in high yields (86–99%) but reduced enantioselectivity (87 : 13–89 : 11 er).

Subsequent work varied the steric and electronic nature of the aryl substituent within the benzoylphosphonate. The incorporation of electron-withdrawing groups in the 4-position were tolerated well, with compounds 16–19 (4-F_3_CC_6_H_4_-, 4-CO_2_MeC_6_H_4_, 4-ClC_6_H_4_ and 4-FC_6_H_4_) prepared in good to high yields (72–90%) with exceptional stereoselectivity (all >95 : 5 dr, >99 : 1 er). Other aryl C(4)-substituents within the benzoylphosphonate were also well-tolerated, giving 4-Me β-lactone 20 and 4-Ph β-lactone 21 in good yields and stereoselectivities (67–78%, both >95 : 5 dr, >99 : 1 er). Further substitution patterns and groups were also compatible, with 3-MeOC_6_H_4_, 3-BrC_6_H_4_, 3-MeC_6_H_4_ and 3,5-(MeO)_2_C_6_H_3_ all providing efficient access to the corresponding β-lactones 22–25 with high diastereo- and enantioselectivity (62–90%, all >95 : 5 dr, >99 : 1 er).

Reduced acid stoichiometry (1.6 equiv.) was used with a 2-naphthoylphosphonate, facilitating access to the corresponding β-lactone 26 in 81% yield with exceptional stereoselectivity (>95 : 5 dr, >99 : 1 er). In addition to substituted benzoylphosphonates, heterocyclic α-ketophosphonates were also well-tolerated, providing C(4)-3-furyl and 3-thiophenyl β-lactones 27 and 28 in high yields and stereoselectivities (68–82%, both >95 : 5 dr, >99 : 1 er). Single crystal X-ray diffraction of both 18 and 26 allowed confirmation of their relative and absolute configuration, which is consistent with the stereochemical rationale previously identified within the group for other formal [2 + 2]-cycloadditions using C(1)-ammonium enolates.^23^ Pleasingly, when dimethyl acetylphosphonate was employed, β-lactone 29 bearing a C(4)-methyl substituent was formed in 61% yield, with >95 : 5 dr and >99 : 1 er.

The reaction was scaled up, and β-lactone 30 was formed on gram scale with no erosion of stereoselectivity (76%, >95 : 5 dr, >99 : 1 er). Furthermore, treatment of 30 with excess morpholine under ambient conditions provided ring-opened α-hydroxyl-β-amidylphosphonate 31 bearing two contiguous stereocentres in high yield, with no detriment to the diastereo- or enantioselectivity (94%, >95 : 5 dr, >99 : 1 er). The limitations of this protocol were discovered throughout the course of these investigations. Strongly electron-donating groups on the aryl phosphonate component were not well-tolerated, with for example 4-MeOC_6_H_4_ substituted α-ketophosphonate 32 leading to less than 5% conversion to the corresponding β-lactone. Furthermore, 2-methylbenzoyl phosphonate 33 was significantly less reactive than its 4-methyl counterpart, leading to <5% conversion of the starting material. While use of 3-furoylphosphonate gave β-lactone 27 in good yield and the product was bench stable, the 2-furoylphosphonate 34 gave no β-lactone product, but instead alkene 35 was obtained in 58% yield, which presumably arises from decarboxylation of the initially formed β-lactone product.^24^

Further investigations focused on the scope of the α-silyl carboxylic acid. A variety of alkyl (Me, Et), as well as allyl and propargyl groups were well-tolerated, giving the corresponding β-lactones 36–39 in high yields (77–98%) and exceptional diastereo- and enantioselectivity (all >95 : 5 dr, >99 : 1 er). Benzyl-derived acids, including those bearing 4-MeC_6_H_4_ and 4-FC_6_H_4_ substituents, also showed excellent reactivity, affording β-lactones 40–42 in good yields (76–82%) with consistently high stereocontrol (all >95 : 5 dr, >99 : 1 er). Furthermore, 3-(naphthalen-2-yl)propanoic acid delivered the corresponding β-lactone 43 in 80% yield, with >95 : 5 dr and >99 : 1 er. The reaction of α-trimethylsilyl phenylacetic acid was also investigated, which provided β-lactone 44 in only a moderate yield and with a noticeable decrease in diastereoselectivity, although high enantioselectivity was maintained (40%, 90 : 10 dr, 99 : 1 er). Disappointingly, branched iso-propyl α-silyl acid 45 showed no reactivity under the reaction conditions.

### Probing double stereodifferentiation: matched and mismatched reaction pairings with enantioenriched anhydride

**Fig. 3:**
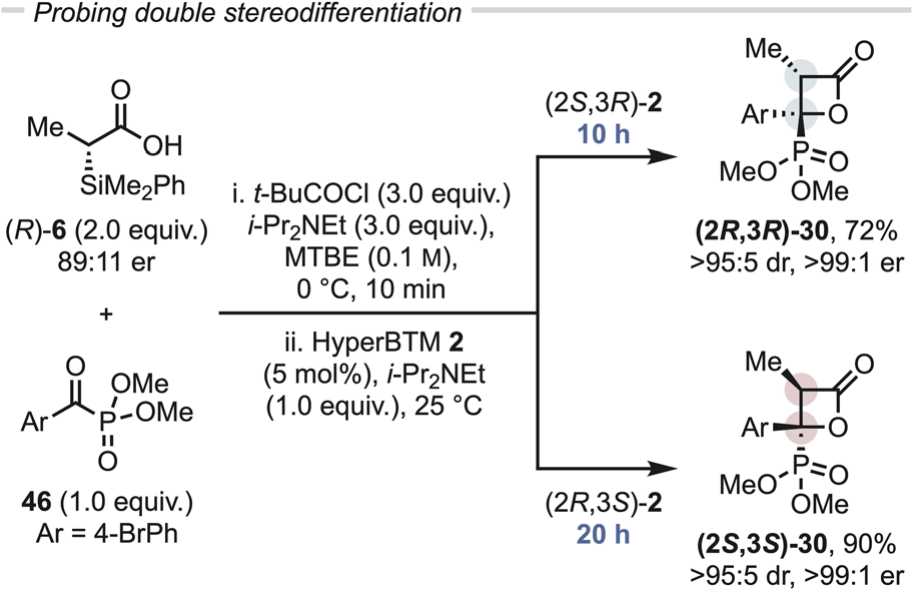
Probing double stereodifferentiation. All yields are isolated yields, dr determined by ^1^H NMR of crude reaction mixture, er determined by HPLC analysis on a chiral stationary phase.

As a control, rather than (±)-α-alkyl-α-silyl mixed anhydride, the use of enantioenriched α-silyl acid 6 (89 : 11 er) was investigated upon reaction with the enantiomers of HyperBTM 1 (Fig. 3). Using (2*S*,3*R*)-1 gave full conversion to (2*R*,3*R*)-30 within 10 hours in 72% yield and excellent stereocontrol (>95 : 5 dr, >99 : 1 er). However, the use of enantiomeric (2*R*,3*S*)-1 gave (2*S*,3*S*)-30 with identical stereocontrol (>95 : 5 dr, >99 : 1 er) but took 20 hours to reach completion. This is consistent with the previously observed kinetic resolution of the α-silyl anhydride taking place upon acylation in this protocol.^21^

### Re-optimisation of reaction conditions: (hetero)aryl substituted C(1)-ammonium enolates by deprotonation

Since the synthesis of β-lactone 44 from an aryl substituted-silyl acid had proceeded with reduced yield and diastereoselectivity compared to C(3)-alkyl or benzyl substituents, further work considered the propensity for formation of β-lactones bearing a C(3)-aryl substituent *via* a deprotonation route (Table 2). Building upon previous work that demonstrated the ability of phenylacetic anhydrides (either isolated or prepared *in situ*) to act as ammonium enolate precursors,^23^ the corresponding pivalic anhydride was prepared *in situ* from phenylacetic acid 47, with addition of α-ketophosphonate 4 leading to β-lactone 44 in 39% yield, 90 : 10 dr and 98 : 2 er (entry 1). Reducing the equivalents of base in the generation of the mixed pivalic anhydride led to a modest increase in yield, whilst maintaining high stereoselectivity (entry 2). Further variation in stoichiometry (see Tables S7 to S10†) led to an optimal ratio of 3 : 3 : 3 of acid 47 : *t*-BuCOCl : i-Pr_2_NEt. Further optimisation showed that varying the rate of acid had a significant impact on the reaction yield (entries 3–6), with addition of the acid over 5 min optimal, giving β-lactone 44 in an isolated yield of 74% with 90 : 10 dr and 96 : 4 er.

**Table 2:** Variation of reaction conditions^a^

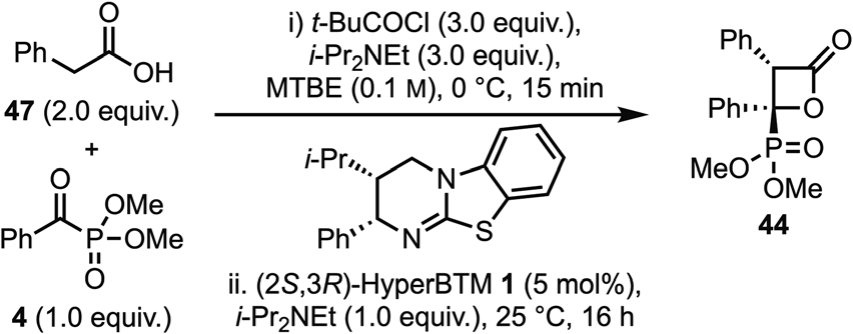				
Entry	Variation	Yield^d^/%	dr^e^	er^f^
1	None	39	90 : 10	98 : 2
2	2 equiv. of i-Pr_2_NEt in (i)	55	93 : 7	99 : 1
3^b^	3 equiv. 47, added over 1 min	72	87 : 13	96 : 4
**4^b^** ^**,**^	**3 equiv. 47, added over 5 min**	**74**	**90 : 10**	**96 : 4**
5^b^^,^^c^	3 equiv. 47, added over 15 min	56	88 : 12	96 : 4
6	(2*S*,3*R*)-HyperBTM (2.5 mol%)	38	82 : 18	83 : 17

a *t*-BuCOCl (0.6 mmol), i-Pr_2_NEt (0.6 mmol) and acid 47 (0.4 mmol) in MTBE (2 mL, 0.1 M) was stirred at 0 °C for 10 min, then i-Pr_2_NEt (0.2 mmol), α-ketophosphonate 4 (0.2 mmol) and (2*S*,3*R*)-HyperBTM 1 (5 mol%) at 25 °C for 16 h.

b 1.6 mL MTBE used in (i), then 0.4 mL added in (ii) to ensure all components rinsed into flask.

c Rate of addition controlled using syringe pump, 0.32 mL min^−1^ for 5 min or 0.11 mL min^−1^ for 15 min.

d Isolated yield.

e Determined by ^1^H NMR of crude reaction mixture.

f Determined by HPLC analysis on a chiral stationary phase.

### Probing *in situ* epimerisation of C(3)-aryl β-lactones under reaction conditions

**Fig. 4:**
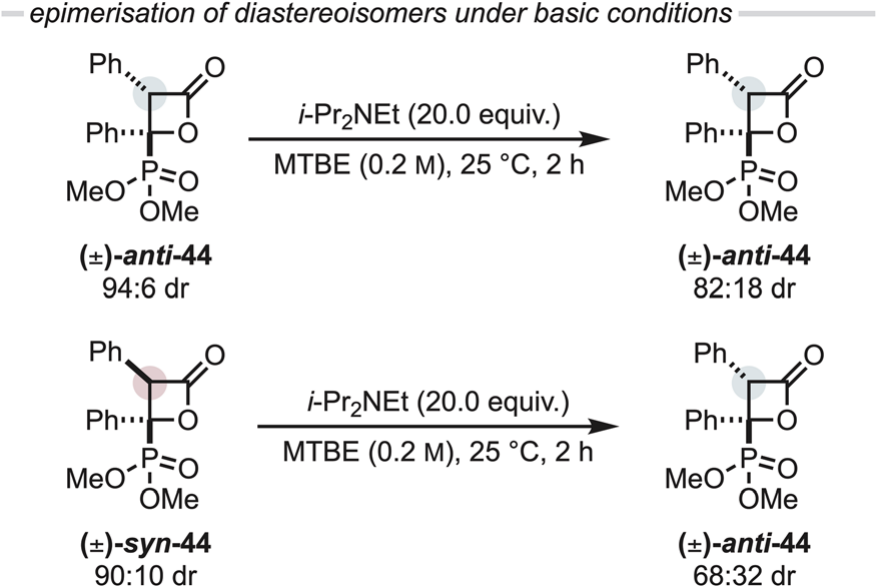
Epimerisation studies.

During the optimisation of this reaction, significant variation in product diastereoselectivity was observed and it was considered that this may be due to epimerisation of the β-lactone product. To evaluate the potential epimerisation of β-lactone 44 under the reaction conditions, isolated diastereomerically enriched samples of (±)-*anti*- and (±)-*syn*-44 (94 : 6 and 10 : 90 dr_*anti*:*syn*_ respectively) were exposed to i-Pr_2_NEt (20.0 equiv.) in MTBE, at 25 °C for 2 h (Fig. 4). After this time the diastereomeric ratio of *anti*-44 was reduced from 94 : 6 to 82 : 18 dr_*anti*:*syn*_, while *anti*-44 (68 : 32 dr_*anti*:*syn*_) was also favoured starting from *syn*-β-lactone 44 (90 : 10 dr_*syn*:*anti*_). Consistent with previous observations, it is likely that the observed epimerisation occurs through base-mediated enolisation at the β-lactone C(3)-position, then reprotonation.^25–28^

### Scope and limitations

**Fig. 5:**
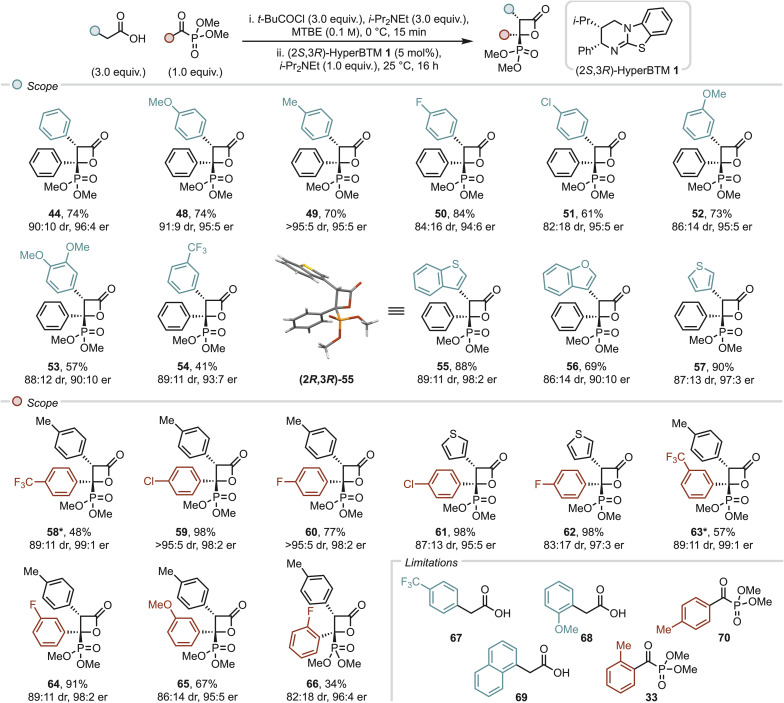
Scope and limitations of formal [2 + 2] cycloaddition of (hetero)arylacetic acids with α-ketophosphonates. All yields are isolated yields, dr determined by ^1^H NMR of crude reaction mixture, er determined by HPLC analysis on a chiral stationary phase. *α-ketophosphonate starting material showed dimerization over time (see ESI†).

With optimised conditions in hand, the generality of this methodology was investigated through variation of the arylacetic acid component (Fig. 5). Electron-donating groups in the aryl C(4)-position were well tolerated, giving β-lactones 48 and 49 in good yields, with high diastereo- and enantioselectivity (70–74%, 91 : 9–>95 : 5 dr, both 95 : 5 er). Halogens were also well-tolerated in this position (61–84%, 94 : 6–95 : 5 er), although β-lactones 50 and 51 were isolated with reduced diastereoselectivity (82 : 18–84 : 16 dr), which may be due to the increased susceptibility towards epimerisation at this stereocentre under the reaction conditions. Further variation around the aryl ring was also successful, with 3-OMe, 3,5-OMe and even 3-CF_3_ β-lactones 52–54 formed in synthetically useful yields, with good diastereo- and enantioselectivities (41–73%, 86 : 14–89 : 11 dr, 90 : 10–95 : 5 er). Pleasingly, heteroaryl substituents were also well-tolerated, with β-lactones 55–57 prepared in good to high yields with high diastereo- and enantioselectivity (69–90%, 86 : 14–89 : 11 dr, 90 : 10–98 : 2 er). Single crystal X-ray analysis of 55 confirmed the relative and absolute configuration, which aligns with that within the C(3)-alkyl scope. Subsequent investigations focused on the scope of the α-ketophosphonate. Incorporation of the electron-withdrawing 4-F_3_CC_6_H_4_ substituents gave a disappointing 48% yield of β-lactone 58, although good diastereoselectivity and excellent enantioselectivity were observed (89 : 11 dr, 99 : 1 er). Pleasingly, the reaction of both 4-tolylacetic acid and 3-thiophenylacetic acid with 4-ClC_6_H_4_– and 4-FC_6_H_5_-substituted benzoylphosphonates worked well, giving β-lactones 59–62 in high yields, with high diastereoselectivity and excellent enantioselectivity (77–98%, 83 : 17–>95 : 5 dr, 95 : 5–98 : 2 er).

Electron-withdrawing groups in other positions were also well-tolerated in this methodology with β-lactones 63–65 prepared in good yields with good diastereoselectivity and excellent enantioselectivity throughout (57–91%, 86 : 14–89 : 11 dr, 95 : 5–99 : 1 er). Interestingly, although aryl C(2)-substituents had not been tolerated in the α-ketophosphonate scope in the reaction with α-silyl acids (Fig. 2) or in the arylacetic acid scope (Fig. 5), 2-FC_6_H_5_ β-lactone 66 was prepared in a synthetically useful 34% yield, with modest diastereoselectivity (82 : 18 dr) but excellent enantioselectivity (96 : 4 er). Unfortunately, strongly Hammett electron-withdrawing 4-F_3_CC_6_H_4_-phenylacetic acid 67 showed low reactivity under the reaction conditions, which may be due to reduced nucleophilicity of the intermediate C(1)-ammonium enolate species. Sterically hindered acids 68 and 69 were also not tolerated. Disappointingly, the incorporation of an electron-donating methyl group in either the 4- or 2-position of the aryl-substituent of the phosphonate (70 and 32) showed no conversion to the desired β-lactone products, which presumably reflects the reduced electrophilicity of the carbonyl carbon, and/or steric hindrance in the case of the 2-MeC_6_H_5_ substituent.

### Proposed mechanism

**Fig. 6:**
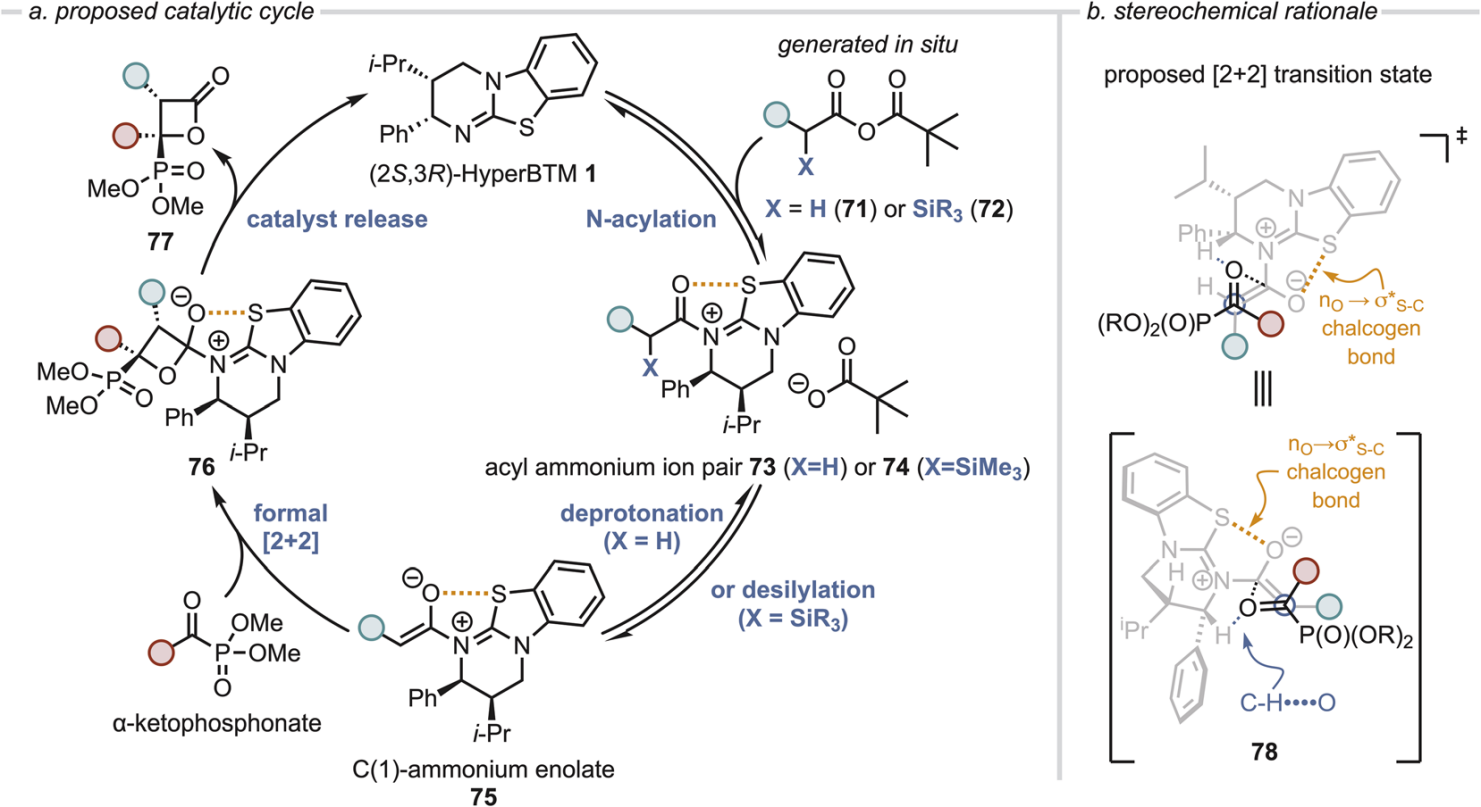
Proposed mechanism and stereochemical rationale.

Based upon these observations and following established precedent, a catalytic cycle for the enantioselective formal [2 + 2] cycloaddition of C(1)-ammonium enolates and α-ketophosphonates is presented (Fig. 6a) with the method of C(1)-ammonium enolate generation dependent upon the starting material. When (hetero)arylacetic mixed anhydrides 71 are used (X = H), *N*-acylation of (2*S*,3*R*)-HyperBTM 1 occurs reversibly to generate acyl ammonium ion pair 73, with deprotonation at the α-position generating the corresponding (*Z*)-C(1)-ammonium enolate 75. When an excess of (±)-α-alkyl-α-silyl mixed anhydride 72 is used, following our previous work preferential acylation of the (*R*)-enantiomer of 72 in a kinetic resolution process is assumed to lead to acyl ammonium ion pair 74 and enantioenriched anhydride 72. Desilylation to generate the corresponding C(1)-ammonium enolate 75 occurs, which may be promoted by the pivalate counterion or an alternative nucleophile such as a chloride anion, or through a Brook-type *C*- to *O*-silyl rearrangement followed by *O*-desilylation.^21^ In both cases, key to the observed stereochemical outcome is formation of the (*Z*)-ammonium enolate and a stabilizing 1,5-O⋯S chalcogen bonding interaction 
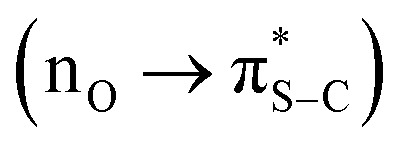
 which ensures coplanarity between the 1,5-O and S-atoms and provides a conformational bias.^29–50^ The relative and absolute configuration observed within the major diastereoisomer of the β-lactone products is consistent with that previously observed in related formal [2 + 2] cycloadditions of C(1)-ammonium enolates with trifluoromethylketones,^23^ isatins^51^ and pyrazolones^52^ and so by analogy a similar concerted asynchronous formal [2 + 2] cycloaddition pathway *via* transition state assembly 78 to give 77 is proposed. Following precedent,^21,51–53^ we speculate that this transition state assembly can be stabilised a non-classical CH⋯O interaction between the acidic α-C–H of the substrate-bound catalyst and the carbonyl C=O (Fig. 6b).^54^ Subsequent catalyst release *via* collapse of the spirocyclic tetrahedral intermediate 76 generates the β-lactone 77 in high diastereo- and enantioselectivity.

## Conclusion

This manuscript reports the development of a highly diastereo- and enantioselective formal [2 + 2] cycloaddition of α-unsubstituted, α-alkyl and α-(hetero)aryl-substituted C(1)-ammonium enolates with α-ketophosphonates using the isothiourea catalyst (2*S*,3*R*)-HyperBTM 1. The scope and limitations of this process have been explored, with good yields and high stereoselectivity observed across a broad range of substrate derivatives incorporating varied substitution at the C(3)- and C(4)-positions as well as the phosphonate group. This cycloaddition procedure is also amenable to scale up using bench grade solvents and reagents, with effective conversion on a 1 g scale demonstrated. The further application of this methodology towards unsymmetrical phosphonates is currently underway within our laboratory.

## Data availability

All data (experimental procedures and characterization data) that support the findings of this study are available within the article and its ESI.† Crystallographic data for compounds (2*R*,3*R*)-18, (2*R*,3*R*)-26 and (2*R*,3*R*)-55 have been deposited with the Cambridge Crystallographic Data Centre under deposition numbers 2395570, 2395571 and 2395572, respectively. The research data supporting this publication can be accessed at: DOI: https://doi.org/10.17630/a6b1715c-641b-47c2-8a28-d493726f8e5f, 2024, data underpinning: “Isothiourea-Catalysed Enantioselective Synthesis of Phosphonate-Functionalised β-lactones”, University of St Andrews Research Portal.

## Author contributions

ADS conceived the project. FMP and YW carried out all experimental studies in consultation with HP and ADS. FMP, YW and ADS wrote the manuscript. DBC, APM and AMZS carried out single crystal X-ray analysis. All authors agreed on the finalised version of the manuscript. FMP and YW contributed equally to this paper and both have the right to put their name first.

## Conflicts of interest

The authors declare no competing interests.

## Supplementary Material

SC-OLF-D5SC00322A-s001

SC-OLF-D5SC00322A-s002

## References

[cit1] Rodriguez J. B., Gallo-Rodriguez C. (2019). ChemMedChem.

[cit2] Dill G. M. , Sammons R. D., Feng P. C. C., Kohn F., Kretzmer K., Mehrsheikh A., Bleeke M., Honegger J. L., Farmer D., Wright D. and Haupfear E. A., in Glyphosate Resistance in Crops and Weeds, ed. V. K. Nandula, Wiley, 1st edn, 2010, pp. 1–33

[cit3] Popovics-Tóth N., Bálint E. (2022). Acta Chim. Slov..

[cit4] Lanzinger D., Salzinger S., Soller B. S., Rieger B. (2015). Ind. Eng. Chem. Res..

[cit5] Samanta S., Zhao C.-G. (2006). J. Am. Chem. Soc..

[cit6] Mandal T., Samanta S., Zhao C.-G. (2007). Org. Lett..

[cit7] Chen X., Wang J., Zhu Y., Shang D., Gao B., Liu X., Feng X., Su Z., Hu C. (2008). Chem.–Eur. J..

[cit8] Kayal S., Mukherjee S. (2015). Org. Lett..

[cit9] Yu J., Zhao X., Miao Z., Chen R. (2011). Org. Biomol. Chem..

[cit10] Gondi V. B., Hagihara K., Rawal V. H. (2009). Angew. Chem., Int. Ed..

[cit11] Frings M., Thomé I., Schiffers I., Pan F., Bolm C. (2014). Chem.–Eur. J..

[cit12] Robinson S. L., Christenson J. K., Wackett L. P. (2019). Nat. Prod. Rep..

[cit13] Yang H. W., Romo D. (1999). Tetrahedron.

[cit14] Smith A. D. , Douglas J., Morrill L. C. and Richmond E., in Methods and Applications of Cycloaddition Reactions in Organic Syntheses, John Wiley & Sons, Ltd, 2014, pp. 89–114

[cit15] Van K. N. , Morrill L. C., Smith A. D. and Romo D., in Lewis Base Catalysis in Organic Synthesis, John Wiley & Sons, Ltd, 2016, pp. 527–654

[cit16] Mukherjee S., Biju A. T. (2018). Chem.–Asian J..

[cit17] Morrill L. C., Smith A. D. (2014). Chem. Soc. Rev..

[cit18] McLaughlin C., Slawin A. M. Z., Smith A. D. (2019). Angew. Chem., Int. Ed..

[cit19] Schwarz K. J., Amos J. L., Klein J. C., Do D. T., Snaddon T. N. (2016). J. Am. Chem. Soc..

[cit20] Lee S. Y., Neufeind S., Fu G. C. (2014). J. Am. Chem. Soc..

[cit21] Wang Y., Young C. M., Liu H., Hartley W. C., Wienhold M., Cordes D. B., Slawin A. M. Z., Smith A. D. (2022). Angew. Chem., Int. Ed..

[cit22] Sun J., He F., Wang Z., Pan D., Zheng P., Mou C., Jin Z., Chi Y. R. (2018). Chem. Commun..

[cit23] Barrios Antúnez D.-J., Greenhalgh M. D., Brueckner A. C., Walden D. M., Elías-Rodríguez P., Roberts P., Young B. G., West T. H., Slawin A. M. Z., Ha-Yeon Cheong P., Smith A. D. (2019). Chem. Sci..

[cit24] Noyce D. S., Banitt E. H. (1966). J. Org. Chem..

[cit25] Matviitsuk A., Greenhalgh M. D., Taylor J. E., Nguyen X. B., Cordes D. B., Slawin A. M. Z., Lupton D. W., Smith A. D. (2020). Org. Lett..

[cit26] Kang G., Yamagami M., Vellalath S., Romo D. (2018). Angew. Chem., Int. Ed..

[cit27] Smith S. R., Douglas J., Prevet H., Shapland P., Slawin A. M. Z., Smith A. D. (2014). J. Org. Chem..

[cit28] Ji D.-S., Liang H., Yang K.-X., Feng Z.-T., Luo Y.-C., Xu G.-Q., Gu Y., Xu P.-F. (2022). Chem. Sci..

[cit29] Birman V. B., Li X., Han Z. (2007). Org. Lett..

[cit30] Liu P., Yang X., Birman V. B., Houk K. N. (2012). Org. Lett..

[cit31] Abbasov M. E., Hudson B. M., Tantillo D. J., Romo D. (2014). J. Am. Chem. Soc..

[cit32] Robinson E. R. T., Walden D. M., Fallan C., Greenhalgh M. D., Cheong P. H.-Y., Smith A. D. (2016). Chem. Sci..

[cit33] Greenhalgh M. D., Smith S. M., Walden D. M., Taylor J. E., Brice Z., Robinson E. R. T., Fallan C., Cordes D. B., Slawin A. M. Z., Richardson H. C., Grove M. A., Cheong P. H., Smith A. D. (2018). Angew. Chem., Int. Ed..

[cit34] Young C. M., Elmi A., Pascoe D. J., Morris R. K., McLaughlin C., Woods A. M., Frost A. B., de la Houpliere A., Ling K. B., Smith T. K., Slawin A. M. Z., Willoughby P. H., Cockroft S. L., Smith A. D. (2020). Angew. Chem., Int. Ed..

[cit35] Nagao Y., Miyamoto S., Miyamoto M., Takeshige H., Hayashi K., Sano S., Shiro M., Yamaguchi K., Sei Y. (2006). J. Am. Chem. Soc..

[cit36] Beno B. R., Yeung K.-S., Bartberger M. D., Pennington L. D., Meanwell N. A. (2015). J. Med. Chem..

[cit37] Pascoe D. J., Ling K. B., Cockroft S. L. (2017). J. Am. Chem. Soc..

[cit38] Breugst M., Koenig J. J. (2020). Eur. J. Org Chem..

[cit39] Mukherjee A. J., Zade S. S., Singh H. B., Sunoj R. B. (2010). Chem. Rev..

[cit40] Fujita K., Iwaoka M., Tomoda S. (1994). Chem. Lett..

[cit41] Fujita K., Murata K., Iwaoka M., Tomoda S. (1995). J. Chem. Soc., Chem. Commun..

[cit42] Fujita K., Murata K., Iwaoka M., Tomoda S. (1995). Tetrahedron Lett..

[cit43] Wirth T. (1995). Angew. Chem., Int. Ed..

[cit44] Bleiholder C., Gleiter R., Werz D. B., Köppel H. (2007). Inorg. Chem..

[cit45] Kolb S., Oliver G. A., Werz D. B. (2020). Angew. Chem., Int. Ed..

[cit46] Benz S., López-Andarias J., Mareda J., Sakai N., Matile S. (2017). Angew. Chem., Int. Ed..

[cit47] Wonner P., Vogel L., Düser M., Gomes L., Kniep F., Mallick B., Werz D. B., Huber S. M. (2017). Angew. Chem., Int. Ed..

[cit48] Wang W., Zhu H., Liu S., Zhao Z., Zhang L., Hao J., Wang Y. (2019). J. Am. Chem. Soc..

[cit49] Merad J., Pons J., Chuzel O., Bressy C. (2016). Eur. J. Org Chem..

[cit50] Birman V. B. (2016). Aldrichimica Acta.

[cit51] Abdelhamid Y., Kasten K., Dunne J., Hartley W. C., Young C. M., Cordes D. B., Slawin A. M. Z., Ng S., Smith A. D. (2022). Org. Lett..

[cit52] Conboy A., Goodfellow A. S., Kasten K., Dunne J., Cordes D. B., Bühl M., Smith A. D. (2024). Chem. Sci..

[cit53] Wu J., Young C. M., Watts A. A., Slawin A. M. Z., Boyce G. R., Bühl M., Smith A. D. (2022). Org. Lett..

[cit54] Agrawal S. K., Majhi P. K., Goodfellow A. S., Tak R. K., Cordes D. B., McKay A. P., Kasten K., Bühl M., Smith A. D. (2024). Angew. Chem., Int. Ed..

