# Supplementary material for: Isothiourea-catalysed enantioselective synthesis of phosphonate-functionalised β-lactones
Source: Chem Sci. 2025 Mar 6;16(16):6828–36. doi: 10.1039/d5sc00322a (PMC11915455; doi:10.1039/d5sc00322a)

Supplementary Information (SI) for Chemical Science.
This journal is © The Royal Society of Chemistry 2025

Isothiourea-Catalysed Enantioselective [2+2] Cycloaddition of C(1)-Ammonium Enolates and α-Ketophosphonates

Ffion M. Platt,[‡a] Yihong Wang, [‡a] David B. Cordes,[a] Aidan P. McKay,[a] Alexandra M. Z. Slawin,[a] Heena Panchal,[b] and Andrew D. Smith[a]

[a] Miss F. M. Platt, Dr Y. Wang, Dr D. B. Cordes, Dr A. P. McKay, Prof. Dr A. M. Z. Slawin, Prof. Dr A. D. Smith, EaStCHEM, School of Chemistry, University of St Andrews, St Andrews, Fife, UK. KY16 9ST. Email: ads10@st-andrews.ac.uk

[b] Dr H. Panchal, Chemical Development, PT&D, AstraZeneca, Etherow Building, Silk Road Business Park, Charter Way, Macclesfield, Cheshire, SK10 2NA.

Supporting Information

1. General Information 2
2. General Procedures 4
3. Optimisation 7
4. Synthesis of α-silyl acids 15
5. Synthesis of α-ketophosphonates 22
6. Catalysis Products – C(3)-Alkyl 32
7. Catalysis Products – C(3)-Aryl 54
8. Crystallographic Analysis 76
9. References 77
Appendix I. ^{1}H, ^{19}F, ^{31}P and ^{13}C NMR Spec 79
Appendix II. HPLC Traces 299

1. General Information

Reactions involving moisture sensitive reagents were carried out in flame-dried glassware under a nitrogen atmosphere using standard vacuum line techniques and anhydrous solvents. (*rac*)-HyperBTM **1**, (2*S,3*R)-HyperBTM **1** and benzotetramisole (BTM) **9** were synthesized in house,[1,2] Tetramisole•HCl **10** was obtained from Sigma-Aldrich. Anhydrous solvents (CH_2Cl_2, PhMe) were obtained by passing through an alumina column (Mbraun SPS-800). Anhydrous MTBE and MeCN were obtained by treatment with activated 4Å molecular sieves. Petrol is defined as petroleum ether 40–60 °C. All other solvents and commercial reagents were used as supplied without further purification unless otherwise stated. EtOAc, Et_2O, CH_2Cl_2 and petrol for purification purposes were used as obtained from suppliers without further purification. Room temperature (rt) refers to 20–25 °C. Temperatures of 0 °C and −78 °C were obtained using ice/water and CO_2(s)/acetone baths respectively. Reactions involving heating were performed using a DrySyn block and a contact thermocouple.

In vacuo refers to the use either a Büchi Rotavapor R-200 with a Büchi V-491 heating bath and Büchi V-800 vacuum controller; a Büchi Rotavapor R-210 with a Büchi V-491 heating bath and Büchi V-850 vacuum controller; a Heidolph Laborota 4001 with vacuum controller; an IKA RV10 rotary evaporator with an IKA HB10 heating bath and ILMVAC vacuum controller; or an IKA RV10 rotary evaporator with an IKA HB10 heating bath and Vacuubrand CVC3000 vacuum controller. Rotary evaporator condensers are fitted to Julabo FL601 Recirculating Coolers filled with ethylene glycol set to −6 °C.

Analytical thin layer chromatography was performed on pre-coated aluminium plates (Kieselgel 60 F254 silica). TLC visualisation was carried out with ultraviolet light (254 nm), followed by staining with a 1% aqueous KMnO4 solution. Automated chromatography was performed on a Biotage Selekt Four running Biotage OS578 with a UV/Vis detector using the method stated and cartridges filled with Kieselgel 60 silica.

Melting points were recorded on an Electrothermal 9100 melting point apparatus and are uncorrected, (*dec*) refers to decomposition.

Optical rotations were measured on a Perkin Elmer Precisly/Model-341 polarimeter operating at the sodium D line with a 100 mm path cell at 20 °C.

HPLC analyses were obtained using either a Shimadzu HPLC consisting of a DGU-20A5 degassing unit, LC-20AT liquid chromatography pump, SIL-20AHT autosampler, CMB-20A communications bus module, SPD-M20A diode array detector and a CTO-20A column oven; or a Shimadzu HPLC consisting of a DGU-20A5R degassing unit, LC-20AD liquid chromatography pump, SIL-20AHT autosampler, SPD-20A UV/Vis detector and a CTO-20A column oven. Separation was achieved using DAICEL CHIRALPAK AD-H, AS-H and IB and CHIRALCEL OD-H columns using the method stated. HPLC traces of

enantiomerically enriched compounds were compared with spectra of an authentic racemic sample prepared from the corresponding reaction using (*rac*)-HyperBTM **1** (5 mol%).

^{1}H, ^{13}C{1H}, ^{31}P{^{1}H} and ^{19}F{^{1}H} nuclear magnetic resonance (NMR) spectra were acquired on either a Bruker Avance II 400 (^{1}H 400 MHz; ^{13}C{^{1}H} 101 MHz, ^{31}P{^{1}H} 162 MHz, ^{19}F{^{1}H} 376 MHz) or a Bruker Avance II 500 (^{1}H 500 MHz; ^{13}C{^{1}H} 126 MHz, ^{31}P{^{1}H} 202 MHz, ^{19}F{^{1}H} 471 MHz) spectrometer at ambient temperature in the deuterated solvent stated. All chemical shifts are quoted in parts per million (ppm) and referenced to the residual solvent peak. All coupling constants, *J*, are quoted in Hz. Multiplicities are indicated by s (singlet), d (doublet), t (triplet), q (quartet), dd (doublet of doublets), dt (doublet of triplets), dq (doublet of quartets), tt (triplet of triplets), ddd (doublet of doublet of doublets) and m (multiplet). The abbreviation Ar is used to denote aromatic, Ph to denote phenyl, Bn to denote benzyl, br to denote broad and app to denote apparent. NMR peak assignments were confirmed using 2D ^{1}H correlated spectroscopy (COSY), ^{1}H−^{13}C heteronuclear single quantum coherence (HSQC) and 2D ^{1}H−^{13}C heteronuclear multiple-bond correlation spectroscopy (HMBC) where necessary.

Infrared spectra were recorded on a Shimadzu IRAffinity-1 Fourier transform IR spectrophotometer fitted with a Specac Quest ATR accessory (diamond puck). Spectra were recorded of either thin films or solids, with characteristic absorption wavenumbers (ν_{max}) reported in cm^{-1}.

Mass spectrometry (HRMS) data were acquired by either electrospray ionisation (ESI), electron impact (EI), atmospheric pressure chemical ionization (APCI) or nanospray ionization (NSI) either at either the University of St Andrews Mass Spectrometry Facility or at the University of Edinburgh Mass Spectrometry Facility.

2. General Procedures

2.1 General Procedure A: Synthesis of α-Silyl Acids

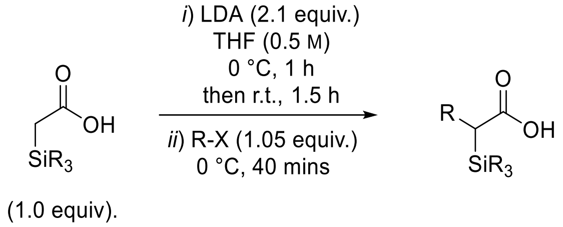

According to a procedure reported by Rogers *et al.*[3] , diisopropylamine (9.6 mmol, 2.1 equiv.) was dissolved in THF (10 mL) under an N_2-atmosphere. The solution was cooled to −78 °C and *n*-BuLi (9.6 mmol, 2.1 equiv.) was added. The mixture was warmed to rt for 15 min before being cooled to −78 °C again. 2-(Trimethylsilyl) acetic acid (4.5 mmol, 1.0 equiv.) was added and the mixture was stirred at 0 °C for 1 h, followed by 1.5 h at rt. Subsequently the specified halide (4.7 mmol, 1.05 equiv.) was added at 0 °C and the mixture was stirred additional 30 min at 0 °C. Then the reaction was quenched by the addition of HCl (1 M) and the pH adjusted to 2. The aqueous layer was extracted with Et_2O (3 × 15 mL). The combined organic layers were dried over $MgSO_4$, filtered and the solvent was removed under reduced pressure. The crude residue was triturated from pentane to give the desired product.

2.2 General Procedure B: Synthesis of Alternative α-Silyl Acids

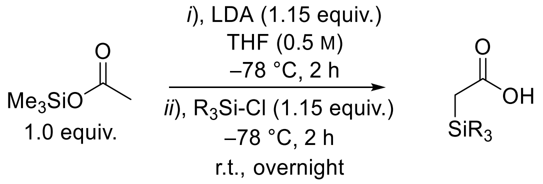

According to a procedure reported by Becker *et al*,[4] to an oven dried round-bottomed flask (250 mL) equipped with a magnetic stirring bar was added diisopropylamine (24.0 mmol, 1.15 equiv.) and anhydrous THF (40 mL). The mixture was cooled to −78 °C, then *n*-BuLi 1.6 M (24.0 mmol, 1.15 equiv.) was added dropwise. The mixture was warmed to rt for 15 min and cooled again to −78 °C. Trimethylsilyl acetate ($CH_3CO_2SiMe_3$) (21.0 mmol, 1.0 equiv.) was added dropwise to the cooled solution of LDA over 15 min and the reaction mixture was stirred for 2 h at −78 °C. Then chlorosilane (24.0 mmol, 1.15 equiv.) in anhydrous THF (5 mL) was added dropwise to the solution over 10 min. The reaction mixture was then stirred at −78 °C for 2 additional hours and allowed to reach rt overnight. A solution of saturated aqueous NaCl solution (30 ml) was added, and the pH was adjusted to 3 using 1 M aqueous HCl. The aqueous layer was extracted with Et_2O (3 × 30 mL) and the combined organic extracts were washed with water, dried over $MgSO_4$, filtered and concentrated under reduced pressure. The residual crude product was dissolved in THF (30 mL) and saturated aqueous NH_4Cl solution (20 mL) was added. The reaction mixture was then stirred at rt for 1 h. Afterwards, the aqueous layer was extracted with Et_2O (3

× 30 mL) and the combined organic extracts were washed with water (30 mL), and dried over $MgSO_4$, filtered and concentrated under reduced pressure. The crude residue was crystallized from hexane to give the desired product.

2.3 General Procedure C: Synthesis of α-ketophosphonates

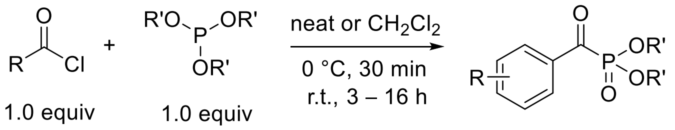

According to a procedure reported by Chi *et al*,[5] to a flame dried round bottomed flask equipped with a magnetic stirrer was added acid chloride (10 mmol, 1.0 equiv.) under nitrogen, and the flask cooled to 0 ℃. Trialkyl phosphite (10 mmol, 1.0 equiv.) was added dropwise at 0 ℃, then following addition the reaction stirred for 30 min at 0 ℃, then allowed to warm to rt over 3 h. The resulting oil was collected by vacuum distillation or column chromatography.

2.4 General Procedure D: Isothiourea-Catalysed Enantioselective [2+2] Cycloaddition of α-silyl acids and α-ketophosphonates

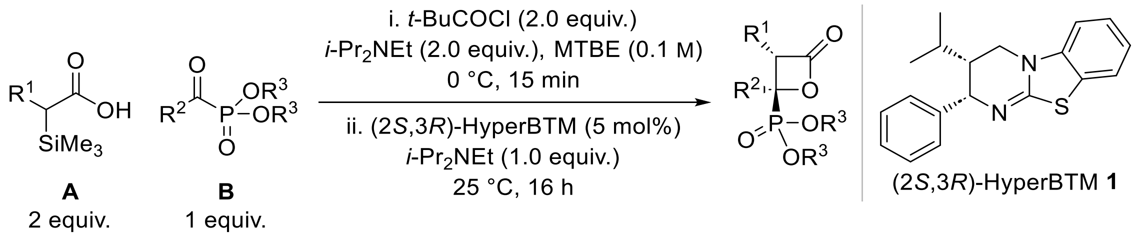

In a flame dried Schlenk tube under an N_2 atmosphere, *N,N*-diisopropylethylamine (3.0 equiv.) and pivaloyl chloride (3.0 equiv.) were added sequentially to a solution of appropriate acid (2.0 equiv.) in anhydrous MTBE (0.1 M) at 0 ℃. The mixture was allowed to stir for 15 min at 0 ℃, followed by the sequential addition of the specified ketone (1.0 equiv.), (2*S*,3*R*)-HyperBTM **1** (5 mol%) and *N,N*-diisopropylethylamine (1.0 equiv.). The mixture was allowed to stir for the specified time at rt. The solvent was then removed under reduced pressure, and the crude residue purified by Biotage Selekt column chromatography in the stated solvent system to give the desired product. Authentic racemic samples were prepared in an analogous fashion using (*rac*)-HyperBTM **1**.

2.5 General Procedure E: Isothiourea-Catalysed Enantioselective [2+2] Cycloaddition of (hetero)arylacetic acids and α-ketophosphonates

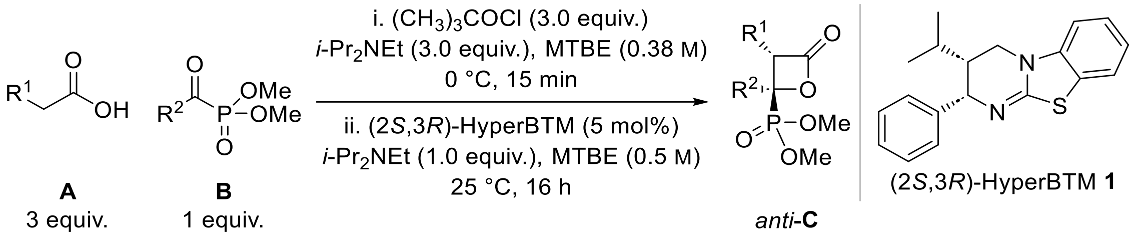

To a flame dried Schlenk tube charged with a magnetic stirrer bar were added pivaloyl chloride (0.6 mmol, 3 equiv) and *i*-Pr_2NEt (0.6 mmol, 3 equiv) and the mixture stirred at 0 ℃. Carboxylic acid **A** (0.6 mmol, 3 equiv) in MTBE (1.6 mL, 0.375 M) was added using a syringe pump (1.6 mL, 0.32 mL/min) at 0 ℃ under vigorous stirring. The mixture was stirred for a further 10 min at 0 °C, then (2*S*,3*R*)-HyperBTM **1** (3.1 mg, 5 mol%), *i*-Pr_2NEt (0.2 mmol, 1 equiv), dimethyl ketophosphonate **B** and MTBE (0.4 mL, 0.5 M) were added and the reaction stirred at 25 °C for 16 h. The crude reaction mixture was purified using flash silica column chromatography. Authentic racemic samples were prepared in an analogous fashion using (*rac*)-HyperBTM.

<u>3. Optimisation</u>

3.1 Optimisation for α-silyl acid and dimethyl benzoylphosphonate

Table S1

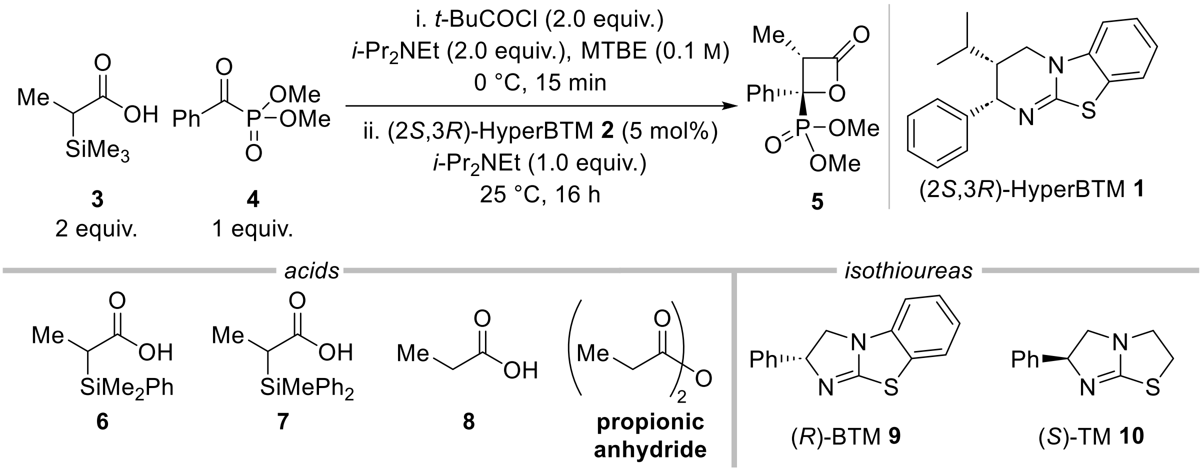

Entry	Variation	Yield[a]/%	dr[b]	er[c]
1	none	80	>95:5	>99:1
2	acid **6**	72	>95:5	>99:1
3	acid **7**	0	-	-
4	acid **8**	0	-	-
5	propionic anhydride[e]	0	-	-
6	(*R*)-BTM **9**	0	-	-
7	(*S*)-TM·HCl **10**	0	-	-
8	acid **3** (1 equiv.)	35	>95:5	>99:1
**9**	**25 ℃**	**98**	**>95:5**	**>99:1**
10	CH_2Cl_2, 25 ℃	75	89:11	94:6
11	CH_3CN, 25 ℃	78	81:19	99:1

12	toluene, 25 ℃	80	>95:5	>99:1
13	THF, 25 ℃	25	>95:5	79:21
14	2.5 mol% (2*S*,3*R*)-HyperBTM **1**	0	-	-

a) *t*-BuCOCl (1.2 mmol), *i*-Pr_2NEt (1.2 mmol) and acid **3** (0.8 mmol) in MTBE (4 mL, 0.1 M) was stirred at 0 ℃ for 10 min before the addition of i-Pr_2NEt (0.4 mmol), α-ketophosphonate **4** (0.4 mmol) and (2S,3R)-HyperBTM **1** (5 mol%) at rt for 16 h. MTBE = methyl tert-butyl ether. rt = room temperature. BTM = benzotetramisole. TM = tetramisole. b) Isolated yield. c) Determined by 1H NMR of crude reaction mixture. d) Determined by HPLC analysis on a chiral stationary phase. e) propionic anhydride (1.0 mmol), *i*-Pr_2NEt (0.5 mmol), α-ketophosphonate **4** (0.4 mmol), and (2S,3R)-HyperBTM **2** (5 mol%) in MTBE (4 mL) at rt for 16 h.

3.2 Optimisation for phenylacetic acid and dimethyl benzoylphosphonate

Table S2. Initial investigations considered different isochalcogenourea catalysts, concluding that the original catalyst HyperBTM **2** was optimal for reactivity and stereoselectivity.

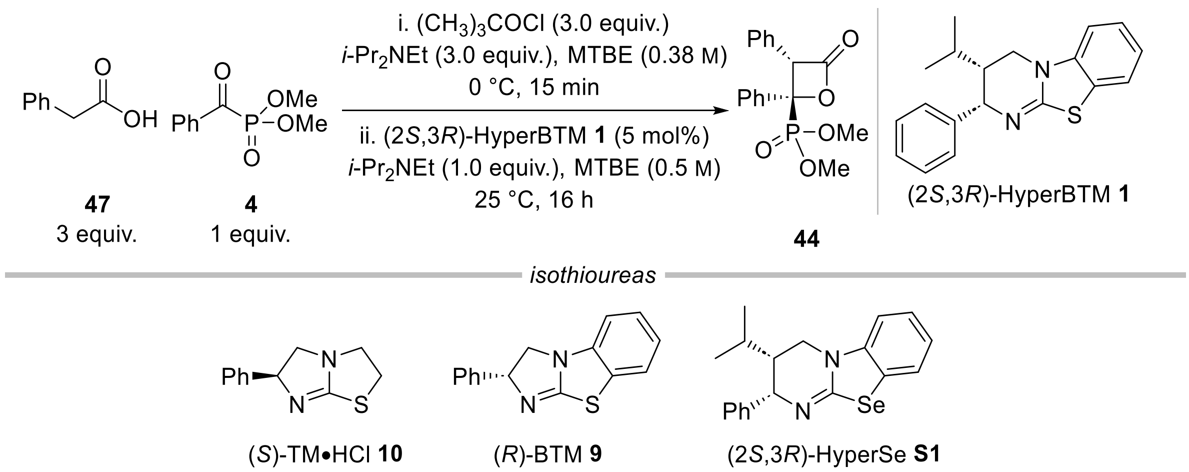

Entry	Variation	Yield[a]/%	dr[b]	er[c]
1	(*S*)-tetramisole•HCl **10** instead of **1**	0	-	-
2	(*R*)-BTM **9** instead of **1**	0	-	-
3	(2*S*,3*R*)-HyperSe **S1** instead of **1**	31	84:16	93:7

[a] Yields determined by 1H NMR analysis using 1,3,5-trimethoxybenzene as an internal standard; [b] dr determined by 1H NMR analysis; [c] er determined by HPLC analysis on a chiral stationary phase.

Table S3. Further investigations monitored the yield and diastereoselectivity over time, concluding that t = 2 h was optimal for diastereoselectivity.

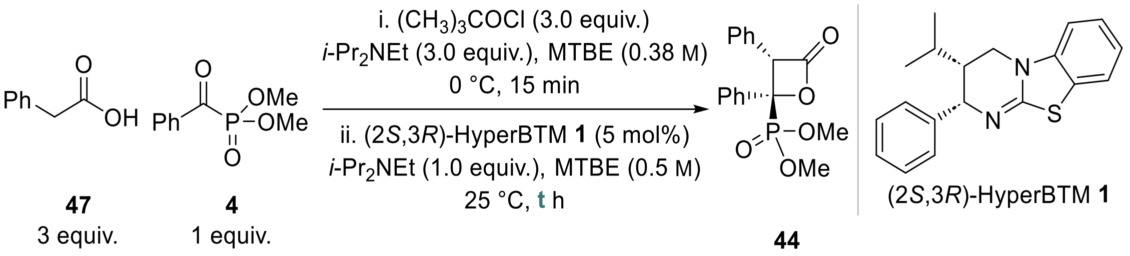

Entry	Variation	Yield[a]/%	dr[b]
1	ii. t = 1 h not 16 h	26	95:5
2	ii. t = 2 h not 16 h	32	95:5
3	ii. t = 3 h not 16 h	36	88:12
4	ii. t = 4 h not 16 h	39	85:15
5	ii. t = 5 h not 16 h	42	84:16

[a] Yields determined by 1H NMR analysis using 1,3,5-trimethoxybenzene as an internal standard; [b] dr determined by 1H NMR analysis; [c] er determined by HPLC analysis on a chiral stationary phase.

Table S4. A solvent screen was carried out at t = 2 h, but the original solvent MTBE was deemed best for both yield and stereoselectivity.

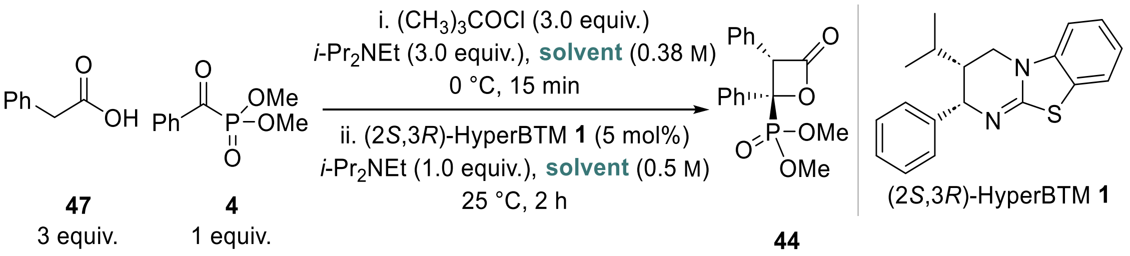

Entry	Variation	Yield[a]/%	dr[b]	er[c]
1	ii. t = 2 h, Et_2O instead of MTBE	21	95:5	91:9
2	ii. t = 2 h, 1,4-dioxane instead of MTBE	8	94:6	94:6

3	ii. t = 2 h, tetrahydrofuran instead of MTBE	7	88:12	94:6
4	ii. t = 2 h, acetonitrile instead of MTBE	22	84:16	92:8
5	ii. t = 2 h, ethyl acetate instead of MTBE	15	88:12	93:7
6	ii. t = 2 h, dichloromethane instead of MTBE	10	84:16	93:7
7	ii. t = 2 h, toluene instead of MTBE	15	88:12	96:4
8	ii. t = 2 h, methanol instead of MTBE	0	-	-

[a] Yields determined by 1H NMR analysis using 1,3,5-trimethoxybenzene as an internal standard; [b] dr determined by 1H NMR analysis; [c] er determined by HPLC analysis on a chiral stationary phase.

Table S5. The temperature was varied for t = 2 h, and while 25 ℃ and 40 ℃ gave similar results, 25 ℃ was chosen for ease of setup.

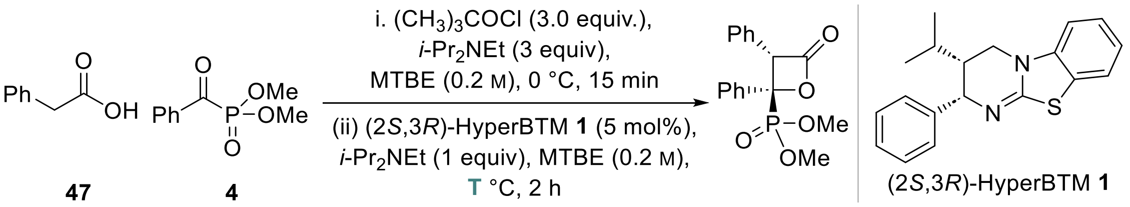

47 3 equiv. **4** 1 equiv. **44**

Entry	Variation	Yield[a]/%	dr[b]	er[c]
1	ii. t = 2 h, T = 0 ℃ instead of 25 ℃	8	87:13	-
2	ii. t = 2 h, T = 40 ℃ instead of 25 ℃	13	88:12	94:6

[a] Yields determined by 1H NMR analysis using 1,3,5-trimethoxybenzene as an internal standard; [b] dr determined by 1H NMR analysis; [c] er determined by HPLC analysis on a chiral stationary phase.

Table S6. Other variations carried out at t = 2 h, initial conditions remained optimal.

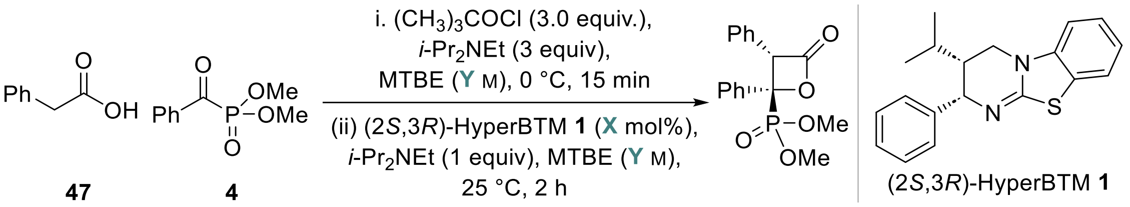

47 2 equiv. **4** 1 equiv. **44**

Entry	Variation	Yield[a]/%	dr[b]	er[c]
1	10 mol% **1** instead of 5 mol%	23	91:9	93:7
2	2.5 mol% **1** instead of 5 mol%	38	82:18	83:17
3	conc. = 0.2 M instead of 0.1 M	17	93:7	96:4

[a] Yields determined by 1H NMR analysis using 1,3,5-trimethoxybenzene as an internal standard; [b] dr determined by 1H NMR analysis; [c] er determined by HPLC analysis on a chiral stationary phase.

Table S7. Alternative carbonyl activation – homoanhydride over 16 h reaction time found to give best result, however a decrease in diastereoselectivity was observed. It was decided to further consider the stoichiometries of materials in step i for optimal conversion and diastereoselectivity.

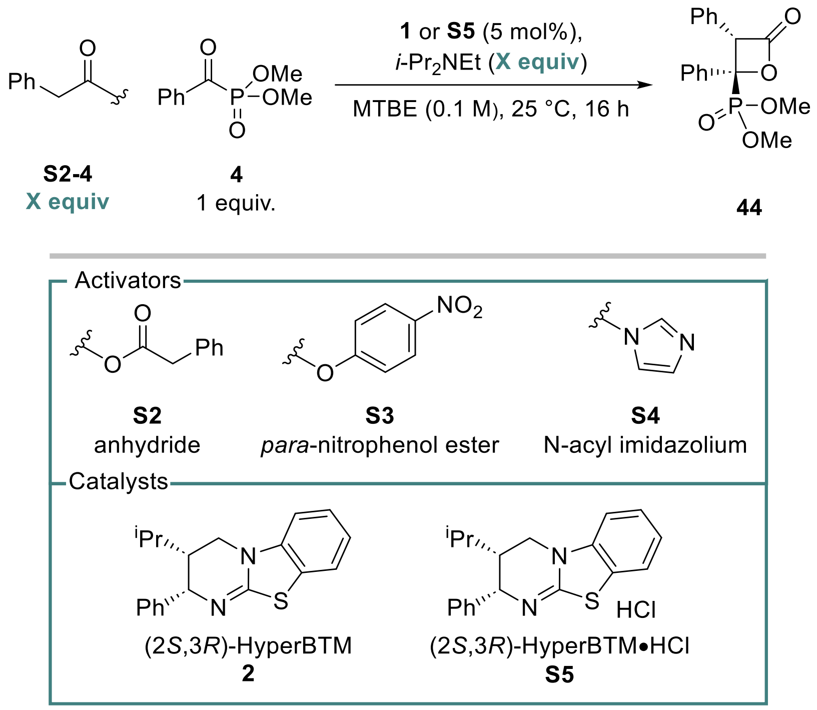

Entry	Variation	Yield[a]/%	dr[b]	er[c]
1	**S2** + **1**, X = 1	17	71:29	95:5
2	**S2** + **1**, X = 1, t = 2 h	20	84:16	93:7
3	**S2** + **1**, X = 2	59	81:19	94:6

4	**S2** + **1**, X = 3	51	84:16	90:10
5	**S3** + **1**, X = 1.5	1	-	-
6	**S4** + **S5**, X = 1	0	-	-
7	**S4** + **S5**, X = 2	0	-	-
8	**S4** + **S5**, X = 3	0	-	-

[a] Yields determined by 1H NMR analysis using 1,3,5-trimethoxybenzene as an internal standard; [b] dr determined by 1H NMR analysis; [c] er determined by HPLC analysis on a chiral stationary phase.

Table S8. Variation of pivaloyl chloride equivalents

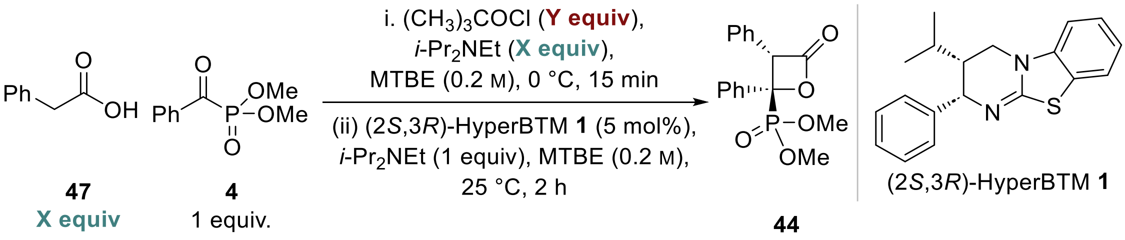

Entry	Ratio X:Y:1	Yield[a]/%	dr[b]	er[c]
1	1:1:1	6	96:4	-
2	1:1.5:1	9	96:4	-
3	1:2:1	7	91:9	-
4	1:3:1	2	78:22	-
5	2:3:1	13	>95:5	98:2
6[d]	2:3:1	58	93:7	99:1

[a] Yields determined by 1H NMR analysis using 1,3,5-trimethoxybenzene as an internal standard; [b] dr determined by 1H NMR analysis; [c] er determined by HPLC analysis on a chiral stationary phase.

Table S9. Variation of base, *i*-Pr_2NEt found to be ideal.

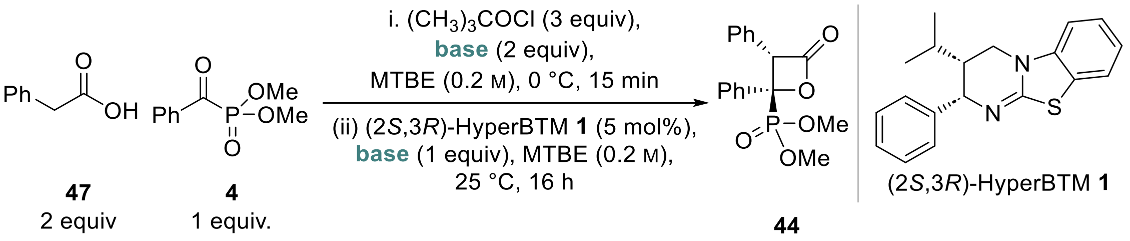

Entry	Variation	Yield[a]/%	dr[b]	er[c]
1[d]	None	0	-	-
2	K_2CO_3	7	86:14	-
3	K_2PO_3	6	88:12	-
4	Et_3N	48	86:14	-

[a] Yields determined by 1H NMR analysis using 1,3,5-trimethoxybenzene as an internal standard; [b] dr determined by 1H NMR analysis; [c] er determined by HPLC analysis on a chiral stationary phase.

Table S10. Variation of acid addition and concentration of step i – dropwise addition of acid found to be optimal for reproducibility and diastereoselectivity.

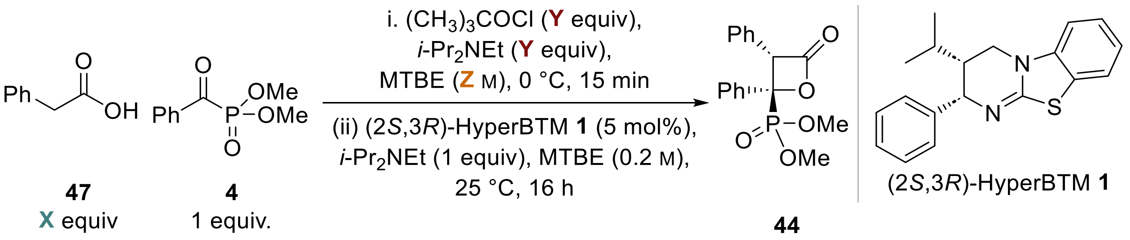

Entry	Variation	Yield[a]/%	dr[b]	er[c]
1	2:3:1, 0.25 M	40	95:5	96:4
2	3:3:1, 0.375 M	60	81:19	97:3
3	3:3:1, 0.30 M	54	85:15	97:3
4	3:3:1, 0.50 M	66	85:15	98:2

5	3:3:1, 0.75 M	76	85:15	96:4
6	3:3:1, 0.75 M, addition over 15 min	60	88:12	96:4
7	3:3:1, 0.75 M, addition over 5 min	53	85:15	95:5
8	3:3:1, 0.375 M, addition over 15 min	58	86:14	92:8
9	3:3:1, 0.375 M, addition over 5 min	58	84:16	94:6
10[d]	3:3:1, 0.375 M, addition over 5 min	78	90:10	96:4

[a] Yields determined by 1H NMR analysis using 1,3,5-trimethoxybenzene as an internal standard; [b] dr determined by 1H NMR analysis; [c] er determined by HPLC analysis on a chiral stationary phase. [d] ice stirred and repacked throughout to maintain 0 ℃ bath.

4. Synthesis of α-silyl acids

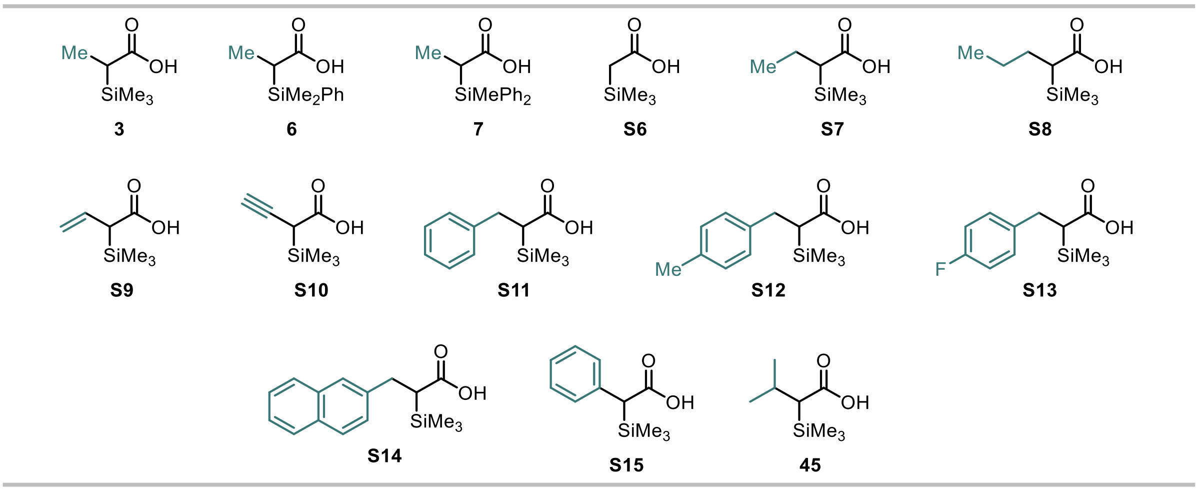

2-(Trimethylsilyl)propanoic acid (3)

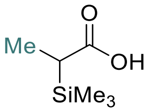

3

Following General Procedure A, diisopropylamine (1.35 mL, 9.6 mmol, 2.1 equiv.), *n*-BuLi (3.8 mL, 9.6 mmol, 2.1 equiv.), 2-(trimethylsilyl) acetic acid 1 (600 mg, 4.5 mmol, 1.0 equiv.), iodomethane (292 µL, 4.7 mmol, 1.05 equiv.) in THF (10 mL), gave, after trituration, 2-(trimethylsilyl)propanoic acid as a white solid (540 mg, 82%) with spectroscopic data in accordance with the literature.[3] **^{1}H NMR** (400 MHz, $CDCl_3$) δ_H: 2.12 (1H, q, *J* 7.0, COC*H*), 1.21 (3H, d, *J* 7.0, CHC*H*$_3$), 0.14 (9H, s, Si(C*H*$_3$)$_3$); **^{13}C{^{1}H} NMR** (126 MHz, $CDCl_3$) δ_C: 183.2 (*C*O), 30.5 (CO*C*H), 10.7 (CH*C*H$_3$), −3.0 (Si(*C*H$_3$)$_3$).

2-(Dimethyl(phenyl)silyl)propanoic acid (6)93:786

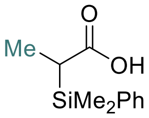

6

Following General Procedure A, diisopropylamine (1.8 mL, 12.8 mmol, 2.1 equiv.), *n*-BuLi (5.1 mL, 12.8 mmol, 2.1 equiv.), 2-(methyldiphenylsilyl)acetic acid S3 (1.07 g, 6.1 mmol, 1.0 equiv.), iodomethane (370 µL, 6.3 mmol, 1.05 equiv.) in THF (15 mL) gave. After trituration, 2-(dimethyl(phenyl)silyl)propanoic acid as a white solid (570 mg, 45%) with data in accordance with the literature.[6] **mp** 38 – 40 °C. **IR** ν_{max} (film) 1678 (C=O), 1115 (C−O). **^{1}H NMR** (400 MHz, $CDCl_3$) δ_H: 7.54 – 7.56 (2H, m, Ph*H*), 7.37 – 7.43 (3H, m, Ph*H*), 2.34 (1H, q, *J* 7.0, COC*H*), 1.17 (3H, d, *J* 7.0, CHC*H*$_3$), 0.45 (6H, d, *J* 2.6, Si(C*H*$_3$)$_2$Ph); **^{13}C{^{1}H}**

NMR (101 MHz, $CDCl_3$) δ_C: 182.5 (*C*O), 135.7 (Ph*C*(1)), 133.9 (Ph*C*(3,5)H), 129.7 (Ph*C*(4)H), 127.9 (Ph*C*(2,6)H), 30.2 (CO*C*H), 11.1 (CH*C*H_3), −3.9 (Si(CH_3*C*H_3), −5.2 (Si(*C*H_3CH_3); **HRMS** (ESI^+) $C_{11}H_{16}O_2NaSi$ $[M+Na]^+$ found 231.0805, requires 231.0812 (−2.7 ppm).

2-(Methyldiphenylsilyl)propanoic acid (7)

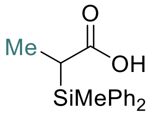

7

Following General Procedure A, diisopropylamine (1.8 mL, 12.8 mmol, 2.1 equiv.), *n*-BuLi (5.1 mL, 12.8 mmol, 2.1 equiv.), 2-(methyldiphenylsilyl)acetic acid S2 (1.56 g, 6.1 mmol, 1.0 equiv.), iodomethane (370 μL, 6.3 mmol, 1.05 equiv.) in THF (15 mL) gave. After trituration, 2-(dimethyl(phenyl)silyl)propanoic acid as a white solid (1.24 g, 75%) with spectroscopic data in accordance with the literature.[7] **^{1}H NMR** (400 MHz, $CDCl_3$) δ_H: 7.57 – 7.60 (4H, m, Ph*H*), 7.35 – 7.46 (6H, m, Ph*H*), 2.70 (1H, q, *J* 7.1, COC*H*), 1.25 (3H, d, *J* 7.1, CHC*H_3*), 0.70 (3H, s, SiC*H_3*); **^{13}C{^{1}H} NMR** (101 MHz, $CDCl_3$) δ_C: 181.7 (*C*O), 134.9 (Ph_B*C*(3,5)), 134.8 (Ph_A*C*(3,5)), 134.2 (Ph_B*C*(4)), 133.8 (Ph_A*C*(4)), 129.8 (Ph*C*(4)), 128.0 (Ph_B*C*(2,6)), 127.9 (Ph_A*C*(2,6)), 29.0 (CO*C*H), 11.9 (CH*C*H_3), −5.3 (Si*C*H_3).

2-(trimethylsilyl)acetic acid (S6)

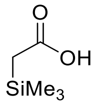

S6

Following a procedure reported by Bertounesque *et al.*[8], diisopropylamine (36.0 mmol, 2.1 equiv.) was dissolved in THF (35 mL) under an N_2-atmosphere. The solution was cooled to −78 °C and *n*-BuLi (36.0 mmol, 2.1 equiv.) was added. The mixture was warmed to rt for 25 min before it cooled to −78 °C again. At −78 °C, acetic acid (17 mmol, 1.0 equiv.) was added slowly, and the mixture was then heated to 68 °C for 24 h. The reaction was cooled to −78 °C again and trimethylsilyl chloride (41.0 mmol, 2.4 equiv.) was added. Then the mixture was warmed to rt overnight. A saturated aqueous NaCl solution (20 mL) was added, and the mixture was acidified to pH=2 by the addition of HCl (1 m). The aqueous layer was extracted with Et_2O (3 × 20 mL) and the organic layer was washed with H_2O (2 × 10 mL). The solvent was removed from the organic layer under reduced pressure and the residue was dissolved in THF (10 mL) before a saturated aqueous NH_4Cl-solution (10 mL) was added. This mixture was stirred for 1 h at rt. Subsequently the aqueous layer was extracted with Et_2O (3 × 20 mL), the organic layer was dried over $MgSO_4$, filtered and the solvent was removed under reduced pressure. The crude residue was triturated with pentane at −21 °C for 24 hours to give the desired product as white solid (1.5 g, 63%) with

spectroscopic data in accordance with the literature.[8] **^{1}H NMR** (400 MHz, $CDCl_3$) δ_H: 1.96 (2H, s, COC*H*$_2$), 0.18 (9H, s, Si(C*H*$_3$)$_3$); **^{13}C{^{1}H} NMR** (126 MHz, $CDCl_3$) δ_C: 179.9 (*C*O), 27.2 (CO*C*H$_2$), 1.5 (Si(*C*H$_3$)$_3$).

2-(Trimethylsilyl)butanoic acid (S7)

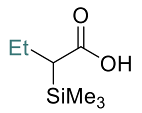

S7

Following General Procedure A, *N*,*N*-diisopropylamine (1.35 mL, 9.6 mmol, 2.1 equiv.), *n*-BuLi (3.8 mL, 9.6 mmol, 2.1 equiv.), 2-(trimethylsilyl) acetic acid 1 (600 mg, 4.5 mmol, 1.0 equiv.) and iodoethane (370 μL, 4.7 mmol, 1.05 equiv.) in THF (10 mL) gave, after purification by Biotage® Isolera™ 4 [SNAP KP-Sil 25 g, 36 mL min^{-1}, petrol: Et_2O (96:4 4 CV, 80:20 24 CV)], 2-(trimethylsilyl)butanoic acid as a white solid (374 mg, 52%) with spectroscopic data in accordance with the literature.[9] **^{1}H NMR** (500 MHz, $CDCl_3$) δ_H: 1.95 (1H, dd, *J* 11.5, 3.0, C*H*), 1.77 – 1.86 (1H, m, CH$_A$*H*$_B$), 1.46 – 1.54 (1H, m, C*H*$_A$H$_B$), 1.01 (3H, t, *J* 7.2, CH$_2$C*H*$_3$), 0.13 (9H, s, Si(C*H*$_3$)$_3$); **^{13}C{^{1}H} NMR** (126 MHz, $CDCl_3$) δ_C: 40.0 (*C*H), 182.0 (*C*O), 20.0 (*C*H$_2$), 15.1 (*C*H$_3$), −2.7 (Si(*C*H$_3$)$_3$).

2-(Trimethylsilyl)pentanoic acid (S8)

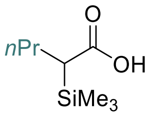

S8

Following General Procedure A, , *N*,*N*-diisopropylamine (1.35 mL, 9.6 mmol, 2.1 equiv.), *n*-BuLi (3.8 mL, 9.6 mmol, 2.1 equiv.), 2-(trimethylsilyl) acetic acid 1 (600 mg, 4.5 mmol, 1.0 equiv.) and 1-iodopropane (536 μL, 5.5 mmol, 1.2 equiv.) in THF (10 mL) gave, after trituration, 2-(trimethylsilyl)pentanoic acid as a white solid (628 mg, 80%) with spectroscopic data in accordance with the literature.[10] **^{1}H NMR** (400 MHz, $CDCl_3$) δ_H: 2.04 (1H, dd, *J* 11.6, 2.5, C*H*), 1.76 – 1.86 (1H, m, CHC*H*$_2$), 1.44 – 1.57 (1H, m, CHC*H*$_2$), 1.27 – 1.41 (2H, m, C*H*$_2$CH$_3$), 0.93 (3H, t, *J* 7.3, CH$_2$C*H*$_3$), 0.13 (9H, s, Si(C*H*$_3$)$_3$); **^{13}C{^{1}H} NMR** (101 MHz, $CDCl_3$) δ_C: 182.1 (*C*O), 37.8 (*C*(3)H), 28.7 (CH*C*H$_2$), 23.6 (*C*H$_2$CH$_3$), 13.9 (*C*H$_3$), −2.8 (Si(*C*H$_3$)$_3$).

2-(Trimethylsilyl)pent-4-enoic acid (S9)

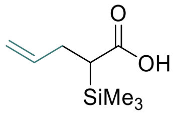

S9

Following General Procedure A, diisopropylamine (1.35 mL, 9.6 mmol, 2.1 equiv.), *n*-BuLi (3.8 mL, 9.6 mmol, 2.1 equiv.), 2-(trimethylsilyl) acetic acid 1 (600 mg, 4.5 mmol, 1.0 equiv.) and 3-bromoprop-1-ene (407 µL, 4.7 mmol, 1.05 equiv.) in THF (10 mL) gave, after trituration, 2-(trimethylsilyl)propanoic acid as a colourless oil (620 mg, 80%) with data in accordance with the literature.[6] **IR** v_{max} (film) 3001 (C-H), 912 (C-H), 1682 (C=O). **^{1}H NMR** (400 MHz, $CDCl_3$) δ_H: 5.82 – 5.90 (1H, m, C*H*=CH$_A$H$_B$), 5.06 – 5.11 (1H, m, CH=CH$_A$*H*$_B$), 4.99 – 5.01 (1H, m, CH=C*H*$_A$H$_B$), 2.25 – 2.58 (1H, m, CH$_A$*H*$_B$), 2.13 − 2.21 (2H, m, COC*H*, C*H*$_A$H$_B$), 0.15 (9H, s, Si(C*H*$_3$)$_3$); **^{13}C{^{1}H} NMR** (126 MHz, $CDCl_3$) δ_C: 181.4 (*C*O), 137.6 (*C*H=CH$_2$), 115.1 (CH=*C*H$_2$), 37.3 (CO*C*H), 30.4 (*C*H$_2$CH=CH$_2$), −2.7 (Si(*C*H$_3$)$_3$); **HRMS** (ESI$^+$) $C_8H_{16}O_2NaSi$ $[M+Na]^+$ found 195.0812, requires 195.0812 (+0.1 ppm).

2-(Trimethylsilyl)pent-4-ynoic acid (S10)

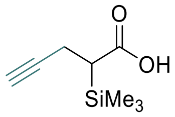

S10

Following General Procedure A, diisopropylamine (1.35 mL, 9.6 mmol, 2.1 equiv.), *n*-BuLi (3.8 mL, 9.6 mmol, 2.1 equiv.), 2-(trimethylsilyl) acetic acid 1 (600 mg, 4.5 mmol, 1.0 equiv.) and 3-bromoprop-1-yne (356 µL, 4.7 mmol, 1.05 equiv.) in THF (10 mL) gave, after triturated, 2-(trimethylsilyl)propanoic acid as a yellow oil (574 mg, 75%) with spectroscopic data in accordance with the literature.[Error! Reference source not found.] **IR** v_{max} (film) 3308 (C-H), 1684 (C=O). **^{1}H NMR** (400 MHz, $CDCl_3$) δ_H: 2.60 – 2.70 (1H, m, CC*H*), 2.31 – 2.42 (2H, m, C*H*$_2$), 2.00 – 2.01 (1H, m, COC*H*), 0.18 (9H, s, Si(C*H*$_3$)$_3$).

3-Phenyl-2-(trimethylsilyl)propanoic acid (S11)

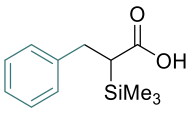

S11

Following General Procedure A, *N*,*N*-diisopropylamine (1.35 mL, 9.6 mmol, 2.1 equiv.), *n*-BuLi (3.8 mL, 9.6 mmol, 2.1 equiv.), 2-(trimethylsilyl) acetic acid 1 (600 mg, 4.5 mmol, 1.0 equiv.) and benzyl bromide

(558 μL, 4.7 mmol, 1.05 equiv.) in THF (10 mL) gave, after trituration, 2-(trimethylsilyl)propanoic acid as a white solid (789 mg, 79%) with spectroscopic data in accordance with the literature.[9] **^{1}H NMR** (400 MHz, $CDCl_3$) δ_H: 7.26 – 7.30 (2H, m, Ph*H*), 7.20 – 7.22 (3H, m, Ph*H*), 3.12 (1H, dd, *J* 14.5, 11.8 $PhCH_AH_B$), 2.76 (1H, dd, *J* 14.5, 3.1 $PhCH_AH_B$), 2.41 (1H, dd, *J* 11.6, 3.1, COC*H*), 0.18 (9H, s, $Si(CH_3)_3$); **^{13}C{^{1}H} NMR** (126 MHz, $CDCl_3$) δ_C: 181.1 (*C*O), 141.7 (Ph*C*(1)), 128.4 (Ph*C*(3,5)H), 128.2 (Ph*C*(2,6)H), 126.1 (Ph*C*(4)H), 39.8 (CO*C*H), 32.5 ($PhCH_2$), −2.7 ($Si(CH_3)_3$).

3-(*p*-Tolyl)-2-(trimethylsilyl)propanoic acid (S12)

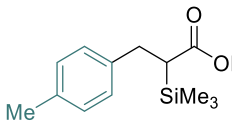

S12

Following General Procedure A, *N*,*N*-diisopropylamine (1.35 mL, 9.6 mmol, 2.1 equiv.), *n*-BuLi (3.8 mL, 9.6 mmol, 2.1 equiv.), 2-(trimethylsilyl) acetic acid 1 (600 mg, 4.5 mmol, 1.0 equiv.) and 1-(bromomethyl)-4-methylbenzene (770 μL, 5.5 mmol, 1.2 equiv.) in THF (10 mL) gave, after trituration, 3-(*p*-Tolyl)-2-(trimethylsilyl)propanoic acid as a white solid (670 mg, 63%) with data in accordance with the literature.[6] **mp** 78 – 81 °C; **IR** ν_{max} (film) 2955 (O−H), 1678 (C=O); **^{1}H NMR** (400 MHz, $CDCl_3$) δ_H: 7.09 – 7.13 (4H, m, Ar*H*), 3.09 (1H, dd, *J* 14.6, 11.8, $CHCH_2$), 2.73 (1H, dd, *J* 14.5, 3.0, $CHCH_2$), 2.39 (1H, dd, *J* 11.6, 3.1, $CHCH_2$), 2.34 (3H, s, $ArCH_3$), 0.19 (9H, s, $Si(CH_3)_3$); **^{13}C{^{1}H} NMR** (101 MHz, $CDCl_3$) δ_C: 181.3 (*C*O), 138.7 (Ar*C*(1)), 135.5 (Ar*C*(4)), 119.1 (Ar*C*(3,5)H), 128.0 (Ar*C*(2,6)H), 39.9 ($CHCH_2$), 31.8 ($CHCH_2$), 21.0 ($ArCH_3$), −2.7 ($Si(CH_3)_3$); **HRMS** (ESI$^+$) $C_{13}H_{20}O_2NaSi$ [M+Na]$^+$ found 259.1118, requires 259.1125 (−2.6 ppm).

3-(4-Fluorophenyl)-2-(trimethylsilyl)propanoic acid (S13)

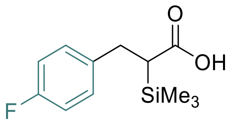

S13

Following General Procedure A, *N*,*N*-diisopropylamine (1.35 mL, 9.6 mmol, 2.1 equiv.), *n*-BuLi (3.8 mL, 9.6 mmol, 2.1 equiv.), 2-(trimethylsilyl) acetic acid 1 (600 mg, 4.5 mmol, 1.0 equiv.) and 1-(bromomethyl)-4-fluorobenzene (680 μL, 5.5 mmol, 1.2 equiv.) in THF (10 mL) gave, after trituration, 3-(4-fluorophenyl)-2-(trimethylsilyl)propanoic acid as a white solid (649 mg, 60%) with data in accordance with the literature.[6] **mp** 64 – 67 °C; **IR** ν_{max} (film) 2957 (O−H), 1680 (C=O); **^{1}H NMR** (400 MHz, $CDCl_3$) δ_H: 7.14 – 7.18 (2H, m, ArC(2,6)*H*), 6.93 – 6.99 (2H, m, ArC(3,5)*H*), 3.07 (1H, dd, *J* 14.6, 11.9, $CHCH_2$), 2.73 (1H, dd, *J* 14.6, 3.2, $CHCH_2$), 2.36 (1H, dd, *J* 11.9, 3.2, $CHCH_2$), 0.18 (9H, s, $Si(CH_3)_3$); **^{19}F NMR** (377 MHz,

$CDCl_3$) δ_F: −117.3 (s); **^{13}C{^{1}H} NMR** (101 MHz, $CDCl_3$) δ_C: 181.1 (*C*O), 161.5 (d, *J* 244.3, Ar*C*(4)), 137.3 (Ar*C*(1)), 129.6 (d, *J* 7.8, Ar*C*(2,6)), 115.1 (d, *J* 21.1, Ar*C*(3,5)), 31.5 (CH*C*H_2), 40.1 (*C*HCH_2), −2.8 (Si(*C*H_3)$_3$); **HRMS** (ESI$^+$) $C_{12}H_{17}O_2FNaSi$ [M+Na]$^+$ found 263.0869, requires 263.0874 (−1.9 ppm).

3-(Naphthalen-2-yl)-2-(trimethylsilyl)propanoic acid (S14)

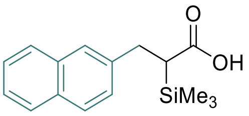

S14

Following General Procedure A, diisopropylamine (1.35 mL, 9.6 mmol, 2.1 equiv.), *n*-BuLi (3.8 mL, 9.6 mmol, 2.1 equiv.), 2-(trimethylsilyl) acetic acid 1 (600 mg, 4.5 mmol, 1.0 equiv.) and 2-(bromomethyl)naphthalene (1.04 g, 4.7 mmol, 1.05 equiv.) in THF (10 mL) gave, after trituration, 2-(trimethylsilyl)propanoic acid as a white solid (771 mg, 63%) with spectroscopic data in accordance with the literature.[11] **^{1}H NMR** (400 MHz, $CDCl_3$) δ_H: 7.74 – 7.82 (3H, m, Ar*H*), 7.66 (1H, s, Ar*H*), 7.40 – 7.49 (2H, m, Ar*H*), 7.34 (1H, dd, *J* 8.4, 1.7, Ar*H*), 3.27 (1H, dd, *J* 14.7, 11.8 ArCH$_A$*H*$_B$), 2.92 (1H, dd, *J* 14.6, 3.1 ArC*H*$_A$H$_B$), 2.51 (1H, dd, *J* 11.6, 3.1, COC*H*), 0.19 (9H, s, Si(C*H*$_3$)$_3$); **^{13}C{^{1}H} NMR** (126 MHz, $CDCl_3$) δ_C: 180.5 (*C*O), 139.2 (Ar*C*(2)), 133.6 (Ar*C*(9)), 132.1 (Ar*C*(10)), 128.0 (Ar*C*(1)), 127.6 (Ar*C*(8)), 127.6 (Ar*C*(5)), 127.0 (Ar*C*(3)), 126.3 (Ar*C*(4)), 125.9 (Ar*C*(7)), 125.2 (Ar*C*(6)), 39.6 (CO*C*H), 32.4 (Ph*C*H_2), −2.7 (Si(*C*H_3)$_3$).

2-Phenyl-2-(trimethylsilyl)acetic acid (S15)

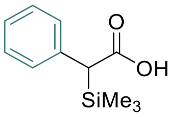

S15

Following a procedure reported by Cheng *et al*,[12] *n*-BuLi (15 mL, 12.0 mmol, 1.2 equiv.) was added to a solution of benzyltrimethylsilane (1.64 g, 10.0 mmol, 1.0 equiv.) in anhydrous THF (25 mL) dropwise over 40 min at 0 °C and the mixture was stirred at rt overnight. Carbon dioxide gas was bubbled into the solution which turned orange-brown until the color disappeared. The reaction was quenched by the addition of HCl (1 M) and the pH adjusted to pH=2, The organic layer was separated and the aqueous solution extracted with Et_2O (3 × 30 mL), the combined organic layers was dried over $MgSO_4$, filtered and the solvent was removed under reduced pressure to give 2-phenyl-2-(trimethylsilyl)acetic acid as a white solid (832 mg, 40%) with spectroscopic data in accordance with the literature.[12] **^{1}H NMR** (500 MHz, $CDCl_3$) δ_H: 7.31 – 7.36 (1H, m, Ph*H*), 7.21 – 7.24 (1H, m, Ph*H*), 3.50 (1H, s, C*H*Ph), 0.12 (9H, s,

$Si(CH_3)_3$); **$^{13}C\{^1H\}$ NMR** (126 MHz, $CDCl_3$) δ_C: 179.7 (*C*O), 128.5 (Ph*C*(2,6)H), 128.2 (Ph*C*(3,5)H), 125.8 (Ph*C*(4)H), 46.4 (*C*H(CH_3)$_2$), −2.4 (Si(CH_3)$_3$).

3-methyl-2-(trimethylsilyl)butanoic acid (45)

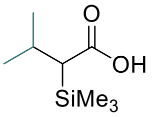

45

Following General Procedure B, diisopropylamine (1.35 mL, 9.6 mmol, 2.1 equiv.), *n*-BuLi (3.8 mL, 9.6 mmol, 2.1 equiv.), 2-(trimethylsilyl) acetic acid (600 mg, 4.5 mmol, 1.0 equiv.), 2-bromopropane (2.4 mL, 22.5 mmol, 5.0 equiv.) in THF (10 mL) leave for overnight gave, after triturated, 2-(trimethylsilyl)propanoic acid as a colorless oil (470 mg, 60%) with data in accordance with the literature.[6] **IR** ν_{max} (film) 1680 (C=O); **^1H NMR** (300 MHz, $CDCl_3$) δ_H: 2.11 – 2.23 (1H, m, C*H*(CH_3)$_2$), 1.81 (1H, d, *J* 10.0, C*H*CO), 1.05 (6H, dd, *J* 9.2, 6.4, CH(C*H*$_3$)$_2$), 0.17 (9H, s, Si(C*H*$_3$)$_3$); **$^{13}C\{^1H\}$ NMR** (126 MHz, $CDCl_3$) δ_C: 181.8 (*C*O), 46.4 (CO*C*H), 28.1 (*C*H(CH_3)$_2$), 23.5 (CH(C_BH_3)$_2$), 22.7 (CH(C_AH_3)$_2$), –1.5 (Si(*C*H$_3$)$_3$); **HRMS** (ESI$^+$) $C_8H_{17}O_2Si$ [M−H]$^-$ found 173.0994, requires 173.1003 (–5.0 ppm).

5. Synthesis of α-ketophosphonates

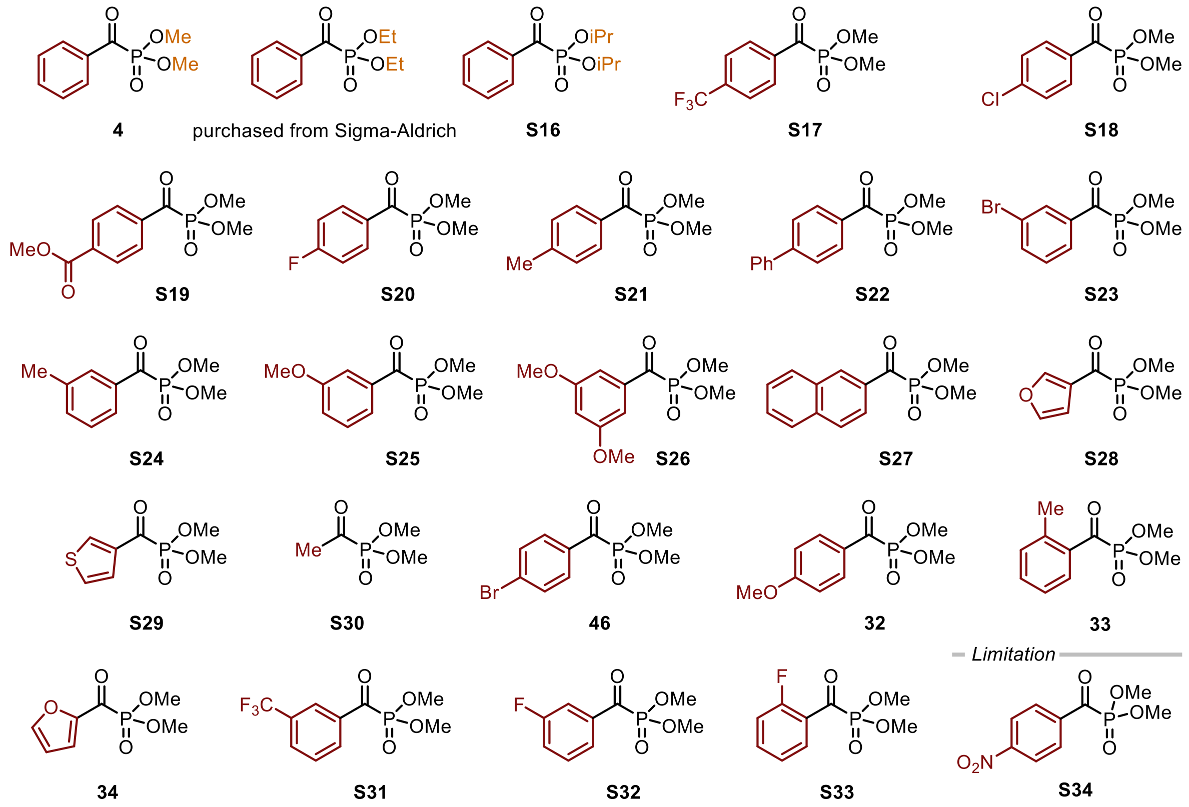

Dimethyl benzoylphosphonate (4)

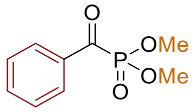

4

Following General Procedure C, trimethyl phosphite (1.18 mL, 10 mmol) was added dropwise into benzoyl chloride (1.16 mL, 10 mmol) at 0 °C, then 3 h at rt, after distillation gave dimethyl benzoylphosphonate (1.92 g, 90%) as a yellow oil with spectroscopic data in accordance with the literature.[5] **IR** ν_{max} (film) 1653 (C=O), 1256 (P=O), 1018 (P−O). **^{1}H NMR** (400 MHz, $CDCl_3$) δ_H: 8.19 – 8.22 (2H, m, PhC(2,6)*H*), 7.58 – 7.62 (1H, m, PhC(4)*H*), 7.45 – 7.49 (2H, m, PhC(3,5)*H*), 3.87 (6H, d, *J* 10.8, OCH_3).

Diisopropyl benzoylphosphonate (S16)

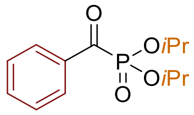

S16

Following General Procedure C, triisopropyl phosphite (2.28 mL, 10 mmol) was added dropwise into benzoyl chloride (1.16 mL, 10 mmol) at 0 °C, then 4 h at rt, after distillation gave ditriisopropyl benzoylphosphonate (1.92 g, 90%) as a yellow oil with spectroscopic data in accordance with the literature.[5] **IR** ν_{max} (film) 1250 (P=O), 980 (P−O). **^{1}H NMR** (400 MHz, $CDCl_3$) δ_H: 8.28 – 8.31 (2H, m, PhC(2,6)*H*), 7.62 – 7.66 (1H, m, PhC(4)*H*), 7.50 – 7.54 (2H, m, PhC(3,5)*H*), 4.80 – 4.91 (2H, m, C*H*(CH_3)$_2$), 1.40 (12H, dd, *J* 6.2, 3.5, CH(C*H*$_3$)$_2$); **^{31}P NMR** (162 MHz, $CDCl_3$) δ_P: −2.7.

Dimethyl (4-(trifluoromethyl)benzoyl)phosphonate (S17)

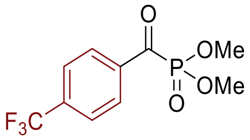

S17

Following General Procedure C, trimethyl phosphite (1.18 mL, 10 mmol) was added dropwise into 4-trifluoromethylbenzoyl chloride (1.49 mL, 10 mmol) at 0 °C, then 3 h at rt, after distillation gave dimethyl (4-trifluoromethylbenzoyl)phosphonate (2.23 g, 80%) as a yellow oil with spectroscopic data in accordance with the literature.[13] **IR** ν_{max} (film) 1259 (P=O), 1028 (P−O). **^{1}H NMR** (400 MHz, $CDCl_3$) δ_H: 8.38 (2H, d, *J* 8.2, ArC(3,5)*H*), 7.80 (2H, d, *J* 8.4, ArC(2,6)*H*), 3.96 (6H, d, *J* 10.8, OC*H*$_3$); **^{31}P NMR** (162 MHz, $CDCl_3$) δ_P: −0.2. *Note: over time this product began to decompose to the analogous byproduct as described on page 30.*

Dimethyl (4-chlorobenzoyl)phosphonate (S18)

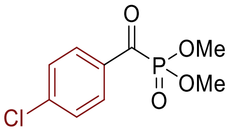

S18

Following General Procedure C, trimethyl phosphite (1.18 mL, 10 mmol) was added dropwise into 4-chloromethylbenzoyl chloride (1.28 mL, 10 mmol) at 0 °C, then rt overnight. After column chromatography gave dimethyl (4-trifluoromethylbenzoyl)phosphonate (2.23 g, 90%) as a yellow oil with spectroscopic data in accordance with the literature.[14] **IR** ν_{max} (film) 1584 (C=O), 1256 (P=O), 1013

(P−O). **¹H NMR** (400 MHz, $CDCl_3$) δ_H: 8.21 – 8.24 (2H, m, ArC(3,5)*H*),), 7.49 – 7.53 (2H, m, ArC(2,6)*H*), 3.94 (6H, d, *J* 10.8, OCH_3; **³¹P NMR** (162 MHz, $CDCl_3$) δ_P: 0.3.

Methyl 4-((dimethoxyphosphoryl)carbonyl)benzoate (S19)

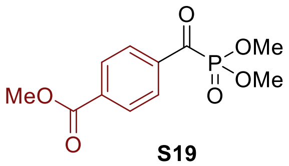

S19

Following General Procedure C, trimethyl phosphite (0.59 mL, 5 mmol) was added dropwise into methyl 4-(chlorocarbonyl)benzoate (1.0 g, 5 mmol) at 0 °C, then 3 h at rt, after distillation gave methyl 4-((dimethoxyphosphoryl)carbonyl)benzoate (748.6 mg, 55% in 78% purity with 22% of trimethyl phosphite) as a pale yellow solid with spectroscopic data in accordance with the literature.[15] **IR** v_{max} (film) 1721 (C=O), 1258 (P=O), 1016 (P−O). **¹H NMR** (400 MHz, $CDCl_3$) δ_H: 8.31 – 8.34 (2H, m, ArC(2,6)*H*), 8.17 – 8.20 (2H, m, ArC(3,5)*H*), 3.98 (3H, s, COOCH_3), 3.96 (6H, d, *J* 10.9, OCH_3); **³¹P NMR** (162 MHz, $CDCl_3$) δ_P: 0.1.

Dimethyl (4-fluorobenzoyl)phosphonate (S20)

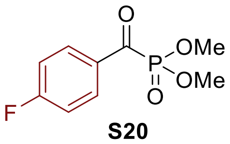

S20

Following General Procedure C, trimethyl phosphite (1.18 mL, 10 mmol) was added dropwise into 4-fluorobenzoyl chloride (1.20 mL, 10 mmol) at 0 °C, then 3 h at rt, after distillation gave dimethyl (4-fluorobenzoyl)phosphonate (2.18 g, 94%) as a yellow oil with spectroscopic data in accordance with the literature.[16] **IR** v_{max} (film) 1593 (C=O), 1231 (P=O), 1020 (P−O). **¹H NMR** (400 MHz, $CDCl_3$) δ_H: 8.28 – 8.33 (2H, m, ArC(3,5)*H*), 7.16 – 7.21 (2H, m, ArC(2,6)*H*), 3.91 (6H, d, *J* 10.8, OCH_3); **³¹P NMR** (162 MHz, $CDCl_3$) δ_P: −0.5.

Dimethyl (4-methylbenzoyl)phosphonate (S21)

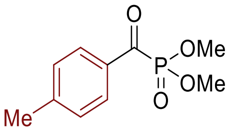

S21

Following General Procedure C, trimethyl phosphite (1.18 mL, 10 mmol) was added dropwise into 4-methylbenzoyl chloride (1.32 mL, 10 mmol) at 0 °C, then 3 h at rt, after distillation gave dimethyl (4-

methylbenzoyl)phosphonate (2.10 g, 92%) as a yellow oil with spectroscopic data in accordance with the literature.[17] **IR** ν_{max} (film) 1601 (C=O), 1256 (P=O), 1018 (P−O). **^{1}H NMR** (400 MHz, $CDCl_3$) δ_H: 8.16 – 8.19 (2H, m, ArC(3,5)*H*), 7.32 – 7.34 (2H, m, ArC(2,6)*H*), 3.93 (6H, d, *J* 10.9, OC*H*$_3$), 2.45 (3H, s, ArC*H*$_3$); **^{31}P NMR** (162 MHz, $CDCl_3$) δ_P: −1.1.

Dimethyl ([1,1'-biphenyl]-4-carbonyl)phosphonate (S22)

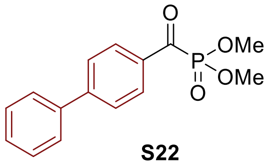

S22

Following General Procedure C, trimethyl phosphite (1.18 mL, 10 mmol) was added dropwise into [1,1'-biphenyl]-4-carbonyl chloride (2.17 g, 10 mmol) at 0 °C, then 16 h at rt, after column chromatography gave dimethyl ([1,1'-biphenyl]-4-carbonyl)phosphonate (1.60 g, 55%) as a pale yellow solid with spectroscopic data in accordance with the literature.[18] **IR** ν_{max} (film) 1597 (C=O), 1256 (P=O), 1022 (P−O). **^{1}H NMR** (400 MHz, $CDCl_3$) δ_H: 8.34 – 8.37 (2H, m, ArC(3,5)*H*), 7.74 – 7.77 (2H, m, ArC(3,5)*H*), 7.63 – 7.66 (2H, m, PhC(2,6)*H*), 7.42 – 7.52 (3H, m, PhC(3,4,5)*H*), 3.97 (6H, d, *J* 10.9, OC*H*$_3$); **^{31}P NMR** (162 MHz, $CDCl_3$) δ_P: 0.9.

Dimethyl (3-bromobenzoyl)phosphonate (S23)

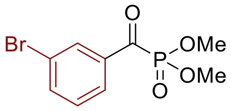

S23

Following General Procedure C, trimethyl phosphite (1.18 mL, 10 mmol) was added dropwise into 3-bromobenzoyl chloride (1.32 mL, 10 mmol) at 0 °C, then 3 h at rt, after distillation gave dimethyl (3-bromobenzoyl)phosphonate (2.61 g, 89%) as a yellow oil with spectroscopic data in accordance with the literature.[19] **IR** ν_{max} (film) 1256 (P=O), 1018 (P−O). **^{1}H NMR** (400 MHz, $CDCl_3$) δ_H: 8.33 (1H, t, *J* 1.7, ArC(2)*H*), 8.27 (1H, ddd, *J* 7.8, 1.6, 1.1, ArC(4)*H*), 7.80 (1H, dq, *J* 8.0, 1.0, ArC(6)*H*), 7.41 (1H, td, *J* 7.9, 0.8, ArC(5)*H*), 3.95 (6H, d, *J* 10.8, OC*H*$_3$); **^{31}P NMR** (162 MHz, $CDCl_3$) δ_P: −0.2.

Dimethyl (3-methylbenzoyl)phosphonate (S24)

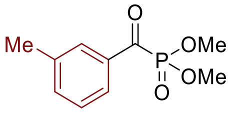

S24

Following General Procedure C, trimethyl phosphite (1.18 mL, 10 mmol) was added dropwise into 3-methylbenzoyl chloride (1.32 g, 10 mmol) at 0 °C, then 3 h at rt, after distillation gave dimethyl (3-methylbenzoyl)phosphonate (1.87 g, 82%) as a yellow oil with spectroscopic data in accordance with the literature.[20] **IR** ν_{max} (film) 1250 (P=O), 1018 (P−O). **^{1}H NMR** (400 MHz, $CDCl_3$) δ_H: 8.09 – 8.11 (1H, m, ArC(6)*H*), 8.04 (1H, s, ArC(2)*H*), 7.47 – 7.50 (1H, m, ArC(4)*H*), 7.43 (1H, t, *J* 7.6, ArC(5)*H*, 3.94 (6H, d, *J* 10.8, OCH_3), 2.42 (3H, s, ArCH_3); **^{31}P NMR** (162 MHz, $CDCl_3$) δ_P: 0.9.

Dimethyl (3-methoxybenzoyl)phosphonate (S25)

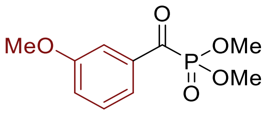

S25

Following General Procedure C, trimethyl phosphite (1.18 mL, 10 mmol) was added dropwise into 3-methoxybenzoyl chloride (1.40 mL, 10 mmol) at 0 °C, then 3 h at rt, after distillation gave dimethyl (3-methoxybenzoyl)phosphonate (2.17 g, 89%) as a yellow oil with spectroscopic data in accordance with the literature.[21] **IR** ν_{max} (film) 1653 (C=O), 1261 (P=O), 1024 (P−O). **^{1}H NMR** (400 MHz, $CDCl_3$) δ_H: 7.96 (1H, ddd, *J* 7.7, 1.5, 1.0, PhC(5)*H*), 7.69 (1H, dd, *J* 2.6, 1.6, ArC(6)*H*), 7.43 – 7.47 (1H, m, ArC(2)*H*), 7.21 (1H, ddd, *J* 8.2, 2.7, 1.0, ArC(4)*H*), 3.94 (6H, d, *J* 10.8, CH(CH_3)$_2$), 3.88 (3H, s, ArOCH_3); **^{31}P NMR** (162 MHz, $CDCl_3$) δ_P: −2.7.

Dimethyl (3,5-dimethoxybenzoyl)phosphonate (S26)

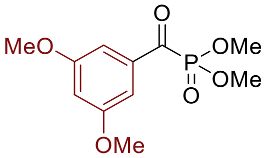

S26

Following General Procedure C, trimethyl phosphite (1.18 mL, 10 mmol) was added dropwise into 3, 5-dimethoxybenzoyl chloride (2.01 g, 10 mmol) at 0 °C, then 3 h at rt, after distillation gave dimethyl (3, 5-dimethoxybenzoyl)phosphonate (2.46 g, 90%) as a yellow oil with spectroscopic data in accordance with the literature.[22] **IR** ν_{max} (film) 1589 (C=O), 1265 (P=O), 1022 (P−O). **^{1}H NMR** (400 MHz, $CDCl_3$) δ_H: 7.40 (2H, d, *J* 2.3, ArC(2, 6)*H*), 6.92 (1H, t, *J* 2.3, ArC(4)*H*), 3.90 (6H, d, *J* 10.9, POCH_3), 3.83 (6H, s, ArOCH_3); **^{31}P NMR** (162 MHz, $CDCl_3$) δ_P: 0.6.

Dimethyl (2-naphthoyl)phosphonate (S27)

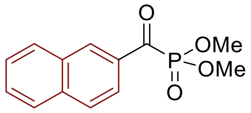

S27

Following General Procedure C, trimethyl phosphite (1.18 mL, 10 mmol) was added dropwise into 2-naphthoyl chloride (1.90 g, 10 mmol) at 0 °C, then 3 h at rt, after distillation gave dimethyl dimethyl (2-naphthoyl)phosphonate (2.43 g, 92%) as a yellow oil with spectroscopic data in accordance with the literature.[15] **IR** v_{max} (film) 1622 (C=O), 1269 (P=O), 1018 (P−O). **^{1}H NMR** (400 MHz, $CDCl_3$) δ_H: 9.05 (1H, d, *J* 0.8, Ar*H*), 8.07 – 8.14 (2H, m, Ar*H*), 7.89 – 7.95 (2H, m, Ar*H*), 7.66 – 7.70 (1H, m, Ar*H*), 7.59 – 7.63 (1H, m, Ar*H*), 3.98 (6H, d, *J* 10.8, OCH_3); **^{31}P NMR** (162 MHz, $CDCl_3$) δ_H: 0.9.

Dimethyl (furan-3-carbonyl)phosphonate (S28)

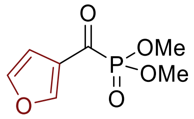

S28

Following General Procedure C, trimethyl phosphite (0.90 mL, 7.66 mmol) was added dropwise into furan-3-carbonyl chloride (1.0 g, 7.66 mmol) at 0 °C, then 4 h at rt, after column chromatography gave dimethyl (furan-3-carbonyl)phosphonate (1.08 g, 69%) as a light brown oil with spectroscopic data in accordance with the literature.[23] **IR** v_{max} (film) 1636 (C=O), 1258 (P=O), 1015 (P−O). **^{1}H NMR** (400 MHz, $CDCl_3$) δ_H: 8.69 – 8.69 (1H, m, ArC(2)*H*), 7.46 – 7.48 (1H, m, ArC(5)*H*), 6.81 – 6.82 (1H, m, ArC(4)*H*, 3.87 (6H, d, *J* 10.9, OCH_3); **^{31}P NMR** (162 MHz, $CDCl_3$) δ_P: −0.9.

Dimethyl (thiophene-3-carbonyl)phosphonate (S29)

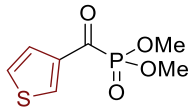

S29

Following General Procedure C, trimethyl phosphite (0.80 mL, 6.82 mmol) was added dropwise into thiophene-3-carbonyl chloride (1.0 g, 6.82 mmol) at 0 °C, then 4 h at rt, after column chromatography gave dimethyl (furan-3-carbonyl)phosphonate (400 mg, 27%) as a light brown oil with spectroscopic data in accordance with the literature.[23] **IR** v_{max} (film) 1636 (C=O), 1258 (P=O), 1016 (P−O). **^{1}H NMR** (400 MHz, $CDCl_3$) δ_H: 8.87 (1H, dd, *J* 2.9, 1.2, ArC(2)*H*), 7.68 (1H, dt, *J* 5.2, 1.2, ArC(5)*H*), 7.37 – 7.39 (1H, m, ArC(4)*H*, 3.92 (6H, d, *J* 10.8, OCH_3); **^{31}P NMR** (162 MHz, $CDCl_3$) δ_P: 0.2.

Dimethyl acetylphosphonate (S30)

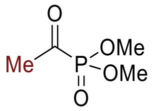

S30

Following General Procedure C, trimethyl phosphite (1.18 mL, 10 mmol) was added dropwise into acetyl chloride (0.71 mL, 10 mmol) at 0 °C, then 16 h at rt, after distillation gave dimethyl acetylphosphonate (1.34 g, 88%) as a colourless oil with spectroscopic data in accordance with the literature.[24] **IR** ν_{max} (film) 1256 (P=O), 1016 (P−O). **^{1}H NMR** (400 MHz, $CDCl_3$) δ_H: 3.87 (6H, d, *J* 10.7, OC*H*$_3$), 2.49 (3H, d, J 5.3, COC*H*$_3$); **^{31}P NMR** (162 MHz, $CDCl_3$) δ_P: −1.1.

Dimethyl (4-bromobenzoyl)phosphonate (46)

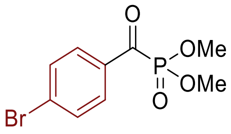

46

Following General Procedure C, trimethyl phosphite (1.18 mL, 10 mmol) was added dropwise into 4-bromobenzoyl chloride (1.32 mL, 10 mmol) at 0 °C, then 3 h at rt, after distillation gave dimethyl (4-bromobenzoyl)phosphonate (2.58 g, 88%) as a yellow oil with spectroscopic data in accordance with the literature.[17] **IR** ν_{max} (film) 1580 (C=O), 1256 (P=O), 1020 (P−O). **^{1}H NMR** (400 MHz, $CDCl_3$) δ_H: 8.13 – 8.16 (2H, m, ArC(2,6)*H*), 7.67 – 7.70 (2H, m, ArC(3,5)*H*), 3.94 (6H, d, *J* 10.9, OC*H*$_3$); **^{31}P NMR** (162 MHz, $CDCl_3$) δ_P: −0.3.

Dimethyl (4-methoxybenzoyl)phosphonate (32)

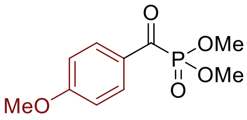

32

Following General Procedure C, trimethyl phosphite (1.18 mL, 10 mmol) was added dropwise into 4-methoxybenzoyl chloride (1.35 mL, 10 mmol) at 0 °C, then 3 h at rt, after distillation gave dimethyl (4-methoxybenzoyl)phosphonate (2.34 g, 96%) as a yellow oil with spectroscopic data in accordance with the literature.[25] **IR** ν_{max} (film) 1589 (C=O), 1256 (P=O), 1015 (P−O). **^{1}H NMR** (400 MHz, $CDCl_3$) δ_H: 8.26 – 8.29 (2H, m, ArC(3,5)*H*), 6.97 – 7.00 (2H, m, ArC(2,6)*H*), 3.92 (6H, d, *J* 10.8, OC*H*$_3$), 3.90 (3H, s, ArOC*H*$_3$); **^{31}P NMR** (162 MHz, $CDCl_3$) δ_P: −1.4.

Dimethyl (2-methylbenzoyl)phosphonate (33)

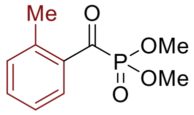

33

Following General Procedure C, trimethyl phosphite (1.18 mL, 10 mmol) was added dropwise into 2-methylbenzoyl chloride (1.32 mL, 10 mmol) at 0 °C, then 3 h at rt, after distillation gave dimethyl (2-methylbenzoyl)phosphonate (2.00 g, 88%) as a yellow oil with spectroscopic data in accordance with the literature.[26] **IR** v_{max} (film) 1651 (C=O), 1254 (P=O), 1018 (P−O). **^{1}H NMR** (400 MHz, $CDCl_3$) δ_H: 8.45 (1H, dd, *J* 7.9, 1.2, ArC(2)*H*), 7.50 (1H, td, *J* 7.5, 1.2, ArC(5)*H*), 7.37 – 7.40 (1H, m, ArC(4)*H*), 7.30 – 7.32 (1H, m, ArC(3)*H*), 3.93 (6H, d, *J* 1.8, OC*H*$_3$); **^{31}P NMR** (162 MHz, $CDCl_3$) δ_P: 0.9.

Dimethyl (furan-2-carbonyl)phosphonate (34)

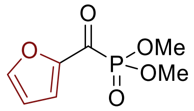

34

Trimethyl phosphite (1.18 mL, 10 mmol) was added dropwise into furan-3-carbonyl chloride (0.99 g, 10 mmol) in toluene (2 mL) at −48 °C, then warm to rt for 12 hours, after column chromatography gave dimethyl (furan-2-carbonyl)phosphonate (714 mg, 35%) as a pale yellow oil with spectroscopic data in accordance with the literature.[23] **IR** v_{max} (film) 1628 (C=O), 1254 (P=O), 1018 (P−O). **^{1}H NMR** (400 MHz, $CDCl_3$) δ_H: 7.87 (1H, dd, *J* 3.7, 0.6, ArC(5)*H*), 7.76 – 7.77 (1H, m, ArC(3)*H*), 6.64 (1H, dd, *J* 3.7, 1.7, ArC(4)*H*, 3.91 (6H, d, *J* 10.9, OC*H*$_3$); **^{31}P NMR** (162 MHz, $CDCl_3$) δ_P: −0.5.

Dimethyl (3-(trifluoromethyl)benzoyl)phosphonate (S31)

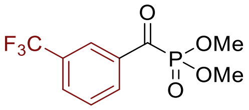

S31

Following General Procedure C with 3-(trifluoromethyl)benzoyl chloride (1.51 mL, 10 mmol) and trimethyl phosphite (1.18 mL, 10 mmol) at 0 °C for 0 min gave an inseparable mixture of the title compound **S31** (2.47 g, 88%) and its dimeric adduct (675 mg, 12%, as described on page 30) as a yellow oil. The product was used immediately in catalysis, accounting for purity. **IR** v_{max} (film) 1663 (C=O), 1261 (P=O), 1028 (P-O)**;** **HRMS** [ESI]$^+$ $C_9H_{10}O_4FNaP$ [M+Na]$^+$ found 255.0189, requires 255.0193 (−1.57 ppm); **^{1}H NMR** (400 MHz, $CDCl_3$): δ_H: 8.53-8.48 (1H, m, C(4)*H*), 8.47-8.44 (1H, m, C(2)*H*), 7.93-7.88 (1H, m, C(6)*H*), 7.71-7.66 (1H, m, C(5)*H*), 3.95 (d, *J* 10.9, POC*H*$_3$); **^{13}C{^{1}H} NMR** (101 MHz, $CDCl_3$): δ_C: 197.7 (d, *J* 179.7,

C=O), 135.9 (d, *J* 64.6, *C*(3)), 133.3 (*C*(4)H), 131.1 (dd, *J* 7.2, 3.6, *C*(5)H), 129.7 (*C*(2)H), 126.2 (dd, *J* 7.3, 3.6, CF_3), 54.4 (d, *J* 7.5, OCH_3); **^{19}F{^{1}H} NMR** (376 MHz, $CDCl_3$): δ_F: − 62.98 ppm; **^{31}P{^{1}H} NMR** (162 MHz, $CDCl_3$): δ_P: −0.3 ppm. *Note: over time this product began to decompose to the analogous byproduct as described on page 30, and hence characterisation data is has been removed from the SI due to the dimeric adduct impurity).*

Dimethyl (3-fluorobenzoyl)phosphonate (S32)

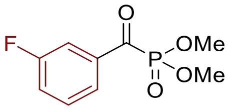

S32

Following General Procedure C with 3-fluorobenzoyl chloride (1.22 mL, 10 mmol) and trimethyl phosphite (1.18 mL, 10 mmol) for 24 h, then vacuum distillation gave the title compound **XX** (1.10 g, 48%) as a yellow oil. **IR** ν_{max} (film) 1657 (C=O),z 1252 (P=O), 1020 (P-O); **HRMS** [ESI]$^+$ $C_9H_{10}O_4FNaP$ [M+Na]$^+$ found 255.0189, requires 255.0193 (−1.57 ppm). **^{1}H NMR** (400 MHz, $CDCl_3$): δ_H: 8.07 (1H, ddd, *J* 7.8, 1.7, 1.1, ArC(4)*H*), 7.86 (1H, ddd, *J* 9.1, 2.7, 1.6, ArC(2)*H*), 7.48 (1H, dddd, *J* 16.0, 7.8, 5.4, 1.1, ArC(5)*H*), 7.32 (1H, dddd, *J* 8.9, 8.2, 2.8, 0.9, ArC(6)*H*), 3.92 (6H, d, *J* 10.9, POCH_3). **^{13}C{^{1}H} NMR** (101 MHz, $CDCl_3$): δ_C: 197.5 (dd, *J* 178.9, 2.1, *C*=O), 162.7 (dd, *J* 249.2, 2.4, Ar*C*(1)F), 137.3 (dd, *J* 64.7, 6.5, Ar*C*(3)), 130.7 (d, *J* 7.5, Ar*C*(5)H), 126.0 (d, *J* 3.1, Ar*C*(4)H), 122.1 (d, *J* 7.2, Ar*C*(6)H), 115.9 (dd, *J* 22.8, 2.3, Ar*C*(2)H), 54.3 (d, *J* 7.5, OCH_3). **^{19}F{^{1}H} NMR** (376 MHz, $CDCl_3$): δ_F: − 110.75 (d, *J* 3.2); **^{31}P {^{1}H} NMR** (162 MHz, $CDCl_3$): δ_P: 0.04 (d, *J* 3.5) ppm.

Dimethyl (2-fluorobenzoyl)phosphonate (S33)

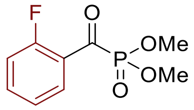

S33

Following General Procedure C with 2-fluorobenzoyl chloride (119 mL, 10 mmol) and trimethyl phosphite (1.18 mL, 10 mmol) for 2.5 h, then vacuum distillation gave the title compound **XX** (1.05 g, 45%) as a yellow oil. **IR** ν_{max} (film): 1609 (C=O), 1261 (P=O), 1030 (P-O); **HRMS** [ESI]$^+$ $C_9H_{10}O_4FNaP$ [M+Na]$^+$ found 255.0193, requires 255.0183 (−3.90 ppm); **^{1}H NMR** (400 MHz, $CDCl_3$): δ_H: 8.16 (1H, ddd, *J* 7.6, 7.1, 1.8, ArC(3)*H*), 7.61 (1H, dddd, *J* 8.3, 7.4, 5.0, 1.8, ArC(5)*H*), 7.29 (1H, ddd, *J* 7.6, 7.6, 1.0 ArC(4)*H*), 7.18 (1H, ddd, *J* 10.7, 8.3, 1.2, ArC(6)*H*), 3.93 (6H, d, *J* 10.9, POCH_3); **^{13}C{^{1}H} NMR** (101 MHz, $CDCl_3$): δ_C: 197.5 (d, *J* 184.6, *C*=O), 161.5 (dd, *J* 262.8, 3.7, Ar*C*(1)F), 136.2 (d, *J* 9.3, Ar*C*(5)H), 131.8 (Ar*C*(3)H), 125.1 (d, *J* 9.8, Ar*C*(2)), 124.5 (d, *J* 3.9, Ar*C*(4)H), 117.1 (dd, *J* 21.4, 2.1, Ar*C*(6)H), 54.4 (d, *J* 7.5, OCH_3); **^{19}F{^{1}H} NMR** (376 MHz, $CDCl_3$): δ_F: −110.7; **^{31}P{^{1}H} NMR** (162 MHz, $CDCl_3$): δ_P: −0.6.

Attempted synthesis of (4-nitrobenzoyl)phosphonate (S34)

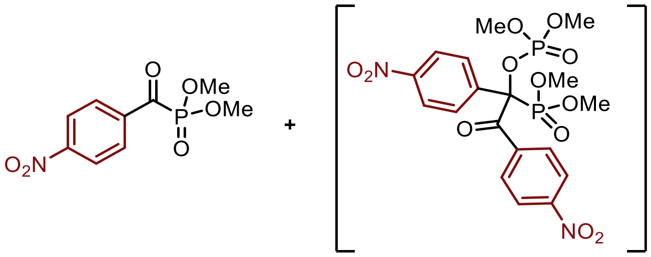

S34 **S35**

Following General Procedure C with 4-nitrobenzoyl chloride (1.86 g, 10 mmol) and trimethyl phosphite (1.18 mL, 10 mmol) for 0 min afforded the title compound **S34** (0 mg, 0%) and **S35** (360 mg, 70%) as a dark yellow oil. Unfortunately, this was unable to be isolated with acceptable purity, but collected data were in line with the literature which originally reported this strange reactivity.[27]

6. Catalysis Products – C(3)-Alkyl

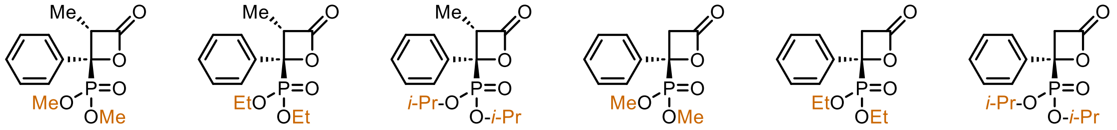

5 11 12 13 14 15

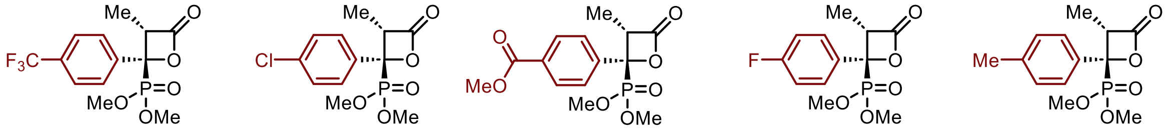

16 17 18 19 20

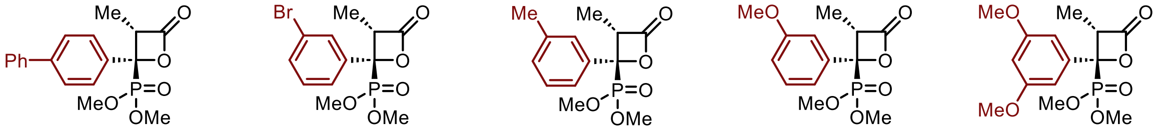

21 22 23 24 25

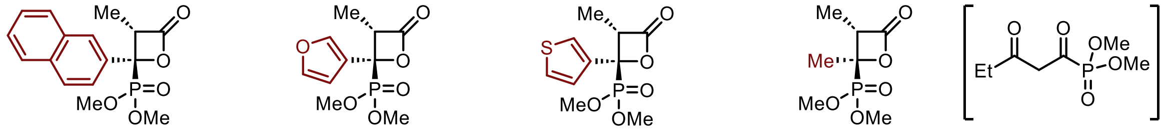

26 27 28 29 S36

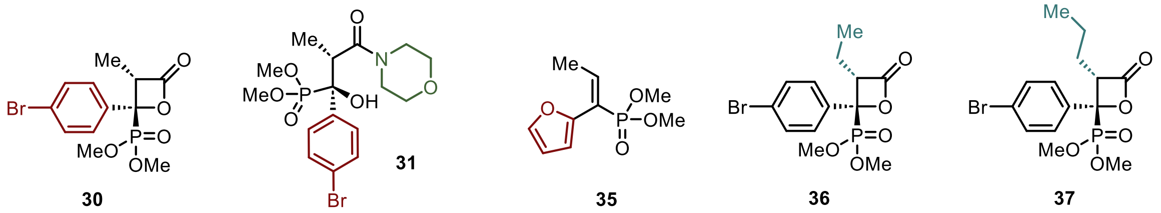

30 31 35 36 37

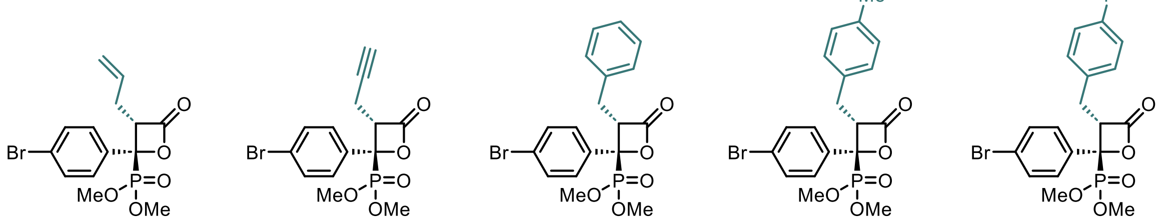

38 39 40 41 42

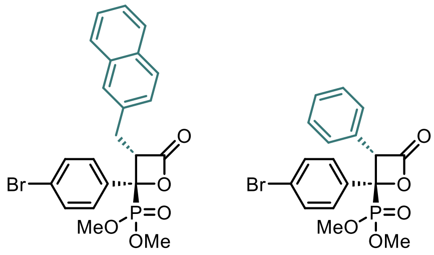

43 44

Limitations

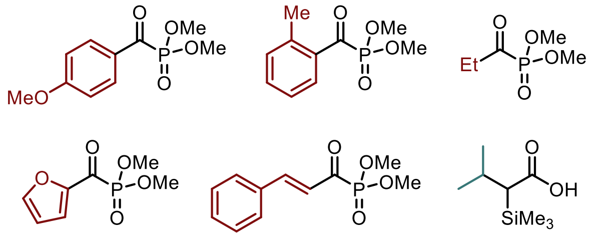

Dimethyl ((2*R*,3*R*)-3-methyl-4-oxo-2-phenyloxetan-2-yl)phosphonate (5)

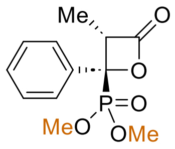

5

Following General Procedure D, 2-(trimethylsilyl)propanoic acid (58.5 mg, 0.4 mmol), *N*,*N*-diisopropylethylamine (69.7 µL, 0.4 mmol), pivaloyl chloride (49.0 µL, 0.4 mmol) and MTBE (2.0 mL) for 15 mins, followed by dimethyl benzoylphosphonate (42.3 mg, 0.2 mmol), (2*S*,3*R*)−HyperBTM (3.1 mg, 10.0 µmol) and *N*,*N*-diisopropylethylamine (34.9 µL, 0.2 mmol) for 16 h gave, after purification by Biotage® Isolera™ 4 [SNAP KP-Sil 10 g, 36 mL min^{-1}, petrol : Et_2O (90:10 to 0:100, 30 CV)], the title compound (53 mg, 98%) as a colourless oil. $[\alpha]_D^{20}$ −70.6 (*c*, 0.6, $CHCl_3$); **Chiral HPLC analysis**: Chiralcel OJ-H (90:10 hexane:IPA, flow rate 1.0 $mLmin^{-1}$, 211 nm, 30 ℃), t_R(2*S*,3*S*): 18.8 min, t_R(2*R*,3*R*): 38.6 min, >99:1 er; **IR** ν_{max} (film) 1834 (C=O), 1258 (P=O), 1026 (P−O); **^{1}H NMR** (400 MHz, $CDCl_3$) δ_H: 7.40 – 7.54 (5H, m, Ar*H*), 4.32 (1H, dq, *J* 10.6, 7.7, C(3)*H*), 3.91 (3H, d, *J* 10.6, OC*H*$_3$), 3.57 (3H, d, *J* 10.5, OC*H*$_3$), 1.06 (3H, d, *J* 7.8, C(3)C*H*$_3$); **^{31}P NMR** (162 MHz, $CDCl_3$) δ_P:18.6; **^{13}C{^{1}H} NMR** (101 MHz, $CDCl_3$) δ_C: 169.9 (d, *J* 7.8, *C*(2)) , 131.4 (d, *J* 5.6, Ph*C*(1)), 128.9 (d, *J* 2.4, Ph*C*(4)H), 128.7 (d, *J* 2.1, Ph*C*(3,5)H), 126.3 (d, *J* 3.9, Ph*C*(2,6)H), 78.8 (d, *J* 170.0, *C*(4)), 55.1 (d, *J* 7.0, O*C*H$_3$), 54.4 (d, *J* 7.6, O*C*H$_3$), 53.4 (*C*(3)), 10.6 (d, *J* 2.3, C(3)*C*H$_3$); **HRMS** (ESI$^+$) $C_{12}H_{16}O_5P$ $[M+H]^+$ found 271.0726, requires 271.0730 (−1.4 ppm).

Diethyl ((2*R*,3*R*)-3-methyl-4-oxo-2-phenyloxetan-2-yl)phosphonate (11)

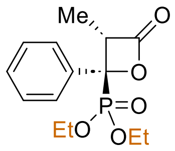

11

Following General Procedure D, 2-(trimethylsilyl)propanoic acid (58.5 mg, 0.4 mmol), *N*,*N*-diisopropylethylamine (69.7 µL, 0.4 mmol), pivaloyl chloride (49.0 µL, 0.4 mmol) and MTBE (2.0 mL) for 15 mins, followed by diethyl benzoylphosphonate (48.4 mg, 0.2 mmol), (2*S*,3*R*)−HyperBTM (3.1 mg, 10.0 µmol) and *N*,*N*-diisopropylethylamine (34.9 µL, 0.2 mmol) for 16 h gave, after purification by Biotage® Isolera™ 4 [SNAP KP-Sil 10 g, 36 mL min^{-1}, petrol : Et_2O (90:10 to 20:80, 30 CV)], the title compound (48 mg, 80%) as a colourless oil. $[\alpha]_D^{20}$ −64.1 (*c*, 0.4, $CHCl_3$)**; Chiral HPLC analysis** Chiralcel OJ-H (95:5 hexane:IPA, flow rate 1.0 $mLmin^{-1}$, 211 nm, 30 ℃), t_R(2*S*,3*S*): 9.0 min, t_R(2*R*,3*R*): 17.8 min, >99:1 er; **IR** ν_{max} (film) 1836 (C=O), 1016 (P−O); **^{1}H NMR** (400 MHz, $CDCl_3$) δ_H: 7.38 – 7.47 (3H, m, Ph*H*), 7.51 – 7.54 (2H, m, Ph*H*), 4.23 – 4.35 (3H, m, C*H*$_2$CH$_3$, C(3)*H*), 3.94 – 4.04 (1H, m, C*H*$_2$CH$_3$), 3.74

– 3.84 (1H, m, CH_2CH$_3$), 1.39 (3H, td, *J* 7.5, 0.5, CH$_2$CH_3), 1.11 (3H, td, *J* 7.1, 0.6, CH$_2$CH_3), 1.06 (3H, d, *J* 7.8, C(3)CH_3); **^{31}P NMR** (162 MHz, CDCl$_3$) δ_P: 16.3; **^{13}C{^{1}H} NMR** (101 MHz, CDCl$_3$) δ_C: 170.1 (d, *J* 7.7, *C*(2)), 131.7 (d, *J* 5.6, Ph*C*(1)), 128.7 (d, *J* 2.4, Ph*C*(4)H), 128.6 (d, *J* 2.0, Ph*C*(3,5)H), 126.3 (d, *J* 4.1, Ph*C*(2,6)H), 78.8 (d, *J* 169.0, *C*(4)), 64.7 (d, *J* 7.4, *C*H$_2$CH$_3$), 64.2 (d, *J* 7.8, *C*H$_2$CH$_3$), 53.2 (d, *J* 1.3, *C*(3)H), 16.4 (d, *J* 5.6, CH$_2$CH$_3$), 16.2 (d, *J* 5.5, CH$_2$CH$_3$), 10.7 (d, *J* 2.4,C(3)*C*H$_3$); **HRMS** (ESI$^+$) $C_{14}H_{20}O_5P$ [M+H]$^+$ found 299.1038, requires 299.1043 (−1.6 ppm).

Diisopropyl ((2*R*,3*R*)-3-methyl-4-oxo-2-phenyloxetan-2-yl)phosphonate (12)

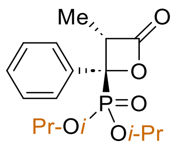

12

Following General Procedure D, 2-(trimethylsilyl)propanoic acid (58.5 mg, 0.4 mmol), *N*,*N*-diisopropylethylamine (69.7 µL, 0.4 mmol), pivaloyl chloride (49.0 µL, 0.4 mmol) and MTBE (2.0 mL) for 15 mins, followed by diisopropyl benzoylphosphonate (54.1 mg, 0.2 mmol), (2*S*,3*R*)−HyperBTM (6.2 mg, 20.0 µmol) and *N*,*N*-diisopropylethylamine (34.9 µL, 0.2 mmol) for 16 h gave, after purification by Biotage® Isolera™ 4 [SNAP KP-Sil 10 g, 36 mL min^{-1}, petrol : Et$_2$O (90:10 to 30:70, 30 CV)], the title compound (33 mg, 50%) as a colourless oil. $[\alpha]_D^{20}$ −60.4 (*c*, 0.5, CHCl$_3$); **Chiral HPLC analysis** Chiralcel OJ-H (98:2 hexane:IPA, flow rate 1.0 mLmin^{-1}, 211 nm, 30 ℃), t_R(2*S*,3*S*): 7.4 min, t_R(2*R*,3*R*): 11.3 min, >99:1 er; **IR** ν_{max} (film) 1840 (C=O), 989 (P−O); **^{1}H NMR** (400 MHz, CDCl$_3$) δ_H: 7.52 – 7.55 (2H, m, Ph*H*), 7.38 – 7.46 (3H, m, Ph*H*), 4.79 – 4.90 (1H, m, C*H*CH$_3$), 4.39 – 4.50 (1H, m, C*H*CH$_3$), 4.26 (1H, dq, *J* 10.5, 7.7, C(3)*H*), 1.40 (3H, d, *J* 1.8, CHCH_3), 1.39 (3H, d, *J* 1.8, CHCH_3), 1.26 (3H, d, *J* 6.2, CHCH_3), 1.04 (3H, d, *J* 7.7, C(3)CH_3), 0.91 (3H, d, *J* 6.3, CHCH_3); **^{31}P NMR** (162 MHz, CDCl$_3$) δ_P: 14.7; **^{13}C{^{1}H} NMR** (101 MHz, CDCl$_3$) δ_C: 170.4 (d, *J* 7.8, *C*(2)), 131.9 (d, *J* 5.9, Ph*C*(1)), 128.6 (d, *J* 2.4, Ph*C*(4)H), 128.4 (d, *J* 2.0, Ph*C*(3,5)H), 126.5 (d, *J* 4.0, Ph*C*(2,6)H), 78.8 (d, *J* 170.2, *C*(4)), 73.6 (d, *J* 7.6, O*C*H), 73.0 (d, *J* 8.0, O*C*H), 53.1 (*C*(3)H), 24.3 (d, *J* 2.6, CH*C*H$_3$), 24.0 (d, *J* 4.6, CH*C*H$_3$), 23.9 (d, *J* 4.3, CH*C*H$_3$), 23.0 (d, *J* 5.8, CH*C*H$_3$), 10.8 (d, *J* 2.3,C(3)*C*H$_3$); **HRMS** (ESI$^+$) $C_{16}H_{24}O_5P$ [M+H]$^+$ found 327.1357, requires 327.1356 (+0.4 ppm).

Dimethyl (R)-(4-oxo-2-phenyloxetan-2-yl)phosphonate (13)

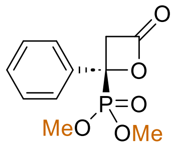

13

Following General Procedure D, 2-(trimethylsilyl)acetic acid (52.9 mg, 0.4 mmol), *N*,*N*-diisopropylethylamine (69.7 μL, 0.4 mmol), pivaloyl chloride (49.0 μL, 0.4 mmol) and MTBE (2.0 mL) for 15 mins, followed by dimethyl benzoylphosphonate (42.3 mg, 0.2 mmol), (2*S*,3*R*)−HyperBTM (3.1 mg, 10.0 μmol) and *N*,*N*-diisopropylethylamine (34.9 μL, 0.2 mmol) for 16 h gave, after purification by Biotage® Isolera™ 4 [SNAP KP-Sil 10 g, 36 mL min^{-1}, petrol : Et_2O (90:10 to 0:100, 30 CV)], the title compound (46 mg, 90%) as a colourless oil. $[\alpha]_D^{20}$ −18.0 (*c*, 0.7, $CHCl_3$); **Chiral HPLC analysis** Chiralcel OJ-H (90:10 hexane:IPA, flow rate 1.0 $mLmin^{-1}$, 211 nm, 30 °C), t_R(2*R*,3*R*): 33.1 min, t_R(2*S*,3*S*): 38.5 min, 87:13 er; **IR** ν_{max} (film) 1838 (C=O), 1258 (P=O), 1026 (P−O); **^{1}H NMR** (400 MHz, $CDCl_3$) δ_H: 7.40 – 7.51 (5H, m, Ph*H*), 4.21 (1H, dd, *J* 16.5, 10.2, C(3)H_A*H*$_B$), 3.86 (3H, d, *J* 10.6, OC*H*$_3$), 3.69 (1H, dd, *J* 16.5, 10.5, C(3)*H*$_A$$H_B$), 3.67 (3H, d, *J* 10.6, OC*H*$_3$); **^{31}P NMR** (162 MHz, $CDCl_3$) δ_P: 18.2; **^{13}C{^{1}H} NMR** (101 MHz, $CDCl_3$) δ_C: 165.4 (d, *J* 6.7, *C*(2)), 134.6 (d, *J* 4.6, Ph*C*(1)), 129.0 (d, *J* 2.6, Ph*C*(4)H), 128.7 (d, *J* 2.1, Ph*C*(3,5)H), 126.2 (d, *J* 3.9, Ph*C*(2,6)H), 74.5 (d, *J* 172.5, *C*(4)), 55.1 (d, *J* 7.0, O*C*H_3), 54.5 (d, *J* 7.4, O*C*H_3), 48.9 (*C*(3)H_2); **HRMS** (ESI$^+$) $C_{11}H_{13}O_5NaP$ $[M+Na]^+$ found 279.0391, requires 279.0393 (−0.6 ppm).

Diethyl (*R*)-(4-oxo-2-phenyloxetan-2-yl)phosphonate (14)

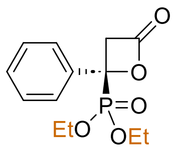

14

Following General Procedure D, 2-(trimethylsilyl)acetic acid (52.9 mg, 0.4 mmol), *N*,*N*-diisopropylethylamine (69.7 μL, 0.4 mmol), pivaloyl chloride (49.0 μL, 0.4 mmol) and MTBE (2.0 mL) for 15 mins, followed by diethyl benzoylphosphonate (48.4 mg, 0.2 mmol), (2*S*,3*R*)−HyperBTM (3.1 mg, 10.0 μmol) and *N*,*N*-diisopropylethylamine (34.9 μL, 0.2 mmol) for 16 h gave, after purification by Biotage® Isolera™ 4 [SNAP KP-Sil 10 g, 36 mL min^{-1}, petrol : Et_2O (90:10 to 20:80, 30 CV)], the title compound (49 mg, 86%) as a colourless oil. $[\alpha]_D^{20}$ −20.0 (*c*, 0.5, $CHCl_3$); **Chiral HPLC analysis** Chiralpak AS-H (95:5 hexane:IPA, flow rate 1.0 $mLmin^{-1}$, 211 nm, 30 °C), t_R(2*R*,3*R*): 8.7 min, t_R(2*S*,3*S*): 10.8 min, 88:12 er; **IR** ν_{max} (film) 1836 (C=O), 1015 (P−O); **^{1}H NMR** (400 MHz, $CDCl_3$) δ_H: 7.38 – 7.51 (5H, m, Ph*H*), 4.16 – 4.26 (3H, m, CH$_A$*H*$_B$CH, C*H*$_2$CH$_3$), 3.91 – 4.11 (2H, m, C(3)H_A*H*$_B$, C*H*$_A$H$_B$CH$_3$), 3.67 (1H, dd, *J* 16.3, 10.5, C(3)*H*$_A$$H_B$), 1.36 (3H, td, *J* 7.1, 0.4, CH$_2$C*H*$_3$), 1.22 (3H, td, *J* 7.1, 0.5, CH$_2$C*H*$_3$); **^{31}P NMR** (162 MHz, $CDCl_3$) δ_P: 15.9; **^{13}C{^{1}H} NMR** (101 MHz, $CDCl_3$) δ_C: 165.7 (d, *J* 6.7, *C*(2)), 134.8 (d, *J* 4.7, Ph*C*(1)), 128.9 (d, *J* 2.5, Ph*C*(4)H), 128.5 (d, *J* 2.2, Ph*C*(3,5)H), 126.3 (d, *J* 3.7, Ph*C*(2,6)H), 74.4 (d, *J* 172.0, *C*(4)), 64.7 (d, *J* 7.0, *C*H$_2$CH$_3$), 64.3 (d, *J* 7.5, *C*H$_2$CH$_3$), 48.8 (*C*(3)H_2), 16.4 (d, *J* 5.6, CH$_2$*C*H$_3$), 16.3 (d, *J* 5.5, CH$_2$*C*H$_3$); **HRMS** (ESI$^+$) $C_{13}H_{18}O_5P$ $[M+H]^+$ found 285.0882, requires 285.0886 (−1.4 ppm).

Diisopropyl (*R*)-(4-oxo-2-phenyloxetan-2-yl)phosphonate (15)

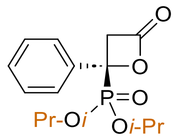

15

Following General Procedure D, 2-(trimethylsilyl)acetic acid (52.9 mg, 0.4 mmol), *N*,*N*-diisopropylethylamine (69.7 μL, 0.4 mmol), pivaloyl chloride (49.0 μL, 0.4 mmol) and MTBE (2.0 mL) for 15 mins, followed by diisopropyl benzoylphosphonate (54.1 mg, 0.2 mmol), (2*S*,3*R*)−HyperBTM (6.2 mg, 20.0 μmol) and *N*,*N*-diisopropylethylamine (34.9 μL, 0.2 mmol) for 16 h gave, after purification by Biotage® Isolera™ 4 [SNAP KP-Sil 10 g, 36 mL min^{-1}, petrol : Et_2O (90:10 to 30:70, 30 CV)], the title compound (55 mg, 88%) as a colourless oil. $[\alpha]_D^{20}$ −20.6 (*c*, 0.5, $CHCl_3$); **Chiral HPLC analysis** Chiralcel OD-H (95:5 hexane:IPA, flow rate 1.0 mLmin^{-1}, 211 nm, 30 ℃), t_R(2*S*,3*S*): 7.0 min, t_R(2*R*,3*R*): 7.7 min, 89:11 er; **IR** ν_{max} (film) 1838 (C=O), 978 (P−O); **^{1}H NMR** (400 MHz, $CDCl_3$) δ_H: 7.48 − 7.52 (2H, m, PhC(3,5)*H*), 7.37 – 7.45 (3H, m, PhC(2,4,6)*H*), 4.72 – 4.83 (1H, m, C*H*CH$_3$), 4.53 – 4.64 (1H, m, C*H*CH$_3$), 4.15 (1H, dd, *J* 16.5, 10.0, C(3)H$_A$*H*$_B$), 3.63 (1H, dd, *J* 16.2, 10.5, C(3)*H*$_A$H$_B$), 1.37 (3H, d, *J* 6.2, CHC*H*$_3$), 1.32 (3H, d, *J* 6.2, CHC*H*$_3$), 1.29 (3H, d, *J* 6.2, CHC*H*$_3$), 1.12 (3H, d, *J* 6.1, CHC*H*$_3$); **^{31}P NMR** (162 MHz, $CDCl_3$) δ_P: 14.1; **^{13}C{^{1}H} NMR** (101 MHz, $CDCl_3$) δ_C: 166.1 (d, *J* 6.7, *C*(2)), 135.0 (d, *J* 4.8, Ph*C*(1)), 128.7 (d, *J* 2.6, Ph*C*(4)H), 128.3 (d, *J* 2.1, Ph*C*(3,5)H), 126.4 (d, *J* 3.9, Ph*C*(2,6)H), 74.4 (d, *J* 174.2, *C*(4)), 73.7 (d, *J* 7.6, O*C*H), 73.1 (d, *J* 7.2, O*C*H), 53.1 (*C*(3)H), 48.8 (*C*(3)), 24.2 (d, *J* 2.9, CH*C*H$_3$), 24.0 (d, *J* 3.6, CH*C*H$_3$), 23.8 (d, *J* 5.2, CH*C*H$_3$), 23.4 (d, *J* 5.6, CH*C*H$_3$); **HRMS** (ESI$^+$) $C_{15}H_{21}NaO_5P$ [M+Na]$^+$ found 335.1017, requires 335.1019 (−0.6 ppm).

Dimethyl ((2R,3R)-3-methyl-4-oxo-2-(4-(trifluoromethyl)phenyl)oxetan-2-yl)phosphonate (16)

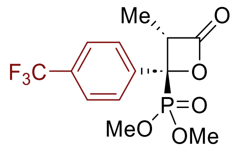

16

Following General Procedure D, 2-(trimethylsilyl)propanoic acid (47 mg, 0.32 mmol), *N*,*N*-diisopropylethylamine (69.7 μL, 0.4 mmol), pivaloyl chloride (49.0 μL, 0.4 mmol) and MTBE (2.0 mL) for 15 mins, followed by dimethyl (4-trifluoromethylbenzoyl)phosphonate (56.4 mg, 0.2 mmol), (2*S*,3*R*)−HyperBTM (3.1 mg, 10.0 μmol) and *N*,*N*-diisopropylethylamine (34.9 μL, 0.2 mmol) rt for 16 h gave, after purification by Biotage® Isolera™ 4 [SNAP KP-Sil 10 g, 36 mL min^{-1}, petrol : Et_2O (90:10 to 0:100, 30 CV)], the title compound (59 mg, 88%) as a pale yellow oil. $[\alpha]_D^{20}$ −44.1 (*c*, 0.2, $CHCl_3$); **Chiral HPLC analysis** Chiralcel OJ-H (90:10 hexane:IPA, flow rate 1.0 mLmin^{-1}, 211 nm, 30 ℃), t_R(2*S*,3*S*): 8.8 min, t_R(2*R*,3*R*): 10.0 min, >99:1 er; **IR** ν_{max} (film) 1844 (C=O), 1325 (P=O), 1034 (P−O); **^{1}H NMR** (400

MHz, $CDCl_3$) δ_H: 7.74 – 7.76 (2H, m, ArC(3,5)*H*), 7.66 – 7.68 (2H, m, ArC(2,6)*H*), 4.37 (1H, dq, *J* 10.6, 7.7, C(3)*H*), 3.94 (3H, d, *J* 10.6, OC*H*$_3$), 3.64 (3H, d, *J* 10.5, OC*H*$_3$), 1.06 (3H, d, *J* 7.8, C(3)C*H*$_3$); **^{19}F NMR** (376 MHz, $CDCl_3$) δ_F: −62.8 (d, *J* 2.1); **^{31}P NMR** (162 MHz, $CDCl_3$) δ_P: 17.9; **^{13}C{^{1}H} NMR** (101 MHz, $CDCl_3$) δ_C: 169.1 (d, *J* 7.9, *C*(2)), 135.8 (d, *J* 5.6, Ar*C*(1)), 131.3 (dd, *J* 33.1, 2.7, Ar*C*(4)), 126.8 (dd, *J* 4.1, 0.3, Ar*C*(2,6)H), 125.7 (m, Ar*C*(3,5)H), 123.7 (q, *J* 272.6, *C*F$_3$), 78.6 (d, *J* 170.0, *C*(4)), 55.2 (d, *J* 7.0, O*C*H$_3$), 54.6 (d, *J* 7.6, O*C*H$_3$), 53.8 (*C*(3)H), 10.7 (d, *J* 2.4,C(3)*C*H$_3$); **HRMS** (ESI$^+$) $C_{13}H_{15}O_5F_3P$ $[M+H]^+$ found 339.0599, requires 339.0604 (−1.3 ppm).

Dimethyl ((2*R*,3*R*)-2-(4-chlorophenyl)-3-methyl-4-oxooxetan-2-yl)phosphonate (17)

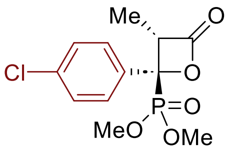

17

Following General Procedure D, 2-(trimethylsilyl)propanoic acid (58.5 mg, 0.4 mmol), *N*,*N*-diisopropylethylamine (69.7 µL, 0.4 mmol), pivaloyl chloride (49.0 µL, 0.4 mmol) and MTBE (2.0 mL) for 15 mins, followed by dimethyl (4-chlorobenzoyl)phosphonate (49.7 mg, 0.2 mmol), (2*S*,3*R*)−HyperBTM (3.1 mg, 10.0 µmol) and *N*,*N*-diisopropylethylamine (34.9 µL, 0.2 mmol) for 16 h gave, after purification by Biotage® Isolera™ 4 [SNAP KP-Sil 10 g, 36 mL min^{-1}, petrol : Et_2O (90:10 to 10:90, 30 CV)], the title compound (44 mg, 72%) as a pale yellow oil. $[\alpha]_D^{20}$ −48.2 (*c*, 0.3, $CHCl_3$); **Chiral HPLC analysis** Chiralcel OJ-H (90:10 hexane:IPA, flow rate 1.0 mLmin^{-1}, 211 nm, 30 ℃), t_R(2*S*,3*S*): 8.5 min, t_R(2*R*,3*R*): 11.0 min, >99:1 er; **IR** ν_{max} (film) 1838 (C=O), 1026 (P−O); **^{1}H NMR** (400 MHz, $CDCl_3$) δ_H: 7.43 – 7.49 (4H, m, Ar*H*), 4.31 (1H, dq, *J* 10.6, 7.8, C(3)*H*), 3.91 (3H, d, *J* 10.6, OC*H*$_3$), 3.61 (3H, d, *J* 10.5, OC*H*$_3$), 1.05 (3H, d, *J* 7.7, C(3)C*H*$_3$); **^{31}P NMR** (162 MHz, $CDCl_3$) δ_P: 18.2; **^{13}C{^{1}H} NMR** (101 MHz, $CDCl_3$) δ_C: 169.4 (d, *J* 7.8, *C*(2)), 135.2 (d, *J* 3.0, Ar*C*(1)), 130.1 (d, *J* 5.8, Ar*C*(4)), 129.0 (d, *J* 2.0, Ar*C*(3,5)H), 127.7 (d, *J* 4.1, Ar*C*(2,6)H), 78.5 (d, *J* 170.0, *C*(4)), 55.2 (d, *J* 7.0, O*C*H$_3$), 54.5 (d, *J* 7.6, O*C*H$_3$), 53.5 (d, *J* 1.1, *C*(3)H), 10.6 (d, *J* 2.4,C(3)*C*H$_3$); **HRMS** (ESI$^+$) $C_{12}H_{14}ClNaO_5P$ $[M+Na]^+$ found 327.0156, requires 327.0160 (−1.1 ppm).

Methyl 4-((2*R*,3*R*)-2-(dimethoxyphosphoryl)-3-methyl-4-oxooxetan-2-yl)benzoate (18)

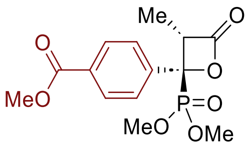

18

Following General Procedure D, 2-(trimethylsilyl)propanoic acid (58.5 mg, 0.4 mmol), *N*,*N*-diisopropylethylamine (69.7 µL, 0.4 mmol), pivaloyl chloride (49.0 µL, 0.4 mmol) and MTBE (2.0 mL) for

15 mins, followed by methyl 4-((dimethoxyphosphoryl)carbonyl)benzoate (54.4 mg, 0.2 mmol), (2*S*,3*R*)−HyperBTM (3.1 mg, 10.0 µmol) and *N*,*N*-diisopropylethylamine (34.9 µL, 0.2 mmol) for 16 h gave, after purification by Biotage® Isolera™ 4 [SNAP KP-Sil 10 g, 36 mL min^{-1}, petrol : Et_2O (90:10 to 0:100, 30 CV)], the title compound (50 mg, 76%) as a white solid. **mp** 94 – 98 °C; $[\alpha]_D^{20}$ −45.1 (*c*, 0.4, $CHCl_3$); **Chiral HPLC analysis** Chiralcel OD-H (90:10 hexane:IPA, flow rate 1.0 mLmin^{-1}, 211 nm, 30 ℃), t_R(2*S*,3*S*): 13.6 min, t_R(2*R*,3*R*): 16.0 min, >99:1 er; **IR** ν_{max} (film) 1838 (C=O), 1263 (P=O), 1018 (P−O); **^{1}H NMR** (400 MHz, $CDCl_3$) δ_H: 8.13 – 8.15 (2H, m, ArC(3,5)*H*), 7.60 – 7.64 (2H, m, ArC(2,6)*H*), 4.35 (1H, dq, *J* 10.7, 7.7, C(3)*H*), 3.96 (3H, COOC*H*$_3$), 3.92 (3H, d, *J* 10.6, OC*H*$_3$), 3.60 (3H, d, *J* 10.5, OC*H*$_3$), 1.05 (3H, d, *J* 7.8, C(3)C*H*$_3$); **^{31}P NMR** (162 MHz, $CDCl_3$) δ_H: 17.9; **^{13}C{^{1}H} NMR** (101 MHz, $CDCl_3$) δ_H: 169.2 (d, *J* 7.6, *C*(2)), 166.7 (*C*OOCH$_3$), 136.6 (d, *J* 5.7, Ar*C*(1)), 130.7 (d, *J* 2.5, Ar*C*(4)), 129.9 (d, *J* 1.8, Ar*C*(3,5)H), 126.4 (d, *J* 4.0, Ar*C*(2,6)H), 78.8 (d, *J* 169.7, *C*(4)), 55.2 (d, *J* 7.0, O*C*H$_3$), 54.6 (d, *J* 7.5, O*C*H$_3$), 53.7 (*C*(3)H), 52.4 (COO*C*H$_3$), 10.7 (d, *J* 2.5,C(3)*C*H$_3$); **HRMS** (ESI$^+$) $C_{14}H_{17}O_7NaP$ [M+Na]$^+$ found 351.0599, requires 351.0604 (−1.5 ppm).

Dimethyl ((2*R*,3*R*)-2-(4-fluorophenyl)-3-methyl-4-oxooxetan-2-yl)phosphonate (19)

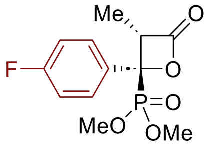

19

Following General Procedure D, 2-(trimethylsilyl)propanoic acid (47 mg, 0.32 mmol), *N*,*N*-diisopropylethylamine (69.7 µL, 0.4 mmol), pivaloyl chloride (49.0 µL, 0.4 mmol) and MTBE (2.0 mL) for 15 mins, followed by dimethyl (4-fluorobenzoyl)phosphonate (46.4 mg, 0.2 mmol), (2*S*,3*R*)−HyperBTM (3.1 mg, 10.0 µmol) and *N*,*N*-diisopropylethylamine (34.9 µL, 0.2 mmol) rt for 16 h gave, after purification by Biotage® Isolera™ 4 [SNAP KP-Sil 10 g, 36 mL min^{-1}, petrol : Et_2O (90:10 to 0:100, 30 CV)], the title compound (52 mg, 90%) as a pale yellow oil. $[\alpha]_D^{20}$ −66.2 (*c*, 0.7, $CHCl_3$); **Chiral HPLC analysis** Chiralcel OJ-H (90:10 hexane:IPA, flow rate 1.0 mLmin^{-1}, 211 nm, 30 ℃), t_R(2*S*,3*S*): 15.0 min, t_R(2*R*,3*R*): 23.5 min, >99:1 er; **IR** ν_{max} (film) 1838 (C=O), 1256 (P=O), 1030 (P−O); **^{1}H NMR** (400 MHz, $CDCl_3$) δ_H: 7.49 – 7.55 (2H, m, ArC(2,6)*H*), 7.14 – 7.20 (2H, m, ArC(3,5)*H*), 4.30 (1H, dq, *J* 10.5, 7.7, C(3)*H*), 3.91 (3H, d, *J* 10.6, OC*H*$_3$), 3.60 (3H, d, *J* 10.6, OC*H*$_3$), 1.05 (3H, d, *J* 7.7, C(3)C*H*$_3$); **^{19}F NMR** (377 MHz, $CDCl_3$) δ_F: −112.3 (d, *J* 4.1); **^{31}P NMR** (162 MHz, $CDCl_3$) δ_P: 18.4 (d, *J* 3.7); **^{13}C{^{1}H} NMR** (101 MHz, $CDCl_3$) δ_C: 169.5 (d, *J* 7.9, *C*(2)), 162.9 (dd, *J* 249.1, 2.8, Ar*C*(4)), 128.3 (dd, *J* 8.2, 4.1, Ar*C*(2,6)H), 127.3 (dd, *J* 5.7, 3.2, Ar*C*(1)H), 115.9 (dd, *J* 21.9, 1.9, Ar*C*(3,5)H), 78.5 (d, *J* 171.0, *C*(4)), 55.1 (d, *J* 6.8, O*C*H$_3$), 54.5 (d, *J* 7.5, O*C*H$_3$), 53.4 (*C*(3)H), 10.6 (d, *J* 2.3,C(3)*C*H$_3$); **HRMS** (ESI$^+$) $C_{12}H_{15}O_5FP$ [M+H]$^+$ found 289.0637, requires 289.0636 (−0.5 ppm).

Dimethyl ((2*R*,3*R*)-3-methyl-4-oxo-2-(*p*-tolyl)oxetan-2-yl)phosphonate (20)

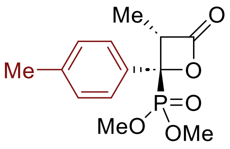

20

Following General Procedure D, 2-(trimethylsilyl)propanoic acid (58.5 mg, 0.4 mmol), *N*,*N*-diisopropylethylamine (69.7 µL, 0.4 mmol), pivaloyl chloride (49.0 µL, 0.4 mmol) and MTBE (2.0 mL) for 15 mins, followed by dimethyl (4-methylbenzoyl)phosphonate (45.6 mg, 0.2 mmol), (2*S*,3*R*)−HyperBTM (3.1 mg, 10.0 µmol) and *N*,*N*-diisopropylethylamine (34.9 µL, 0.2 mmol) for 16 h gave, after purification by Biotage® Isolera™ 4 [SNAP KP-Sil 10 g, 36 mL min^{-1}, petrol : Et_2O (90:10 to 0:100, 30 CV)], the title compound (45 mg, 78%) as a colourless oil. $[\alpha]_D^{20}$ −52.6 (*c*, 0.4, $CHCl_3$); **Chiral HPLC analysis** Chiralcel OD-H (90:10 hexane:IPA, flow rate 1.0 mLmin^{-1}, 211 nm, 30 ℃), t_R(2*S*,3*S*): 7.5 min, t_R(2*R*,3*R*): 9.5 min, >99:1 er; **IR** $ν_{max}$ (film) 1834 (C=O), 1020 (P−O); **^{1}H NMR** (400 MHz, $CDCl_3$) δ_H: 7.38 – 7.42 (2H, m, ArC(2,6)*H*), 7.26 – 7.28 (2H, m, ArC(3,5)*H*), 4.28 (1H, dq, *J* 10.6, 7.7, C(3)*H*), 3.90 (3H, d, *J* 10.5, OCH_3), 3.57 (3H, d, *J* 10.5, OCH_3), 2.39 (3H, d, *J* 1.4, ArCH_3), 1.05 (3H, d, *J* 7.7, C(3)CH_3); **^{31}P NMR** (162 MHz, $CDCl_3$) δ_P: 18.7; **^{13}C{^{1}H} NMR** (101 MHz, $CDCl_3$) δ_C: 170.0 (d, *J* 7.8, *C*(2)), 138.8 (d, *J* 2.6, Ar*C*(4)), 129.4 (d, *J* 1.9, Ar*C*(3,5)H), 128.3 (d, *J* 5.6, Ar*C*(1)H), 126.1 (d, *J* 4.0, Ar*C*(2,6)H), 78.9 (d, *J* 170.0, *C*(4)), 55.1 (d, *J* 6.9, O*C*H_3), 54.4 (d, *J* 7.4, O*C*H_3), 53.3 (d, *J* 1.6, *C*(3)H), 21.2 (Ar*C*H_3), 10.6 (d, *J* 2.3,C(3)*C*H_3); **HRMS** (ESI$^+$) $C_{13}H_{18}O_5P$ [M+H]$^+$ found 285.0884, requires 285.0886 (−0.7 ppm).

Dimethyl ((2*R*,3*R*)-2-([1,1'-biphenyl]-4-yl)-3-methyl-4-oxooxetan-2-yl)phosphonate (21)

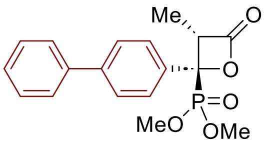

21

Following General Procedure D, 2-(trimethylsilyl)propanoic acid (58.5 mg, 0.4 mmol), *N*,*N*-diisopropylethylamine (69.7 µL, 0.4 mmol), pivaloyl chloride (49.0 µL, 0.4 mmol) and MTBE (2.0 mL) for 15 mins, followed by dimethyl ([1,1'-biphenyl]-4-carbonyl)phosphonate (58.1 mg, 0.2 mmol), (2*S*,3*R*)−HyperBTM (3.1 mg, 10.0 µmol) and *N*,*N*-diisopropylethylamine (34.9 µL, 0.2 mmol) for 16 h gave, after purification by Biotage® Isolera™ 4 [SNAP KP-Sil 10 g, 36 mL min^{-1}, petrol : Et_2O (90:10 to 0:100, 30 CV)], the title compound (46 mg, 67%) as a white solid. **mp** 104 – 107 °C; $[\alpha]_D^{20}$ −24.6 (*c*, 0.4, $CHCl_3$); **Chiral HPLC analysis** Chiralcel OD-H (90:10 hexane:IPA, flow rate 1.0 mLmin^{-1}, 211 nm, 30 ℃), t_R(2*S*,3*S*): 11.2 min, t_R(2*R*,3*R*): 14.3 min, >99:1 er; **IR** $ν_{max}$ (film) 1834 (C=O), 1022 (P−O); **^{1}H NMR** (500 MHz, $CDCl_3$) δ_H: 7.70 – 7.72 (2H, m, PhC(2,6)*H*), 7.63 – 7.65 (2H, m, PhC(3,5)*H*), 7.59 – 7.61 (2H,

m, ArC(2,6)*H*), 7.47 – 7.50 (2H, m, ArC(3,5)*H*), 7.38 – 7.42 (1H, m, PhC(4)*H*), 4.35 (1H, dq, *J* 10.6, 7.7, C(3)*H*), 3.94 (3H, d, *J* 10.5, OC*H*$_3$), 3.63 (3H, d, *J* 10.5, OC*H*$_3$), 1.12 (3H, d, *J* 7.7, C(3)C*H*$_3$); **^{31}P NMR** (202 MHz, CDCl$_3$) δ_P: 18.6; **^{13}C{^{1}H} NMR** (126 MHz, CDCl$_3$) δ_C: 169.8 (d, *J* 7.8, *C*(2)), 141.7 (d, *J* 2.6, Ph*C*(1)), 140.0 (Ar*C*(4)), 130.3 (d, *J* 5.6, Ar*C*(1)), 128.9 (Ph*C*(3,5)H), 127.8 (Ph*C*(4)H), 127.3 (d, *J* 2.1, Ar*C*(2,6)H), 127.1 (Ar*C*(3,5)H), 126.7 (d, *J* 4.1, Ph*C*(2,6)H), 78.9 (d, *J* 170.2, *C*(4)), 55.2 (d, *J* 7.0, O*C*H$_3$), 54.5 (d, *J* 7.6, O*C*H$_3$), 53.5 (d, *J* 1.4, *C*(3)H), 10.7 (d, *J* 2.6,C(3)*C*H$_3$); **HRMS** (ESI$^+$) $C_{18}H_{19}NaO_5P$ [M+Na]$^+$ found 369.0858, requires 369.0862 (−1.2 ppm).

Dimethyl ((2*R*,3*R*)-2-(3-bromophenyl)-3-methyl-4-oxooxetan-2-yl)phosphonate (22)

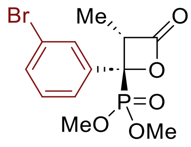

22

Following General Procedure D, 2-(trimethylsilyl)propanoic acid (58.5 mg, 0.4 mmol), *N*,*N*-diisopropylethylamine (69.7 μL, 0.4 mmol), pivaloyl chloride (49.0 μL, 0.4 mmol) and MTBE (2.0 mL) for 15 mins, followed by dimethyl (3-bromobenzoyl)phosphonate (58.6 mg, 0.2 mmol), (2*S*,3*R*)−HyperBTM (3.1 mg, 10.0 μmol) and *N*,*N*-diisopropylethylamine (34.9 μL, 0.2 mmol) for 16 h gave, after purification by Biotage® Isolera™ 4 [SNAP KP-Sil 10 g, 36 mL min^{-1}, petrol : Et$_2$O (90:10 to 0:100, 30 CV)], the title compound (57 mg, 82%) as a pale yellow oil. $[\alpha]_D^{20}$ −40.6 (*c*, 0.5, CHCl$_3$); **Chiral HPLC analysis** Chiralcel OD-H (90:10 hexane:IPA, flow rate 1.0 mLmin^{-1}, 211 nm, 30 ℃), t_R(2*S*,3*S*): 9.0 min, t_R(2*R*,3*R*): 11.0 min, >99:1 er; **IR** ν_{max} (film) 1840 (C=O), 1261 (P=O), 1030 (P−O); **^{1}H NMR** (400 MHz, CDCl$_3$) δ_H: 7.68 – 7.69 (2H, m, ArC(4)*H*), 7.55 – 7.58 (2H, m, ArC(2)*H*), 7.46 – 7.49 (1H, m, ArC(5)*H*), 7.33 – 7.37 (1H, m, ArC(6)*H*), 4.32 (1H, dq, *J* 10.6, 7.7, C(3)*H*), 3.92 (3H, d, *J* 10.5, OC*H*$_3$), 3.65 (3H, d, *J* 10.4, OC*H*$_3$), 1.07 (3H, d, *J* 7.7, C(3)C*H*$_3$); **^{31}P NMR** (162 MHz, CDCl$_3$) δ_H: 18.1; **^{13}C{^{1}H} NMR** (101 MHz, CDCl$_3$) δ_H: 169.2 (d, *J* 7.6, *C*(2)), 133.9 (d, *J* 6.0, Ar*C*(1)), 132.1 (d, *J* 2.1, Ar*C*(2)H), 130.2 (d, *J* 2.0, Ar*C*(5)H), 129.2 (d, *J* 4.4, Ar*C*(4)H), 125.0 (d, *J* 3.8, Ar*C*(6)H), 123.1 (d, *J* 2.1, Ar*C*(3)), 78.3 (d, *J* 167.0, *C*(4)), 55.2 (d, *J* 7.0, O*C*H$_3$), 54.6 (d, *J* 7.8, O*C*H$_3$), 53.7 (*C*(3)H), 10.7 (d, *J* 2.4,C(3)*C*H$_3$); **HRMS** (ESI$^+$) $C_{12}H_{15}O_5{}^{79}BrP$ [M+H]$^+$ found 348.9832, requires 348.9835 (−0.9 ppm).

Dimethyl ((2*R*,3*R*)-3-methyl-4-oxo-2-(*m*-tolyl)oxetan-2-yl)phosphonate (23)

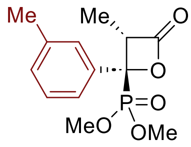

23

Following General Procedure D, 2-(trimethylsilyl)propanoic acid (58.5 mg, 0.4 mmol), *N*,*N*-diisopropylethylamine (69.7 μL, 0.4 mmol), pivaloyl chloride (49.0 μL, 0.4 mmol) and MTBE (2.0 mL) for 15 mins, followed by dimethyl (3-methylbenzoyl)phosphonate (45.6 mg, 0.2 mmol), (2*S*,3*R*)−HyperBTM (3.1 mg, 10.0 μmol) and *N*,*N*-diisopropylethylamine (34.9 μL, 0.2 mmol) for 16 h gave, after purification by Biotage® Isolera™ 4 [SNAP KP-Sil 10 g, 36 mL min^{-1}, petrol : Et_2O (90:10 to 0:100, 30 CV)], the title compound (35 mg, 62%) as a colourless oil. $[\alpha]_D^{20}$ −59.1 (*c*, 0.3, $CHCl_3$); **Chiral HPLC analysis** Chiralcel OD-H (90:10 hexane:IPA, flow rate 1.0 mLmin^{-1}, 211 nm, 30 ℃), t_R(2*S*,3*S*): 7.3 min, t_R(2*R*,3*R*): 8.7 min, >99:1 er; **IR** v_{max} (film) 1834 (C=O), 1024 (P−O); **^{1}H NMR** (400 MHz, $CDCl_3$) δ_H: 7.29 – 7.37 (3H, m, Ar*H*), 7.21 – 7.23 (1H, m, Ar*H*), 4.29 (1H, dq, *J* 10.6, 7.7, C(3)*H*), 3.91 (3H, d, *J* 10.5, OC*H*$_3$), 3.58 (3H, d, *J* 10.5, OC*H*$_3$), 2.41 (3H, s, ArC*H*$_3$), 1.06 (3H, d, *J* 7.7, C(3)C*H*$_3$); **^{31}P NMR** (162 MHz, $CDCl_3$) δ_P: 18.7; **^{13}C{^{1}H} NMR** (101 MHz, $CDCl_3$) δ_C: 170.0 (d, *J* 7.7, *C*(2)), 138.6 (d, *J* 2.0, Ar*C*(1)), 131.3 (d, *J* 5.6, Ar*C*(3)), 129.7 (d, *J* 2.4, Ar*C*(2)H), 128.5 (d, *J* 2.4, Ar*C*(5)H), 126.6 (d, *J* 4.3, Ar*C*(4)H), 123.4 (d, *J* 4.0, Ar*C*(6)H), 79.0 (d, *J* 169.3, *C*(4)), 55.1 (d, *J* 7.0, O*C*H$_3$), 54.4 (d, *J* 7.6, O*C*H$_3$), 53.3 (*C*(3)H), 21.6 (Ar*C*H$_3$), 10.6 (d, *J* 2.3,C(3)*C*H$_3$); **HRMS** (ESI$^+$) $C_{12}H_{15}O_5BrP$ [M+Na]$^+$ found 307.0699, requires 307.0706 (−2.2 ppm).

Dimethyl ((2*R*,3*R*)-2-(3-methoxyphenyl)-3-methyl-4-oxooxetan-2-yl)phosphonate (24)

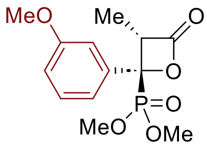

24

Following General Procedure D, 2-(trimethylsilyl)propanoic acid (58.5 mg, 0.4 mmol), *N*,*N*-diisopropylethylamine (69.7 μL, 0.4 mmol), pivaloyl chloride (49.0 μL, 0.4 mmol) and MTBE (2.0 mL) for 15 mins, followed by dimethyl (3-methoxybenzoyl)phosphonate (48.8 mg, 0.2 mmol), (2*S*,3*R*)−HyperBTM (3.1 mg, 10.0 μmol) and *N*,*N*-diisopropylethylamine (34.9 μL, 0.2 mmol) for 16 h gave, after purification by Biotage® Isolera™ 4 [SNAP KP-Sil 10 g, 36 mL min^{-1}, petrol : Et_2O (90:10 to 0:100, 30 CV)], the title compound (54.0 mg, 90%) as a colourless oil. $[\alpha]_D^{20}$ −55.1 (*c*, 0.3, $CHCl_3$); **Chiral HPLC analysis** Chiralcel OJ-H (90:10 hexane:IPA, flow rate 1.0 mLmin^{-1}, 211nm, 30 ℃), t_R(2*S*,3*S*): 20.9 min, t_R(2*R*,3*R*): 27.1 min, >99:1 er; **IR** v_{max} (film) 1838 (C=O), 1032 (P−O); **^{1}H NMR** (400 MHz, $CDCl_3$) δ_H: 7.38 (1H, t, *J* 8.0, ArC(5)*H*), 7.06 – 7.11 (2H, m, ArC(4,6)*H*), 6.93 – 6.96 (1H, m, ArC(2)*H*), 4.29 (1H, dq, *J* 10.6, 7.7, C(3)*H*), 3.91 (3H, d, *J* 10.5, OC*H*$_3$), 3.85 (3H, s, ArOC*H*$_3$), 3.60 (3H, d, *J* 10.5, OC*H*$_3$), 1.07 (3H, d, *J* 7.8, C(3)C*H*$_3$); **^{31}P NMR** (162 MHz, $CDCl_3$) δ_P: 18.5; **^{13}C{^{1}H} NMR** (101 MHz, $CDCl_3$) δ_P: 169.8 (d, *J* 7.8, *C*(2)), 159.8 (d, *J* 1.7, Ar*C*(3)), 133.0 (d, *J* 5.9, Ar*C*(1)), 129.8 (d, *J* 2.0, Ar*C*(5)H), 118.5 (d, *J* 3.9, Ar*C*(6)H), 114.5 (d, *J* 2.3, Ar*C*(2)H), 111.7 (d, *J* 4.3, Ar*C*(4)H), 78.8 (d, *J* 170.4, *C*(4)), 55.4 (ArO*C*H$_3$), 55.2 (d, *J* 6.8, O*C*H$_3$), 54.4 (d, *J* 7.6, O*C*H$_3$), 53.4 (*C*(3)H), 10.6 (d, *J* 2.5, C(3)*C*H$_3$); **HRMS** (ESI$^+$) $C_{13}H_{17}O_6NaP$ [M+Na]$^+$ found 323.0647, requires 323.0655 (−2.5 ppm).

Dimethyl ((2*R*,3*R*)-2-(3,5-dimethoxyphenyl)-3-methyl-4-oxooxetan-2-yl)phosphonate (25)

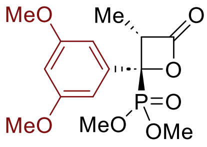

25

Following General Procedure D, 2-(trimethylsilyl)propanoic acid (58.5 mg, 0.4 mmol), *N*,*N*-diisopropylethylamine (69.7 µL, 0.4 mmol), pivaloyl chloride (49.0 µL, 0.4 mmol) and MTBE (2.0 mL) for 15 mins, followed by dimethyl (3,5-dimethoxybenzoyl)phosphonate (54.8 mg, 0.2 mmol), (2*S*,3*R*)−HyperBTM (3.1 mg, 10.0 µmol) and *N*,*N*-diisopropylethylamine (34.9 µL, 0.2 mmol) for 16 h gave, after purification by Biotage® Isolera™ 4 [SNAP KP-Sil 10 g, 36 mL min^{-1}, petrol : Et_2O (90:10 to 0:100, 30 CV)], the title compound (53 mg, 80%) as a pale yellow oil. $[\alpha]_D^{20}$ −46.1 (*c*, 0.4, $CHCl_3$); **Chiral HPLC analysis** Chiralcel OD-H (90:10 hexane:IPA, flow rate 1.0 mLmin^{-1}, 211nm, 30 ℃), t_R(2*S*,3*S*): 10.6 min, t_R(2*R*,3*R*): 14.6 min, >99:1 er; **IR** ν_{max} (film) 1832 (C=O), 1026 (P−O); **^{1}H NMR** (500 MHz, $CDCl_3$) δ_H: 6.67 – 6.68 (2H, m, ArC(2, 6)*H*), 6.47 – 6.49 (1H, m, ArC(3)*H*), 4.27 (1H, dq, *J* 10.6, 7.7, C(3)*H*), 3.91 (3H, d, *J* 10.5, OCH_3), 3.83 (6H, s, ArOCH_3), 3.64 (3H, d, *J* 10.4, OCH_3), 1.09 (3H, d, *J* 7.7, C(3)CH_3); **^{31}P NMR** (202 MHz, $CDCl_3$) δ_P: 18.5; **^{13}C{^{1}H} NMR** (126 MHz, $CDCl_3$) δ_C: 169.8 (d, *J* 7.8, *C*(2)), 161.2 (d, *J* 1.6, Ar*C*(3,5)), 133.7 (d, *J* 6.2, Ar*C*(1)), 104.2 (d, *J* 4.1, Ar*C*(2,6)H), 100.8 (d, *J* 2.3, Ar*C*(4)H), 78.9 (d, *J* 169.6, *C*(4)), 55.5 (ArO*C*H_3), 55.2 (d, *J* 6.9, O*C*H_3), 54.4 (d, *J* 7.6, O*C*H_3), 53.5 (*C*(3)H), 10.5 (d, *J* 2.3,C(3)*C*H_3); **HRMS** (ESI$^+$) $C_{14}H_{19}O_7NaP$ [M+Na]$^+$ found 353.0748, requires 353.0761 (−3.4 ppm).

Dimethyl ((2*R*,3*R*)-3-methyl-2-(naphthalen-2-yl)-4-oxooxetan-2-yl)phosphonate (26)

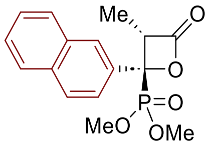

26

Following General Procedure D, 2-(trimethylsilyl)propanoic acid (47 mg, 0.32 mmol), *N*,*N*-diisopropylethylamine (69.7 µL, 0.4 mmol), pivaloyl chloride (49.0 µL, 0.4 mmol) and MTBE (2.0 mL) for 15 mins, followed by dimethyl (2-naphthoyl)phosphonate (52.8 mg, 0.2 mmol), (2*S*,3*R*)−HyperBTM (3.1 mg, 10.0 µmol) and *N*,*N*-diisopropylethylamine (34.9 µL, 0.2 mmol) rt for 16 h gave, after purification by Biotage® Isolera™ 4 [SNAP KP-Sil 10 g, 36 mL min^{-1}, petrol : Et_2O (90:10 to 0:100, 30 CV)], the title compound (52 mg, 81%) as a white solid. **mp** 66 – 70 °C; $[\alpha]_D^{20}$ −23.0 (*c*, 0.2, $CHCl_3$); **Chiral HPLC analysis** Chiralcel OD-H (90:10 hexane:IPA, flow rate 1.0 mLmin^{-1}, 211 nm, 30 ℃), t_R(2*S*,3*S*): 10.0 min, t_R(2*R*,3*R*): 13.7 min, >99:1 er; **IR** ν_{max} (film) 1838 (C=O), 1032 (P−O); **^{1}H NMR** (400 MHz, $CDCl_3$) δ_H: 8.05 (1H, t, *J* 2.2, Ar*H*), 7.88 – 7.95 (3H, m, Ar*H*), 7.62 (1H, dt, *J* 8.6, 1.6, Ar*H*), 7.55 – 7.59 (2H, m, Ar*H*),

4.40 (1H, dq, *J* 10.6, 7.8, C(3)*H*), 3.95 (3H, d, *J* 10.5, OC*H*3), 3.56 (3H, d, *J* 10.4, OC*H*3), 1.09 (3H, d, *J* 7.7, C(3)C*H*3); **^{31}P NMR** (162 MHz, $CDCl_3$) δ_P: 18.4; **^{13}C{^{1}H} NMR** (101 MHz, $CDCl_3$) δ_C: 170.0 (d, *J* 7.7, *C*(2)), 133.2 (d, *J* 1.6,Ar*C*(2)), 133.0 (d, *J* 1.7, Ar*C*(9)), 129.0 (d, *J* 5.4, Ar*C*(10)), 128.5 (Ar*C*(3)H), 128.4 (Ar*C*(5)H), 127.8 (Ar*C*(8)H), 127.0 (Ar*C*(4)H), 126.8 (Ar*C*(1)H), 125.9 (d, *J* 5.8, Ar*C*(7)H), 123.5 (d, *J* 2.5, Ar*C*(6)H), 79.2 (d, *J* 169.8, *C*(4)), 55.2 (d, *J* 7.0, O*C*H3), 54.5 (d, *J* 7.6, O*C*H3), 53.6 (*C*(3)H), 10.7 (d, *J* 2.3,C(3)*C*H3); **HRMS** (ESI$^+$) $C_{16}H_{17}O_5NaP$ [M+Na]$^+$ found 343.0699, requires 343.0706 (−2.0 ppm).

Dimethyl ((2*R*,3*R*)-2-(furan-3-yl)-3-methyl-4-oxooxetan-2-yl)phosphonate (27)

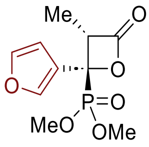

27

Following General Procedure D, 2-(trimethylsilyl)propanoic acid (58.5 mg, 0.4 mmol), *N*,*N*-diisopropylethylamine (69.7 μL, 0.4 mmol), pivaloyl chloride (49.0 μL, 0.4 mmol) and MTBE (2.0 mL) for 15 mins, followed by dimethyl (furan-3-carbonyl)phosphonate (40.8 mg, 0.2 mmol), (2*S*,3*R*)−HyperBTM (3.1 mg, 10.0 μmol) and *N*,*N*-diisopropylethylamine (34.9 μL, 0.2 mmol) for 16 h gave, after purification by Biotage® Isolera™ 4 [SNAP KP-Sil 10 g, 36 mL min^{-1}, petrol : Et_2O (90:10 to 0:100, 30 CV)], the title compound (36 mg, 68%) as a pale yellow oil. $[\alpha]_D^{20}$ −46.0 (*c*, 0.4, $CHCl_3$); **Chiral HPLC analysis** Chiralcel OJ-H (90:10 hexane:IPA, flow rate 1.0 mLmin^{-1}, 211 nm, 30 ℃), t_R(2*S*,3*S*): 15.3 min, t_R(2*R*,3*R*): 17.1 min, >99:1 er; **IR** ν_{max} (film) 1834 (C=O), 1022 (P−O); **^{1}H NMR** (400 MHz, $CDCl_3$) δ_H: 7.60 – 7.61 (1H, m, ArC(2)*H*), 7.52 – 7.52 (1H, m, ArC(5)*H*), 6.52 – 6.53 (1H, m, ArC(4)*H*, 4.25 (1H, dq, *J* 10.3, 7.6, C(3)*H*), 3.91 (3H, d, *J* 10.5, OC*H*3), 3.75 (3H, d, *J* 10.5, OC*H*3), 1.14 (3H, d, *J* 7.7, C(3)C*H*3); **^{31}P NMR** (162 MHz, $CDCl_3$) δ_P: 18.1; **^{13}C{^{1}H} NMR** (101 MHz, $CDCl_3$) δ_C: 169.7 (d, *J* 8.5, *C*(2)), 144.2 (Ar*C*(5)H), 141.0 (d, *J* 7.4, Ar*C*(2)H), 117.9 (d, *J* 6.7, Ar*C*(3)), 109.1 (d, *J* 2.6, Ar*C*(4)H), 75.1 (d, *J* 175.2, *C*(4)), 55.1 (d, *J* 7.2, O*C*H3), 54.3 (d, *J* 7.2, O*C*H3), 53.5 (d, *J* 2.1, *C*(3)H), 10.2 (d, *J* 2.1, C(3)*C*H3); **HRMS** (ESI$^+$) $C_{10}H_{13}NaO_6P$ [M+Na]$^+$ found 283.0337, requires 283.0342 (−1.8 ppm).

Dimethyl ((2*R*,3*R*)-3-methyl-4-oxo-2-(thiophen-3-yl)oxetan-2-yl)phosphonate (28)

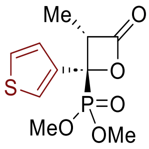

28

Following General Procedure D, 2-(trimethylsilyl)propanoic acid (58.5 mg, 0.4 mmol), *N*,*N*-diisopropylethylamine (69.7 μL, 0.4 mmol), pivaloyl chloride (49.0 μL, 0.4 mmol) and MTBE (2.0 mL) for

15 mins, followed by dimethyl (thiophene-3-carbonyl)phosphonate (44.0 mg, 0.2 mmol), (2*S*,3*R*)−HyperBTM (3.1 mg, 10.0 μmol) and *N*,*N*-diisopropylethylamine (34.9 μL, 0.2 mmol) for 16 h gave, after purification by Biotage® Isolera™ 4 [SNAP KP-Sil 10 g, 36 mL min^{-1}, petrol : Et_2O (90:10 to 0:100, 30 CV)], the title compound (45 mg, 82%) as a yellow oil. $[\alpha]_D^{20}$ −50.9 (*c*, 0.4, $CHCl_3$); **Chiral HPLC analysis** Chiralcel OD-H (90:10 hexane:IPA, flow rate 1.0 mLmin^{-1}, 211 nm, 30 ℃), t_R(2*S*,3*S*): 10.6 min, t_R(2*R*,3*R*): 14.6 min, >99:1 er; **IR** ν_{max} (film) 1836 (C=O), 1026 (P−O); **^{1}H NMR** (500 MHz, $CDCl_3$) δ_H: 7.49 – 7.50 (1H, m, ArC(2)*H*), 7.44 – 7.46 (1H, m, ArC(5)*H*), 7.21 (1H, dt, *J* 5.1, 1.2, ArC(4)*H*, 4.25 (1H, dq, *J* 10.4, 7.7, C(3)*H*), 3.91 (3H, d, *J* 10.6, OC*H*$_3$), 3.63 (3H, d, *J* 10.5, OC*H*$_3$), 1.06 (3H, d, *J* 7.7, C(3)C*H*$_3$); **^{31}P NMR** (202 MHz, $CDCl_3$) δ_P: 17.9; **^{13}C{^{1}H} NMR** (126 MHz, $CDCl_3$) δ_C: 169.9 (d, *J* 8.3, *C*(2)), 132.6 (d, *J* 6.7, Ar*C*(3)), 127.2 (Ar*C*(5)H), 125.8 (d, *J* 2.3, Ar*C*(4)H), 123.7 (d, *J* 6.6, Ar*C*(2)H), 77.8 (d, *J* 172.1, *C*(4)), 55.0 (d, *J* 6.8, O*C*H$_3$), 54.3 (d, *J* 7.5, O*C*H$_3$), 53.7 (d, *J* 1.7, *C*(3)H), 10.2 (d, *J* 2.2, C(3)*C*H$_3$); **HRMS** (ESI$^+$) $C_{10}H_{13}NaO_5PS$ [M+Na]$^+$ found 299.0104, requires 299.0114 (−3.1 ppm).

Dimethyl ((2*S*,3*R*)-2,3-dimethyl-4-oxooxetan-2-yl)phosphonate (29)

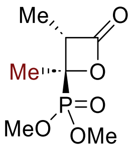

29

Following General Procedure D, 2-(trimethylsilyl)propanoic acid (58.5 mg, 0.4 mmol), *N*,*N*-diisopropylethylamine (69.7 μL, 0.4 mmol), pivaloyl chloride (49.0 μL, 0.4 mmol) and MTBE (2.0 mL) for 15 mins, followed by dimethyl acetylphosphonate (30.4 mg, 0.2 mmol), (2*S*,3*R*)−HyperBTM (3.1 mg, 10.0 μmol) and *N*,*N*-diisopropylethylamine (34.9 μL, 0.2 mmol) for 16 h gave, after purification by Biotage® Isolera™ 4 [SNAP KP-Sil 10 g, 36 mL min^{-1}, petrol : Et_2O (90:10 to 0:100, 30 CV)], the title compound (26 mg, 61%) as a coloourless oil. $[\alpha]_D^{20}$ −20.4 (*c*, 0.3, $CHCl_3$); **Chiral GC analysis**, Rt-βDEXsm Column (110 ℃ hold for 60 min, 1.0 ℃min^{-1}, 110 – 130 ℃, 0.5 ℃min^{-1}), t_R(2*R*,3*R*): 115.2 min, t_R(2*S*,3*S*): 116.9 min, >99:1 er; **IR** ν_{max} (film) 1825 (C=O), 1011 (P−O); **^{1}H NMR** (400 MHz, $CDCl_3$) δ_H: 4.06 (1H, dq, *J* 11.5, 7.7, C(3)*H*), 3.88 (6H, dd, *J* 10.5, 3.5, OC*H*$_3$), 1.68 (3H, d, *J* 14.5, C(4)C*H*$_3$), 1.34 (3H, d, *J* 7.6, C(3)C*H*$_3$); **^{31}P NMR** (162 MHz, $CDCl_3$) δ_P: 22.1; **^{13}C{^{1}H} NMR** (101 MHz, $CDCl_3$) δ_C: 170.2 (d, *J* 9.5, *C*(2)), 75.7 (d, *J* 172.9, *C*(4)), 54.3 (d, *J* 6.9, O*C*H$_3$), 54.0 (d, *J* 7.0, O*C*H$_3$), 50.7 (d, *J* 1.10, *C*(3)H), 15.9 (d, *J* 7.0,C(4)*C*H$_3$), 8.5 (d, *J* 2.8,C(3)*C*H$_3$); **HRMS** (ESI$^+$) $C_7H_{14}O_5P$ [M+H]$^+$ found 209.0572, requires 209.0573 (−0.9 ppm).

Dimethyl (3-oxopentanoyl)phosphonate (S36)

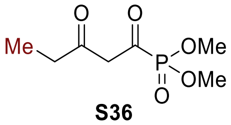

S36

Following General Procedure D, 2-(trimethylsilyl)propanoic acid (58.5 mg, 0.4 mmol), *N*,*N*-diisopropylethylamine (69.7 µL, 0.4 mmol), pivaloyl chloride (49.0 µL, 0.4 mmol) and MTBE (2.0 mL) for 15 mins, followed by dimethyl acetylphosphonate (30.4 mg, 0.2 mmol), (2*S*,3*R*)−HyperBTM (3.1 mg, 10.0 µmol) and *N*,*N*-diisopropylethylamine (34.9 µL, 0.2 mmol) for 16 h gave, after purification by Biotage® Isolera™ 4 [SNAP KP-Sil 10 g, 36 mL min^{-1}, petrol : Et_2O (90:10 to 30:70, 30 CV)], the title compound (9 mg, 22%) as a colourless oil. **IR** ν_{max} (film) 1016 (P−O); **^{1}H NMR** (500 MHz, $CDCl_3$) δ_H: 6.08 (1H, dd, *J* 11.2, 2.2, COCH$_A$*H*$_B$CO), 5.79 (1H, dd, *J* 35.4, 2.1, COC*H*$_A$H$_B$CO), 3.80 (6H, d, *J* 11.2, OC*H*$_3$), 2.52 (2H, q, *J* 7.5, CH$_3$C*H*$_2$), 1.23 (3H, t, *J* 7.6, C*H*$_3$); **^{31}P NMR** (162 MHz, $CDCl_3$) δ_P: 10.7; **^{13}C{^{1}H} NMR** (126 MHz, $CDCl_3$) δ_C: 172.0 (d, *J* 2.7, CH$_3$CH$_2$CO), 144.8 (d, *J* 222.4, *C*OPO), 121.7 (CO*C*H$_2$CO), 53.3 (d, *J* 5.2, O*C*H$_3$), 27.4 (*C*HCH$_3$), 8.9 (CH$_2$*C*H$_3$); **HRMS** (ESI$^+$) $C_7H_{13}O_5NaP$ [M+Na]$^+$ found 231.0388, requires 231.0393 (−2.1 ppm).

Dimethyl ((2*R*,3*R*)-2-(4-bromophenyl)-3-methyl-4-oxooxetan-2-yl)phosphonate (30)

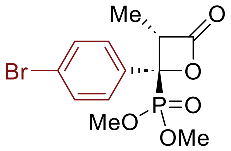

30

Following General Procedure D, 2-(trimethylsilyl)propanoic acid (58.5 mg, 0.4 mmol), *N*,*N*-diisopropylethylamine (69.7 µL, 0.4 mmol), pivaloyl chloride (49.0 µL, 0.4 mmol) and MTBE (2.0 mL) for 15 mins, followed by dimethyl (4-bromobenzoyl)phosphonate (58.2 mg, 0.2 mmol), (2*S*,3*R*)−HyperBTM (3.1 mg, 10.0 µmol) and *N*,*N*-diisopropylethylamine (34.9 µL, 0.2 mmol) for 16 h gave, after purification by Biotage® Isolera™ 4 [SNAP KP-Sil 10 g, 36 mL min^{-1}, petrol : Et_2O (90:10 to 0:100, 30 CV)], the title compound (53 mg, 76%) as a pale yellow oil. $[\alpha]_D^{20}$ −37.8 (*c*, 0.4, $CHCl_3$); **Chiral HPLC analysis** Chiralcel OD-H (90:10 hexane:IPA, flow rate 1.0 mLmin^{-1}, 211 nm, 30 ℃), t_R(2*S*,3*S*): 8.8 min, t_R(2*R*,3*R*): 11.6 min, >99:1 er; **IR** ν_{max} (film) 1836 (C=O), 1026 (P−O); **^{1}H NMR** (400 MHz, $CDCl_3$) δ_H: 7.60 – 7.63 (2H, m, ArC(3,5)*H*), 7.39 – 7.43 (2H, m, ArC(2,6)*H*), 4.31 (1H, dq, *J* 10.6, 7.7, C(3)*H*), 3.91 (3H, d, *J* 10.5, OC*H*$_3$), 3.62 (3H, d, *J* 10.5, OC*H*$_3$), 1.06 (3H, d, *J* 7.8, C(3)C*H*$_3$); **^{31}P NMR** (162 MHz, $CDCl_3$) δ_P: 18.0; **^{13}C{^{1}H} NMR** (101 MHz, $CDCl_3$) δ_C: 169.3 (d, *J* 7.8, *C*(2)), 132.0 (d, *J* 1.9, Ar*C*(3,5)H), 130.7 (d, *J* 5.8, Ar*C*(1)), 128.0 (d, *J* 4.1, Ar*C*(2,6)H), 123.4 (d, *J* 3.2, Ar*C*(4)), 78.5 (d, *J* 170.0, *C*(4)), 55.2 (d, *J* 6.9, O*C*H$_3$), 54.5

(d, *J* 7.5, O*C*H$_3$), 53.5 (*C*(3)H), 10.7 (d, *J* 2.3,C(3)*C*H$_3$); **HRMS** (ESI$^+$) $C_{12}H_{14}{}^{79}BrNaO_5P$ [M+Na]$^+$ found 370.9648, requires 370.9654 (−1.7 ppm).

Dimethyl ((1*R*,2*R*)-1-(4-bromophenyl)-1-hydroxy-2-methyl-3-morpholino-3-oxopropyl)phosphonate (31)

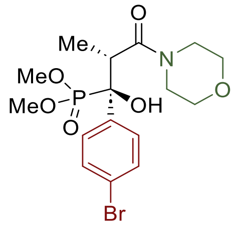

31

Following General Procedure D, 2-(trimethylsilyl)propanoic acid (58.5 mg, 0.4 mmol), *N*,*N*-diisopropylethylamine (69.7 µL, 0.4 mmol), pivaloyl chloride (49.0 µL, 0.4 mmol) and MTBE (2.0 mL) for 15 mins, followed by dimethyl (4-bromobenzoyl)phosphonate (58.2 mg, 0.2 mmol) (2*S*,3*R*)−HyperBTM (3.1 mg, 10.0 µmol) and *N*,*N*-diisopropylethylamine (34.9 µL, 0.2 mmol) for 16 h, than morpholine (104.5 mg, 1.2 mmol) added, rt for 4 h gave, after purification by Biotage® Isolera™ 4 [SNAP KP-Sil 10 g, 36 mL min^{-1}, CH_2Cl_2 : MeOH (100:0 to 95:5, 30 CV)], the title compound (82 mg, 94%) as a white foam. $[\alpha]_D^{20}$ +59.8 (*c*, 0.6, CHCl$_3$); **Chiral HPLC analysis** Chiralcel OD-H (95:5 hexane:IPA, flow rate 0.5 mLmin^{-1}, 211 nm, 30 ℃), t_R(2*S*,3*S*): 34.4 min, t_R(2*R*,3*R*): 38.7 min, >99:1 er; **IR** ν_{max} (film) 1609 (C=O), 1234 (P=O), 1026 (P−O); **mp** 158 – 161 °C; **^{1}H NMR** (500 MHz, CDCl$_3$) δ_H: 7.57 – 7.60 (2H, m, ArC(3,5)*H*), 7.52 – 7.53 (2H, m, ArC(2,6)*H*), 6.88 (1H, s, O*H*), 3.58 – 3.90 (3H, m, NC*H*$_2$, C*H*$_2$O), 3.79 (3H, *J* 10.1, OC*H*$_3$), 3.70 – 3.78 (3H, m, C*H*$_2$O), 3.53 – 3.63 (3H, m, NC*H*$_2$, C*H*CH$_3$), 3.35 (3H, d, *J* 10.1, OC*H*$_3$), 0.86 (3H, d, *J* 7.0, CHC*H*$_3$); **^{31}P NMR** (162 MHz, CDCl$_3$) δ_P: 22.7; **^{13}C{^{1}H} NMR** (126 MHz, CDCl$_3$) δ_C: 174.9 (*C*O), 137.4 (d, *J* 1.7, Ar*C*(1)), 131.5 (Ar*C*(3,5)H), 127.9 (Ar*C*(2,6)H), 121.9 (d, *J* 3.9, Ar*C*(4)), 77.8 (d, *J* 161.1, *C*PO), 66.6 (d, *J* 6.8, *C*H$_2$O), 54.5 (d, *J* 2.5, O*C*H$_3$), 54.5 (d, *J* 3.8, O*C*H$_3$), 46.8 (N*C*$_B$H$_2$), 42.3 (N*C*$_A$H$_2$), 37.0 (d, *J* 6.5, C*H*CH$_3$), 12.5 (d, *J* 10.7, CHC*H*$_3$); **HRMS** (ESI$^+$) $C_{16}H_{23}{}^{79}BrNaO_6P$ [M+Na]$^+$ found 458.0328, requires 458.0339 (−2.3 ppm).

Dimethyl (E)-(1-(furan-2-yl)prop-1-en-1-yl)phosphonate (35)

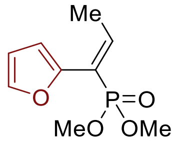

35

Following General Procedure D, 2-(trimethylsilyl)propanoic acid (58.5 mg, 0.4 mmol), *N*,*N*-diisopropylethylamine (69.7 µL, 0.4 mmol), pivaloyl chloride (49.0 µL, 0.4 mmol) and MTBE (2.0 mL) for 15 mins, followed by dimethyl (furan-2-carbonyl)phosphonate (40.8 mg, 0.2 mmol), (2*S*,3*R*)−HyperBTM (3.1 mg, 10.0 µmol) and *N*,*N*-diisopropylethylamine (34.9 µL, 0.2 mmol) for 16 h gave, after purification by Biotage® Isolera™ 4 [SNAP KP-Sil 10 g, 36 mL min^{-1}, petrol : Et_2O (90:10 to 0:100, 30 CV)], the title compound (28 mg, 65%) as a pale yellow oil. **IR** ν_{max} (film) 1015 (P−O), 826 (C=C); **^{1}H NMR** (500 MHz, $CDCl_3$) δ_H: 7.48 (1H, d, *J* 1.4, ArC(5)*H*), 6.96 (1H, dq, *J* 23.4, 7.2, C*H*), 6.53 (1H, d, *J* 3.5, ArC(3)*H*), 6.45 (1H, ddd, *J* 3.4, 1.8, 0.4, ArC(4)*H*), 3.75 (6H, d, *J* 11.2, OCH_3), 2.15 (3H, dd, *J* 7.3, 3.3, CH_3); **^{31}P NMR** (202 MHz, $CDCl_3$) δ_P: 20.5; **^{13}C{^{1}H} NMR** (126 MHz, $CDCl_3$) δ_C: 148.8 (d, *J* 19.9, Ar*C*(2)), 145.0 (d, *J* 7.2, C=*C*H), 142.4 (Ar*C*(5)H), 120.1 (d, *J* 186.8, *C*=CH), 111.1 (d, *J* 3.0, Ar*C*(3)H), 111.0 (Ar*C*(4)H), 52.7 (d, *J* 5.5, O*C*H_3), 16.7 (d, *J* 19.0, CH*C*H_3); **HRMS** (ESI$^+$) $C_9H_{14}O_4P$ $[M+H]^+$ found 217.0618, requires 217.0624 (−3.1 ppm).

Dimethyl ((2*R*,3*R*)-2-(4-bromophenyl)-3-ethyl-4-oxooxetan-2-yl)phosphonate (36)

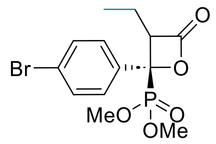

36

Following General Procedure D, 2-(trimethylsilyl)butanoic acid (64.1 mg, 0.4 mmol), *N*,*N*-diisopropylethylamine (69.7 µL, 0.4 mmol), pivaloyl chloride (49.0 µL, 0.4 mmol) and MTBE (2.0 mL) for 15 mins, followed by dimethyl (4-bromobenzoyl)phosphonate (58.2 mg, 0.2 mmol), (2*S*,3*R*)−HyperBTM (3.1 mg, 10.0 µmol) and *N*,*N*-diisopropylethylamine (34.9 µL, 0.2 mmol) for 16 h gave, after purification by Biotage® Isolera™ 4 [SNAP KP-Sil 10 g, 36 mL min^{-1}, petrol : Et_2O (90:10 to 0:100, 30 CV)], the title compound (67 mg, 92%) as a colourless oil. $[\alpha]_D^{20}$ −45.7 (*c*, 0.5, $CHCl_3$); **Chiral HPLC analysis** Chiralcel OD-H (90:10 hexane:IPA, flow rate 1.0 mLmin^{-1}, 211 nm, 30 ℃), t_R(2*S*,3*S*): 7.4 min, t_R(2*R*,3*R*): 8.2 min, >99:1 er; **IR** ν_{max} (film) 1834 (C=O), 1028 (P−O); **^{1}H NMR** (400 MHz, $CDCl_3$) δ_H: 7.58 – 7.62 (2H, m, ArC(3,5)*H*), 7.43 – 7.47 (2H, m, ArC(2,6)*H*), 4.12 (1H, td, *J* 10.5, 6.1, C(3)*H*), 3.92 (3H, d, *J* 10.6, OCH_3), 3.62 (3H, d, *J* 10.5, OCH_3), 1.44 – 1.53 (1H, m, CH_2CH$_3$), 1.22 – 1.30 (1H, m, CH_2CH$_3$), 1.00 (3H, t, *J* 7.4, CH$_2$CH_3); **^{31}P NMR** (162 MHz, $CDCl_3$) δ_P:18.1; **^{13}C{^{1}H} NMR** (101 MHz, $CDCl_3$) δ_C: 168.8 (d, *J* 7.5, *C*(2)), 131.9 (d, *J* 1.8, Ar*C*(3,5)H), 131.0 (d, *J* 6.0, Ar*C*(1)H), 127.8 (d, *J* 4.2, Ar*C*(2,6)H), 123.3 (d, *J* 3.1, Ar*C*(4)), 78.2 (d, *J* 170.0, *C*(4)), 59.7 (*C*(3)H), 55.2 (d, *J* 7.0, O*C*H$_3$), 54.5 (d, *J* 7.8, O*C*H$_3$), 19.9 (d, *J* 2.4, *C*H$_2$CH$_3$), 11.1 (CH$_2$*C*H$_3$); **HRMS** (ESI$^+$) $C_{13}H_{16}{}^{79}BrNaO_5P$ $[M+Na]^+$ found 384.9803, requires 384.9811 (−2.1 ppm).

Dimethyl ((2*R*,3*R*)-2-(4-bromophenyl)-4-oxo-3-propyloxetan-2-yl)phosphonate (37)

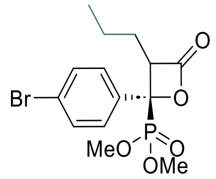

37

Following General Procedure D, 2-(trimethylsilyl)pentanoic acid (69.7 mg, 0.4 mmol), *N*,*N*-diisopropylethylamine (69.7 µL, 0.4 mmol), pivaloyl chloride (49.0 µL, 0.4 mmol) and MTBE (2.0 mL) for 15 mins, followed by dimethyl (4-bromobenzoyl)phosphonate (58.2 mg, 0.2 mmol), (2*S*,3*R*)−HyperBTM (3.1 mg, 10.0 µmol) and *N*,*N*-diisopropylethylamine (34.9 µL, 0.2 mmol) for 16 h gave, after purification by Biotage® Isolera™ 4 [SNAP KP-Sil 10 g, 36 mL min^{-1}, petrol : Et_2O (90:10 to 0:100, 30 CV)], the title compound (68 mg, 90%) as a pale yellow oil. $[\alpha]_D^{20}$ −35.4 (*c*, 0.6, $CHCl_3$); **Chiral HPLC analysis** Chiralcel OJ-H (95:5 hexane:IPA, flow rate 1.0 mLmin^{-1}, 211 nm, 30 ℃), t_R(2*S*,3*S*): 10.2 min, t_R(2*R*,3*R*): 11.9 min, >99:1 er; **IR** ν_{max} (film) 1834 (C=O), 1026 (P−O); **^{1}H NMR** (500 MHz, $CDCl_3$) δ_H: 7.60 – 7.61 (2H, m, ArC(3,5)*H*), 7.44 – 7.46 (2H, m, ArC(2,6)*H*), 4.18 (1H, td, *J* 10.6, 5.8, C(3)*H*), 3.92 (3H, d, *J* 10.6, OCH_3), 3.62 (3H, d, *J* 10.5, OCH_3), 1.48 – 1.57 (1H, m, CH_2CH$_2$), 1.34 – 1.44 (2H, m, CH_2CH_2CH$_3$), 1.15 – 1.23 (1H, m, CH_2CH$_3$), 0.86 (3H, t, *J* 7.3, CH_3); **^{31}P NMR** (202 MHz, $CDCl_3$) δ_P: 18.1; **^{13}C{^{1}H} NMR** (101 MHz, $CDCl_3$) δ_C: 168.9 (d, *J* 7.7, *C*(2)), 131.9 (d, *J* 1.8, Ar*C*(3,5)H), 131.1 (d, *J* 6.2, Ar*C*(1)), 127.9 (d, *J* 4.2, Ar*C*(2,6)H), 123.4 (d, *J* 3.2, Ar*C*(4)), 78.2 (d, *J* 170.4, *C*(4)), 58.2 (*C*(3)H), 55.2 (d, *J* 6.9, O*C*H$_3$), 54.5 (d, *J* 7.4, O*C*H$_3$), 28.3 (d, *J* 2.2, C(3)H*C*H$_2$), 19.9 (*C*H$_2$CH$_3$), 13.7 (*C*H$_3$); **HRMS** (ESI$^+$) $C_{14}H_{18}{}^{79}BrNaO_5P$ [M+Na]$^+$ found 398.9964, requires 398.9967 (−0.9 ppm).

Dimethyl ((2*R*,3*R*)-3-allyl-2-(4-bromophenyl)-4-oxooxetan-2-yl)phosphonate (38)

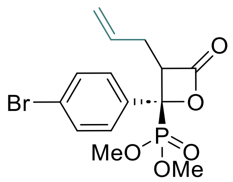

38

Following General Procedure D, 2-(trimethylsilyl)pent-4-enoic acid (68.9 mg, 0.4 mmol), *N*,*N*-diisopropylethylamine (69.7 µL, 0.4 mmol), pivaloyl chloride (49.0 µL, 0.4 mmol) and MTBE (2.0 mL) for 15 mins, followed by dimethyl (4-bromobenzoyl)phosphonate (58.2 mg, 0.2 mmol), (2*S*,3*R*)−HyperBTM (3.1 mg, 10.0 µmol) and *N*,*N*-diisopropylethylamine (34.9 µL, 0.2 mmol) for 16 h gave, after purification by Biotage® Isolera™ 4 [SNAP KP-Sil 10 g, 36 mL min^{-1}, petrol : Et_2O (90:10 to 0:100, 30 CV)], the title compound (58 mg, 77%) as a yellow oil. $[\alpha]_D^{20}$ −30.7 (*c*, 0.5, $CHCl_3$); **Chiral HPLC analysis** Chiralcel OJ-H (90:10 hexane:IPA, flow rate 1.0 mLmin^{-1}, 211 nm, 30 ℃), t_R(2*S*,3*S*): 9.7 min, t_R(2*R*,3*R*): 13.1 min,

>99:1 er; **IR** ν_{max} (film) 1840 (C=O), 1032 (P−O); **^{1}H NMR** (400 MHz, $CDCl_3$) δ_H: 7.59 – 7.62 (2H, m, ArC(3,5)*H*), 7.44 – 7.48 (2H, m, ArC(2,6)*H*), 5.65 – 5.75 (1H, m, C*H*=CH_2), 5.06 – 5.13 (2H, m, CH=CH_2), 4.28 – 4.34 (1H, m, C(3)*H*), 3.93 (3H, d, *J* 10.5, OCH_3), 3.62 (3H, d, *J* 10.5, OCH_3), 2.04 – 2.22 (2H, m, C(3)HCH_2); **^{31}P NMR** (162 MHz, $CDCl_3$) δ_P: 17.7; **^{13}C{^{1}H} NMR** (101 MHz, $CDCl_3$) δ_C: 168.2 (d, *J* 7.8, *C*(2)), 132.0 (*C*H=CH_2), 131.9 (d, *J* 1.8, Ar*C*(3,5)H), 130.7 (d, *J* 5.9, Ar*C*(1)H), 128.0 (d, *J* 4.1, Ar*C*(2,6)H), 123.5 (d, *J* 3.1, Ar*C*(4)), 78.4 (d, *J* 169.4, *C*(4)), 118.4 (CH=*C*H_2), 57.5 (d, *J* 1.4, *C*(3)H), 55.3 (d, *J* 6.9, O*C*H_3), 54.6 (d, *J* 7.6, O*C*H_3), 30.0 (d, J 2.4, C(3)*C*H_2); **HRMS** (ESI$^+$) $C_{14}H_{16}{}^{79}BrNaO_5P$ [M+Na]$^+$ found 396.9804, requires 396.9811 (−1.8 ppm).

Dimethyl ((2*R*,3*R*)-2-(4-bromophenyl)-4-oxo-3-(prop-2-yn-1-yl)oxetan-2-yl)phosphonate (39)

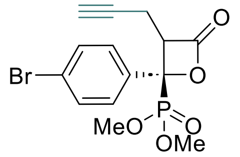

39

Following General Procedure D, 2-(trimethylsilyl)pent-4-ynoic acid (68.1 mg, 0.4 mmol), *N*,*N*-diisopropylethylamine (69.7 µL, 0.4 mmol), pivaloyl chloride (49.0 µL, 0.4 mmol) and MTBE (2.0 mL) for 15 mins, followed by dimethyl (4-bromobenzoyl)phosphonate (58.2 mg, 0.2 mmol), (2*S*,3*R*)−HyperBTM (3.1 mg, 10.0 µmol) and *N*,*N*-diisopropylethylamine (34.9 µL, 0.2 mmol) for 16 h gave, after purification by Biotage® Isolera™ 4 [SNAP KP-Sil 10 g, 36 mL min^{-1}, petrol : Et_2O (90:10 to 0:100, 30 CV)], the title compound (55 mg, 74%) as a yellow oil. $[\alpha]_D^{20}$ −0.6 (*c*, 0.5, $CHCl_3$); **Chiral HPLC analysis** Chiralcel OJ-H (90:10 hexane:IPA, flow rate 1.0 mL min^{-1}, 211 nm, 30 ℃), t_R(2*S*,3*S*): 20.2 min, t_R(2*R*,3*R*): 30.2 min, >99:1 er; **IR** ν_{max} (film) 1842 (C=O), 1028 (P−O); **^{1}H NMR** (500 MHz, $CDCl_3$) δ_H: 7.60 – 7.62 (2H, m, ArC(3,5)*H*), 7.48 – 7.51 (2H, m, ArC(2,6)*H*), 4.41 – 4.46 (1H, m, C(3)*H*), 3.91 (3H, d, *J* 10.7, OCH_3), 3.65 (3H, d, *J* 10.5, OCH_3), 2.26 – 2.41 (2H, m, C(3)HCH_2), 2.04 (1H, t, *J* 2.7, C≡C*H*); **^{31}P NMR** (202 MHz, $CDCl_3$) δ_P: 17.2; **^{13}C{^{1}H} NMR** (126 MHz, $CDCl_3$) δ_C: 166.6 (d, *J* 7.8, *C*(2)), 132.0 (d, *J* 1.9, Ar*C*(3,5)H), 129.9 (d, *J* 5.3, Ar*C*(1)H), 128.1 (d, *J* 4.1, Ar*C*(2,6)H), 123.8 (d, *J* 3.2, Ar*C*(4)), 78.4 (d, *J* 170.7, *C*(4)), 71.7 (C≡*C*H), 71.7 (*C*≡CH), 57.1 (C≡*C*H), 57.1 (d, *J* 2.0, *C*(3)), 55.4 (d, *J* 7.0, O*C*H_3), 54.6 (d, *J* 7.5, O*C*H_3), 15.7 (d, *J* 2.7, C(3)*C*H_2); **HRMS** (ESI$^+$) $C_{14}H_{15}{}^{79}BrO_5P$ [M+H]$^+$ found 372.9833, requires 372.9835 (−0.5 ppm).

Dimethyl ((2*R*,3*R*)-3-benzyl-2-(4-bromophenyl)-4-oxooxetan-2-yl)phosphonate (40)

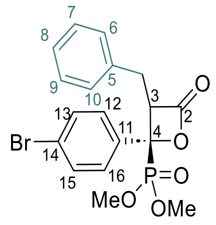

40

Following General Procedure D, 3-phenyl-2-(trimethylsilyl)propanoic acid (88.9 mg, 0.4 mmol), *N*,*N*-diisopropylethylamine (69.7 µL, 0.4 mmol), pivaloyl chloride (49.0 µL, 0.4 mmol) and MTBE (2.0 mL) for 15 mins, followed by dimethyl (4-bromobenzoyl)phosphonate (58.2 mg, 0.2 mmol), (2*S*,3*R*)−HyperBTM (3.1 mg, 10.0 µmol) and *N*,*N*-diisopropylethylamine (34.9 µL, 0.2 mmol) for 16 h gave, after purification by Biotage® Isolera™ 4 [SNAP KP-Sil 10 g, 36 mL min^{-1}, petrol : Et_2O (90:10 to 0:100, 30 CV)], the title compound (65 mg, 76%) as a pale yellow oil. $[\alpha]_D^{20}$ +7.3 (*c*, 0.5, $CHCl_3$); **Chiral HPLC analysis** Chiralcel OJ-H (90:10 hexane:IPA, flow rate 1.0 $mLmin^{-1}$, 211 nm, 30 ℃), t_R(2*S*,3*S*): 20.1 min, t_R(2*R*,3*R*): 30.9 min, >99:1 er; **IR** ν_{max} (film) 1836 (C=O), 1028 (P−O); **^{1}H NMR** (500 MHz, $CDCl_3$) δ_H: 7.58 – 7.60 (2H, m, C(13,15)*H*), 7.43 – 7.44 (2H, m, C(7,9)*H*), 7.24 – 7.32 (3H, m, C(6,8,10)*H*), 7.12 – 7.14 (2H, m, C(12,16)*H*), 4.55 (1H, td, *J* 9.9, 7.0, C(3)*H*), 3.90 (3H, d, *J* 10.6, OC*H*$_3$), 3.62 (3H, d, *J* 10.5, OC*H*$_3$), 2.67 (2H, ddd, *J* 43.0, 15.0, 7.0, C(3)C*H*$_2$); **^{31}P NMR** (202 MHz, $CDCl_3$) δ_P: 17.7; **^{13}C{^{1}H} NMR** (126 MHz, $CDCl_3$) δ_C: 168.1 (d, *J* 7.5, *C*(2)), 135.7 (*C*(5)), 131.9 (d, *J* 1.5, *C*(13,15)H), 130.7 (d, *J* 6.0, *C*(11)), 128.6 (*C*(7,9)H), 128.6 (*C*(6,10)H), 128.1 (d, *J* 4.1, *C*(12,16)H), 127.1 (*C*(8)H), 123.6 (d, *J* 2.9, *C*(14)), 78.5 (d, *J* 169.9, *C*(4)), 58.8 (*C*(3)H), 55.2 (d, *J* 7.0, O*C*H$_3$), 54.5 (d, *J* 7.7, O*C*H$_3$), 31.9 (d, *J* 2.2, C(3)*C*H$_2$); **HRMS** (ESI^+) $C_{18}H_{18}{}^{79}BrNaO_5P$ $[M+Na]^+$ found 446.9962, requires 446.9967 (−1.2 ppm).

Dimethyl ((2*R*,3*R*)-2-(4-bromophenyl)-3-(4-methylbenzyl)-4-oxooxetan-2-yl)phosphonate (41)

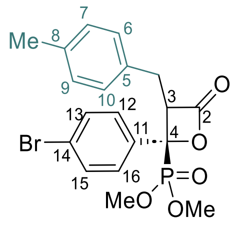

41

Following General Procedure D, 3-(*p*-tolyl)-2-(trimethylsilyl)propanoic acid (94.6 mg, 0.4 mmol), *N*,*N*-diisopropylethylamine (69.7 µL, 0.4 mmol), pivaloyl chloride (49.0 µL, 0.4 mmol) and MTBE (2.0 mL) for 15 mins, followed by dimethyl (4-bromobenzoyl)phosphonate (58.2 mg, 0.2 mmol), (2*S*,3*R*)−HyperBTM (3.1 mg, 10.0 µmol) and *N*,*N*-diisopropylethylamine (34.9 µL, 0.2 mmol) for 16 h gave, after purification by Biotage® Isolera™ 4 [SNAP KP-Sil 10 g, 36 mL min^{-1}, petrol : Et_2O (90:10 to 0:100, 30 CV)], the title

compound (68 mg, 79%) as a pale yellow oil. $[\alpha]_D^{20}$ +9.8 (*c*, 0.3, $CHCl_3$); **Chiral HPLC analysis** Chiralcel OJ-H (90:10 hexane:IPA, flow rate 1.0 mLmin^{-1}, 211 nm, 30 ℃), t_R(2*S*,3*S*): 14.4 min, t_R(2*R*,3*R*): 25.3 min, >99:1 er; **IR** ν_{max} (film) 1838 (C=O), 1032 (P−O); **^{1}H NMR** (400 MHz, $CDCl_3$) δ_H: 7.58 – 7.61 (2H, m, C(13,15)*H*), 7.42 – 7.46 (2H, m, C(12,16)*H*), 7.10 – 7.12 (2H, m, C(7,9)*H*), 7.01 – 7.03 (2H, m, C(6,10)*H*), 4.52 (1H, td, *J* 9.8, 6.9, C(3)*H*), 3.90 (3H, d, *J* 10.6, OCH_3), 3.62 (3H, d, *J* 10.5, OCH_3), 2.63 (2H, ddd, *J* 43.0, 15.0, 7.0, C(3)CH_2), 2.34 (3H, s, ArCH_3); **^{31}P NMR** (202 MHz, $CDCl_3$) δ_P: 17.8; **^{13}C{^{1}H} NMR** (101 MHz, $CDCl_3$) δ_C: 168.2 (d, *J* 7.5, *C*(2)), 136.7 (*C*(8)), 132.5 (*C*(5)), 131.9 (d, *J* 1.6, *C*(13,15)H), 130.7 (d, *J* 5.9, *C*(11)), 129.4 (*C*(7,9)H), 128.5 (*C*(6,10)H), 128.1 (d, *J* 4.2, *C*(12,16)H), 123.5 (d, *J* 3.0, *C*(14)), 78.5 (d, *J* 169.3, *C*(4)), 58.9 (*C*(3)), 55.3 (d, *J* 7.0, O*C*H_3), 54.6 (d, *J* 7.6, O*C*H_3), 31.6 (d, *J* 2.2, C(3)*C*H_2), 21.1 (Ar*C*H_3); **HRMS** (ESI$^+$) $C_{19}H_{20}{}^{79}BrNaO_5P$ [M+Na]$^+$ found 461.0117, requires 461.0124 (−1.5 ppm).

Dimethyl ((2*R*,3*R*)-2-(4-bromophenyl)-3-(4-fluorobenzyl)-4-oxooxetan-2-yl)phosphonate (42)

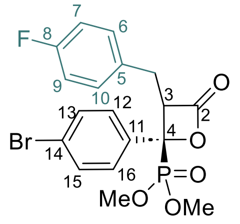

42

Following General Procedure D, 3-(4-fluorophenyl)-2-(trimethylsilyl)propanoic acid (96.1 mg, 0.4 mmol), *N*,*N*-diisopropylethylamine (69.7 µL, 0.4 mmol), pivaloyl chloride (49.0 µL, 0.4 mmol) and MTBE (2.0 mL) for 15 mins, followed by dimethyl (4-bromobenzoyl)phosphonate (58.2 mg, 0.2 mmol), (2*S*,3*R*)−HyperBTM (3.1 mg, 10.0 µmol) and *N*,*N*-diisopropylethylamine (34.9 µL, 0.2 mmol) for 16 h gave, after purification by Biotage® Isolera™ 4 [SNAP KP-Sil 10 g, 36 mL min^{-1}, petrol : Et_2O (90:10 to 0:100, 30 CV)], the title compound (72 mg, 82%) as a pale yellow oil. $[\alpha]_D^{20}$ +1.9 (*c*, 0.3, $CHCl_3$); **Chiral HPLC analysis** Chiralcel OJ-H (90:10 hexane:IPA, flow rate 1.0 mLmin^{-1}, 211 nm, 30 ℃), t_R(2*S*,3*S*): 19.0 min, t_R(2*R*,3*R*): 36.7 min, >99:1 er; **IR** ν_{max} (film) 1838 (C=O), 1030 (P−O); **^{1}H NMR** (500 MHz, $CDCl_3$) δ_H: 7.59 – 7.61 (2H, m, C(13,15)*H*), 7.42 – 7.46 (2H, m, C(12,16)*H*), 7.07 – 7.11 (2H, m, C(6,10)*H*), 6.96 – 7.01 (2H, m, C(7,9)*H*), 4.48 (1H, td, *J* 10.0, 6.7, C(3)*H*), 3.91 (3H, d, *J* 10.5, OCH_3), 3.62 (3H, d, *J* 10.5, OCH_3), 2.63 (2H, ddd, *J* 61.1, 14.9, 7.0, C(3)CH_2); **^{19}F NMR** (471 MHz, $CDCl_3$) δ_F: −115.5 (s); **^{31}P NMR** (202 MHz, $CDCl_3$) δ_P: 17.6; **^{13}C{^{1}H} NMR** (126 MHz, $CDCl_3$) δ_C: 168.0 (d, *J* 7.6, *C*(2)), 161.9 (d, *J* 246.1, *C*(8)), 132.0 (d, *J* 1.5, *C*(13,15)H), 131.3 (d, *J* 3.2, *C*(5)), 130.6 (d, *J* 6.0, *C*(11)), 130.2 (d, *J* 8.0, *C*(6,10)H), 128.0 (d, *J* 4.2, *C*(12,16)H), 123.7 (d, *J* 3.1, *C*(14)), 115.5 (d, *J* 21.2, *C*(7,9)H), 78.4 (d, *J* 169.7, *C*(4)), 58.8 (*C*(3)), 55.3 (d, *J* 7.0, O*C*H_3), 54.6 (d, *J* 7.7, O*C*H_3), 31.2 (d, *J* 2.0, C(3)*C*H_2); **HRMS** (ESI$^+$) $C_{18}H_{17}{}^{79}BrFNaO_5P$ [M+Na]$^+$ found 464.9860, requires 464.9873 (−2.8 ppm).

Dimethyl ((2*R*,3*R*)-2-(4-bromophenyl)-3-(naphthalen-2-ylmethyl)-4-oxooxetan-2-yl)phosphonate (43)

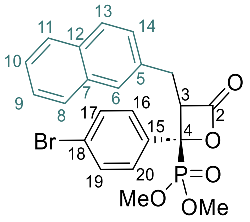

43

Following General Procedure D, 3-(naphthalen-2-yl)-2-(trimethylsilyl)propanoic acid (109.0 mg, 0.4 mmol), *N*,*N*-diisopropylethylamine (69.7 μL, 0.4 mmol), pivaloyl chloride (49.0 μL, 0.4 mmol) and MTBE (2.0 mL) for 15 mins, followed by dimethyl (4-bromobenzoyl)phosphonate (58.2 mg, 0.2 mmol), (2*S*,3*R*)−HyperBTM (3.1 mg, 10.0 μmol) and *N*,*N*-diisopropylethylamine (34.9 μL, 0.2 mmol) for 16 h gave, after purification by Biotage® Isolera™ 4 [SNAP KP-Sil 10 g, 36 mL min^{-1}, petrol : Et_2O (90:10 to 0:100, 30 CV)], the title compound (76 mg, 80%) as a yellow oil. $[\boldsymbol{\alpha}]_{\mathbf{D}}^{\mathbf{20}}$ +39.3 (*c*, 0.3, $CHCl_3$); **Chiral HPLC analysis** Chiralcel OD-H (95:5 hexane:IPA, flow rate 0.5 mLmin^{-1}, 211 nm, 30 ℃), t_R(2*S*,3*S*): 60.7 min, t_R(2*R*,3*R*): 66.7 min, >99:1 er; **IR** ν_{max} (film) 1842 (C=O), 1034 (P−O); **^{1}H NMR** (500 MHz, $CDCl_3$) δ_H: 7.77 – 7.84 (3H, m, Ar*H*), 7.57 – 7.59 (2H, m, C(17,19)*H*), 7.47 – 7.54 (3H, m, Ar*H*), 7.43 – 7.47 (2H, m, C(16,20)*H*), 7.27 (1H, dd, *J* 8.5, 1.8, Ar*H*), 4.64 – 4.70 (1H, m, C(3)*H*), 3.91 (3H, d, *J* 10.5, OCH_3), 3.63 (3H, d, *J* 10.5, OCH_3), 2.85 (2H, ddd, *J* 25.0, 15.0, 7.0, C(3)HCH_2); **^{31}P NMR** (202 MHz, $CDCl_3$) δ_P: 17.8; **^{13}C{^{1}H} NMR** (1026 MHz, $CDCl_3$) δ_C: 168.1 (d, *J* 7.5, *C*(2)), 133.4 (*C*(6)), 133.0 (*C*(13)), 132.4 (*C*(14)), 132.0 (d, *J* 1.6, *C*(17,19)H), 130.6 (d, *J* 6.0, *C*(5)), 128.4 (*C*(9)H), 128.1 (d, *J* 4.2, *C*(16,20)H), 127.7 (CH_2Ar*C*(7,12)), 127.3 (*C*(15)H), 126.7 (*C*(8)H), 126.3 (*C*(11)H), 125.9 (*C*(10)H), 123.6 (d, *J* 3.1, *C*(18)), 78.5 (d, *J* 169.1, *C*(4)), 58.7 (d, *J* 1.3, *C*(3)H), 55.3 (d, *J* 6.7, O*C*H_3), 54.6 (d, *J* 7.6, O*C*H_3), 32.0 (d, J 2.2, C(3)*C*H_2); **HRMS** (ESI$^+$) $C_{22}H_{20}{}^{79}BrNaO_5P$ $[M+Na]^+$ found 497.0119, requires 497.0124 (−1.0 ppm).

Dimethyl ((2*R*,3*R*)-4-oxo-2,3-diphenyloxetan-2-yl)phosphonate (44)

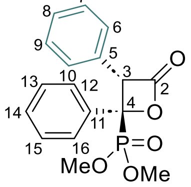

44

Following General Procedure D, 2-phenyl-2-(trimethylsilyl)acetic acid (83.3 mg, 0.4 mmol), *N*,*N*-diisopropylethylamine (69.7 μL, 0.4 mmol), pivaloyl chloride (49.0 μL, 0.4 mmol) and MTBE (2.0 mL) for

15 mins, followed by dimethyl benzoylphosphonate (42.8 mg, 0.2 mmol), (2*S*,3*R*)−HyperBTM (3.1 mg, 10.0 μmol) and *N*,*N*-diisopropylethylamine (34.9 μL, 0.2 mmol) for 16 h gave, after purification by Biotage® Isolera™ 4 [SNAP KP-Sil 10 g, 36 mL min^{-1}, petrol : Et_2O (90:10 to 10:90, 30 CV)], the compound (27 mg, 40%) as a colourless oil. $[\alpha]_D^{20}$ +81.3 (*c*, 0.5, $CHCl_3$); **Chiral HPLC analysis** Chiralpak ID (90:10 hexane:IPA, flow rate 1.0 mLmin^{-1}, 211 nm, 30 ℃), t_R (2*S*,3*S*): 19.7 min, t_R (2*R*,3*R*): 21.4 min, 99:1 er; **IR** ν_{max} (film) 1842 (C=O), 1030 (P−O); **^{1}H NMR** (400 MHz, $CDCl_3$) δ_H: 7.14 – 7.22 (10H, m, Ar*H*), 6.91 – 6.94 (2H, m, Ph*H*), 5.57 (1H, d, *J* 10.9, C(3)*H*), 3.96 (3H, d, *J* 10.5, OC*H*$_3$), 3.60 (3H, d, *J* 10.5, OC*H*$_3$); **^{31}P NMR** (162 MHz, $CDCl_3$) δ_P: 18.1; **^{13}C{^{1}H} NMR** (101 MHz, $CDCl_3$) δ_C: 167.9 (d, *J* 8.6, *C*(2)), 131.0 (d, *J* 5.2, *C*(11)), 129.6 (*C*(6,10)H), 129.1 (d, *J* 2.8, *C*(5)), 128.7 (C(8)H), 128.5 (*C*(7,9)H), 128.4 (d, *J* 2.8, *C*(14)H), 128.0 (d, *J* 2.0, *C*(13,15)H), 126.8 (d, *J* 4.0, *C*(12,16)H), 80.3 (d, *J* 170.0, *C*(4)), 64.9 (d, *J* 1.7, *C*(3)H), 55.2 (d, *J* 7.0, O*C*H$_3$), 54.6 (d, *J* 7.6, O*C*H$_3$); **HRMS** (ESI$^+$) $C_{17}H_{17}NaO_5P$ [M+Na]$^+$ found 355.0702, requires 355.0706 (−1.0 ppm). *Please see page 53 for characterisation of the same compound as a mixture of diastereomers following re-optimisation of the reaction.*

7. Catalysis Products – C(3)-Aryl

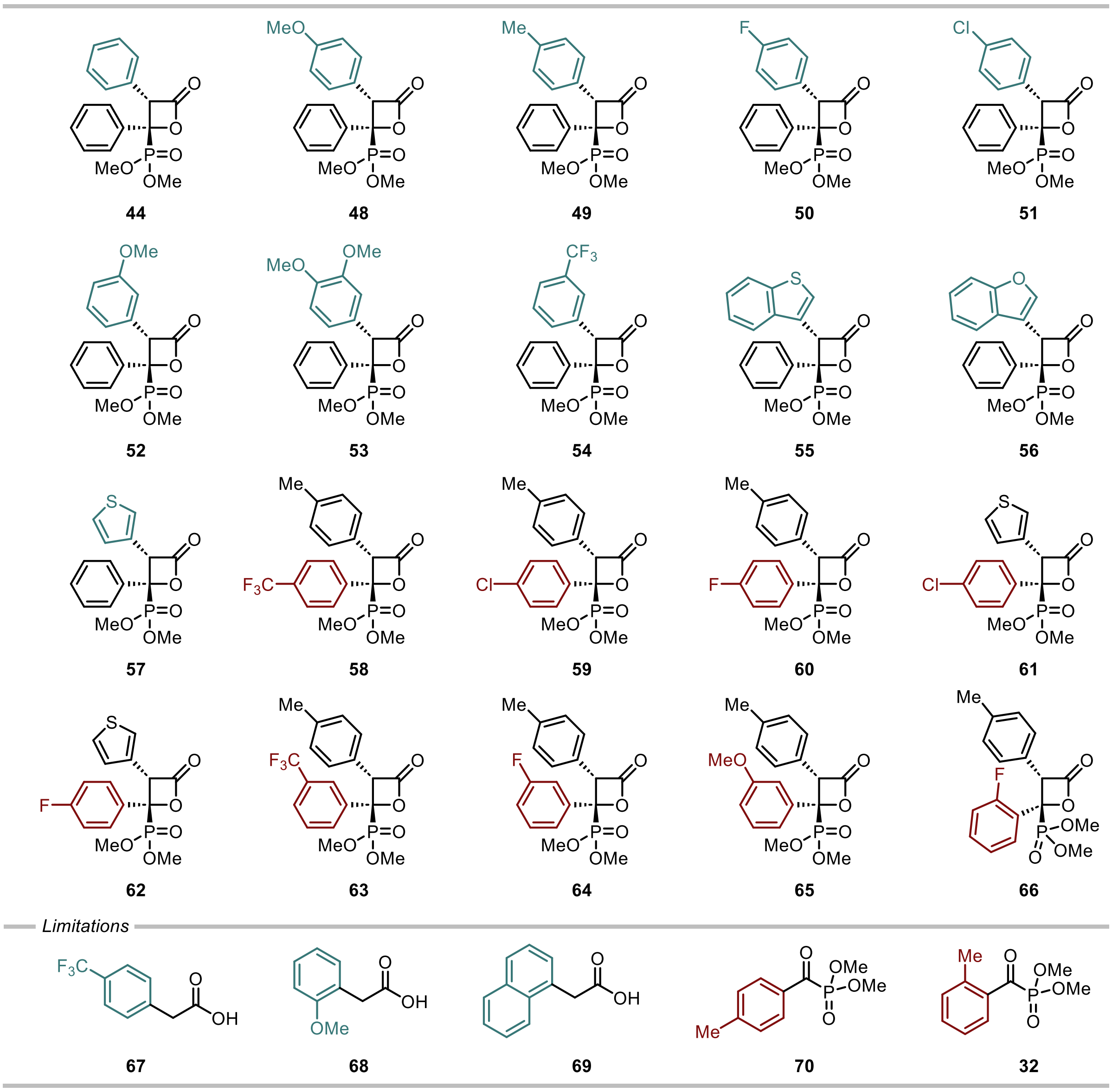

Dimethyl ((2*R*,3*R*)-4-oxo-2,3-diphenyloxetan-2-yl)phosphonate (*anti*-44) and dimethyl ((2*R*,3*S*)-4-oxo-2,3-diphenyloxetan-2-yl)phosphonate (*syn*-44)

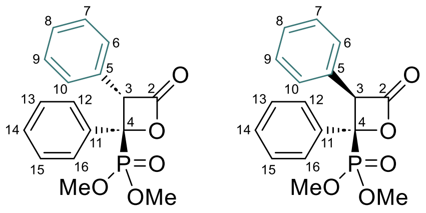

anti-**44** *syn*-**44**

Following **General Procedure D** with pivaloyl chloride (74 µL, 0.6 mmol), *i*-Pr_2NEt (105 µL, 0.6 mmol) and phenylacetic acid **48** (81.7 mg, 0.6 mmol) in MTBE (1.6 mL, 0.375 M). Then *i*-Pr_2NEt (35 µL, 0.2 mmol), (2*S*,3*R*)-HyperBTM (3.1 mg, 5 mol%), and dimethyl benzoylphosphonate **4** (35 µL, 0.2 mmol) in MTBE (0.4 mL, 0.5 M) at 25 °C for 16 h gave the crude product (90:10 $dr_{anti:syn}$). The crude mixture was purified by flash silica column chromatography [toluene : Et_2O (75:25 40 mL, 72.5:27.5 40 mL, 70:30 40 mL, 67.5:32.5 40 mL, 65:35 40 mL), R_f = 0.25 (*syn*-**44**) and 0.13 (*anti*-**44**)], then the solvents removed *in vacuo* to yield the combined *anti* and *syn*-diastereomers (83:17 $dr_{anti:syn}$) (46.5 mg, 70 %) as an inseparable mixture and a colourless oil with data in accordance with the literature.[28] $[\alpha]_D^{20}$ +81.3 (c, 0.5, $CHCl_3$); **IR** ν_{max} (film) 1842 (C=O), 1030 (P−O); **HRMS** (ESI$^+$) $C_{17}H_{17}NaO_5P$ $[M+Na]^+$ found 355.0702, requires 355.0706 (−1.0 ppm)

*Data for major diastereomer anti-***44**: **Chiral HPLC analysis** Chiralpak AD-H (95:5 hexane : IPA, flow rate 2.0 mLmin^{-1}, 220 nm, 30 ℃) t_R(2*S*,3*S*): 8.5 min, t_R(2*R*,3*R*): 10.7 min 96:4 er_{anti}; **^{1}H NMR** (400 MHz, $CDCl_3$) δ_H: 7.22-7.11 (8H, m, C(7,8,9)*H* and C(11-16)*H*), 6.92-6.88 (2H, m, C(6,10)*H*), 5.55 (1H, d *J* 10.9 Hz, C(3)*H*), 3.94 (3H, d *J* 10.6 Hz, POC*H*$_3$), 3.58 (3H, d *J* 10.6 Hz, POC*H*$_3$); **^{13}C{^{1}H} NMR** 167.9 (d, *J* 8.9, *C*(2)), 131.0 (d, *J* 4.2, *C*(11)), 129.6 (*C*(6,10)H), 129.1 (d, *J* 3.2, (*C*(5)), 128.7 (*C*(4)H), 128.5 (*C*(7,9)H), 128.4 (*C*(14)H), 128.0 (d, *J* 2.5, *C*(13,15)H), 126.8 (d, *J* 4.0, *C*(12,16)H), 80.4 (d, *J* 170.0, *C*(4)), 64.9 (d, *J* 2.1, *C*(3)), 55.2 (d, *J* 7.0, PO*C*H$_3$), 54.6 (d, *J* 7.7, PO*C*H$_3$); **^{31}P{^{1}H} NMR** (162 MHz, $CDCl_3$) δ_P: 18.1 ppm.

*Data for minor diastereomer syn-***44**: **Chiral HPLC analysis** Chiralpak AD-H (95:5 hexane : IPA, flow rate 2.0 mLmin^{-1}, 220 nm, 30 ℃) t_R(2*S*,3*R*): 12.9 min, t_R(2*R*,3*S*): 17.1 min 96:4 er_{syn}; **^{1}H NMR** (400 MHz, $CDCl_3$) δ_H: 8.12-8.07 (1H, m, C(8)*H*), 7.71-7.66 (2H, m, C(12,16)*H*), 7.60-7.56 (2H, m, C(7,9)*H*), 7.53-7.42 (4H, m, C(6,10)*H* and C(13,15)*H*), 7.12-7.10 (1H, m, C(14)*H*), 5.20 (1H, d, *J* 13.2, C(3)*H*), 3.38 (3H, d, *J* 10.8, POC*H*$_3$), 3.37 (3H, d, *J* 10.7, POC*H*$_3$); **^{13}C{^{1}H} NMR** (101 MHz, $CDCl_3$) δ_C: 130.1 (*C*(8)H), 128.8 *C*(7,9)H), 128.7 *C*(13,15)H), 128.6 (*C*(6,10)H), 128.4 (*C*(14)H), 125.7 (d, *J* 3.6, *C*(12,16)H), 67.4 (d, *J* 3.0, *C*(3)), 54.4 (d, *J* 7.0, PO*C*H$_3$), 53.4 (d, *J* 7.0, PO*C*H$_3$); **^{31}P{^{1}H} NMR** (162 MHz, $CDCl_3$) δ_P: 15.7 ppm.

Dimethyl ((2*R*,3*R*)-3-(4-methoxyphenyl)-4-oxo-2-phenyloxetan-2-yl)phosphonate (*anti*-48) and dimethyl ((2*R*,3*S*)-3-(4-methoxyphenyl)-4-oxo-2-phenyloxetan-2-yl)phosphonate (*syn*-48)

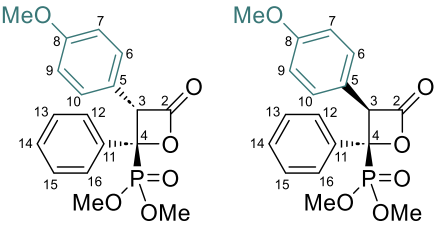

anti-**48** *syn*-**48**

Following **General Procedure D** with pivaloyl chloride (74 µL, 0.6 mmol), *i*-Pr_2NEt (105 µL, 0.6 mmol) and 4-methoxyphenylacetic acid (99.7 mg, 0.6 mmol) in MTBE (1.6 mL, 0.375 M). Then *i*-Pr_2NEt (35 µL, 0.2 mmol), (2*S*,3*R*)-HyperBTM (3.1 mg, 5 mol%), and dimethyl benzoylphosphonate **4** (35 µL, 0.2 mmol) in MTBE (0.4 mL, 0.5 M) at 25 °C for 16 h gave the crude product (91:9 $dr_{anti:syn}$). The crude mixture was purified by flash silica column chromatography [formic acid : toluene : Et_2O (1:40:59 40 mL, 1:45:54 40 mL, 1:50:49 40 mL, 1:55:44 40 mL, 1:60:39 40 mL), R_f = 0.18 (*syn*-**48**) and 0.09 (*anti*-**48**)], then the solvents removed *in vacuo* to yield the combined *anti* and *syn*-diastereomers (86:14 $dr_{anti:syn}$) (40.6 mg, 56%) as an inseparable mixture and a yellow oil. $[\alpha]_D^{20}$ + 69.6 (*c* 1.1, $CHCl_3$); **IR** ν_{max} (film) 1836 (C=O), 1028 (P−O); **HRMS** $[ESI]^+$ $C_{17}H_{17}NaO_5P$ $[M+Na]^+$ found 385.0811, requires 385.0805 (−1.68 ppm).

Data for major diastereomer anti-**48: Chiral HPLC analysis** Chiralpak AD-H (95:5 hexane : IPA, flow rate 2.0 mLmin^{-1}, 220 nm, 30 ℃) t_R(2*S*,3*S*): 13.8 min, t_R(2*R*,3*R*): 15.8 min, 95:5 er_{anti}. **^{1}H NMR** ($CDCl_3$, 400 MHz) δ_H: 7.23-7.11 (5H, m, C(12-16)*H*), 6.82-6.76 (2H, dt, *J* 8.4, 2.0, C(7,9)*H*), 6.69-6.63 (2H, dt, *J* 8.8, 2.4, C(6,10)*H*), 5.50 (1H, d, *J* 10.8, C(3)*H*), 3.93 (3H, d, *J* 10.5, POCH_3), 3.71 (3H, s, OCH_3), 3.57 (3H, d, *J* 10.5, POCH_3); **^{13}C{^{1}H} NMR** (101 MHz, $CDCl_3$) δ_C: 168.5 (*C*(2)), 159.7 (*C*(8)OMe), 131.1 (d, *J* 5.4, *C*(11)), 130.9 *C*(6,10)H), 128.4 (d, *J* 2.3, *C*(14)H), 128.0 (d, *J* 2.3, *C*(13,15)H), 126.9 (d, *J* 3.9, *C*(12,16)H), 121.1 (*C*(5)), 113.9 (*C*(7,9)H), 80.6 (d, *J* 169.0, *C*(4)), 64.4 (d, *J* 2.1, *C*(3)H), 55.2 (O*C*H_3), 55.2 (d, *J* 7.6, PO*C*H_3), 54.5 (d, *J* 7.6, PO*C*H_3); **^{31}P{^{1}H} NMR** (162 MHz, $CDCl_3$) δ_P: 18.3 ppm.

Data for minor diastereomer syn-**48**: **Chiral HPLC analysis** Chiralpak AD-H (95:5 hexane : IPA, flow rate 2.0 mLmin^{-1}, 220 nm, 30 ℃) t_R(2*S*,3*R*): 20.9 min, t_R(2*R*,3*S*): 30.0 min, 88:12 er_{syn}. **^{1}H NMR** ($CDCl_3$, 400 MHz) δ_H: 7.69-7.63 (2H, C(12,16)*H*), 7.52-7.40 (5H, m, C(6,10)*H* and C(13,14,15)*H*), 6.99-6.95 (2H, m, C(7,9)*H*), 5.14 (1H, d, *J* 12.7, C(3)*H*), 3.83 (3H, s, OCH_3), 3.45 (3H, d, *J* 10.8, POCH_3), 3.36 (3H, d, *J* 10.6, POCH_3); **^{13}C{^{1}H} NMR** (101 MHz, $CDCl_3$) δ_C: 168.4 (*C*(2)), 159.8 (*C*(8)OMe), 130.3 (*C*(6,10)H), 128.9 (d, *J* 2.6, *C*(14)H), 128.4 (d, *J* 2.3, *C*(13,15)H), 125.7 (d, *J* 3.9, *C*(12,16)H), 121.0 (*C*(5)), 113.9 (*C*(7,9)H), 84.2 (d, *J* 212.5, *C*(4)), 67.1 (d, *J* 2.7, *C*(3)H), 55.3 (O*C*H_3), 54.4 (d, *J* 6.7, PO*C*H_3), 53.6 (d, *J* 7.3, PO*C*H_3);; **^{31}P{^{1}H} NMR** (162 MHz, $CDCl_3$) δ_P: 15.9 ppm.

Dimethyl ((2*R*,3*R*)-4-oxo-2-phenyl-3-(p-tolyl)oxetan-2-yl)phosphonate (*anti*-49)

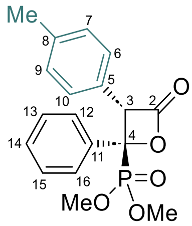

anti-**49**

Following **General Procedure D** with pivaloyl chloride (74 µL, 0.6 mmol), *i*-Pr_2NEt (105 µL, 0.6 mmol) and 4-tolylacetic acid (90.1 mg, 0.6 mmol) in MTBE (1.6 mL, 0.375 M). Then *i*-Pr_2NEt (35 µL, 0.2 mmol), (2*S*,3*R*)-HyperBTM (3.1 mg, 5 mol%), and dimethyl benzoylphosphonate **4** (35 µL, 0.2 mmol) in MTBE (0.4 mL, 0.5 M) at 25 °C for 16 h gave the crude product (>95:5 $dr_{anti:syn}$). The crude mixture was purified by flash column chromatography on silica gel [toluene : EtOAc 70:30 100 mL, 65:35 100 mL, 60:40 100 mL, 55:45 100 mL), R_f = 0.23 (*anti*-**49**), then the solvents removed *in vacuo* to yield the product as a single diastereomer (44.3 mg, 64%) and a colourless oil. $[\alpha]_D^{20}$ + 41.8 (*c* 0.85, $CHCl_3$); **IR** ν_{max} (film) 1842 (C=O), 1261 (P=O), 1059 (P-O), 1032 (P-O); **HRMS** $[ESI]^+$ $C_{18}H_{19}O_5NaP$ $[M+Na]^+$ found 369.0851, requires 369.0862 (−3.07 ppm). **Chiral HPLC analysis** Chiralpak AD-H (97:3 hexane : IPA, flow rate 1.5 mLmin^{-1}, 220 nm, 30 ℃) t_R(2*S*,3*S*): 20.3 min, t_R(2*R*,3*R*): 22.8 min, 95:5 er_{anti}. **^{1}H NMR** (400 MHz, $CDCl_3$): δ_H: 7.67 (2H, d, *J* 7.6, C(7,9)*H*), 7.52-7.42 (5H, m, C(12-16)*H*), 7.01-6.97 (2H, m, C(6,10)*H*), 5.16 (1H, d, *J* 12.6, C(3)*H*), 3.41 (3H, d, *J* 11.0, POCH_3), 3.36 (3H, d, *J* 10.6, POCH_3), 2.27 (3H, s, C(3)ArC(4)CH_3); **^{13}C{^{1}H} NMR** (101 MHz, $CDCl_3$) δ_C: 137.9 (*C*(8)CH_3), 129.1 (*C*(6,10)H), 128.2 (*C*(7,9)H), 125.7 (*C*(5)), 67.3 (*C*(3)H), 53.5 (d, *J* 7.3, PO*C*H_3), 52.7 (d, *J* 8.0, PO*C*H_3), 21.5 (C(3)ArC(4)*C*H_3); **^{31}P{^{1}H} NMR** (162 MHz, $CDCl_3$) δ_P: 15.8 ppm.

Dimethyl ((2*R*,3*R*)-3-(4-fluorophenyl)-4-oxo-2-phenyloxetan-2-yl)phosphonate (*anti*-50) and dimethyl ((2*R*,3*S*)-3-(4-fluorophenyl)-4-oxo-2-phenyloxetan-2-yl)phosphonate (*syn*-50)

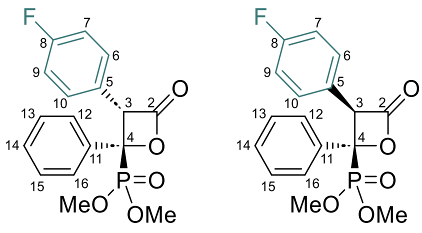

anti-**50** *syn*-**50**

Following **General Procedure D** with pivaloyl chloride (74 µL, 0.6 mmol), *i*-Pr_2NEt (105 µL, 0.6 mmol) and 4-fluorophenylacetic acid (92.5 mg, 0.6 mmol) in MTBE (1.6 mL, 0.375 M). Then *i*-Pr_2NEt (35 µL, 0.2 mmol), (2*S*,3*R*)-HyperBTM (3.1 mg, 5 mol%), and dimethyl benzoylphosphonate **4** (35 µL, 0.2 mmol) in MTBE (0.4 mL, 0.5 M) at 25 °C for 16 h gave the crude product (84:16 $dr_{anti:syn}$). The crude mixture was purified by flash silica column chromatography [toluene : Et_2O (80:20 150 mL, 75:25 150 mL, 70:30 150 mL, 65:35 150 mL), R_f = 0.06 (*syn*-**50**) and 0.15 (*anti*-**50**)], then the solvents removed *in vacuo* to yield *anti*-**51** as a colourless oil (49.4 mg, 71%) and *syn*-**51** as a colourless oil (9.4 mg, 13%).

*Data for major diastereomer anti-***50:** $[\alpha]_D^{20}$ + 57.6 (*c* 1.00, $CHCl_3$); **Chiral HPLC analysis** Chiralpak AD-H (95:5 hexane : IPA, flow rate 1.0 mLmin^{-1}, 211 nm, 30 ℃) t_R(2*S*,3*S*): 16.5 min, t_R(2*R*,3*R*): 20.0 min, 95:5 er_{anti}; **IR** ν_{max} (film) 1840 (C=O), 1514 (P=O), 1061 (P-O), 1032 (P-O); **^{1}H NMR** ($CDCl_3$, 400 MHz): 7.21-7.12 (5H, m, C(12-16)*H*), 6.90-6.79 (4H, m, (C6,7,9,10)*H*), 5.53 (1H, d, *J* 10.8, C(3)*H*), 3.94 (3H, d, *J* 10.5, POC*H*$_3$), 3.57 (3H, d, *J* 10.5, POC*H*$_3$); **^{13}C{^{1}H} NMR** (101 MHz, $CDCl_3$) δ_C: 167.7 (d, *J* 8.8, *C*(2)), 162.7 (d, *J* 248.8, *C*(8)F), 131.3 (d, *J* 8.5, *C*(6,10)H), 130.9 (d, *J* 5.2, *C*(11)), 128.6 (d, *J* 2.7, *C*(14)H), 128.2 (d, *J* 2.4, *C*(13,15)H), 126.7 (d, *J* 4.0, *C*(12,16)H), 125.1 (dd, *J* 3.4, 3.4, *C*(5)), 115.7 (d, *J* 21.8, *C*(7,9)H), 80.4 (d, *J* 169.3, *C*(4)), 64.1 (d, *J* 2.0, *C*(3), 55.3 (d, *J* 6.9, PO*C*H$_3$), 54.6 (d, *J* 7.5, PO*C*H$_3$); **^{19}F{^{1}H} NMR** (376 MHz, $CDCl_3$) δ_F: −112.15 ppm; **^{31}P{^{1}H} NMR** (162 MHz, $CDCl_3$) δ_P: 17.9 ppm; **HRMS** [ESI]$^+$ $C_{17}H_{16}O_5FNaP$ [M+Na]$^+$ found 373.0602, requires 373.0612 (−2.57 ppm).

*Data for minor diastereomer syn-***50**: $[\alpha]_D^{20}$ − 14.7 (*c* 0.30, $CHCl_3$); **Chiral HPLC analysis** Chiralpak AD-H (95:5 hexane : IPA, flow rate 1.0 mLmin^{-1}, 211 nm, 30 ℃) t_R(2*R*,3*S*): 27.8 min, t_R(2*S*,3*R*): 30.1 min, 94:6 $er_{anti.}$; **IR** ν_{max} (film) 1840 (C=O), 1225 (P=O), 1024 (P-O); **^{1}H NMR** ($CDCl_3$, 400 MHz): 7.68-7.64 (2H, m, C(12,16)*H*), 7.59-7.53 (2H, m, C(6,10)*H*), 7.53-7.47 (2H, m, C(13,15)*H*), 7.47-7.41 (1H, m, C(14)*H*), 7.20-7.13 (2H, m, *C*(7,9)H), 5.16 (dd, *J* 12.6, 1.0, C(3)*H*), 3.26 (d, *J* 10.8, POC*H*$_3$), 3.38 (d, *J* 10.6, POC*H*$_3$); **^{13}C{^{1}H} NMR** (101 MHz, $CDCl_3$) δ_C: 166.7 (d, *J* 4.2, *C*(2)), 161.6 (*C*(8)F), 136.0 (d, *J* 5.0, *C*(11)), 130.8 (d, *J* 8.5, *C*(6,10)H), 129.1 (d, *J* 2.9, *C*(14)H), 128.9 (dd, *J* 1.8, 0.3, *C*(13,15)H), 125.7 (d, *J* 3.5, *C*(12,16)H), 125.3 (d, *J* 2.7, *C*(5)), 115.6 (d, *J* 21.8, *C*(7,9)H), 81.5 (d, *J* 171.8, *C*(4)), 66.7 (d, *J* 2.7, *C*(3)H), 54.5 (d, *J* 7.1, PO*C*H$_3$), 53.5 (d, *J* 7.7, PO*C*H$_3$); **^{19}F{^{1}H} NMR** (MHz, $CDCl_3$) δ_F: −112.43 ppm;

^{31}P{^{1}H} NMR (162 MHz, $CDCl_3$) δ_P: 15.6 ppm. ; **HRMS** [ESI]$^+$ $C_{17}H_{16}O_5FNaP$ [M+Na]$^+$ found 373.0608, requires 373.0612 (−0.96 ppm).

Dimethyl ((2*R*,3*R*)-3-(4-chlorophenyl)-4-oxo-2-phenyloxetan-2-yl)phosphonate (*anti*-51) and dimethyl ((2*R*,3*S*)-3-(4-chlorophenyl)-4-oxo-2-phenyloxetan-2-yl)phosphonate (*syn*-51)

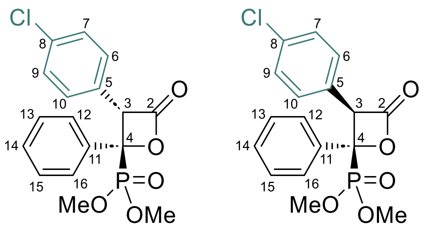

anti-**51** *syn*-**51**

Following **General Procedure D** with pivaloyl chloride (74 µL, 0.6 mmol), *i*-Pr_2NEt (105 µL, 0.6 mmol) and 4-fluorophenylacetic acid (92.5 mg, 0.6 mmol) in MTBE (1.6 mL, 0.375 M). Then *i*-Pr_2NEt (35 µL, 0.2 mmol), (2*S*,3*R*)-HyperBTM (3.1 mg, 5 mol%), and dimethyl benzoylphosphonate **4** (35 µL, 0.2 mmol) in MTBE (0.4 mL, 0.5 M) at 25 °C for 16 h gave the crude product (84:16 $dr_{anti:syn}$). The crude mixture was purified by flash silica column chromatography [toluene : Et_2O (80:20 150 mL, 75:25 150 mL, 70:30 150 mL, 65:35 150 mL), R_f = 0.06 (*syn*-**51**) and 0.15 (*anti*-**51**)], then the solvents removed *in vacuo* to yield *anti*-**51** as a colourless oil (36.7 mg, 50%) and *syn*-**51** as a colourless oil (8.0 mg, 11%).

*Data for major diastereomer anti-***51:** $[\alpha]_D^{20}$ + 93.0 (*c* 1.13, $CHCl_3$); **IR** ν_{max} (film) 1842 (C=O), 1495 (P=O), 1059 (P-O), 1032 (P-O); **Chiral HPLC analysis** Chiralpak AD-H (95:5 hexane : IPA, flow rate 1.0 mLmin^{-1}, 211 nm, 30 ℃) t_R(2*S*,3*S*): 20.3 min, t_R(2*R*,3*R*): 23.0 min, 95:5 er_{anti}. **^{1}H NMR** ($CDCl_3$, 400 MHz): 7.24-7.18 (3H, m, C(13-15)*H*), 7.16 (2H, ddd, *J* 7.8, 2.1, 2.1, C(12,16)*H*), 7.12 (2H, d, *J* 8.5, C(7,9)*H*), 6.83 (2H, d, *J* 8.5, C(6,10)*H*), 5.52 (1H, d, *J* 10.9, C(3)*H*), 3.94 (3H, d, *J* 10.5, POC*H*$_3$), 3.57 (3H, d, *J* 10.5, POC*H*$_3$); **^{13}C{^{1}H} NMR** (101 MHz, $CDCl_3$) δ_C: 167.4 (d, *J* 8.7, *C*(2)), 134.8 (*C*(8)Cl), 130.8 (*C*6,10)H), 130.8 (d, *J* 3.4, *C*(5)), 128.8 (*C*(7,9)H), 128.7 (d, *J* 2.8, *C*(11)), 128.2 (d, *J* 2.3, *C*(13,15)H), 127.6 (d, *J* 3.2, *C*(14)H), 126.7 (d, *J* 4.0, *C*(12,16)H), 80.3 (d, *J* 169.1, *C*(4)), 64.2 (d, *J* 2.1, *C*(3)H), 55.3 (d, *J* 6.9, PO*C*H$_3$), 54.6 (d, *J* 7.6, PO*C*H$_3$); **^{31}P{^{1}H} NMR** (162 MHz, $CDCl_3$) δ_P: 17.8 ppm ; **HRMS** [ESI]$^+$ $C_{17}H_{16}ClO_5NaP$ [M+Na]$^+$ found 389.0325, requires 389.0318 (+1.77 ppm).

*Data for minor diastereomer syn-***51**: $[\alpha]_D^{20}$ + 2.0 (*c* 0.26, $CHCl_3$); **IR** ν_{max} (film) 1842 (C=O), 1030 (P-O); **Chiral HPLC analysis** Chiralpak AD-H (95:5 hexane : IPA, flow rate 1.0 mLmin^{-1}, 211 nm, 30 ℃) t_R(2*R*,3*S*): 29.8 min, t_R(2*S*,3*R*): 35.2 min, 93:7 er_{syn}; **^{1}H NMR** ($CDCl_3$, 400 MHz): 7.67-7.63 (2H, m, C(12,16)*H*), 7.54-7.40 (7H, m, C(6,7,9,10)*H* and C(13-15)*H*), 5.15 (1H, d, *J* 12.5, C(3)*H*), 3.46 (3H, d, *J* 10.8, POC*H*$_3$), 3.39 (3H, d, *J* 10.6, POC*H*$_3$); **^{13}C{^{1}H} NMR** (101 MHz, $CDCl_3$) δ_C: 166.4 (d, *J* 4.0, *C*(2)), 134.8 (*C*(8)), 130.2 (*C*(6,10)H), 130.1 (*C*(5)), 129.1 (d, *J* 2.6, *C*(14)H), 128.9 (d, *J* 2.0, *C*(13,15)H), 128.8 (*C*(7,9)H), 128.0 (d, *J* 2.6, *C*(11)), 125.7 (d, *J* 4.0, *C*(12,16)H), 81.4 (d, *J* 171.5, *C*(4)), 66.7 (d, *J* 3.0,

C(3)H), 54.6 (d, *J* 6.9, PO*C*H_3), 53.6 (d, *J* 6.7, PO*C*H_3); **^{31}P{^{1}H} NMR** (162 MHz, $CDCl_3$) δ_P: 15.9 ppm; **HRMS** [ESI]$^+$ $C_{17}H_{16}ClO_5NaP$ [M+Na]$^+$ found 389.0321, requires 389.0318 (+0.87 ppm).

Dimethyl ((2*R*,3*R*)-3-(3-methoxyphenyl)-4-oxo-2-phenyloxetan-2-yl)phosphonate (*anti*-52) and dimethyl ((2*R*,3*S*)-3-(3-methoxyphenyl)-4-oxo-2-phenyloxetan-2-yl)phosphonate (*syn*-52)

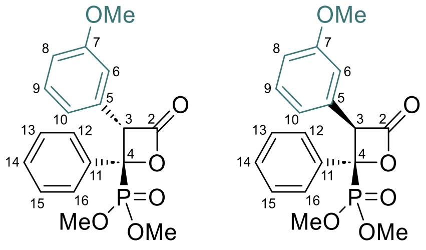

anti-**52** *syn*-**52**

Following **General Procedure D** with pivaloyl chloride (74 µL, 0.6 mmol), *i*-Pr_2NEt (105 µL, 0.6 mmol) and 3-methoxyphenylacetic acid (99.7 mg, 0.6 mmol) in MTBE (1.6 mL, 0.375 M). Then *i*-Pr_2NEt (35 µL, 0.2 mmol), (2*S*,3*R*)-HyperBTM (3.1 mg, 5 mol%), and dimethyl benzoylphosphonate **4** (35 µL, 0.2 mmol) in MTBE (0.4 mL, 0.5 M) at 25 °C for 16 h gave the crude product (86:14 $dr_{anti:syn}$). The crude mixture was purified by flash silica column chromatography [toluene : Et_2O (85:15 100 mL, 82.5:17.5 100 mL, 80:20 100 mL, 77.5:22.5 100 mL, 75:25 100 mL), R_f = 0.15 (*syn*-**52**) and 0.09 (*anti*-**52**)], then the solvents removed *in vacuo* to yield the combined *anti* and *syn*-diastereomers (85:15 $dr_{anti:syn}$) (43.1 mg, 58%) as an inseparable mixture and a yellow oil. $[\alpha]_D^{20}$ + 48.0 (*c* 1.0, $CHCl_3$); **IR** ν_{max} (film) 1838 (C=O), 1032 (P-O); **HRMS** [ESI]$^+$ $C_{18}H_{18}O_6NaP$ [M+Na]$^+$ found 385.0795, requires 385.0811 (−4.27 ppm).

Data for major diastereomer anti-**52: Chiral HPLC analysis** Chiralcel OD-H (95:5 hexane : IPA, flow rate 1.0 mLmin^{-1}, 211 nm, 40 ℃) t_R(2*S*,3*S*): 32.0 min, t_R(2*R*,3*R*): 36.7 min, 95:5 er_{anti}. **^{1}H NMR** (500 MHz, $CDCl_3$): δ_H: 7.22-7.15 (5H, m, C(12-16)*H*), 7.06 (1H, t, *J* 7.6, C(9)*H*), 6.72 (1H, dq, *J* 8.3, 0.8, C(8)*H*), 6.52 (1H, dt, *J* 7.6, 1.2, C(10)*H*), 6.35 (1H, m, C(6)*H*), 5.50 (1H, d, *J* 10.8, C(3)*H*), 3.93 (3H, d, *J* 10.4, POC*H_3*), 3.58 (3H, d, *J* 10.4, POC*H_3*), 3.58 (3H, s, OC*H_3*); **^{13}C{^{1}H} NMR** (126 MHz, $CDCl_3$) δ_C: 167.8 (d, *J* 7.4, *C*(2)), 159.5 (*C*(7)OCH_3), 131.0 (d, *J* 5.2, *C*(11)), 130.3 (d, *J* 3.3, *C*(5)), 129.6 (*C*(9)H), 128.5 (d, *J* 3.1, *C*(14)H), 128.0 (d, *J* 1.8, *C*(13,15)H), 126.7 (d, *J* 3.7, *C*(12,16)H), 122.0 (*C*(10)H), 114.9 (*C*(8)H), 114.6 (*C*(6)H, 80.4 (d, *J* 169.3, C(4)), 64.9 (d, *J* 1.1, *C*(3)H), 55.2 (d, *J* 7.4, PO*C*H_3), 55.2 (O*C*H_3), 54.6 (d, *J* 7.4, PO*C*H_3); **^{31}P{^{1}H} NMR** (202 MHz, $CDCl_3$) δ_P: 18.1 ppm.

Data for minor diastereomer syn-**52**: **Chiral HPLC analysis** Chiralcel OD-H (95:5 hexane : IPA, flow rate 1.0 mLmin^{-1}, 211 nm, 40 ℃) t_R(2*S*,3*R*): 49.0 min, t_R(2*R*,3*S*): 53.6 min, 82:18 er_{syn}. **^{1}H NMR** (500 MHz, $CDCl_3$): δ_H: 7.70-7.66 (2H, m, C(12,16)*H*), 7.50 (2H, t, *J* 7.9, C(13,15)*H*), 7.46-7.41 (1H, m, C(14)*H*), 7.36 (1H, t, *J* 7.9, C(9)*H*), 7.17-7.14 (2H, m, C(8,10)*H*), 6.92 (1H, dd, *J* 8.2, 2.5, C(6)*H*), 5.17 (1H, d, *J* 12.8, C(3)*H*), 3.86 (3H, s, OC*H_3*), 3.42 (3H, d, *J* 11.2, POC*H_3*), 3.39 (3H, d, *J* 10.8, POC*H_3*). **^{13}C{^{1}H} NMR** (126 MHz, $CDCl_3$) δ_C: 166.5 (d, *J* 6.0, *C*(2)), 159.6 (*C*(7)OCH_3), 136.2 (d, *J* 7.4, *C*(11)), 130.8 (*C*(5)),

129.6 (C(9)H), 129.0 (d, J 2.4, C(14)H), 128.8 (d, J 1.3, C(13,15)H), 125.7 (d, J 3.7, C(12,16)H), 121.0 (C(10)H), 114.4 (C(8)H), 114.3 (C(6)H), 67.2 (d, J 3.0, C(3)H), 55.4 (OCH$_3$), 54.5 (d, J 6.8, POCH$_3$), 53.6 (d, J 6.8, POCH$_3$); **^{31}P{^{1}H} NMR** (202 MHz, CDCl$_3$) δ_P: 15.8 ppm.

Dimethyl ((2*R*,3*R*)-3-(3,4-dimethoxyphenyl)-4-oxo-2-phenyloxetan-2-yl)phosphonate (*anti*-53) and dimethyl ((2*R*,3*S*)-3-(3,4-dimethoxyphenyl)-4-oxo-2-phenyloxetan-2-yl)phosphonate (*syn*-53)

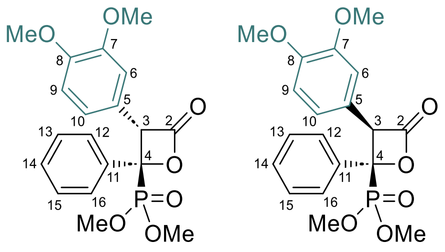

anti-**53** *syn*-**53**

Following **General Procedure D** with pivaloyl chloride (74 µL, 0.6 mmol), *i*-Pr$_2$NEt (105 µL, 0.6 mmol) and 2-(3,4-dimethoxyphenyl)acetic acid (117.7 mg, 0.6 mmol) in MTBE:MeCN (9:1 mixture, 1.6 mL, 0.375 M). Then *i*-Pr$_2$NEt (35 µL, 0.2 mmol), (2*S*,3*R*)-HyperBTM (3.1 mg, 5 mol%), and dimethyl benzoylphosphonate **4** (35 µL, 0.2 mmol) in MTBE (0.4 mL, 0.5 M) at 25 °C for 16 h gave the crude product (88:22 dr$_{anti:syn}$). The crude mixture was purified by flash silica column chromatography [toluene : CH$_2$Cl$_2$: EtOAc (3:7:0 40 mL, 1:9:0 40 mL, 1:8:1 40 mL, 1:7.5:1.5 40 mL, 1:7:2 40 mL, 1:6.5:2.5 40 mL, 1:6:3 40 mL, 1:4.5:4.5 40 mL, 0:0:10 40 mL), R_f = 0.32 (*anti*-**53**) and 0.26 (*syn*-**53**)], then the solvents removed *in vacuo* to yield the combined *anti* and *syn*-diastereomers (89:11 dr$_{anti:syn}$) (42.4 mg, 56%) as an inseparable mixture and a yellow oil. $[\alpha]_D^{20}$ + 4.2 (*c* 8.5, CHCl$_3$); **IR** ν_{max} (film) 1840 (C=O), 1028 (P-O); **HRMS** [ESI]$^+$ C$_{19}$H$_{21}$O$_7$NaP [M+Na]$^+$ found 415.0911, requires 415.0917 (−1.47 ppm).

Data for major diastereomer anti-**53: Chiral HPLC analysis** Chiralcel OD-H (95:5 hexane : IPA, flow rate 1.0 mLmin^{-1}, 220 nm, 40 ℃) t_R(2*S*,3*S*): 28.1 min, t_R(2*R*,3*R*): 33.4 min, 90:10 er$_{anti}$. **^{1}H NMR** (400 MHz, CDCl$_3$): δ_H: 7.24-7.15 (5H, m, C(12-16)H), 6.66 (1H, d, J 7.9, C(6)H), 6.57 (1H, dd, J 8.2, 2.0, C(10)H), 6.16 (1H, d, J 2.0, C(9)H), 5.57 (1H, d, J 11.0, C(3)H), 3.94 (3H, d, J 10.4, POCH_3), 3.79 (3H, s, C(8)OCH_3), 3.57 (3H, d, J 11.0, POCH_3), 3.54 (3H, s, C(7)OCH_3); **^{13}C{^{1}H} NMR** (101 MHz, CDCl$_3$) δ_C: 168.3 (d, J 9.2 C(2)), 149.3 (C(8)OCH$_3$), 148.7 (C(7)OCH$_3$), 131.3 (d, J 5.0, C(11)), 128.5 (d, J 2.6, C(14)H), 128.1 (d, J 1.9, C(13,15)H), 126.8 (d, J 4.1, C(12,16)H), 122.7 (C(10)H), 121.3 (d, J 3.1, C(11)H), 112.2 (C(9)H), 110.8 (C(6)H), 80.5 (d, J 168.9, C(4)), 64.7 (d, J 1.9, C(3)H), 55.8 (C(8)OCH$_3$), 55.7 (C(7)OCH$_3$), 55.2 (d, J 7.0, POCH$_3$), 54.6 (d, J 7.7, POCH$_3$); **^{31}P{^{1}H} NMR** (162 MHz, CDCl$_3$) δ_P: 18.1 ppm.

Data for minor diastereomer syn-**53**: **Chiral HPLC analysis** Chiralcel OD-H (95:5 hexane : IPA, flow rate 1.0 mLmin^{-1}, 220 nm, 40 ℃) t_R(2*S*,3*R*): 43.5 min, t_R(2*R*,3*S*): 51.5 min, 84:16 er$_{syn}$. **^{1}H NMR** (400 MHz, CDCl$_3$): δ_H: 7.68-7.64 (2H, m, C(12,16)H), 7.49 (2H, t, J 7.9, C(13,15)H), 7.46-7.40 (1H, m, C(14)H),

7.14-7.10 (2H, m, C(9,10)*H*), 6.92 (1H, d, *J* 7.9, C(6)*H*), 5.15 (1H, d, *J* 12.2, C(3)*H*), 3.96 (3H, s, C(8)OC*H*$_3$), 3.91 (3H, s, C(7)OC*H*$_3$), 3.45 (3H, d, *J* 11.0, POC*H*$_3$), 3.37 (3H, d, *J* 11.0, POC*H*$_3$); **^{13}C{^{1}H} NMR** (101 MHz, CDCl$_3$) δ_C: 164.3 (d, *J* 6.0, *C*(2)), 149.3 (*C*(8)OCH$_3$), 148.9 (*C*(7)OCH$_3$), 136.3 (d, *J* 5.1, *C*(11)), 129.0 (d, *J* 2.4, *C*(14)H), 128.8 (d, *J* 1.6, *C*(13,15)H), 125.6 (d, *J* 3.7, *C*(12,16)H), 121.7 (*C*(5)), 121.4 (*C*(10)H), 112.2 (*C*(9)H), 111.0 (*C*(6)H), 81.7 (d, *J* 172.6, *C*(4)), 67.3 (d, *J* 3.7, *C*(3)H), 56.1 (C(8)O*C*H$_3$), 55.9 (C(7)O*C*H$_3$), 54.4 (d, *J* 7.7, PO*C*H$_3$), 53.6 (d, *J* 7.7, PO*C*H$_3$); **^{31}P{^{1}H} NMR** (162 MHz, CDCl$_3$) δ_P: 16.1 ppm.

Dimethyl ((2*R*,3*R*)-4-oxo-2-phenyl-3-(3-(trifluoromethyl)phenyl)oxetan-2-yl)phosphonate (*anti*-54) and dimethyl ((2*R*,3*S*)-4-oxo-2-phenyl-3-(3-(trifluoromethyl)phenyl)oxetan-2-yl)phosphonate (*syn*-54)

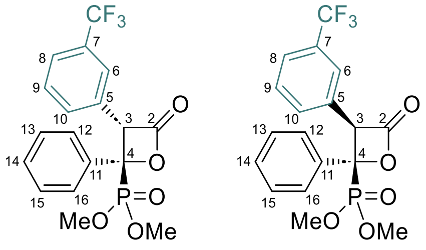

anti-**54** *syn*-**54**

Following **General Procedure D** with pivaloyl chloride (74 µL, 0.6 mmol), *i*-Pr$_2$NEt (105 µL, 0.6 mmol) and 3-(trifluoromethyl)phenylacetic acid (122.5 mg, 0.6 mmol) in MTBE (1.6 mL, 0.375 M). Then *i*-Pr$_2$NEt (35 µL, 0.2 mmol), (2*S*,3*R*)-HyperBTM (3.1 mg, 5 mol%), and dimethyl benzoylphosphonate **4** (35 µL, 0.2 mmol) in MTBE (0.4 mL, 0.5 M) at 25 °C for 16 h gave the crude product (77:23 dr$_{anti:syn}$). The crude mixture was purified by flash silica column chromatography [toluene : EtOAc (80:20 40 mL, 75:25 40 mL, 70:30 40 mL, 65:35 40 mL, 60:40 40 mL, 55:45 40 mL, 50:50 40 mL), R_f = 0.50 (*syn*-**55**) and 0.33 (*anti*-**55**)], then the solvents removed *in vacuo* to give *anti*-**54** (26.9 mg, 34%) as a yellow oil and *syn*-**55** (5.6 mg, 7%) as a yellow oil. *Note: both anti-**54** and syn-**54** were isolated, but epimerised over time, so the NMR spectra for each contain peaks corresponding to the other diastereomer, which have not been assigned for clarity.*

*Data for major diastereomer anti-***54**: $[\alpha]_D^{20}$ + 6.9 (*c* 5.6, CHCl$_3$); **Chiral HPLC analysis** Chiralpak AD-H (97:3 hexane : IPA, flow rate 1.0 mLmin^{-1}, 220 nm, 40 ℃) t_R(2*S*,3*S*): 16.6 min, t_R(2*R*,3*R*): 20.0 min, t_R(2*S*,3*R*), 86:14 er$_{anti}$; **IR** ν_{max} (film) 1842 (C=O), 1034 (P-O); **^{1}H NMR** (400 MHz, CDCl$_3$): δ_H: 8.16-8.11 (1H, m, Ar*H*), 7.55-7.43 (2H, m, Ar*H*), 7.29 (1H, dd, *J* 9.3, 7.9, Ar*H*), 7.24-7.14 (4H, m, Ar*H*), 7.12-7.07 (2H, m, Ar*H*), 5.63 (1H, d, *J* 10.8, C(3)*H*), 3.99 (3H, d, *J* 10.5, POC*H*$_3$), 3.61 (3H, d, *J* 10.5, POC*H*$_3$); **^{13}C{^{1}H} NMR** (101 MHz, CDCl$_3$) δ_C: 166.9 (d, *J* 3.3, *C*=O), 133.6 (Ar*C*), 132.8 (Ar*C*H), 130.6 (d, *J* 4.3, Ar*C*), 130.2 (Ar*C*H), 129.1 (Ar*C*H), 128.8 (d, *J* 2.6, Ar*C*H), 128.5 (Ar*C*H), 128.5 (Ar*C*H), 128.3 (d, *J* 2.0, Ar*C*H), 126.6 (d, *J* 4.1, Ar*C*H), 126.4 (q, *J* 3.5, *C*F$_3$), 125.5 (d, *J* 3.5, Ar*C*CF$_3$), 79.9 (dd, *J* 72.6, 3.1, *C*(4)), 64.5 (d, *J* 1.7, *C*(3)H), 55.4 (d, *J* 6.6, PO*C*H$_3$), 54.7 (d, *J* 7.8, PO*C*H$_3$); **^{31}P{^{1}H} NMR** (162 MHz, CDCl$_3$)

δ_P: 17.6 ppm; **^{19}F{^{1}H} NMR** (376 MHz, $CDCl_3$) δ_F: −63.0 ppm; **HRMS** [ESI]$^+$ $C_{18}H_{16}O_5F_3NaP$ [M+Na]$^+$ found 423.0574, requires 423.0580 (−1.34 ppm).

*Data for minor diastereomer syn-***54**: $[\alpha]_D^{20}$ – 0.2 (*c* 9.1, $CHCl_3$); **Chiral HPLC analysis** Chiralpak AD-H (97:3 hexane : IPA, flow rate 1.0 mLmin^{-1}, 220 nm, 40 ℃) t_R(2*S*,3*R*): 22.6 min, t_R(2*R*,3*S*): 24.9 min, 93:7 er$_{syn}$; **IR** ν_{max} (film) 1842 (C=O), 1034 (P-O); **^{1}H NMR** (400 MHz, $CDCl_3$): δ_H: 7.69-7.63 (2H, m, Ar*H*), 7.59-7.54 (2H, m, Ar*H*), 7.52-7.47 (2H, m, C(12,16)*H*), 7.47-7.42 (1H, m, C(14)*H*), 7.17-7.11 (2H, m, C(13,15)*H*), 5.16 (1H, dd, *J* 12.6, 1.0, C(3)*H*), 3.46 (3H, d, *J* 10.8, POC*H*$_3$), 3.38 (3H, d, *J* 10.6, POC*H*$_3$); **^{13}C{^{1}H} NMR** (101 MHz, $CDCl_3$) δ_C: 162.1 (*C*=O), 134.1 (Ar*C*H),133.9 (Ar*C*), 132.4 (Ar*C*H), 129.8 (d, *J* 4.8, Ar*C*H), 129.5 (Ar*C*), 129.3 (d, *J* 2.4, Ar*C*H), 129.1 (Ar*C*H), 129.0 (d, *J* 1.6, Ar*C*H), 127.7 (q, *J* 2.4, Ar*C*CF_3), 127.3 (q, *J* 4.8, *C*F_3), 125.7 (d, *J* 3.1, Ar*C*H), 66.7 (d, *J* 2.8, *C*(3)H), 54.6 (d, *J* 6.6, PO*C*H_3), 53.4 (d, *J* 7.2, PO*C*H_3); **^{31}P{^{1}H} NMR** (162 MHz, $CDCl_3$) δ_P: 15.2 ppm; **^{19}F{^{1}H} NMR** (376 MHz, $CDCl_3$) δ_F: −62.6 ppm; **HRMS** [ESI]$^+$ $C_{18}H_{16}O_5F_3NaP$ [M+Na]$^+$ found 423.0575, requires 423.0580 (−1.10 ppm).

Dimethyl ((2R,3R)-3-(benzo[b]thiophen-3-yl)-4-oxo-2-phenyloxetan-2-yl)phosphonate (*anti*-55) and dimethyl ((2R,3S)-3-(benzo[b]thiophen-3-yl)-4-oxo-2-phenyloxetan-2-yl)phosphonate (*syn*-55)

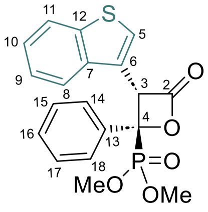

anti-**55**

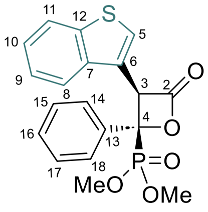

syn-**55**

Following **General Procedure D** with pivaloyl chloride (74 µL, 0.6 mmol), *i*-Pr_2NEt (105 µL, 0.6 mmol) and 2-(benzo[b]thiophen-3-yl)acetic acid (192.2 mg, 0.6 mmol) in MTBE (1.6 mL, 0.375 M). Then *i*-Pr_2NEt (35 µL, 0.2 mmol), (2*S*,3*R*)-HyperBTM (3.1 mg, 5 mol%), and dimethyl benzoylphosphonate **4** (35 µL, 0.2 mmol) in MTBE (0.4 mL, 0.5 M) at 25 °C for 16 h gave the crude product (84:16 dr$_{anti:syn}$). The crude mixture was purified by flash silica column chromatography [toluene : Et_2O (80:20 150 mL, 75:25 150 mL, 70:30 150 mL, 65:35 150 mL), R_f = 0.06 (*syn*-**55**) and 0.15 (*anti*-**55**)], then the solvents removed *in vacuo* to yield *anti*-**55** as a colourless oil (60.8 mg, 78%) and *syn*-**55** as a colourless oil (7.5 mg, 10%).

*Data for major diastereomer anti-***55:** $[\alpha]_D^{20}$ + 280.4 (*c* 0.24, $CHCl_3$); **Chiral HPLC analysis** Chiralpak AD-H (95:5 hexane : IPA, flow rate 1.0 mLmin^{-1}, 211 nm, 30 ℃) t_R(2*S*,3*S*): 18.1 min, t_R(2*R*,3*R*): 30.3 min, 98:2 er$_{anti}$; **IR** ν_{max} (film) 1834 (C=O), 1260 (P=O), 1059 (P-O), 1030 (P-O); **^{1}H NMR** ($CDCl_3$, 400 MHz) δ_H: 7.90 (1H, d, *J* 8.1, C(11)*H*), 7.77 (1H, d, *J* 8.1, C(8)*H*), 7.44 (1H, dd, *J* 7.4, 7.4, C(9)*H*), 7.36 (1H, dd, *J* 7.7, 7.7, C(10)*H*), 7.19-7.03 (5H, m, C(14-18)*H*), 6.97 (1H, s, C(5)*H*), 5.90 (1H, d, *J* 10.7, C(3)*H*), 3.99 (3H, d, *J* 10.1, POC*H*$_3$), 3.60 (3H, d, *J* 10.7, POC*H*$_3$); **^{13}C{^{1}H} NMR** (101 MHz, $CDCl_3$) δ_C: 167.7 (d, *J* 9.0, *C*(2)), 139.7 (*C*(12)), 137.5 (*C*(7)), 131.1 (d, *J* 5.3, *C*(13)), 128.6 (d, *J* 2.9, *C*(16)H), 128.0 (d, *J* 1.9,

C(15,17)H), 127.4 (*C*(5)H), 126.2 (d, *J* 3.7, *C*(14,18)H)), 124.8 (*C*(9)H), 124.8 (*C*(10)H), 123.7 (d, *J* 2.7, *C*(6)), 122.8 (*C*(8)H), 121.5 (*C*(11)H), 80.8 (d, *J* 170.0, *C*(4)), 58.5 (d, *J* 1.9, *C*(3)H), 55.3 (d, *J* 6.4, PO*C*H3), 54.7 (d, *J* 7.2, PO*C*H3); **^{31}P{^{1}H} NMR** (162 MHz, CDCl3) δ_P: 18.0 ppm; **HRMS** [ESI]$^+$ $C_{18}H_{19}O_5NaP$ [M+Na]$^+$ found 369.0851, requires 369.0862 (−3.07 ppm).

Data for minor diastereomer syn-**55**: $[\alpha]_D^{20}$ − 138.9 (*c* 0.29, CHCl3); **Chiral HPLC analysis** Chiralpak AS-H (97:3 hexane : IPA, flow rate 1.0 mLmin^{-1}, 211 nm, 40 ℃) t_R(2*S*,3*R*): 16.7 min, t_R(2*R*,3*S*): 21.6 min, 98:2 er$_{syn}$; **IR** ν_{max} (film) 1836 (C=O), 1263 (P=O), 1030 (P-O); **^{1}H NMR** (CDCl3, 400 MHz) δ_H: 7.91 (1H, dd, *J* 7.3, 1.4, C(11)*H*), 7.86 (1H, s, C(5)*H*), 7.74 (2H, dd, *J* 7.6, 1.7, C(15,17)*H*), 7.67 (1H, dd, *J* 7.2, 1.9, C(8)*H*), 7.57-7.39 (5H, m, C(9,10)*H* and C(14,16,18)*H*)), 5.35 (1H, d, *J* 12.5, C(3)*H*), 3.54 (3H, d, *J* 10.2, POC*H*3), 3.23 (3H, d, *J* 10.9, POC*H*3); **^{13}C{^{1}H} NMR** (101 MHz, CDCl3) δ_C: 167.1 (d, *J* 4.2, *C*(2)), 139.7 (*C*(12)), 139.4 (*C*(13)), 138.0 (*C*(7)), 129.2 (d, *J* 1.9, *C*(16)H)), 129.0 (*C*(5)H), 129.0 (d, *J* 1.4, *C*(14,18)H), 125.1 (d, *J* 3.6, *C*(15,17)H), 124.8 (*C*(9)H)), 124.6 (*C*(10)H), 123.1 (*C*(11)H), 121.8 (*C*(6)), 121.2 (*C*(8)H), 80.5 (d, *J* 194.6, *C*(4)), 62.6 (d, *J* 3.1, *C*(3)H), 54.2 (d, *J* 6.2, PO*C*H3), 53.6 (d, *J* 8.7, PO*C*H3); **^{31}P{^{1}H} NMR** (162 MHz, CDCl3) δ_P: 16.3 ppm.

Dimethyl ((2*R*,3*R*)-3-(benzofuran-3-yl)-4-oxo-2-phenyloxetan-2-yl)phosphonate (*anti*-56) and dimethyl ((2*R*,3*S*)-3-(benzofuran-3-yl)-4-oxo-2-phenyloxetan-2-yl)phosphonate (*syn*-56)

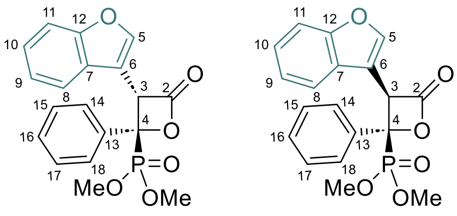

anti-**56** *syn*-**56**

Following **General Procedure D** with pivaloyl chloride (74 µL, 0.6 mmol), *i*-Pr2NEt (105 µL, 0.6 mmol) and 2-(benzofuran-3-yl)acetic acid (105.7 mg, 0.6 mmol) in MTBE (1.6 mL, 0.375 M). Then *i*-Pr2NEt (35 µL, 0.2 mmol), (2*S*,3*R*)-HyperBTM (3.1 mg, 5 mol%), and dimethyl benzoylphosphonate **4** (35 µL, 0.2 mmol) in MTBE (0.4 mL, 0.5 M) at 25 °C for 16 h gave the crude product (84:16 dr$_{anti:syn}$). The crude mixture was purified by flash silica column chromatography [toluene : Et2O (80:20 150 mL, 75:25 150 mL, 70:30 150 mL, 65:35 150 mL), R_f = 0.06 (*syn*-**56**) and 0.15 (*anti*-**56**)], then the solvents removed *in vacuo* to yield *anti*-**56** as a colourless oil (44.2 mg, 59%) and *syn*-**56** as a colourless oil (7.2 mg, 10%).

Data for major diastereomer anti-**56:** $[\alpha]_D^{20}$ + 137.6 (*c* 0.42, CHCl3); **Chiral HPLC analysis** Chiralcel OD-H (95:5 hexane : IPA, flow rate 1.0 mLmin^{-1}, 211 nm, 30 ℃) t_R(2*S*,3*S*): 20.1 min, t_R(2*R*,3*R*): 26.8 min, 90:10 er$_{anti}$; **IR** ν_{max} (film) 1840 (C=O), 1260 (P=O), 1059 (P-O), 1030 (P-O); **^{1}H NMR** (CDCl3, 400 MHz): 7.38 (1H, ddd, *J* 4.5, 1.2, 0.7, C(11)*H*), 7.36 (1H, ddd, *J* 4.5, 1.2, 0.7, C(10)*H*), 7.33 (1H, d, *J* 0.9, C(5)*H*), 7.30-7.27 (2H, m, C(14,18)*H*), 7.23 (1H, ddd, *J* 8.4, 7.1, 1.2, C(9)*H*), 7.17-7.10 (4H, m, C(8,15-17)*H*),

5.71 (1H, dd, *J* 10.4, 0.8, C(3)*H*), 3.97 (3H, d, *J* 10.5, POC*H*3), 3.60 (3H, d, *J* 10.5, POC*H*3); **^{13}C{^{1}H} NMR** (101 MHz, $CDCl_3$) δ_C: 167.1 (d, *J* 9.0, *C*(2)), 155.0 (*C*(12)), 144.4 (*C*(5)H), 131.1 (d, *J* 5.1, *C*(13)), 128.8 (d, *J* 2.8, *C*(16)H), 128.1 (d, *J* 2.5, *C*(15,17)H), 126.4 (d, *J* 4.0, *C*(14,18)H), 125.9 (*C*(7)), 124.9 (*C*(9)H), 123.1 (*C*(8)H), 119.6 (*C*(11)H), 111.6 (*C*(10)H), 109.8 (*C*(6)), 80.1 (d, *J* 169.3, *C*(4)), 55.3 (d, *J* 2.1, *C*(3)H), 55.3 (d, *J* 6.9, POCH3), 54.7 (d, *J* 7.6 POCH3); **^{31}P{^{1}H} NMR** (162 MHz, $CDCl_3$) δ_P: ppm; **HRMS** [ESI]$^+$ $C_{18}H_{19}O_5NaP$ [M+Na]$^+$ found 369.0851, requires 369.0862 (−3.07 ppm).

*Data for minor diastereomer syn-***56**: $[\alpha]_D^{20}$ − 47.9 (*c* 0.32, $CHCl_3$); **Chiral HPLC analysis** Chiralcel OD-H (95:5 hexane : IPA, flow rate 1.0 mLmin^{-1}, 211 nm, 30 ℃) t_R(2*R*,3*S*): 24.7 min, t_R(2*S*,3*R*): 31.3 min, 90:10 er$_{syn}$; **IR** ν_{max} (film) 1842 (C=O), 1030 (P-O); **^{1}H NMR** ($CDCl_3$, 400 MHz): 7.96 (1H, d, *J* 0.9, C(5)*H*), 7.70 (2H, ddd, *J* 7.9, 1.8, 1.8, C(14,18)*H*), 7.64 (1H, dd, *J* 6.6, 2.2, C(11)*H*), 7.54 (3H, m, C(10)*H* and C(15,17)*H*), 7.47 (1H, m, C(16)*H*), 7.35 (2H, m, C(8)*H* and C(9)*H*), 5.22 (1H, dd, *J* 12.4, 1.0, C(3)*H*), 3.54 (3H, d, *J* 10.8, POC*H*3), 3.34 (3H, d, *J* 10.6, POC*H*3); **^{13}C{^{1}H} NMR** (101 MHz, $CDCl_3$) δ_C: 166.5 (d, *J* 3.6, *C*(2)), 155.1 (*C*(12)), 145.5 (*C*(5)H), 135.9 (d, *J* 5.4, *C*(13)), 129.2 (d, *J* 2.4, *C*(16)H), 129.0 (d, *J* 2.0, *C*(15,17)H), 126.5 (*C*(7)), 125.4 (d, *J* 3.4, *C*(14,18)H), 125.0 (*C*(8)H), 123.3 (*C*(9)H), 119.5 (*C*(11)H), 111.9 (*C*(10)H), 109.6 (d, *J* 3.7, *C*(6)), 81.8 (d, *J* 173.2, *C*(4)), 58.9 (d, *J* 2.6, *C*(3)H), 54.5 (d, *J* 6.8, PO*C*H3), 53.7 (d, *J* 7.3, PO*C*H3); **^{31}P{^{1}H} NMR** (162 MHz, $CDCl_3$) δ_P: 16.0 ppm; **HRMS** [ESI]$^+$ $C_{18}H_{19}O_5NaP$ [M+Na]$^+$ found 369.0851, requires 369.0862 (−3.07 ppm).

Dimethyl ((2*R*,3*R*)-4-oxo-2-phenyl-3-(thiophen-3-yl)oxetan-2-yl)phosphonate (*anti*-57) and dimethyl ((2*R*,3*S*)-4-oxo-2-phenyl-3-(thiophen-3-yl)oxetan-2-yl)phosphonate (*syn*-57)

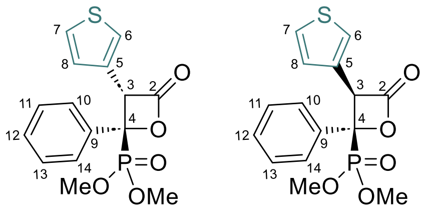

anti-**57** *syn*-**57**

Following **General Procedure D** with pivaloyl chloride (74 µL, 0.6 mmol), *i*-Pr_2NEt (105 µL, 0.6 mmol) and 2-(thiophen-3-yl)acetic acid (85.3 mg, 0.6 mmol) in MTBE (1.6 mL, 0.375 M). Then *i*-Pr_2NEt (35 µL, 0.2 mmol), (2*S*,3*R*)-HyperBTM (3.1 mg, 5 mol%), and dimethyl benzoylphosphonate **4** (35 µL, 0.2 mmol) in MTBE (0.4 mL, 0.5 M) at 25 °C for 16 h gave the crude product (84:16 dr$_{anti:syn}$). The crude mixture was purified by flash silica column chromatography [toluene : Et_2O (80:20 150 mL, 75:25 150 mL, 70:30 150 mL, 65:35 150 mL), R_f = 0.06 (*syn*-**57**) and 0.15 (*anti*-**57**)], then the solvents removed *in vacuo* to yield *anti*-**57** as a colourless oil (53.0 mg, 78%) and *syn*-**57** as a colourless oil (7.9 mg, 12%). $[\alpha]_D^{20}$ + 57.8 (*c* 1.00, $CHCl_3$); **IR** ν_{max} (film) 1842 (C=O), 1259 (P=O), 1059 (P-O), 1030 (P-O); **HRMS** [ESI]$^+$ $C_{18}H_{19}O_5NaP$ [M+Na]$^+$ found 369.0851, requires 369.0862 (−3.07 ppm).

*Data for major diastereomer anti-***57: Chiral HPLC analysis** Chiralcel OJ-H (97:3 hexane : IPA, flow rate 1.5 mLmin^{-1}, 211 nm, 30 ℃) t_R(2*S*,3*S*): 28.0 min, t_R(2*R*,3*R*): 33.1 min, 97:3 er$_{anti}$; **^{1}H NMR** ($CDCl_3$, 400 MHz): 7.25-7.18 (5H, m, C(10-14)*H*), 7.07 (1H, dd, *J* 5.0, 3.0, C(8)*H*), 6.98 (1H, ddd, *J* 3.0, 1.3, 0.6, C(6)*H*), 6.48 (1H, dd, *J* 5.0, 1.3, C(7)*H*), 5.62 (1H, d, *J* 10.6, C(3)*H*), 3.92 (3H, d, *J* 10.5, POCH_3), 3.57 (3H, d, *J* 10.5, POCH_3); **^{13}C{^{1}H} NMR** (101 MHz, $CDCl_3$) δ_C: 167.7 (d, *J* 8.4, *C*(2)), 131.2 (d, *J* 5.4, *C*(9)), 128.8 (d, *J* 3.8, *C*(5)), 128.6 (d, *J* 2.7, *C*(12)H), 128.1 (d, *J* 1.6, *C*(11,13)H), 127.5 (*C*(7)H), 126.6 (d, *J* 3.8, *C*(10,14)H), 126.3 (*C*(8)H), 126.0 (*C*(6)H), 80.1 (d, *J* 169.0, *C*(4)), 59.9 (d, *J* 1.9, *C*(3)H), 55.2 (d, *J* 7.3, PO*C*H_3), 54.6 (d, *J* 7.3, PO*C*H_3); **^{31}P{^{1}H} NMR** (162 MHz, $CDCl_3$) δ_P: 18.0 ppm.

*Data for minor diastereomer syn-***57**: **Chiral HPLC analysis** Chiralpak AD-H (95:5 hexane : IPA, flow rate 1.0 mLmin^{-1}, 211 nm, 30 ℃) t_R(2*S*,3*R*): 31.4 min, t_R(2*R*,3*S*): 41.6 min, 97:3 er$_{syn}$; **^{1}H NMR** ($CDCl_3$, 400 MHz): 7.65-7.60 (2H, m, C(10,14)*H*), 7.57 (1H, ddd, *J* 2.7, 1.2, 1.2, C(6)*H*), 7.48 (2H, ddd, *J* 7.4, 7.4, C(11,13)*H*), 7.43 (1H, dd, *J* 7.2, 1.5, C(12)*H*), 7.40 (1H, dd, *J* 5.0, 3.0, C(8)*H*), 7.30 (1H, dd, *J* 5.1, 1.3, C(7)*H*), 5.15 (1H, dd, *J* 12.7, 1.1, C(3)*H*), 3.44 (3H, d, *J* 10.8, POCH_3), 3.41 (3H, d, *J* 10.6, POCH_3); **^{13}C{^{1}H} NMR** (101 MHz, $CDCl_3$) δ_C: 166.8 (d, *J* 6.1, *C*(2), 135.9 (d, *J* 6.0, *C*(9)), 129.1 (d, *J* 2.7, *C*(8)H), 129.0 (d, *J* 2.7, *C*(12)H), 128.9 (d, *J* 2.7, *C*(11,13)H), 128.2 (*C*(5), 127.8 (*C*(7)H), 125.7 (d, *J* 3.5, *C*(10,14)H), 124.9 (*C*(6)H), 81.4 (d, *J* 146.0, *C*(4)), 63.2 (d, *J* 2.7, *C*(3)H), 54.5 (d, *J* 6.5, PO*C*H_3), 53.6 (d, *J* 6.5, PO*C*H_3); **^{31}P{^{1}H} NMR** (162 MHz, $CDCl_3$) δ_P: 15.8 ppm.

Dimethyl ((2*R*,3*R*)-4-oxo-3-(p-tolyl)-2-(4-(trifluoromethyl)phenyl)oxetan-2-yl)phosphonate (*anti*-58) and dimethyl ((2*R*,3*S*)-4-oxo-3-(p-tolyl)-2-(4-(trifluoromethyl)phenyl)oxetan-2-yl)phosphonate (*syn*-58)

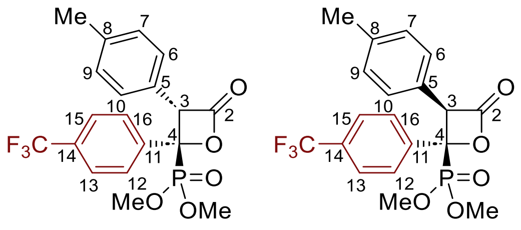

anti-**58** *syn*-**58**

Following **General Procedure D** with pivaloyl chloride (74 μL, 0.6 mmol), *i*-Pr_2NEt (105 μL, 0.6 mmol) and 4-tolylacetic acid (90.1 mg, 0.6 mmol) in MTBE (1.6 mL, 0.375 M). Then *i*-Pr_2NEt (35 μL, 0.2 mmol), (2*S*,3*R*)-HyperBTM (3.1 mg, 5 mol%), and dimethyl (4-(trifluoromethyl)benzoyl)phosphonate **S17** (56.4 mg, 0.2 mmol) in MTBE (0.4 mL, 0.5 M) at 25 °C for 16 h gave the crude product (88:12 dr$_{anti:syn}$). The crude mixture was purified by flash silica column chromatography [CH_2Cl_2 : EtOAc (95:5 200 mL, 90:10 200 mL, 85:15 200 mL,), R_f = 0.42 (*anti*-**58**) and 0.70 (*syn*-**58**)], then the solvents removed *in vacuo* to yield *anti*-**58** as a colourless oil (32.8 mg, 40%) and *syn*-**58** as a colourless oil (4.5 mg, 5%).

*Data for major diastereomer anti-***58**: $[\alpha]_D^{20}$ + 96.5 (*c* 0.29, $CHCl_3$); **Chiral HPLC analysis** Chiralpak AD-H (95:5 hexane : IPA, flow rate 1.0 mLmin^{-1}, 211 nm, 30 ℃) t_R(2*S*,3*S*): 17.1 min, t_R(2*R*,3*R*): 19.6 min, 99:1 er$_{anti}$; **IR** ν_{max} (film) 1846 (C=O), 1325 (P=O), 1063 (P-O); **^{1}H NMR** ($CDCl_3$, 400 MHz): 7.47 (2H, d, *J* 8.4, C(13,15)*H*), 7.30 (2H, dd, *J* 8.6, 1.9, C(12,16)*H*), 6.98 (2H, d, *J* 7.6, C(7,9)*H*), 6.78 (2H, d, *J* 7.4, C(6,10)*H*), 5.57 (1H, d, *J* 10.7, C(3)*H*), 3.97 (3H, d, *J* 11.6, POC*H*$_3$), 3.67 (3H, d, *J* 10.5,POC*H*$_3$), 2.26 (3H, s, C*H*$_3$); **^{13}C{^{1}H} NMR** (101 MHz, $CDCl_3$) δ_C: 167.5 (d, *J* 8.72, *C*(2)), 139.1 (*C*(8)CH$_3$), 135.5 (d, *J* 5.0, *C*(11), 130.5 (dd, *J* 32.4, 2.5, *C*(14)), 129.5 (*C*(7,9)H), 129.3 (*C*(6,10)H), 127.3 (d, *J* 3.4, *C*(12,16)H), 125.4 (d, *J* 3.2, *C*(5)), 125.1 (*C*F$_3$)), 124.9 (dd, *J* 4.1, 2.3, *C*(13,15)H), 80.1 (d, *J* 169.0, *C*(4)), 65.0 (*C*(3)H), 55.3 (d, *J* 6.9, PO*C*H$_3$), 54.7 (d, *J* 7.5, PO*C*H$_3$), 21.2 (*C*H$_3$); **^{19}F{^{1}H} NMR** (MHz, $CDCl_3$) δ_F: −62.8 (d, *J* 1.6) ppm; **^{31}P{^{1}H} NMR** (162 MHz, $CDCl_3$) δ_P: 17.6 (d, *J* 1.8) ppm; **HRMS** [ESI]$^+$ $C_{19}H_{18}O_5F_3NaP$ [M+Na]$^+$ found 437.0739, requires 437.0736 (0.45 ppm).

*Data for minor diastereomer syn-***58**: $[\alpha]_D^{20}$ − 2.2 (*c* 0.47, $CHCl_3$); **Chiral HPLC analysis** Chiralpak AD-H (95:5 hexane : IPA, flow rate 1.0 mLmin^{-1}, 211 nm, 30 ℃) t_R(2*S*,3*S*): 17.9 min, t_R(2*R*,3*R*): 28.6 min, 98:2 er$_{anti}$; **IR** ν_{max} (film) 1846 (C=O), 1325 (P=O), 1069 (P-O), 1032 (P-O); **^{1}H NMR** ($CDCl_3$, 400 MHz): 7.82 (2H, dd, *J* 8.5, 1.8, C(12,16)*H*), 7.76 (2H, d, *J* 8.4, C(13,15)*H*), 7.43 (2H, d, *J* 8.0, C(7,9)*H*), 7.26 (d, *J* 8.0, C(6,10)*H*), 5.14 (1H, d, *J* 12.7, C(3)*H*), 3.43 (3H, d, *J* 5.3, POC*H*$_3$), 3.41 (3H, d, *J* 5.2, POC*H*$_3$), 2.38 (3H, s, C*H*$_3$); **^{13}C{^{1}H} NMR** (101 MHz, $CDCl_3$) δ_C: 166.3 (d, *J* 3.7, *C*(2)), 138.9 (*C*(8)CH$_3$), 129.4 (*C*(6,10)H), 128.7 (*C*(7,9)H), 126.2 (d, *J* 3.6, *C*(12,16)H), 125.8 (dd, *J* 7.1, 2.6, *C*(13,15)H), 80.8 (d, *J* 254.9, *C*(4)), 67.5 (d, *J* 2.4, *C*(3)), 54.4 (d, *J* 7.3, PO*C*H$_3$), 53.7 (d, *J* 6.8, PO*C*H$_3$), 21.3 (*C*H$_3$). Note: aromatic quaternary carbons unable to be assigned; **^{19}F{^{1}H} NMR** (MHz, $CDCl_3$) δ_F: −62.8 (d, *J* 1.7) ppm; **^{31}P{^{1}H} NMR** (162 MHz, $CDCl_3$) δ_P: 15.1 (d, *J* 1.3) ppm; **HRMS** [ESI]$^+$ $C_{19}H_{18}O_5F_3NaP$ [M+Na]$^+$ found 437.0736, requires 437.0736 (0.03 ppm).

Dimethyl ((2*R*,3*R*)-2-(4-chlorophenyl)-4-oxo-3-(p-tolyl)oxetan-2-yl)phosphonate (*anti*-59)

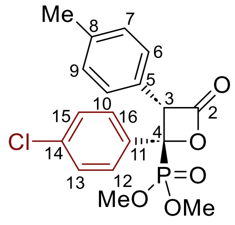

anti-**59**

Following General Procedure D with pivaloyl chloride (74 µL, 0.6 mmol), *i*-Pr_2NEt (105 µL, 0.6 mmol) and *p*-tolylacetic acid (90.1 mg, 0.6 mmol) in MTBE (1.6 mL, 0.375 M). Then *i*-Pr_2NEt (35 µL, 0.2 mmol), (2*S*,3*R*)-HyperBTM (3.1 mg, 5 mol%), and dimethyl (4-chlorobenzoyl)phosphonate **S18** (49.7 mg, 0.2 mmol) in MTBE (0.4 mL, 0.5 M) at 25 °C for 16 h gave the crude product (>95:5 dr$_{anti:syn}$). The crude mixture was purified by flash silica column chromatography [hexane : EtOAc (70:30 150 mL, 60:40 200 mL, 55:45 100 mL, 50:50 100 mL, 45:55 100 mL, 40:60 100 mL), Rf = 0.28 (*anti*-**59**), then the solvents

removed *in vacuo* to yield the *anti*-diastereomer (74.7 mg, 98%) as a colourless oil. $[\boldsymbol{\alpha}]_{\boldsymbol{D}}^{\mathbf{20}}$ + 78.7 (*c* 83, $CHCl_3$); **Chiral HPLC Data** Chiralpak AD-H (95:5 hexane : IPA, flow rate 1.0 mLmin^{-1}, 220 nm, 30 ℃) t_R(2*S*,3*S*): 17.5 min, t_R(2*R*,3*R*): 22.5 min, 98:2 er$_{anti}$; **IR** ν_{max} (film) 1846 (C=O), 1261 (P=O), 1061 (P-O), 1036 (P-O); **^{1}H NMR** (400 MHz, $CDCl_3$): δ_H: 7.16 (2H, d, *J* 8.5, C(13,15)*H*), 7.08 (2H, d, *J* 8.9, C12,16)*H*), 6.97 (2H, d, *J* 7.5, C(7,9)*H*), 6.76 (2H, d, *J* 6.8, C(6,10)*H*), 5.50 (1H, d, *J* 10.8, C(3)*H*), 3.92 (3H, d, *J* 10.5, POC*H*$_3$), 3.62 (3H, d, *J* 10.5, POC*H*$_3$), 2.24 (3H, s, ArC*H*$_3$); **^{13}C{^{1}H} NMR** (101 MHz, $CDCl_3$): δ_C: 167.7 (d, *J* 8.8, *C*(2)), 138.9 (*C*(8)CH_3)), 134.7 (d, *J* 3.7, *C*(11)), 129.9 (d, *J* 5.6, *C*(14)Cl), 129.5 (*C*(6,10)H), 129.4 (*C*(7,9)H), 128.3 (*C*13,15)H), 128.3 (d, *J* 5.7, *C*(12,16)H), 125.6 (d, *J* 3.7, *C*(5))), 80.0 (d, *J* 169.7, *C*(4)), 64.7 (d, *J* 1.2, *C*(3)H), 55.2 (d, *J* 6.8, PO*C*H$_3$), 54.6 (d, *J* 7.7, PO*C*H$_3$), 21.2 (Ar*C*H$_3$); **^{31}P{^{1}H} NMR** (162 MHz, $CDCl_3$): δ_P: 17.9 ppm; **HRMS** [ESI]$^+$ $C_{18}H_{19}ClO_5P$ [M+H]$^+$ found 381.0639, requires 381.0653 (−3.70 ppm).

Dimethyl ((2*R*,3*R*)-2-(4-fluorophenyl)-4-oxo-3-(p-tolyl)oxetan-2-yl)phosphonate (*anti*-60)

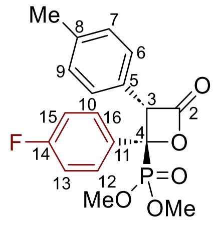

anti-**60**

Following **General Procedure D** with pivaloyl chloride (74 µL, 0.6 mmol), *i*-Pr_2NEt (105 µL, 0.6 mmol) and 4-tolylacetic acid (90.1 mg, 0.6 mmol) in MTBE (1.6 mL, 0.375 M). Then *i*-Pr_2NEt (35 µL, 0.2 mmol), (2*S*,3*R*)-HyperBTM (3.1 mg, 5 mol%), and dimethyl (4-fluorobenzoyl)phosphonate **S20** (46.4 mg, 0.2 mmol) in MTBE (0.4 mL, 0.5 M) at 25 °C for 16 h gave the crude product (>95:5 dr$_{anti:syn}$). The crude mixture was purified by flash silica column chromatography [hexane : EtOAc (60:40 100 mL, 55:45 100 mL, 50:50 100 mL, 45:55 100 mL, 40:60 100 mL, 35:65 100 mL), R_f = 0.29, then the solvents removed *in vacuo* to yield *anti*-**60** as a single diastereomer and a colourless oil (58.3 mg, 80%). $[\alpha]_D^{20}$ + 11.1 (*c* 0.35, $CHCl_3$); **Chiral HPLC analysis** Chiralcel OD-H (95:5 hexane : IPA, flow rate 1.0 mLmin^{-1}, 211 nm, 30 ℃) t_R(2*S*,3*S*): 15.4 min, t_R(2*R*,3*R*): 20.3 min, 98:2 er$_{anti}$; **IR** ν_{max} (film) 1838 (C=O), 1260 (P=O), 1059 (P-O), 1032 (P-O); **^{1}H NMR** ($CDCl_3$, 400 MHz): 7.13 (2H, ddd, *J* 9.0, 5.2, 2.2, C(12,16)*H*)), 6.96 (2H, d, *J* 7.7, C(6,10)*H*), C(6,10)*H*), 6.88 (2H, ddd, *J* 9.1, 8.5, 0.8, C(13,15)*H*), 6.76 (2H, ddd, *J* 7.7, 2.2, 1.8, C(7,9)*H*), 5.49 (1H, d, *J* 10.8, C(3)*H*), 3.93 (3H, d, *J* 10.6, POC*H*$_3$), 3.61 (3H, d, *J* 10.5, POC*H*$_3$), 2.24 (3H, s, ArC*H*$_3$); **^{13}C{^{1}H} NMR** (101 MHz, $CDCl_3$) δ_C: 167.9 (d, *J* 8.8, *C*(2)), 162.6 (dd, *J* 248.6, 2.7, *C*(14)F), 138.8 (*C*(8)CH_3)), 129.4 (*C*(6,7,9,10)H)), 128.8 (dd, *J* 8.4, 4.0, *C*(12,16)H), 127.1 (dd, *J* 5.3, 3.1, *C*(11)), 125.8 (d, *J* 3.2, *C*(5)), 115.2 (dd, *J* 21.8, 2.3, *C*(13,15)H), 80.0 (d, *J* 170.2, *C*(4)), 64.7 (d, *J* 1.2, *C*(3)H), 55.2 (d, *J* 6.9, PO*C*H$_3$), 54.6 (d, *J* 7.6, PO*C*H$_3$), 21.2 (*C*H$_3$); **^{19}F{^{1}H} NMR** (376 MHz, $CDCl_3$) δ_F: −112.8

(d, J 4.3); **^{31}P{^{1}H} NMR** (162 MHz, $CDCl_3$) δ_P: 18.1 (d, J 4.0) ppm; **HRMS** [ESI]$^+$ $C_{18}H_{18}O_5FNaP$ [M+Na]$^+$ found 387.0771, requires 387.0768 (+0.79 ppm).

Dimethyl ((2R,3R)-2-(4-chlorophenyl)-4-oxo-3-(thiophen-3-yl)oxetan-2-yl)phosphonate (*anti*-61) and dimethyl ((2R,3S)-2-(4-chlorophenyl)-4-oxo-3-(thiophen-3-yl)oxetan-2-yl)phosphonate (*syn*-61)

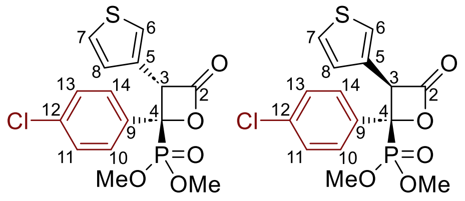

anti-**61** *syn*-**61**

Following **General Procedure D** with pivaloyl chloride (74 µL, 0.6 mmol), *i*-Pr_2NEt (105 µL, 0.6 mmol) and 3-thiophenylacetic acid (85.3 mg, 0.6 mmol) in MTBE (1.6 mL, 0.375 M). Then *i*-Pr_2NEt (35 µL, 0.2 mmol), (2*S*,3*R*)-HyperBTM (3.1 mg, 5 mol%), and dimethyl (4-chlorobenzoyl)phosphonate **S18** (49.7 mg, 0.2 mmol) in MTBE (0.4 mL, 0.5 M) at 25 °C for 16 h gave the crude product (86:14 $dr_{anti:syn}$). The crude mixture was purified by flash silica column chromatography [hexane : EtOAc (70:30 (100 mL), 60:40 (200 mL), 55:45 (200 mL), 50:50 (200 mL)), R_f = 0.35 (*syn*-**61**)and 0.26 (*anti*-**61**)], then the solvents removed *in vacuo* to yield *anti*-**61** as a colourless oil (61.9 mg, 83%) and *syn*-**61** as a colourless oil (10.4 mg, 14%).

Data for major diastereomer anti-**61**: $[\alpha]_D^{20}$ + 60.9 (*c* 1.22, $CHCl_3$); **Chiral HPLC analysis** Chiralpak AD-H (95:5 hexane : IPA, flow rate 1.0 mLmin^{-1}, 220 nm, 30 ℃) t_R(2*S*,3*S*): 18.8 min, t_R(2*R*,3*R*): 23.3 min, 94:6 er_{anti}; **IR** ν_{max} (film) 1844 (C=O), 1261 (P=O), 1059 (P-O), 1034 (P-O); **^{1}H NMR** ($CDCl_3$, 400 MHz): 7.26-7.21 (2H, m, C(11,13)*H*), 7.20-7.12 (3H, m, C(8)*H* and C(10,14)*H*), 7.03 (1H, ddd, J 2.9, 1.3, 0.6, C(7)*H*), 6.52 (1H, dd, J 5.0, 1.4, C(6)*H*), 5.64 (1H, d, J 10.6, C(3)*H*), 3.95 (3H, d, J 10.6, POCH_3), 3.65 (3H, d, J 10.5, POCH_3); **^{13}C{^{1}H} NMR** (101 MHz, $CDCl_3$) δ_C: 167.3 (d, J 8.7, *C*(2)), 134.9 (d, J 2.9, *C*(5)), 129.9 (d, J 5.1, *C*(9)H), 128.5 (d, J 3.3, *C*(12)H), 128.4 (d, J 2.4, *C*(11,13)H), 128.0 (d, J 3.9, *C*(10,14)H), 127.3 (*C*(6)H), 126.8 (*C*(7)H), 126.1 (*C*(8)H), 79.7 (d, J 170.0, *C*(4)), 60.0 (d, J 1.8, *C*(3)H), 55.3 (d, J 6.9, PO*C*H_3), 54.7 (PO*C*H_3); **^{31}P{^{1}H} NMR** (162 MHz, $CDCl_3$) δ_P: 17.6 ppm; **HRMS** [ESI]$^+$ $C_{15}H_{15}O_5ClPS$ [M+H]$^+$ found 373.0060, requires 373.0061 (−0.21 ppm).

Data for minor diastereomer syn-**61**: $[\alpha]_D^{20}$ + 13.8 (*c* 0.08, $CHCl_3$); **Chiral HPLC analysis** Chiralpak IA (95:5 hexane : IPA, flow rate 1.0 mLmin^{-1}, 220 nm, 30 ℃) t_R(2*R*,3*S*): 31.5 min, t_R(2*S*,3*R*): 47.1 min, 94:6 er_{syn}; **IR** ν_{max} (film) 1838 (C=O), 1263 (P=O), 1030 (P-O), 1016 (P-O); **^{1}H NMR** ($CDCl_3$, 500 MHz): 7.61-7.53 (3H, m, C(6)*H* and C(10,14)*H*), 7.47 (2H, d, J 8.7, C(11,13)*H*), 7.41 (1H, dd, J 5.0, 2.9, C(8)*H*), 7.27 (1H, dd, J 5.0, 1.4, C(7)*H*), 5.11 (1H, d, J 12.6, C(3)*H*), 3.48 (3H, d, J 8.6, POCH_3), 3.47 (3H, d, J 8.6, POCH_3); **^{13}C{^{1}H} NMR** (126 MHz, $CDCl_3$) δ_C: 166.4 (d, J 4.1, *C*(2)), 135.3 (d, J 3.2, *C*(12)), 134.5 (d, J

5.0, *C*(8)), 129.1 (d, *J* 2.3, *C*(11,13)H), 128.8 (d, *J* 2.7, *C*(5)H), 127.7 (*C*(7)H), 127.1 (d, *J* 3.6, *C*(10,14)H), 126.2 (*C*(8)H), 125.0 (*C*(6)H); **^{31}P{^{1}H} NMR** (202 MHz, $CDCl_3$) δ_P: 15.4 ppm; **HRMS** [ESI]$^+$ $C_{15}H_{15}O_5ClPS$ [M+H]$^+$ found 373.0047, requires 373.0061 (−3.70 ppm).

Dimethyl ((2*R*,3*R*)-2-(4-fluorophenyl)-4-oxo-3-(thiophen-3-yl)oxetan-2-yl)phosphonate (*anti*-62) and dimethyl ((2*R*,3*S*)-2-(4-fluorophenyl)-4-oxo-3-(thiophen-3-yl)oxetan-2-yl)phosphonate (*syn*-62)

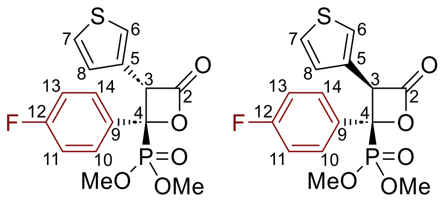

anti-**62** *syn*-**62**

Following **General Procedure D** with pivaloyl chloride (74 µL, 0.6 mmol), *i*-Pr_2NEt (105 µL, 0.6 mmol) and 3-thiophenylacetic acid (85.3 mg, 0.6 mmol) in MTBE (1.6 mL, 0.375 M). Then *i*-Pr_2NEt (35 µL, 0.2 mmol), (2*S*,3*R*)-HyperBTM (3.1 mg, 5 mol%), and dimethyl (4-fluorobenzoyl)phosphonate **S20** (46.4 mg, 0.2 mmol) in MTBE (0.4 mL, 0.5 M) at 25 °C for 16 h gave the crude product (87:13 $dr_{anti:syn}$). The crude mixture was purified by flash silica column chromatography [hexane : EtOAc (70:30 200 mL, 60:40 200 mL, 50:50 200 mL, 40:60 200 mL), R_f = 0.24 (*syn*-**62**) and 015 (*anti*-**62**)], then the solvents removed *in vacuo* to yield *anti*-**62** as a colourless oil (60.0 mg, 86%, 88:12 $dr_{anti:syn}$) and *syn*-**62** as a colourless oil (8.4 mg, 12%).

Data for major diastereomer anti-**62**: $[\alpha]_D^{20}$ + 4.4 (*c* 0.59, $CHCl_3$); **Chiral HPLC analysis** Chiralpak AS-H (95:5 hexane : IPA, flow rate 1.0 mLmin^{-1}, 211 nm, 30 ℃) t_R(2*S*,3*S*): 18.3 min, t_R(2*R*,3*R*): 20.7 min, 97:3 er_{anti}; **IR** ν_{max} (film) 1842 (C=O), 1259 (P=O), 1057 (P-O), 1032 (P-O); **^{1}H NMR** ($CDCl_3$, 400 MHz): δ_H: 7.19 (2H, ddd, *J* 9.1, 5.2, 2.3, C(10,14)*H*), 7.12 (1H, dd, *J* 5.0, 2.9, C(8)*H*), 7.00 (1H, dd, *J* 2.9, 0.8, C(7)*H*), 6.93 (2H, dd, *J* 8.4, 8.4, C(11,13)*H*), 6.49 (1H, dd, *J* 5.0, 1.3, C(6)*H*), 5.60 (1H, d, *J* 10.5, C(3)*H*), 3.93 (3H, d, *J* 10.5, POC*H*$_3$), 3.61 (3H, d, *J* 10.5, POC*H*$_3$); **^{13}C{^{1}H} NMR** (101 MHz, $CDCl_3$) δ_C: 167.2 (d, *J* 8.7, *C*(2)), 163.2 (d, *J* 7.7, *C*(12)F), 133.2 (*C*(5)), 128.6 (*C*(11,13)H), 127.4 (*C*(6)H), 126.6 (*C*(9)H), 125.9 (*C*(7)H)), 123.1 (*C*(8)H), 115.3 (dd, *J* 23.2, 8.4, *C*(10,14)H), 79.6 (d, *J* 147.9, *C*(4)), 60.0 (d, *J* 4.2, *C*(3)H), 55.3 (d, *J* 7.2, PO*C*H$_3$), 54.6 (d, *J* 6.7, PO*C*H$_3$); **^{19}F{^{1}H} NMR** (376 MHz, $CDCl_3$) δ_F: −112.4 (d, *J* 4.4); **^{31}P{^{1}H} NMR** (162 MHz, $CDCl_3$) δ_P: 17.8 (d, *J* 3.8); **HRMS** [ESI]$^+$ $C_{15}H_{15}FO_5PS$ [M+H]$^+$ found 357.0346, requires 357.0356 (− 2.91 ppm).

Data for minor diastereomer syn-**62**: $[\alpha]_D^{20}$ − 23.0 (*c* 0.56, $CHCl_3$); **Chiral HPLC analysis** Chiralpak AD-H (94:6 hexane : IPA, flow rate 1.0 mLmin^{-1}, 220 nm, 30 ℃) t_R(2*S*,3*R*): 24.5 min, t_R(2*R*,3*S*): 41.2 min, 96:3 er_{syn}; **IR** ν_{max} (film) 1838 (C=O), 1261 (P=O), 1028 (P-O); **^{1}H NMR** ($CDCl_3$, 400 MHz): 7.62 (2H, ddd, *J* 9.0, 5.1, 2.1, C(10,14)*H*), 7.56 (1H, dd, *J* 2.6, 1.2, C(6)*H*), 7.41 (1H, dd, *J* 5.0, 3.0, C(8)*H*), 7.28 (1H,

dd, *J* 5.0, 1.5, C(7)*H*), 7.18 (2H, dd, *J* 8.4, 8.4, C(11,13)*H*), 5.12 (1H, dd, *J* 12.5, 1.3, C(3)*H*), 3.45 (3H, d, *J* 10.7, POC*H*3), 3.44 (3H, d, *J* 10.8, POC*H*3); **^{13}C{^{1}H} NMR** (101 MHz, $CDCl_3$) δ_C: 166.5 (d, *J* 4.4, *C*(2)), 163.0 (dd, *J* 2.49.0, 2.3, *C*(12)F), 131.8 (dd, *J* 4.9, 3.5, *C*(9)), 128.9 (d, *J* 2.1, *C*(5)), 127.7 (dd, *J* 8.7, 3.3, *C*(10,14)H), 127.7 (*C*(7)H), 126.2 (*C*(8)H), 124.9 (*C*(6)H), 116.0 (dd, *J* 22.2, 1.8, *C*(11,13)H), 80.8 (d, *J* 172.9, *C*(4)), 63.3 (d, *J* 3.3, *C*(4)H), 54.5 (d, *J* 7.0, PO*C*H3), 53.6 (d, *J* 7.2, PO*C*H3); **^{19}F{^{1}H} NMR** (376 MHz, $CDCl_3$) δ_F: −111.6 ppm; **^{31}P{^{1}H} NMR** (162 MHz, $CDCl_3$) δ_P: 15.6 ppm; **HRMS** [ESI]$^+$ $C_{15}H_{14}FO_5NaPS$ [M+Na]$^+$ found 379.0176, requires 379.0176 (+ 0.03 ppm).

Dimethyl ((2*R*,3*R*)-4-oxo-3-(p-tolyl)-2-(3-(trifluoromethyl)phenyl)oxetan-2-yl)phosphonate (*anti*-63) and dimethyl ((2*R*,3*R*)-4-oxo-3-(p-tolyl)-2-(3-(trifluoromethyl)phenyl)oxetan-2-yl)phosphonate (*syn*-63)

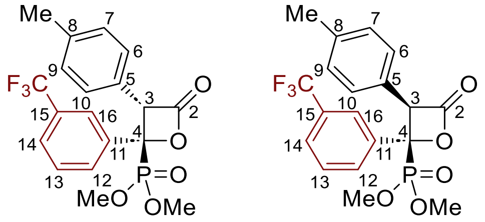

anti-**63** *syn*-**63**

Following **General Procedure D** with pivaloyl chloride (74 µL, 0.6 mmol), *i*-Pr_2NEt (105 µL, 0.6 mmol) and 4-tolylacetic acid (90.1 mg, 0.6 mmol) in MTBE (1.6 mL, 0.375 M). Then *i*-Pr_2NEt (35 µL, 0.2 mmol), (2*S*,3*R*)-HyperBTM (3.1 mg, 5 mol%), and dimethyl (3-(trifluoromethyl)benzoyl)phosphonate **S31** (56.4 mg, 0.2 mmol) in MTBE (0.4 mL, 0.5 M) at 25 °C for 16 h gave the crude product (90:10 $dr_{anti:syn}$). The crude mixture was purified by flash silica column chromatography [CH_2Cl_2 : EtOAc (100:0 150 mL, 95:5 100 mL, 90:10 100 mL, 85:15 100 mL), R_f = 0.48 (*syn*-**XX**) and 0.60 (*anti*-**63**)], then the solvents removed *in vacuo* to yield *anti*-**63** as a colourless oil (41.4 mg, 50%) and *syn*-**63** as a colourless oil (4.1 mg, 5%). Unfortunately, due to difficulties purifying these compounds, there are minor impurities in the NMR spectra.

Data for major diastereomer anti-**63**: $[\alpha]_D^{20}$ + 75.9 (*c* 1.44, $CHCl_3$); **Chiral HPLC analysis** Chiralpak IA (97:3 hexane : IPA, flow rate 1.0 mLmin^{-1}, 211 nm, 30 ℃) t_R(2*R*,3*R*): 17.1 min, t_R(2*S*,3*S*): 18.6 min, 99:1 er_{anti}; **IR** ν_{max} (film) 1846 (C=O), 1333 (P=O), 1061 (P-O), 1036 (P-O); **^{1}H NMR** ($CDCl_3$, 400 MHz): 7.39 (1H, d, *J* 6.9, C(16)*H*), 7.26-7.21 (2H, m, C(14,15)*H*), 7.09 (1H, dd, *J* 8.0, 7.8, C(12)*H*), 6.89 (2H, d, *J* 7.7, C(7,9)*H*), 6.68 (2H, d, *J* 7.4, C(6,10)*H*), 5.50 (1H, d, *J* 11.1, C(3)*H*), 3.87 (3H, d, *J* 10.6, POC*H*3), 3.60 (3H, d, *J* 10.6, POC*H*3), 2.16 (3H, s, C*H*3); **^{13}C{^{1}H} NMR** (101 MHz, $CDCl_3$) δ_C: 167.4 (d, *J* 8.8, *C*(2)), 139.1 (*C*(8)CH3), 132.7 (d, *J* 5.6, *C*(11)), 130.3 (d, *J* 3.1, *C*(14)H), 129.4 (*C*(7,9)H), 129.3 *C*(13), 129.3 (*C*(6,10)H), 128.5 (d, *J* 1.9, *C*(15)H), 125.4 (d, *J* 3.2, *C*(5)), 125.1 (dd, *J* 3.9, 3.9, *C*(16)H), 123.9 (dd, *J* 4.6, 4.2, *C*(12)H), 123.8 (d, *J* 272.7, *C*F3), 79.9 (d, *J* 170.3, *C*(4)), 65.1 (d, *J* 1.2, *C*(3)H), 55.2 (d, *J* 6.9, PO*C*H3), 54.8 (d, *J* 7.5, PO*C*H3), 21.1 (*C*H3); **^{19}F{^{1}H} NMR** (376 MHz, $CDCl_3$) δ_F: −63.0 ppm; **^{31}P{^{1}H}**

NMR (162 MHz, $CDCl_3$) δ_P: 17.7 ppm; **HRMS** [ESI]$^+$ $C_{19}H_{18}O_5F_3NaP$ [M+Na]$^+$ found 437.0734, requires 437.0736 (−0.49 ppm).

*Data for minor diastereomer syn-***63**: $[\alpha]_D^{20}$ − 7.4 (*c* 0.23, $CHCl_3$); **Chiral HPLC analysis** Chiralpak AD-H (96:4 hexane : IPA, flow rate 1.0 mLmin^{-1}, 211 nm, 30 ℃) t_R(2*R*,3*S*): 16.5 min, t_R(2*S*,3*R*): 19.1 min, >99:1 er_{syn}; **IR** v_{max} (film) 1848 (C=O), 1128 (P=O), 1032 (P-O); **^{1}H NMR** ($CDCl_3$, 400 MHz): 7.90 (2H, d, *J* 6.6, C(14,15)*H*), 7.71 (1H, d, *J* 7.6, C(12)*H*), 7.64 (1H, dd, *J* 8.2, 8.2, C(16)*H*), 7.43 (2H, d, *J* 8.1, C(6,10)*H*), 7.26 (2H, d, *J* 7.9, C(7,9)*H*), 5.16 (1H, d, *J* 12.8, C(3)*H*), 3.43 (3H, d, *J* 10.9, POC*H*$_3$), 3.40 (3H, d, *J* 10.8, POC*H*$_3$), 2.38 (3H, s, C*H*$_3$); **^{13}C{^{1}H} NMR** (101 MHz, $CDCl_3$) δ_C: (please note: some signals were not visible due to low concentration of this minor diastereomer) 137.0 (*C*(8)CH_3), 130.3 (*C*(14)H), 129.4 (*C*(13)), 129.4 (*C*(7,9)H), 129.3 (*C*(6,10)H), 128.7 (*C*(15)H), 125.9 (*C*(16)H), 122.5 (*C*(12)H), 67.5 (d, *J* 2.4, *C*(3)H), 54.5 (d, *J* 6.9, PO*C*H_3), 53.7 (d, *J* 7.4, PO*C*H_3), 21.1 (*C*H_3); **^{19}F{^{1}H} NMR** (MHz, $CDCl_3$) δ_F: −62.7 ppm; **^{31}P{^{1}H} NMR** (162 MHz, $CDCl_3$) δ_P: 15.1 ppm; **HRMS** [ESI]$^+$ $C_{19}H_{19}O_5F_3P$ [M+H]$^+$ found 415.0903, requires 415.0917 (−3.30 ppm).

Dimethyl ((2*R*,3*R*)-2-(3-fluorophenyl)-4-oxo-3-(p-tolyl)oxetan-2-yl)phosphonate (*anti*-64) and dimethyl ((2*R*,3*S*)-2-(3-fluorophenyl)-4-oxo-3-(p-tolyl)oxetan-2-yl)phosphonate (*syn*-64)

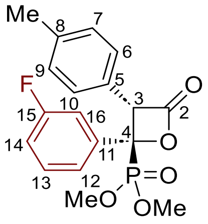

anti-**64**

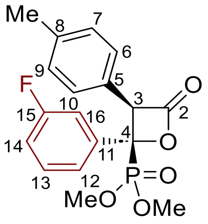

syn-**64**

Following **General Procedure D** with pivaloyl chloride (74 µL, 0.6 mmol), *i*-Pr_2NEt (105 µL, 0.6 mmol) and 4-tolylacetic acid (90.1 mg, 0.6 mmol) in MTBE (1.6 mL, 0.375 M). Then *i*-Pr_2NEt (35 µL, 0.2 mmol), (2*S*,3*R*)-HyperBTM (3.1 mg, 5 mol%), and dimethyl (3-fluorobenzoyl)phosphonate **S32** (46.4 mg, 0.2 mmol) in MTBE (0.4 mL, 0.5 M) at 25 °C for 16 h gave the crude product (90:10 $dr_{anti:syn}$). The crude mixture was purified by flash silica column chromatography [hexane : EtOAc (50:50 200 mL, 40:60 200 mL,), R_f = 0.21 (*anti*-**64**) and 0.35 (*syn*-**64**)], then the solvents removed *in vacuo* to yield *anti*-**64** as a colourless oil (57.1 mg, 78%) and *syn*-**64** as a colourless oil (7.8 mg, 11%).

*Data for major diastereomer anti-***64**: $[\alpha]_D^{20}$ + 67.1 (*c* 3.04, $CHCl_3$); **Chiral HPLC analysis** Chiralcel OD-H (95:5 hexane : IPA, flow rate 1.0 mLmin^{-1}, 211 nm, 30 ℃) t_R(2*S*,3*S*): 14.7 min, t_R(2*R*,3*R*): 16.9 min, 98:2 er_{anti}; **IR** v_{max} (film) 1844 (C=O), 1261 (P=O), 1059 (P-O), 1032 (P-O); **^{1}H NMR** ($CDCl_3$, 400 MHz): 7.19-7.09 (1H, m, C(12)*H*), 6.95 (2H, d, *J* 7.7, C(7,9)*H*), 6.93-6.86 (3H, m, C(14,15,16)*H*), 6.77 (2H, ddd, *J* 8.1, 4.6, 2.3, C(6,10)*H*), 5.51 (1H, d, *J* 10.8, C(3)*H*), 3.93 (3H, d, *J* 10.6, POC*H*$_3$), 3.64 (3H, d, *J* 10.5, POC*H*$_3$), 2.23 (3H, s, C(8)C*H*$_3$); **^{13}C{^{1}H} NMR** (101 MHz, $CDCl_3$) δ_C: 167.7 (d, *J* 8.7, *C*(2)), 162.3 (dd, *J*

247.1, 2.5, *C*(13)F), 138.8 (*C*(8)CH$_3$), 133.9 (dd, *J* 7.8, 5.6, *C*(11)), 129.7 (dd, *J* 8.2, 2.4, *C*(12)H), 129.4 (*C*(7,9)H), 129.3 (*C*(6,10)H), 125.6, d, *J* 3.3, *C*(5)), 122.6 (dd, *J* 3.6, 3.6, *C*(15)H), 115.5 (dd, *J* 21.0, 2.9, *C*(14)H), 114.1 (dd, *J* 24.1, 4.1, *C*(16)H), 79.9 (dd, *J* 169.7, 2.1, *C*(4)), 64.9 (d, *J* 1.8, *C*(3)H), 55.2 (d, *J* 6.9, PO*C*H$_3$), 54.7 (d, *J* 7.6, PO*C*H$_3$), 21.1 (C(8)*C*H$_3$); **^{19}F{^{1}H} NMR** (376 MHz, CDCl$_3$) δ_F: −112.0 ppm; **^{31}P{^{1}H} NMR** (162 MHz, CDCl$_3$) δ_P: 17.8 (d, *J* 0.9) ppm; **HRMS** [ESI]$^+$ $C_{18}H_{19}FO_5P$ [M+H]$^+$ found 365.0961, requires 365.0949 (+3.40 ppm).

*Data for minor diastereomer syn-***64**: $[\alpha]_D^{20}$ + 38.0 (*c* 0.05, CHCl$_3$); **Chiral HPLC analysis** Chiralpak IA (95:5 hexane : IPA, flow rate 1.0 mLmin^{-1}, 211 nm, 30 ℃) t_R(2*R*,3*S*): 20.9 min, t_R(2*S*,3*R*): 28.1 min, 97:3 er$_{syn}$; **IR** ν$_{max}$ (film) 1836 (C=O), 1263 (P=O), 1028 (P-O); **^{1}H NMR** (CDCl$_3$, 400 MHz): 7.51-7.45 (2H, m, C(14,15)*H*), 7.42 (2H, d, *J* 7.9, C(6,10)*H*), 7.41-7.37 (1H, m, C(12)*H*), 7.25 (2H, d, *J* 7.3, C(7,9)*H*),7.13-7.10 (1H, m, C(16)*H*), 5.14 (1H, d, *J* 12.6, C(3)*H*), 3.42 (3H, d, *J* 10.9, POC*H*$_3$), 3.40 (3H, d, *J* 10.7, POC*H*$_3$), 2.37 (3H, C(8)C*H*$_3$); **^{13}C{^{1}H} NMR** (101 MHz, CDCl$_3$) δ_C: 166.6 (d, *J* 4.5, *C*(2)), 162.7 (dd, *J* 248.0, 1.8, *C*(13)F), 138.8 (d, *J* 5.5, *C*(11)), 138.8 (*C*(8)CH$_3$), 130.6 (dd, *J* 8.1, 1.5, *C*(14)H), 129.3 (*C*(7,9)H), 128.7 (*C*(6,10)H), 126.0 (d, *J* 2.4, *C*(5), 121.4 (dd, *J* 4.3, 3.6, *C*(15)H), 116.1 (dd, *J* 21.1, 1.5, *C*(16)H), 113.1 (dd, *J* 23.8, 3.3, *C*(12)H), 81.0 (dd, *J* 172.1, 1.1, *C*(4)), 67.5 (d, *J* 2.8, *C*(3)H), 54.5 (d, *J* 6.8, PO*C*H$_3$), 53.6 (d, *J* 7.2, PO*C*H$_3$), 21.3 (C(8)*C*H$_3$); **^{19}F{^{1}H} NMR** (376 MHz, CDCl$_3$) δ_F: −110.8 ppm; **^{31}P{^{1}H} NMR** (162 MHz, CDCl$_3$) δ_P: 15.3 ppm; **HRMS** [ESI]$^+$ $C_{18}H_{18}FO_5P$ [M]$^+$ found 364.0871, requires 364.0870 (+0.16 ppm).

Dimethyl ((2*R*,3*R*)-2-(3-methoxyphenyl)-4-oxo-3-(p-tolyl)oxetan-2-yl)phosphonate (*anti*-65)

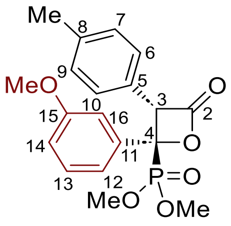

anti-**65**

Following **General Procedure D** with pivaloyl chloride (74 µL, 0.6 mmol), *i*-Pr$_2$NEt (105 µL, 0.6 mmol) and 4-tolylacetic acid (90.1 mg, 0.6 mmol) in MTBE (1.6 mL, 0.375 M). Then *i*-Pr$_2$NEt (35 µL, 0.2 mmol), (2*S*,3*R*)-HyperBTM (3.1 mg, 5 mol%), and dimethyl (3-methoxybenzoyl)phosphonate **S25** (48.8 mg, 0.2 mmol) in MTBE (0.4 mL, 0.5 M) at 25 °C for 16 h gave the crude product (86:14 dr$_{anti:syn}$). The crude mixture was purified by flash silica column chromatography [hexane : EtOAc (60:40 200 mL, 55:45 200 mL, 50:50 200 mL, 40:60 200 mL), R_f = 0.19 (*anti*-**65**)], then the solvents removed *in vacuo* to yield only *anti*-**65** (50.4 mg, 67%) as a yellow oil. Further attempts to reproduce the reaction and isolate *syn*-**65** were made, but to no avail, it is suspected that it may degrade on silica. $[\alpha]_D^{20}$ + 63.0 (*c* 0.10, CHCl$_3$); **Chiral HPLC analysis** Chiralpak ID (95:5 hexane : IPA, flow rate 1.5 mLmin^{-1}, 211 nm, 30 ℃) t_R(2*S*,3*S*): 31.0 min, t_R(2*R*,3*R*): 37.1 min, 95:5 er$_{anti}$; **IR** ν$_{max}$ (film) 1842 (C=O), 1263 (P=O), 1036 (P-O); **^{1}H NMR**

(400 MHz, $CDCl_3$): δ_H: 7.09 (1H, dd, *J* 7.9, 7.9, C(16)*H*), 6.96 (2H, d, *J* 7.3, C(7,9)*H*), 6.80 (2H, d, *J* 6.5, C(6,10)*H*), 6.74 (2H, d, *J* 7.5, 7.5, C(14,15)*H*), 6.67 (1H, s, C(12)*H*), 5.49 (1H, d, *J* 11.2, C(3)*H*), 3.93 (3H, d, *J* 10.6, POCH_3), 3.62 (3H, s, C(13)OCH_3), 3.61 (3H, d, *J* 11.2, POCH_3), 2.24 (3H, s, CH_3); **^{13}C{^{1}H} NMR** (102 MHz, $CDCl_3$) δ_C: 168.3 (d, *J* 8.4, *C*(2)), 159.2 (*C*(13)OCH_3), 138.6 (*C*(8)CH_3), 132.6 (*C*(11)), 129.5 (*C*(6,10)H), 129.3 (*C*(7,9)H), 129.1 (d, *J* 1.9, *C*(16)H), 126.0 (d, *J* 3.4, *C*(5)), 119.3 (d, *J* 4.0, *C*(15)H), 114.8 (d, *J* 2.9, *C*(14)H), 112.0 (d, *J* 3.4, *C*(12)H), 81.0 (d, *J* 136.7, *C*(4)), 64.7 (d, *J* 6.0, *C*(3)H), 55.2 (C(13)O*C*H_3), 55.2 (d, *J* 6.8, PO*C*H_3), 54.5 (d, *J* 7.5, PO*C*H_3), 21.1 (*C*H_3); **^{31}P{^{1}H} NMR** (162 MHz, $CDCl_3$) δ_P: 18.2 ppm; **HRMS** [ESI]$^+$ $C_{19}H_{22}O_6P$ [M+H]$^+$ found 377.1142, requires 377.1149 (−1.72 ppm).

Dimethyl ((2*R*,3*R*)-2-(2-fluorophenyl)-4-oxo-3-(p-tolyl)oxetan-2-yl)phosphonate (*anti*-66) and dimethyl ((2*R*,3*S*)-2-(2-fluorophenyl)-4-oxo-3-(p-tolyl)oxetan-2-yl)phosphonate (*syn*-66)

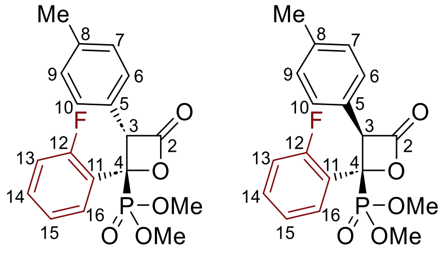

anti-**66** *syn*-**66**

Following General Procedure D with pivaloyl chloride (74 μL, 0.6 mmol), *i*-Pr_2NEt (105 μL, 0.6 mmol) and 4-tolylacetic acid (90.1 mg, 0.6 mmol) in MTBE (1.6 mL, 0.375 M). Then *i*-Pr_2NEt (35 μL, 0.2 mmol), (2*S*,3*R*)-HyperBTM (3.1 mg, 5 mol%), and dimethyl (2-fluorobenzoyl)phosphonate **S33** (46.4 mg, 0.2 mmol) in MTBE (0.4 mL, 0.5 M) at 25 °C for 16 h gave the crude product (86:14 $dr_{anti:syn}$). The crude mixture was purified by flash silica column chromatography [hexane : EtOAc (70:30 150 mL, 65:35 150 mL, 60:40 150 mL, 55:45 150 mL), R_f = 0.09 (*syn*-**66**) and 0.22 (*anti*-**66**)], then the solvents removed *in vacuo* to yield *anti*-**66** as a colourless oil (21.3 mg, 29%) and *syn*-**66** as a colourless oil (3.5 mg, 5%). Unfortunately, due to difficulties purifying these compounds, there are minor impurities in the NMR spectra.

Data for major diastereomer anti-**66**: $[\alpha]_D^{20}$ + 75.9 (*c* 1.44, $CHCl_3$); **Chiral HPLC analysis** Chiralpak AD-H (95:5 hexane : IPA, flow rate 1.0 mLmin^{-1}, 211 nm, 30 ℃) t_R(2*R*,3*R*): 20.7 min, t_R(2*S*,3*S*): 25.8 min, 96:4 er_{anti}; **IR** ν_{max} (film) 1844 (C=O), 1261 (P=O), 1059 (P-O), 1034 (P-O); **^{1}H NMR** ($CDCl_3$, 400 MHz): (*please note, there are some solvent impurities present that were unable to be removed from this spectrum*) 7.56-7.46 (1H, m, C(13)*H*), 7.24-7.12 (2H, m, C(14,15)*H*), 6.94 (2H, d, *J* 7.7, C(7,9)*H*), 6.80 (2H, d, *J* 7.7, C(6,10)*H*), 6.72 (1H, dd, *J* 9.4, 9.4, C(16)*H*), 5.53 (1H, d, *J* 10.1, C(3)*H*), 3.90 (3H, dd, *J* 10.7, 1.7, POCH_3), 3.79 (3H, dd, *J* 10.7, 1.7, POCH_3), 2.25 (3H, s, ArCH_3); **^{13}C{^{1}H} NMR** (101 MHz, $CDCl_3$) δ_C: 168.0 (d, *J* 6.9, *C*(2)), 158.6 (d, *J* 252.9, *C*(12)F), 138.7 (*C*(8)CH_3), 131.0 (dd, *J* 8.7, 2.7, *C*(15)H), 129.5 (*C*(6,10)H), 129.3 (d, *J* 18.6, *C*(11)), 128.7 (*C*(7,9)H), 128.7 (dd, *J* 4.4, 4.4, *C*(13)H), 126.3

(*C*(5)), 124.3 (*C*(14)H), 115.8 (d, *J* 21.6, *C*(16)H), 80.3, dd, *J* 172.5, 4.7, *C*(4)), 64.8 (d, *J* 2.3, *C*(3)H), 55.0 (d, *J* 6.4, PO*C*H_3), 54.7 (d, *J* 6.8, PO*C*H_3), 21.2 (CH_3)); **^{19}F{^{1}H} NMR** (376 MHz, $CDCl_3$) δ_F: −107.3; **^{31}P{^{1}H} NMR** (162 MHz, $CDCl_3$) δ_P: 17.8 (d, *J* 4.1); **HRMS** [ESI]$^+$ $C_{18}H_{19}O_5FP$ [M+H]$^+$ found 365.0951, requires 365.0949 (−0.66 ppm).

Data for minor diastereomer syn-**66**: $[\alpha]_D^{20}$ + 6.7 (*c* 0.09, $CHCl_3$); **Chiral HPLC analysis** Chiralpak IA (96:4 hexane : IPA, flow rate 1.5 mLmin^{-1}, 211 nm, 40 ℃) t_R(2*R*,3*S*): 17.2 min, t_R(2*S*,3*R*): 18.4 min, 75:25 er_{syn}; **IR** v_{max} (film) 1707 (C=O), 1265 (P=O), 1038 (P-O); **^{1}H NMR** ($CDCl_3$, 400 MHz): (*please note, there are some solvent impurities present that were unable to be removed from this spectrum*) 7.60-7.53 (3H, m, C(6,10)*H* and C(14)*H*), 7.48-7.41 (1H, m, C(13)*H*), 7.30-7.16 (4H, m, C(7,9)*H* and C(15,16)*H*), 5.29 (1H, d, *J* 12.7, C(3)*H*), 3.46 (3H, d, *J* 10.8, POC*H*$_3$), 3.39 (3H, d, *J* 10.8, POC*H*$_3$), 2.37 (3H, s, C*H*$_3$); **^{13}C{^{1}H} NMR** (101 MHz, $CDCl_3$) δ_C: 167.2 (d, *J* 3.9, *C*(2)), 163.1 (dd, *J* 8.9, 2.7, *C*(12)F), 138.7 (d, *J* 22.0, *C*(11)), 138.6 (*C*(8)CH_3), 131.3 (d, *J* 3.9, *C*(13)H), 129.7 (d, *J* 5.4, *C*(6,10)H), 129.1 (*C*(7,9)H), 126.3 (d, *J* 3.8, *C*(15)H), 124.5 (d, *J* 1.9, *C*(14)H), 116.1 (d, *J* 3.5, *C*(16)H), 80.6 (d, *J* 171.6, *C*(4)), 66.3 (d, *J* 3.2, *C*(3)H), 54.2 (d, *J* 6.3, PO*C*H_3), 53.7 (d, *J* 6.8, PO*C*H_3), 21.3 (*C*H_3); **^{19}F{^{1}H} NMR** (MHz, $CDCl_3$) δ_F: −111.1 (d, *J* 3.7) ppm; **^{31}P{^{1}H} NMR** (162 MHz, $CDCl_3$) δ_P: 15.1 (d, *J* 3.8) ppm; **HRMS** [ESI]$^+$ $C_{18}H_{19}O_5F_1P$ [M+H]$^+$ found 365.0955, requires 365.0949 (+1.75 ppm).

8. Crystallographic Analysis

X-ray diffraction data for compound **18** were collected using a Rigaku FR-X Ultrahigh Brilliance Microfocus RA generator/confocal optics with XtaLAB P200 diffractometer [Mo Kα radiation (λ = 0.71073 Å)]. Diffraction data for compounds **26** and **55** were collected using a Rigaku MM-007HF High Brilliance RA generator/confocal optics with either XtaLAB P200 or P100 diffractometers [Cu Kα radiation (λ = 1.54187 Å)]. Data for all compounds analysed were collected using either CrystalClear[29] (using ω steps and accumulating area detector images spanning at least a hemisphere of reciprocal space) or CrysAlisPro[30] (using a calculated strategy) and processed (including correction for Lorentz, polarization and absorption) using CrysAlisPro. Structures were solved by dual-space methods (SHELXT[31]) and refined by full-matrix least-squares against F^2 (SHELXL-2019/3[32]). Non-hydrogen atoms were refined anisotropically, and hydrogen atoms were refined using a riding model. All calculations were performed using the Olex2[33] interface. Selected crystallographic data are presented in Table S11. CCDC 2395570-2395572 contains the supplementary crystallographic data for this paper. These data can be obtained free of charge from The Cambridge Crystallographic Data Centre via www.ccdc.cam.ac.uk/structures.

Table S11. Selected crystallographic data.

	**18**	**26**	**55**
formula	$C_{14}H_{17}O_7P$	$C_{16}H_{17}O_5P$	$C_{19}H_{17}O_5PS$
fw	328.24	320.26	388.35
temperature [K]	173	173	125
crystal description	Colourless plate	Colourless rod	Colourless needle
crystal size [mm^3]	0.18×0.03×0.01	0.36×0.05×0.03	0.16×0.02×0.01
space group	$P1$	$P2_12_12_1$	$P2_1$
a [Å]	6.35385(12)	6.2790(2)	10.3633(5)
b [Å]	7.76034(15)	8.1645(3)	7.8457(3)
c [Å]	16.0686(3)	30.2310(10)	12.0190(6)
α [°]	92.9757(16)		
β [°]	90.6187(17)		110.666(6)
γ [°]	101.8462(16)		
vol [Å]^3	774.21(3)	1549.79(9)	914.35(8)
Z	2	4	2
ρ (calc) [g/cm^3]	1.408	1.373	1.411
μ [mm^{-1}]	0.209	1.768	2.644
F(000)	344	672	404
reflections collected	43057	16081	33520
independent reflections (R_{int})	8879 (0.0455)	2816 (0.0915)	3660 (0.1138)
parameters, restraints	405, 3	202, 0	238, 1
GoF on F^2	1.025	1.010	1.138
R_1 [$I > 2\sigma(I)$]s	0.0368	0.0446	0.0621
wR_2 (all data)	0.0876	0.1135	0.1855
largest diff. peak/hole [e/Å^3]	0.342, -0.211	0.079, -0.469	0.412, -0.495
Flack parameter	-0.03(3)	-0.03(3)	-0.04(2)

9. References

1 D. Daniels, S. Smith, T. Lebl, P. Shapland and A. Smith, *Synthesis*, 2014, **47**, 34–41.

2 L. C. Morrill, J. Douglas, T. Lebl, A. M. Z. Slawin, D. J. Fox and A. D. Smith, *Chem. Sci.*, 2013, **4**, 4146.

3 M. J. Crimmin, P. J. O'Hanlon and N. H. Rogers, *J. Chem. Soc., Perkin Trans. 1*, 1985, 541.

4 A. V. Shtelman and J. Y. Becker, *Tetrahedron Lett.*, 2008, **49**, 3101–3103.

5 J. Sun, F. He, Z. Wang, D. Pan, P. Zheng, C. Mou, Z. Jin and Y. R. Chi, *Chem. Commun.*, 2018, **54**, 6040–6043.

6 Y. Wang, C. M. Young, H. Liu, W. C. Hartley, M. Wienhold, David. B. Cordes, A. M. Z. Slawin and A. D. Smith, *Angew. Chem. Int. Ed.*, 2022, **61**, e202208800.

7 A. V. Shtelman and J. Y. Becker, *Tetrahedron*, 2011, **67**, 1135–1141.

8 M. Bellassoued, J. E. Dubois and E. Bertounesque, *Synth. Commun.*, 1987, **17**, 1181–1183.

9 P. A. Grieco, C.-L. J. Wang and S. D. Burke, *J. Chem. Soc., Chem. Commun.*, 1975, 537.

10 P. F. Hudrlik and D. Peterson, *J. Am. Chem. Soc.*, 1975, **97**, 1464–1468.

11 A. J. Blake, C. L. Friend, R. J. Outram, N. S. Simpkins and A. J. Whitehead, *Tetrahedron Lett.*, 2001, **42**, 2877–2881.

12 W. T. Brady and T. C. Cheng, *J. Organomet. Chem.*, 1977, **137**, 287–292.

13 S. Polat-Cakir and A. S. Demir, *Tetrahedron*, 2011, **67**, 2396–2401.

14 Y. Huang, F. Berthiol, B. Stegink, M. M. Pollard and A. J. Minnaard, *Adv. Synth. Catal.*, 2009, **351**, 1423–1430.

15 H. Cohen and C. Benezra, *Can. J. Chem.*, 1974, **52**, 66–79.

16 O. Seven, S. Polat-Cakir, Md. S. Hossain, M. Emrullahoglu and A. S. Demir, *Tetrahedron*, 2011, **67**, 3464–3469.

17 E. Migianu, I. Mallard, N. Bouchemal and M. Lecouvey, *Tetrahedron Lett.*, 2004, **45**, 4511–4513.

18 Nguyen Lan Mong, E. Niesor and C. L. Bentzen, *J. Med. Chem.*, 1987, **30**, 1426–1433.

19 X. Li, A. K. Szardenings, C. P. Holmes, L. Wang, A. Bhandari, L. Shi, M. Navre, L. Jang and J. R. Grove, *Tetrahedron Lett.*, 2006, **47**, 19–22.

20 S. Kong, W. Fan, G. Wu and Z. Miao, *Angew. Chem. Int. Ed.*, 2012, **51**, 8864–8867.

21 Y. Zhou, Y. Zhang and J. Wang, *Org. Biomol. Chem.*, 2016, **14**, 10444–10453.

22 X. Chen, J. Wang, Y. Zhu, D. Shang, B. Gao, X. Liu, X. Feng, Z. Su and C. Hu, *Chem. Eur. J.*, 2008, **14**, 10896–10899.

23 D. V. Griffiths, M. J. Al-Jeboori, Y.-K. Cheong, P. Duncanson, J. E. Harris, M. C. Salt and H. V. Taylor, *Org. Biomol. Chem.*, 2008, **6**, 577–585.

24 J. Dussart-Gautheret, J. Deschamp, T. Legigan, M. Monteil, E. Migianu-Griffoni and M. Lecouvey, *Molecules*, 2021, **26**, 7609.

25 M. Sprecher and D. Kost, *J. Am. Chem. Soc.*, 1994, **116**, 1016–1026.

26 N. Ishida, T. Yano, T. Yuhki and M. Murakami, *Chem. Asian. J.*, 2017, **12**, 1905–1908.

27 D. V. Griffiths, H. A. R. Jamali and J. C. Tebby, *Phosphorus, Sulfur Relat. Elem.*, 1981, **11**, 95–99.

28 Y. Wang, University of St Andrews, 2022.

29 *CrystalClear-SM Expert* v2.1. Rigaku Americas, *The Woodlands*, *Texas*, *USA*, and Rigaku Corporation, *Tokyo*, *Japan*, 2015.

30. *CrysAlisPro* v1.171.40.14a, 42.53a, 42.94a Rigaku Oxford Diffraction, Rigaku Corporation, *Tokyo*, *Japan*, 2018-2023.

31. G. M. Sheldrick, SHELXT – Integrated space-group and crystal structure determination, *Acta Crystallogr., Sect. A: Found. Adv.*, 2015, 71, 3-8.

32. G. M. Sheldrick, Crystal structure refinement with SHELXL. *Acta Crystallogr., Sect. C: Struct. Chem.*, 2015, 71, 3-8.

33. O. V. Dolomanov, L. J. Bourhis, R. J. Gildea, J. A. K. Howard, H. Puschmann, OLEX2: a complete structure solution, refinement and analysis program. *J. Appl. Crystallogr.*, 2009, 42, 339-341.

Appendix I. ^{1}H, ^{19}F, ^{31}P and ^{13}C NMR Spec

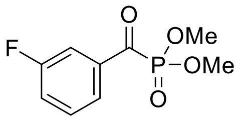

S32

^{1}H, $CDCl_3$, 400 MHz

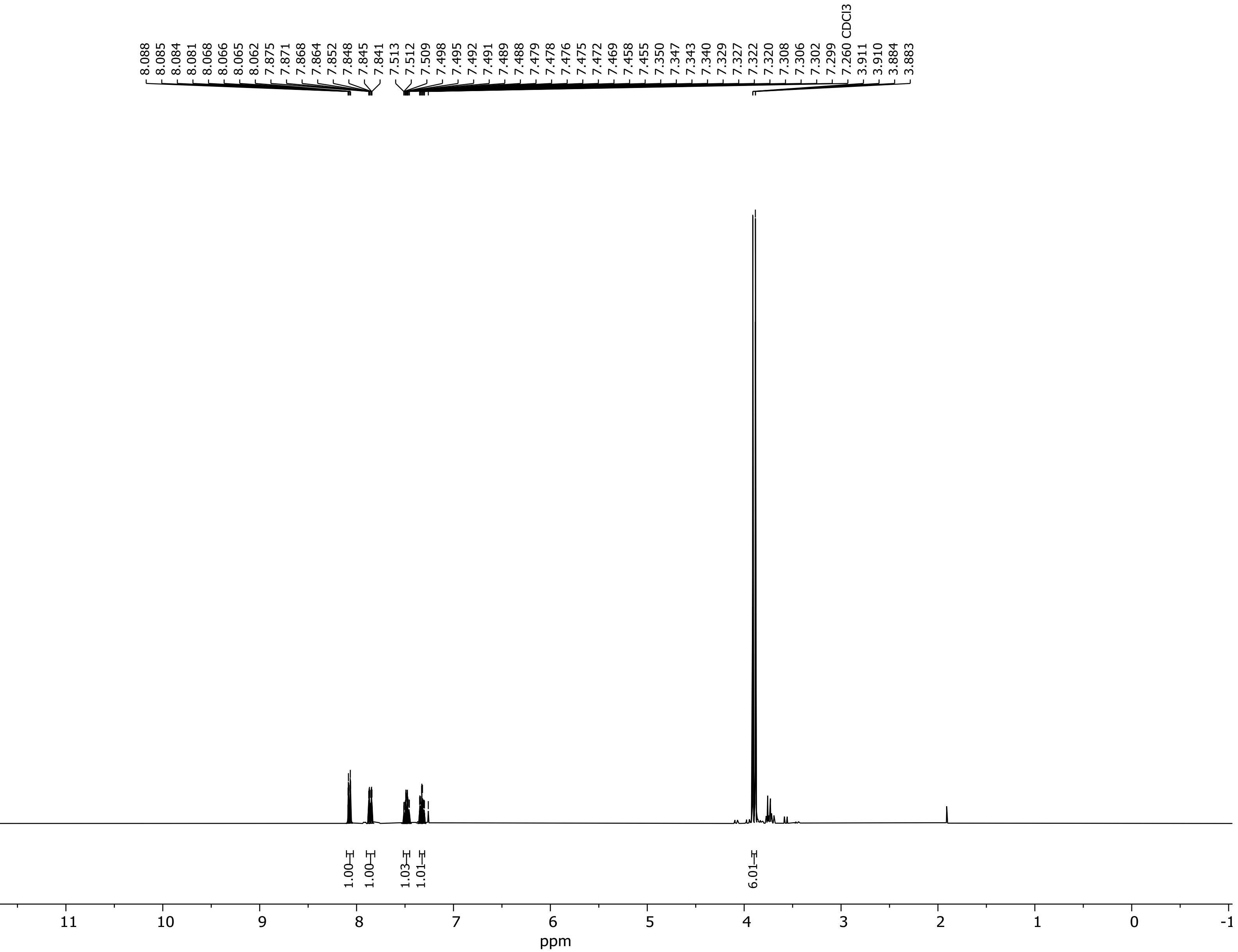

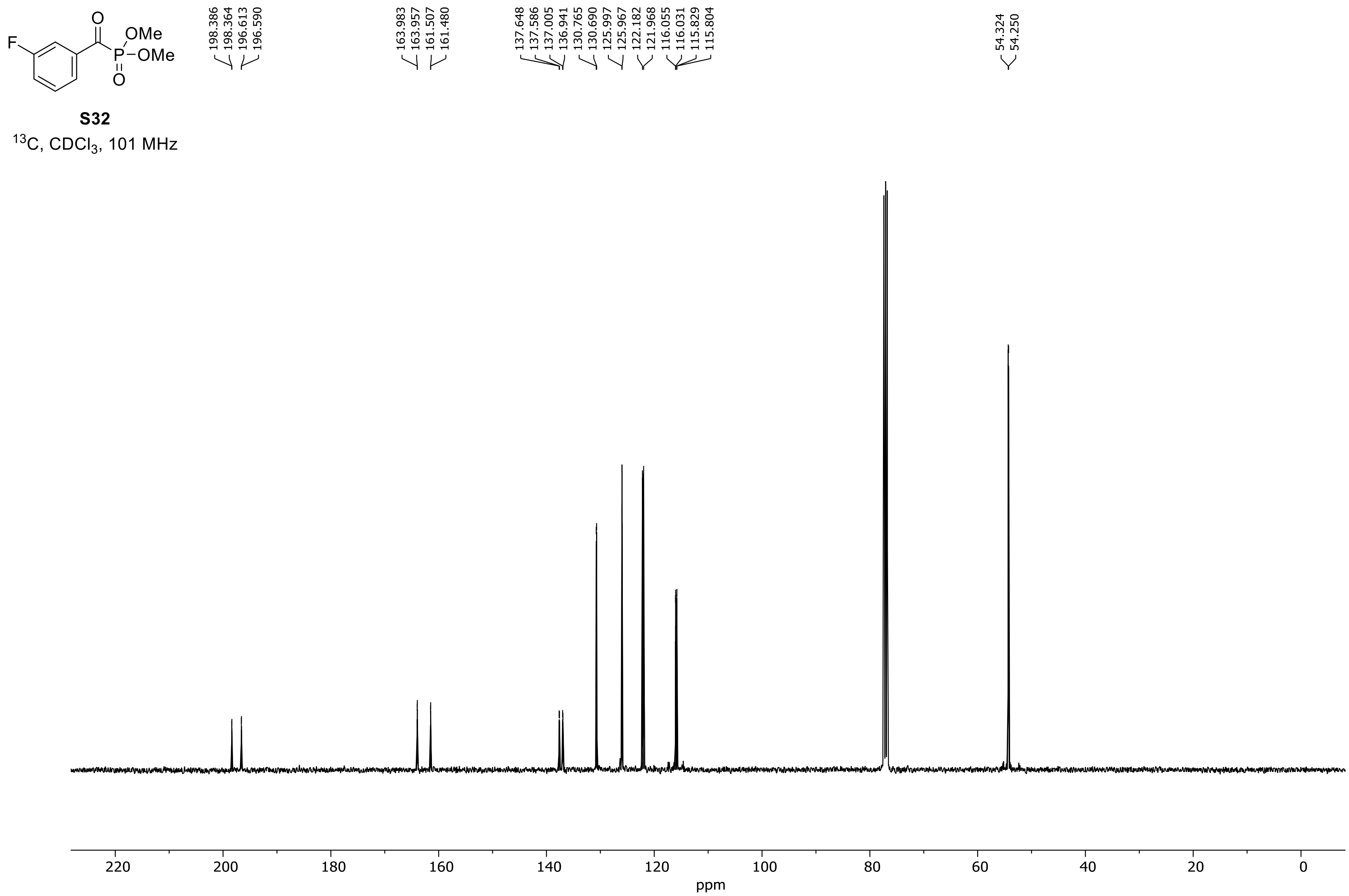

F
O
OMe
P–OMe
O
S32
^{13}C, $CDCl_3$, 101 MHz
198.386
198.364
196.613
196.590
163.983
163.957
161.507
161.480
137.648
137.586
137.005
136.941
130.765
130.690
125.997
125.967
122.182
121.968
116.055
116.031
115.829
115.804
54.324
54.250
220
200
180
160
140
120
100
80
60
40
20
0
ppm

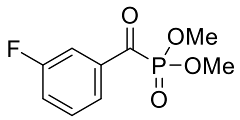

S32

^{19}F, $CDCl_3$, 376 MHz

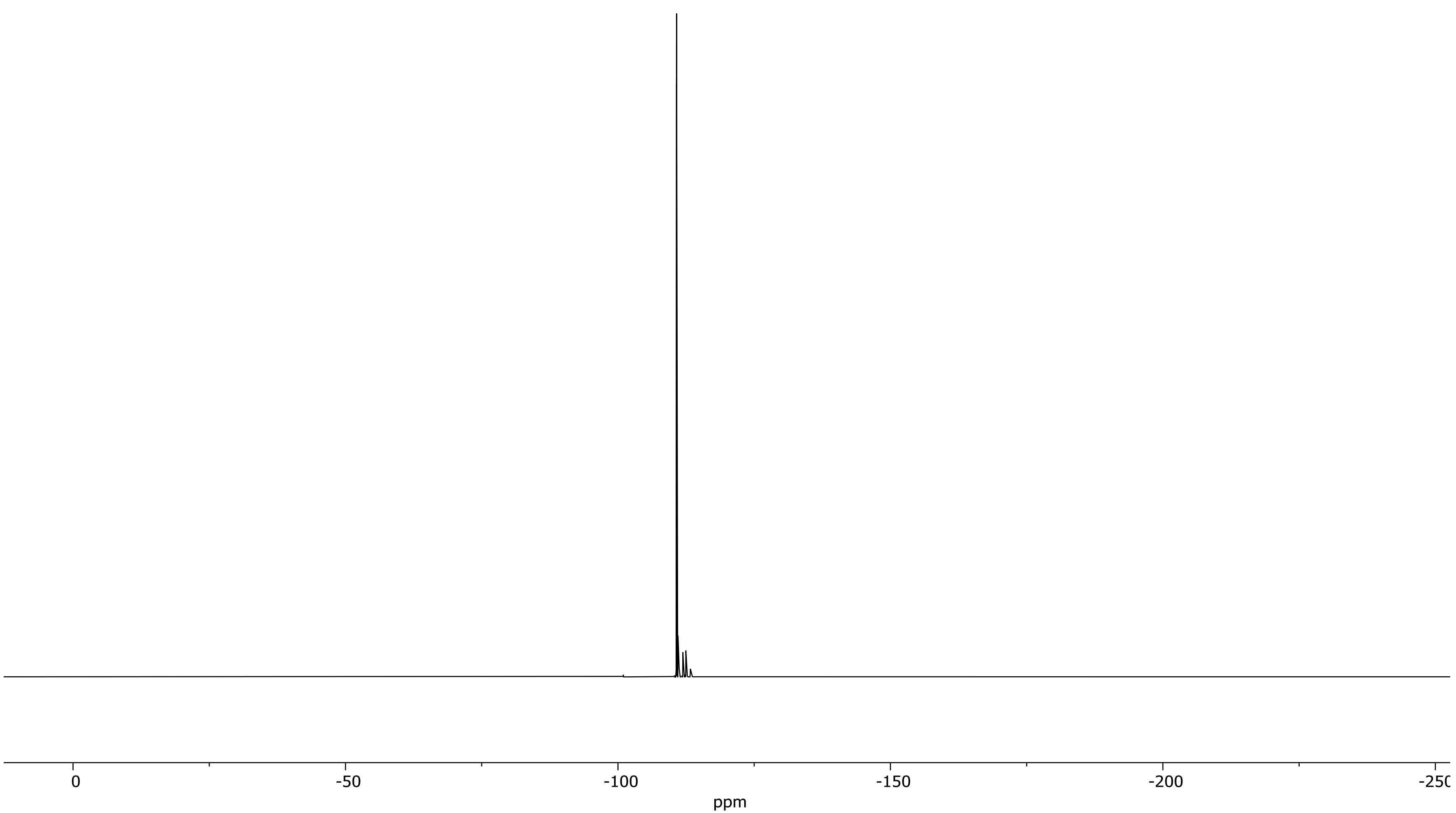

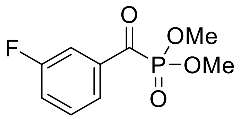

S32

^{31}P, $CDCl_3$, 162 MHz

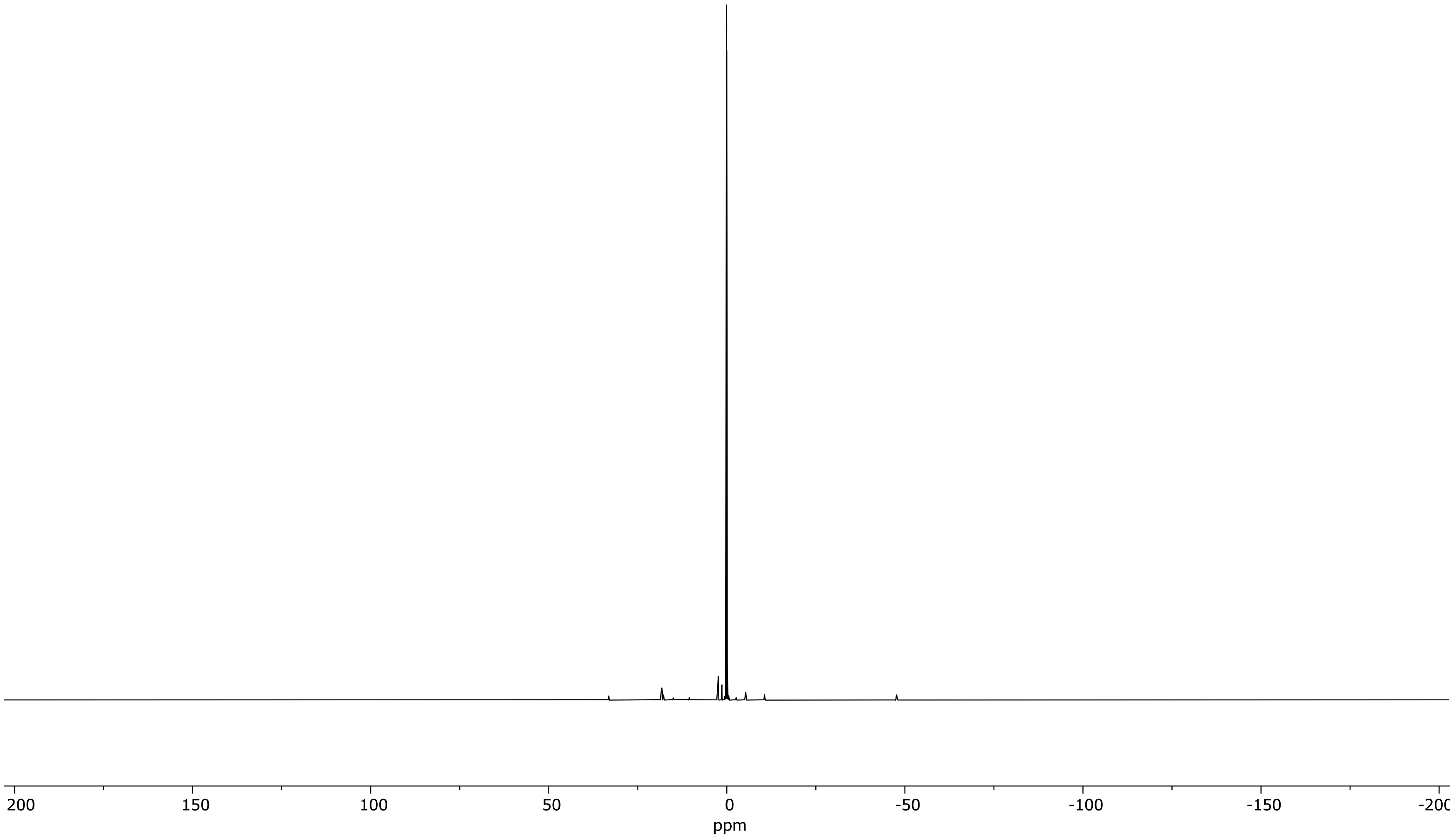

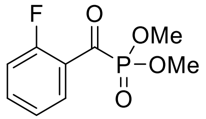

S33

^{1}H, $CDCl_3$, 400 MHz

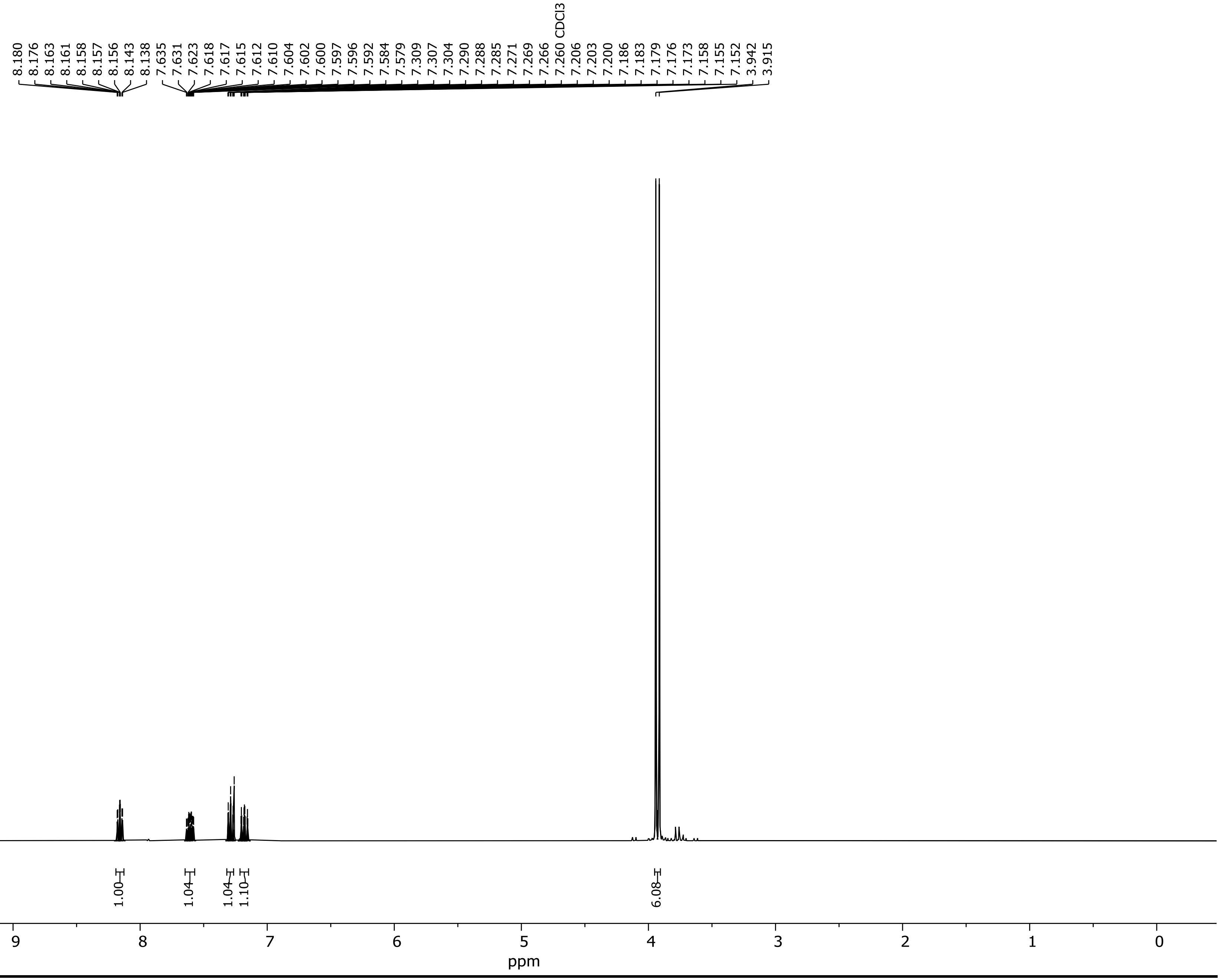

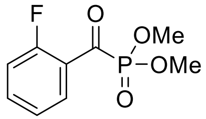

S33

^{13}C, $CDCl_3$, 101 MHz

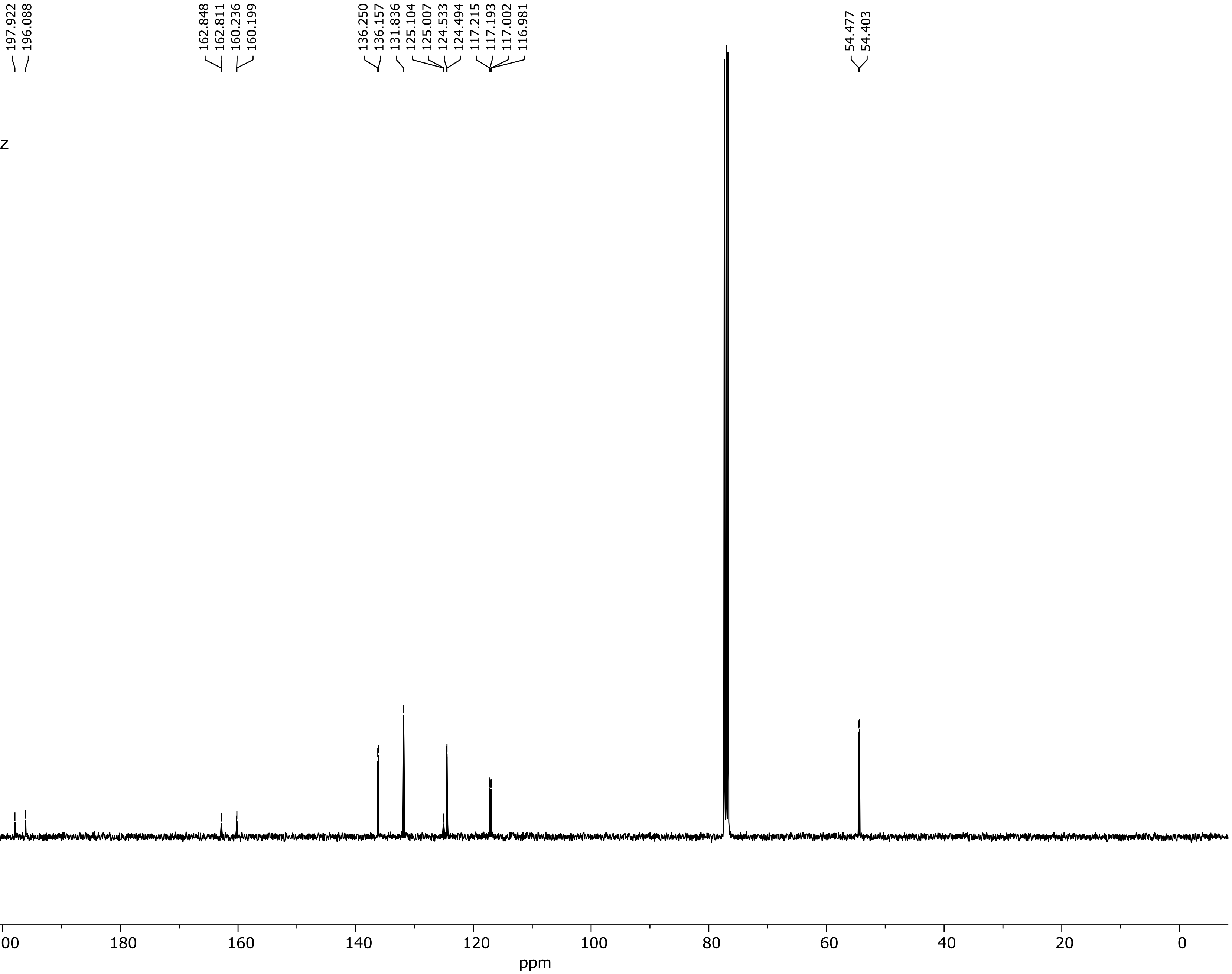

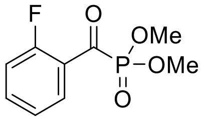

S33

^{19}F, $CDCl_3$, 376 MHz

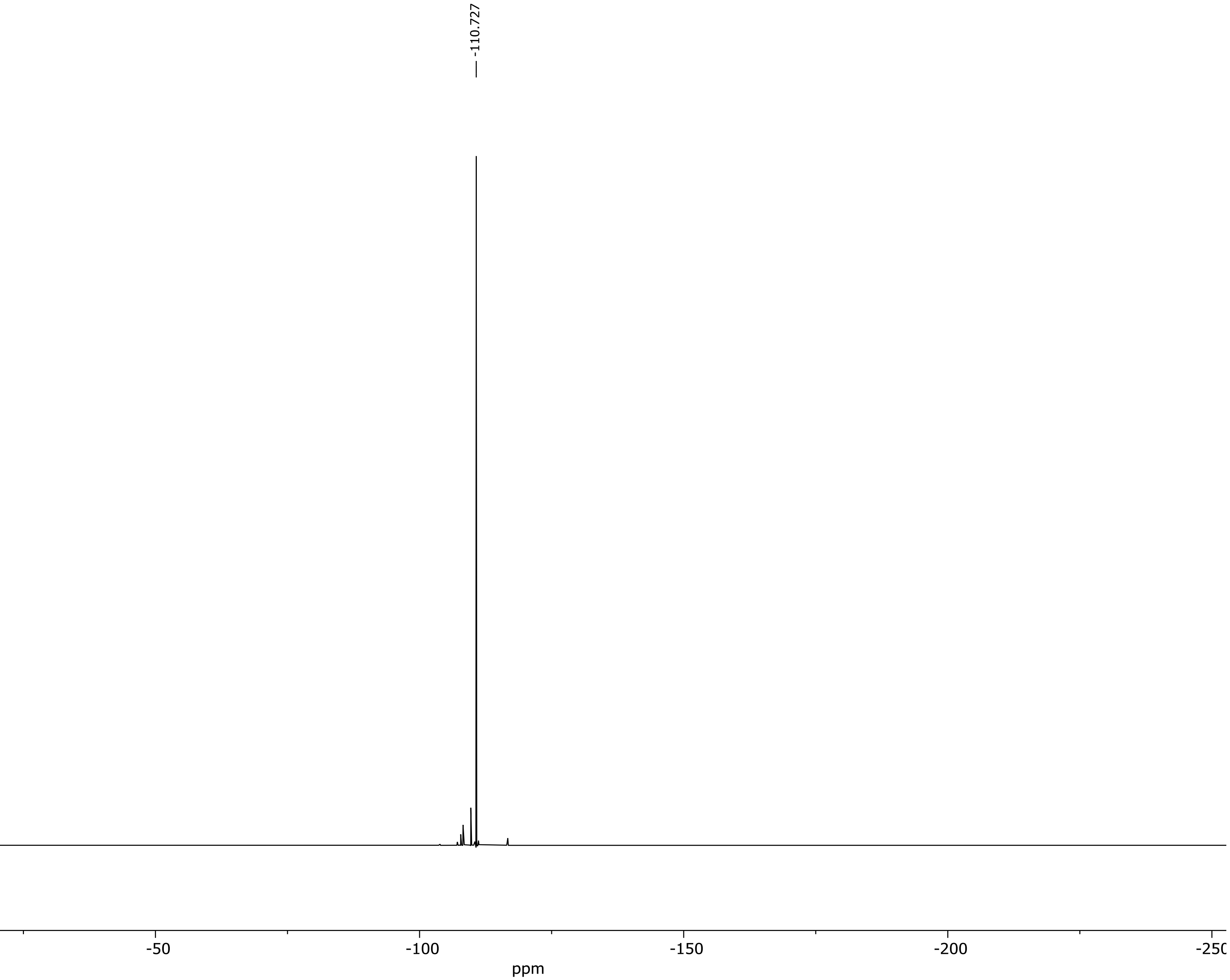

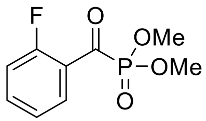

S33

^{31}P, $CDCl_3$, 162 MHz

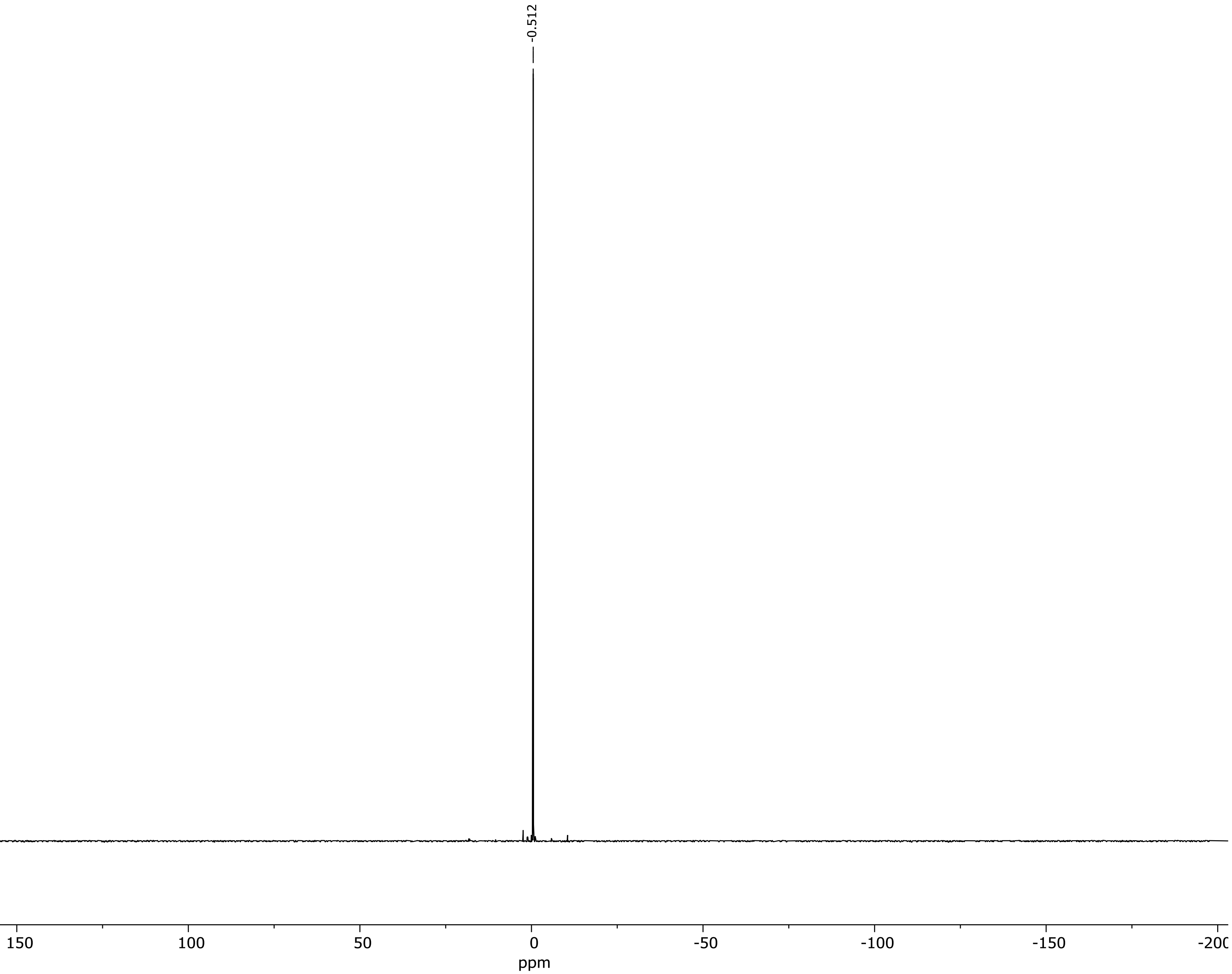

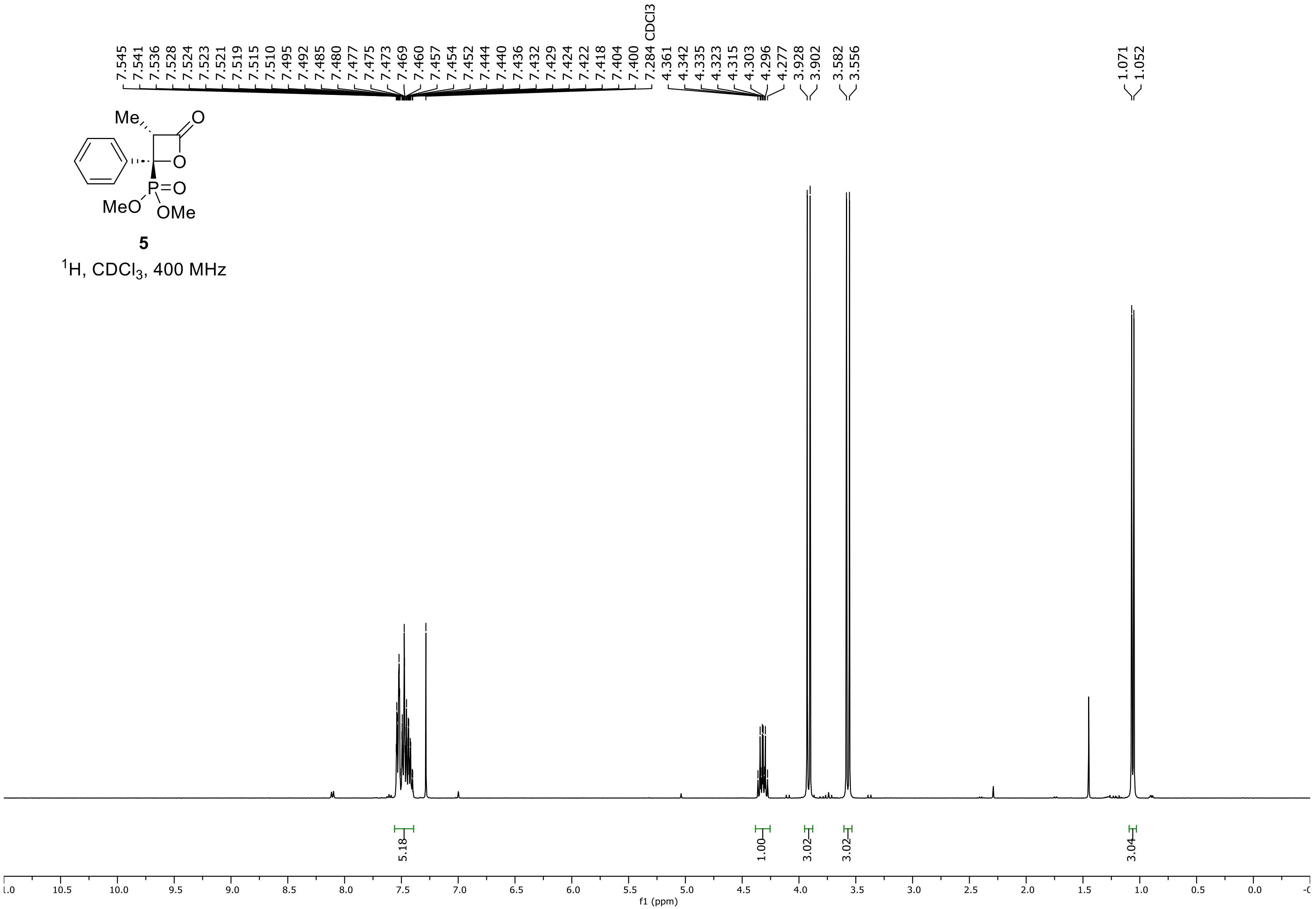

Me
O
O
P=O
MeO
OMe
5
¹H, CDCl3, 400 MHz
7.545 7.541 7.536 7.528 7.524 7.523 7.521 7.519 7.515 7.510 7.495 7.492 7.485 7.480 7.477 7.475 7.473 7.469 7.460 7.457 7.454 7.452 7.444 7.440 7.436 7.432 7.429 7.424 7.422 7.418 7.404 7.400 7.284 CDCl3
4.361 4.342 4.335 4.323 4.315 4.303 4.296 4.277
3.928 3.902
3.582 3.556
1.071 1.052
5.18
1.00
3.02
3.02
3.04
f1 (ppm)

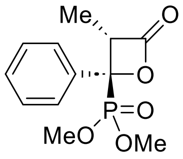

5

^{13}C, $CDCl_3$, 101 MHz

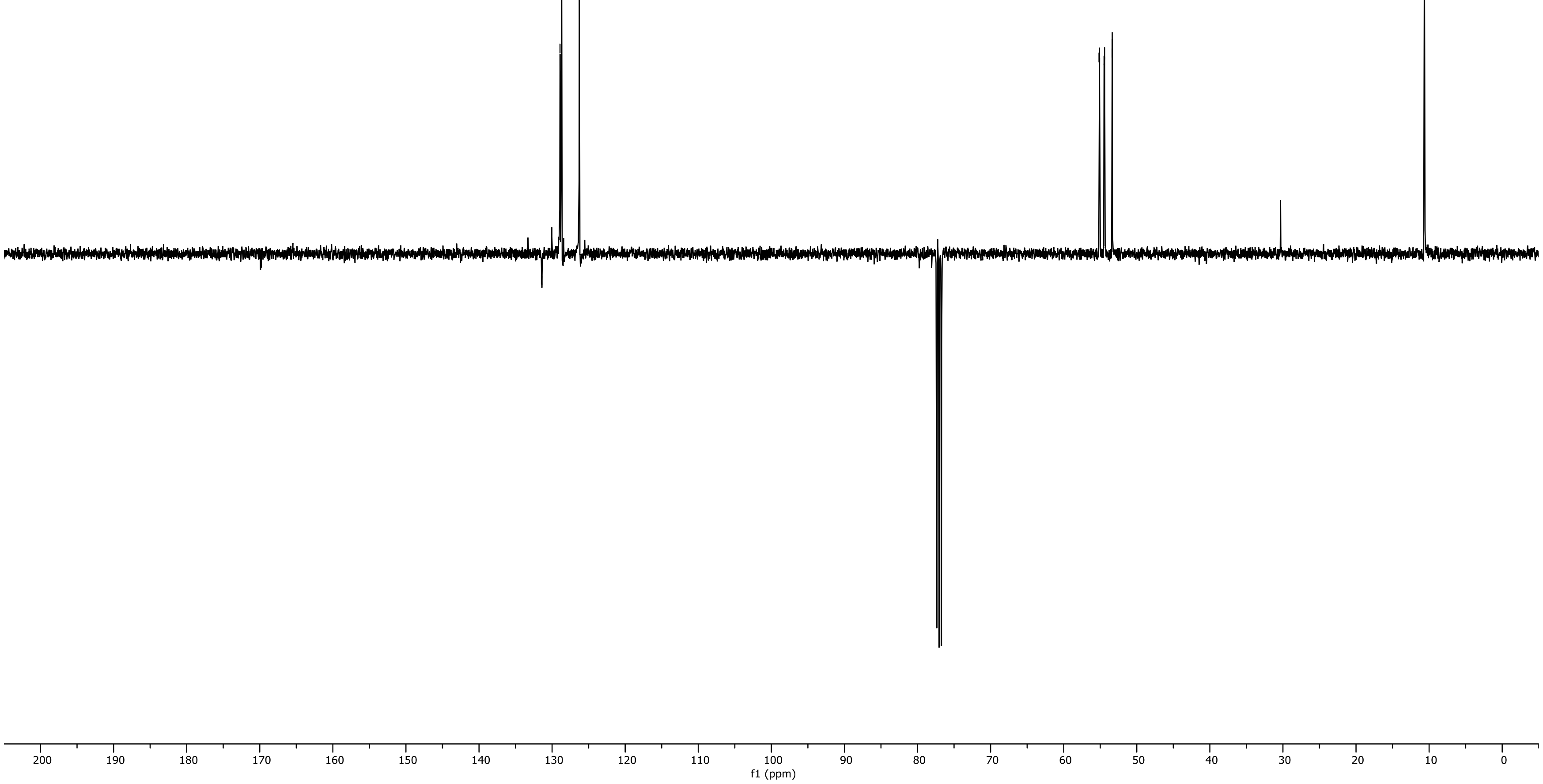

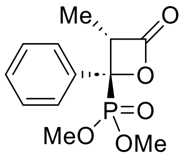

5

^{31}P, $CDCl_3$, 162 MHz

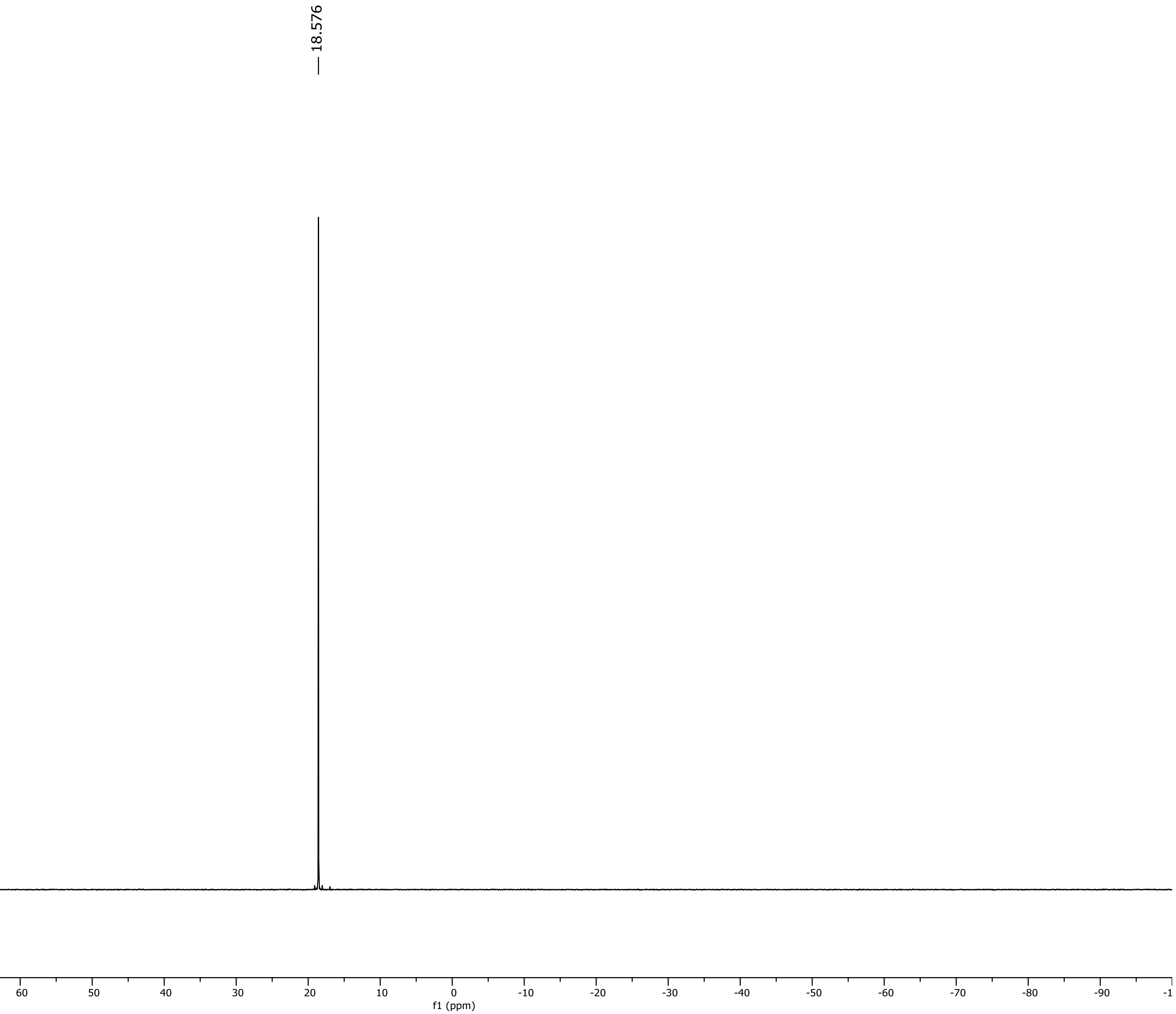

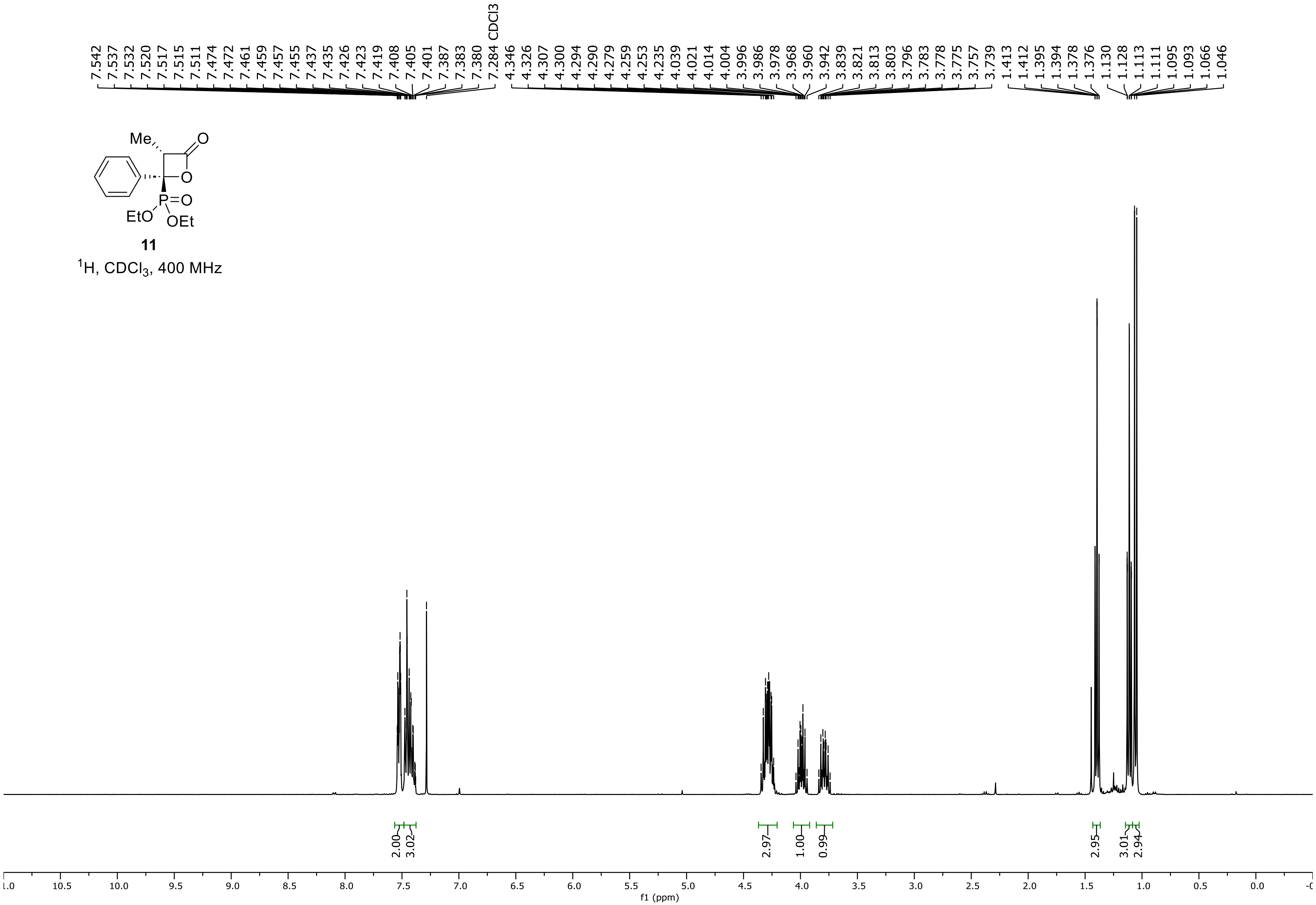
7.542 7.537 7.532 7.520 7.517 7.515 7.511 7.474 7.472 7.461 7.459 7.457 7.455 7.437 7.435 7.426 7.423 7.419 7.408 7.405 7.401 7.387 7.383 7.380 7.284 CDCl3
4.346 4.326 4.307 4.300 4.294 4.290 4.279 4.259 4.253 4.235 4.039 4.021 4.014 4.004 3.996 3.986 3.978 3.968 3.960 3.942 3.839 3.821 3.813 3.803 3.796 3.783 3.778 3.775 3.757 3.739
1.413 1.412 1.395 1.394 1.378 1.376 1.130 1.128 1.113 1.111 1.095 1.093 1.066 1.046
Me
O
EtO
OEt
P=O
11
1H, CDCl3, 400 MHz
2.00 3.02 2.97 1.00 0.99 2.95 3.01 2.94
f1 (ppm)

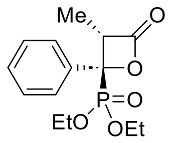

11

^{13}C, $CDCl_3$, 101 MHz

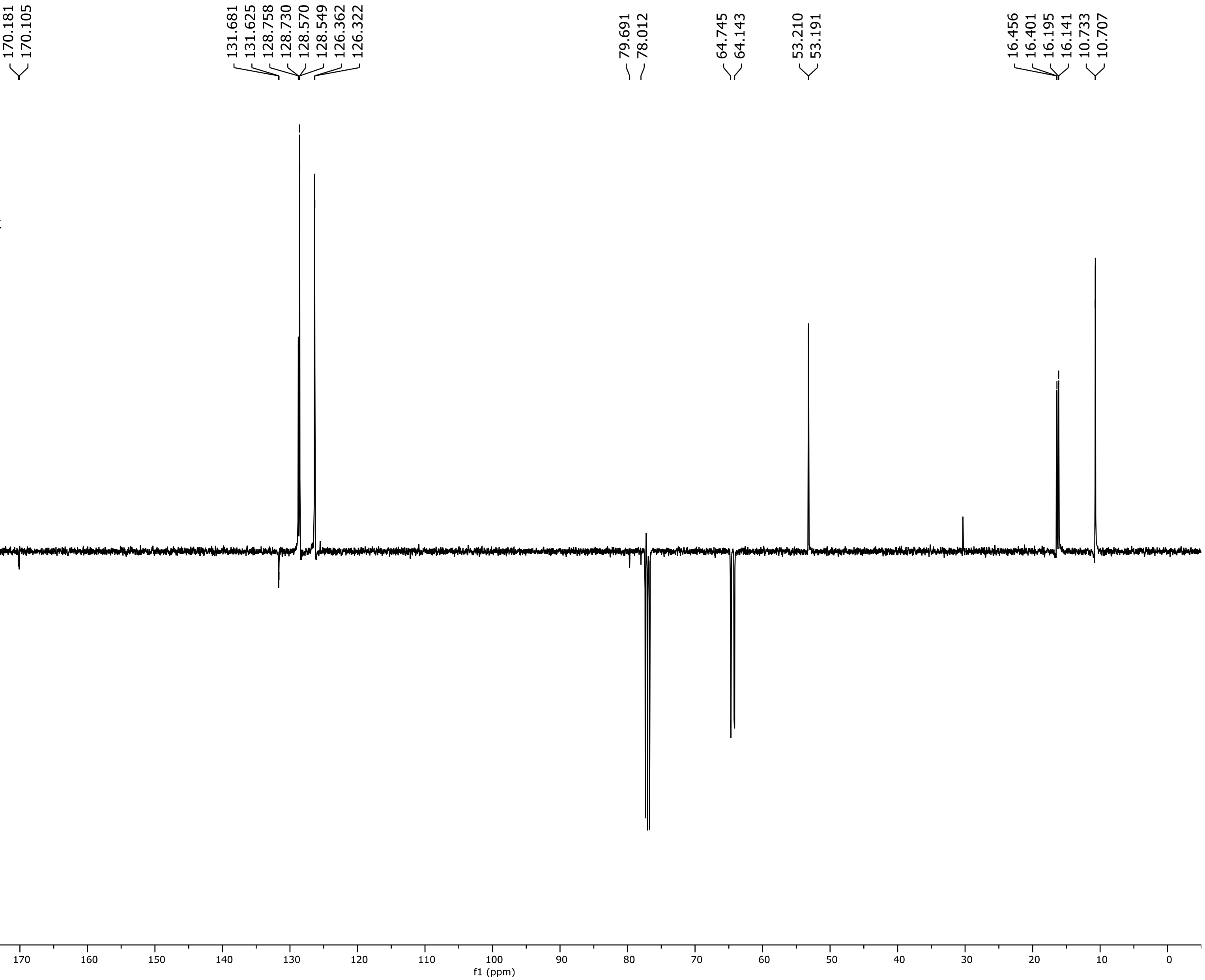

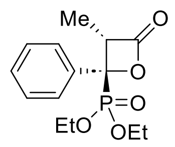

11

^{31}P, $CDCl_3$, 162 MHz

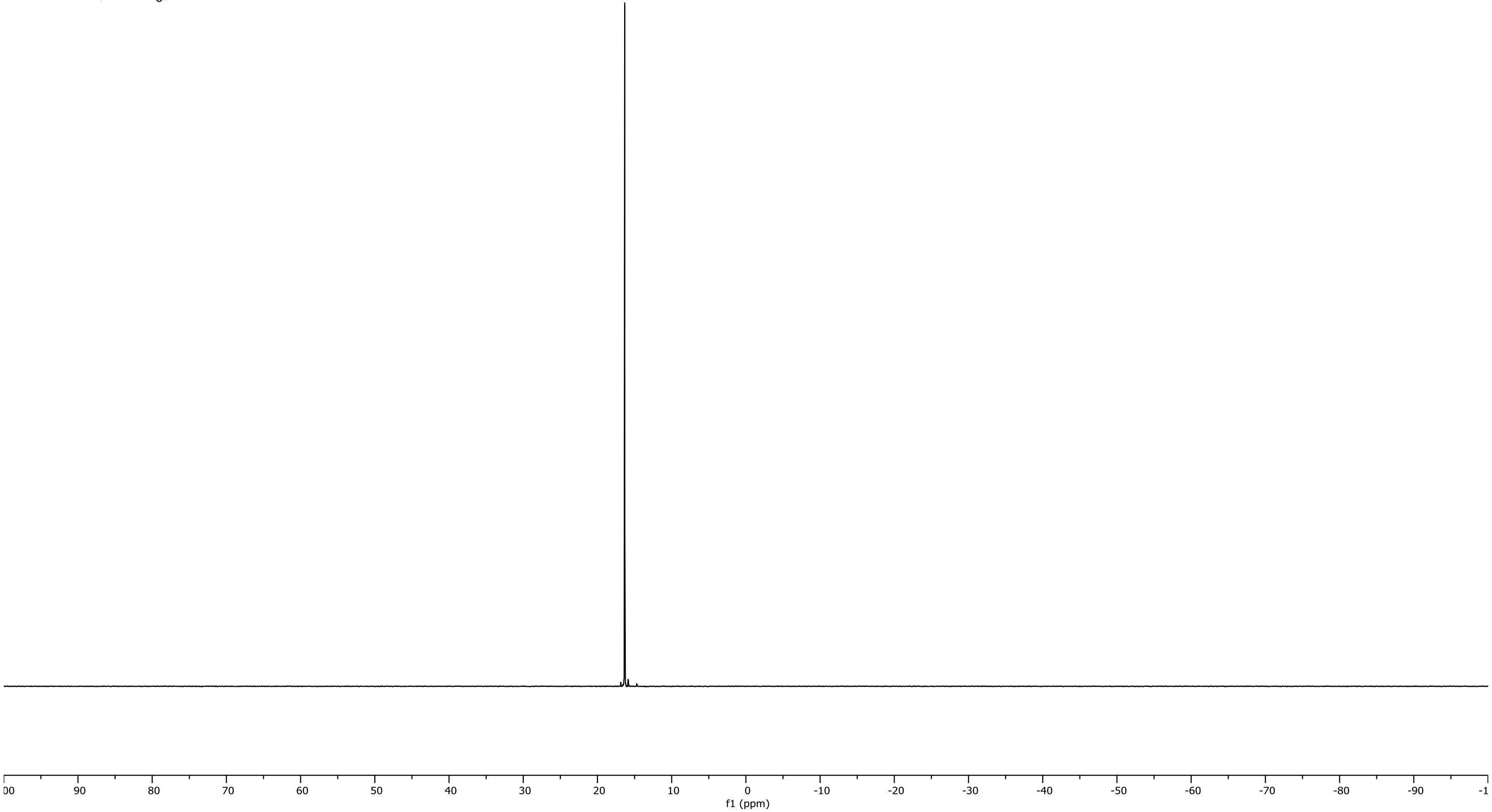

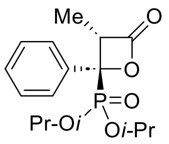

12

^{1}H, $CDCl_3$, 400 MHz

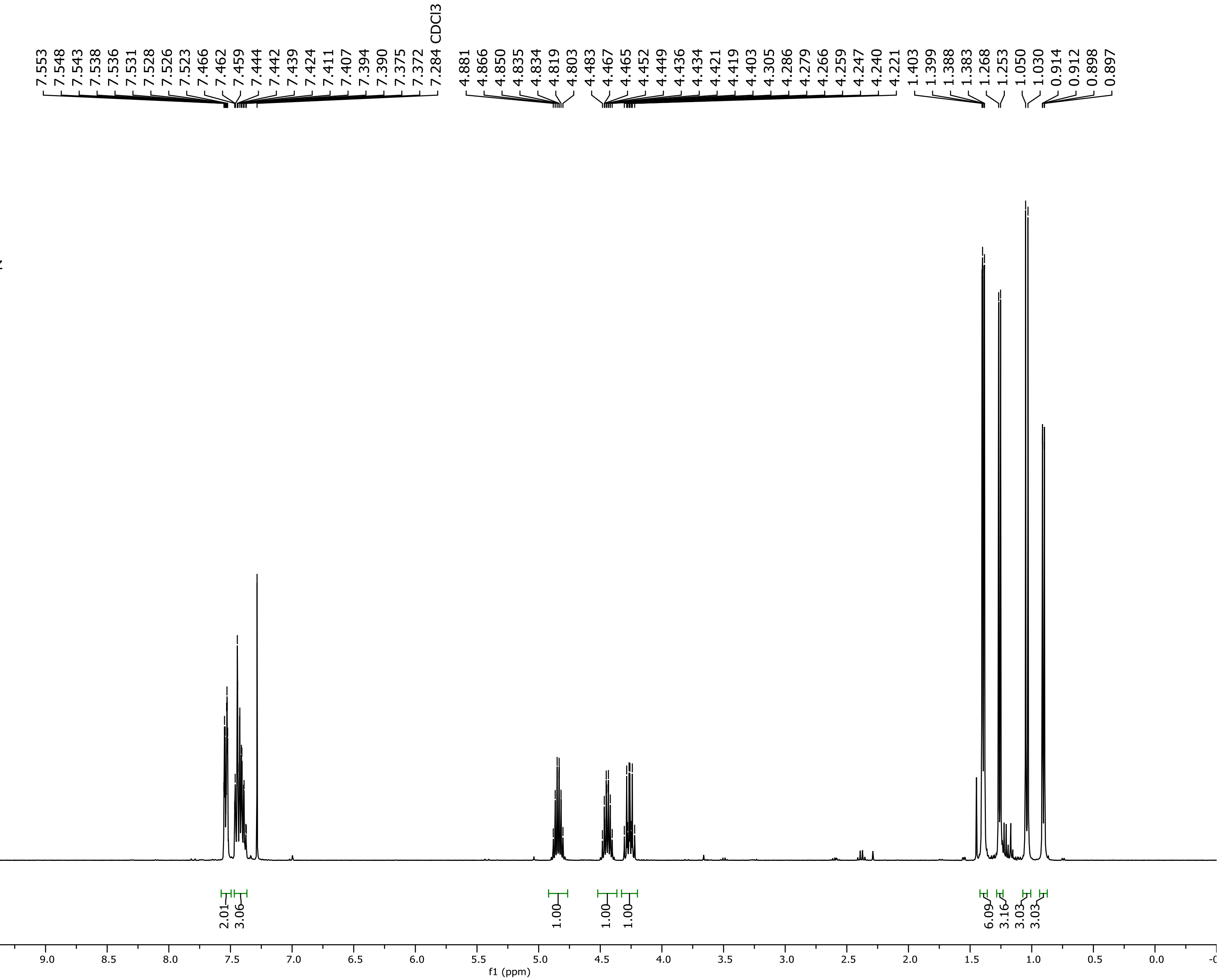

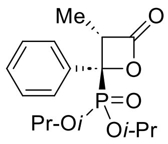

12

^{13}C, $CDCl_3$, 101 MHz

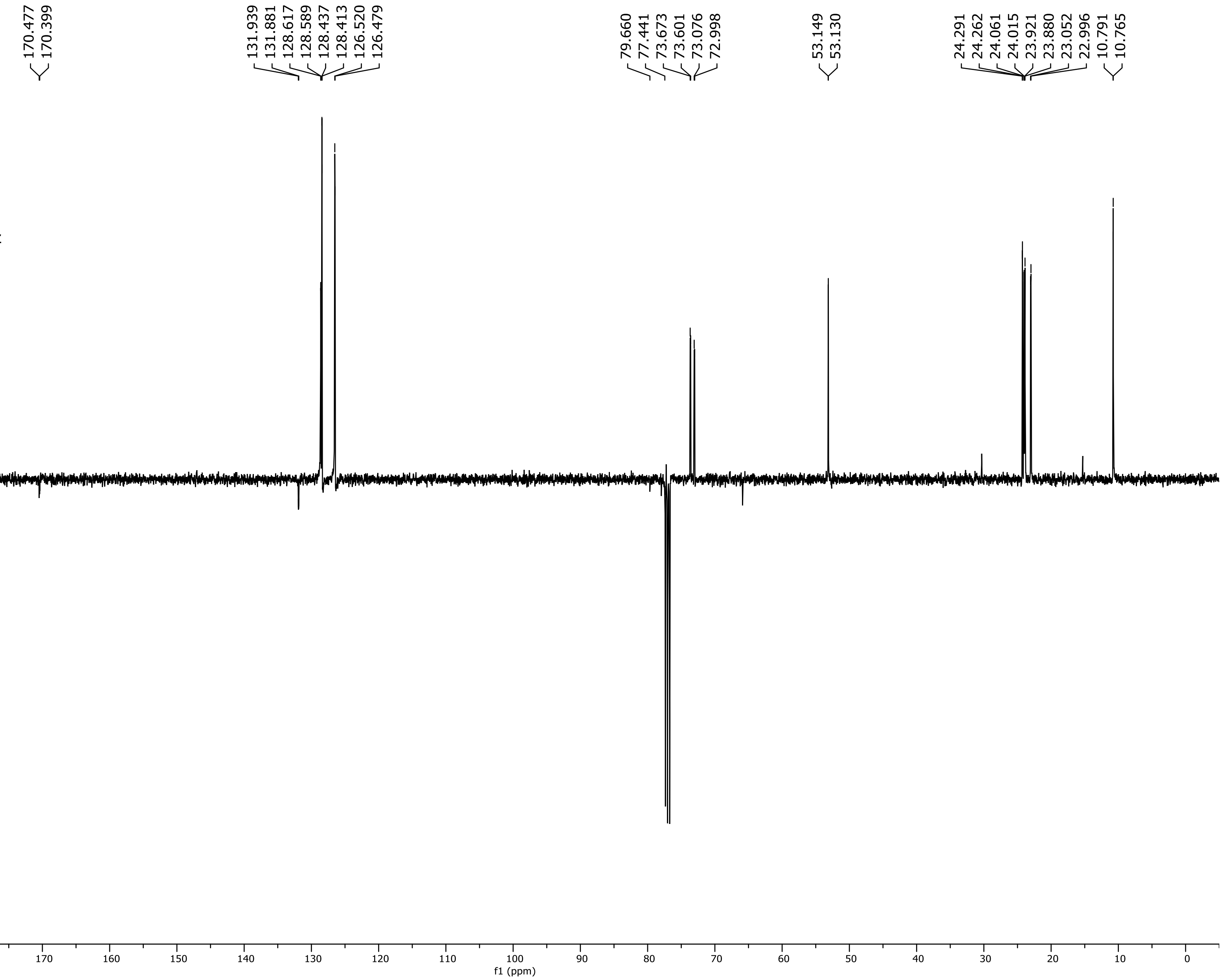

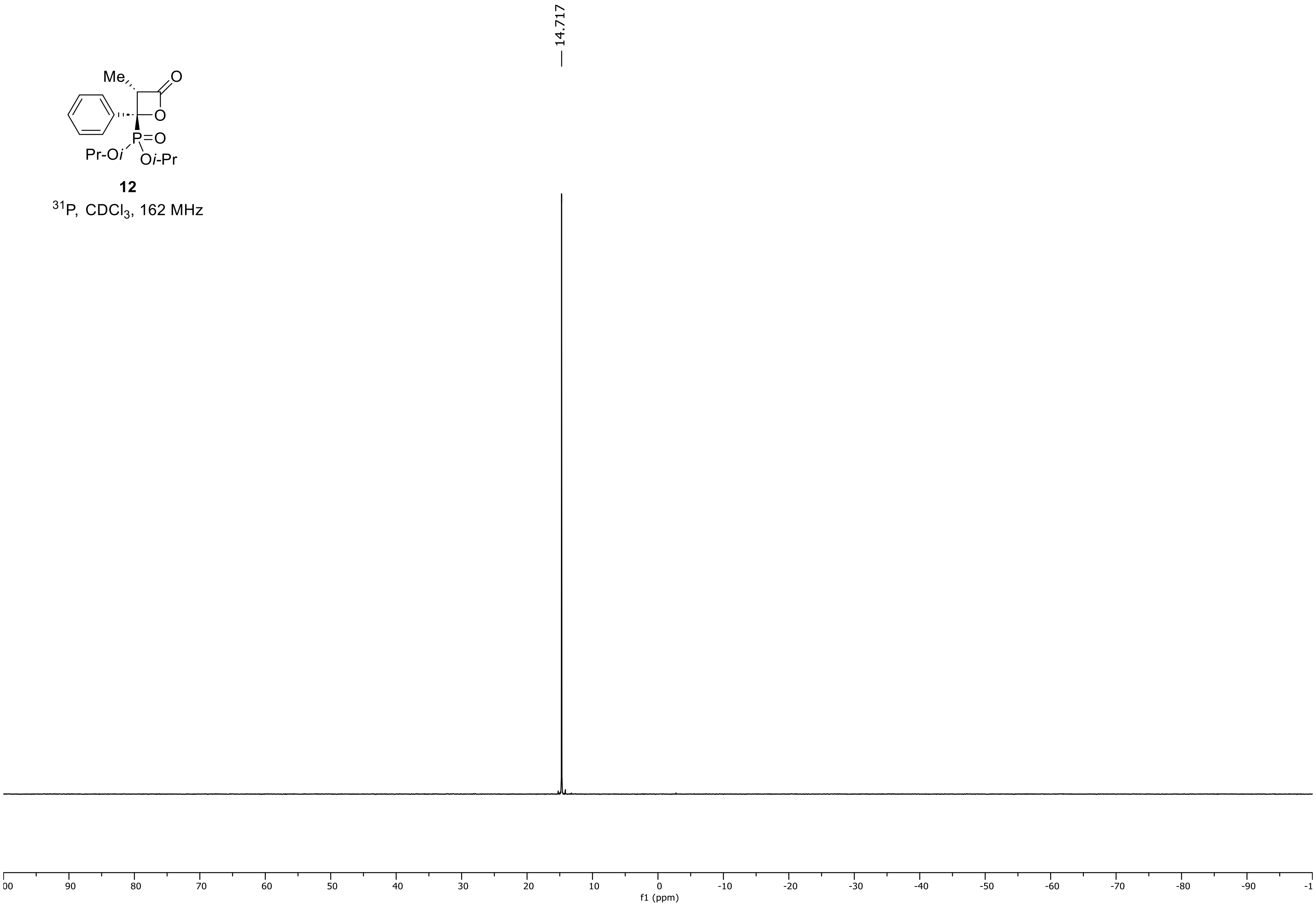

12

^{31}P, $CDCl_3$, 162 MHz

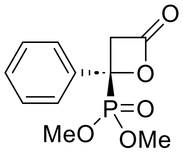

13

^{1}H, $CDCl_3$, 400 MHz

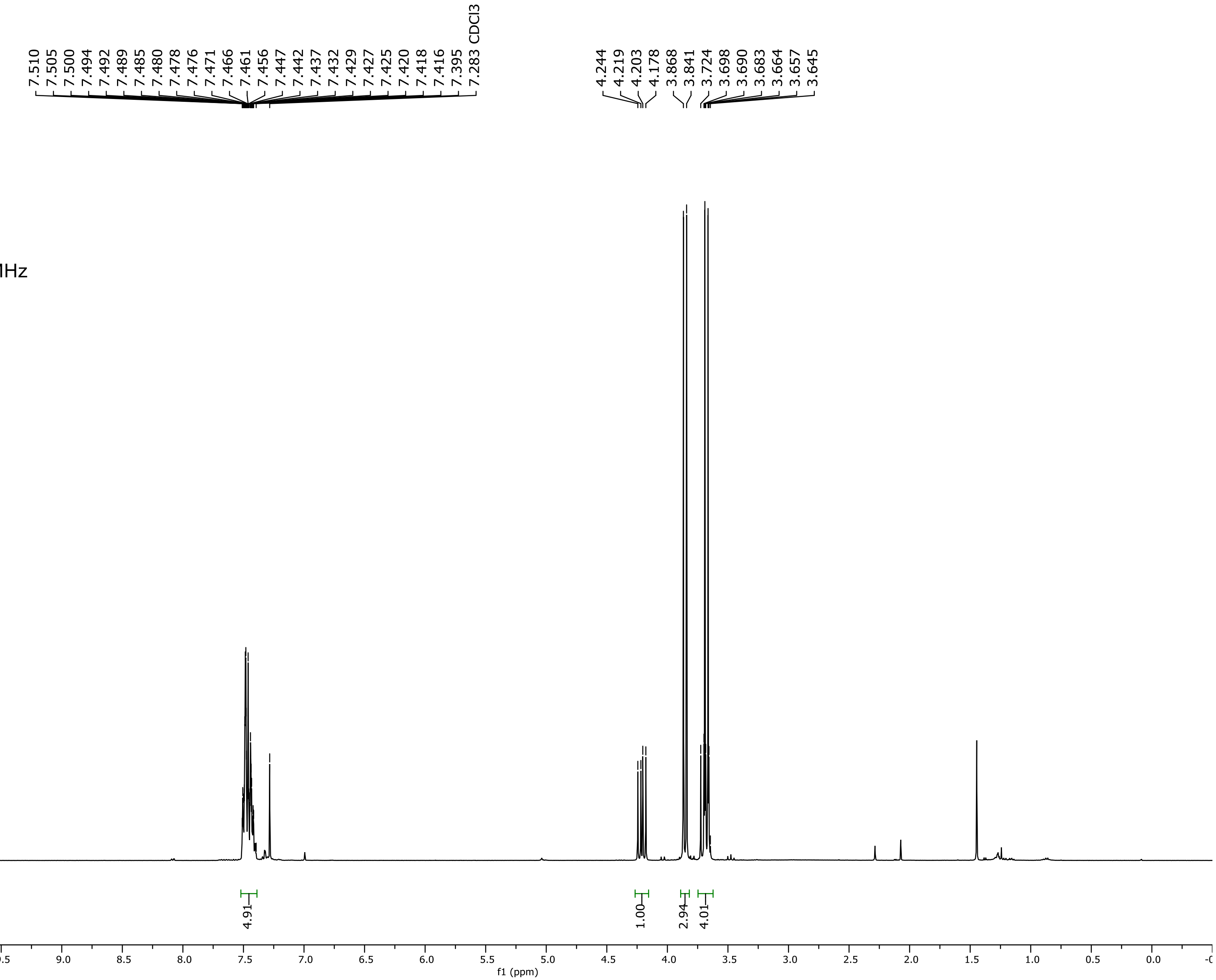

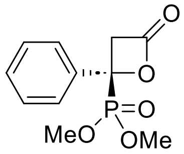

13

^{13}C, $CDCl_3$, 101 MHz

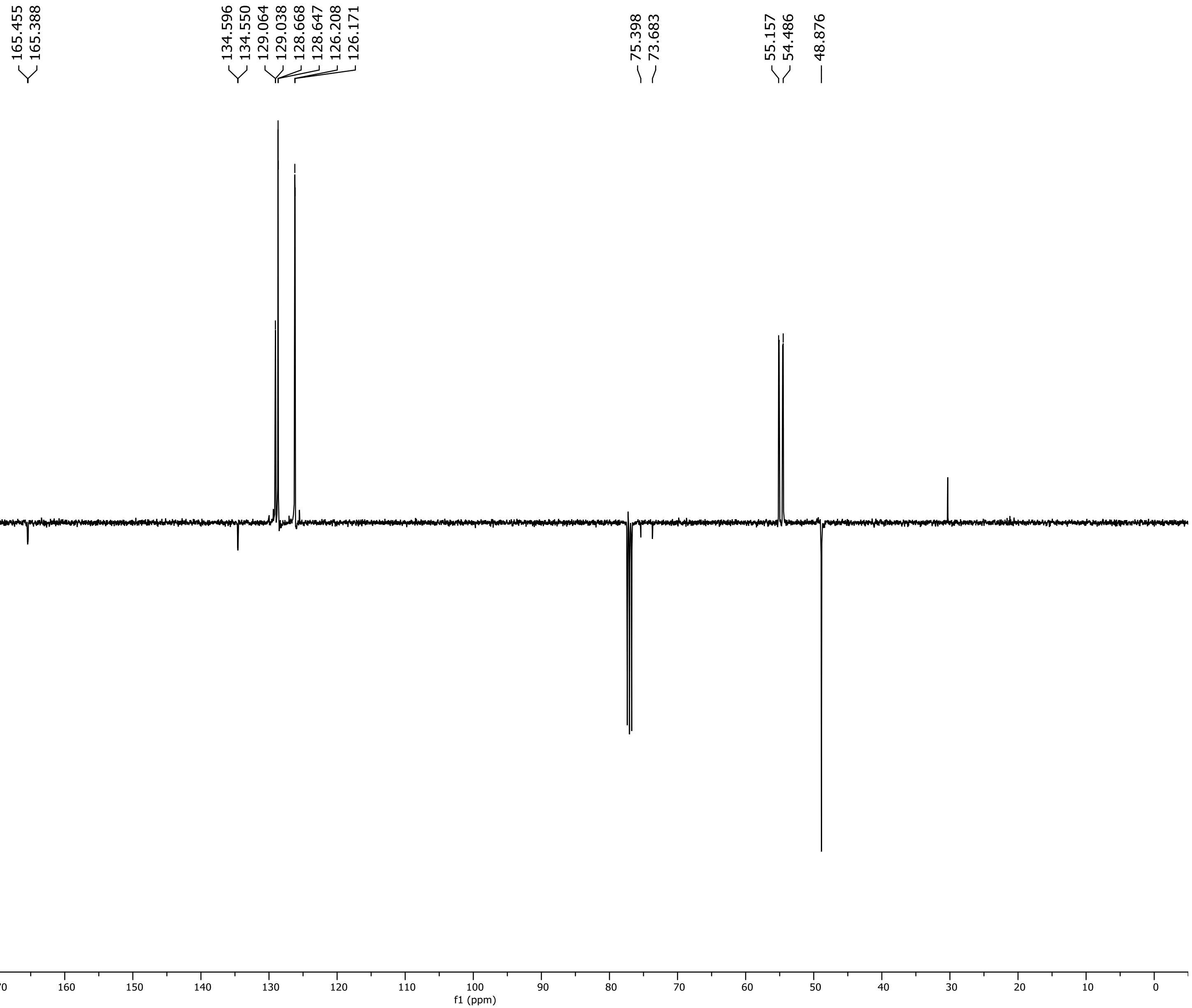

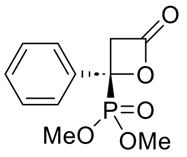

13

^{31}P, $CDCl_3$, 162 MHz

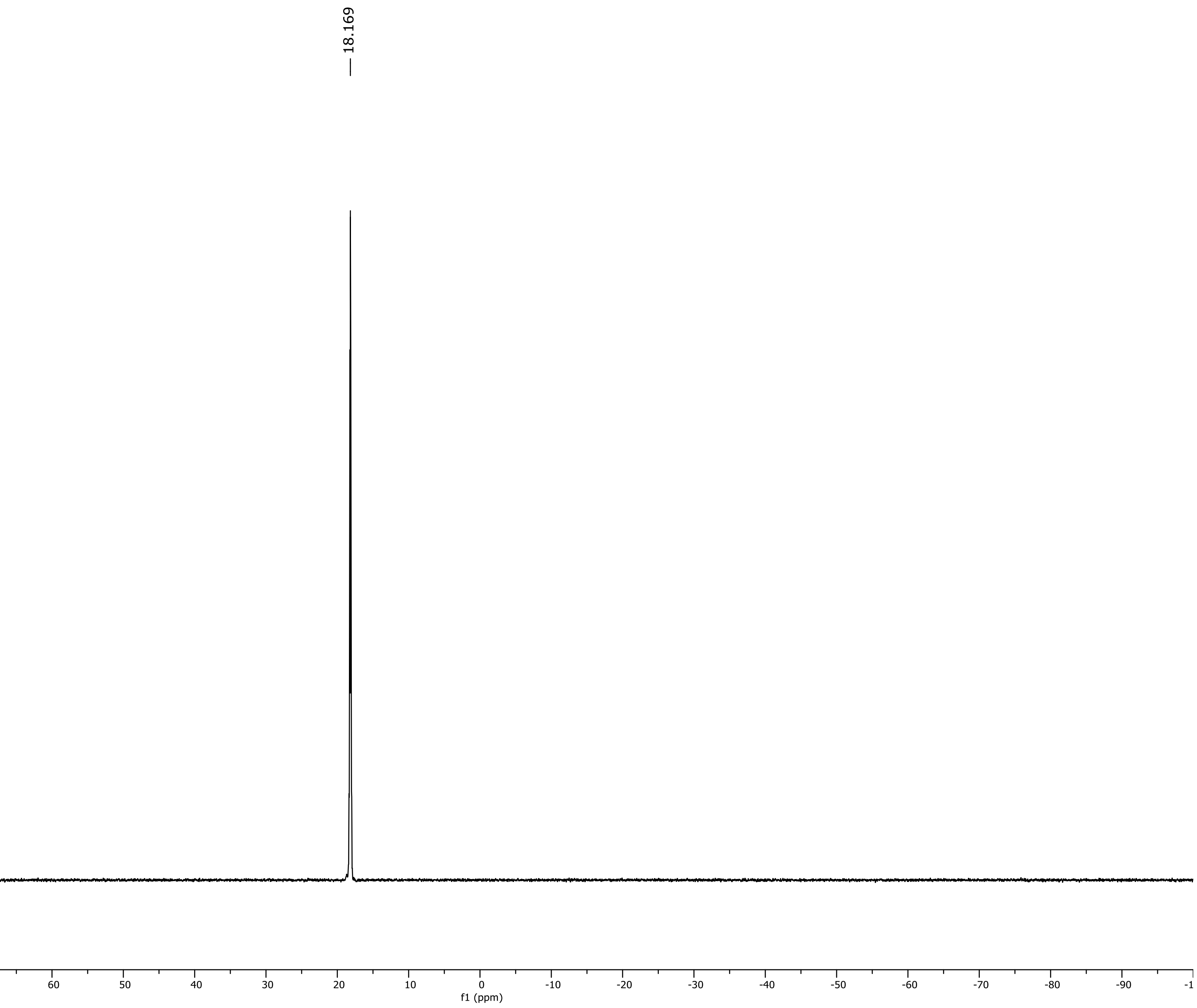

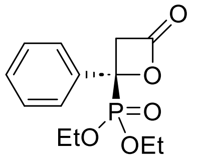

14

^1H, $CDCl_3$, 400 MHz

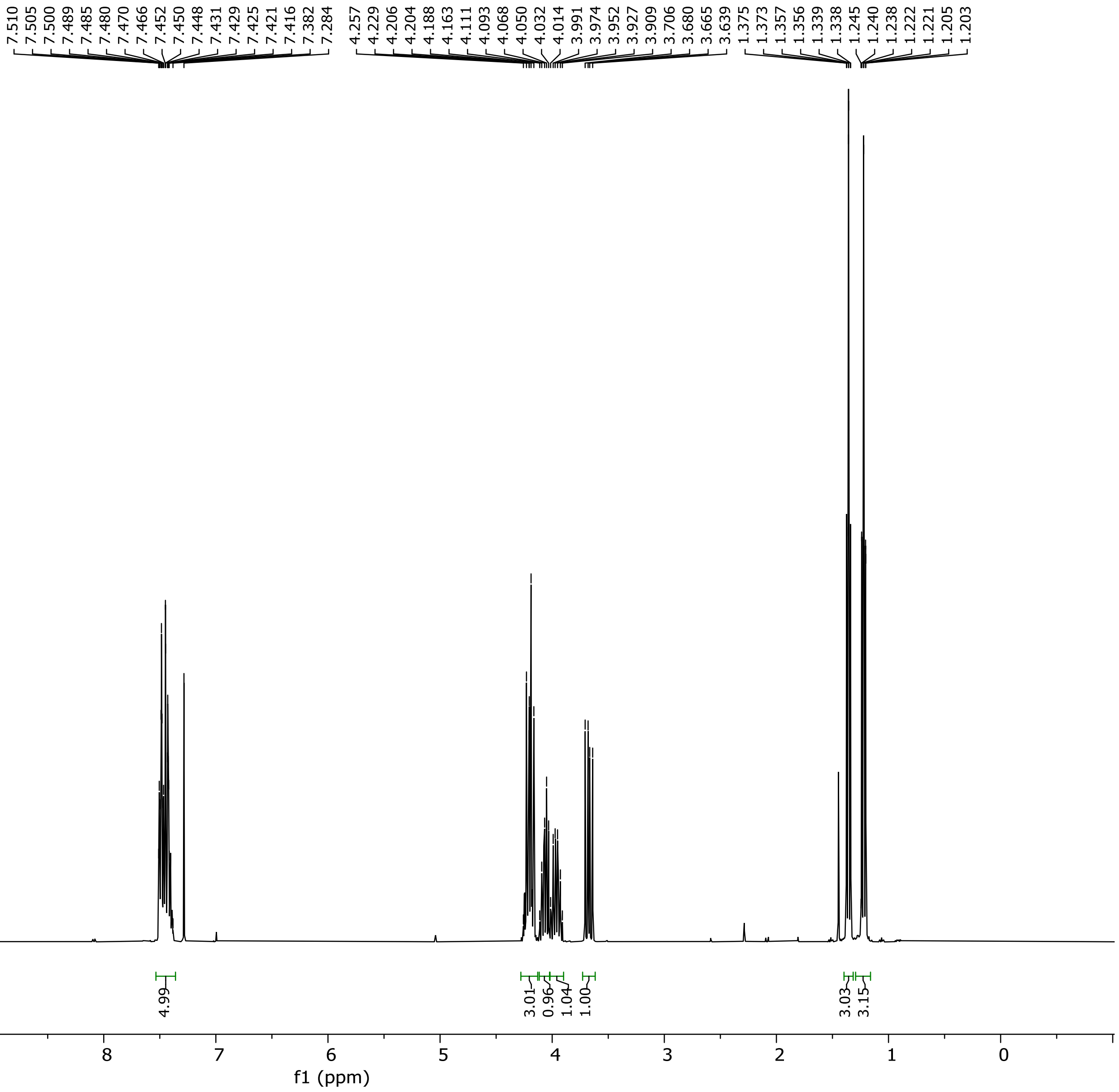

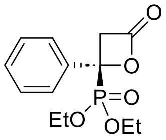

14

^{13}C, $CDCl_3$, 101 MHz

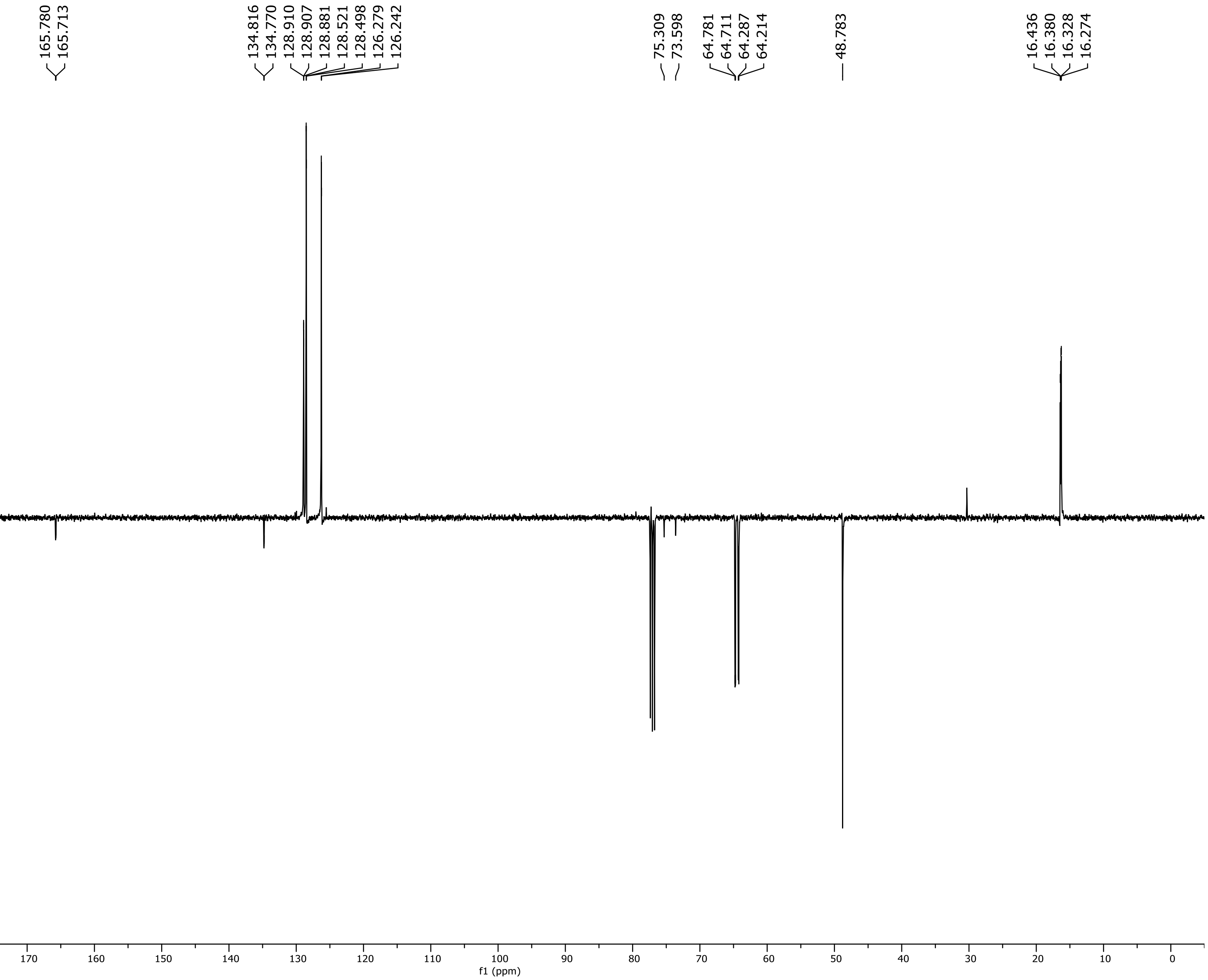

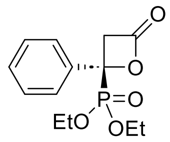

14

^{31}P, $CDCl_3$, 162 MHz

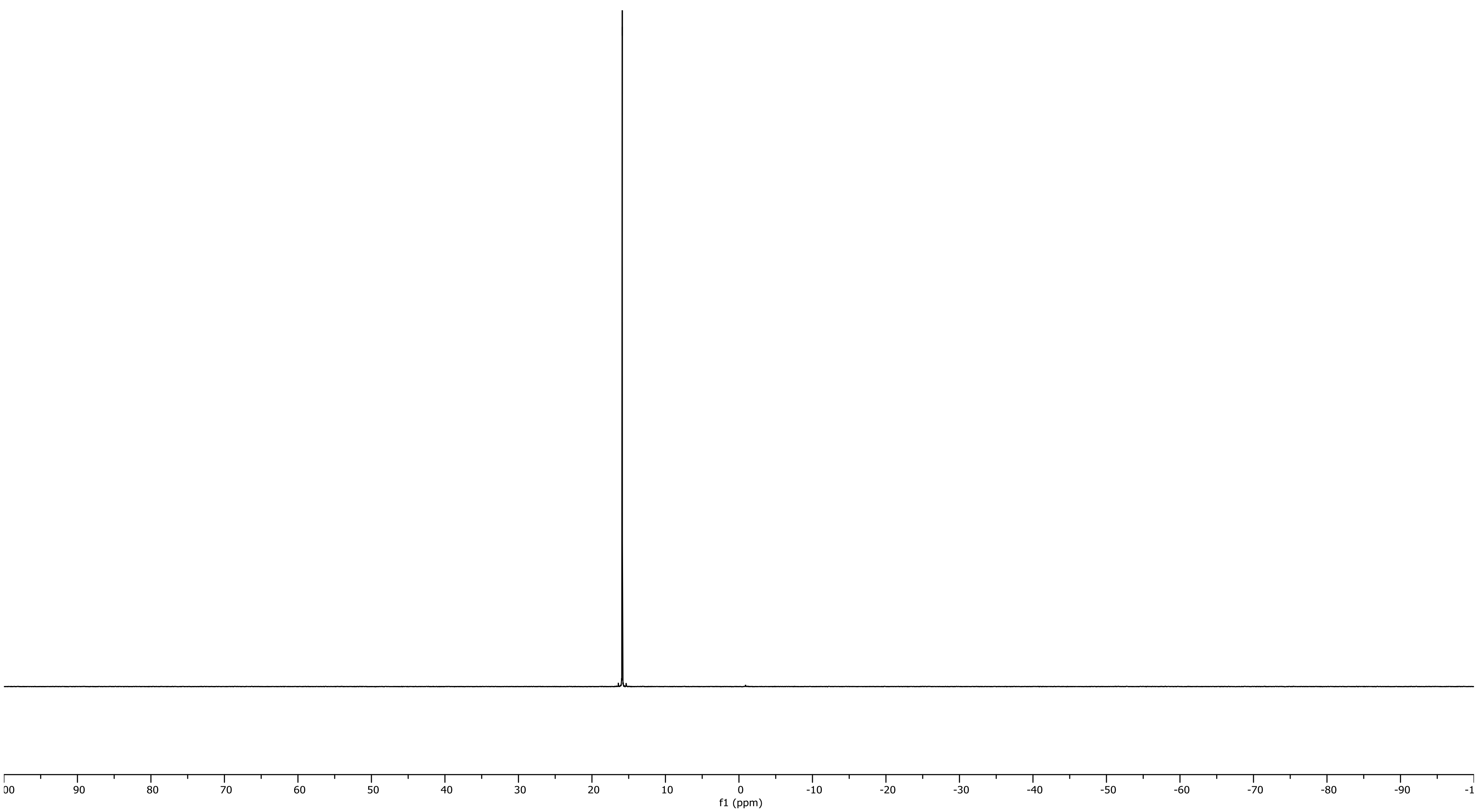

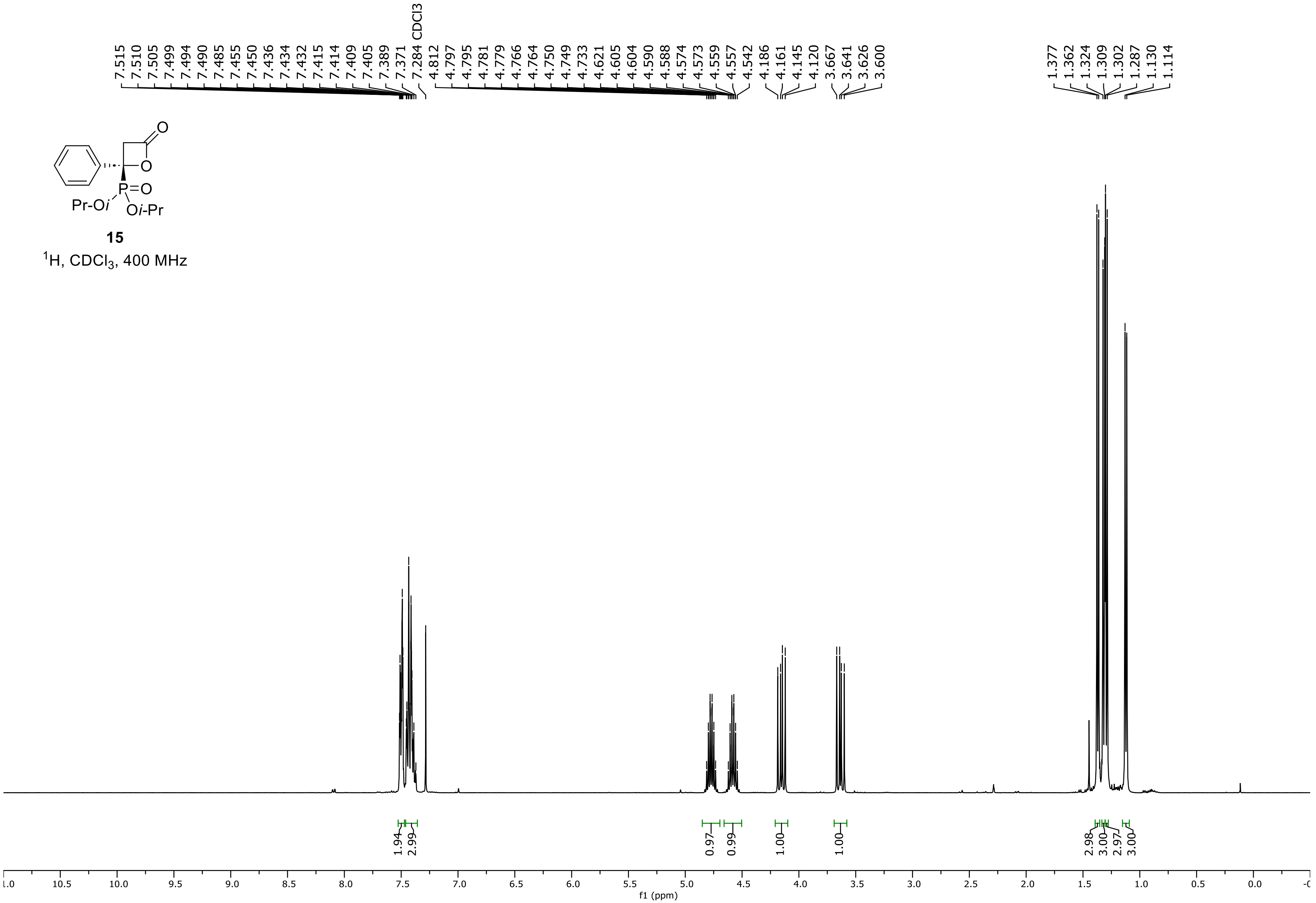

15

^{1}H, $CDCl_3$, 400 MHz

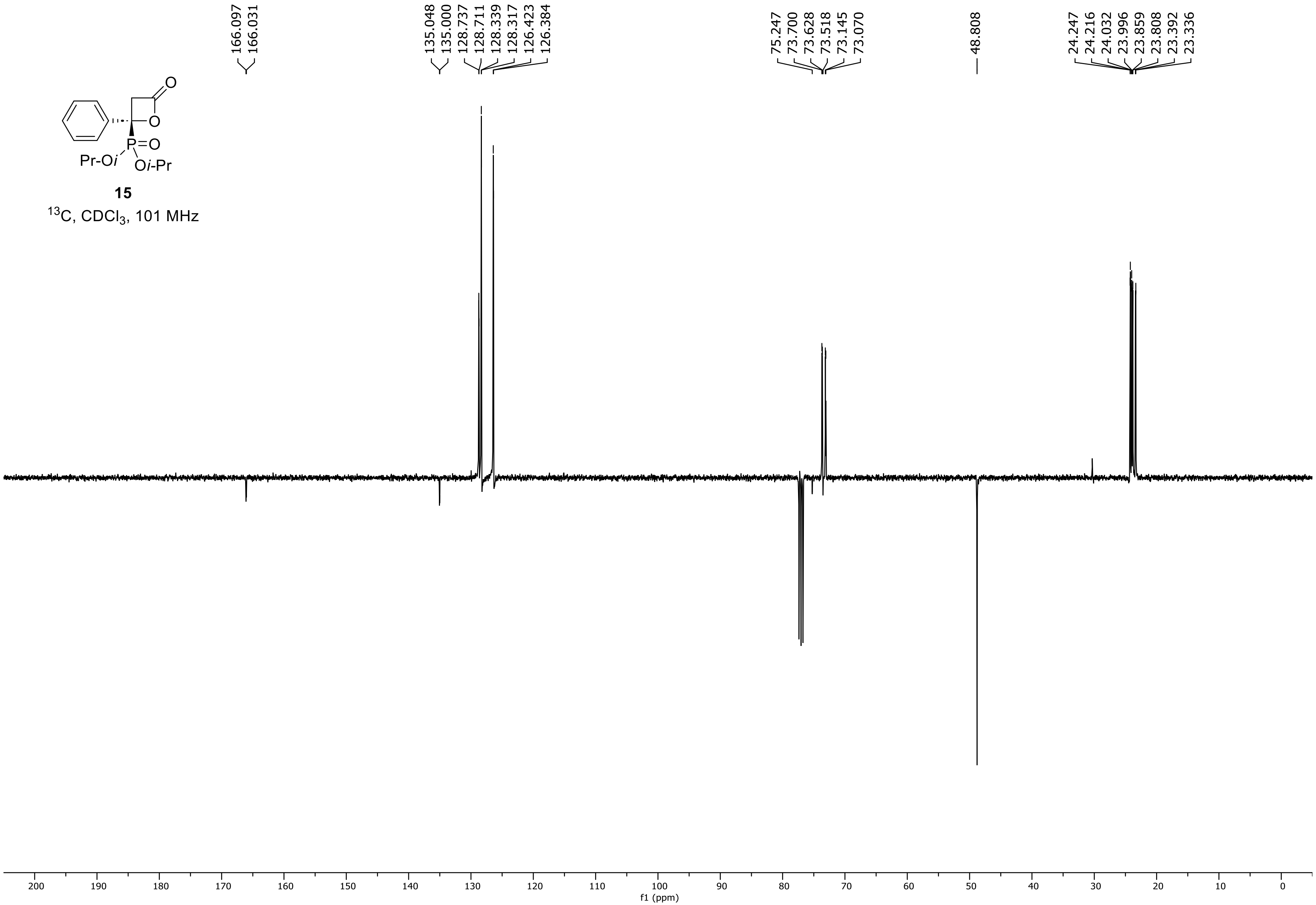
166.097
166.031
135.048
135.000
128.737
128.711
128.339
128.317
126.423
126.384
75.247
73.700
73.628
73.518
73.145
73.070
48.808
24.247
24.216
24.032
23.996
23.859
23.808
23.392
23.336
O
O
P=O
Pr-O*i*
O*i*-Pr
15
^{13}C, $CDCl_3$, 101 MHz
200 190 180 170 160 150 140 130 120 110 100 90 80 70 60 50 40 30 20 10 0
f1 (ppm)

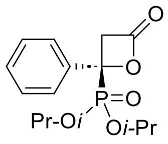

15

^{31}P, $CDCl_3$, 162 MHz

14.149

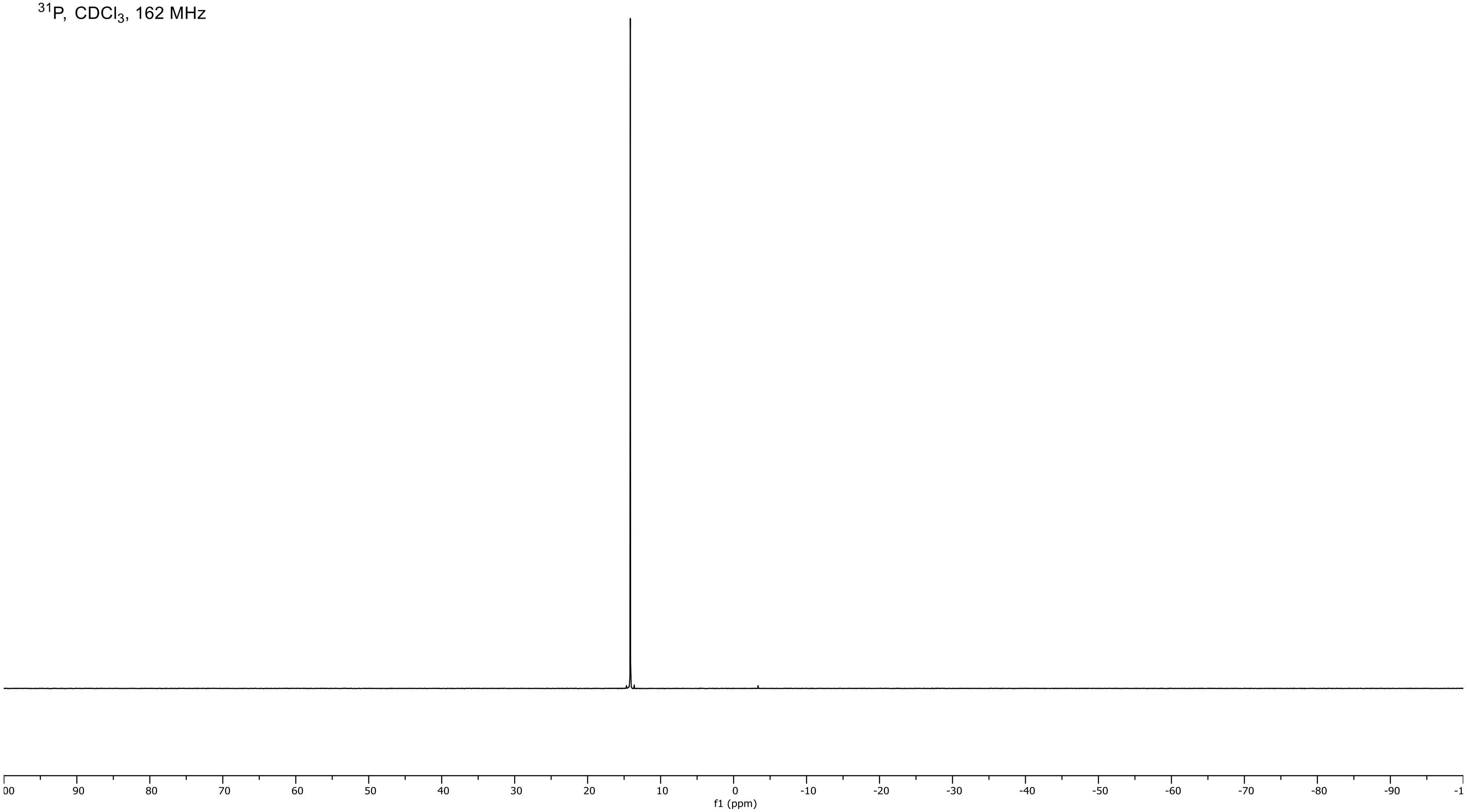

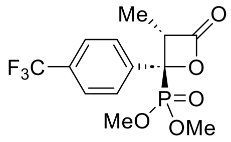

16

^{1}H, $CDCl_3$, 400 MHz

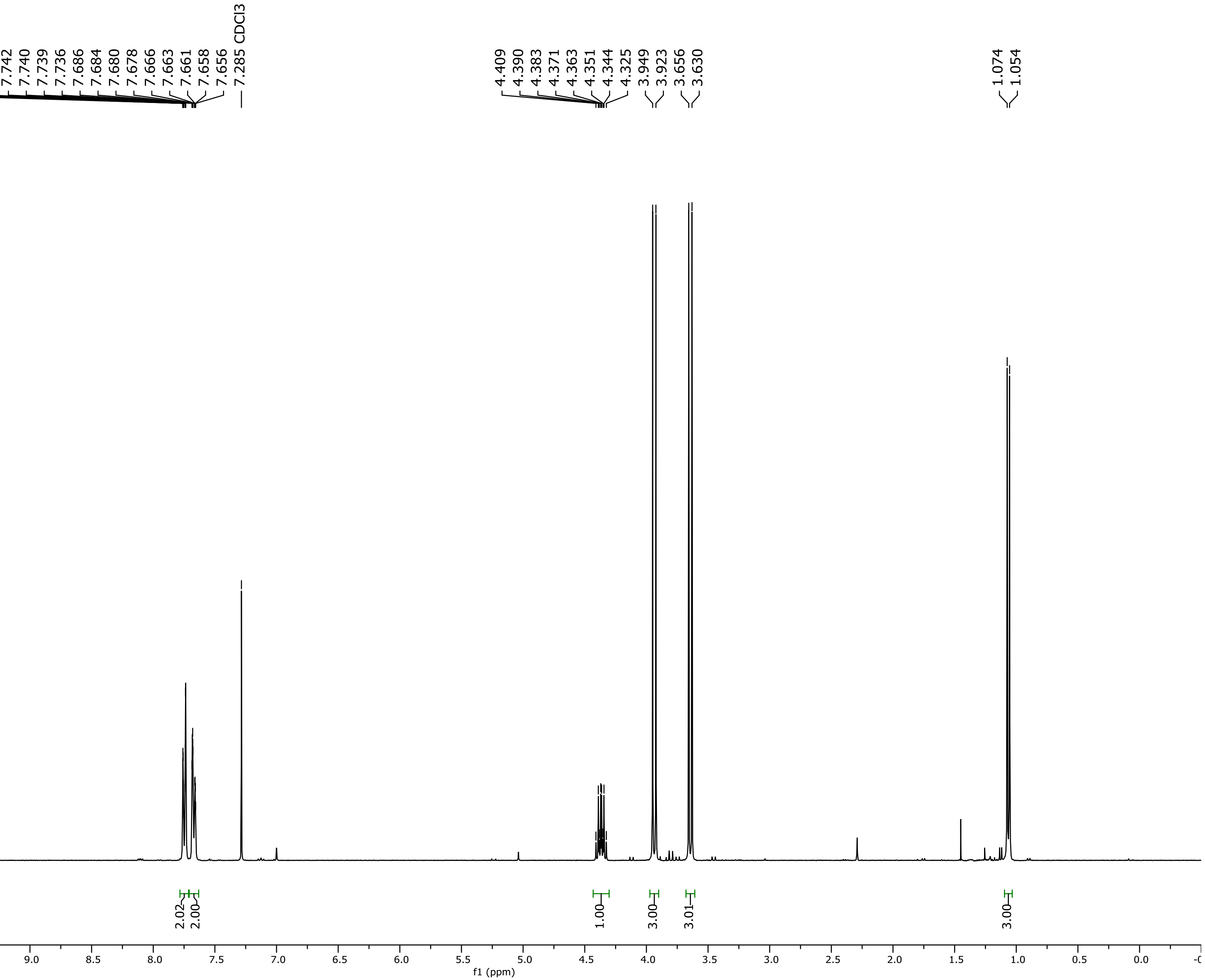

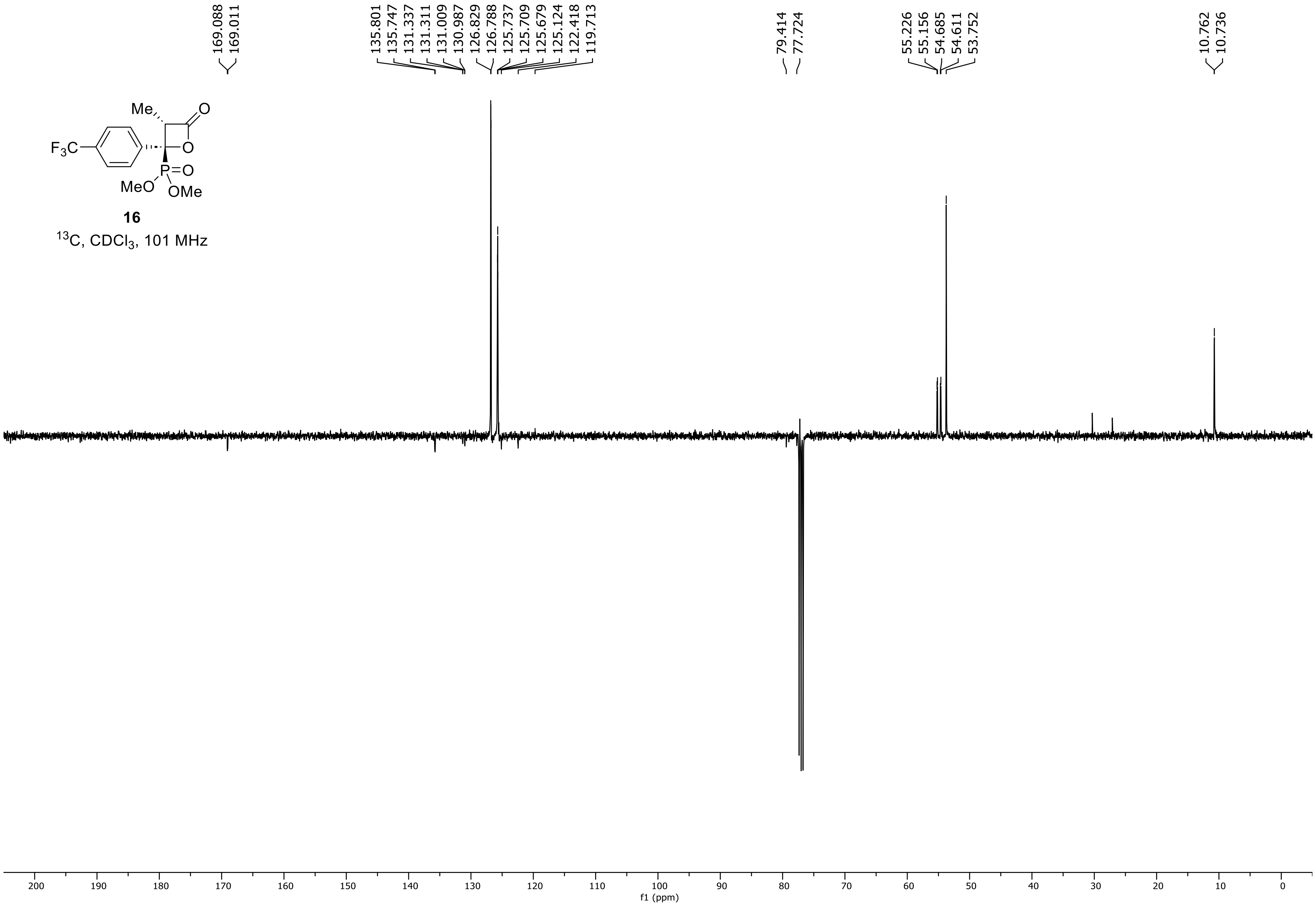

16

^{13}C, $CDCl_3$, 101 MHz

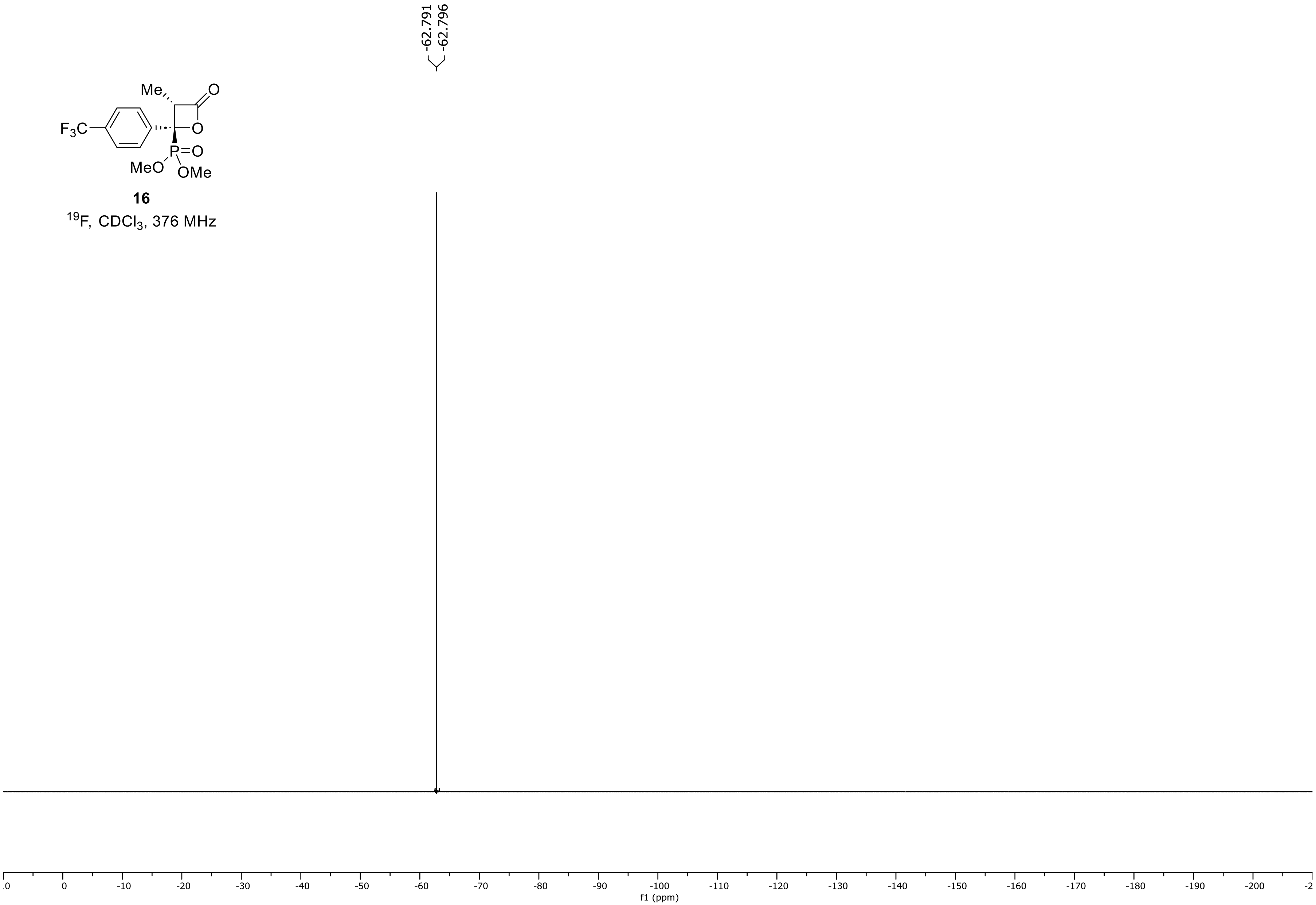

16

^{19}F, $CDCl_3$, 376 MHz

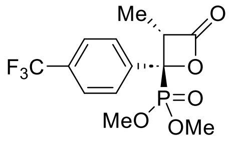

16

^{31}P, $CDCl_3$, 162 MHz

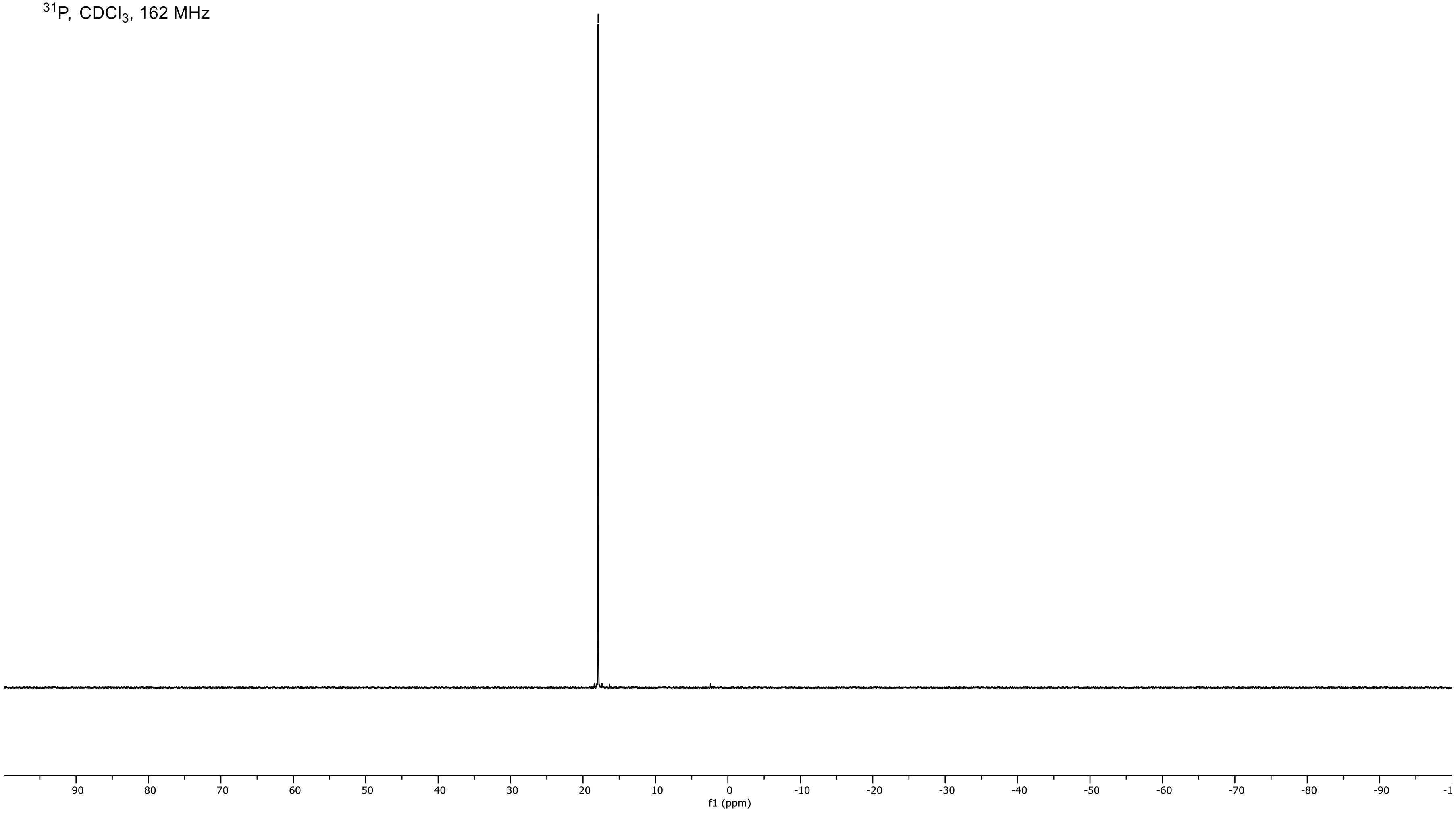

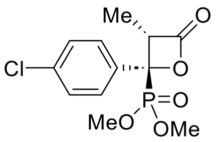

17

^1H, $CDCl_3$, 400 MHz

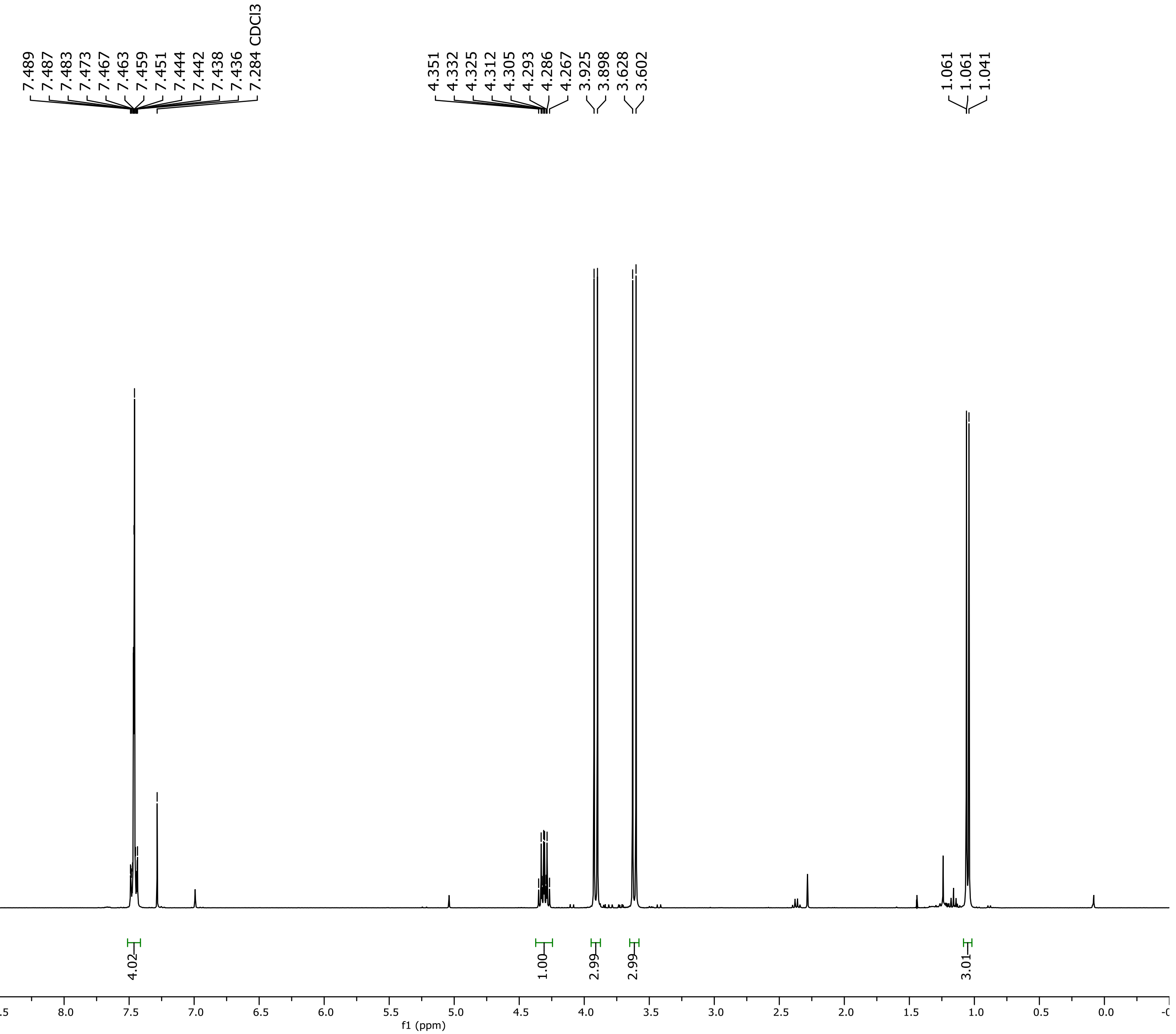

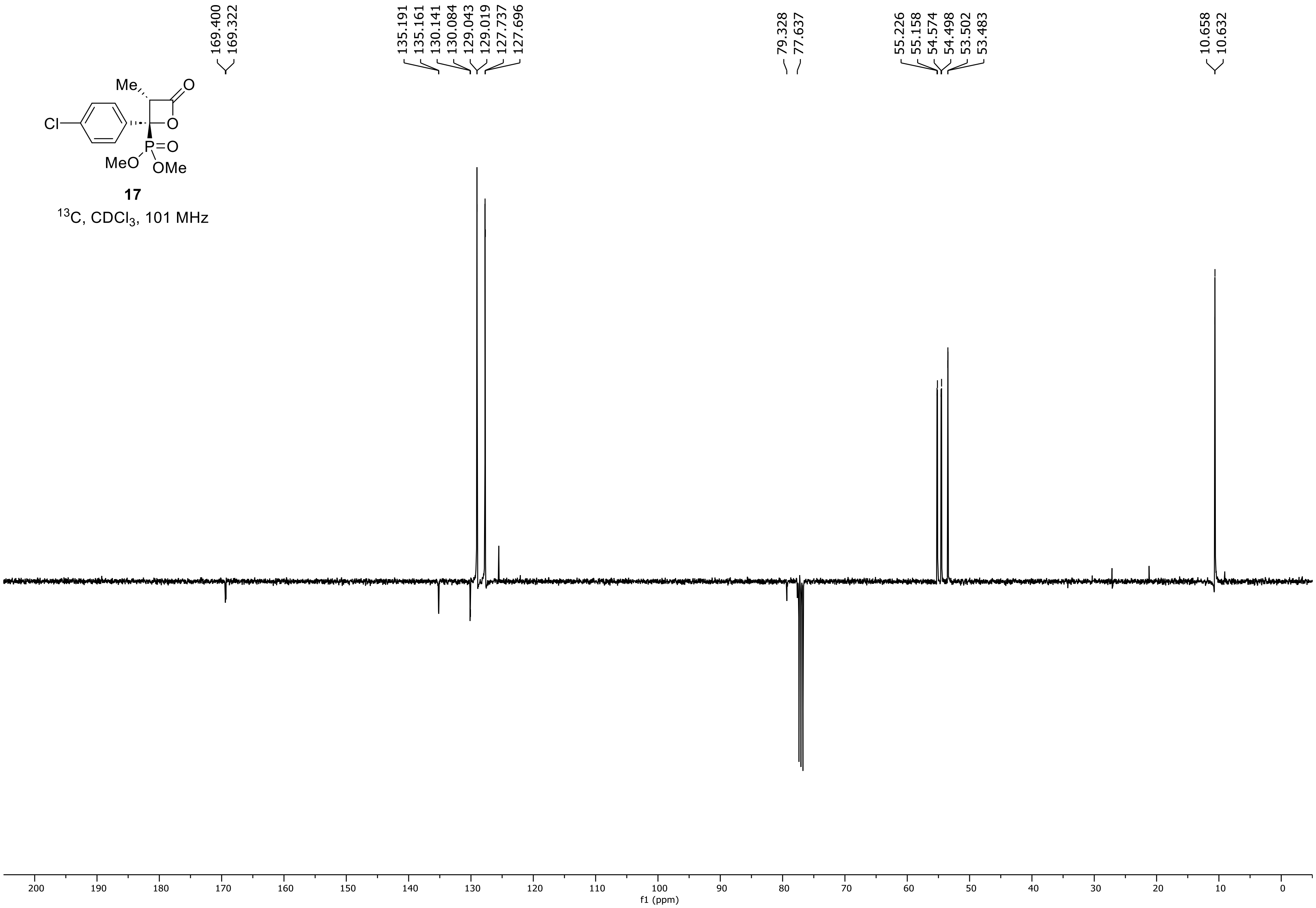
169.400
169.322
135.191
135.161
130.141
130.084
129.043
129.019
127.737
127.696
79.328
77.637
55.226
55.158
54.574
54.498
53.502
53.483
10.658
10.632
Me
O
Cl
O
P=O
MeO
OMe
17
13C, CDCl3, 101 MHz
200
190
180
170
160
150
140
130
120
110
100
90
80
70
60
50
40
30
20
10
0
f1 (ppm)

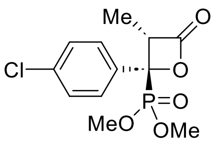

17

^{31}P, $CDCl_3$, 162 MHz

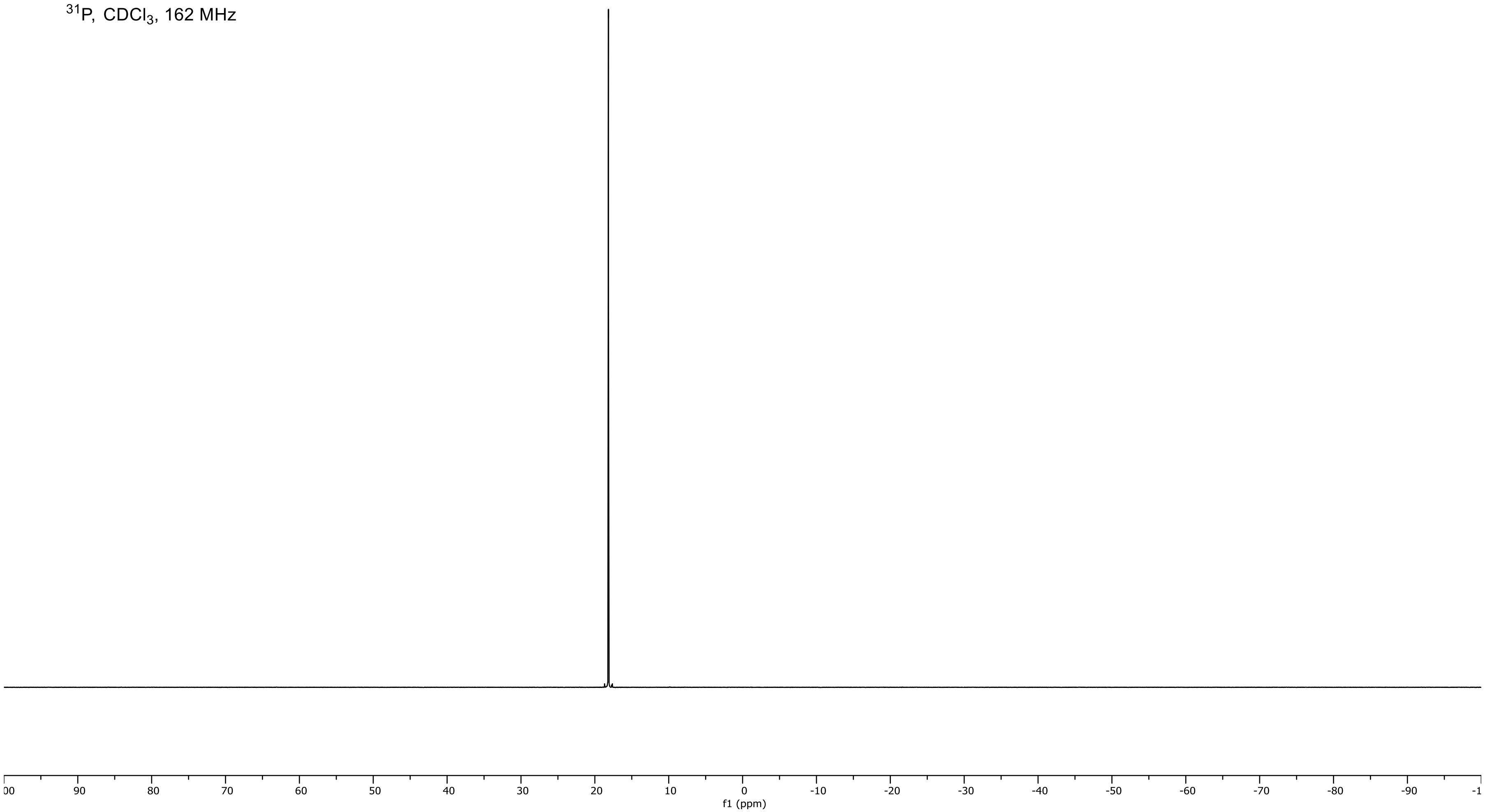

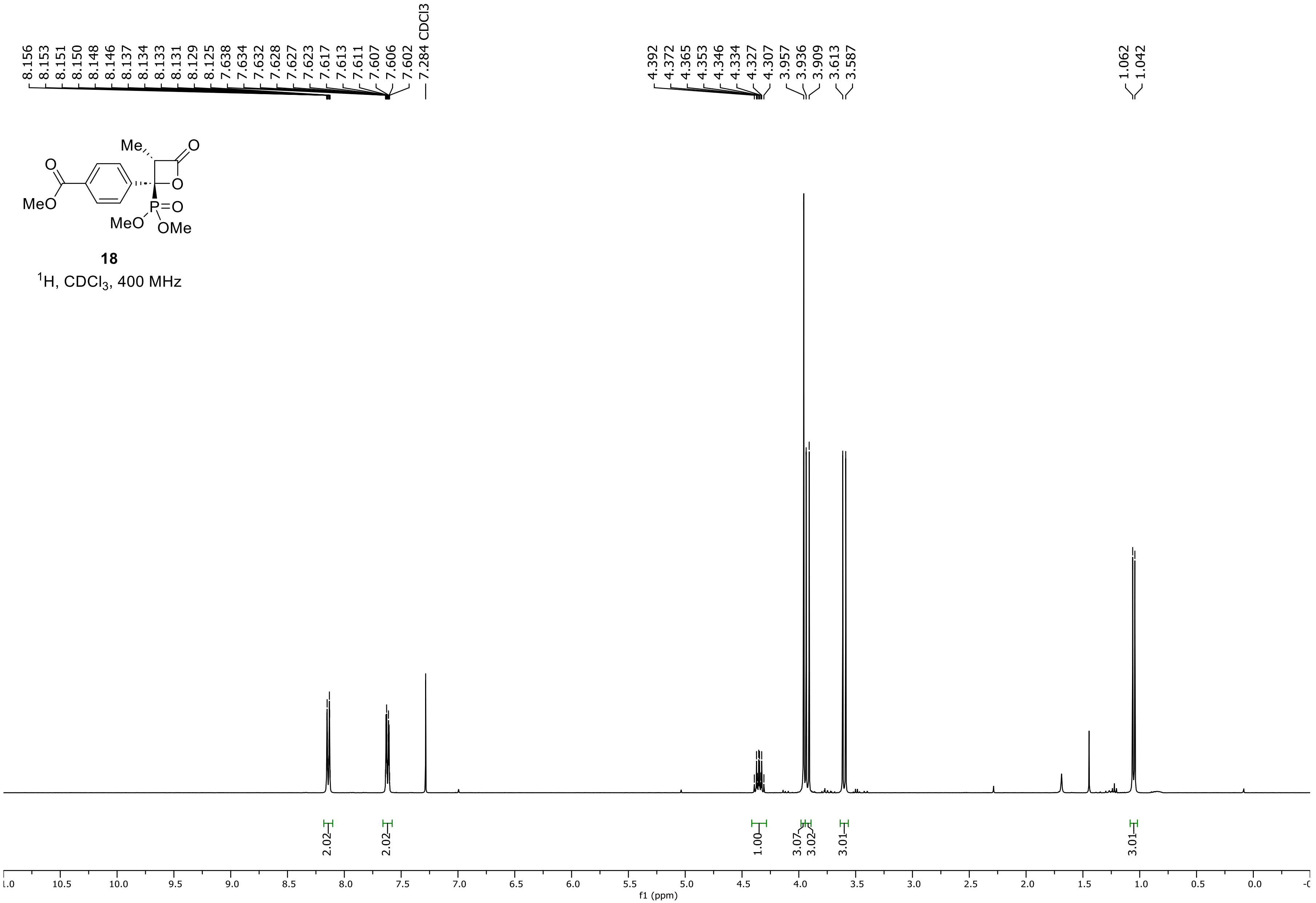

18

^{1}H, $CDCl_3$, 400 MHz

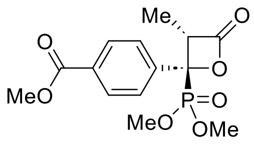

18

^{13}C, $CDCl_3$, 101 MHz

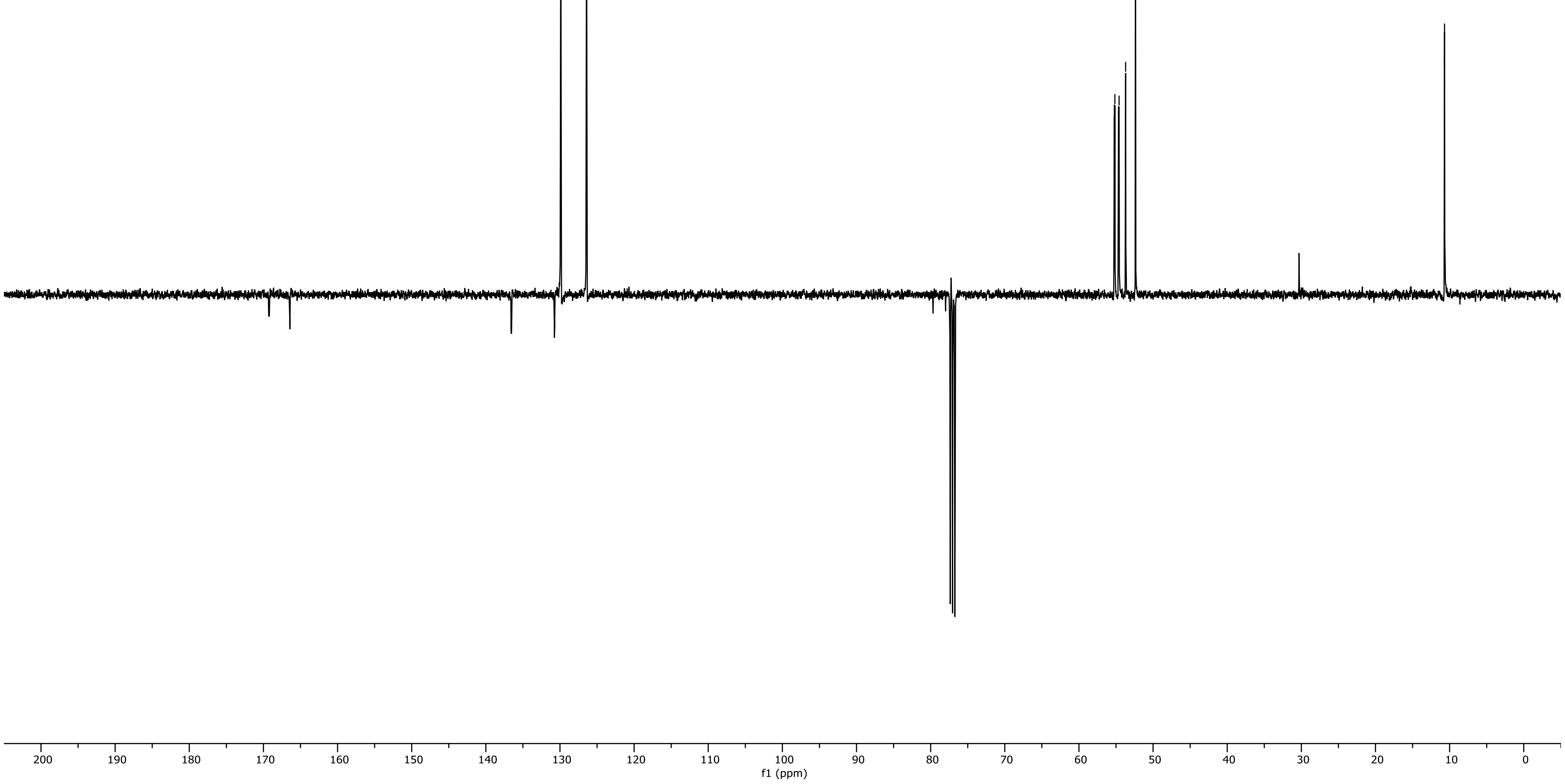

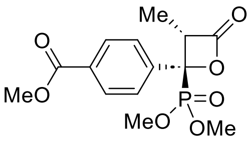

18

^{31}P, $CDCl_3$, 162 MHz

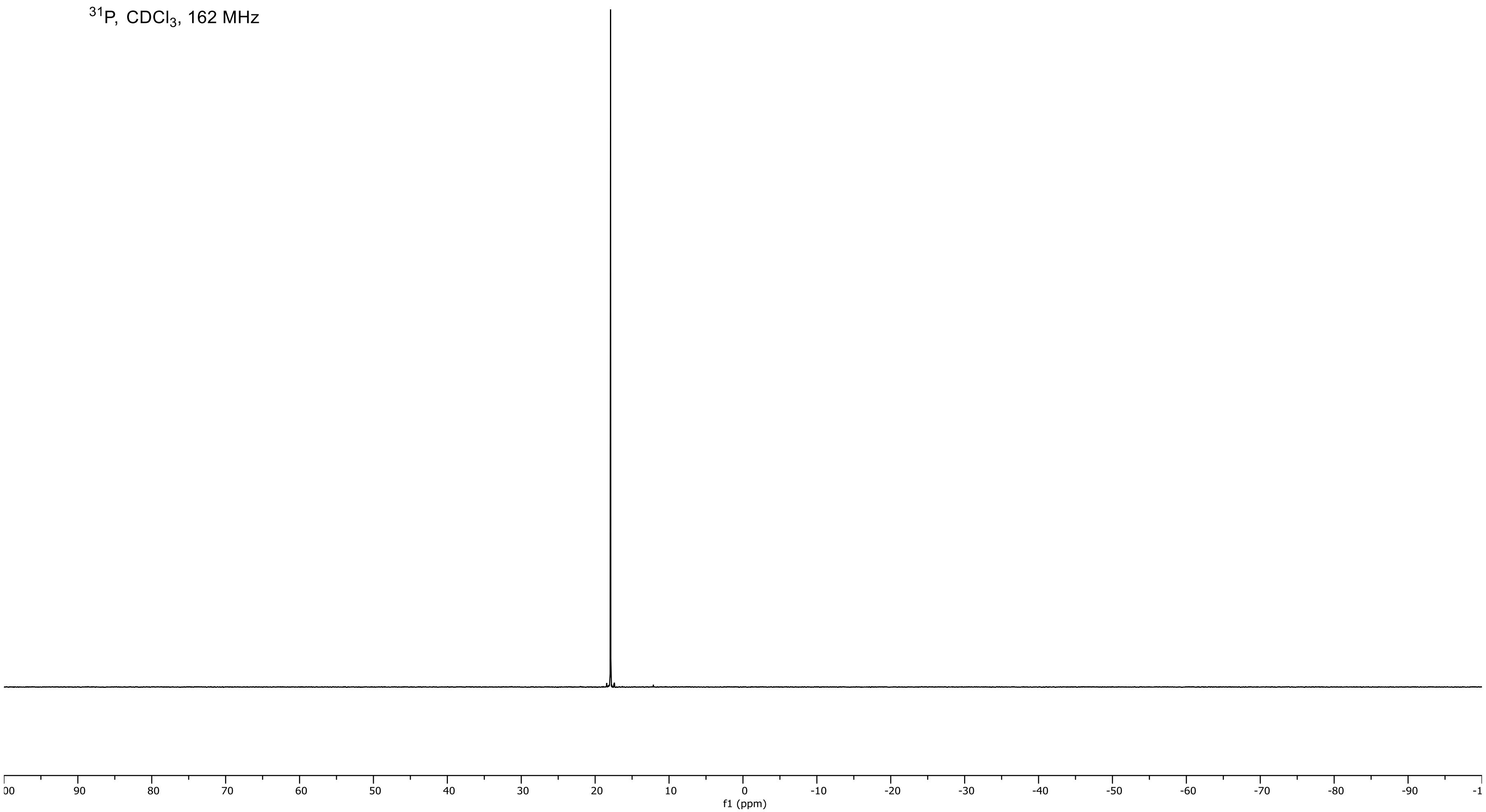

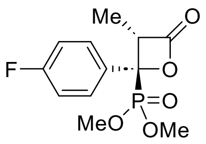

19

^{1}H, $CDCl_3$, 400 MHz

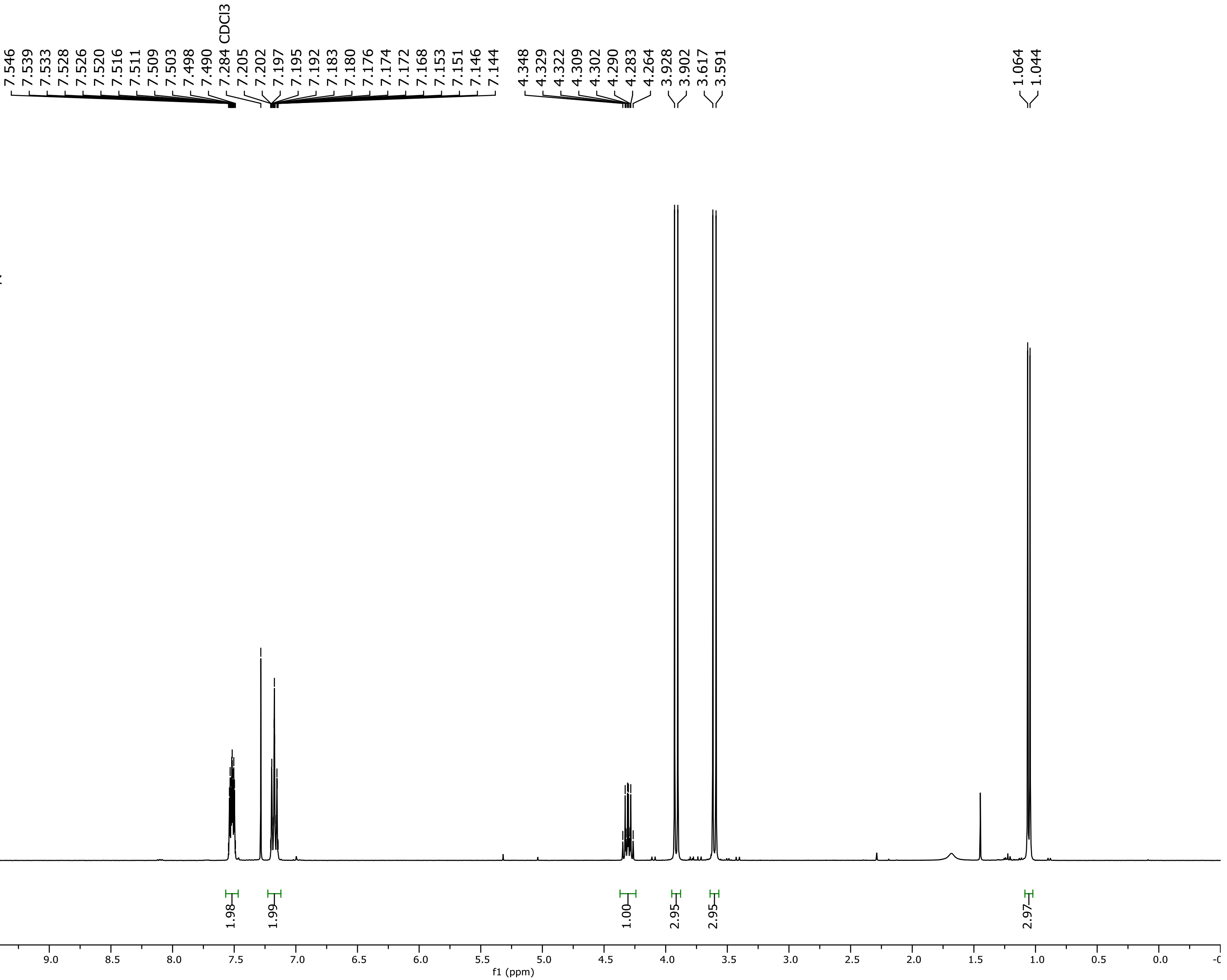

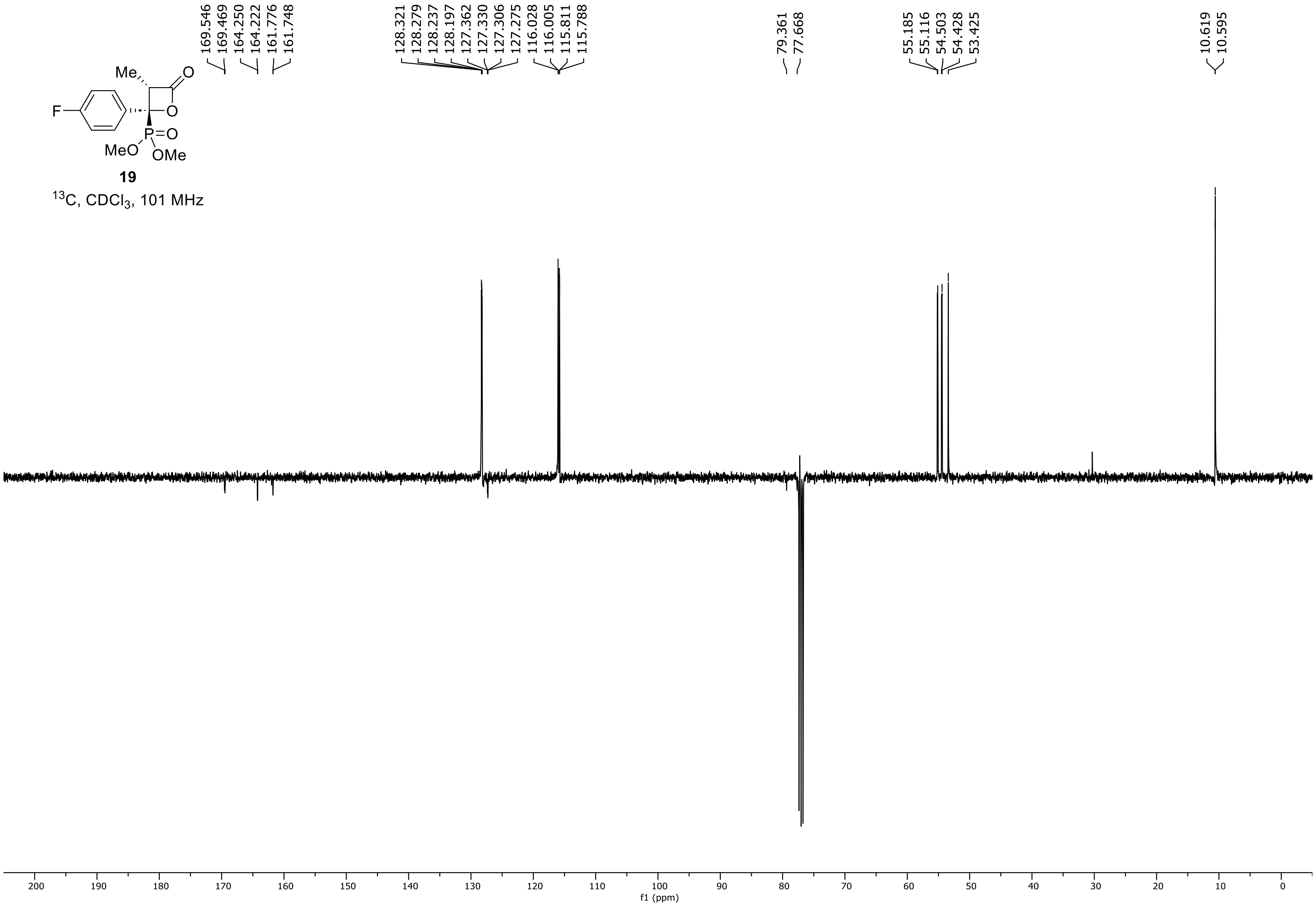
169.546
169.469
164.250
164.222
161.776
161.748
128.321
128.279
128.237
128.197
127.362
127.330
127.306
127.275
116.028
116.005
115.811
115.788
79.361
77.668
55.185
55.116
54.503
54.428
53.425
10.619
10.595
Me
O
F
O
P=O
MeO
OMe
19
13C, CDCl3, 101 MHz
200 190 180 170 160 150 140 130 120 110 100 90 80 70 60 50 40 30 20 10 0
f1 (ppm)

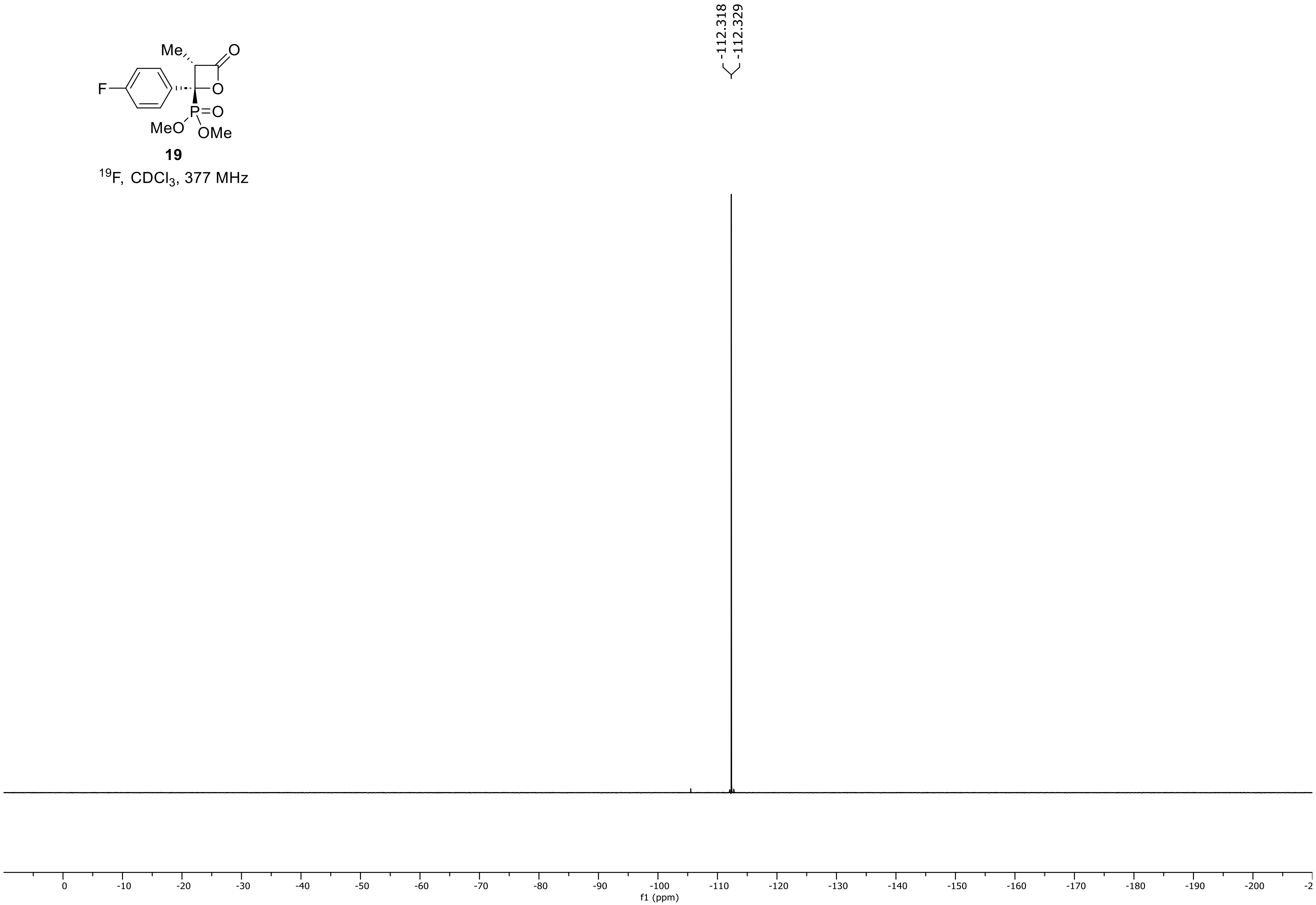

19

^{19}F, $CDCl_3$, 377 MHz

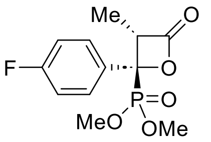

19

^{31}P, $CDCl_3$, 162 MHz

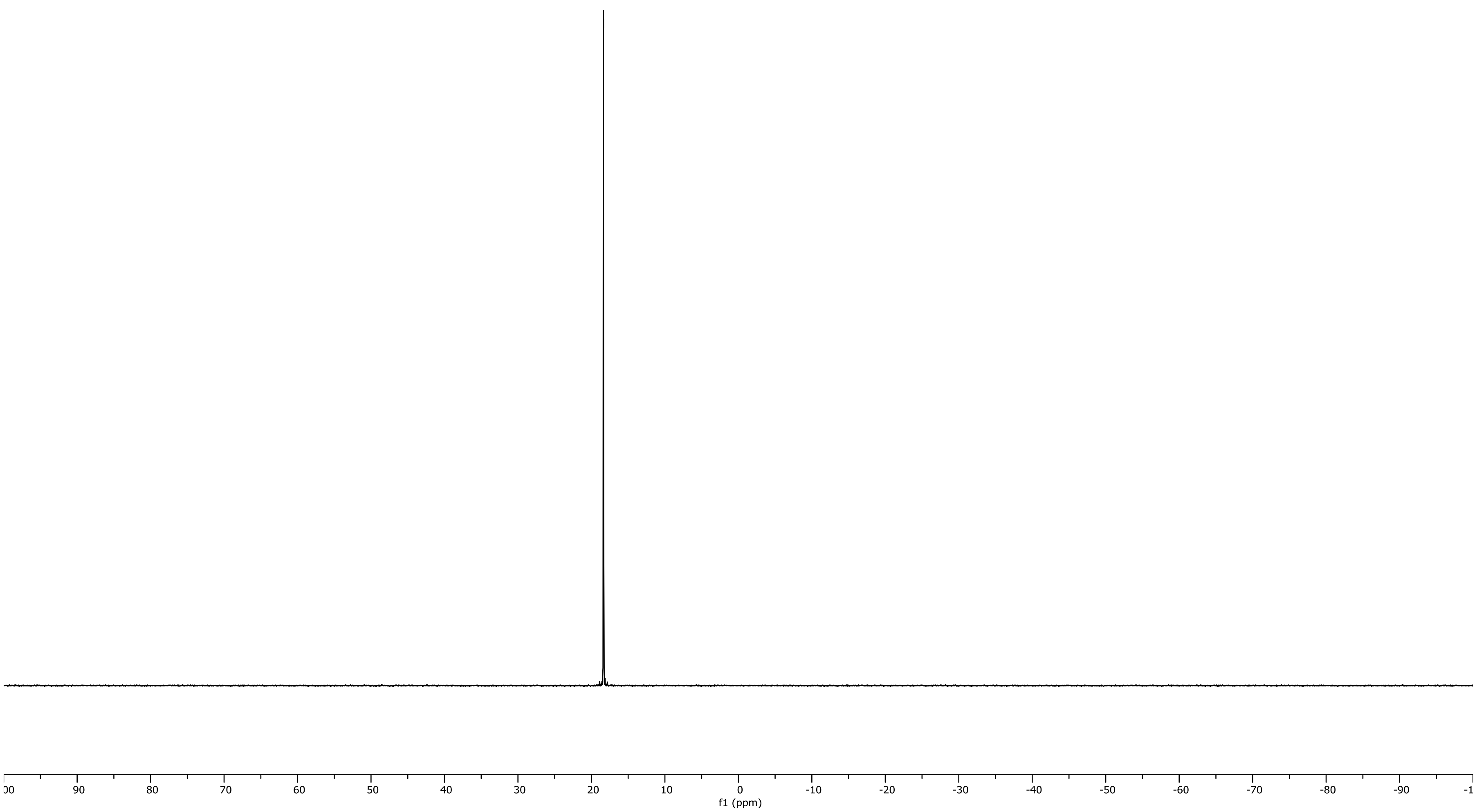

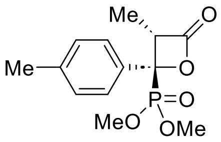

20

^{1}H, $CDCl_3$, 400 MHz

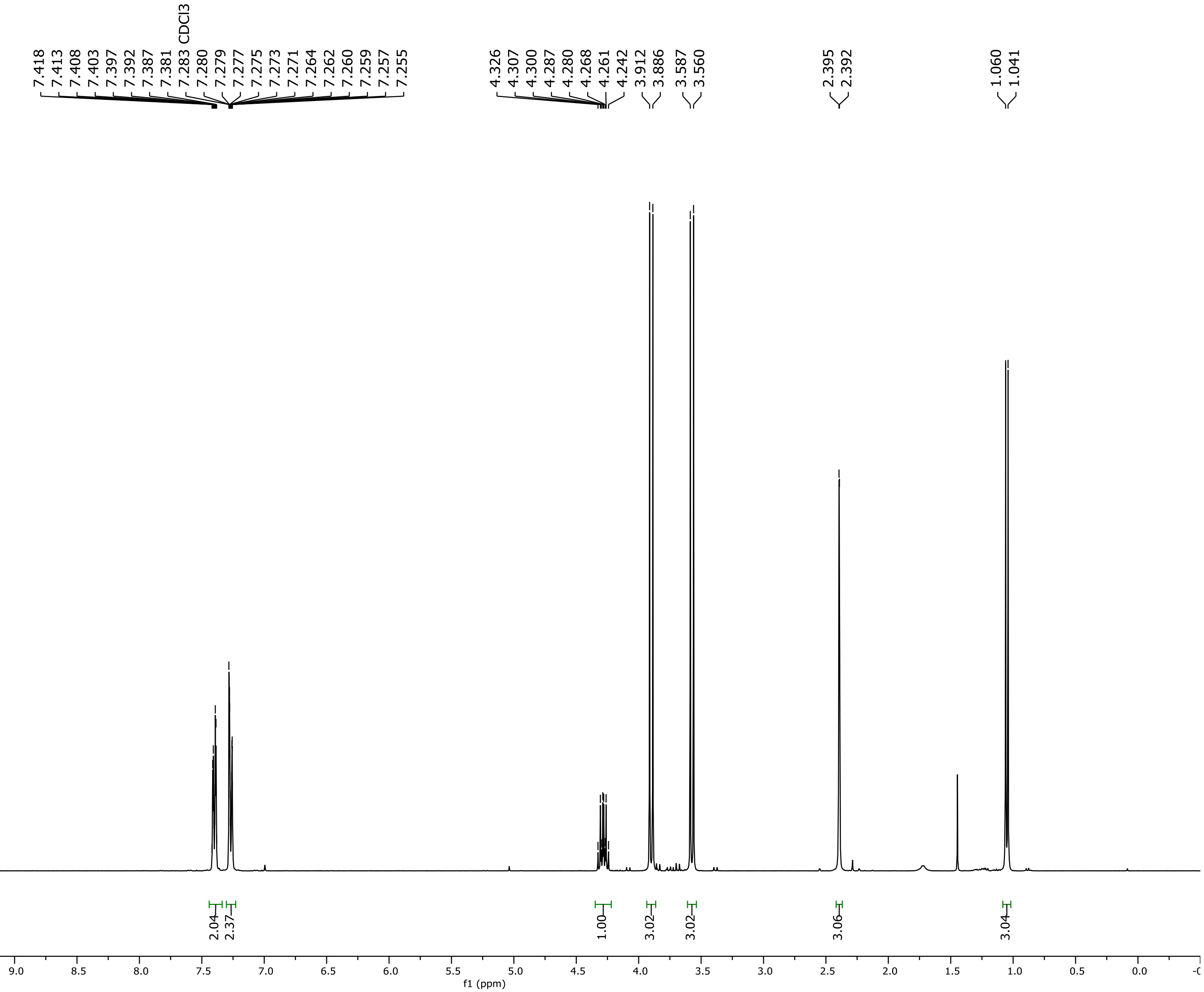

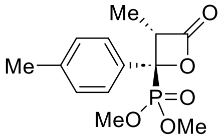

20

^{13}C, $CDCl_3$, 101 MHz

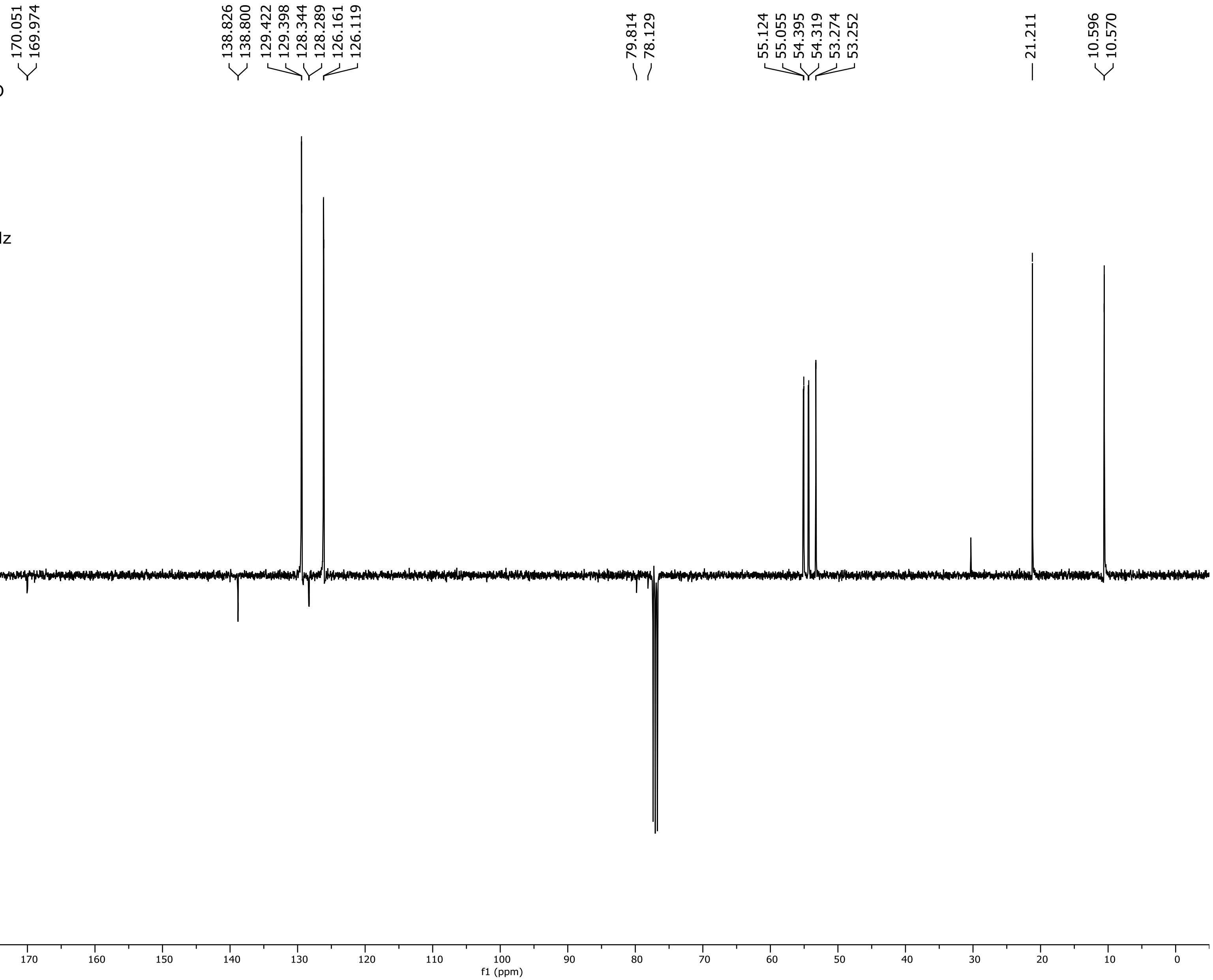

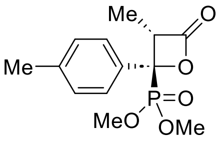

20

^{31}P, $CDCl_3$, 162 MHz

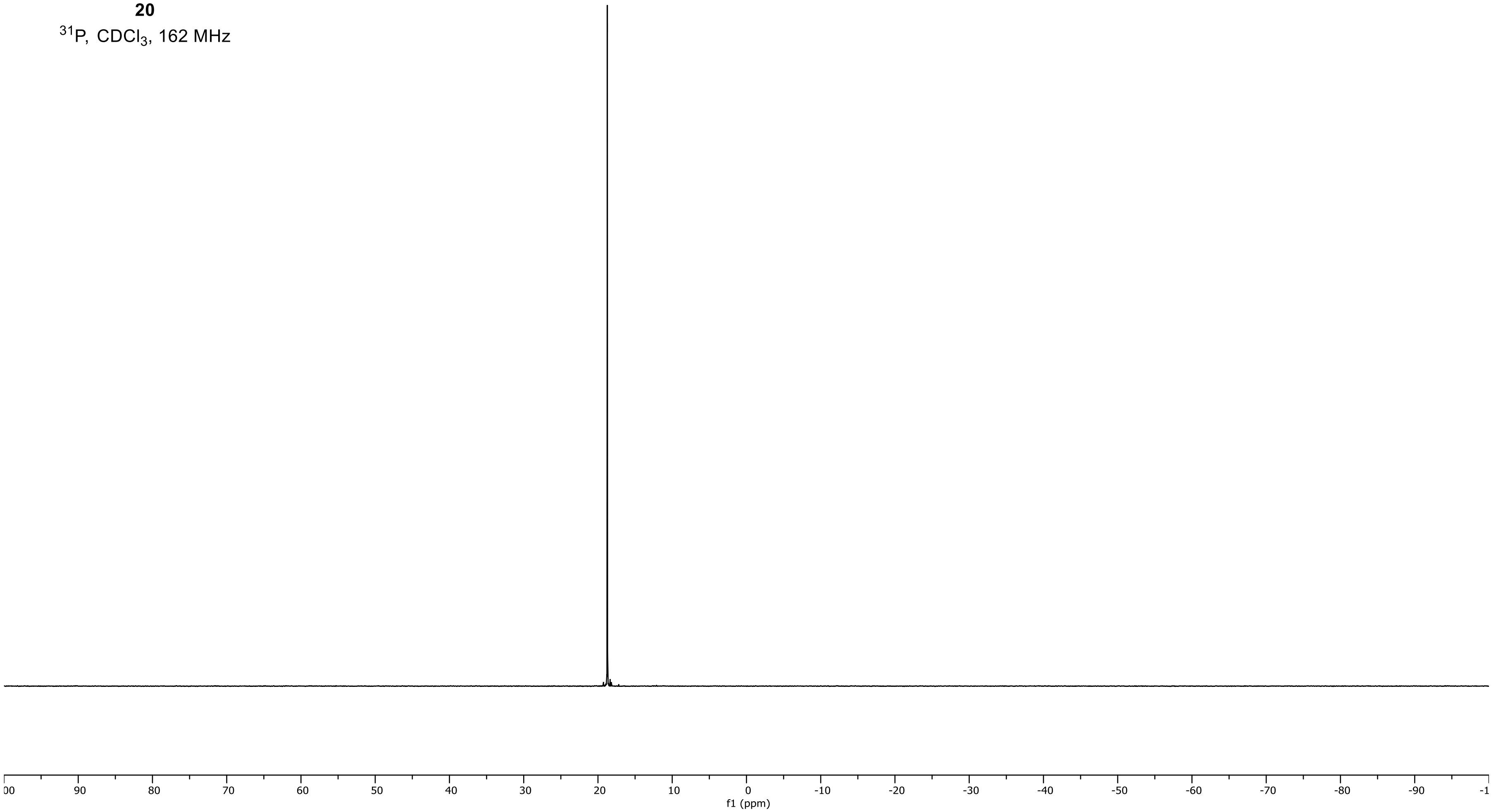

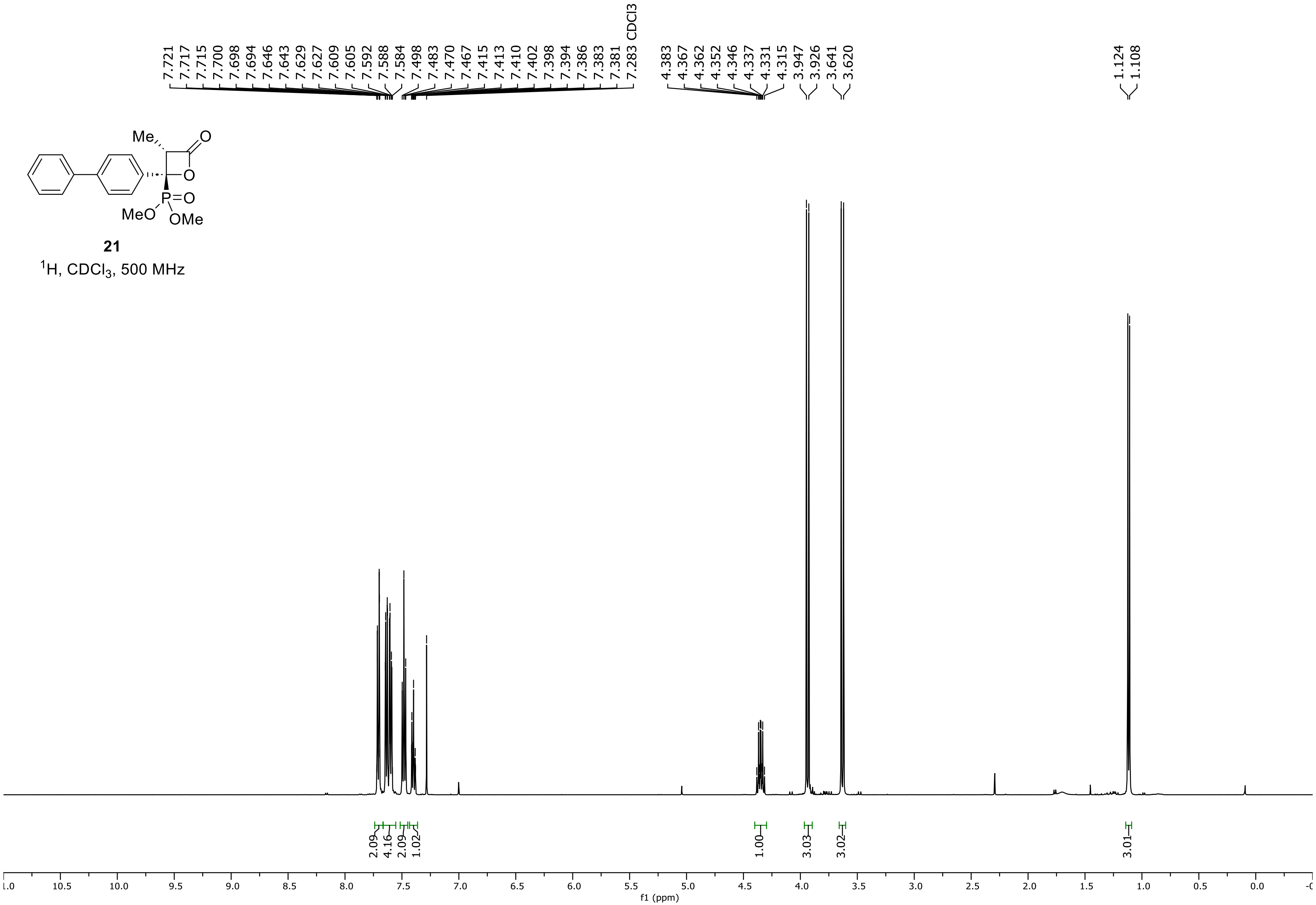

21

^1H, $CDCl_3$, 500 MHz

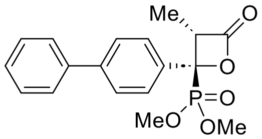

21

^{13}C, $CDCl_3$, 126 MHz

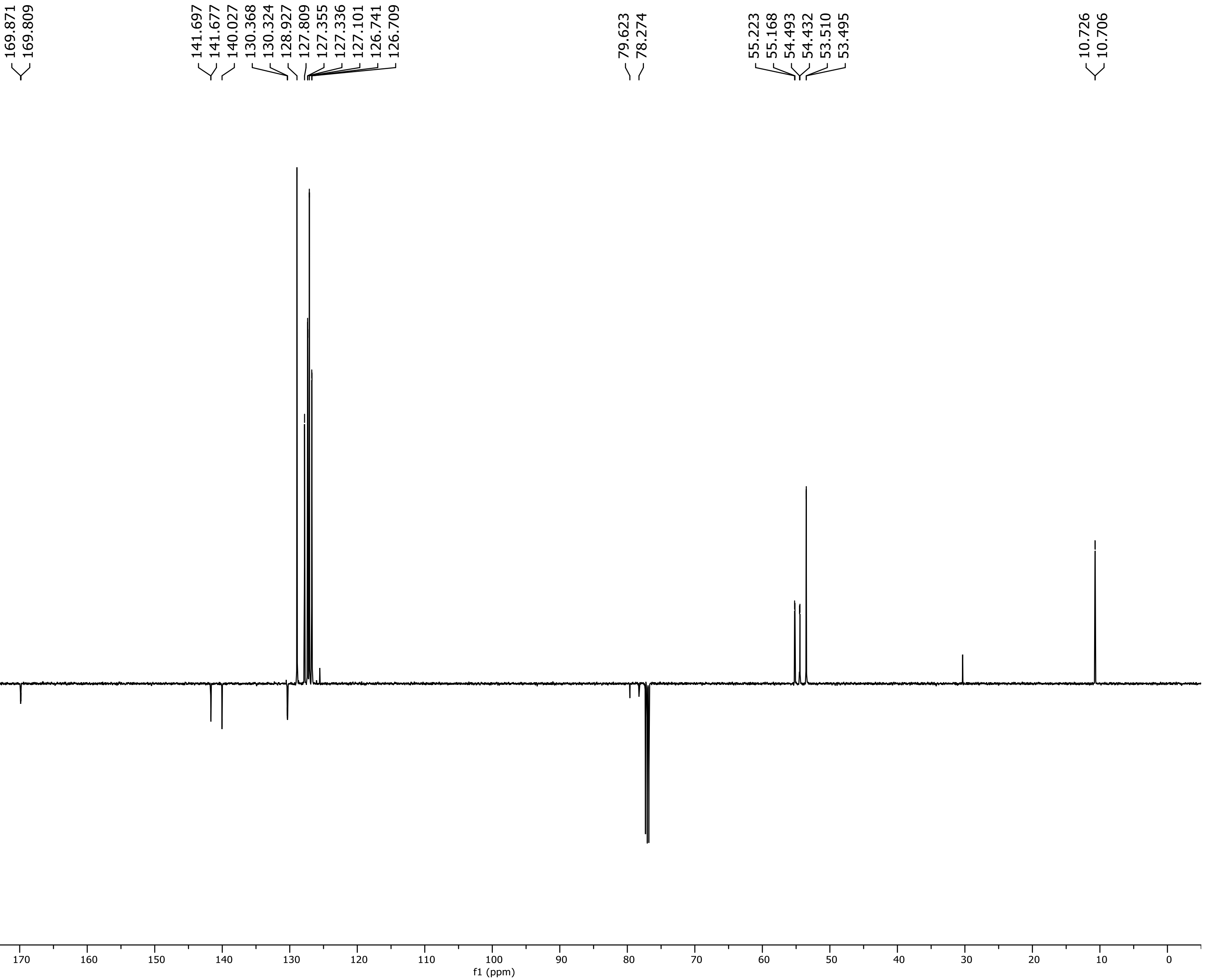

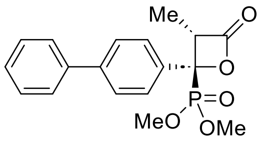

21

^{31}P, $CDCl_3$, 202 MHz

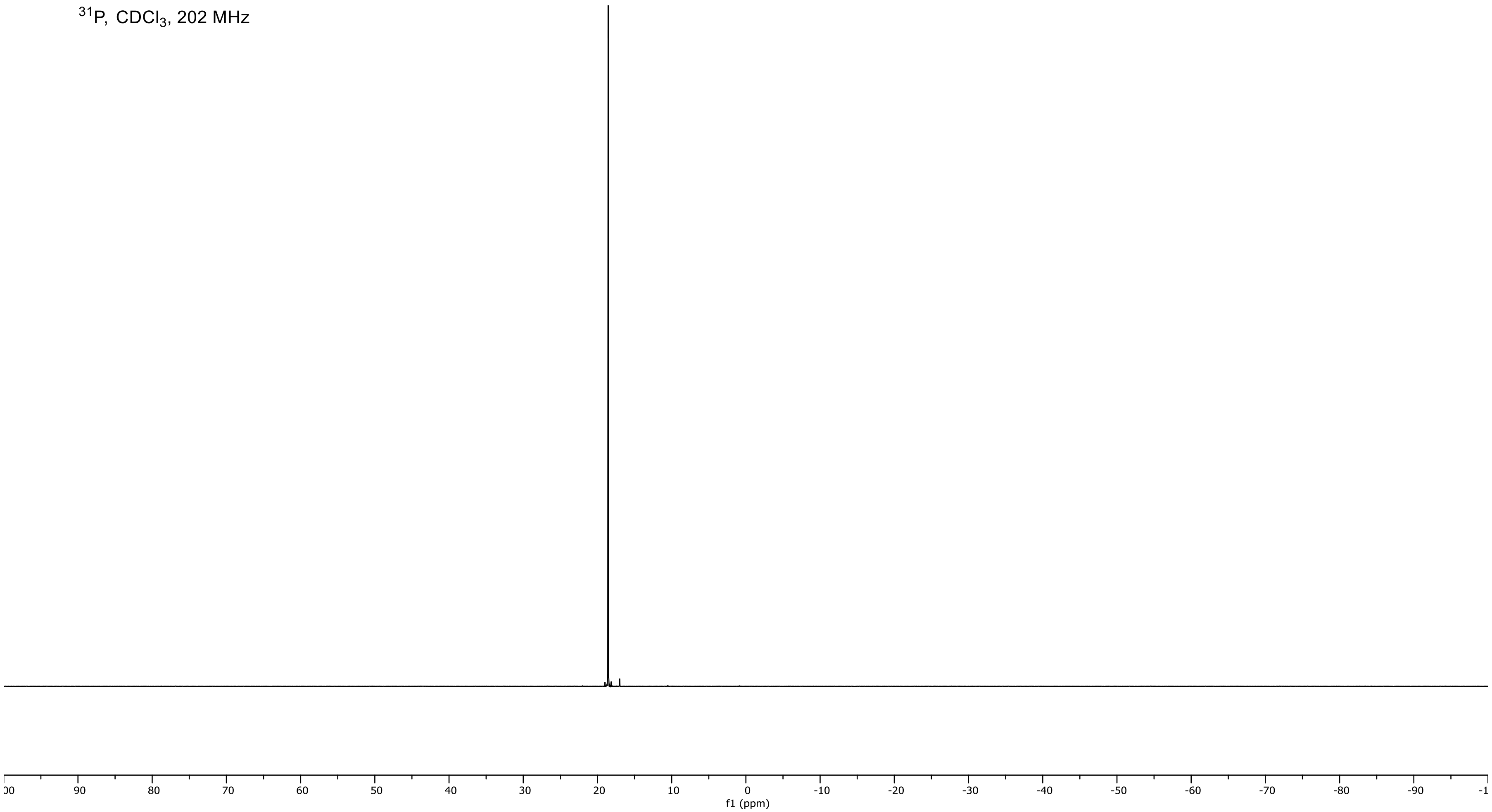

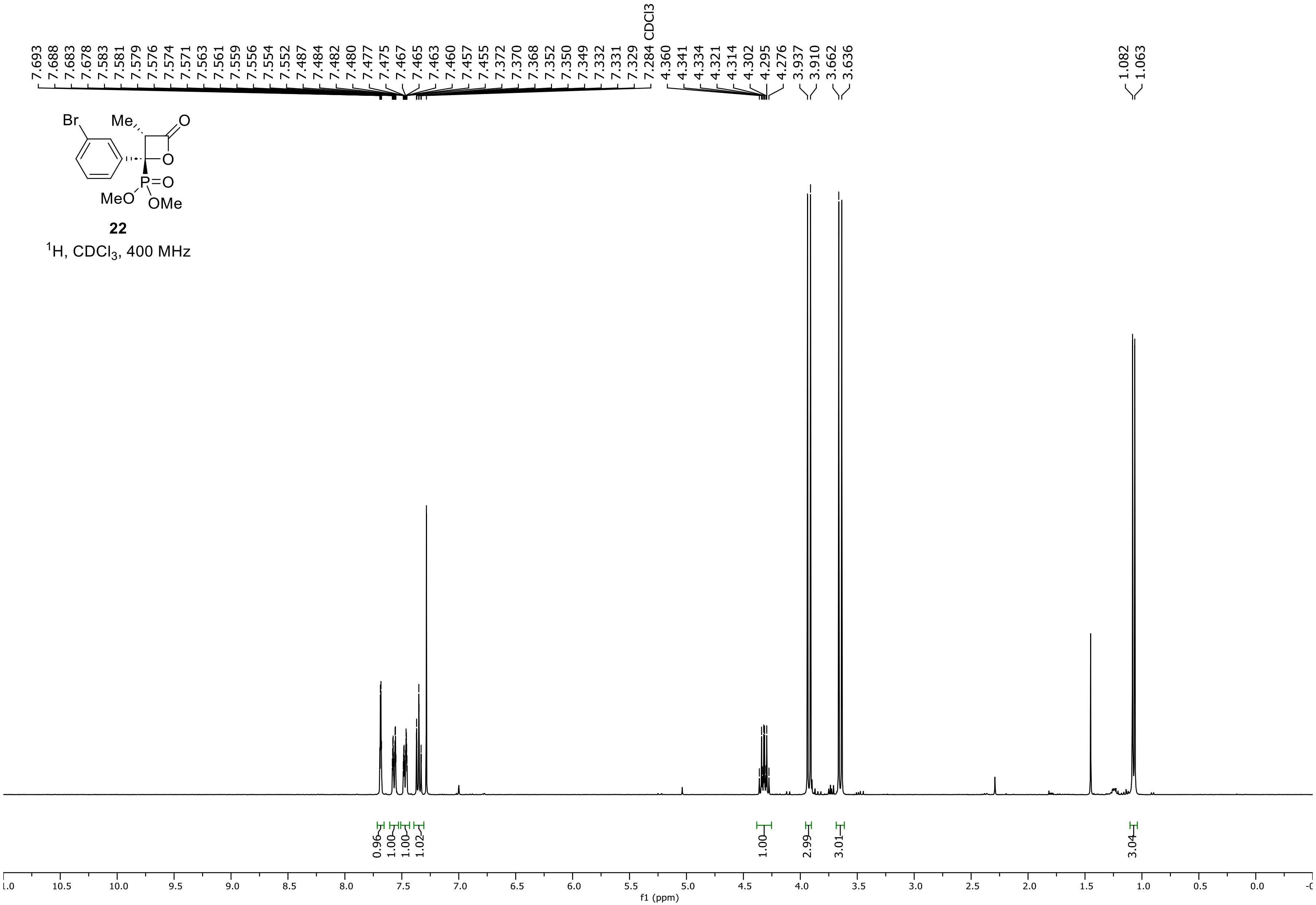
7.693
7.688
7.683
7.678
7.583
7.581
7.579
7.576
7.574
7.571
7.563
7.561
7.559
7.556
7.554
7.552
7.487
7.484
7.482
7.480
7.477
7.475
7.467
7.465
7.463
7.460
7.457
7.455
7.372
7.370
7.368
7.352
7.350
7.349
7.332
7.331
7.329
7.284 CDCl3
4.360
4.341
4.334
4.321
4.314
4.302
4.295
4.276
3.937
3.910
3.662
3.636
1.082
1.063
Br
Me
O
O
P=O
MeO
OMe
22
¹H, CDCl₃, 400 MHz
0.96
1.00
1.00
1.02
1.00
2.99
3.01
3.04
f1 (ppm)

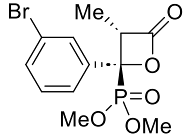

22

^{13}C, $CDCl_3$, 101 MHz

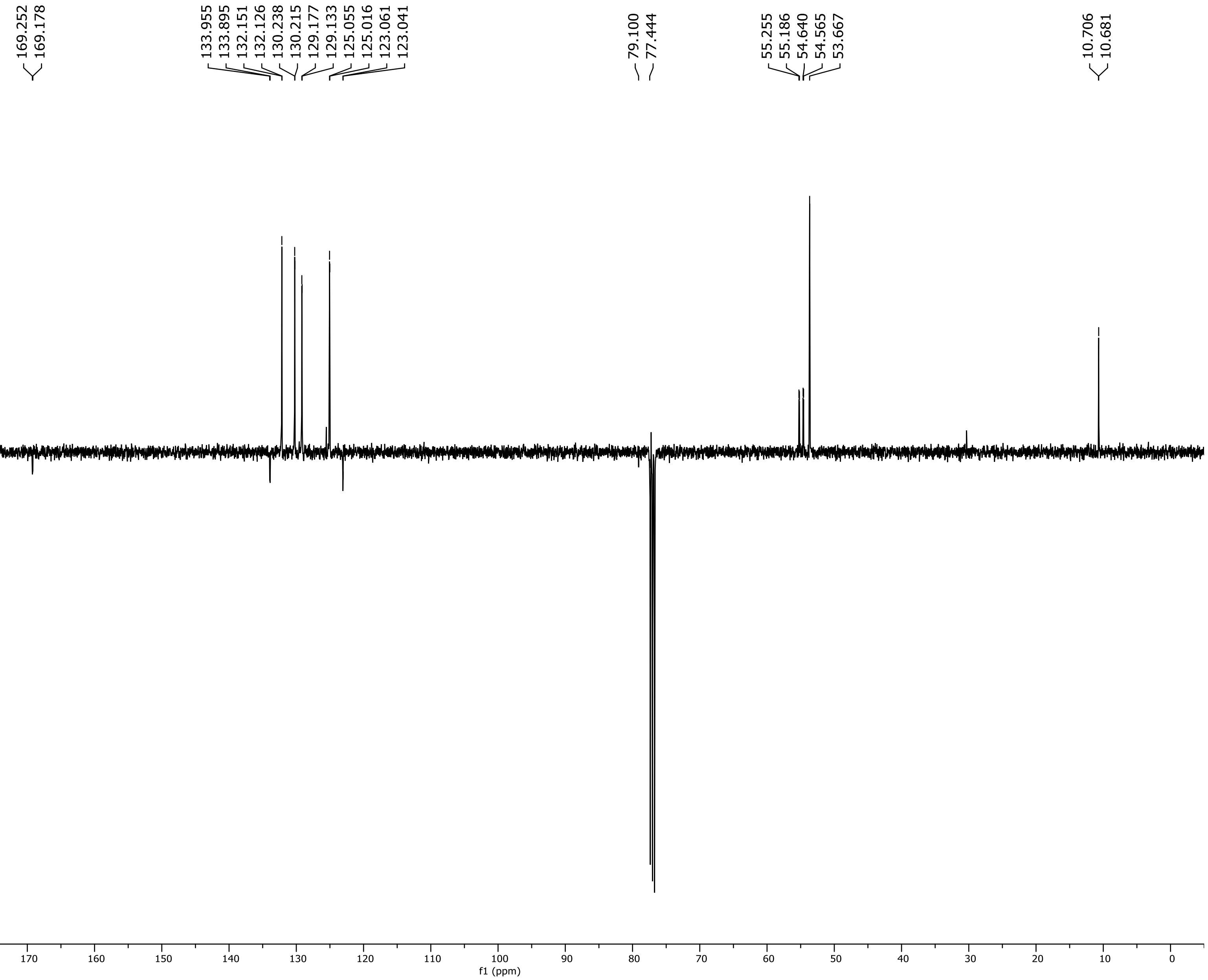

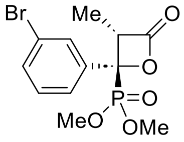

22

^{31}P, $CDCl_3$, 162 MHz

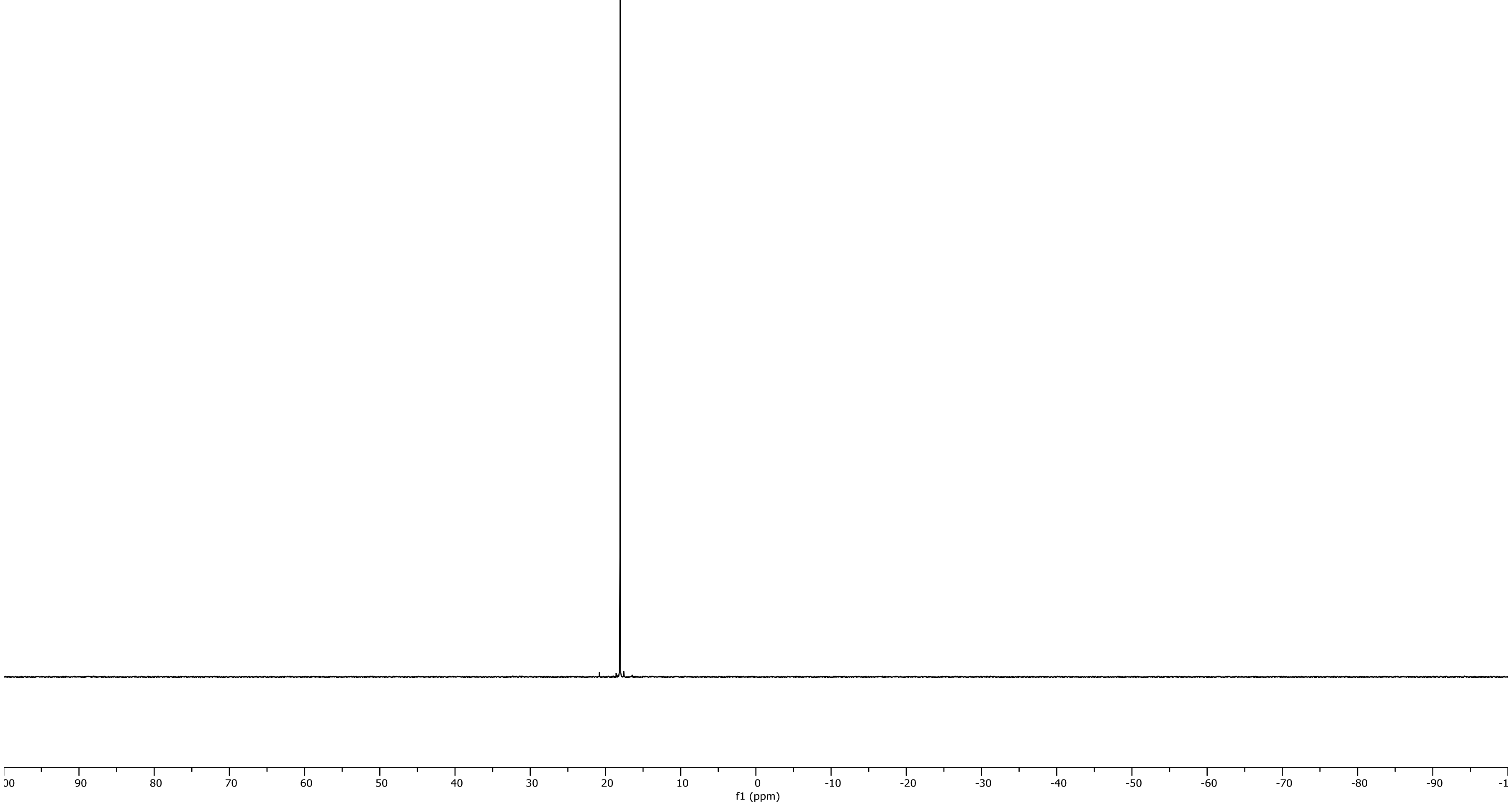

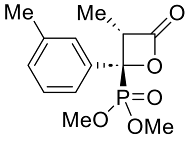

23

^1H, $CDCl_3$, 400 MHz

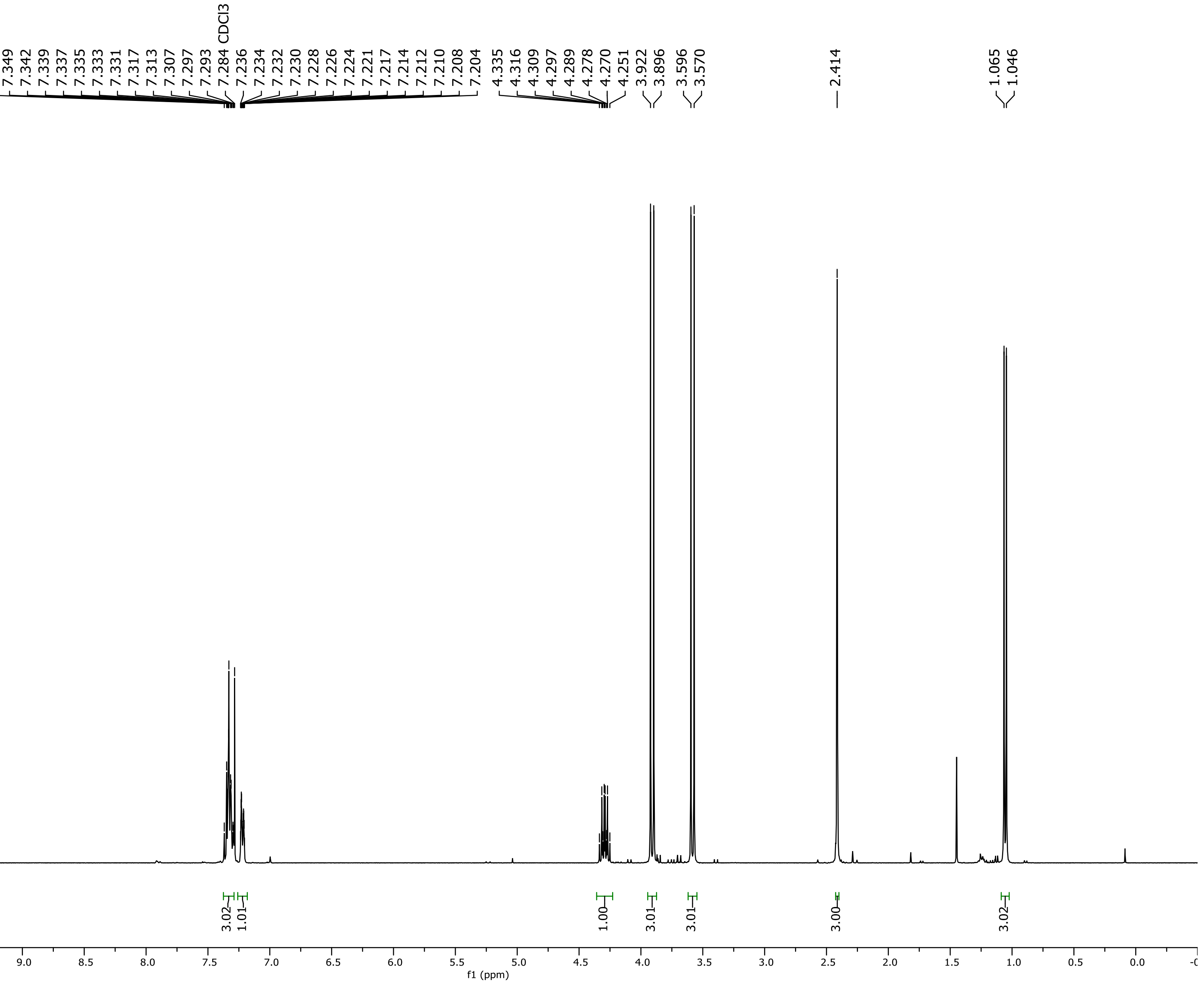

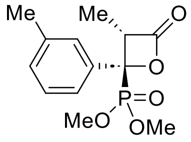

23

^{13}C, $CDCl_3$, 101 MHz

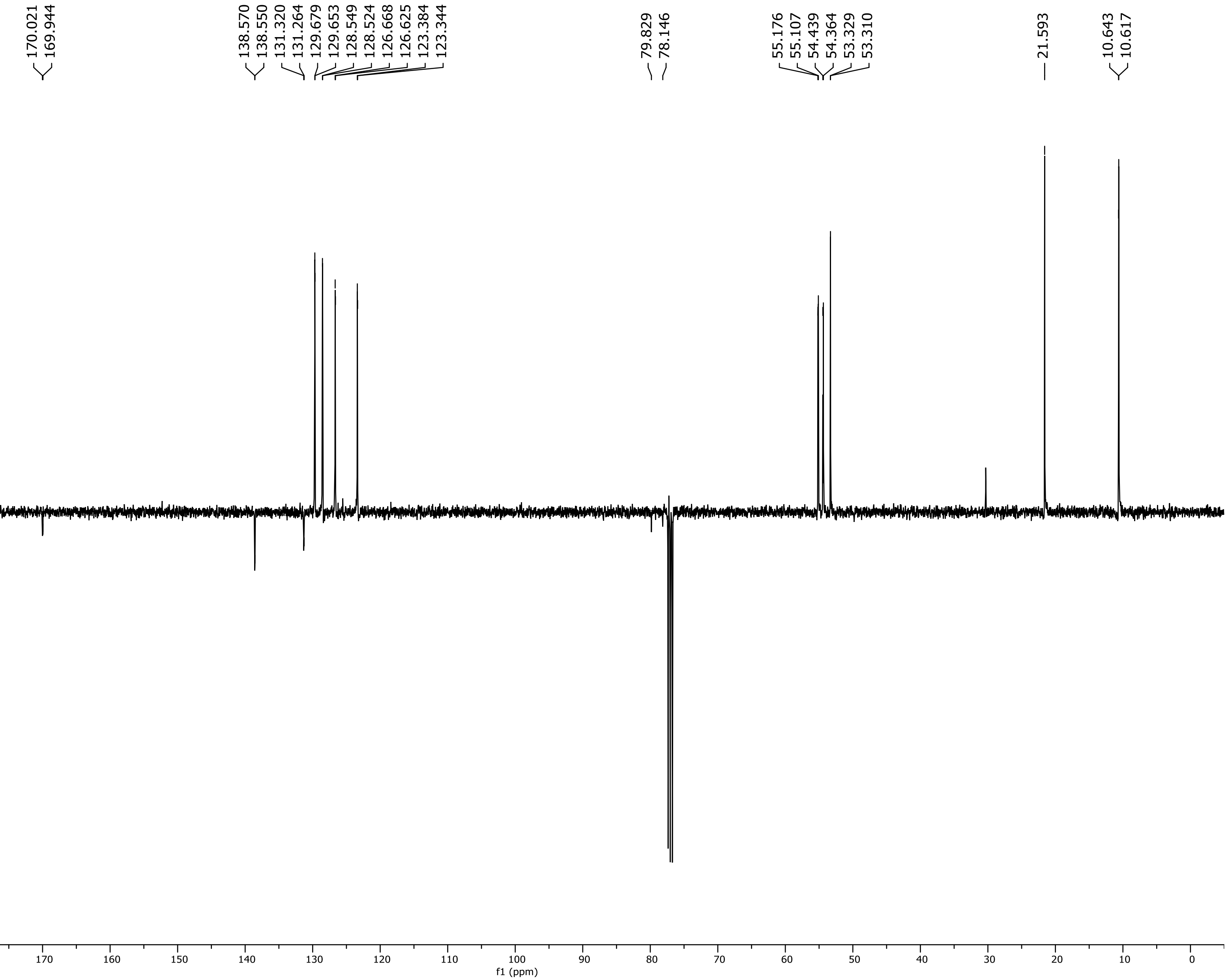

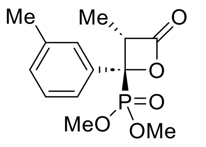

23

^{31}P, $CDCl_3$, 162 MHz

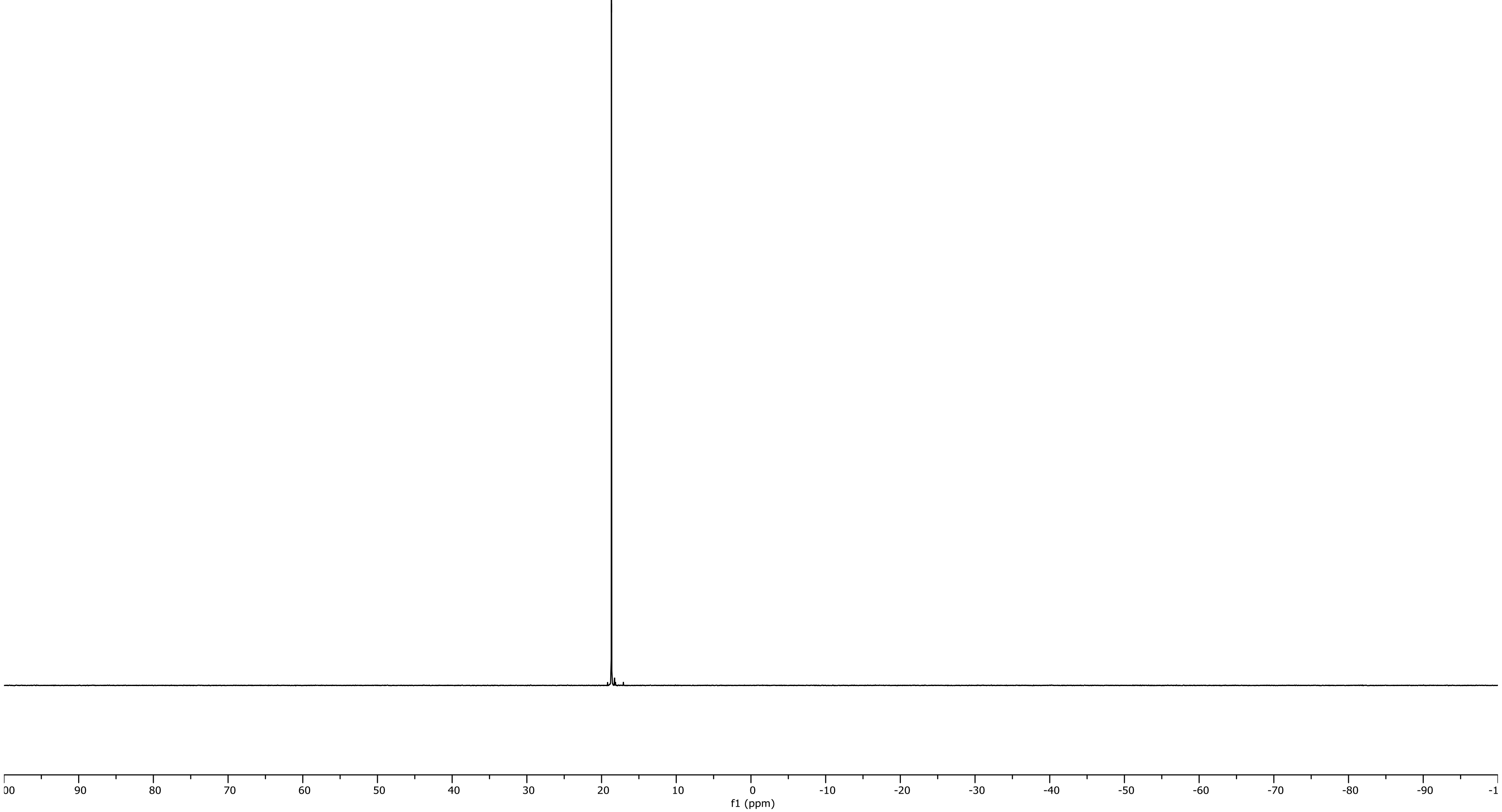

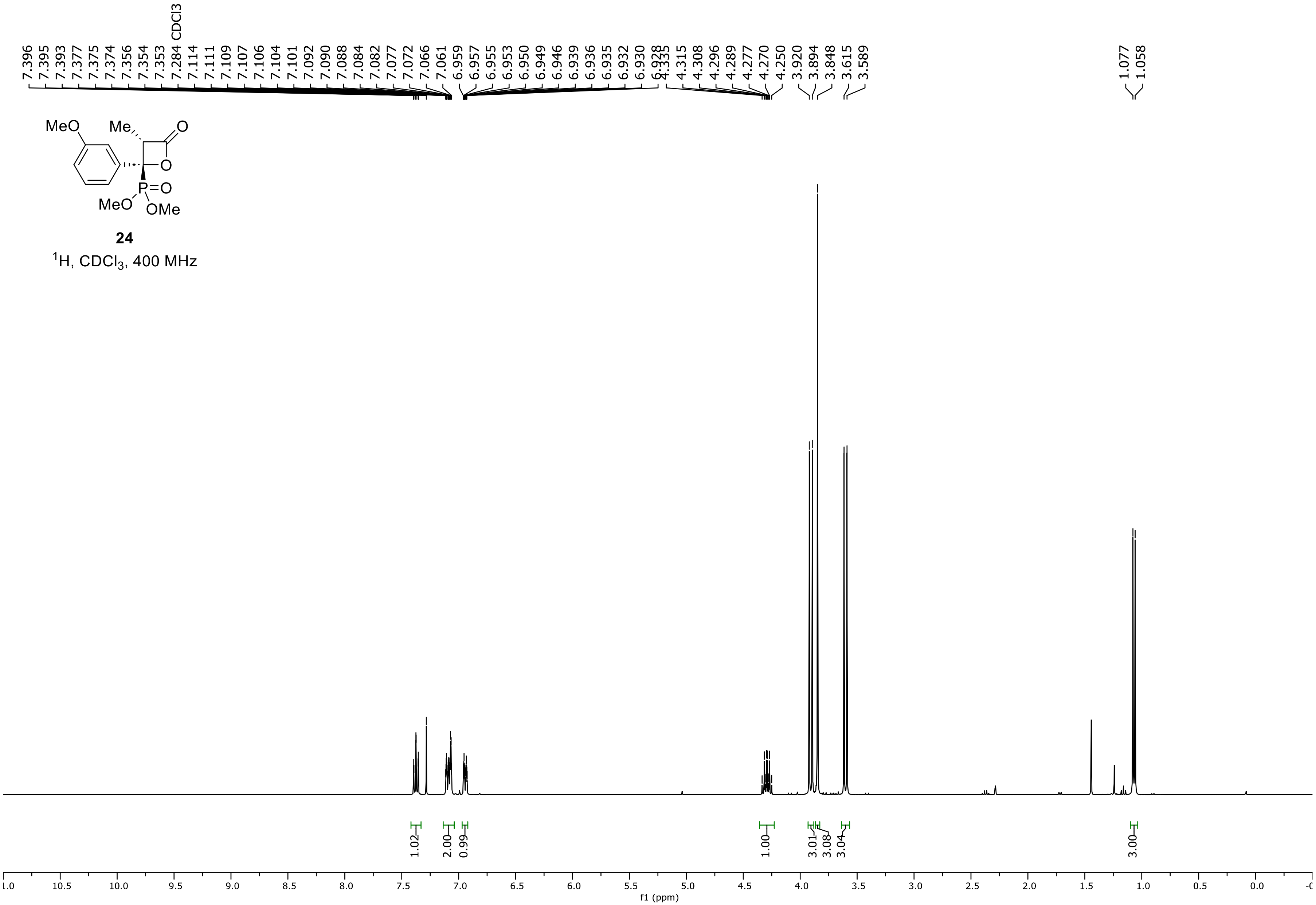

24

^{1}H, $CDCl_3$, 400 MHz

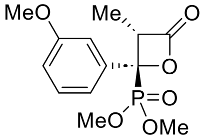

24

^{13}C, $CDCl_3$, 101 MHz

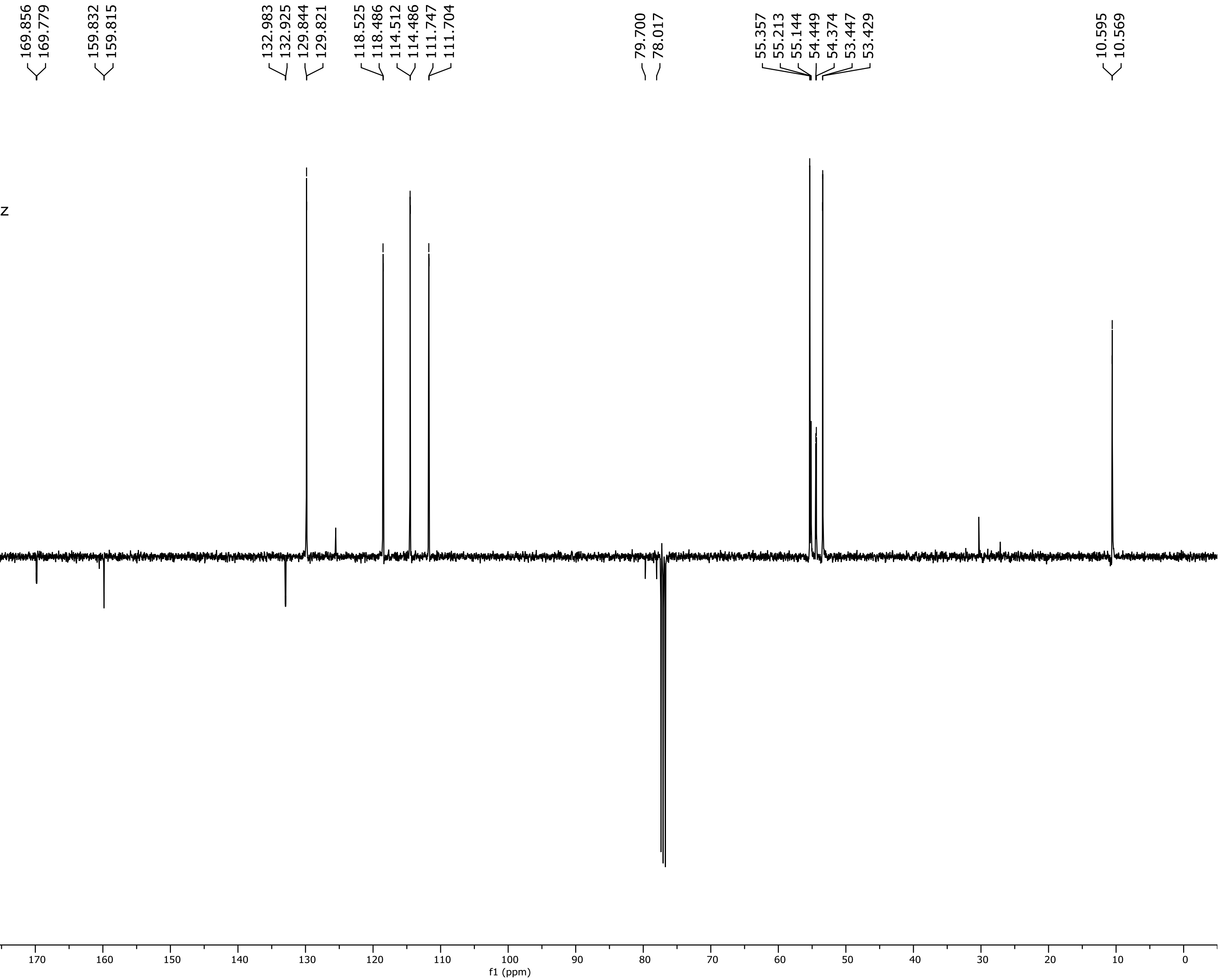

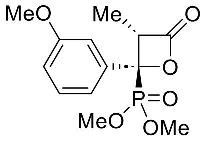

24

^{31}P, $CDCl_3$, 162 MHz

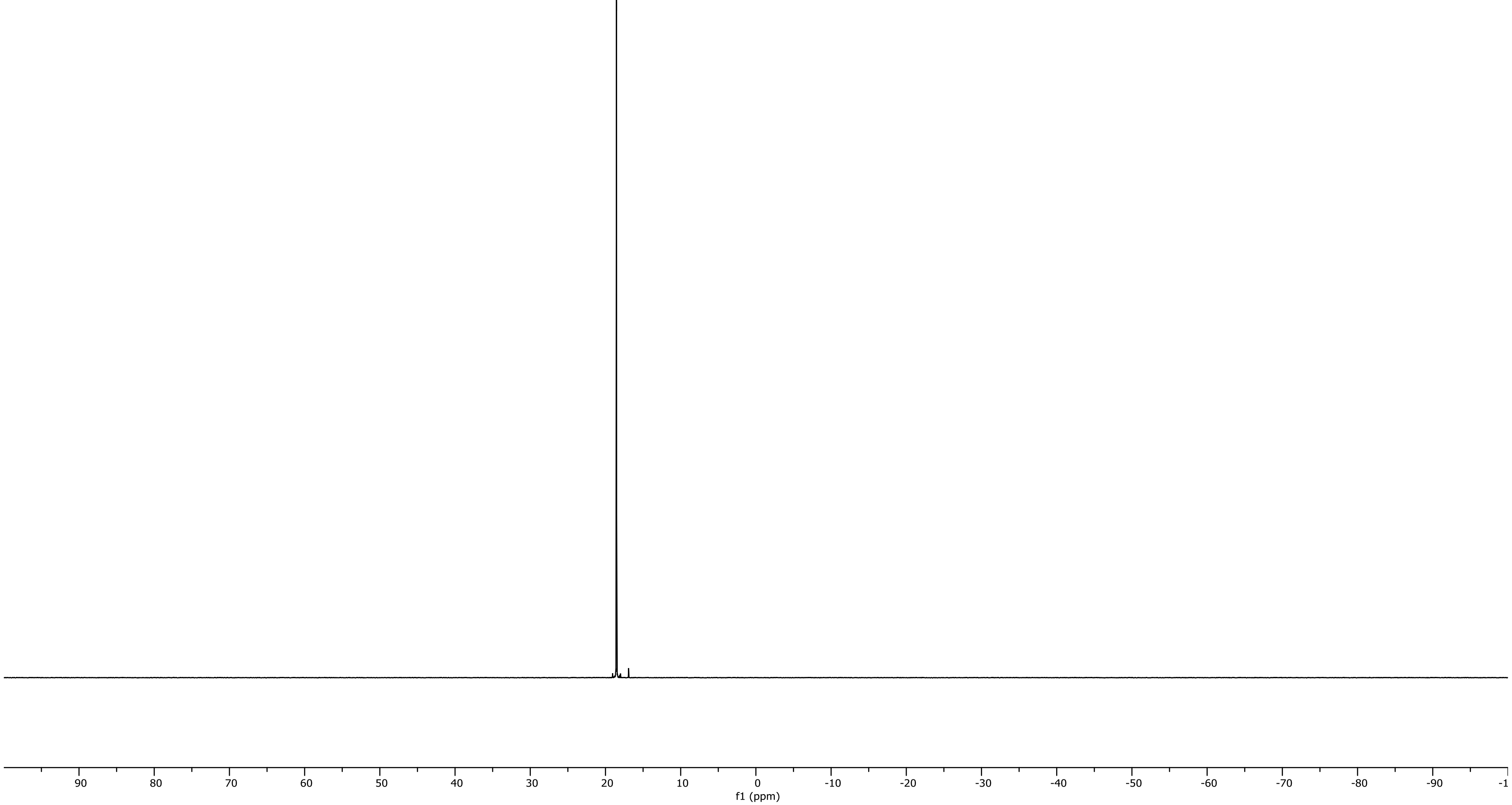

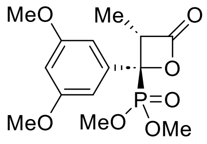

25

^{1}H, $CDCl_3$, 500 MHz

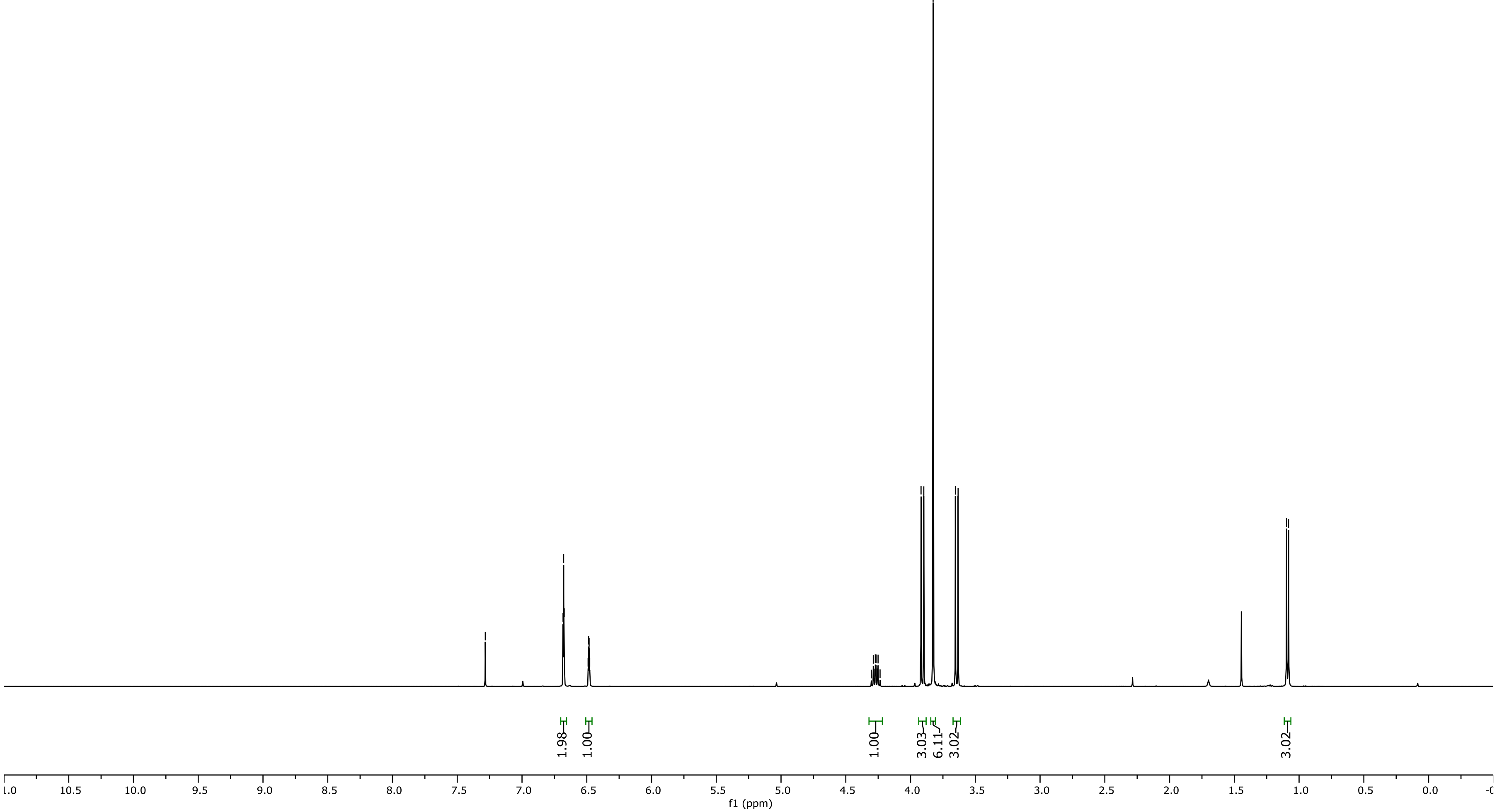

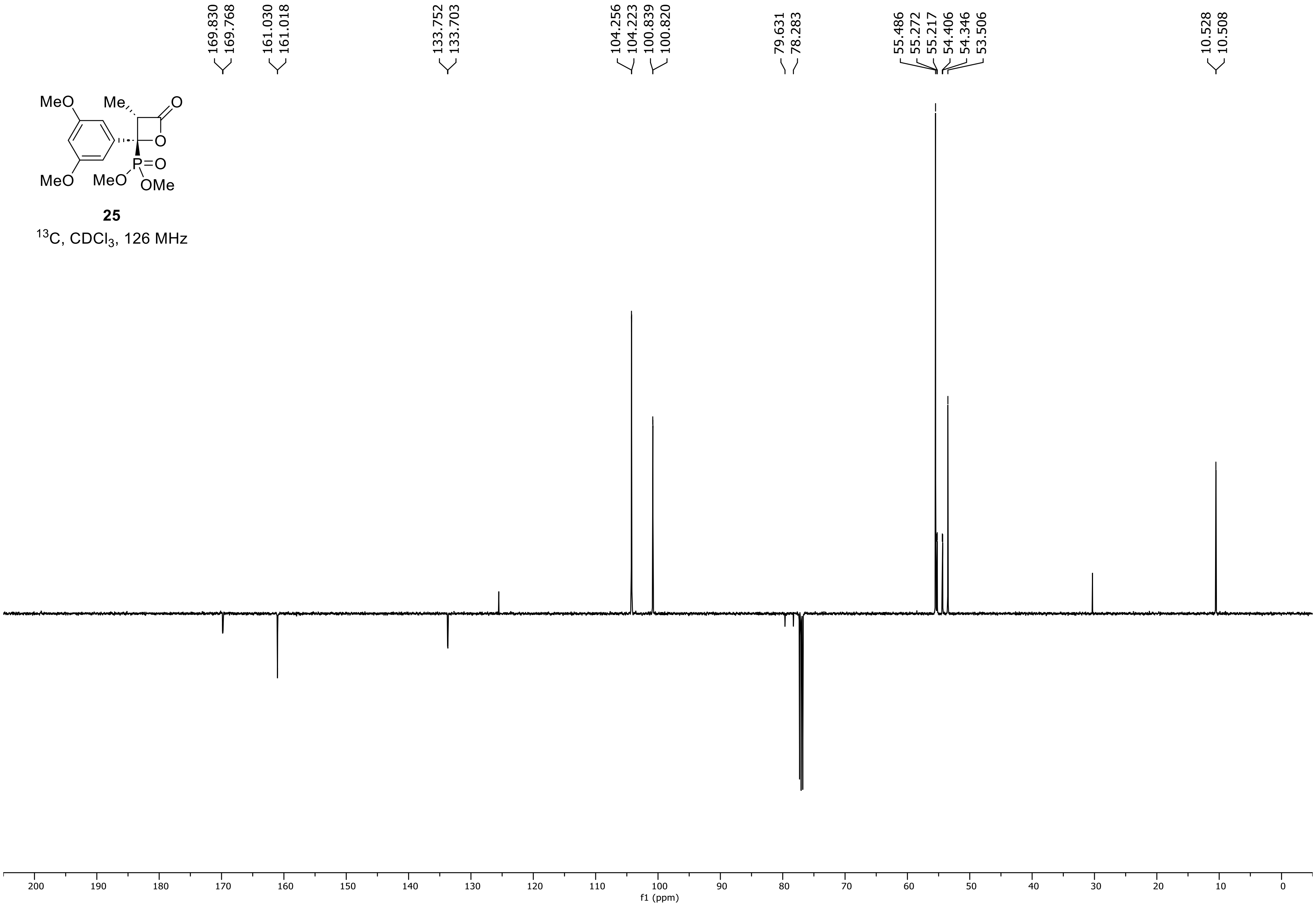
169.830
169.768
161.030
161.018
133.752
133.703
104.256
104.223
100.839
100.820
79.631
78.283
55.486
55.272
55.217
54.406
54.346
53.506
10.528
10.508
MeO
Me
O
O
P=O
MeO
MeO
OMe
25
^{13}C, $CDCl_3$, 126 MHz
200
190
180
170
160
150
140
130
120
110
100
90
80
70
60
50
40
30
20
10
0
f1 (ppm)

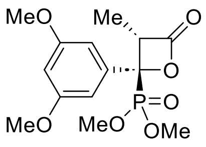

25

^{31}P, $CDCl_3$, 202 MHz

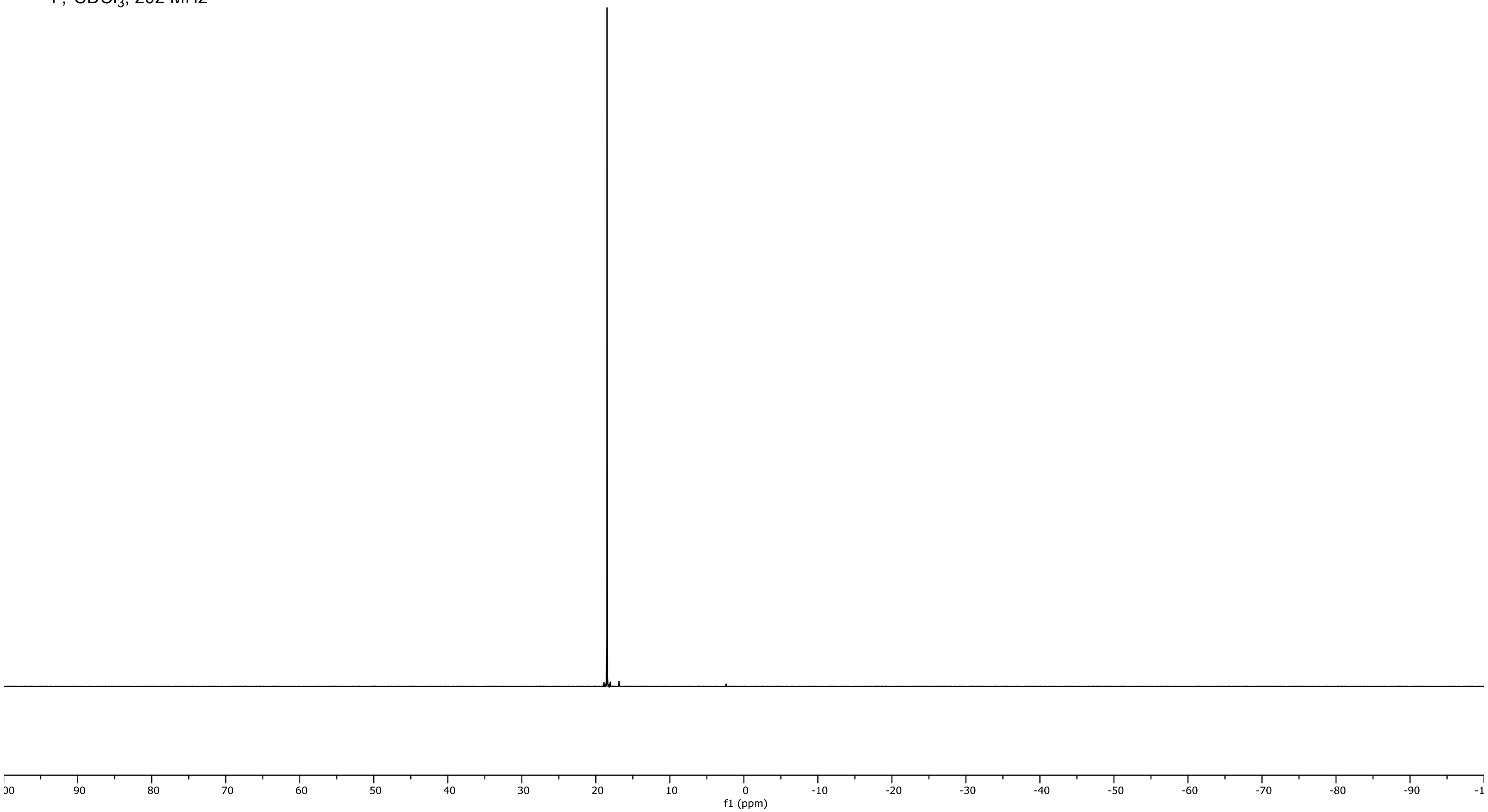

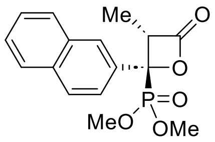

26

^1H, $CDCl_3$, 400 MHz

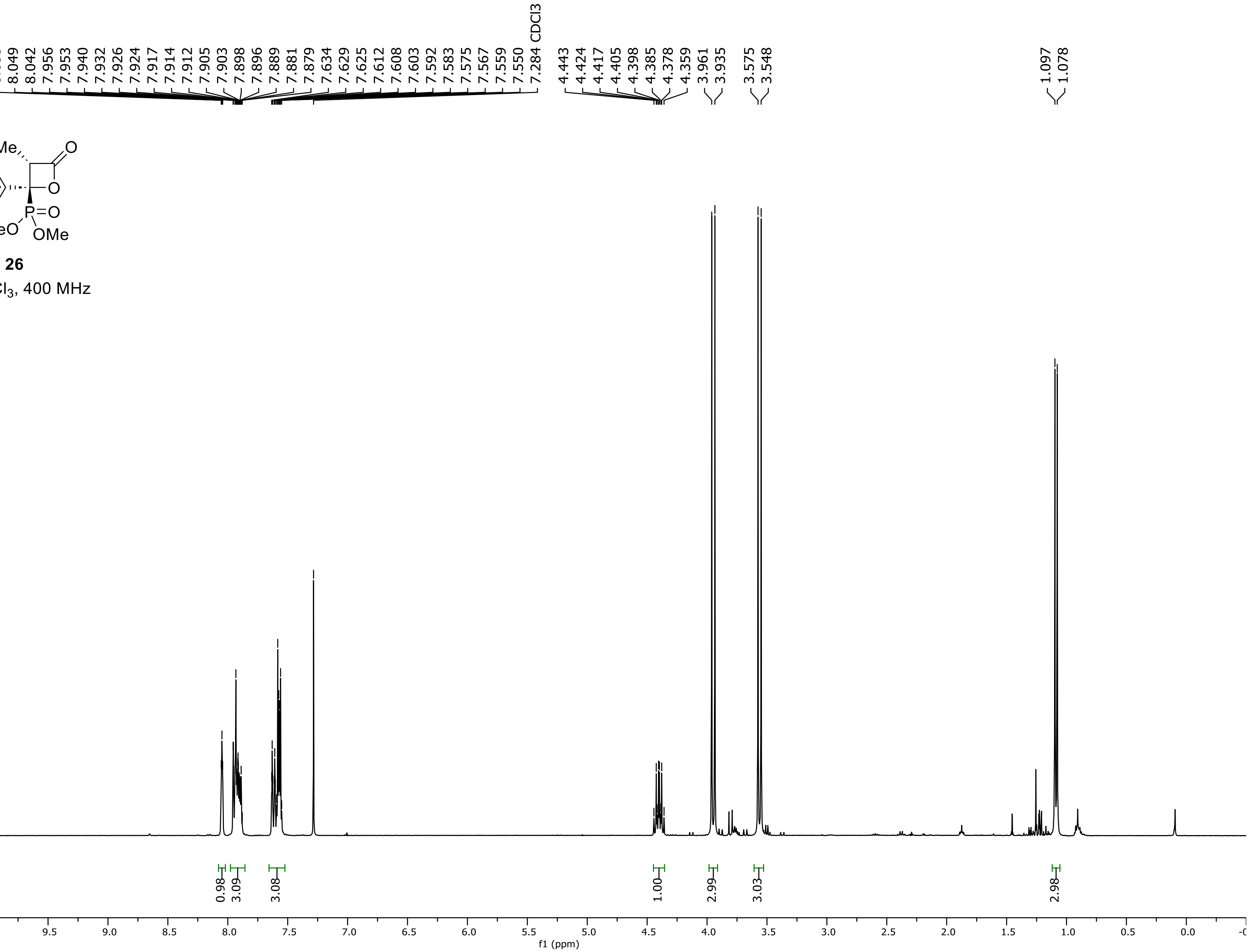

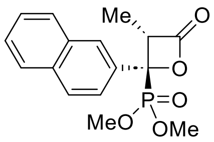

26

^{13}C, $CDCl_3$, 101 MHz

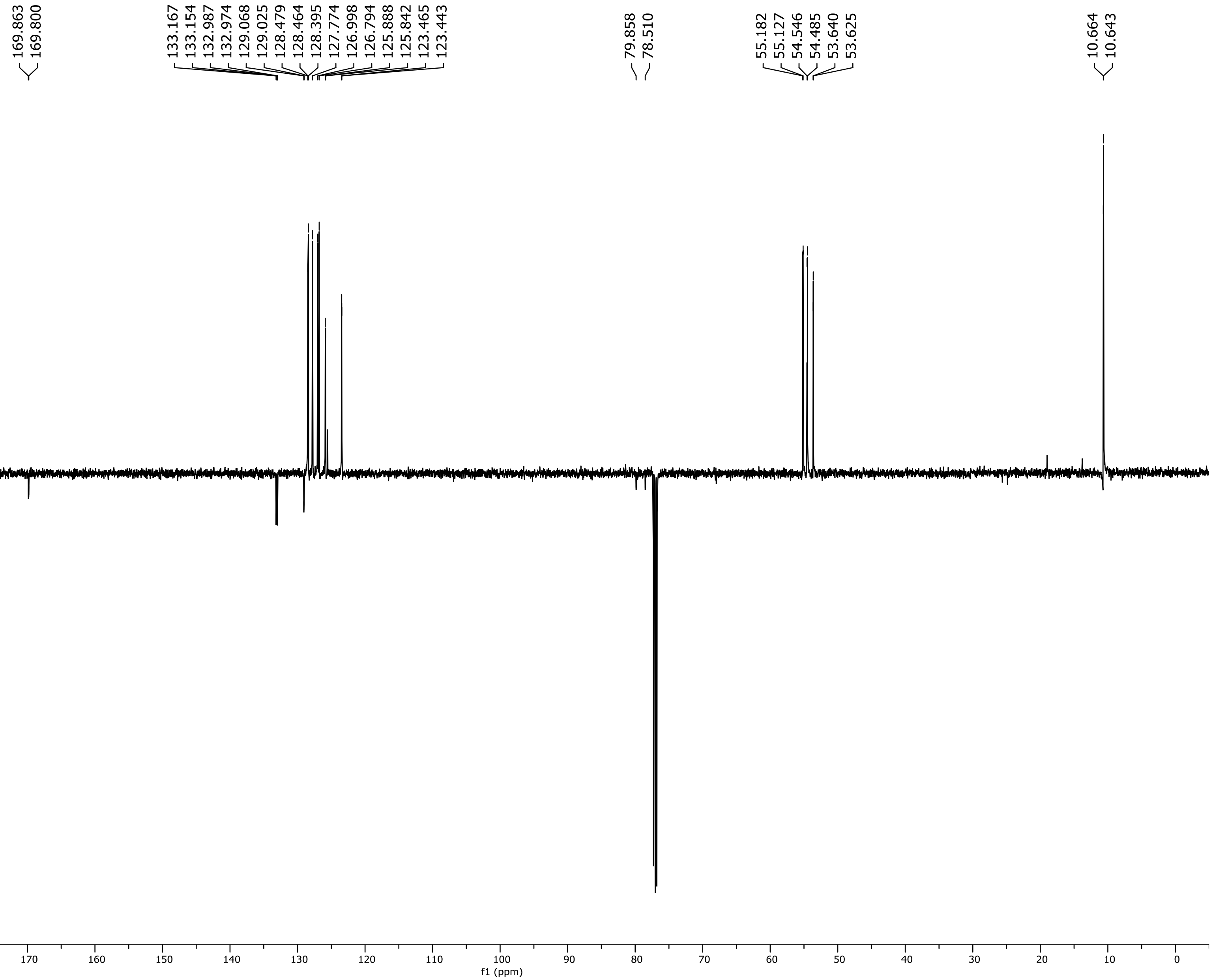

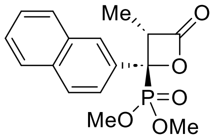

26

^{31}P, $CDCl_3$, 162 MHz

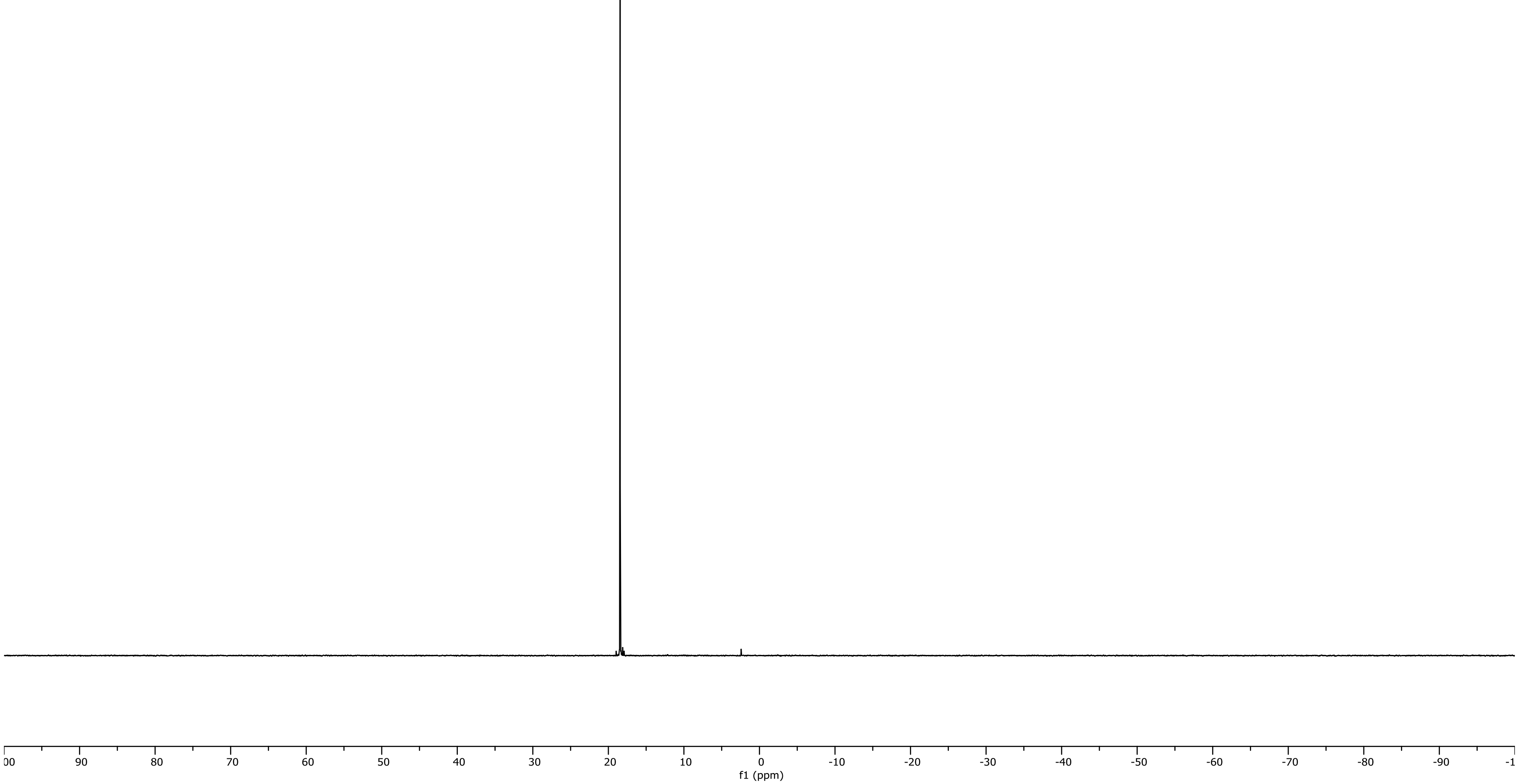

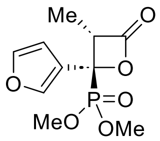

27

^{1}H, $CDCl_3$, 400 MHz

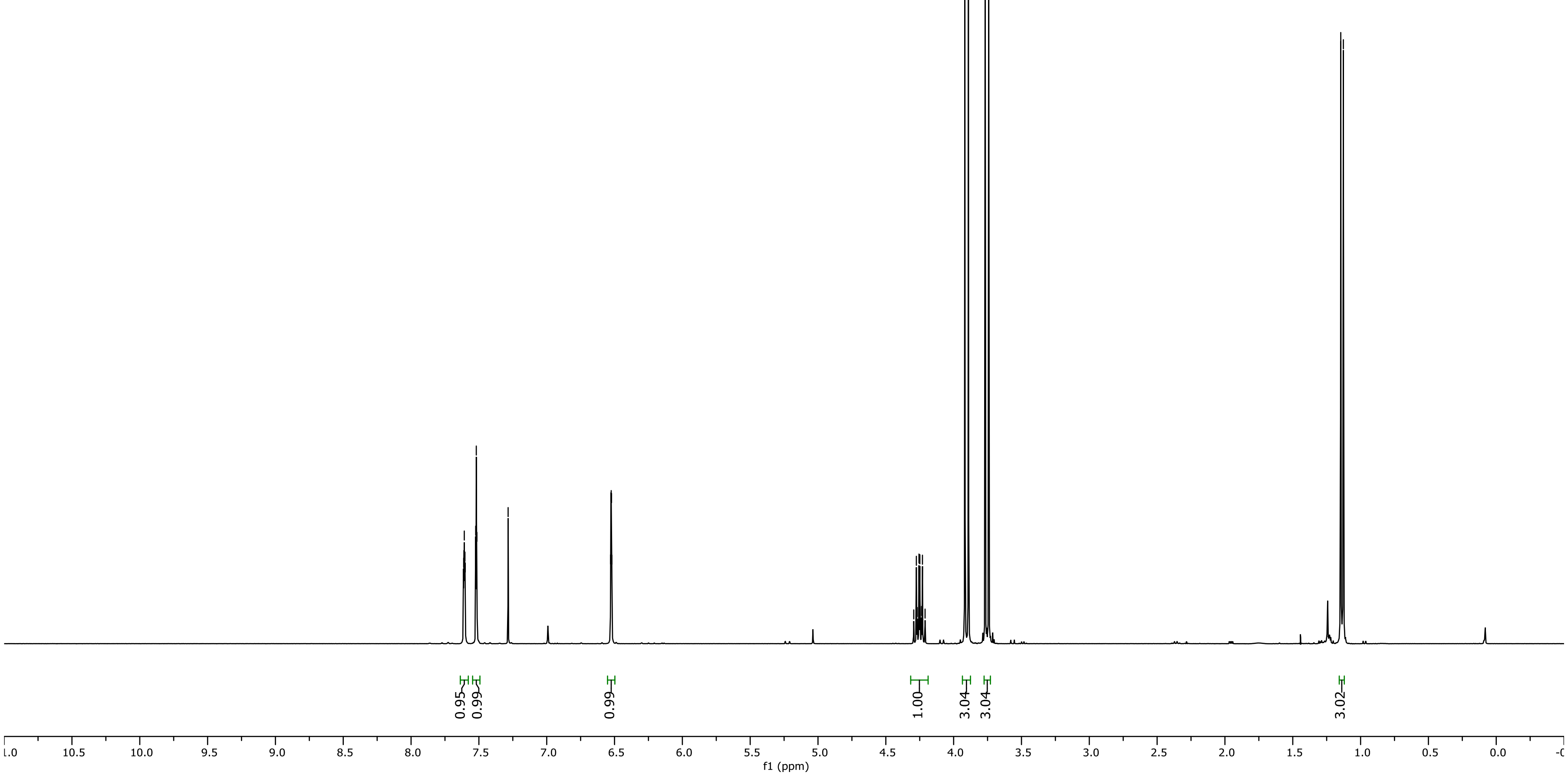

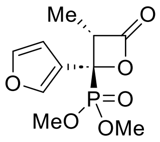

27

^{13}C, $CDCl_3$, 101 MHz

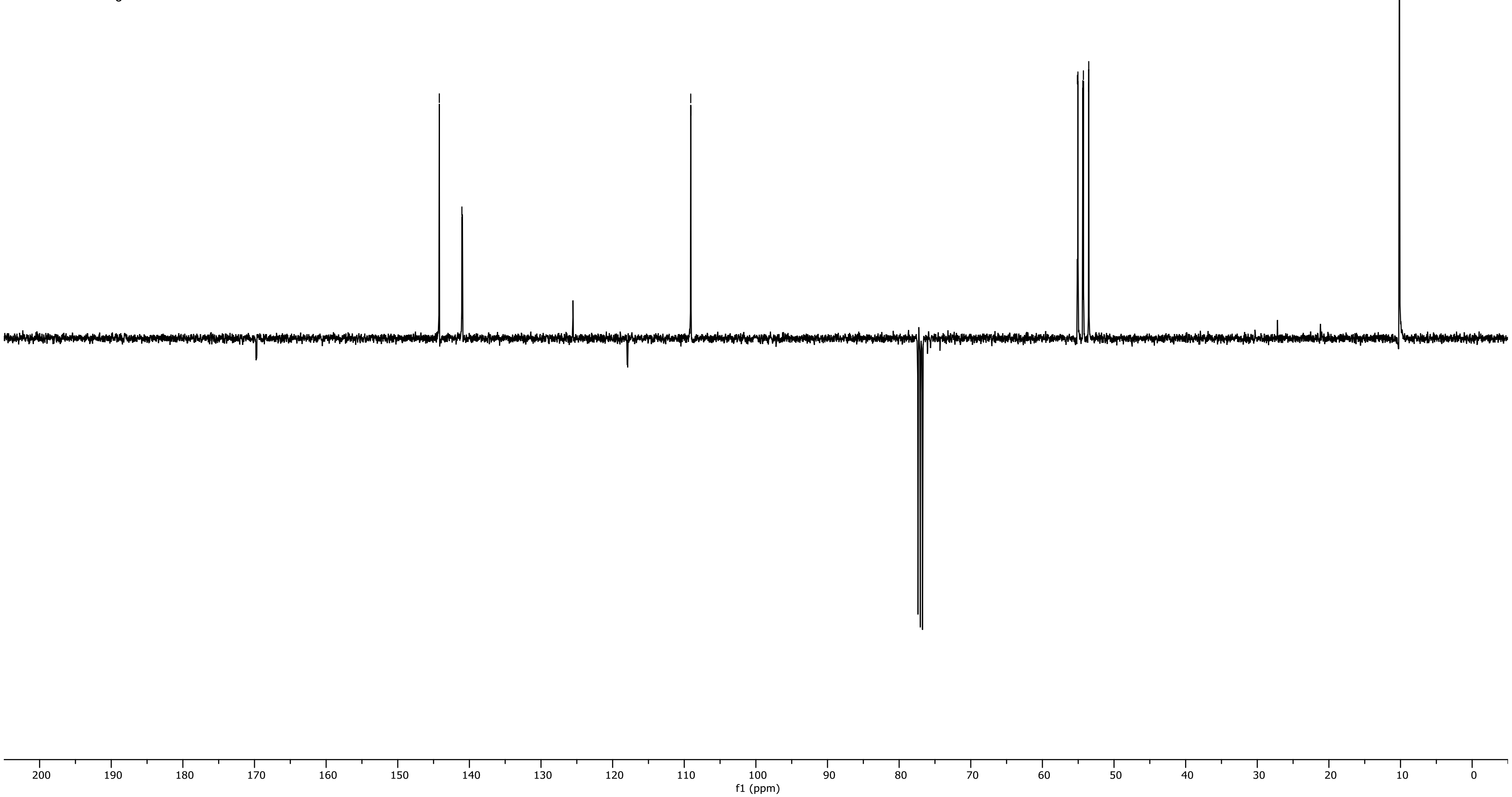

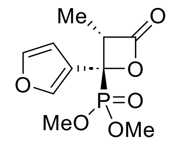

27

^{31}P, $CDCl_3$, 162 MHz

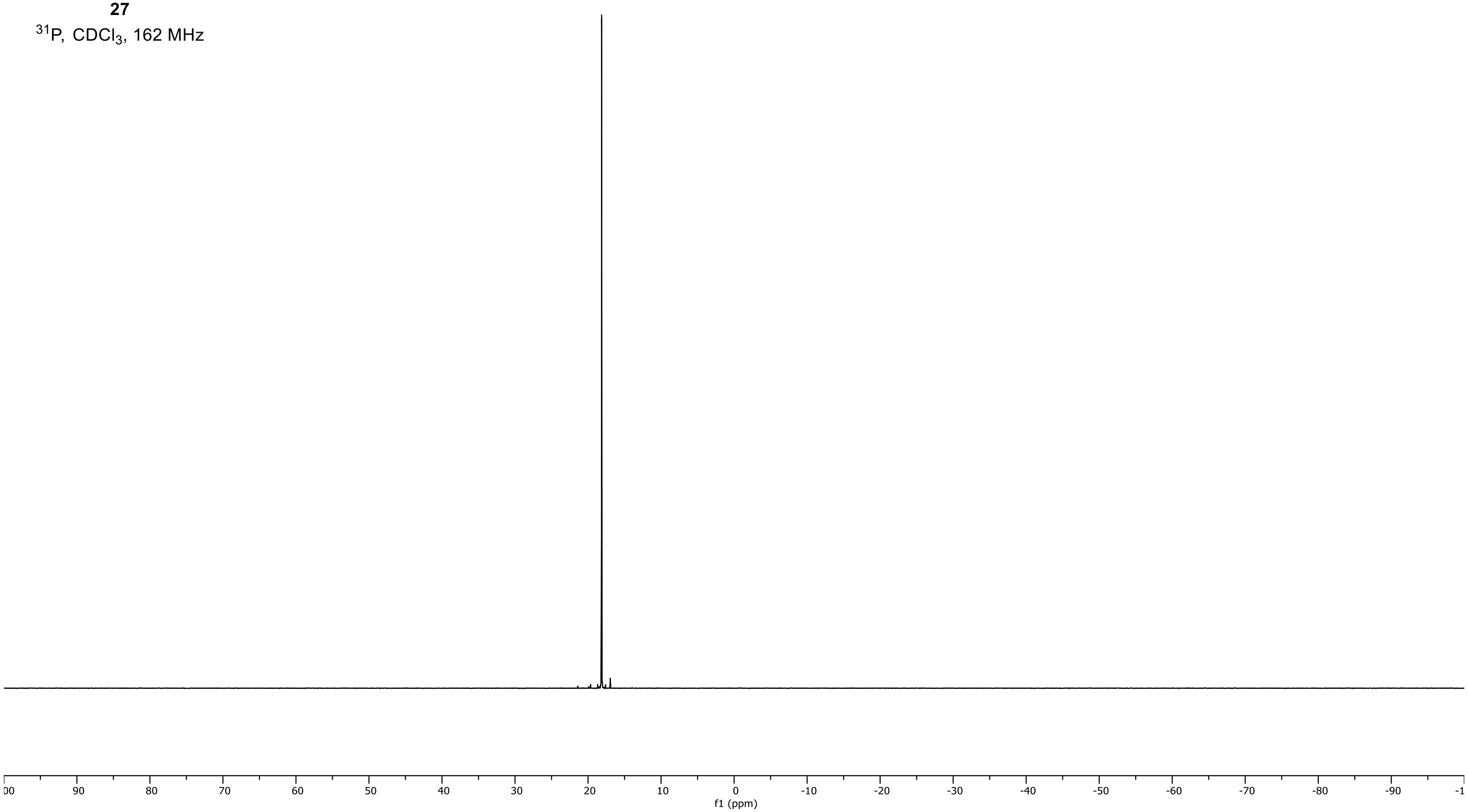

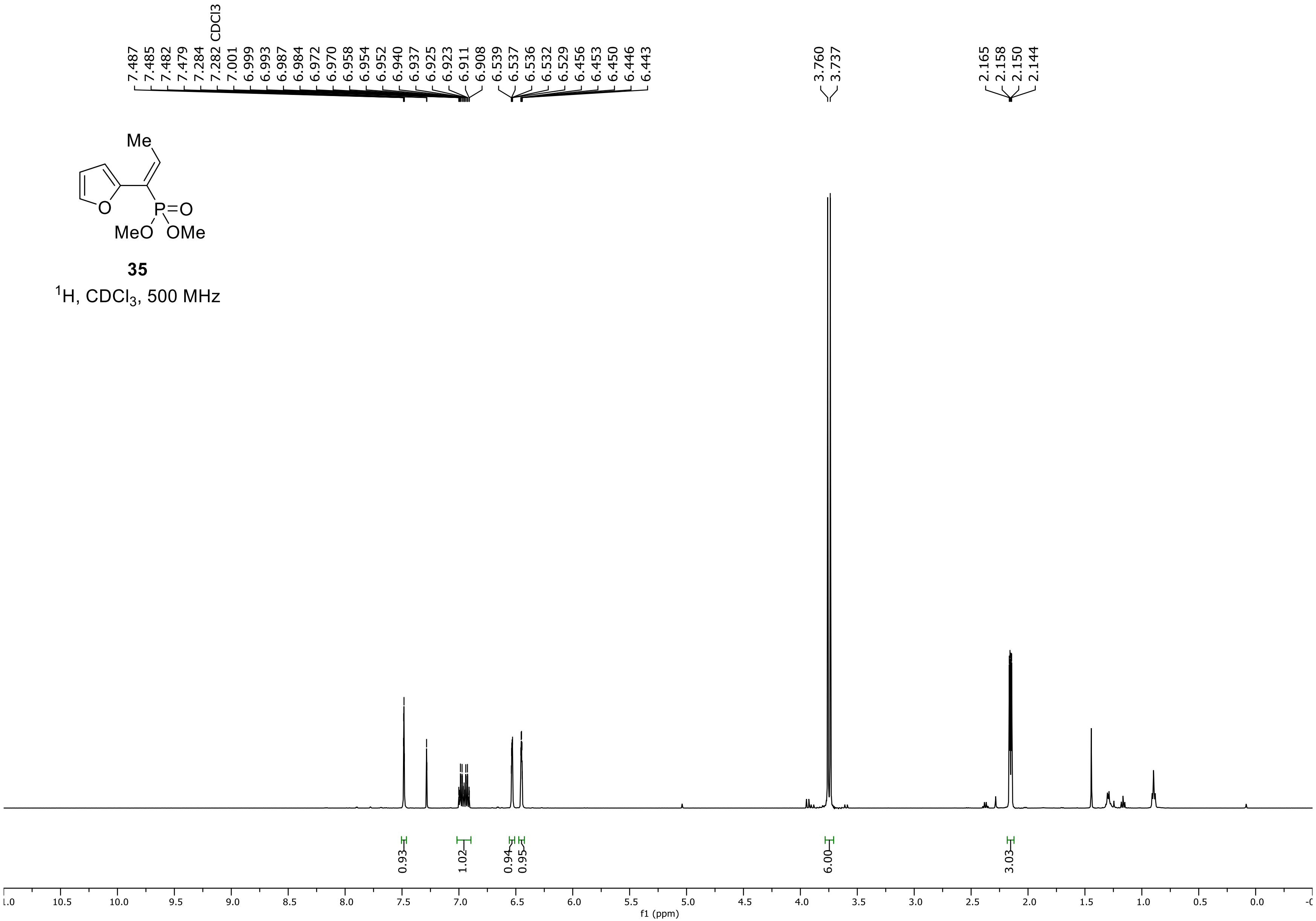

7.487 7.485 7.482 7.479 7.284 7.282 CDCl3 7.001 6.999 6.993 6.987 6.984 6.972 6.970 6.958 6.954 6.952 6.940 6.937 6.925 6.923 6.911 6.908 6.539 6.537 6.536 6.532 6.529 6.456 6.453 6.450 6.446 6.443
3.760 3.737
2.165 2.158 2.150 2.144
Me
O
P=O
MeO OMe
35
^{1}H, CDCl$_3$, 500 MHz
0.93 1.02 0.94 0.95 6.00 3.03
f1 (ppm)

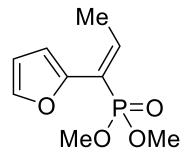

35

^{13}C, $CDCl_3$, 126 MHz

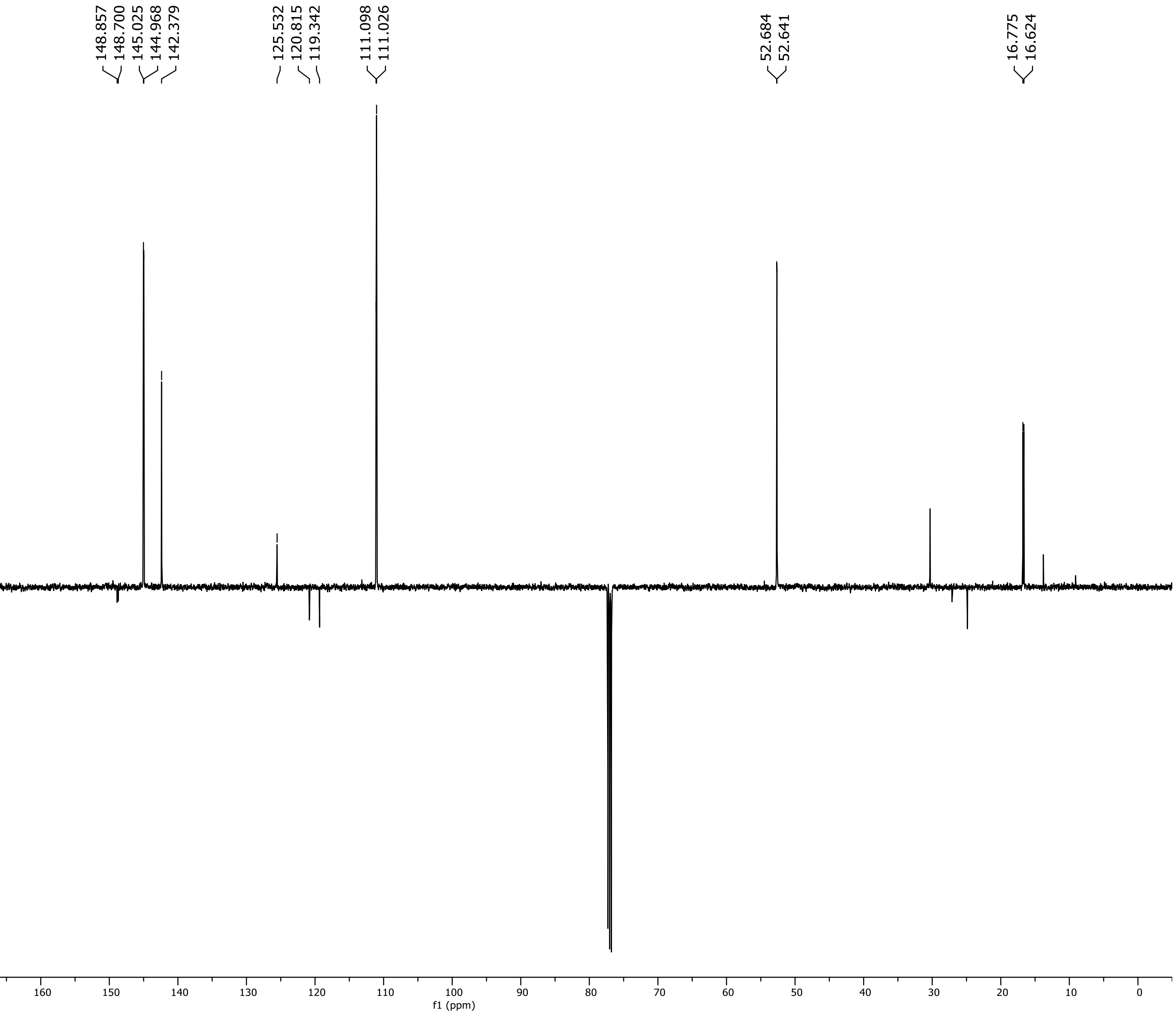

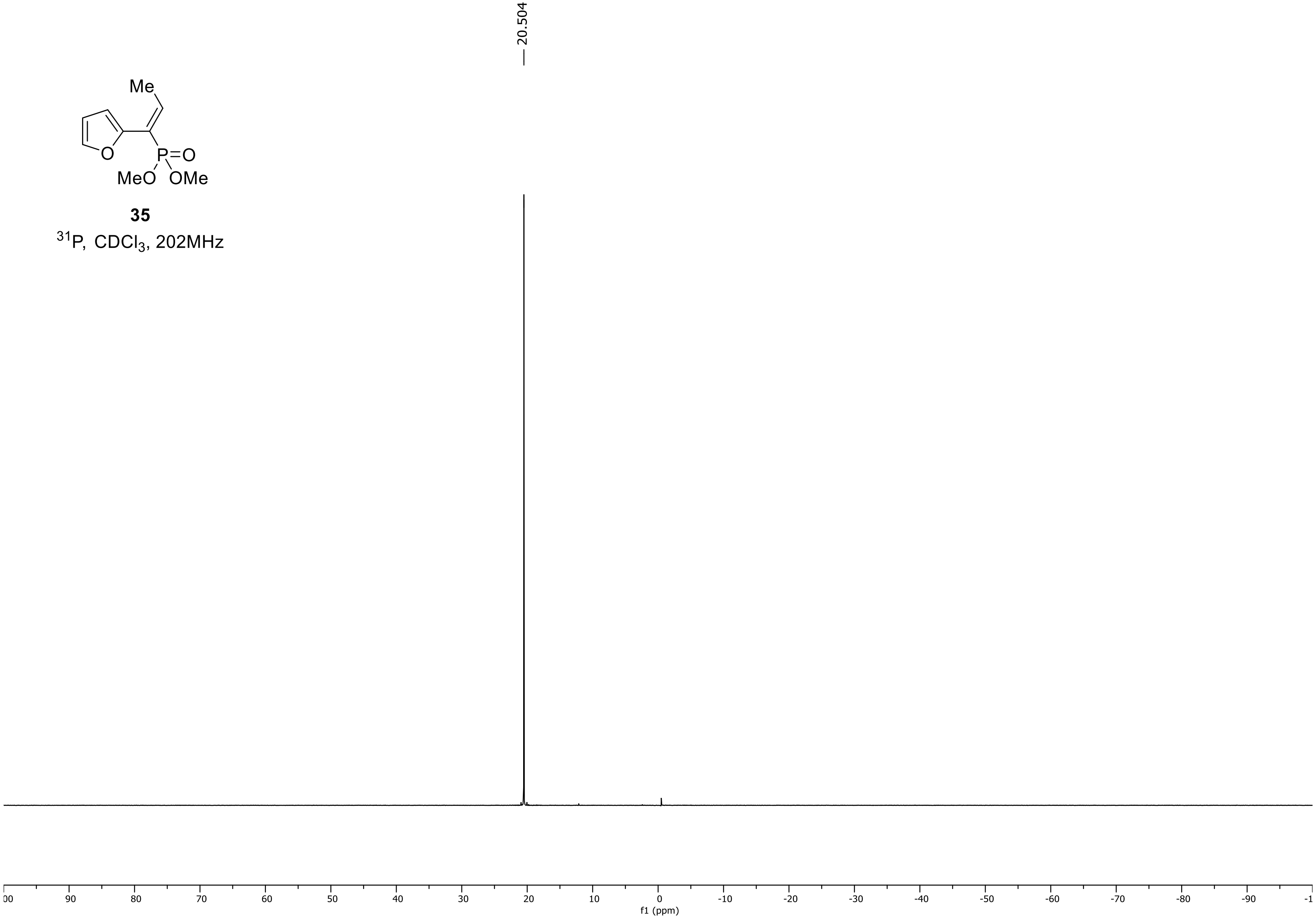

35

^{31}P, $CDCl_3$, 202MHz

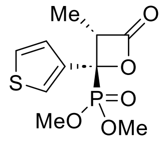

28

^{1}H, $CDCl_3$, 500 MHz

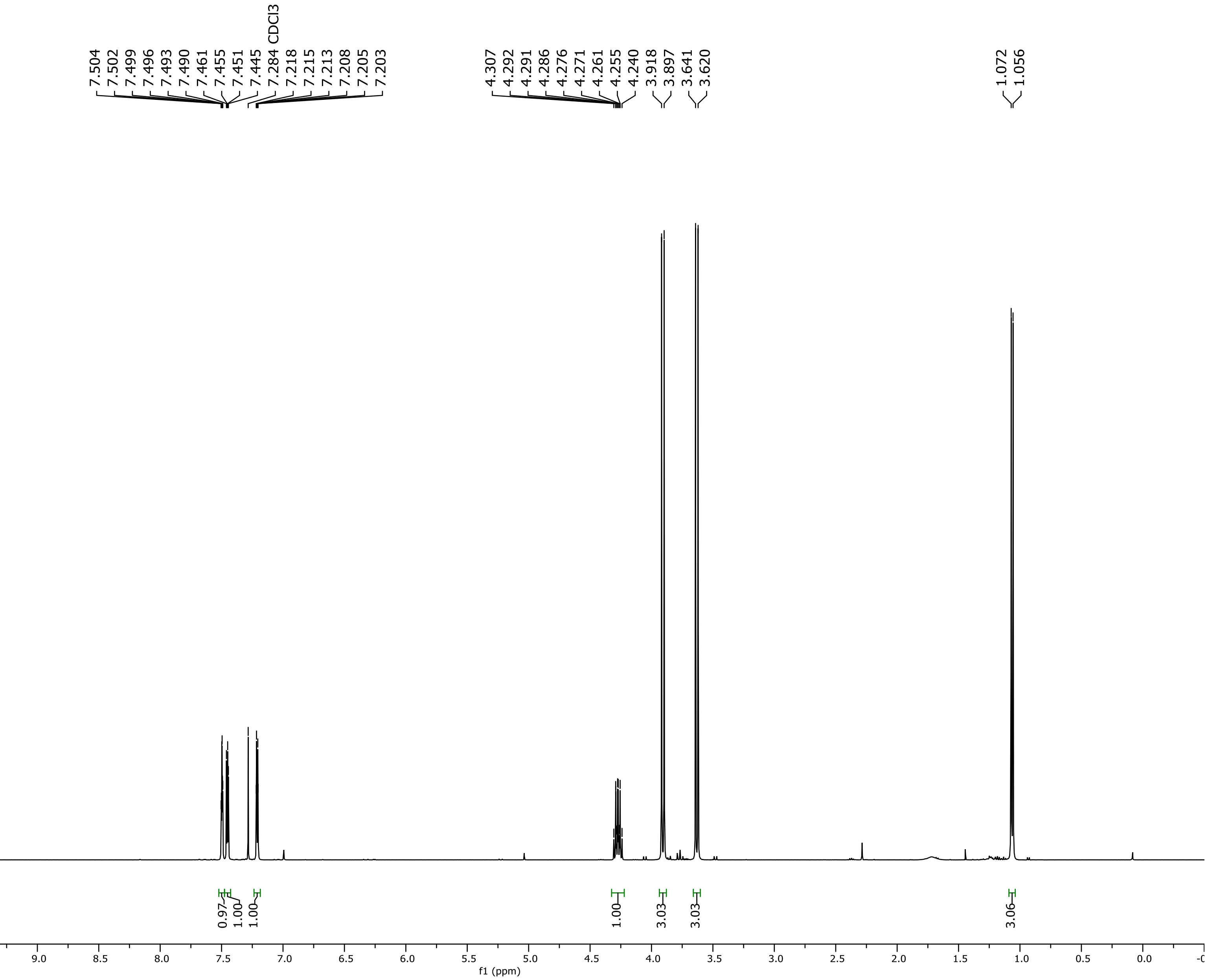

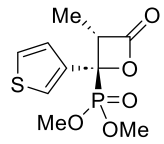

28

^{13}C, $CDCl_3$, 126 MHz

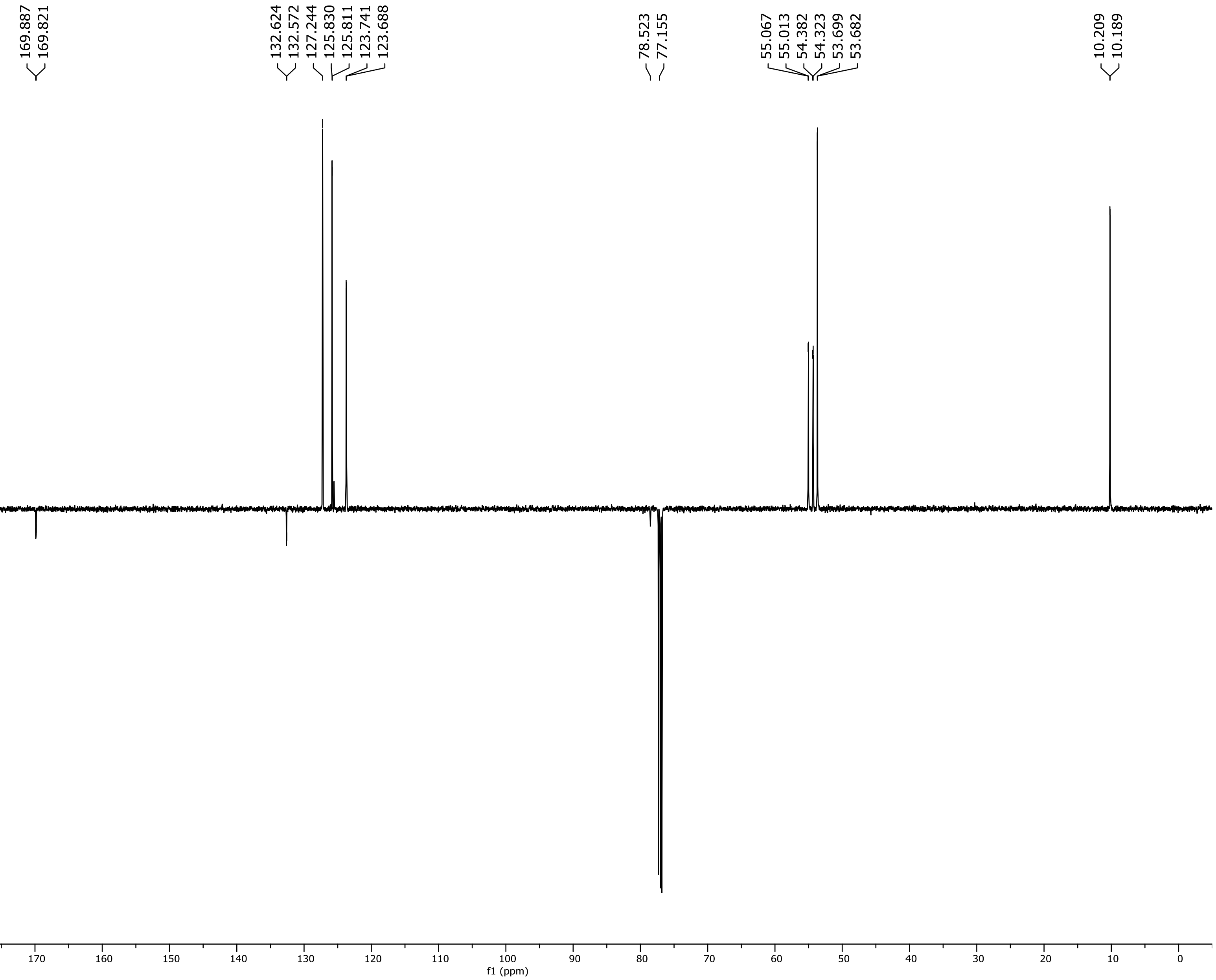

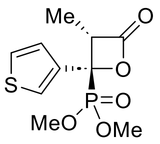

28

^{31}P, $CDCl_3$, 202 MHz

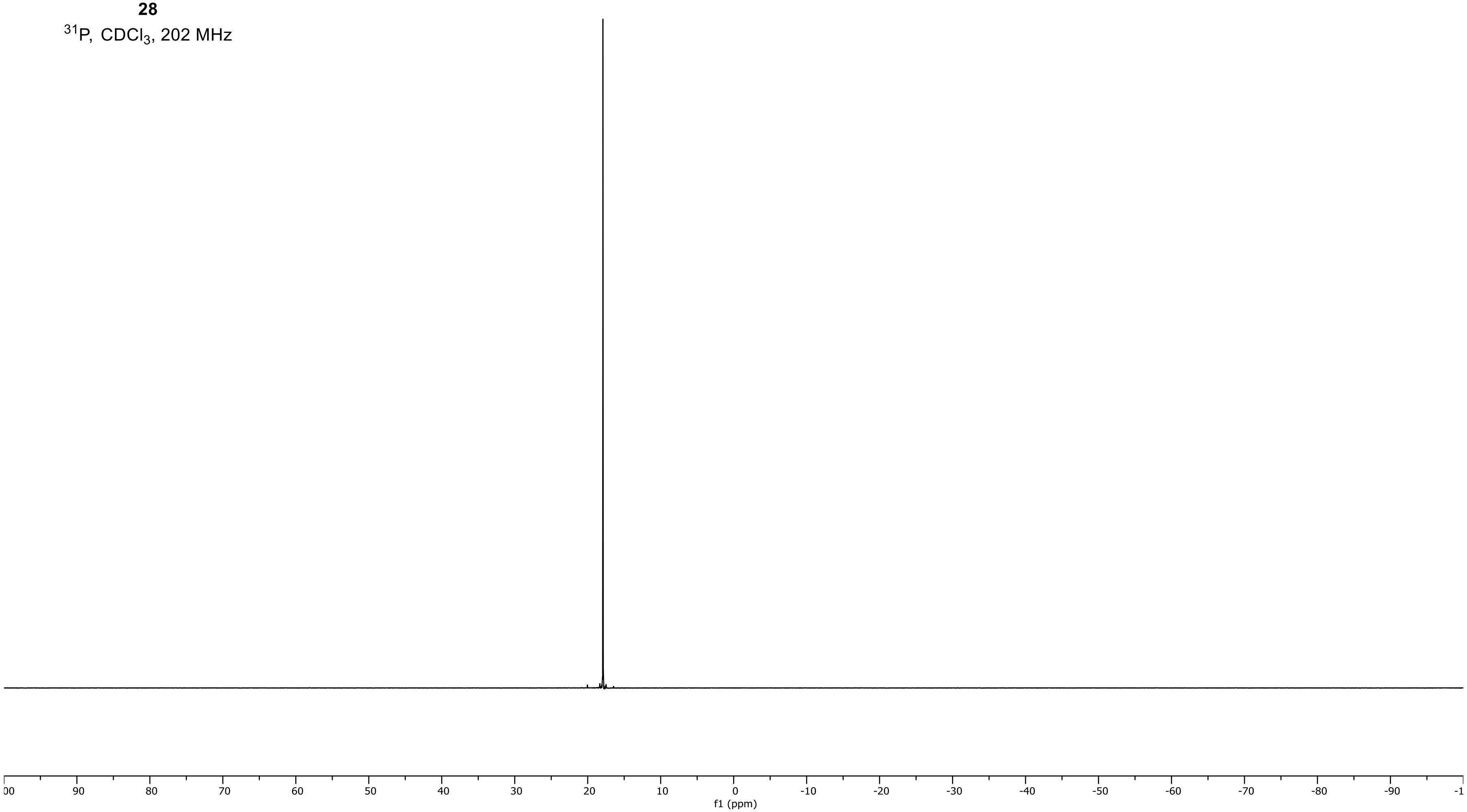

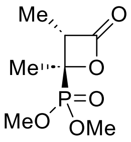

29

^{1}H, $CDCl_3$, 400 MHz

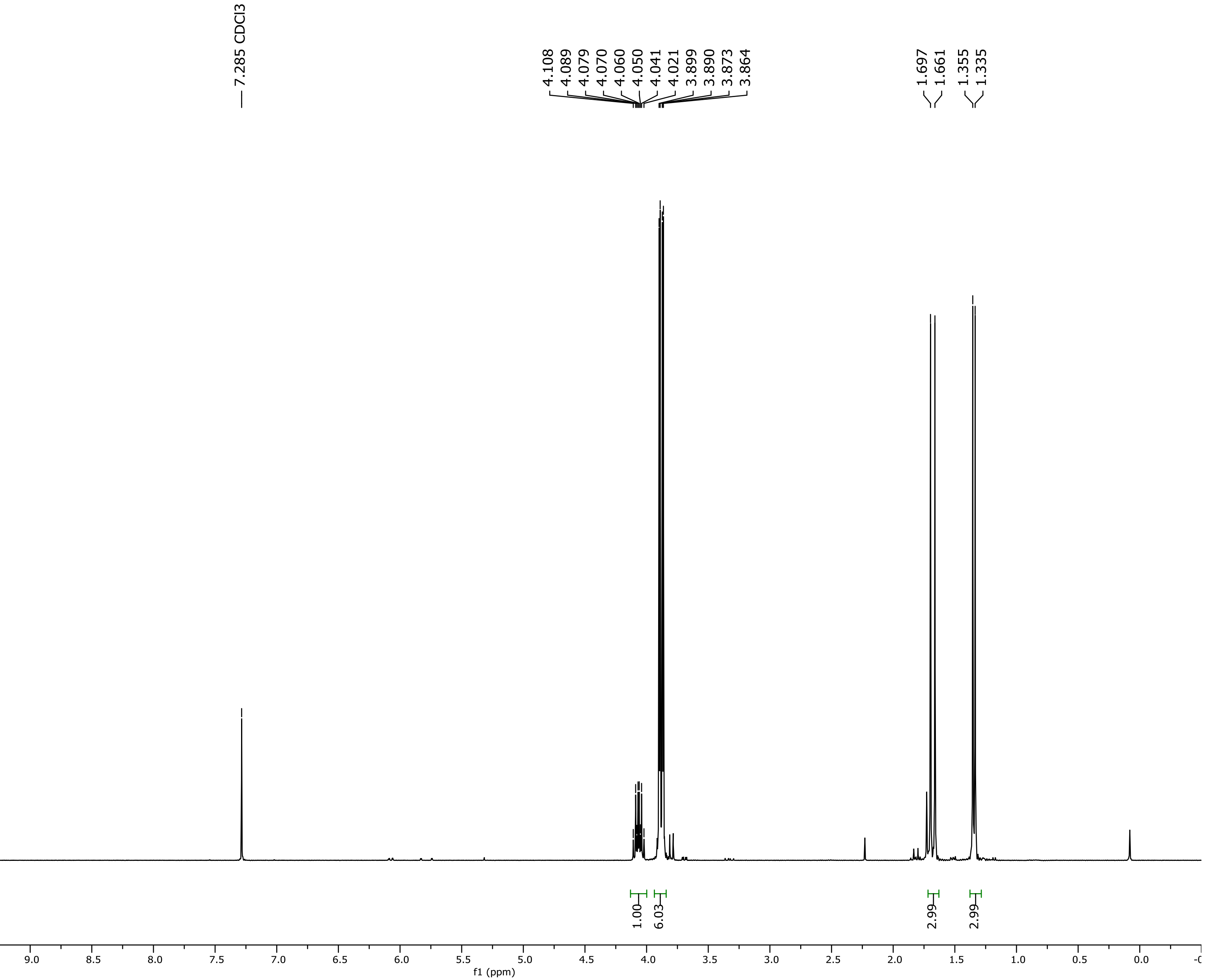

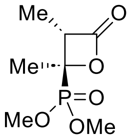

29

^{13}C, $CDCl_3$, 101 MHz

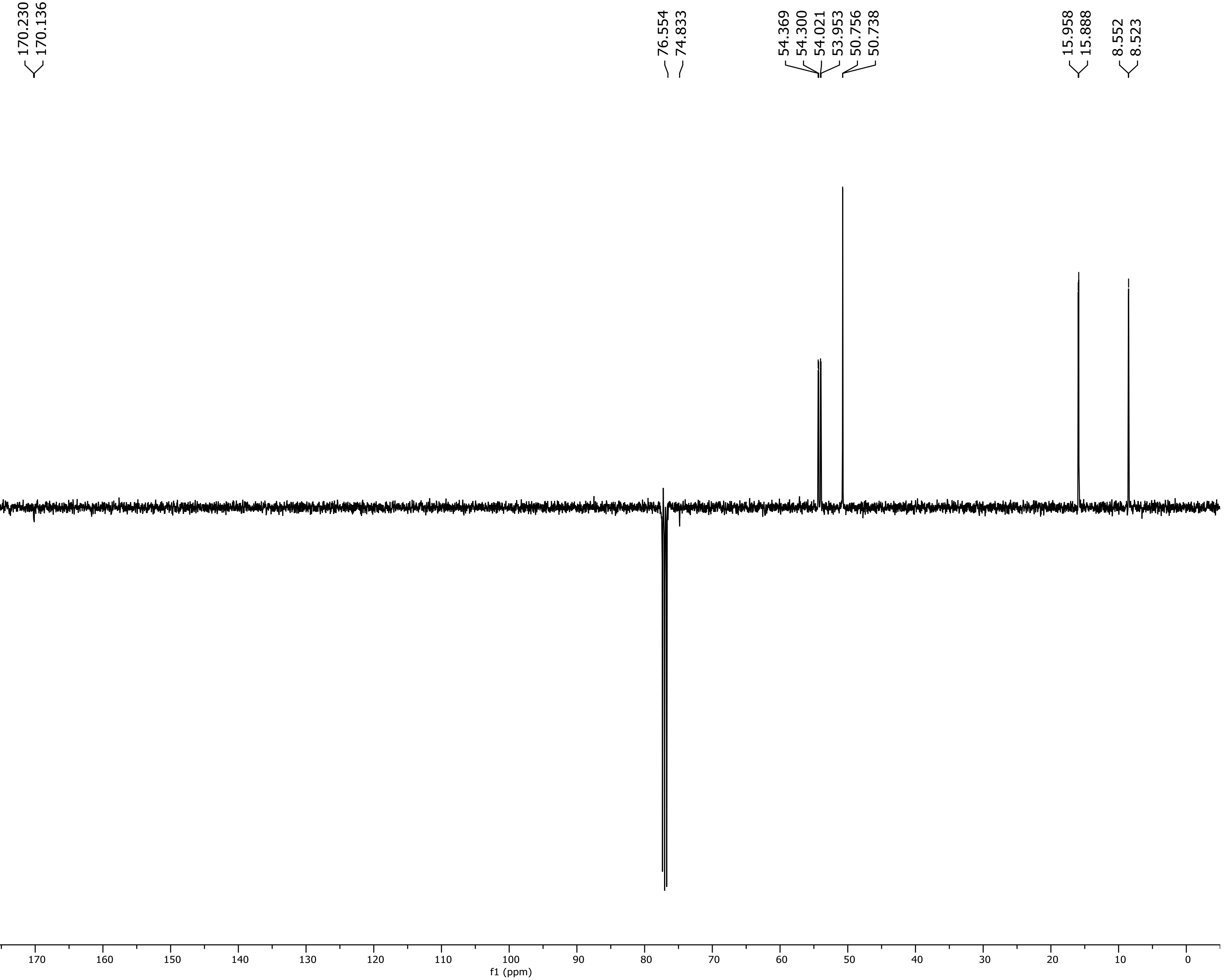

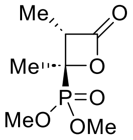

29

^{31}P, $CDCl_3$, 162 MHz

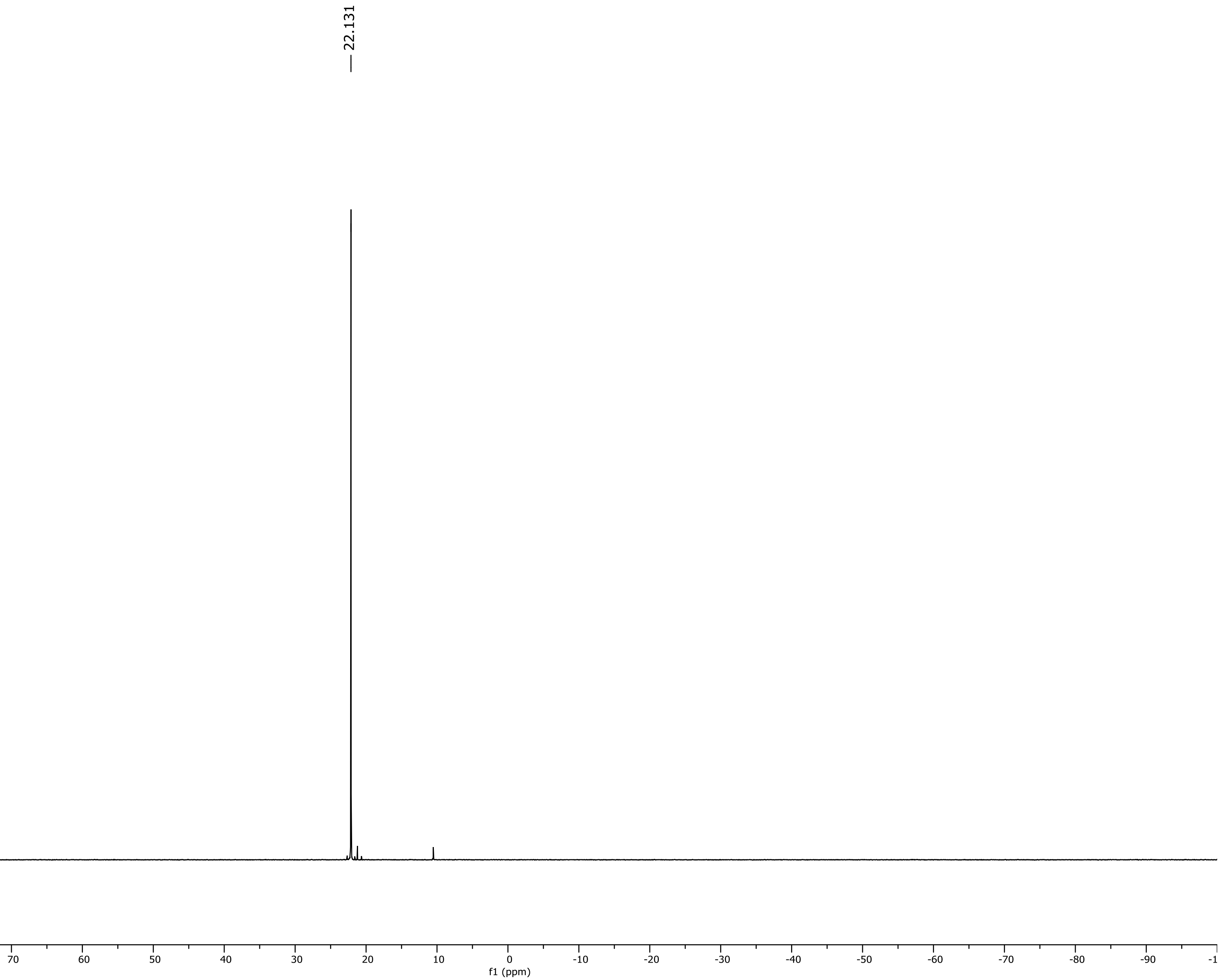

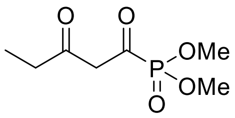

S36

^1H, $CDCl_3$, 500 MHz

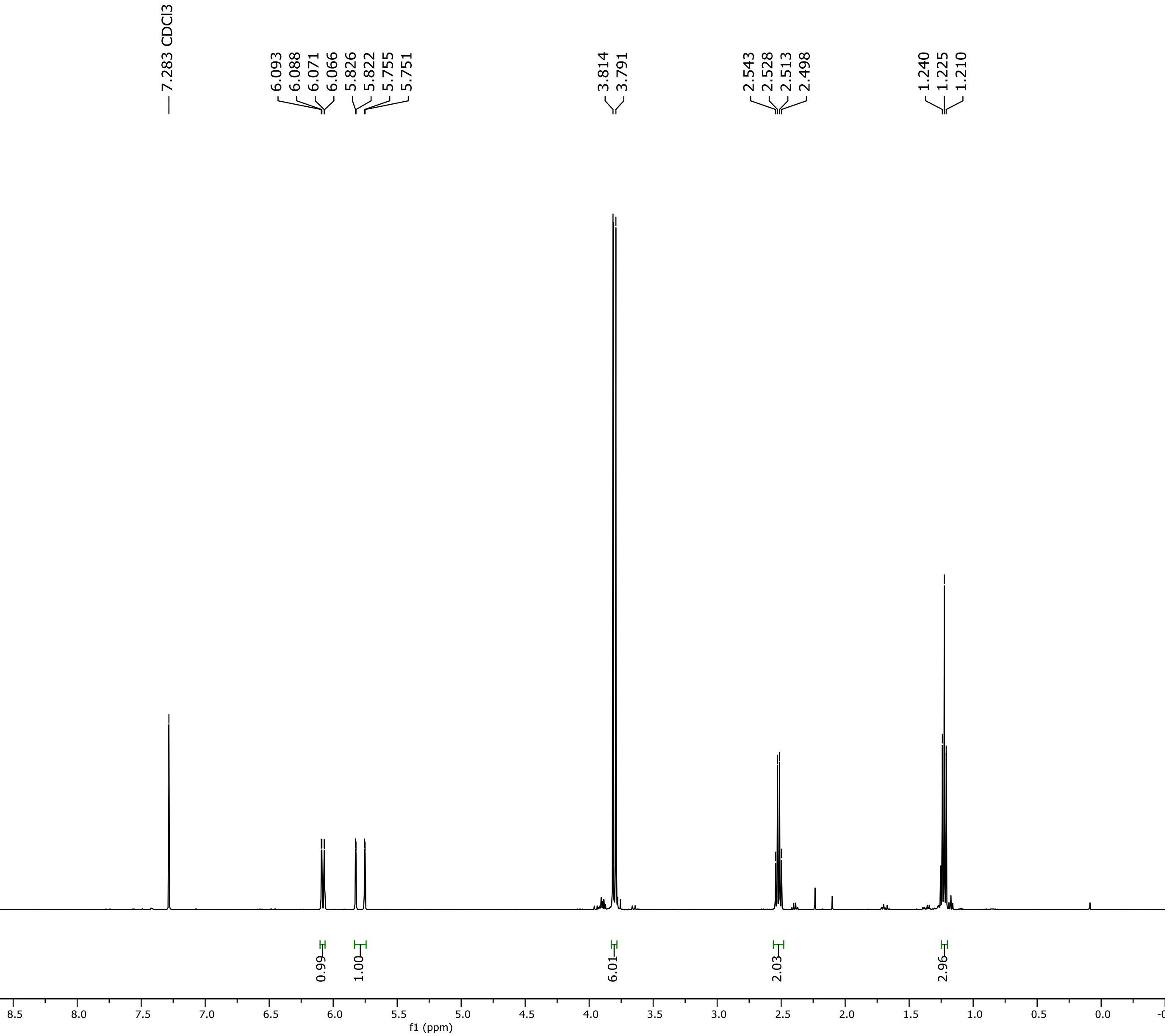

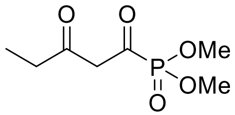

S36

^{13}C, $CDCl_3$, 126 MHz

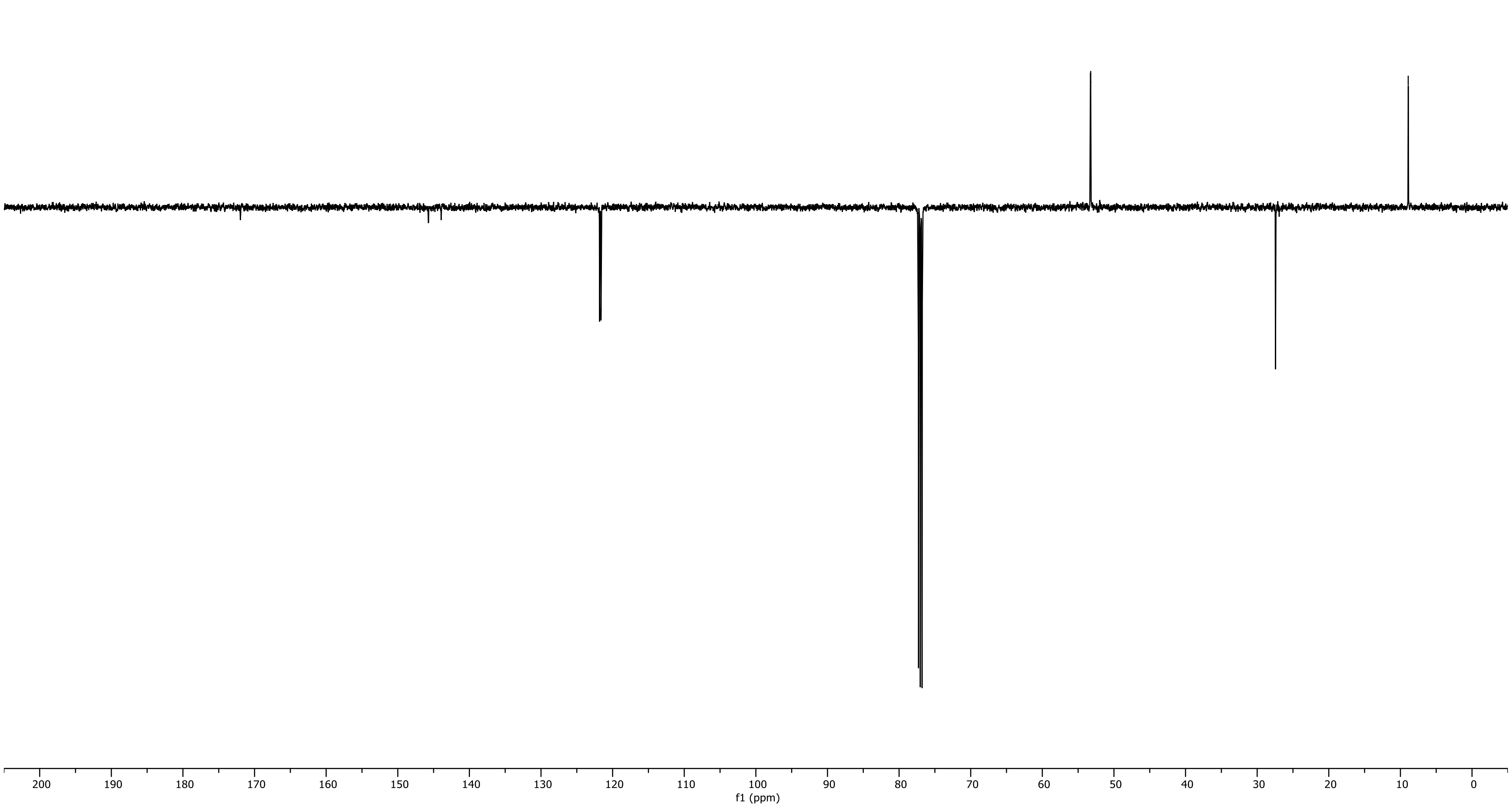

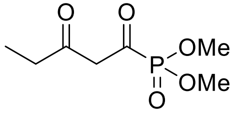

S36

^{31}P, $CDCl_3$, 162 MHz

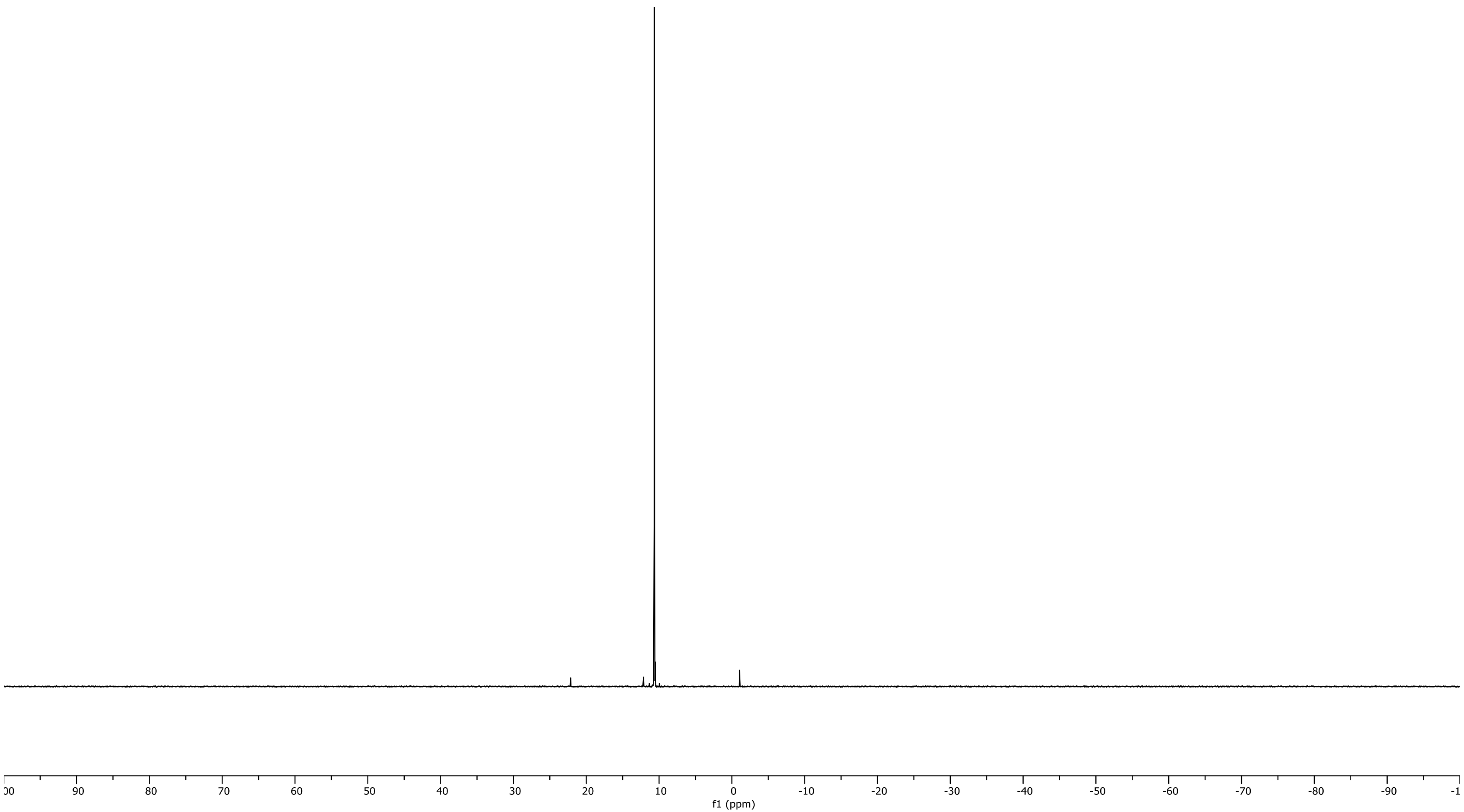

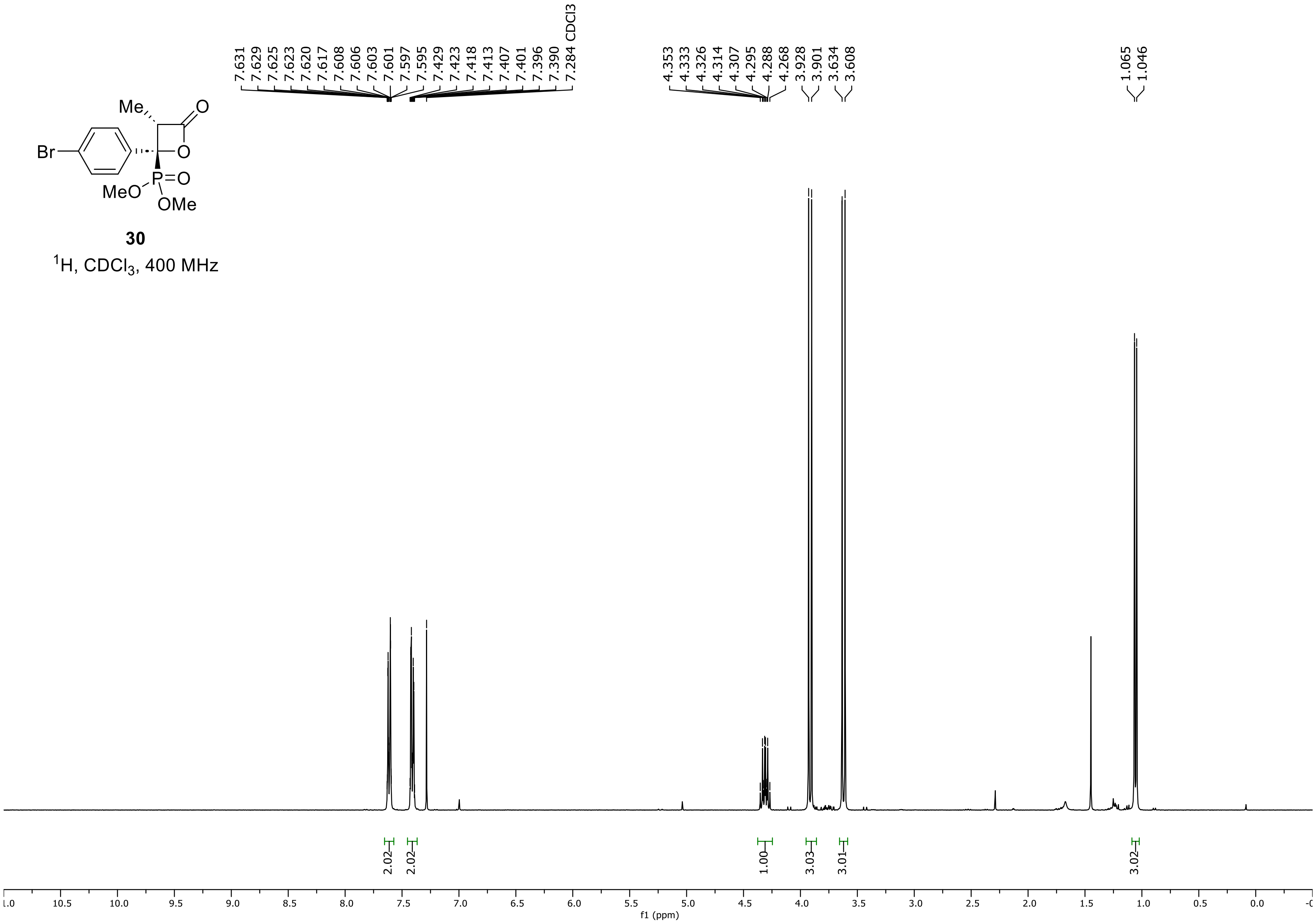

30

^{1}H, $CDCl_3$, 400 MHz

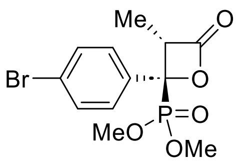

30

^{13}C, $CDCl_3$, 101 MHz

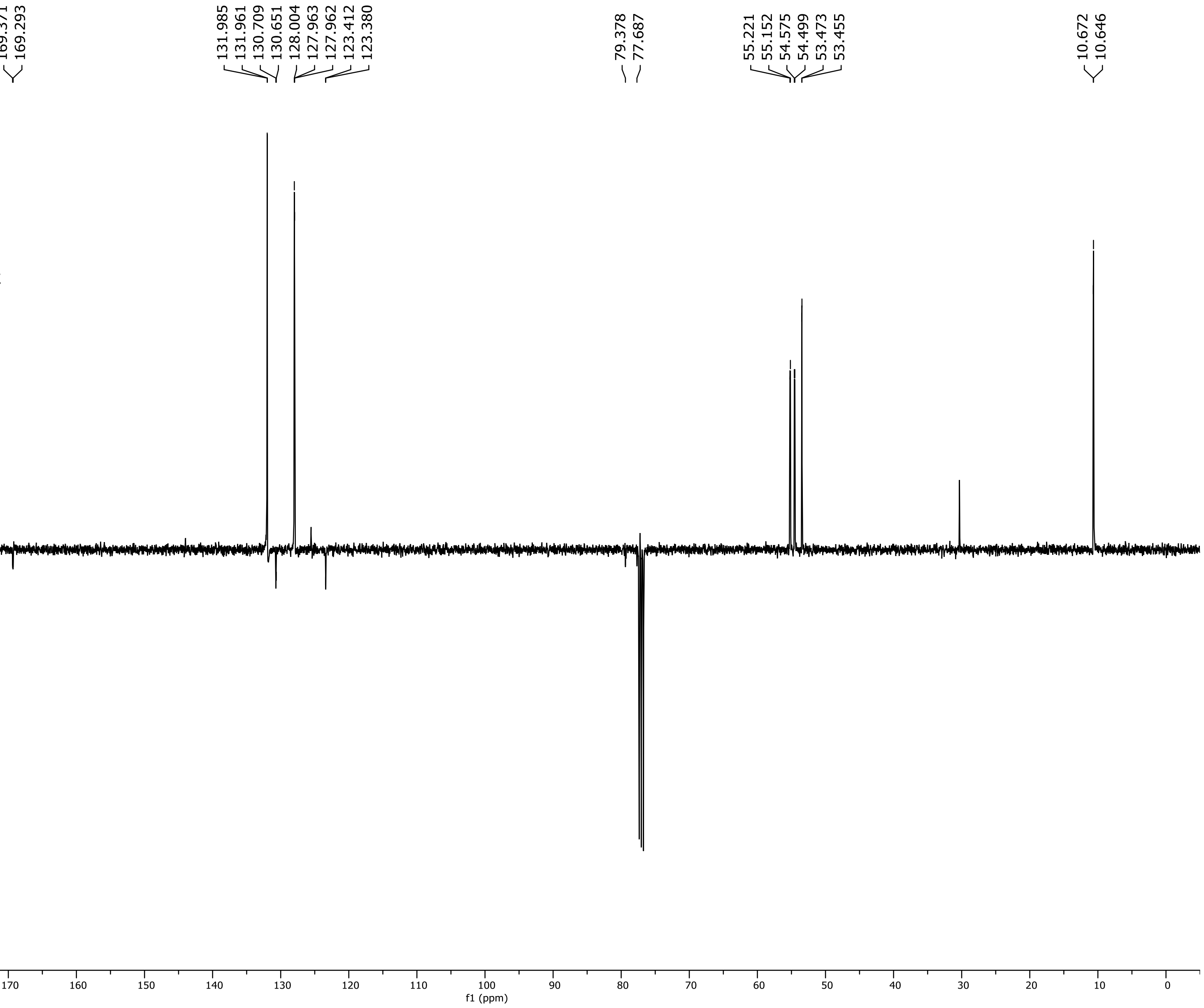

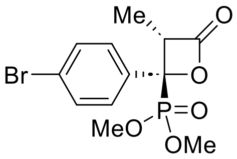

30

^{31}P, $CDCl_3$, 162 MHz

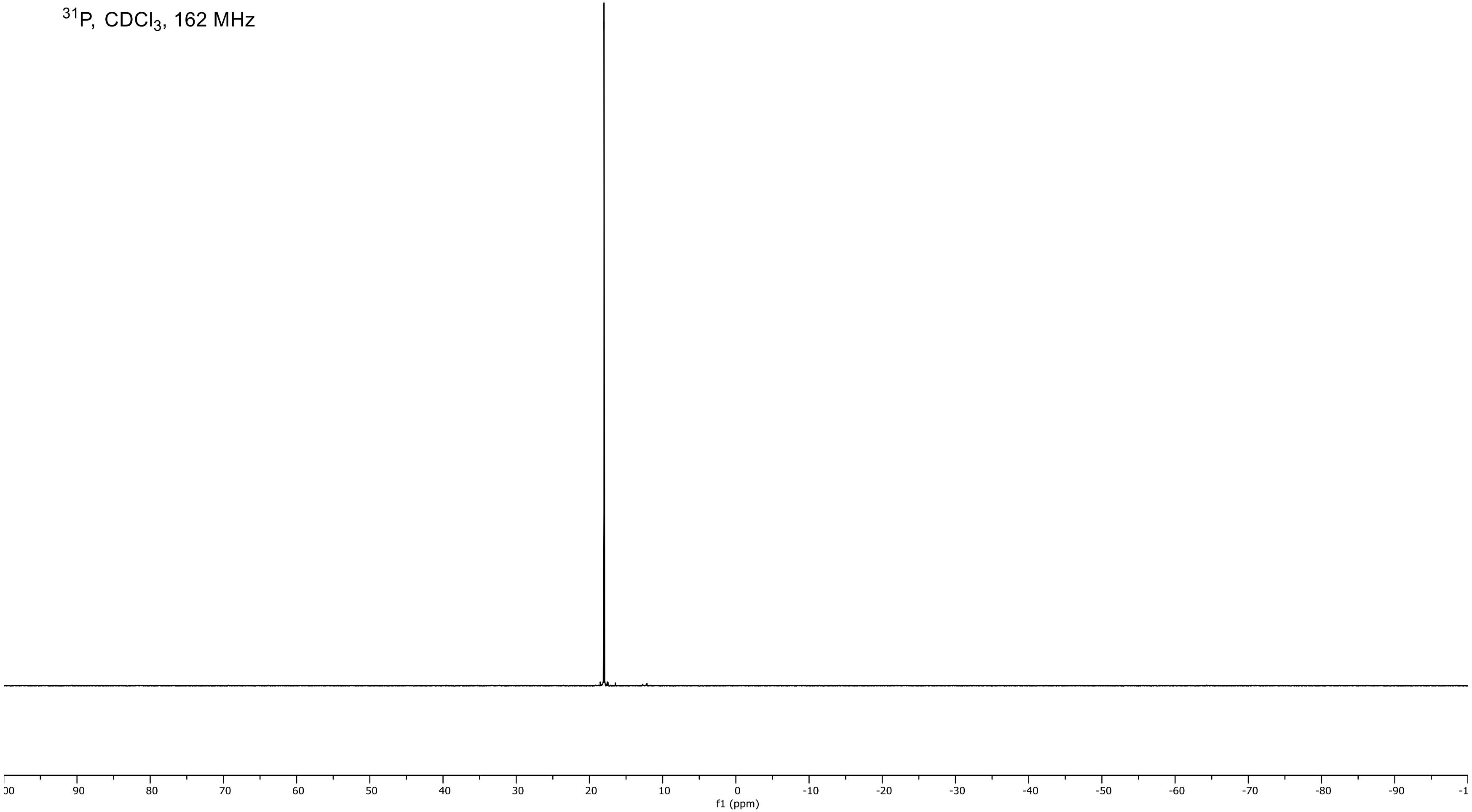

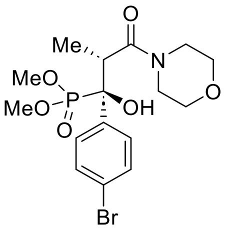

31

^{1}H, $CDCl_3$, 500 MHz

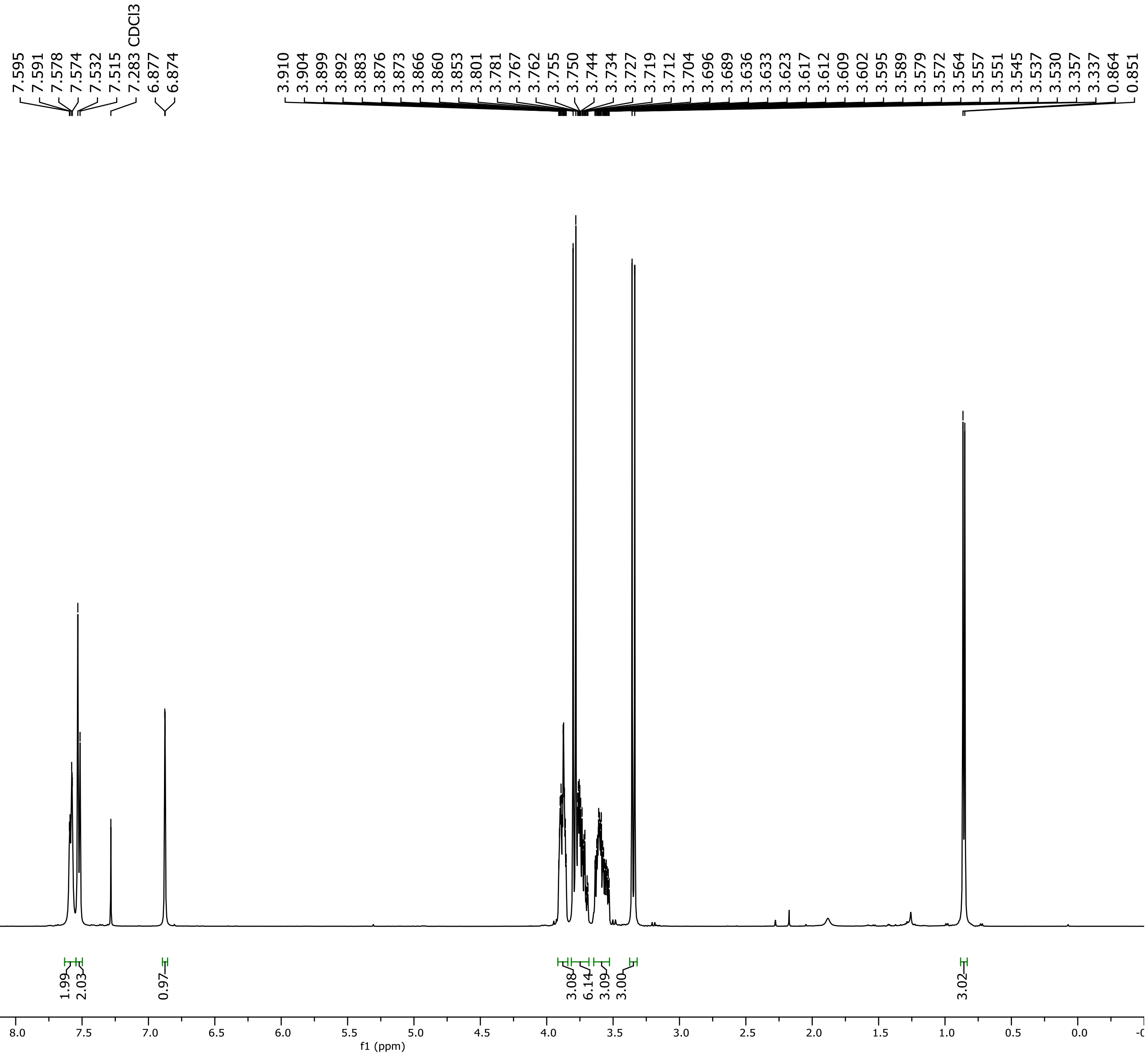

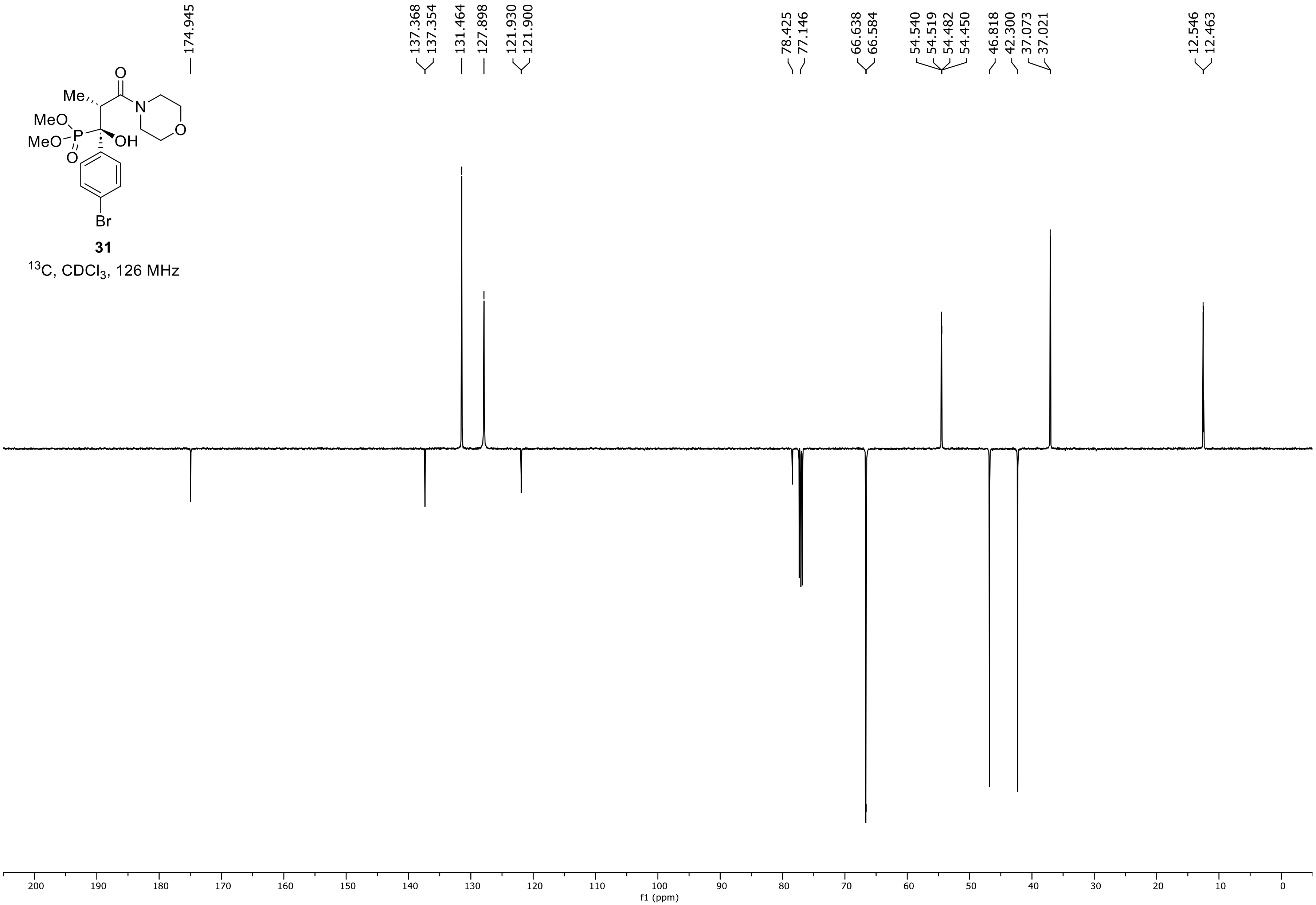
174.945
137.368
137.354
131.464
127.898
121.930
121.900
78.425
77.146
66.638
66.584
54.540
54.519
54.482
54.450
46.818
42.300
37.073
37.021
12.546
12.463
Me
MeO
MeO
P
O
OH
N
O
Br
31
^{13}C, CDCl$_3$, 126 MHz
200 190 180 170 160 150 140 130 120 110 100 90 80 70 60 50 40 30 20 10 0
f1 (ppm)

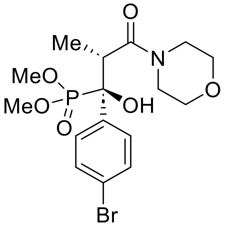

31

^{31}P, $CDCl_3$, 162 MHz

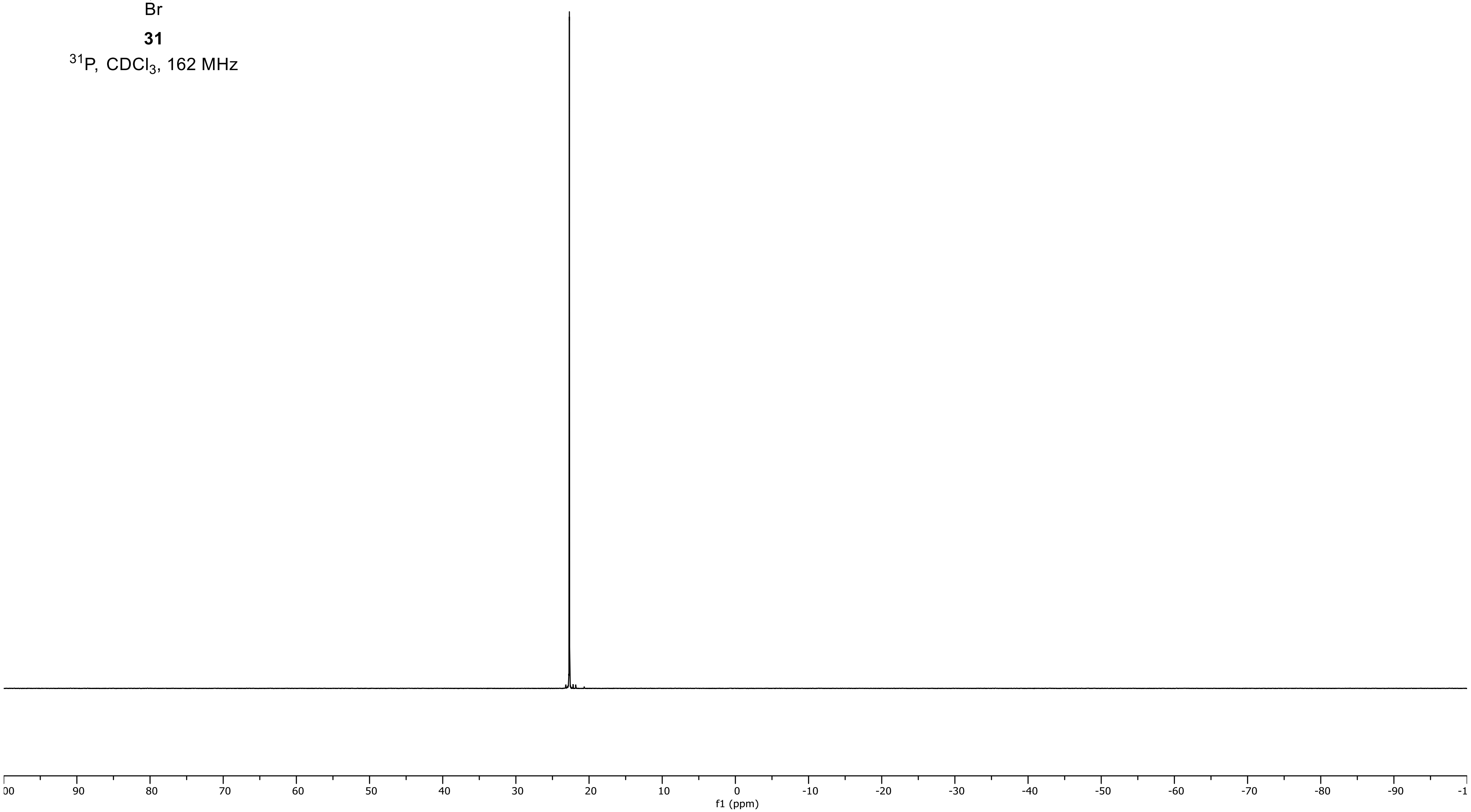

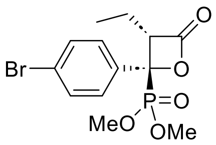

36

^1H, $CDCl_3$, 400 MHz

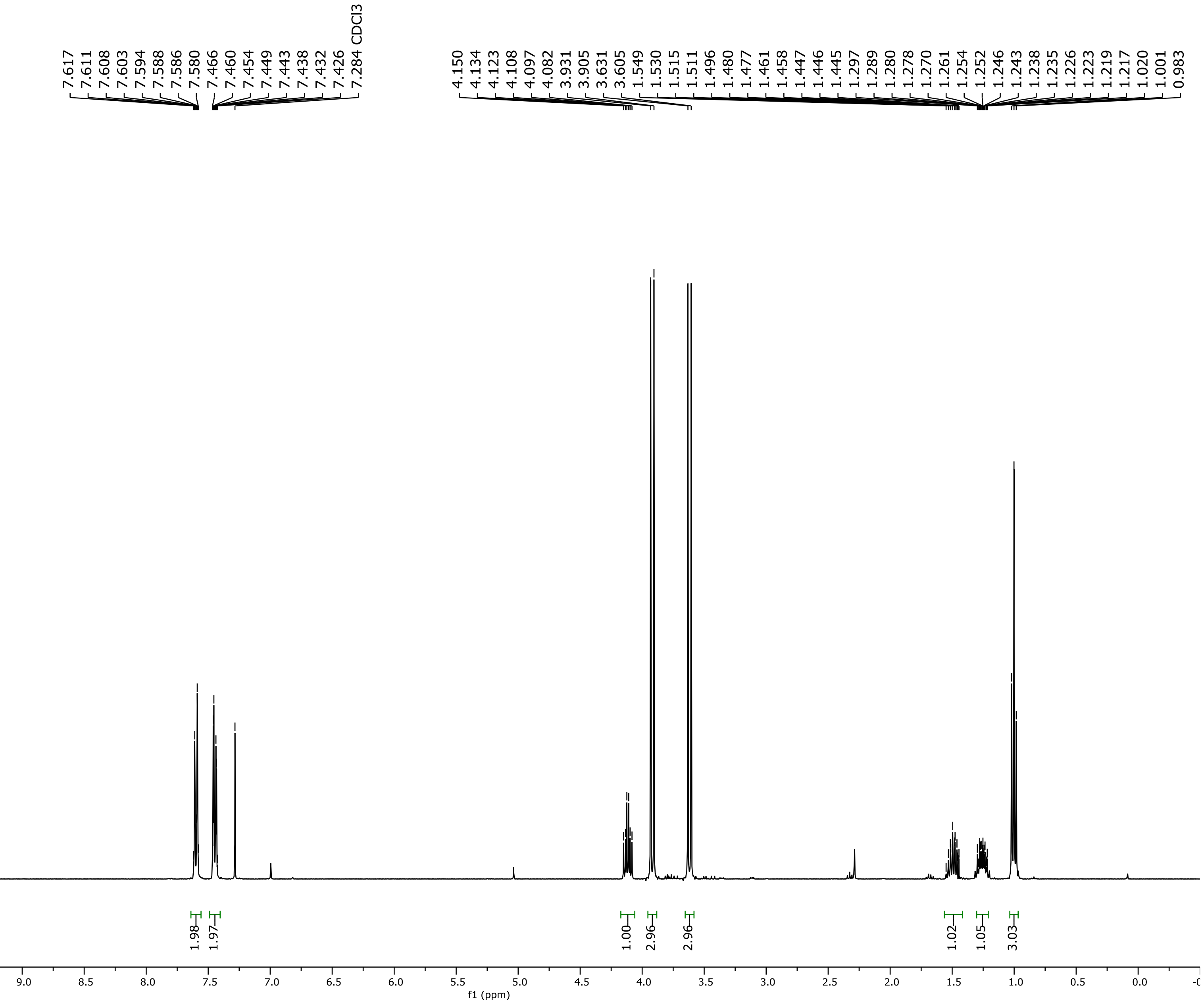

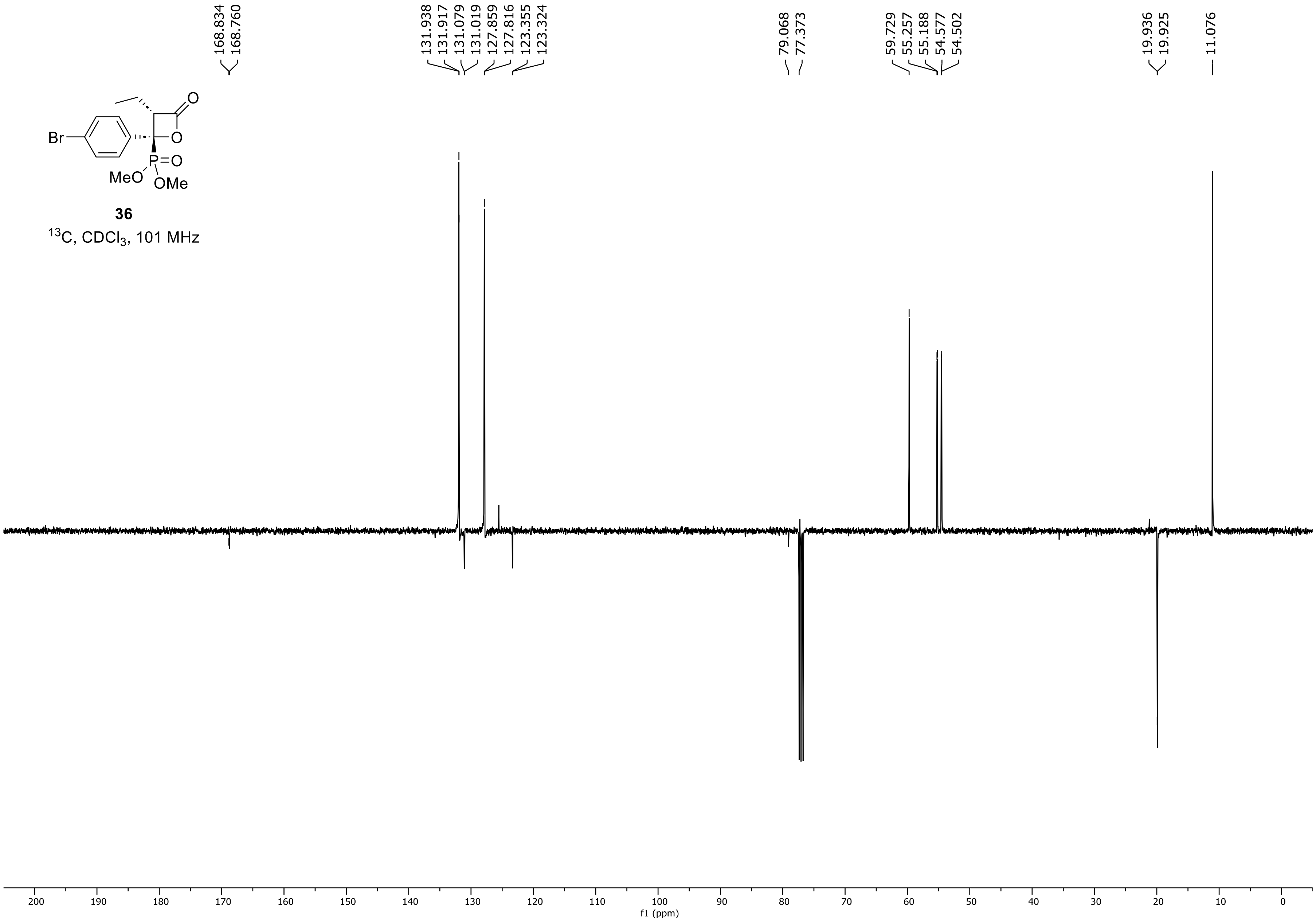

36

^{13}C, $CDCl_3$, 101 MHz

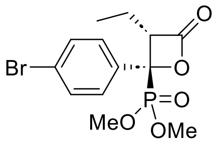

36

^{31}P, $CDCl_3$, 162 MHz

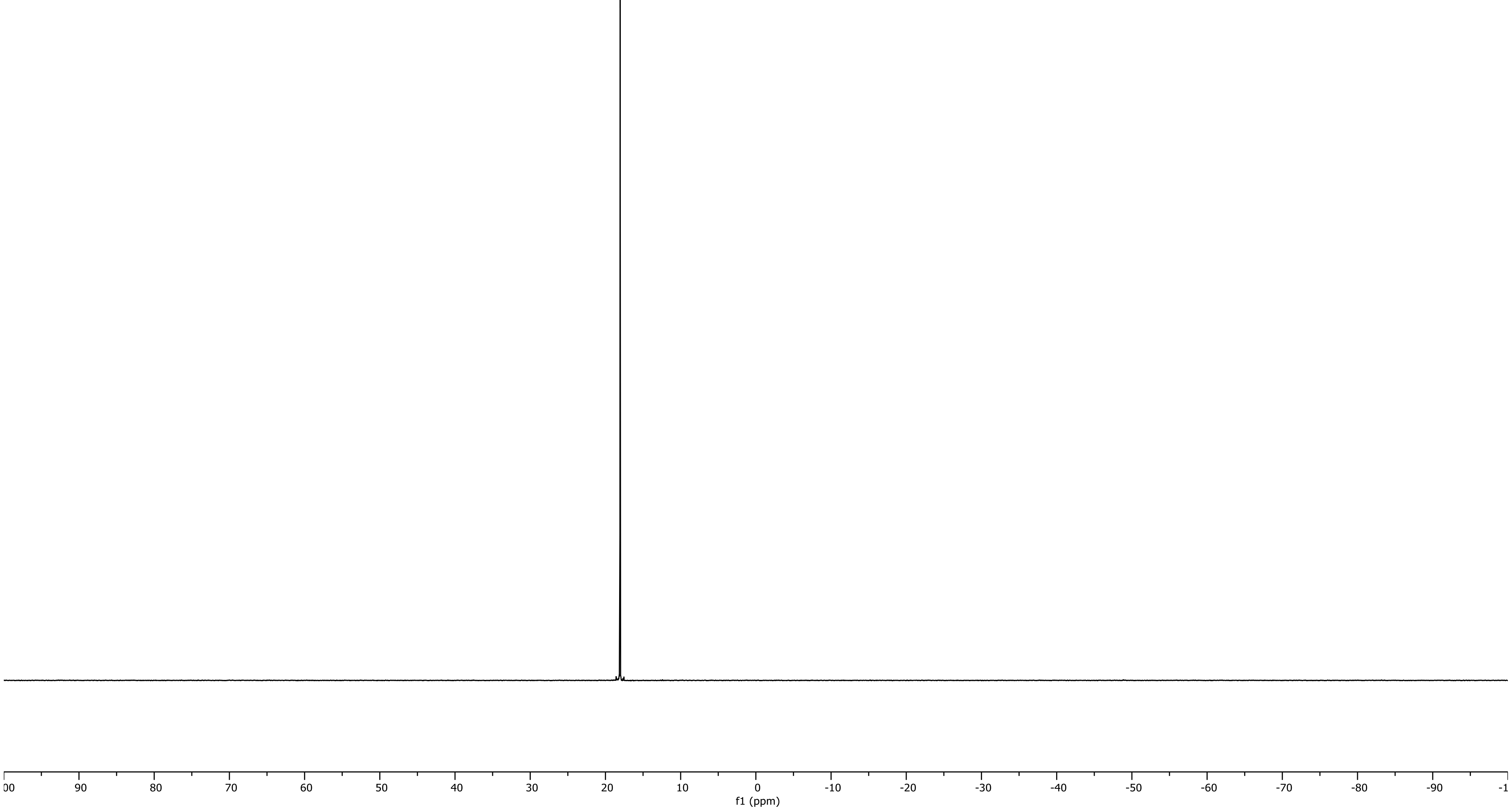

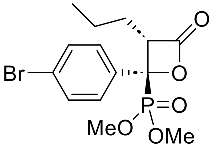

37

^{1}H, $CDCl_3$, 500 MHz

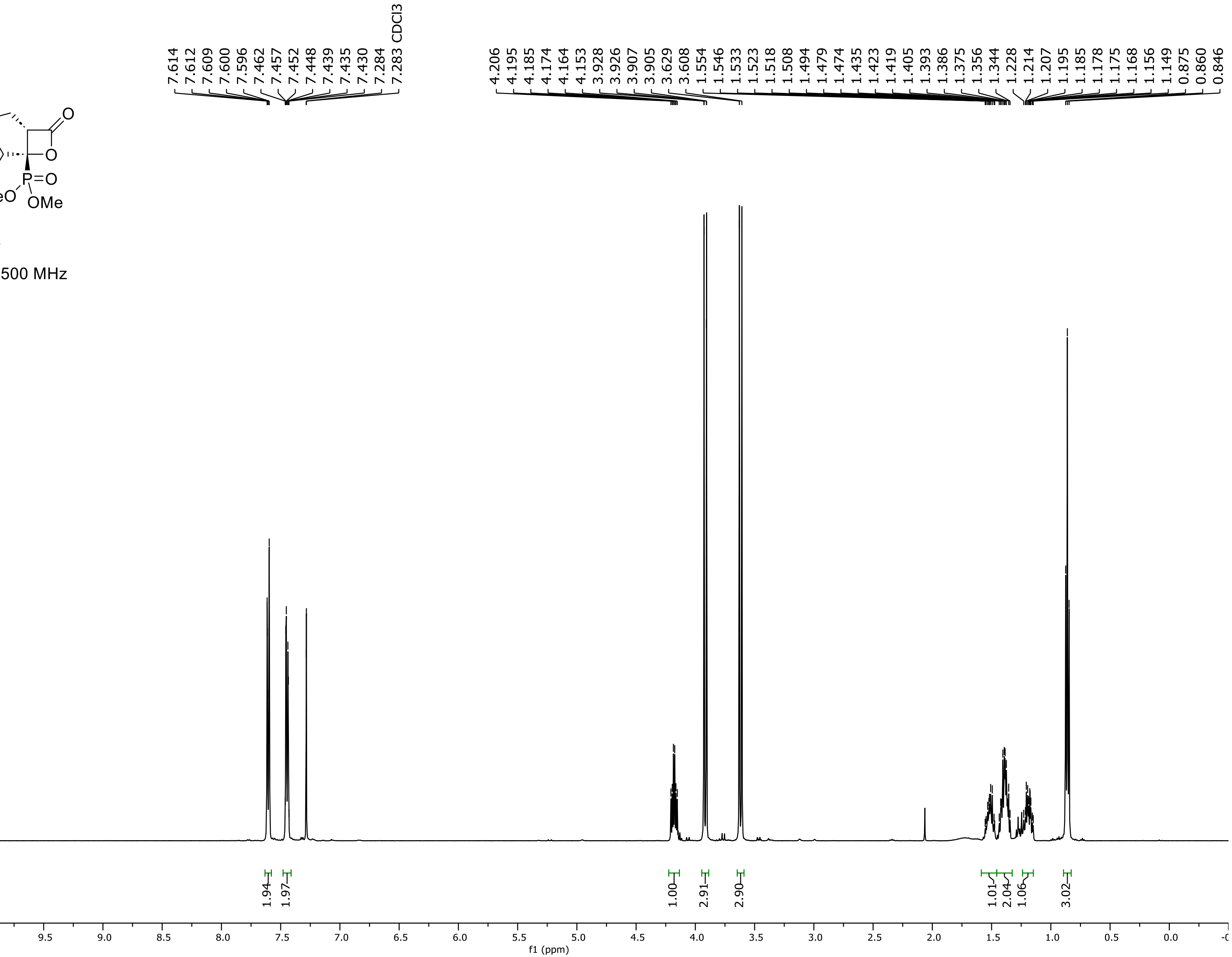

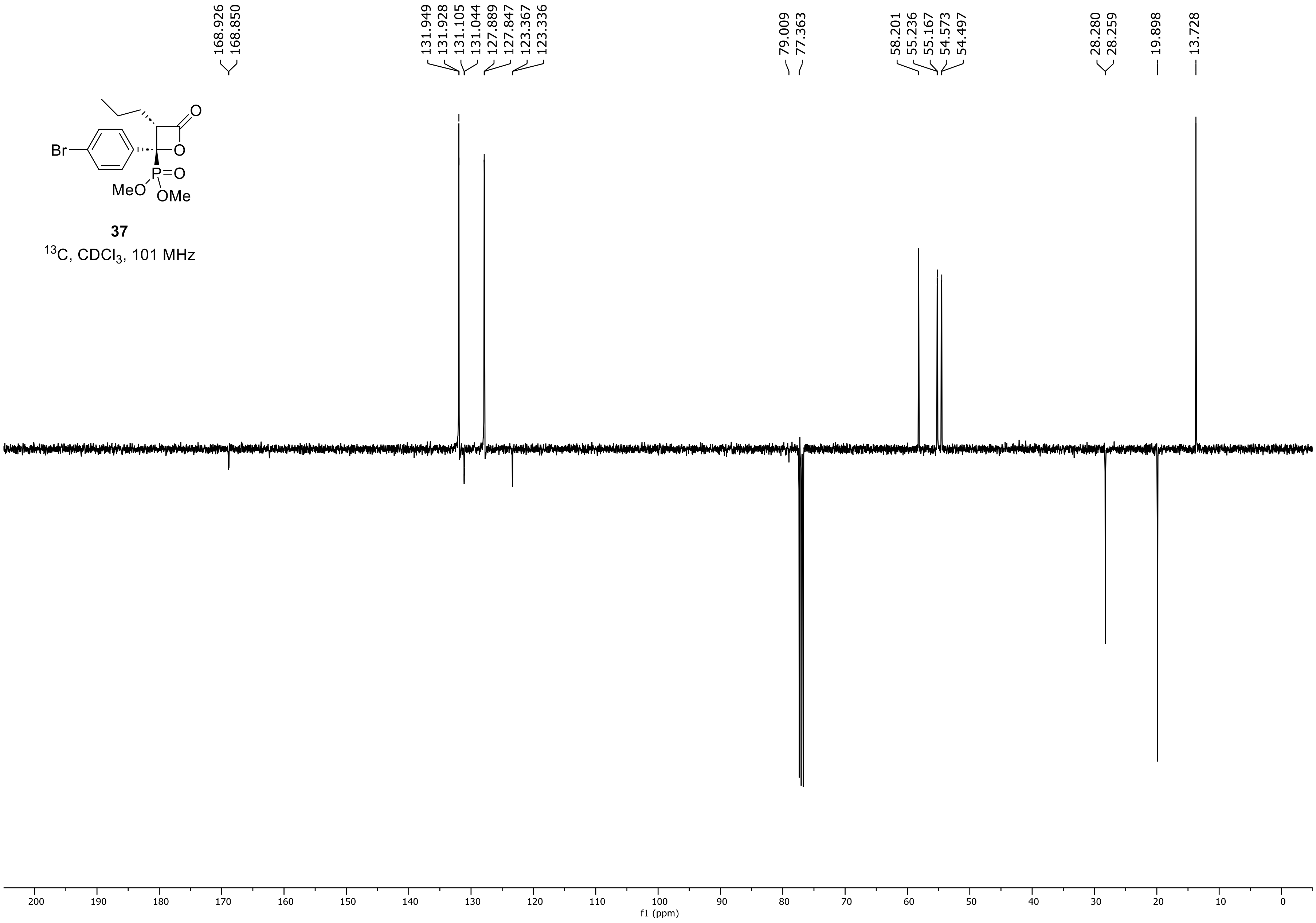

37

^{13}C, $CDCl_3$, 101 MHz

37

^{31}P, $CDCl_3$, 202 MHz

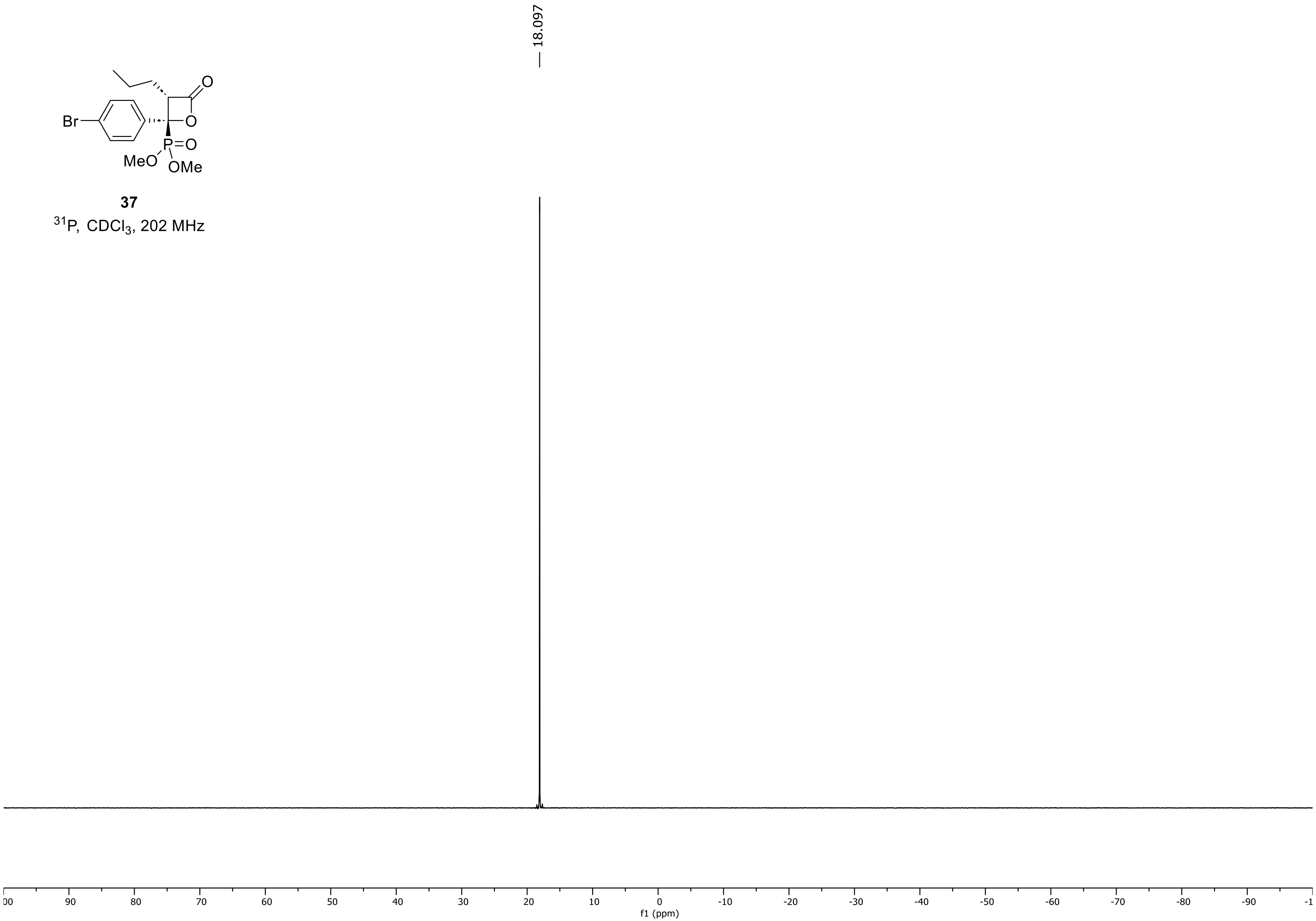

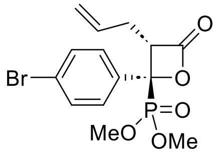

38

^{1}H, $CDCl_3$, 400 MHz

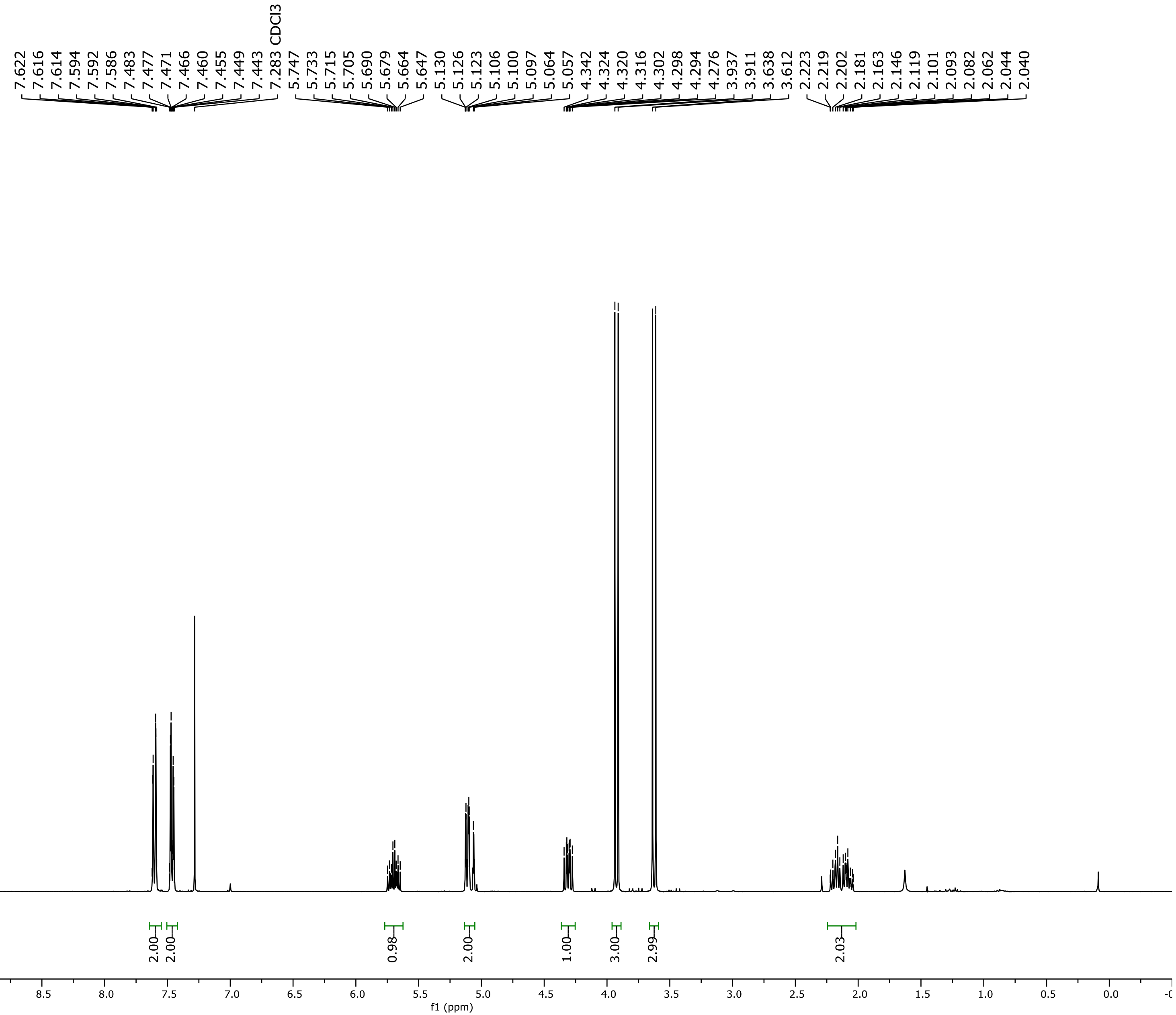

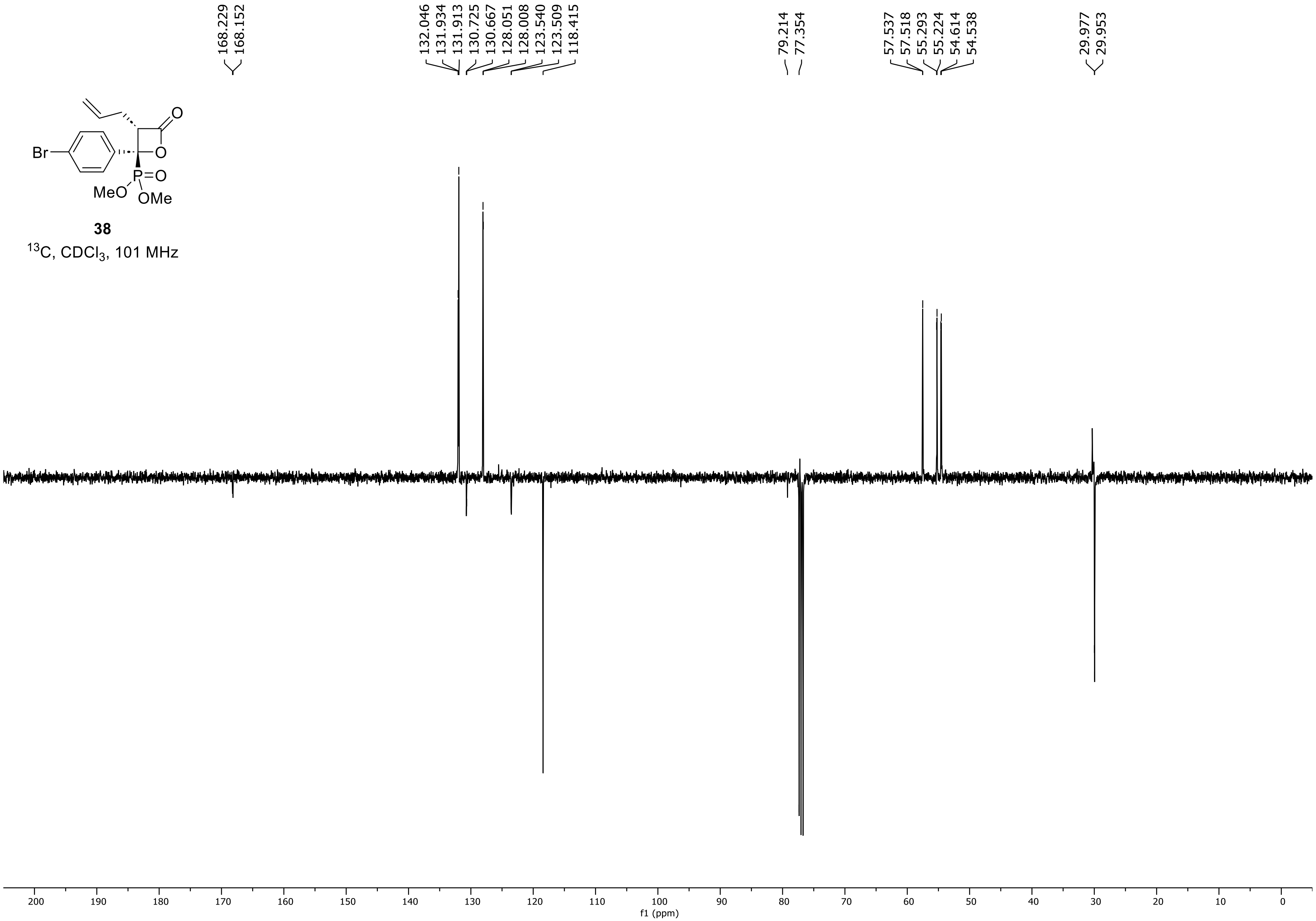

38

^{13}C, $CDCl_3$, 101 MHz

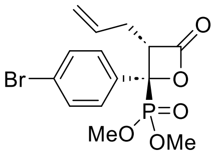

38

^{31}P, $CDCl_3$, 162 MHz

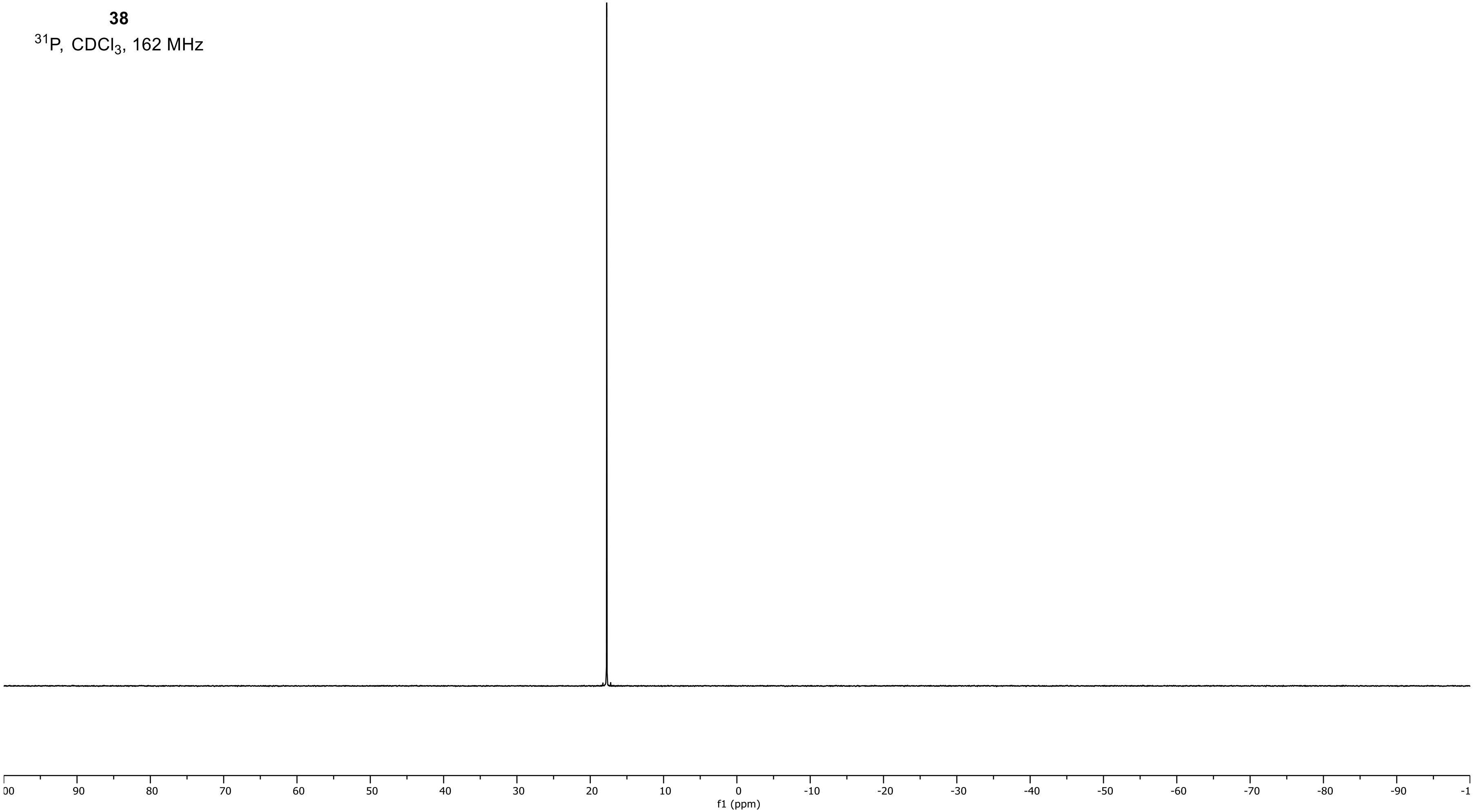

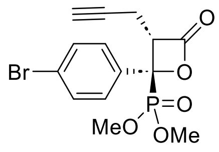

39

^1H, $CDCl_3$, 500 MHz

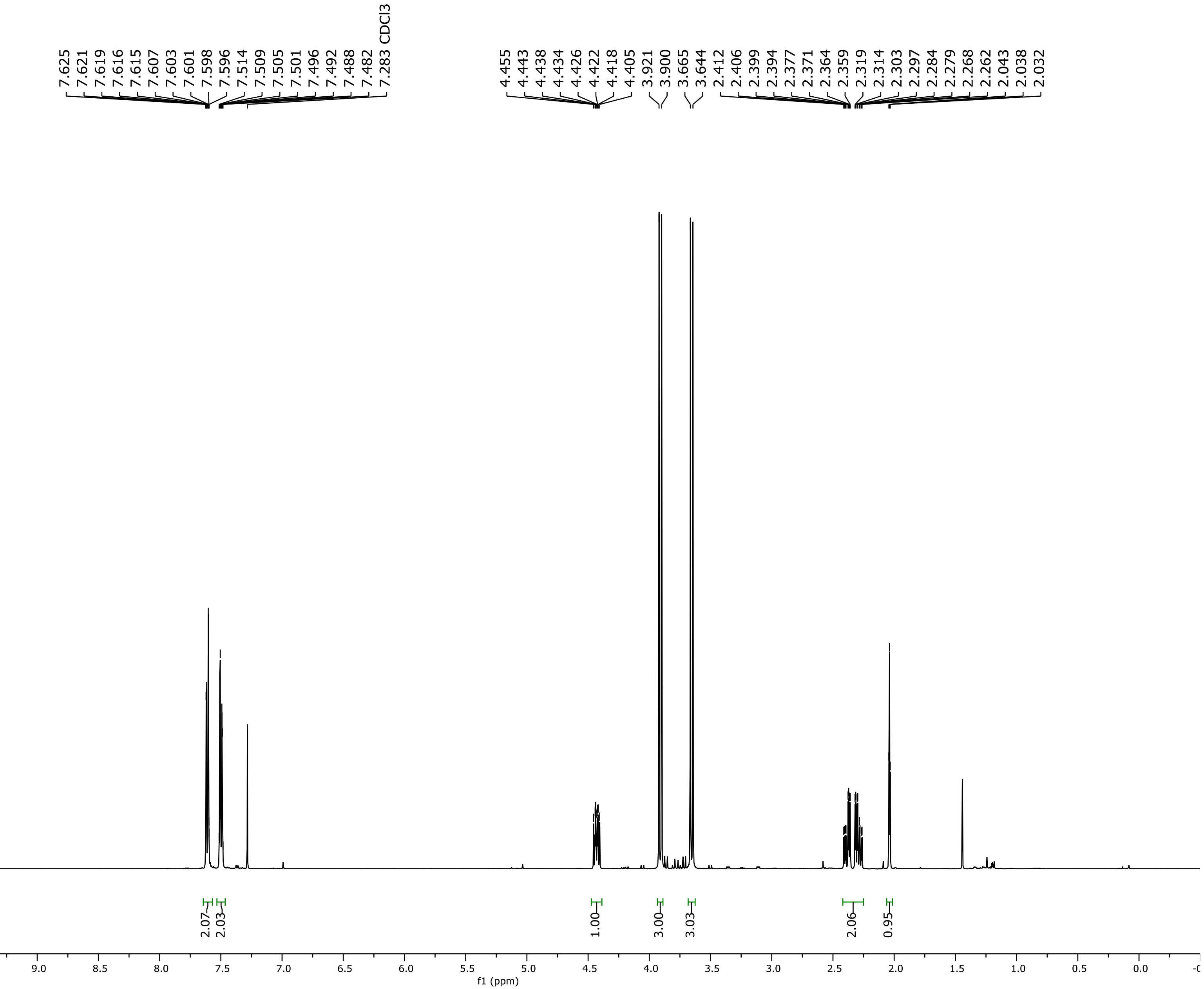

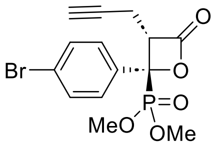

39

^{13}C, $CDCl_3$, 126 MHz

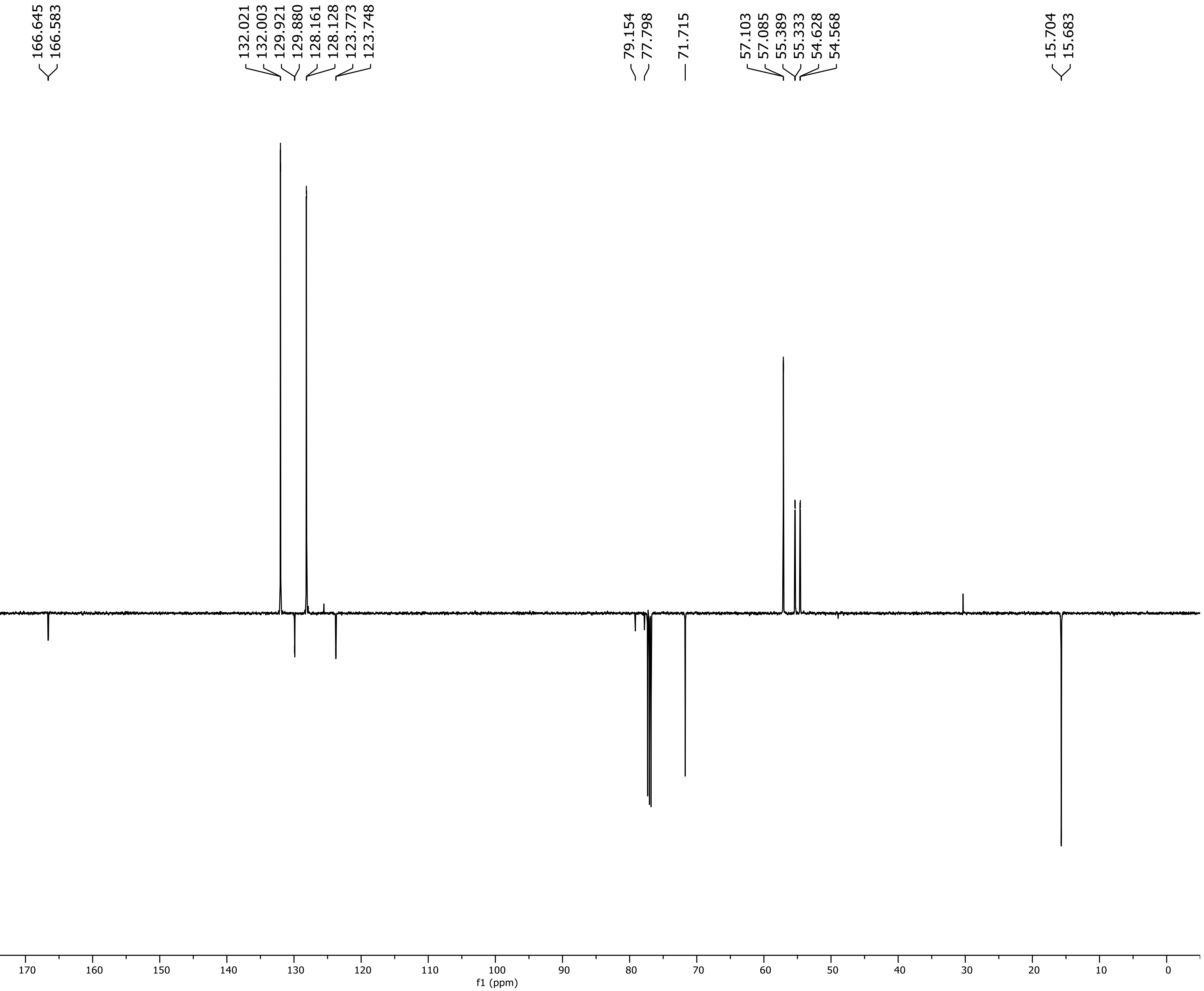

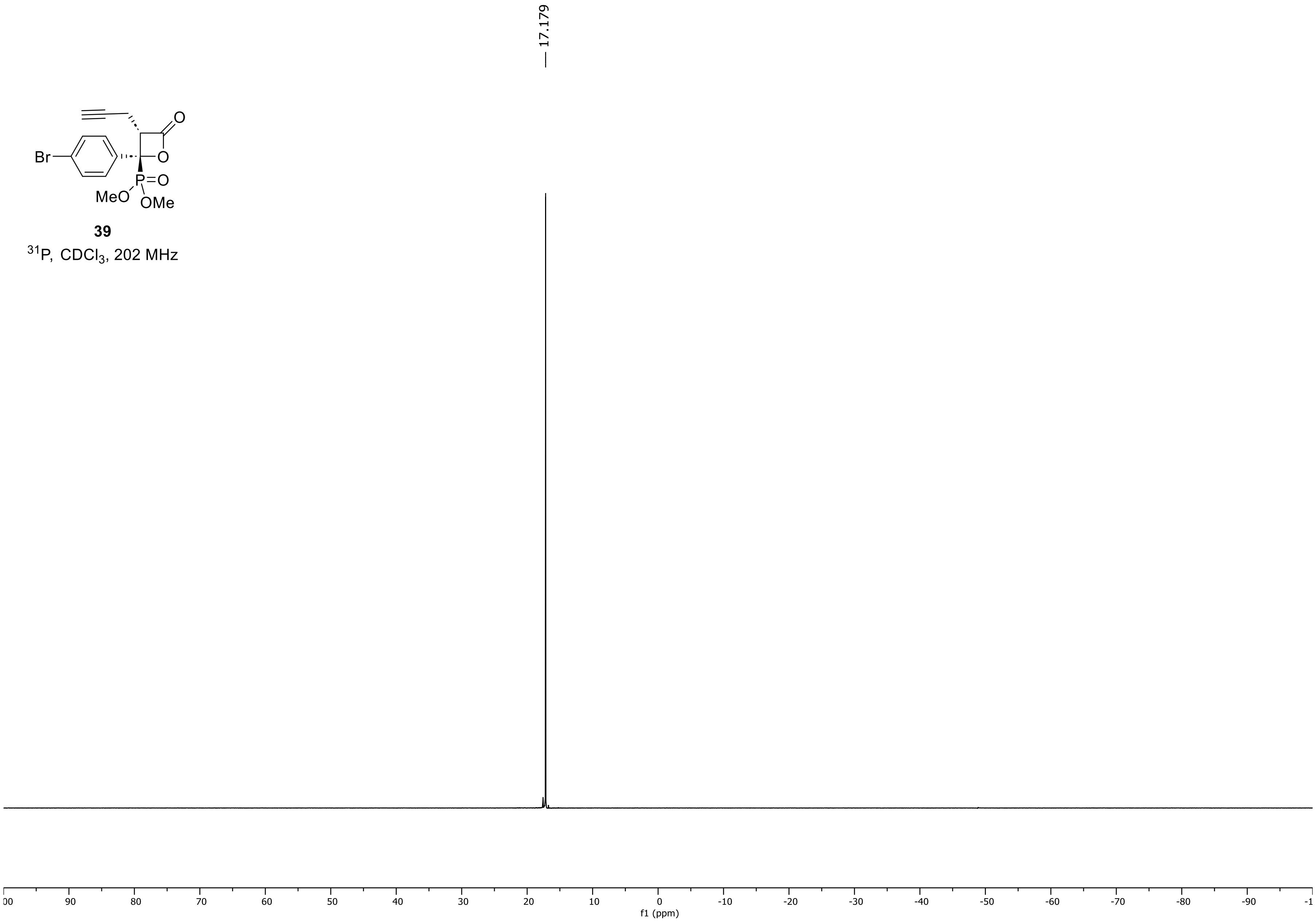

39

^{31}P, $CDCl_3$, 202 MHz

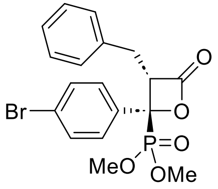

40

^{1}H, $CDCl_3$, 500 MHz

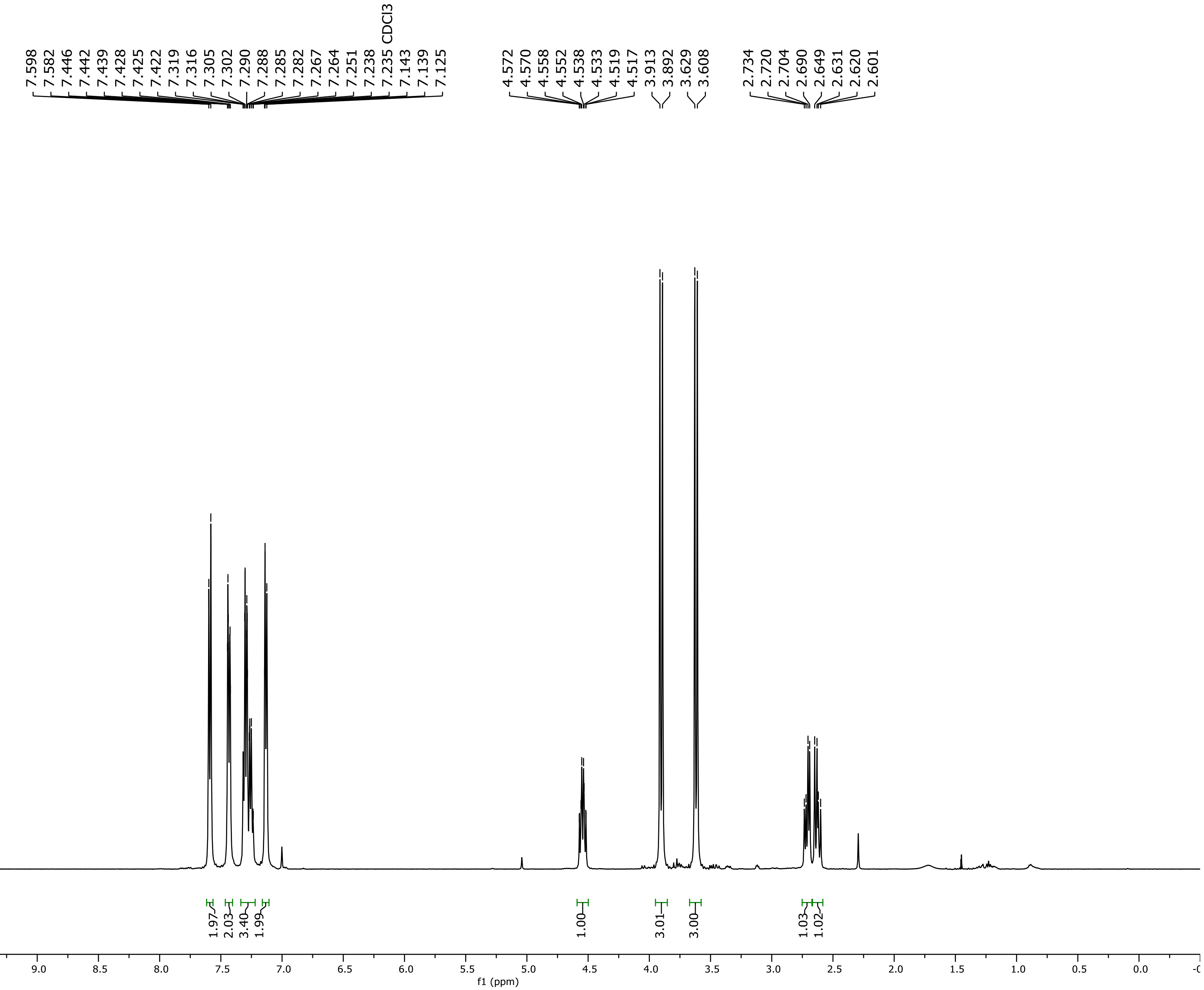

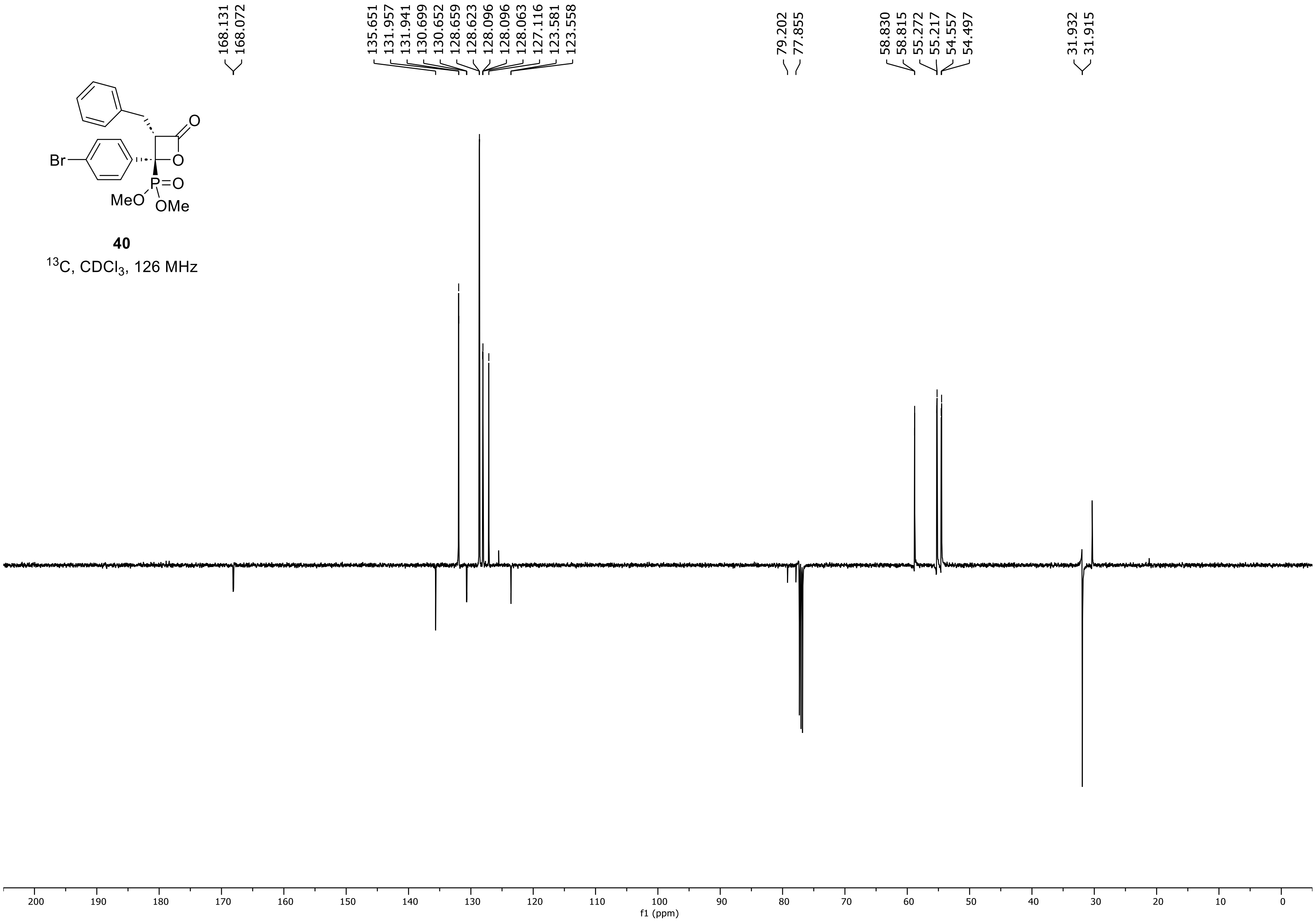

40

^{13}C, $CDCl_3$, 126 MHz

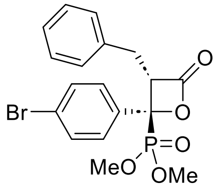

40

^{31}P, $CDCl_3$, 202 MHz

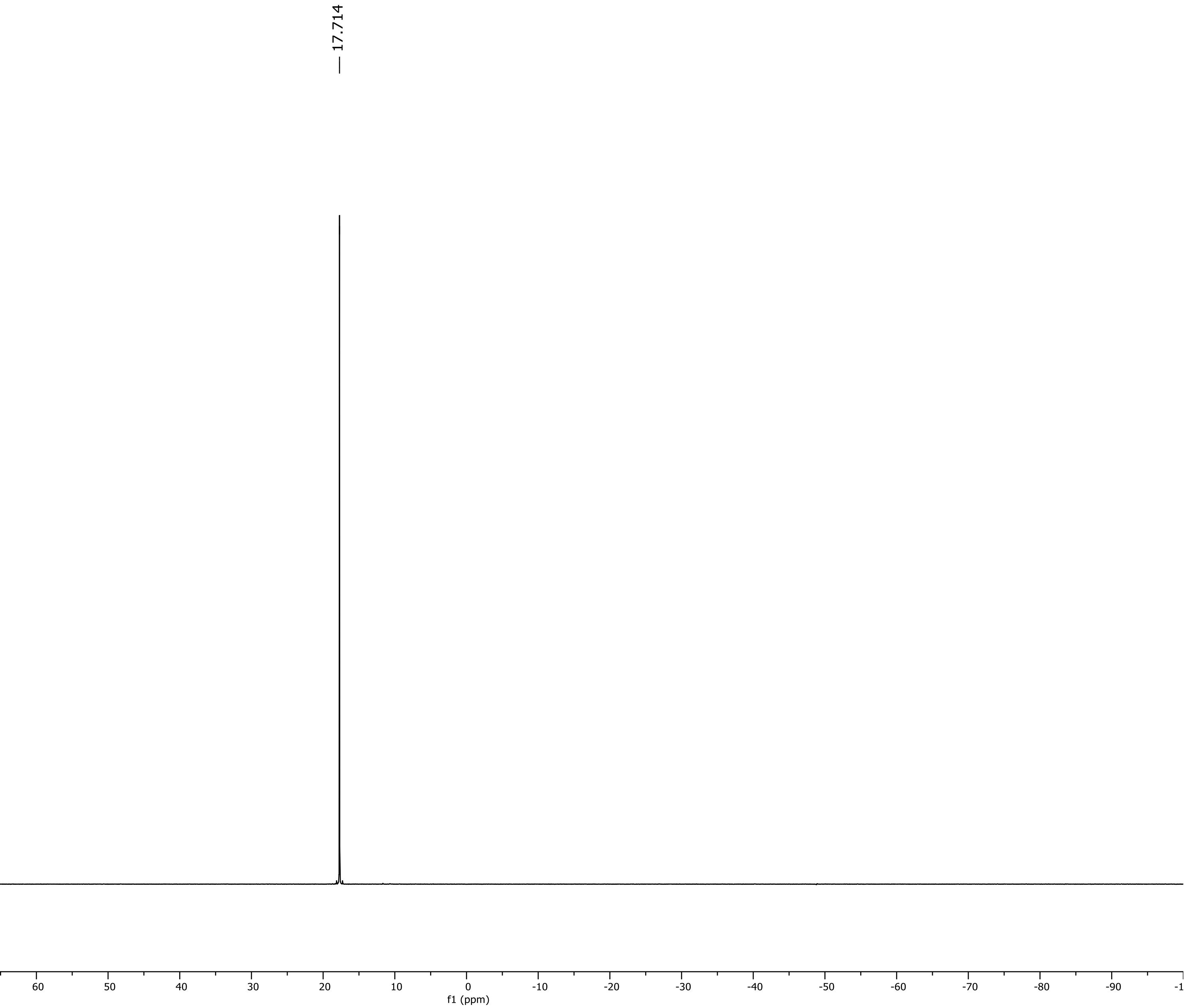

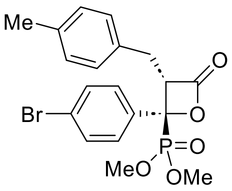

41

^{1}H, $CDCl_3$, 400 MHz

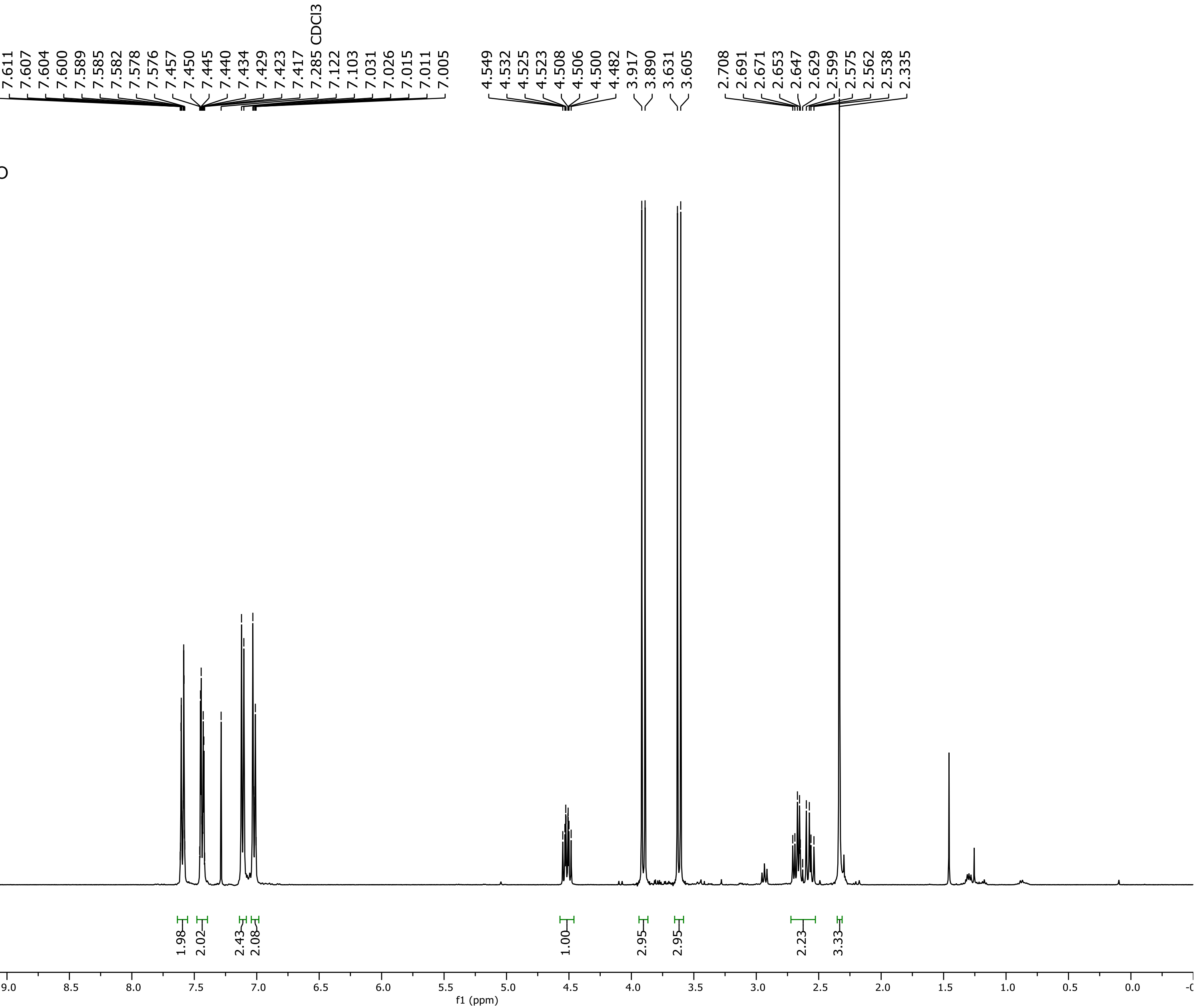

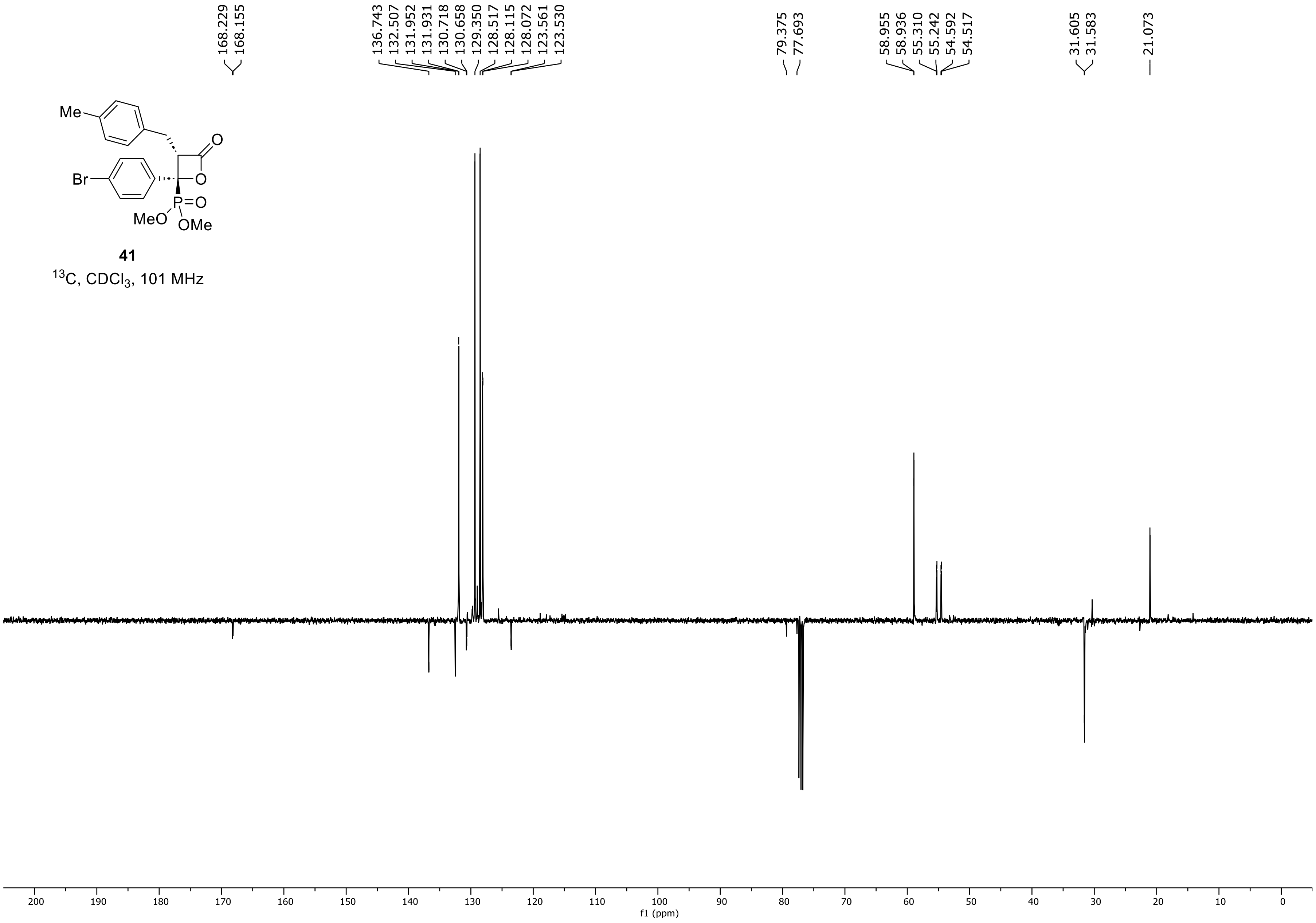

41

^{13}C, $CDCl_3$, 101 MHz

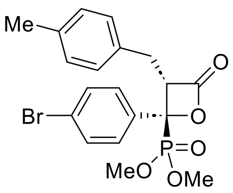

41

^{31}P, $CDCl_3$, 202 MHz

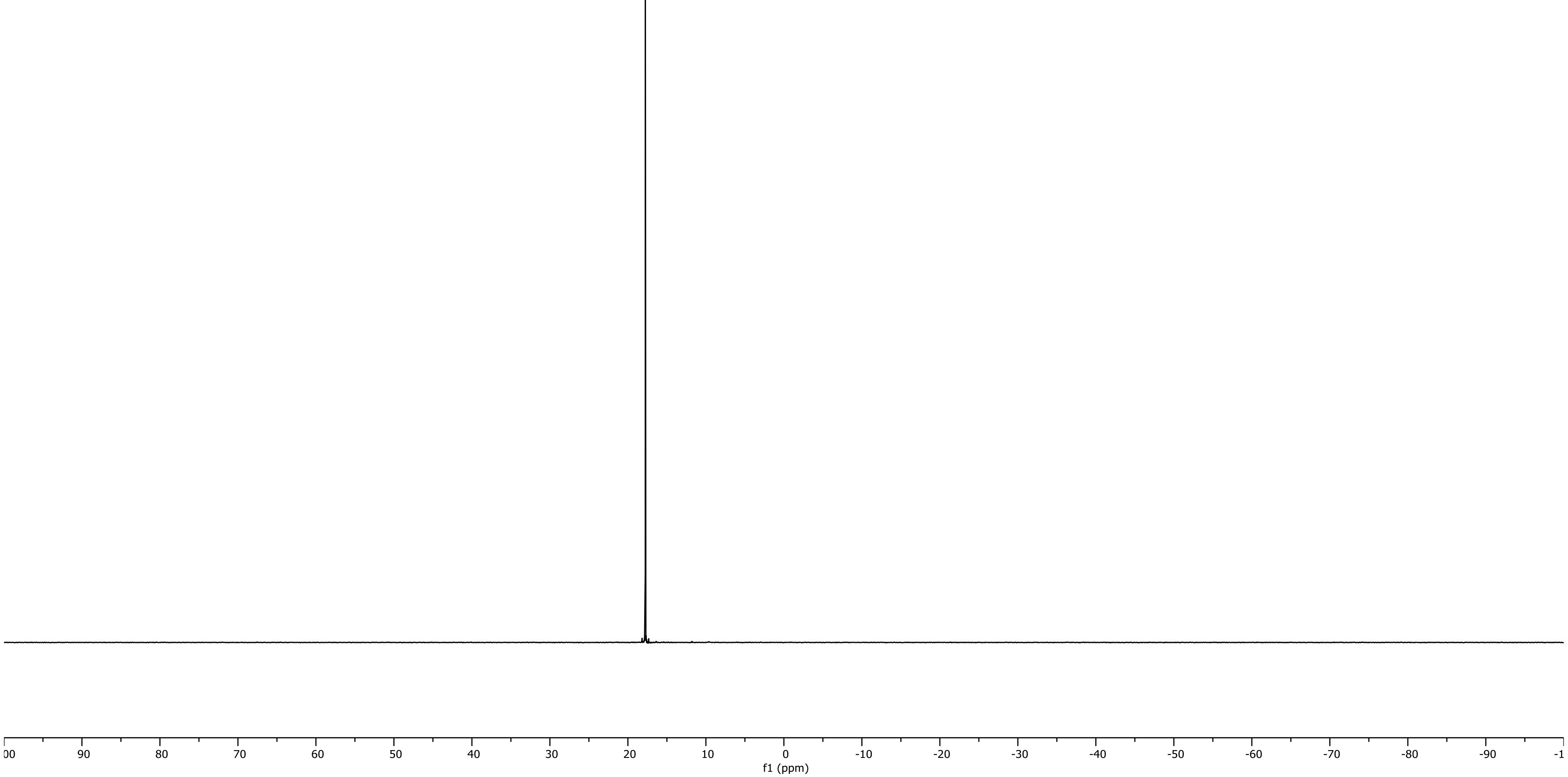

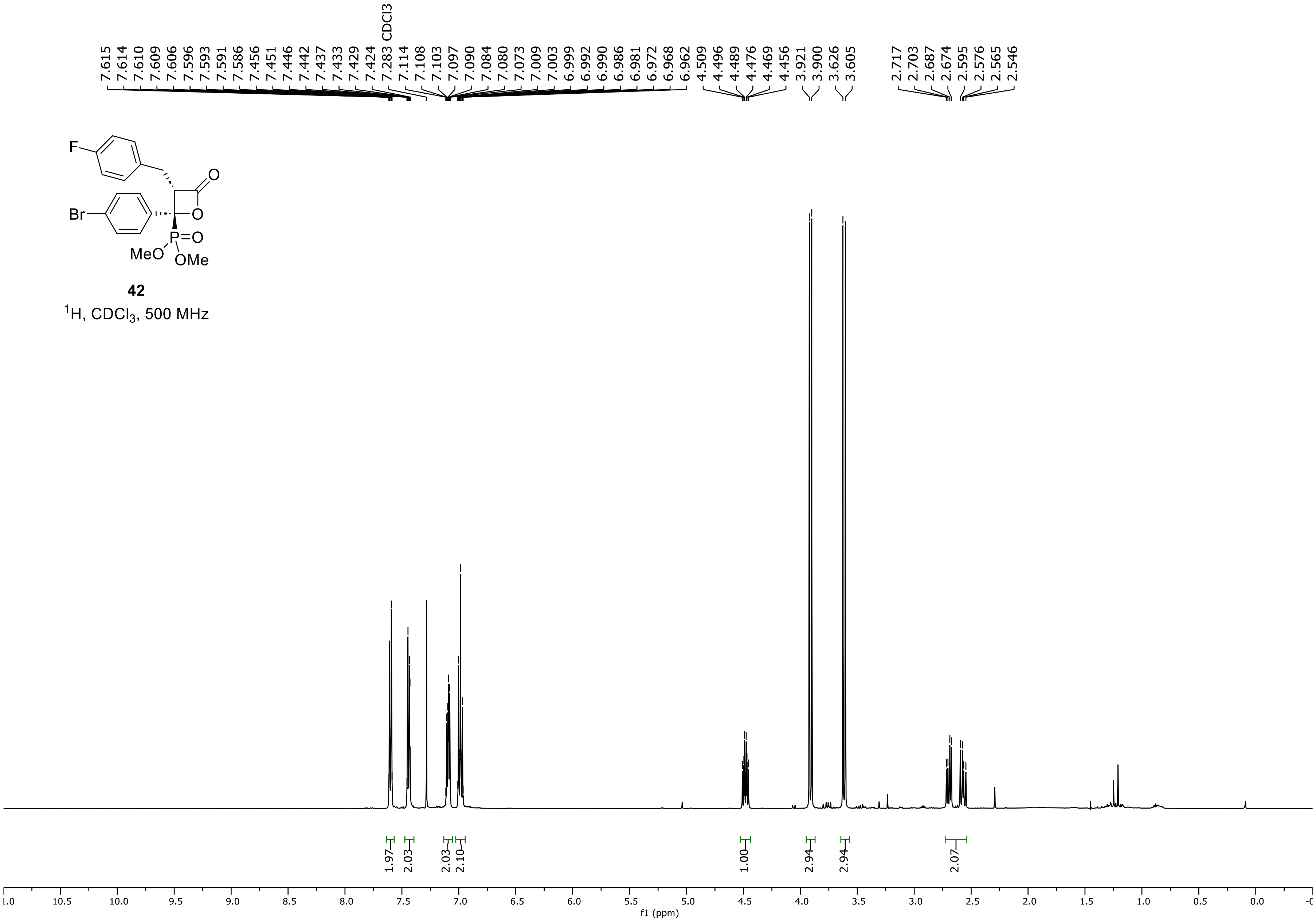
7.615
7.614
7.610
7.609
7.606
7.596
7.593
7.591
7.586
7.456
7.451
7.446
7.442
7.437
7.433
7.429
7.424
7.283 CDCl3
7.114
7.108
7.103
7.097
7.090
7.084
7.080
7.073
7.009
7.003
6.999
6.992
6.990
6.986
6.981
6.972
6.968
6.962
4.509
4.496
4.489
4.476
4.469
4.456
3.921
3.900
3.626
3.605
2.717
2.703
2.687
2.674
2.595
2.576
2.565
2.546
F
O
Br
O
P=O
MeO
OMe
42
1H, CDCl3, 500 MHz
1.97
2.03
2.03
2.10
1.00
2.94
2.94
2.07
f1 (ppm)

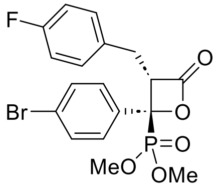

42

^{13}C, $CDCl_3$, 126 MHz

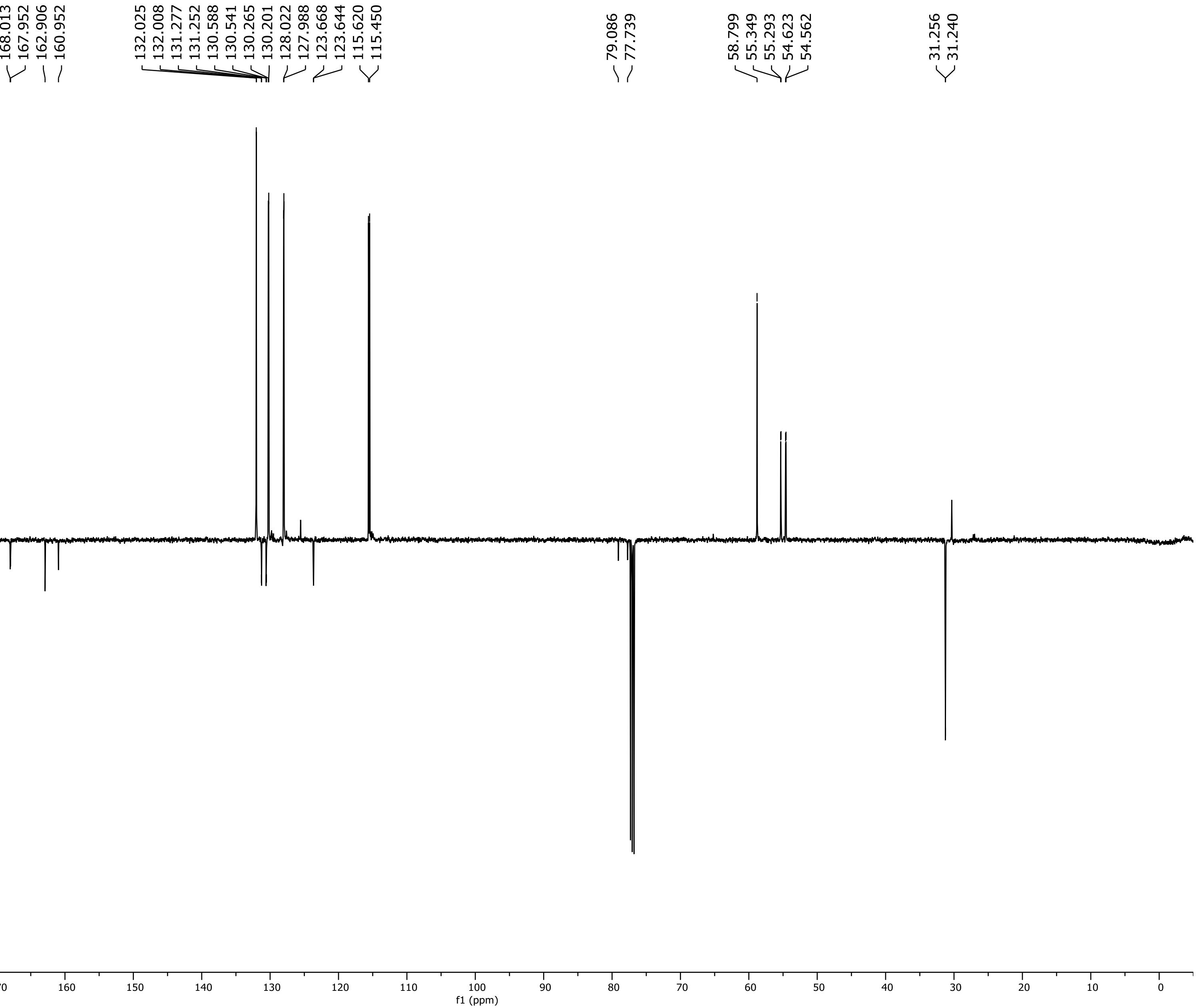

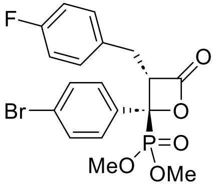

42

^{19}F, $CDCl_3$, 471 MHz

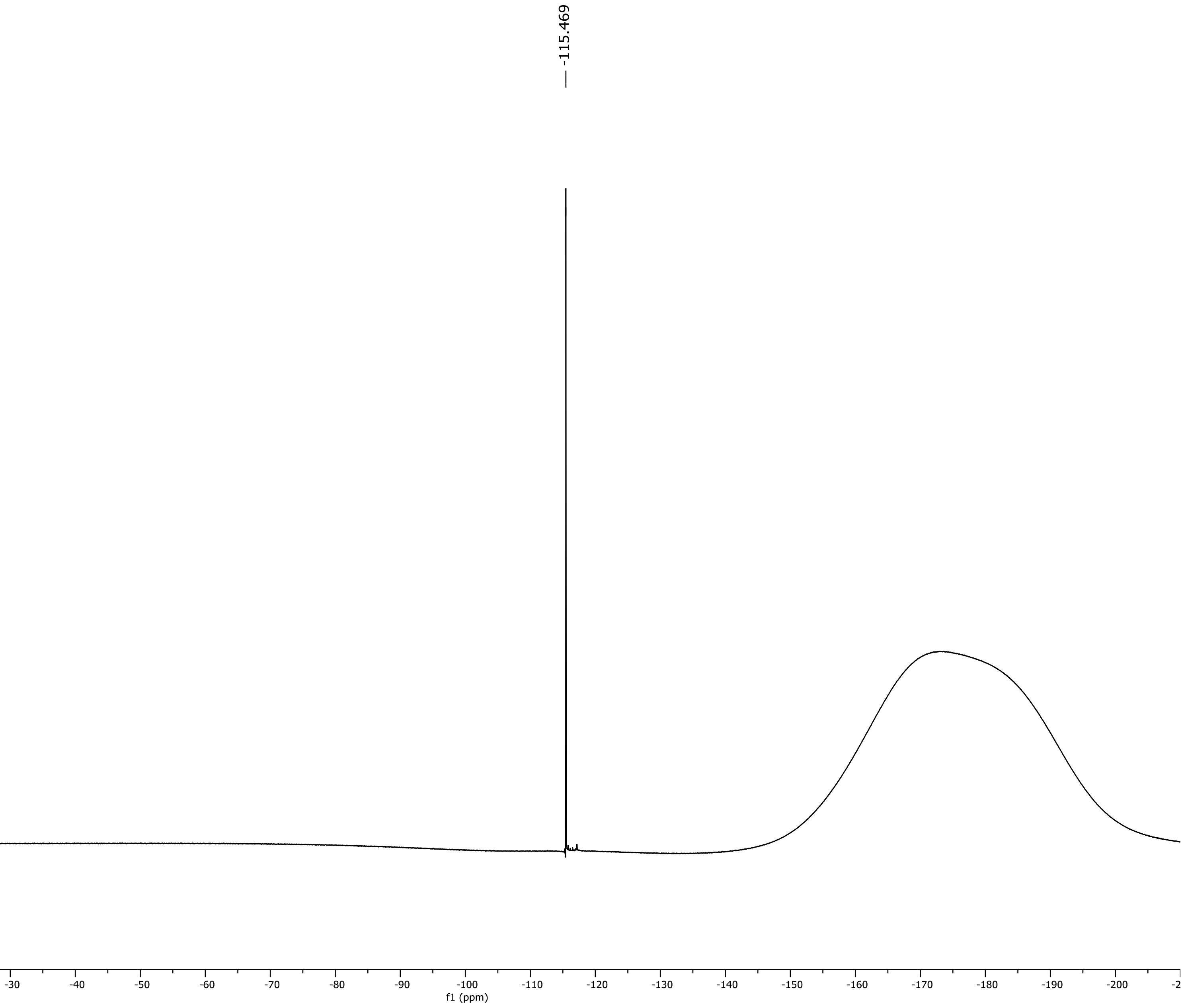

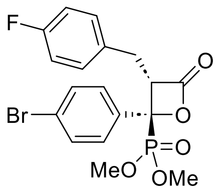

42

^{31}P, $CDCl_3$, 202 MHz

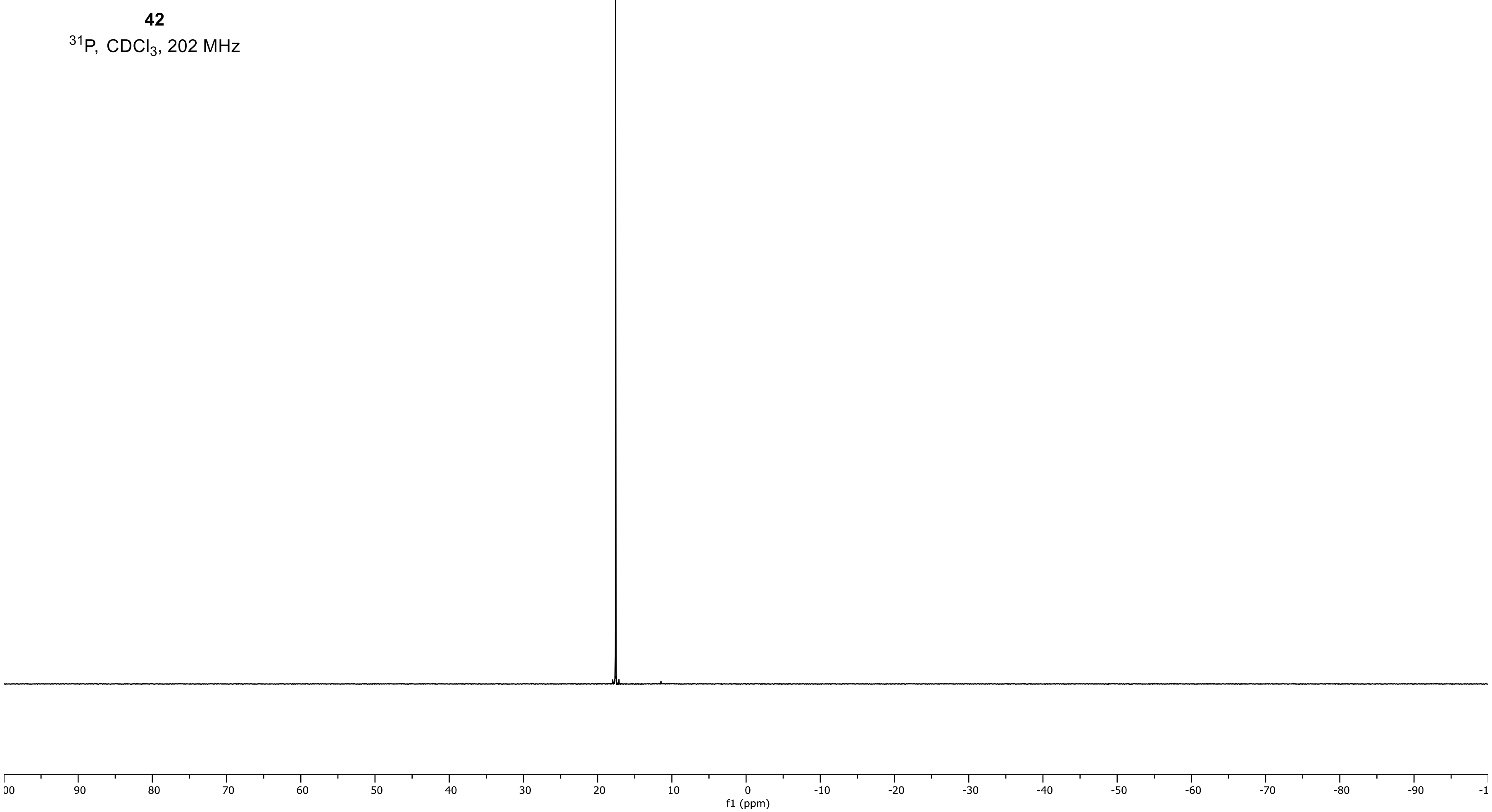

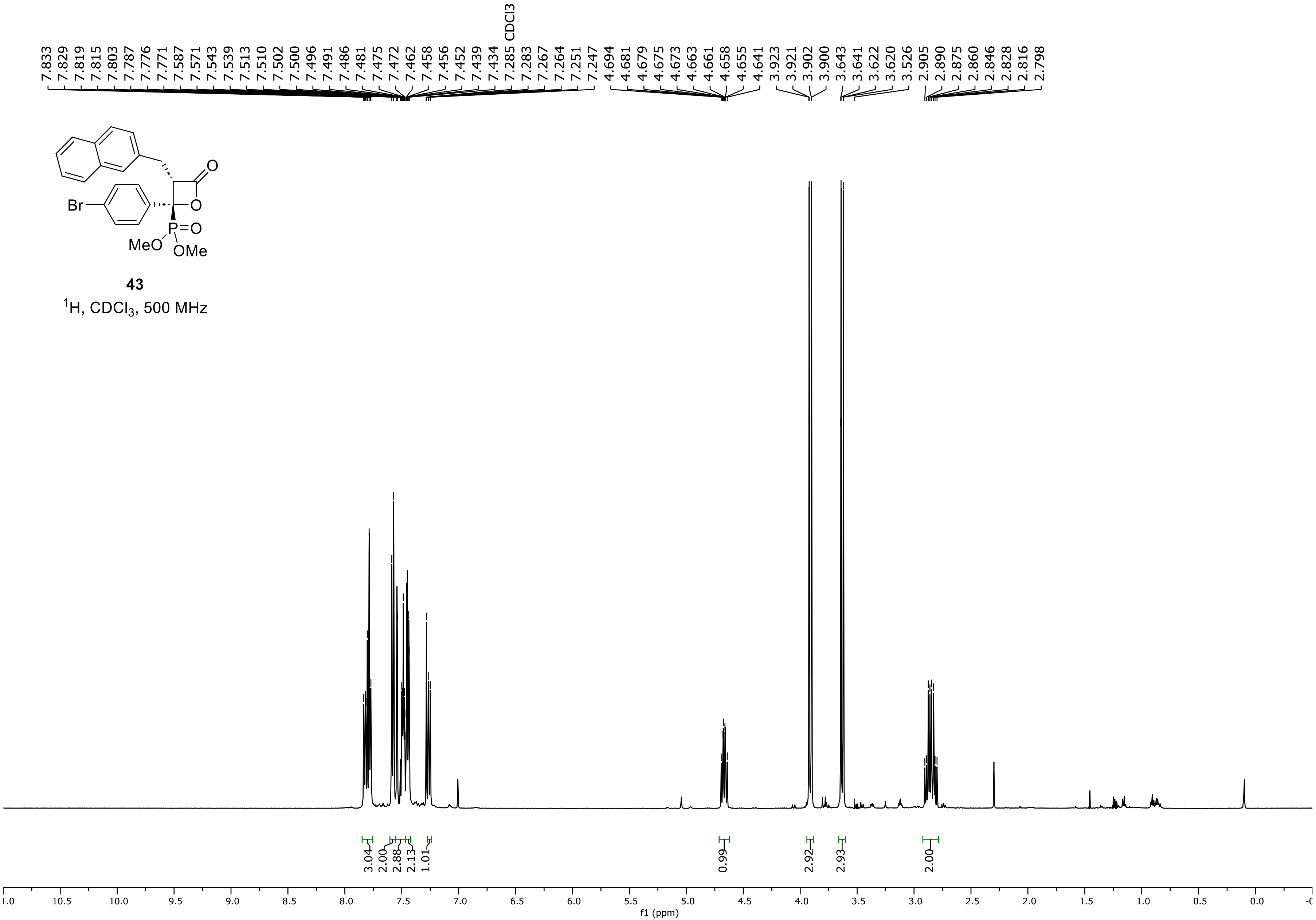
7.833
7.829
7.819
7.815
7.803
7.787
7.776
7.771
7.587
7.571
7.543
7.539
7.513
7.510
7.502
7.500
7.496
7.491
7.486
7.481
7.475
7.472
7.462
7.458
7.456
7.452
7.439
7.434
7.285 CDCl3
7.283
7.267
7.264
7.251
7.247
4.694
4.681
4.679
4.675
4.673
4.663
4.661
4.658
4.655
4.641
3.923
3.921
3.902
3.900
3.643
3.641
3.622
3.620
3.526
2.905
2.890
2.875
2.860
2.846
2.828
2.816
2.798
Br
O
O
P=O
MeO
OMe
43
1H, CDCl3, 500 MHz
3.04
2.00
2.88
2.13
1.01
0.99
2.92
2.93
2.00
10.5
10.0
9.5
9.0
8.5
8.0
7.5
7.0
6.5
6.0
5.5
5.0
4.5
4.0
3.5
3.0
2.5
2.0
1.5
1.0
0.5
0.0
f1 (ppm)

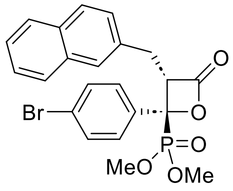

43

^{13}C, $CDCl_3$, 126 MHz

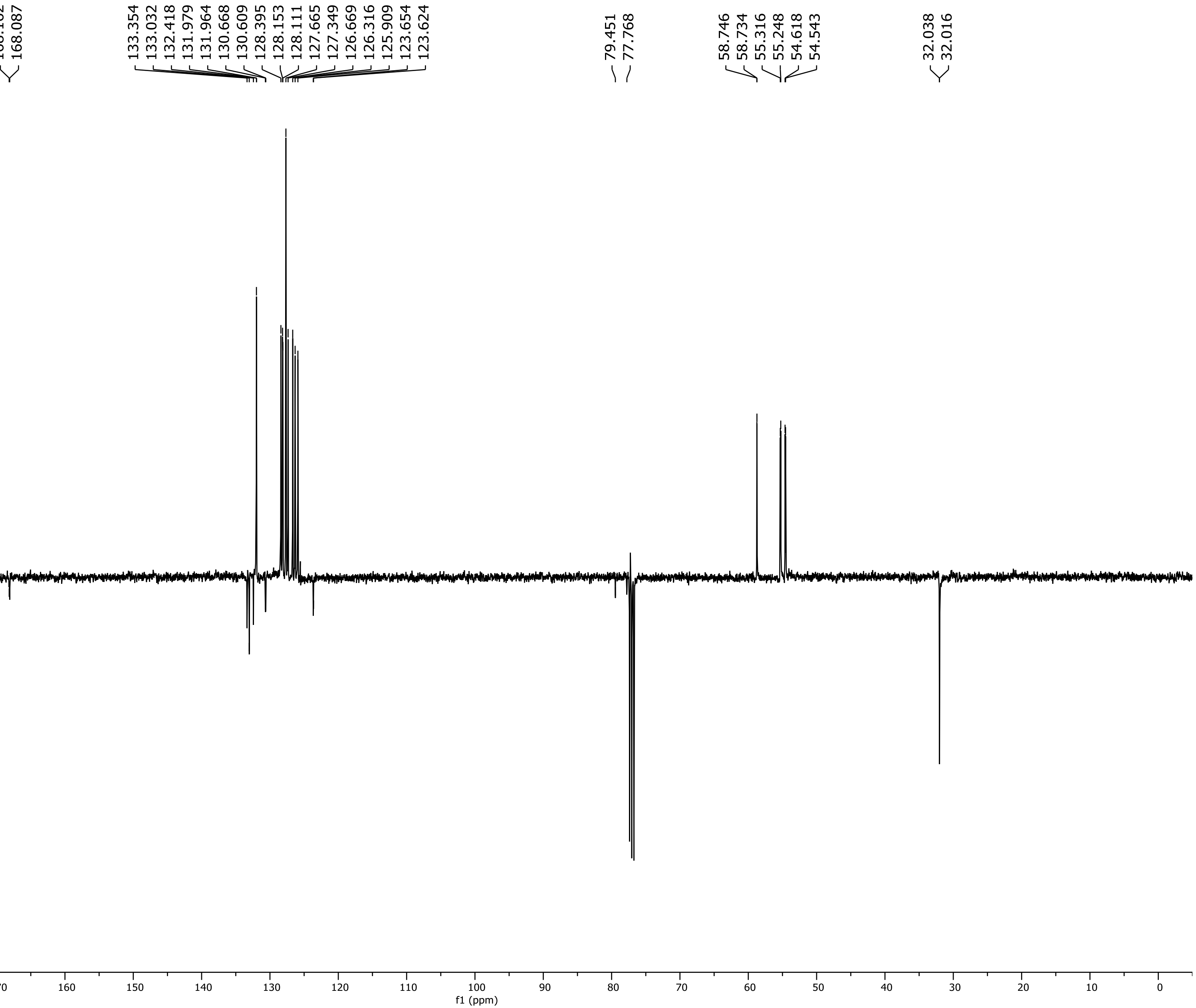

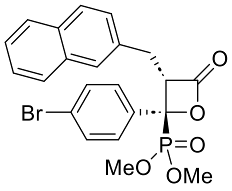

43

^{31}P, $CDCl_3$, 202 MHz

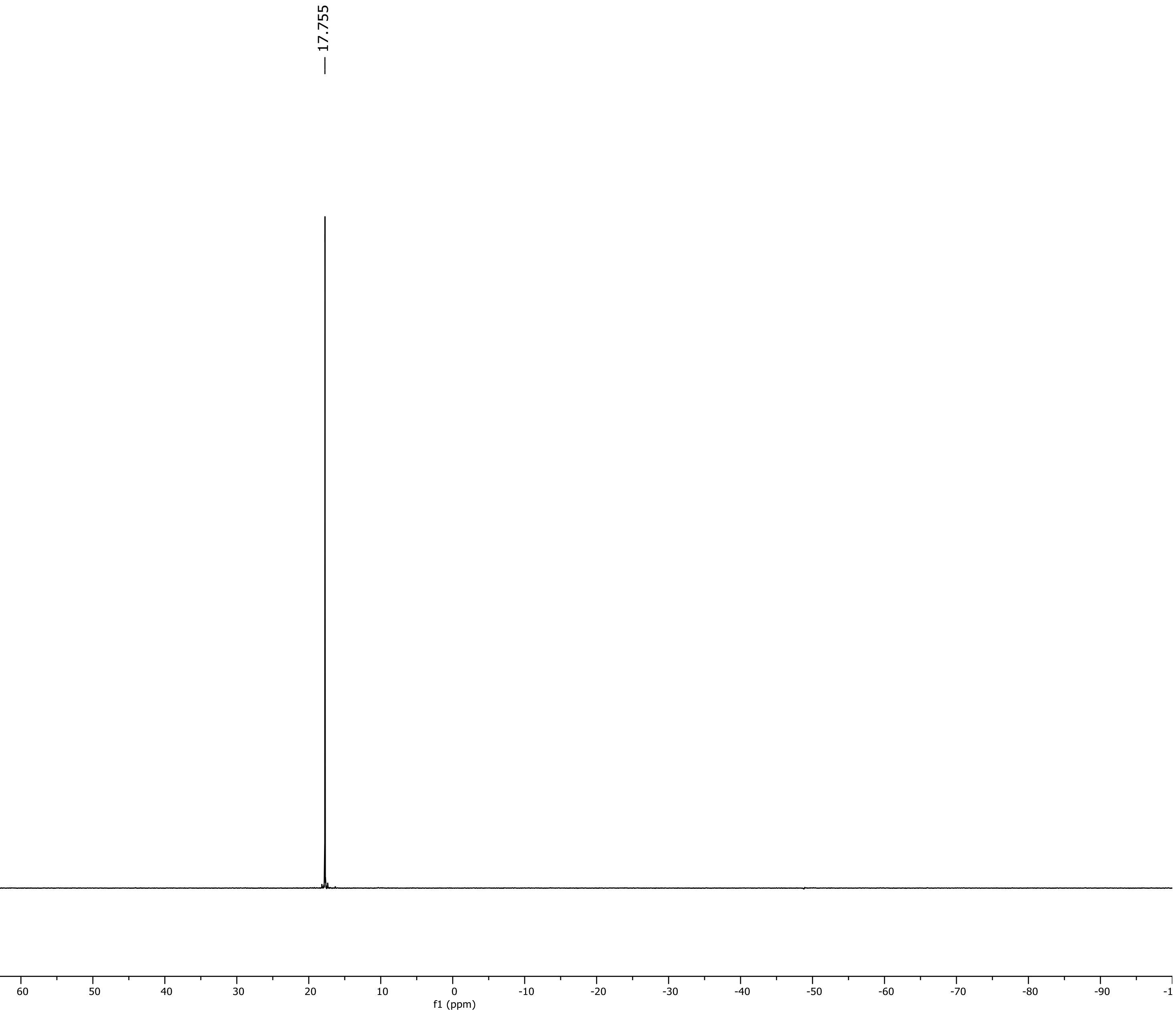

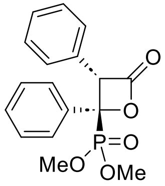

44

^{1}H, $CDCl_3$, 400 MHz

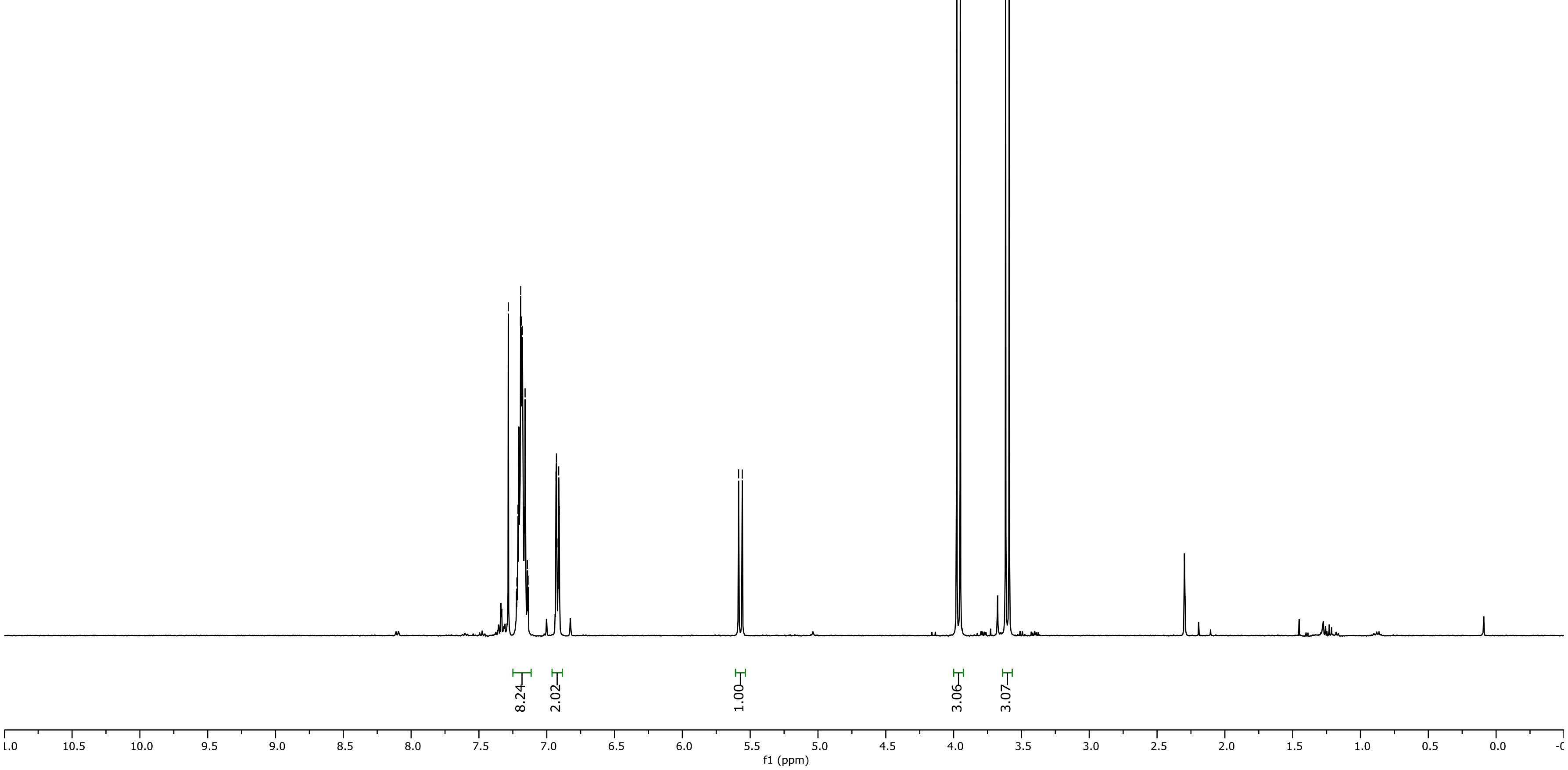

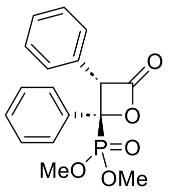

44

^{13}C, $CDCl_3$, 101 MHz

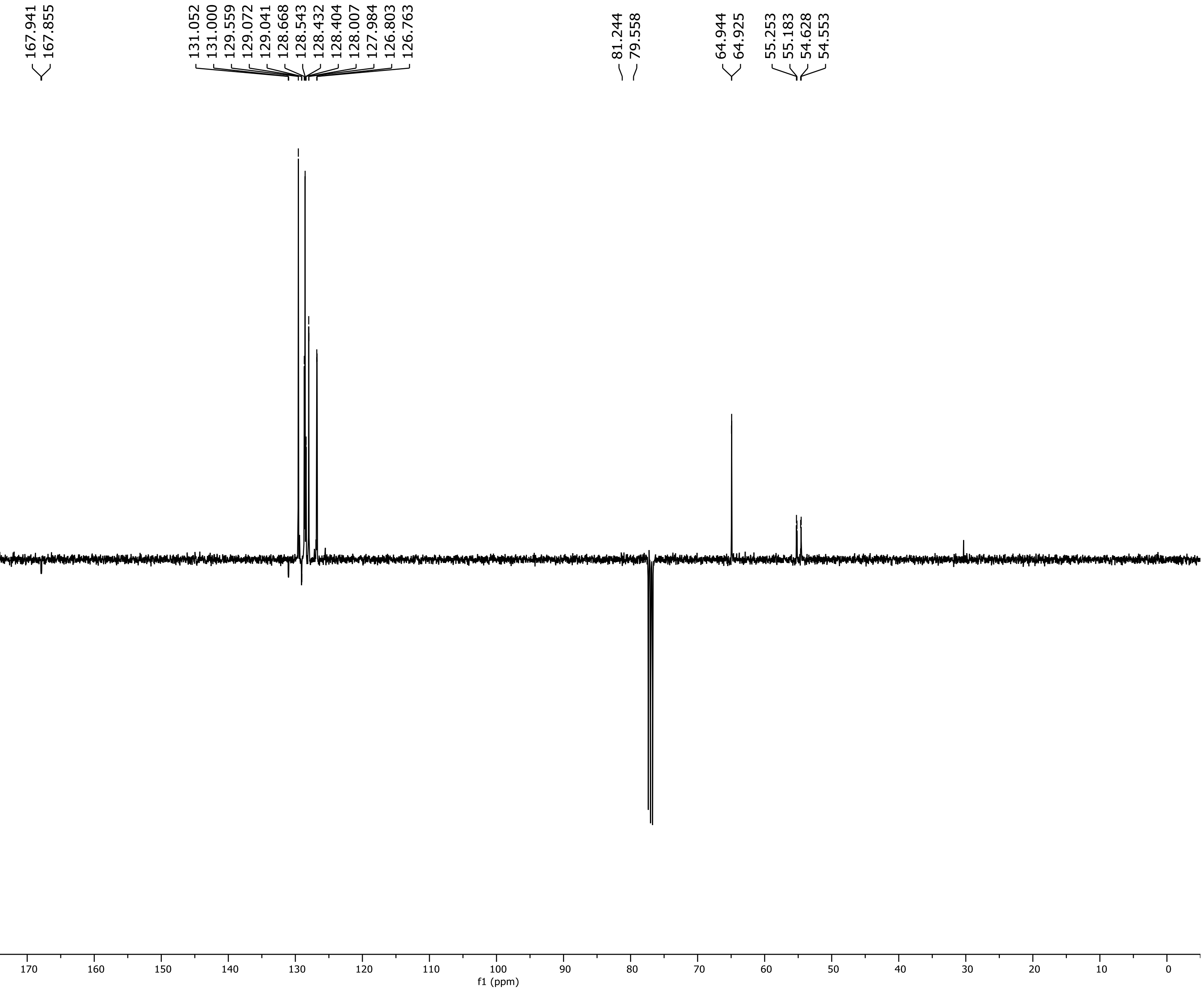

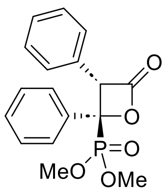

44

^{31}P, $CDCl_3$, 162 MHz

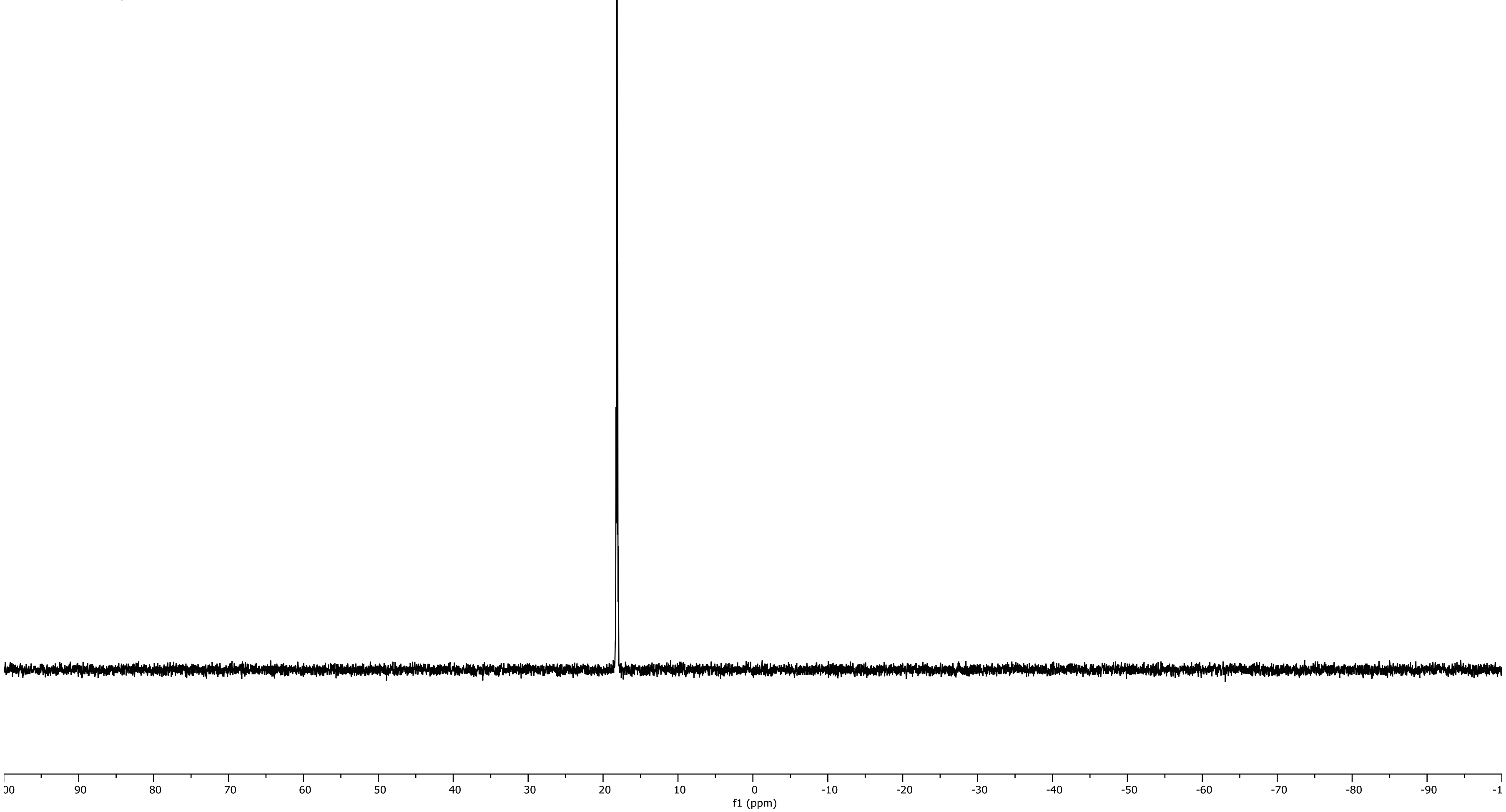

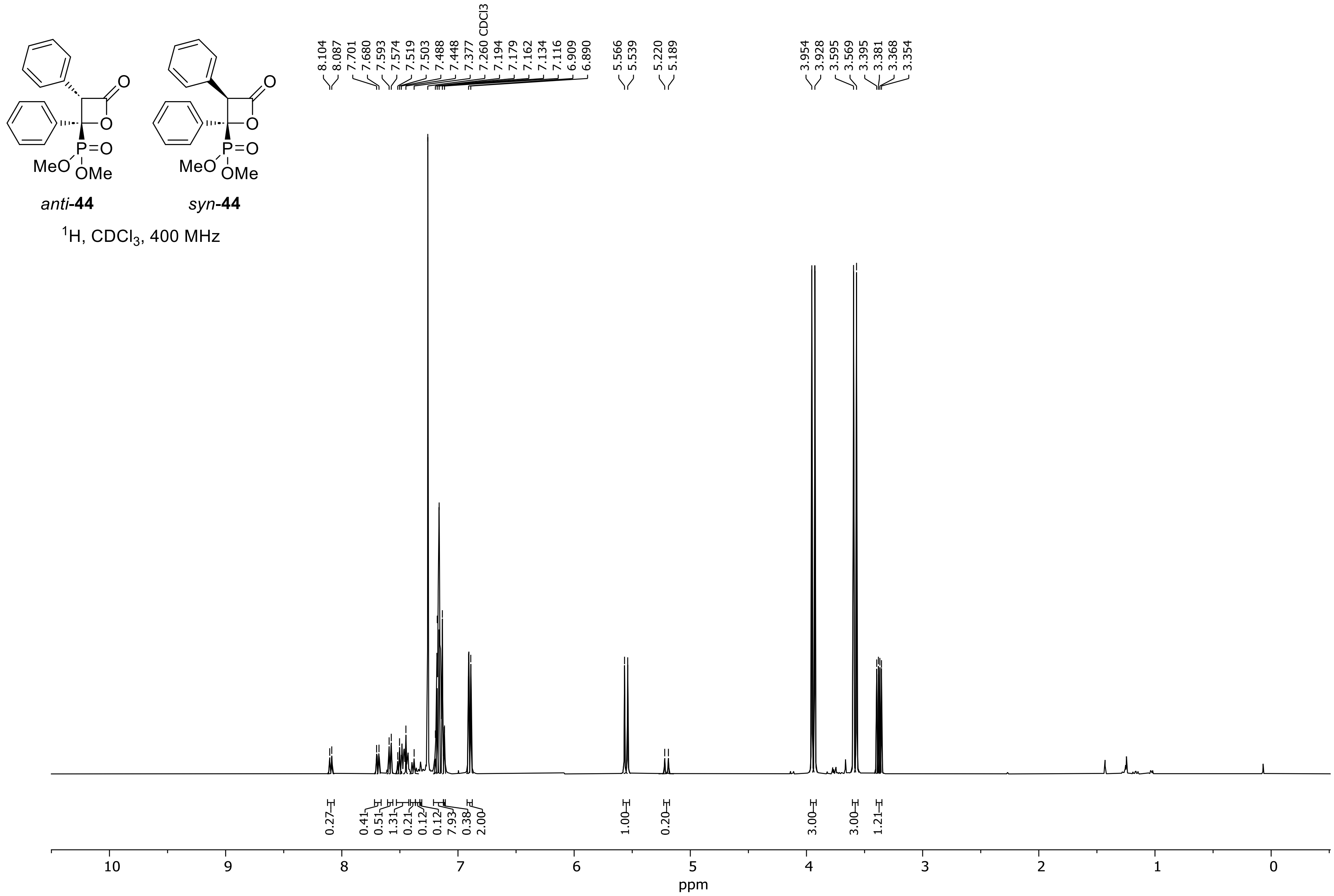
anti-44
syn-44
1H, CDCl3, 400 MHz
ppm

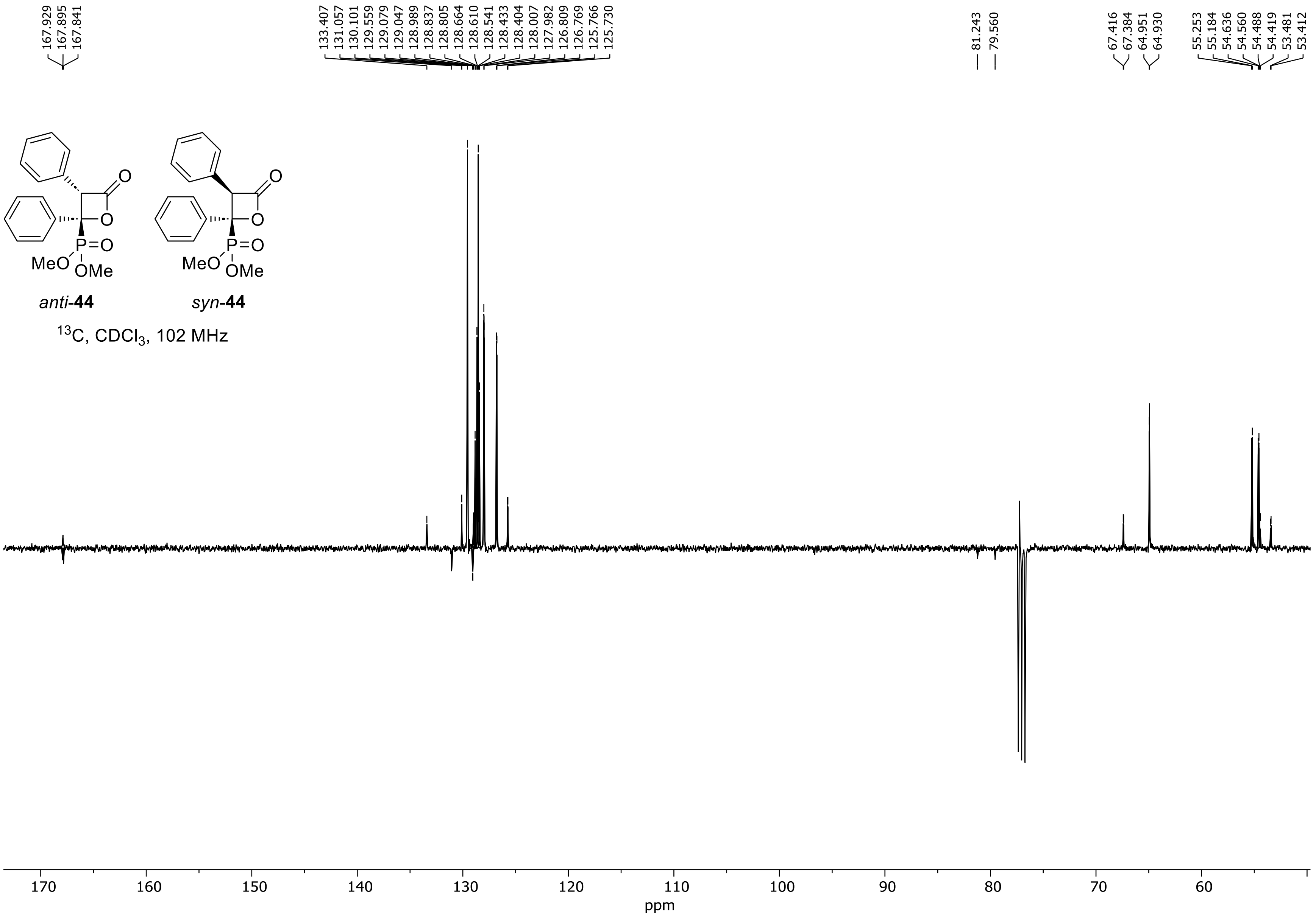
167.929
167.895
167.841
133.407
131.057
130.101
129.559
129.079
129.047
128.989
128.837
128.805
128.664
128.610
128.541
128.433
128.404
128.007
127.982
126.809
126.769
125.766
125.730
81.243
79.560
67.416
67.384
64.951
64.930
55.253
55.184
54.636
54.560
54.488
54.419
53.481
53.412
MeO
OMe
P=O
anti-44
syn-44
13C, CDCl3, 102 MHz
170
160
150
140
130
120
110
100
90
80
70
60
ppm

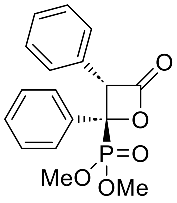

anti-**44**

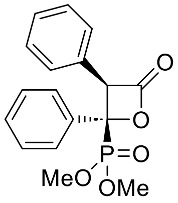

syn-**44**

^{31}P, $CDCl_3$, 162 MHz

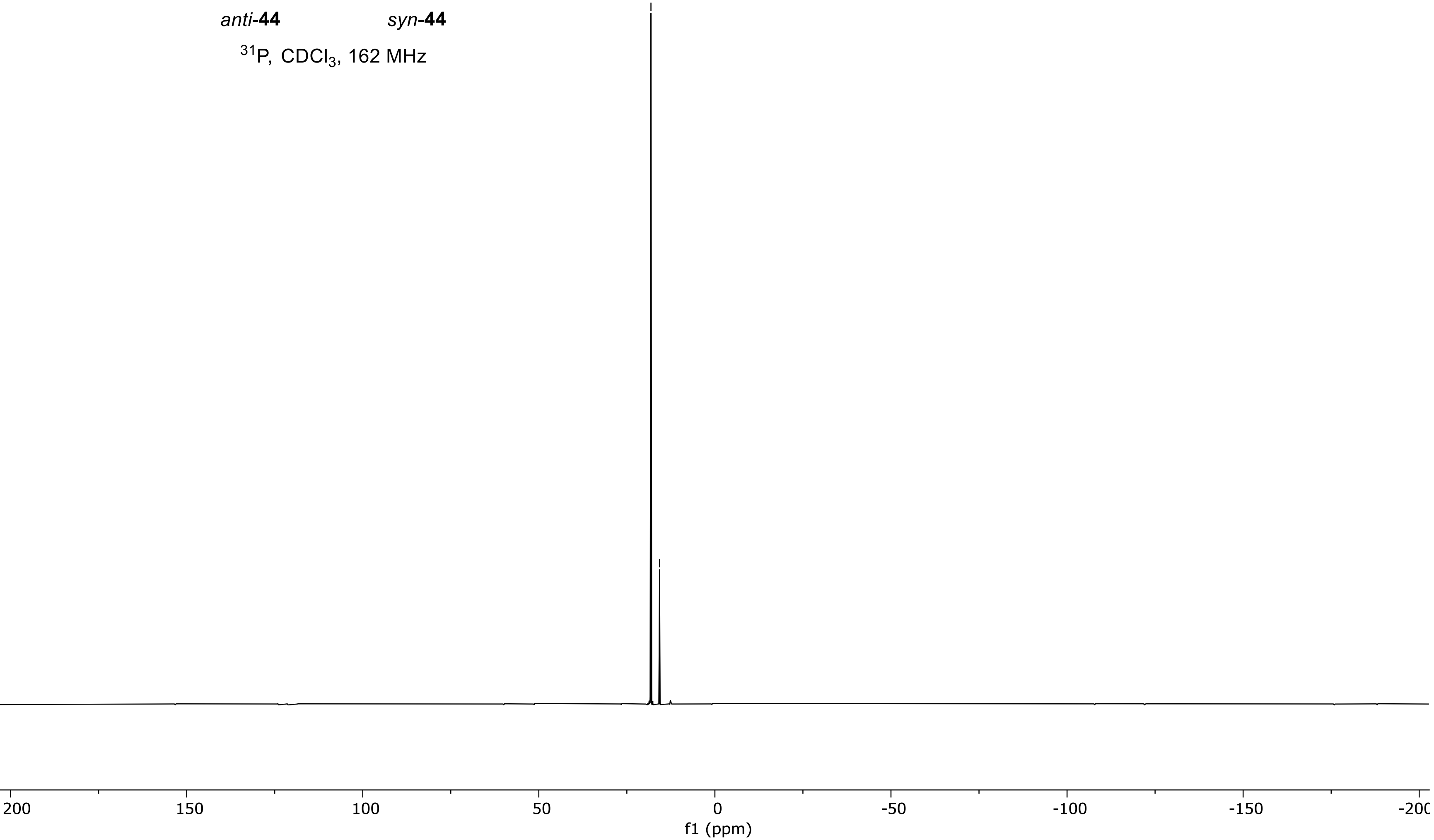

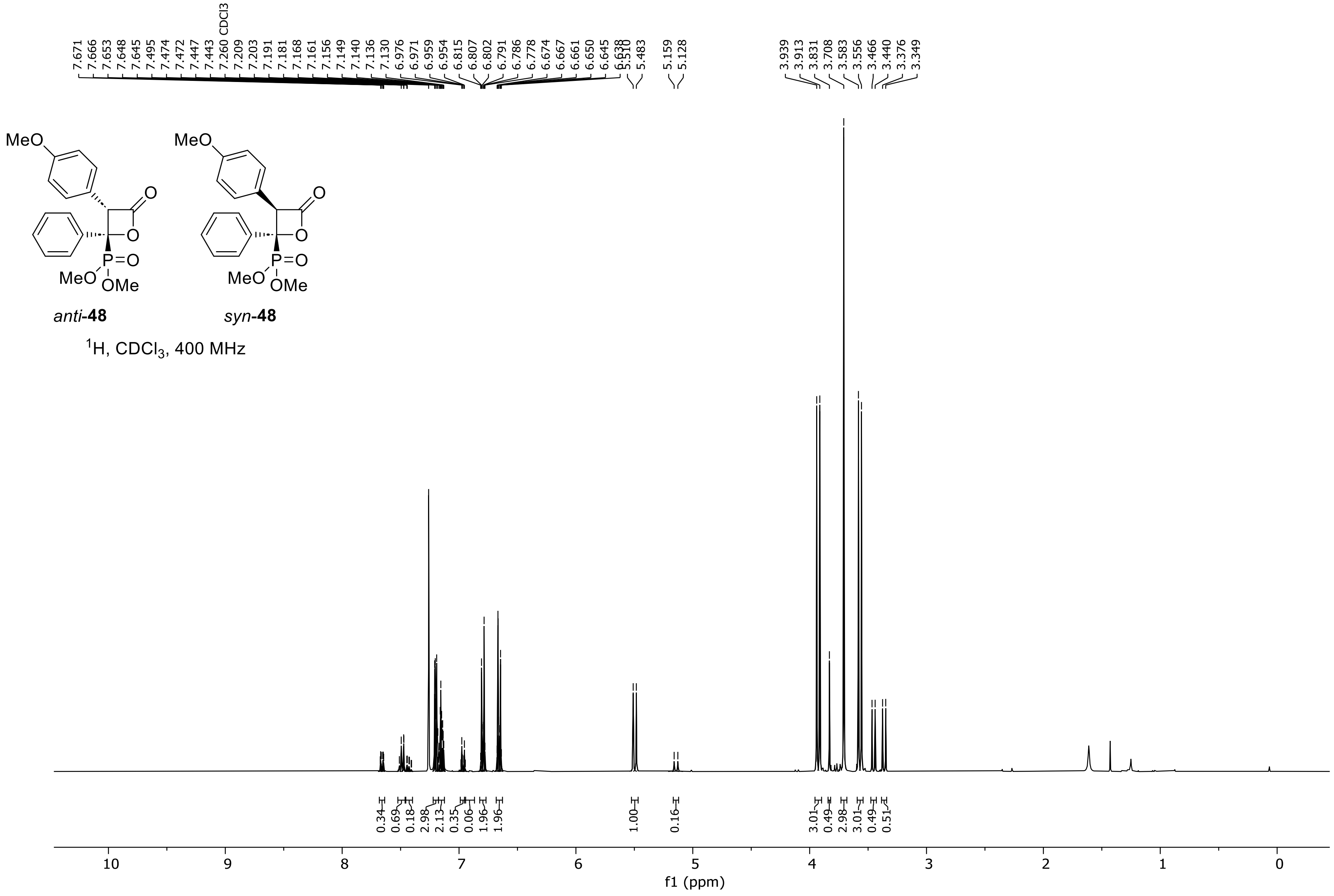
7.671
7.666
7.653
7.648
7.645
7.495
7.474
7.472
7.447
7.443
7.260 CDCl3
7.209
7.203
7.191
7.181
7.168
7.161
7.156
7.149
7.140
7.136
7.130
6.976
6.971
6.959
6.954
6.815
6.807
6.802
6.791
6.786
6.778
6.674
6.667
6.661
6.650
6.645
6.638
5.510
5.483
5.159
5.128
3.939
3.913
3.831
3.708
3.583
3.556
3.466
3.440
3.376
3.349
MeO
O
O
P=O
MeO
OMe
anti-48
syn-48
1H, CDCl3, 400 MHz
0.34
0.69
0.18
2.98
2.13
0.35
0.06
1.96
1.96
1.00
0.16
3.01
0.49
2.98
3.01
0.49
0.51
10
9
8
7
6
5
4
3
2
1
0
f1 (ppm)

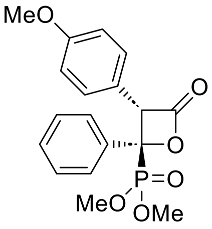

anti-**48**

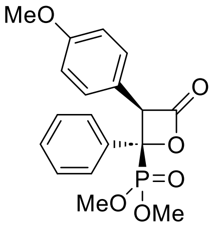

syn-**48**

^{13}C, $CDCl_3$, 101 MHz

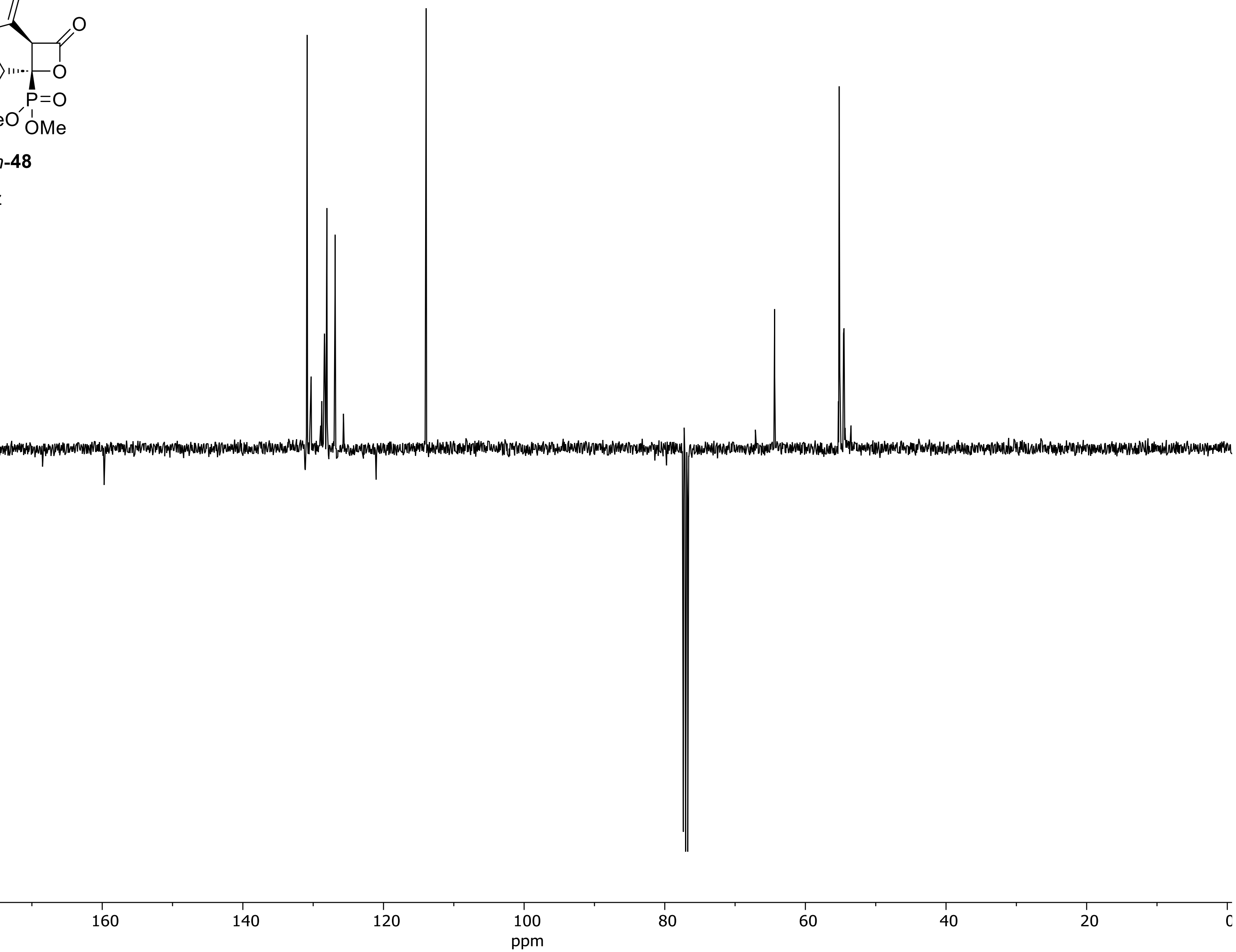

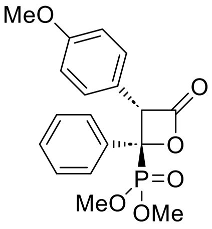

anti-**48**

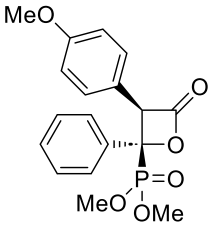

syn-**48**

^{31}P, $CDCl_3$, 162 MHz

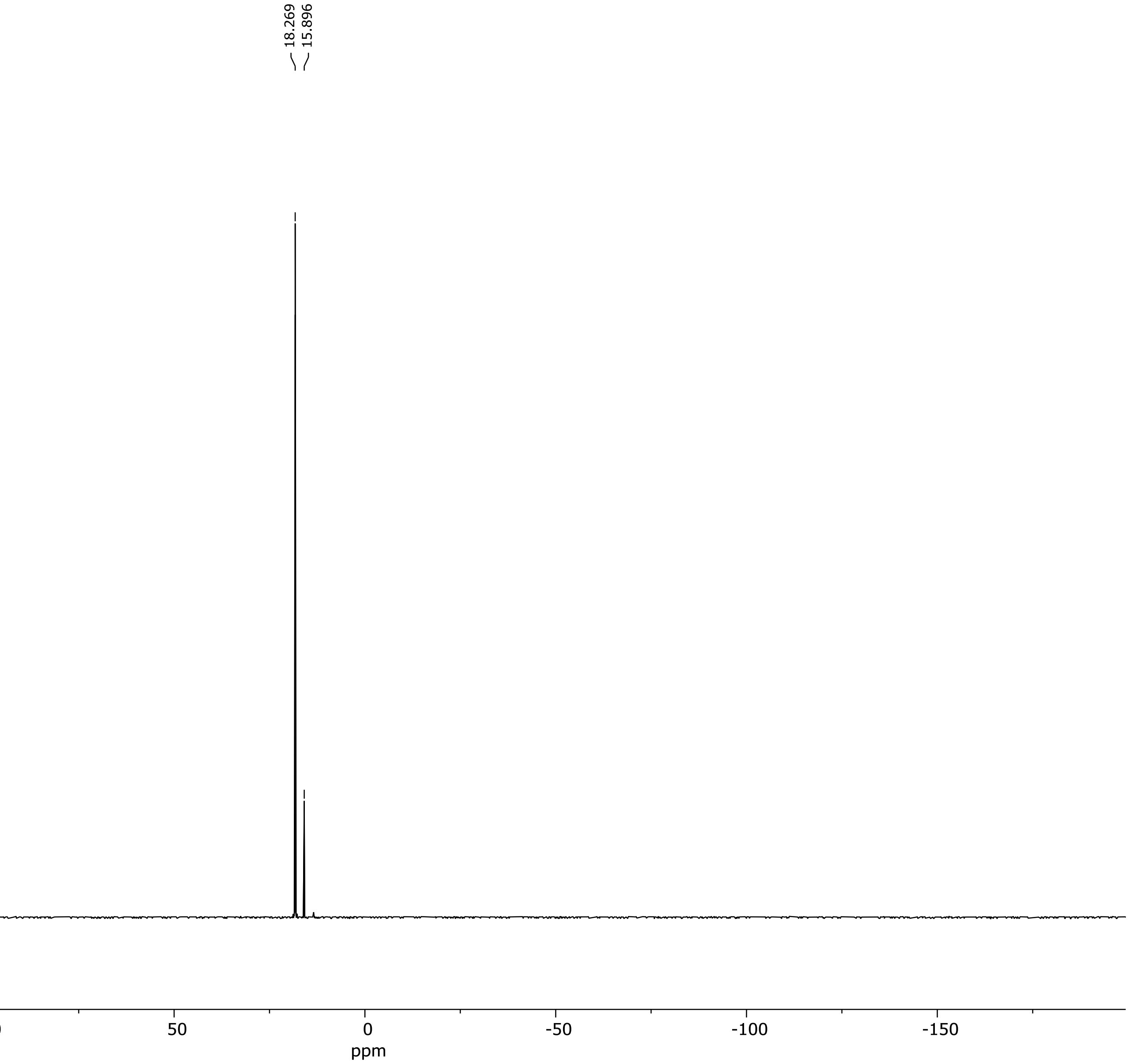

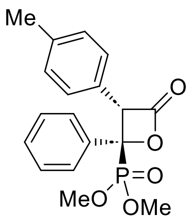

anti-**49**

^{1}H, $CDCl_3$, 400 MHz

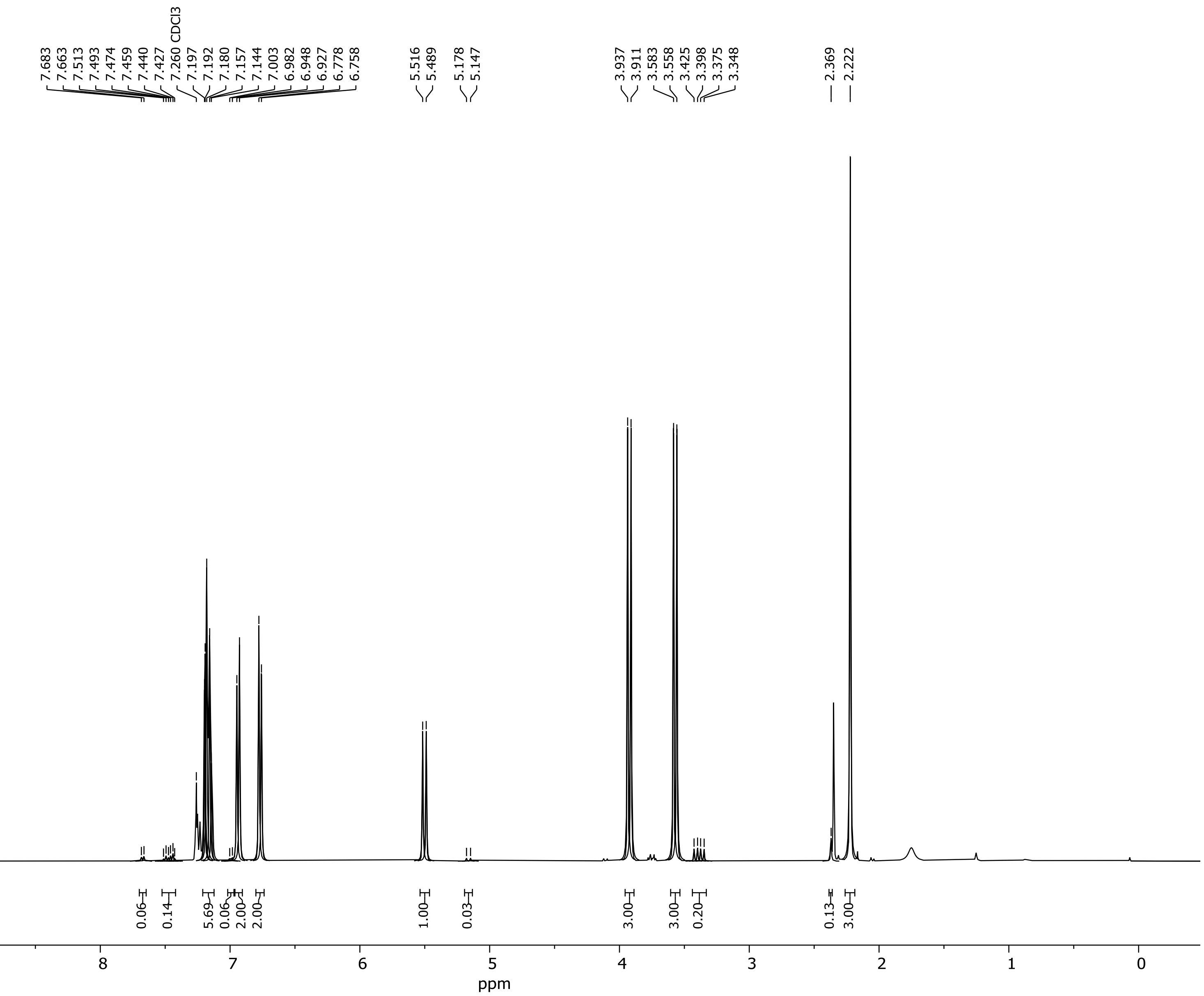

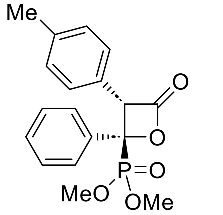

anti-**49**

^{13}C, $CDCl_3$, 101 MHz

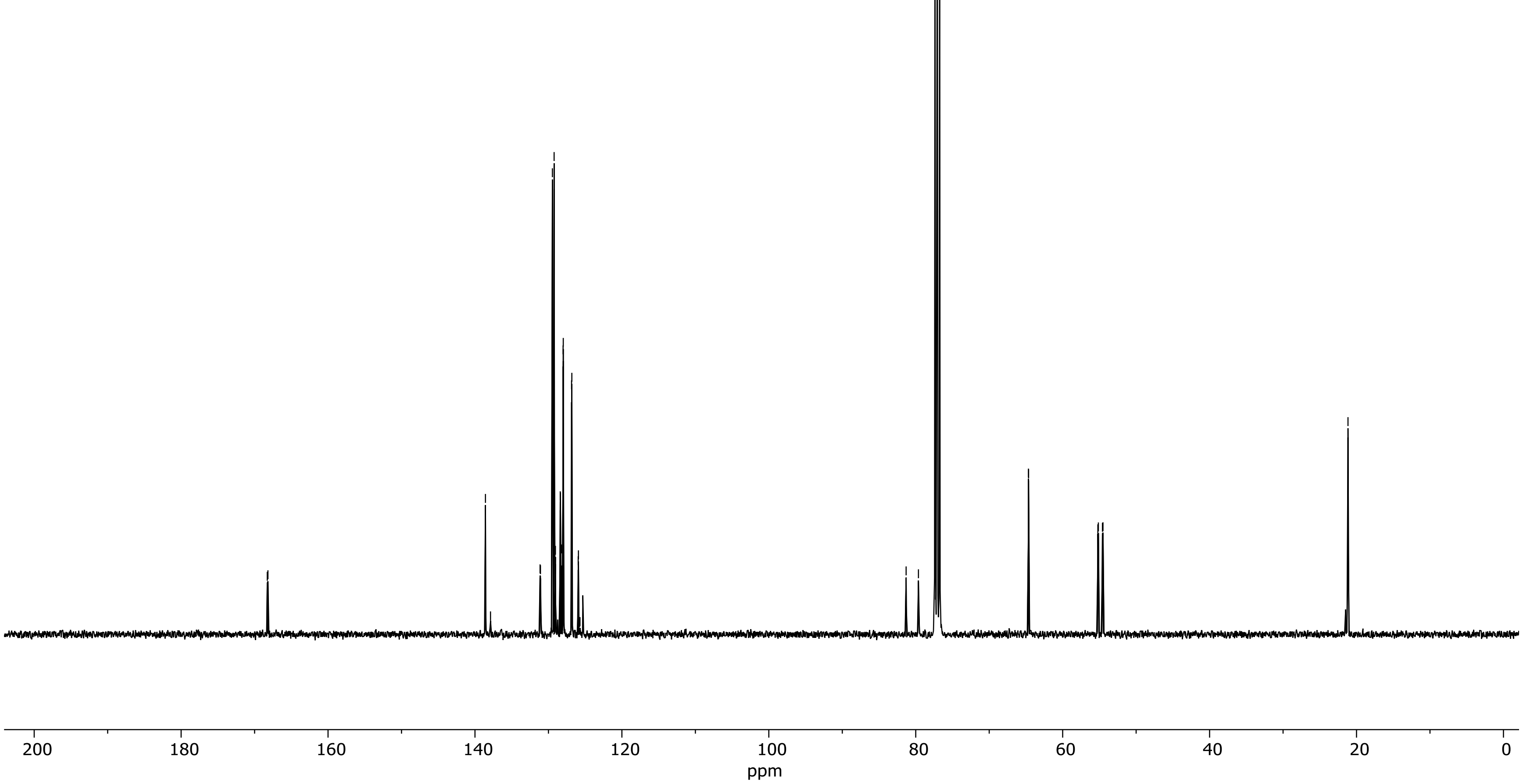

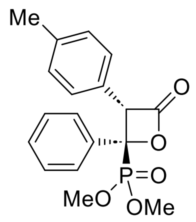

anti-**49**

^{31}P, $CDCl_3$, 162 MHz

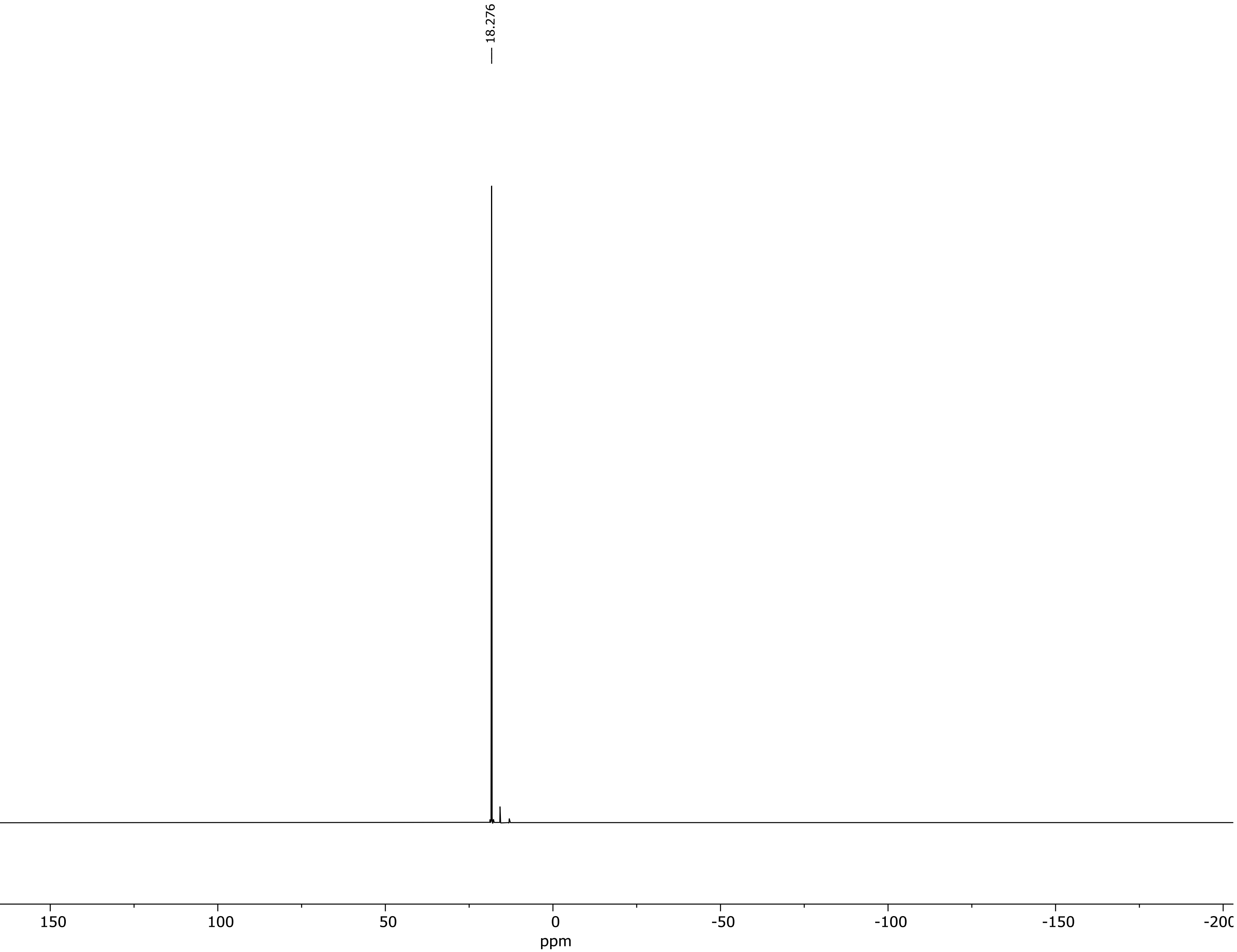

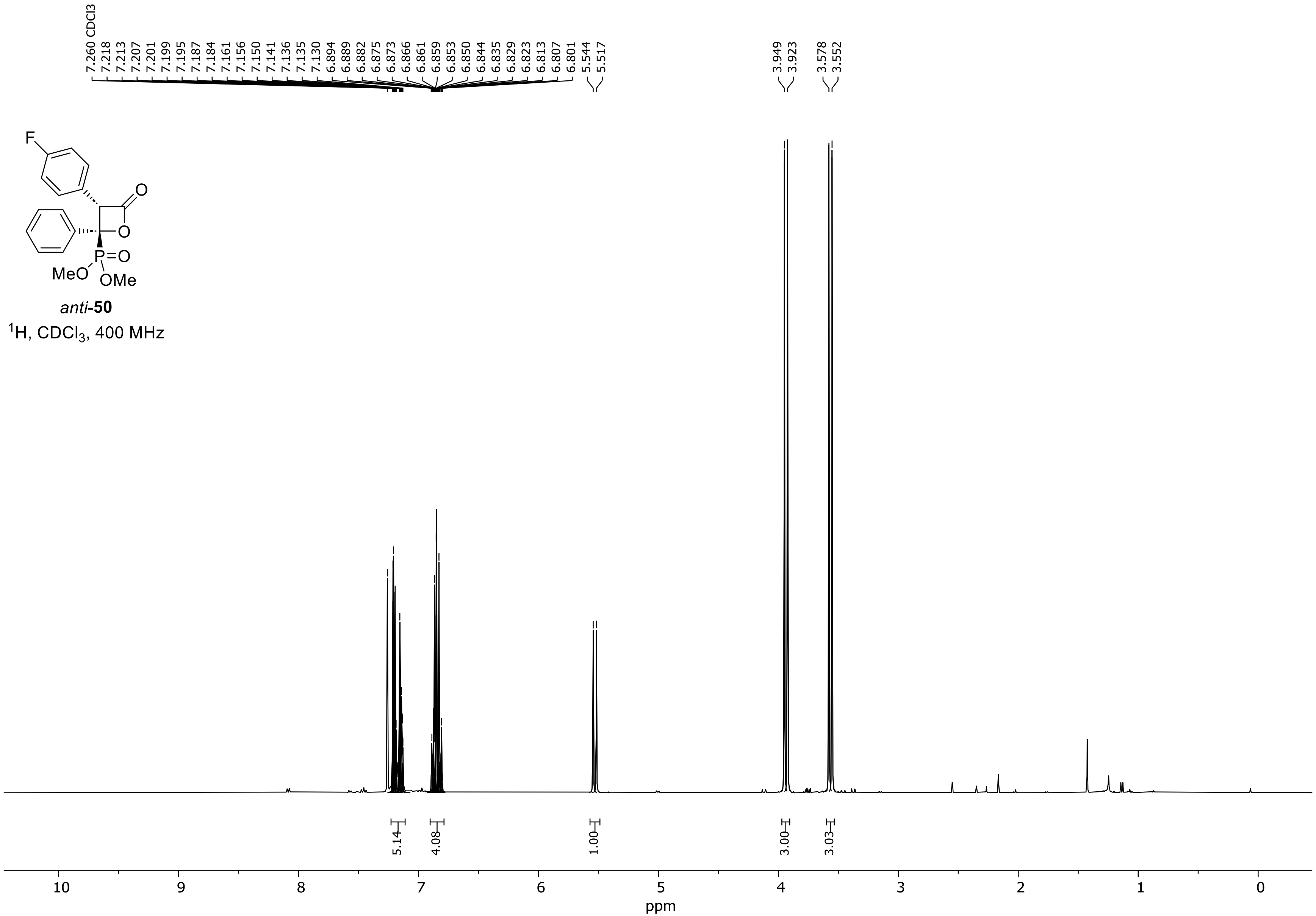
7.260 CDCl3
7.218
7.213
7.207
7.201
7.199
7.195
7.187
7.184
7.161
7.156
7.150
7.141
7.136
7.135
7.130
6.894
6.889
6.882
6.875
6.873
6.866
6.861
6.859
6.853
6.850
6.844
6.835
6.829
6.823
6.813
6.807
6.801
5.544
5.517
3.949
3.923
3.578
3.552
F
O
O
P=O
MeO
OMe
anti-50
1H, CDCl3, 400 MHz
5.14
4.08
1.00
3.00
3.03
10
9
8
7
6
5
4
3
2
1
0
ppm

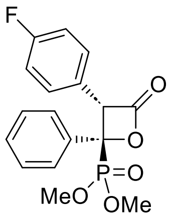

anti-**50**

^{13}C, $CDCl_3$, 101 MHz

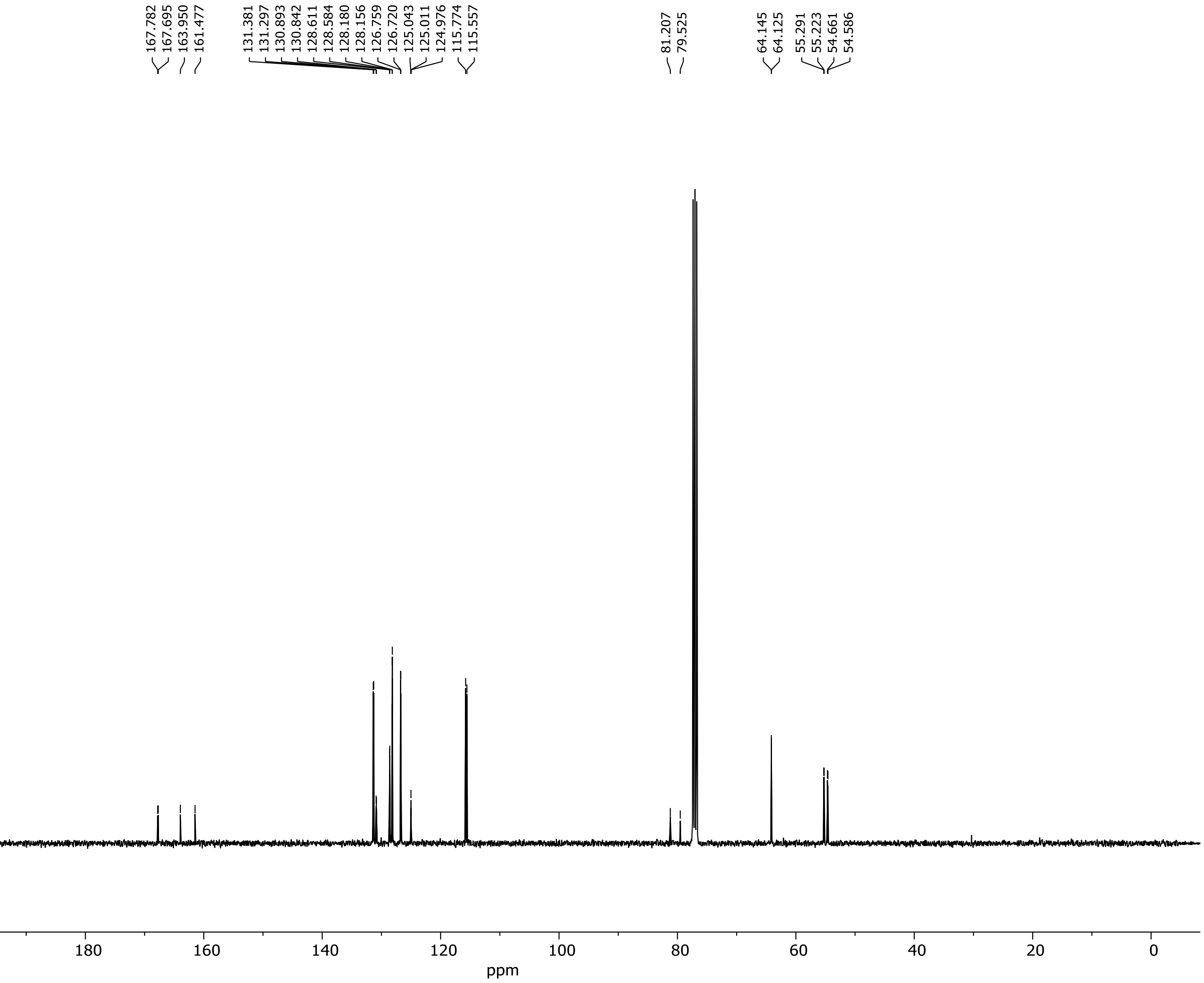

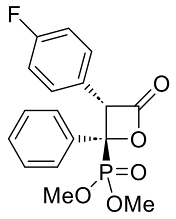

anti-**50**

^{31}P, $CDCl_3$, 162 MHz

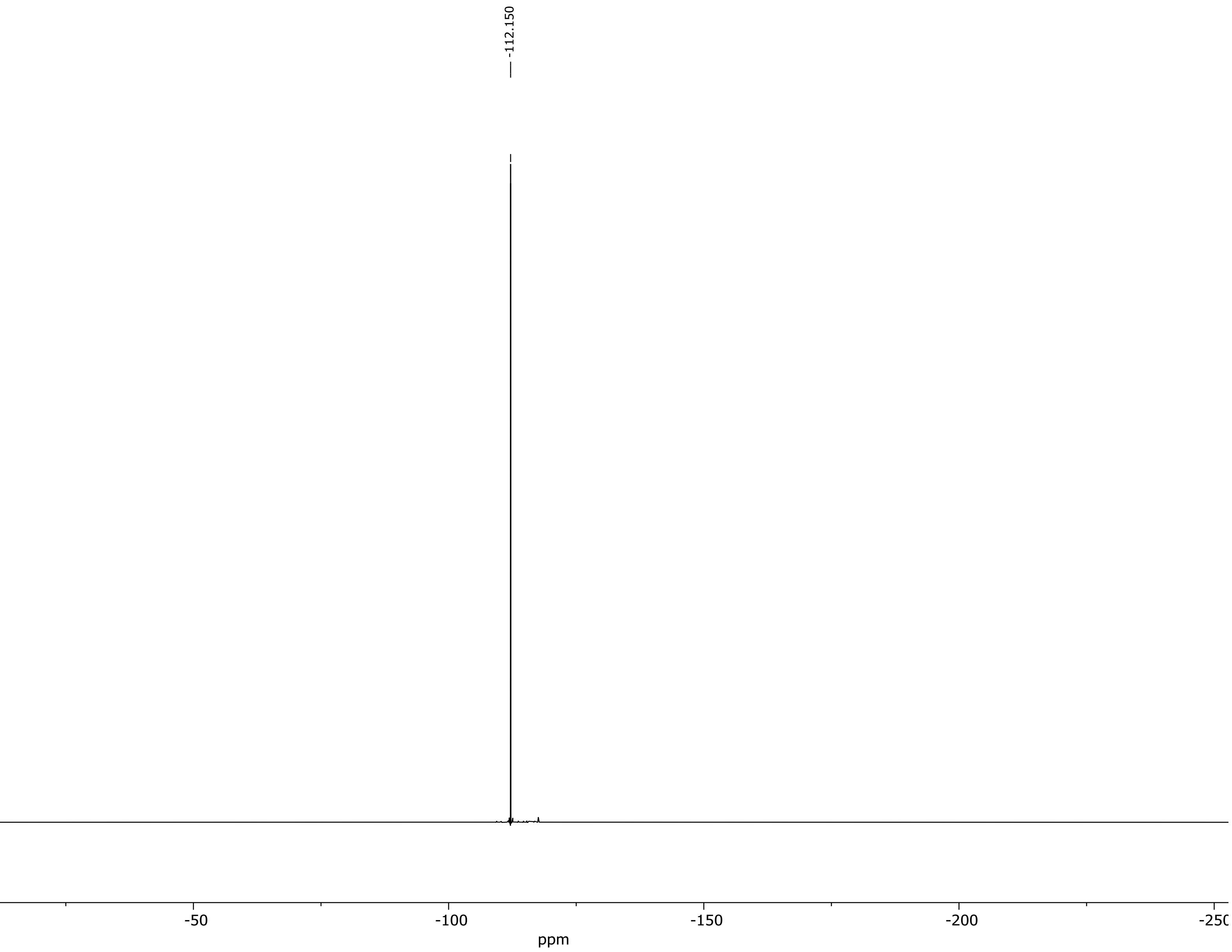

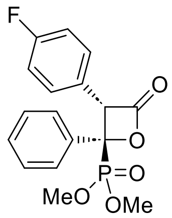

anti-**50**

^{19}F, $CDCl_3$, 376 MHz

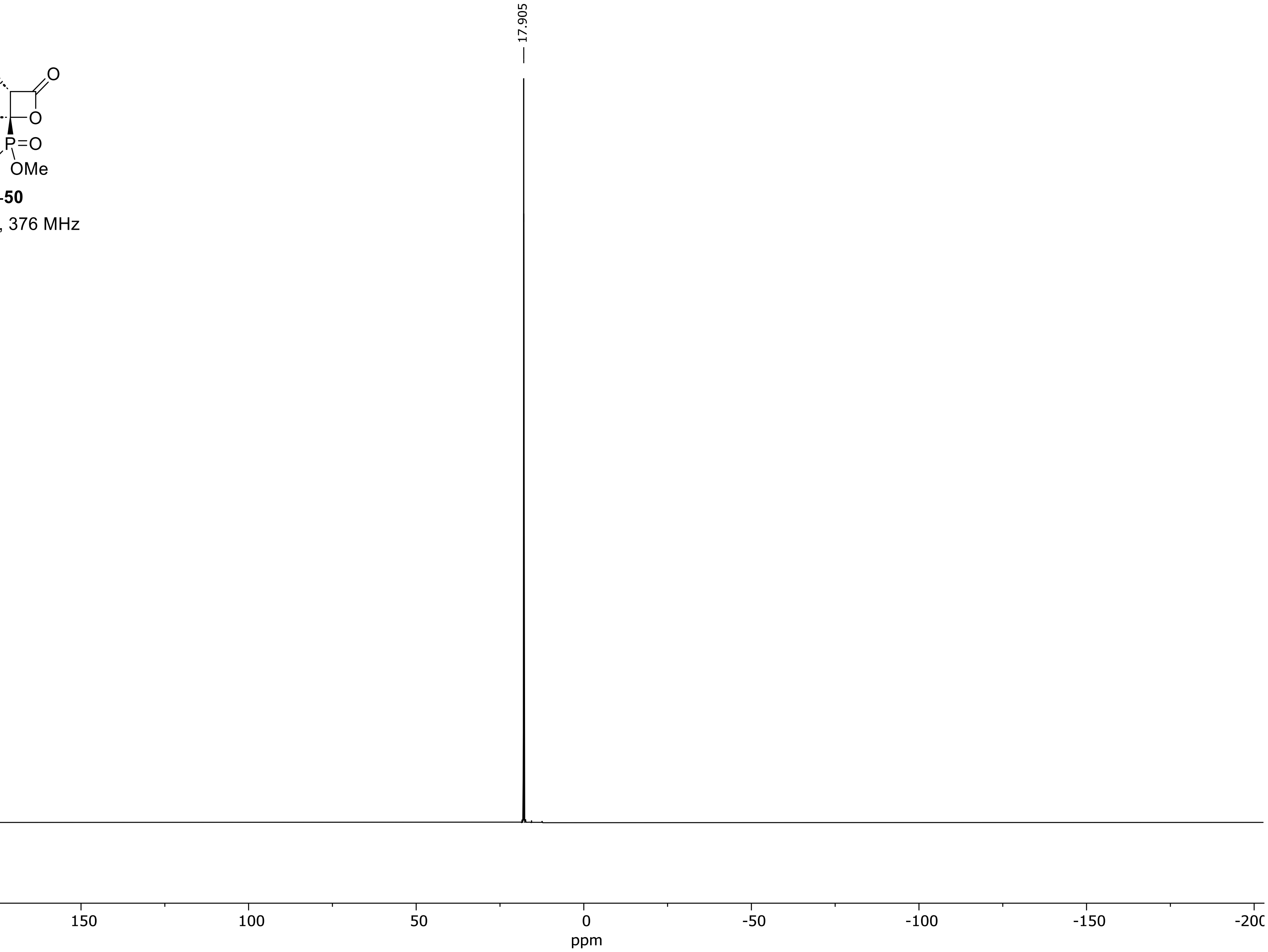

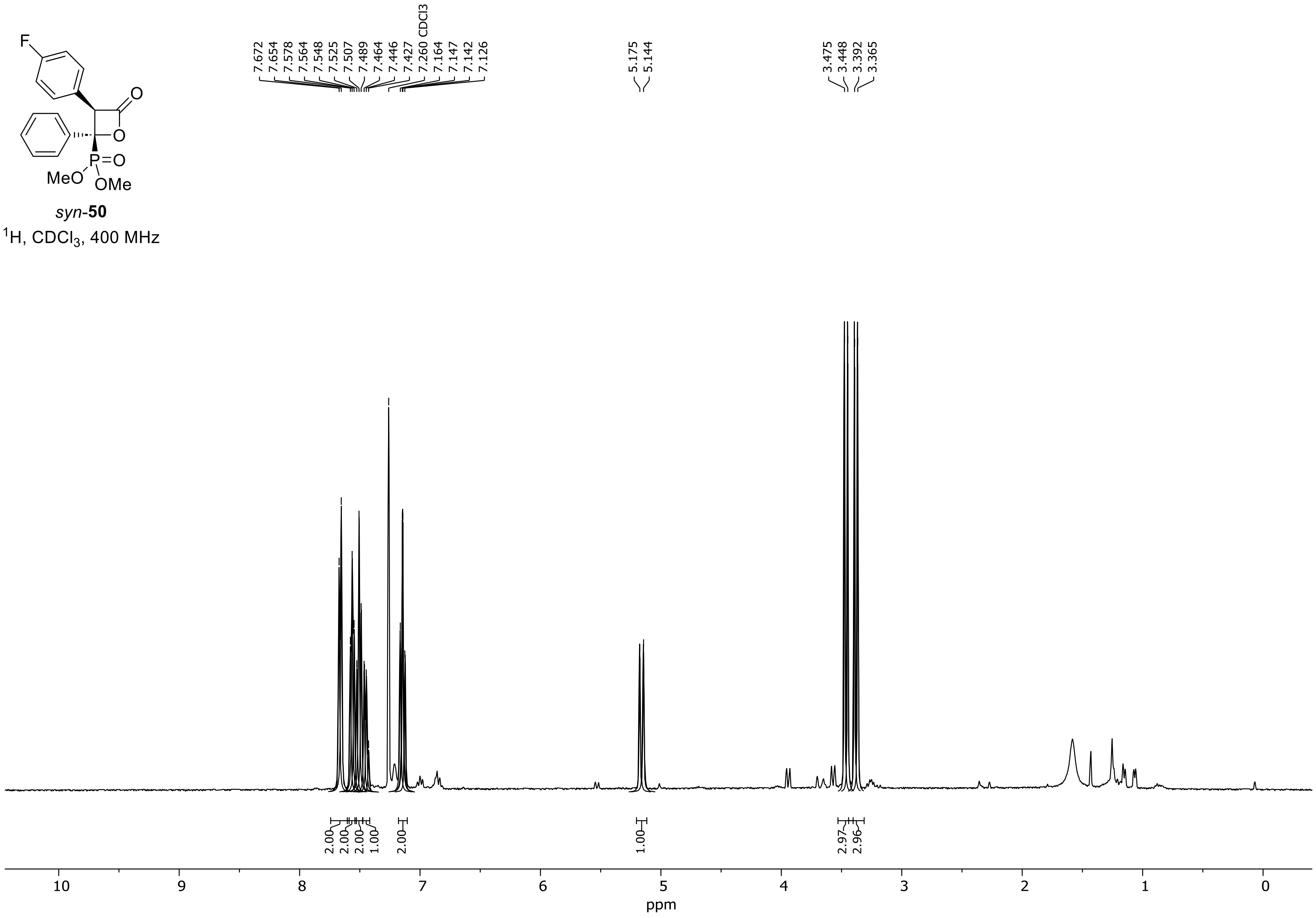

syn-**50**

^{1}H, $CDCl_3$, 400 MHz

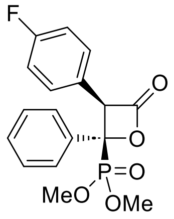

syn-**50**

^{13}C, $CDCl_3$, 101 MHz

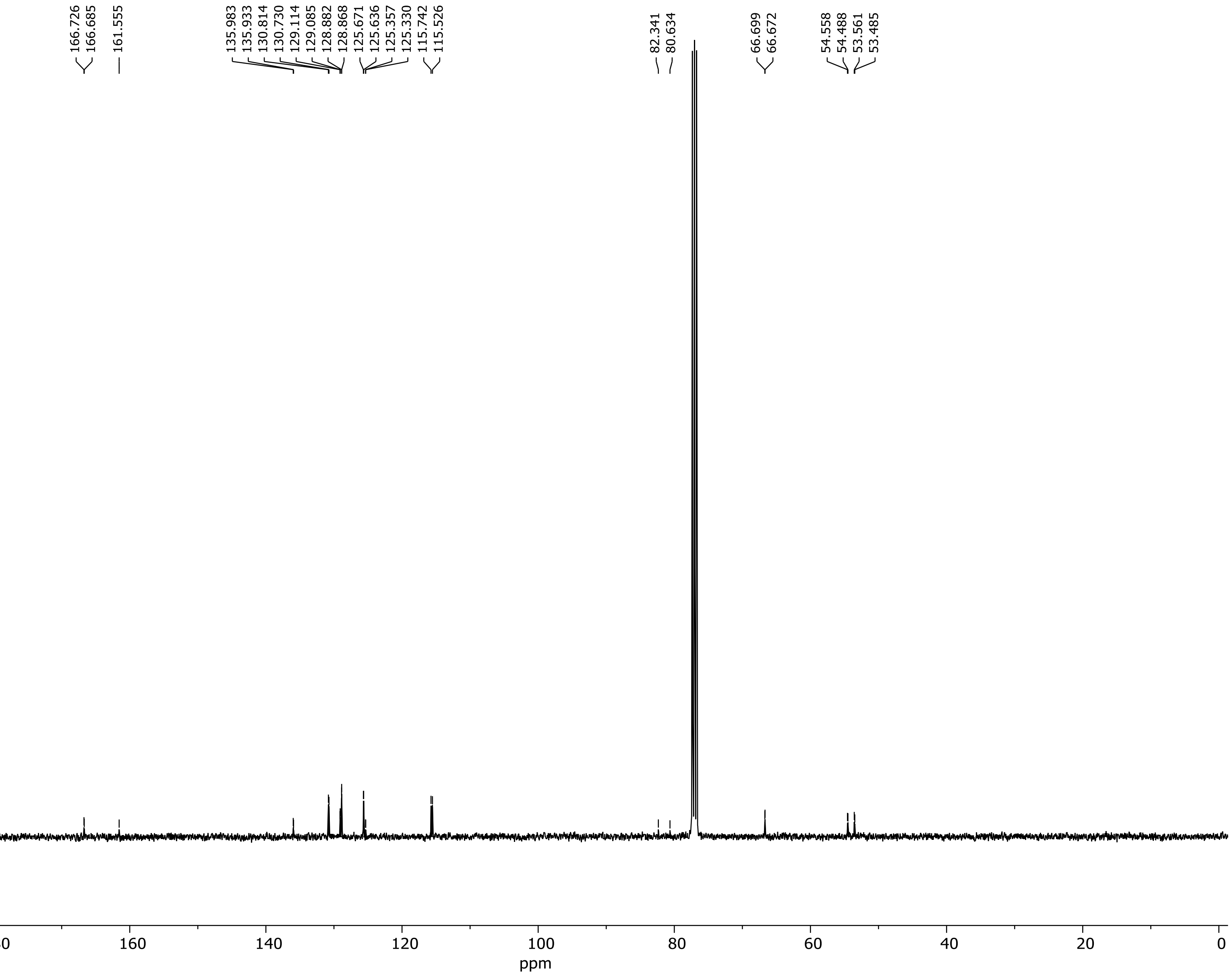

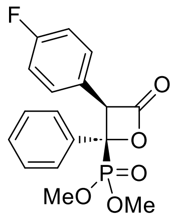

syn-**50**

^{31}P, $CDCl_3$, 162 MHz

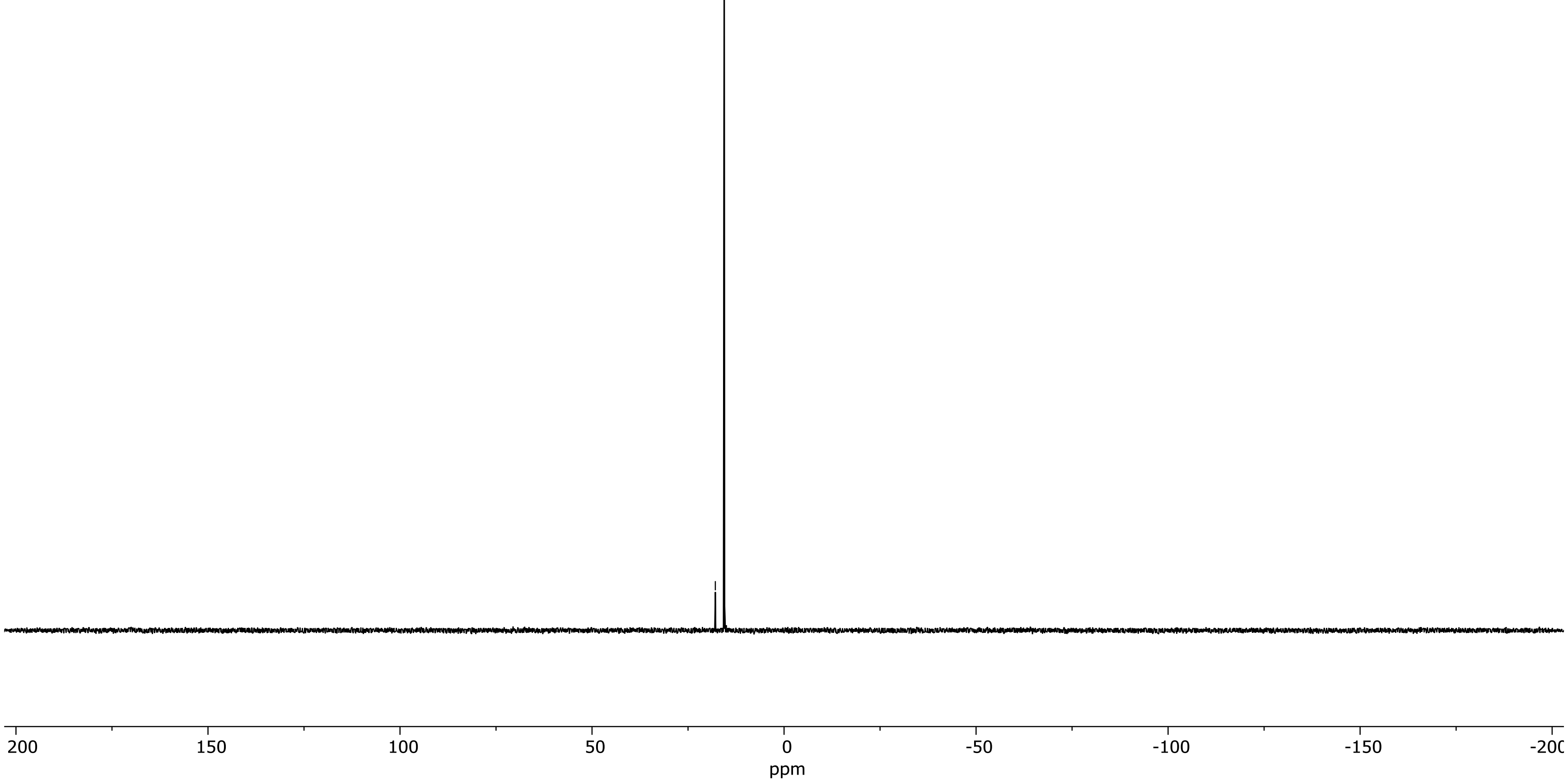

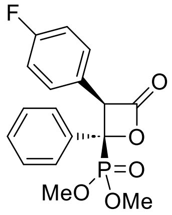

syn-**50**

^{19}F, $CDCl_3$, 376 MHz

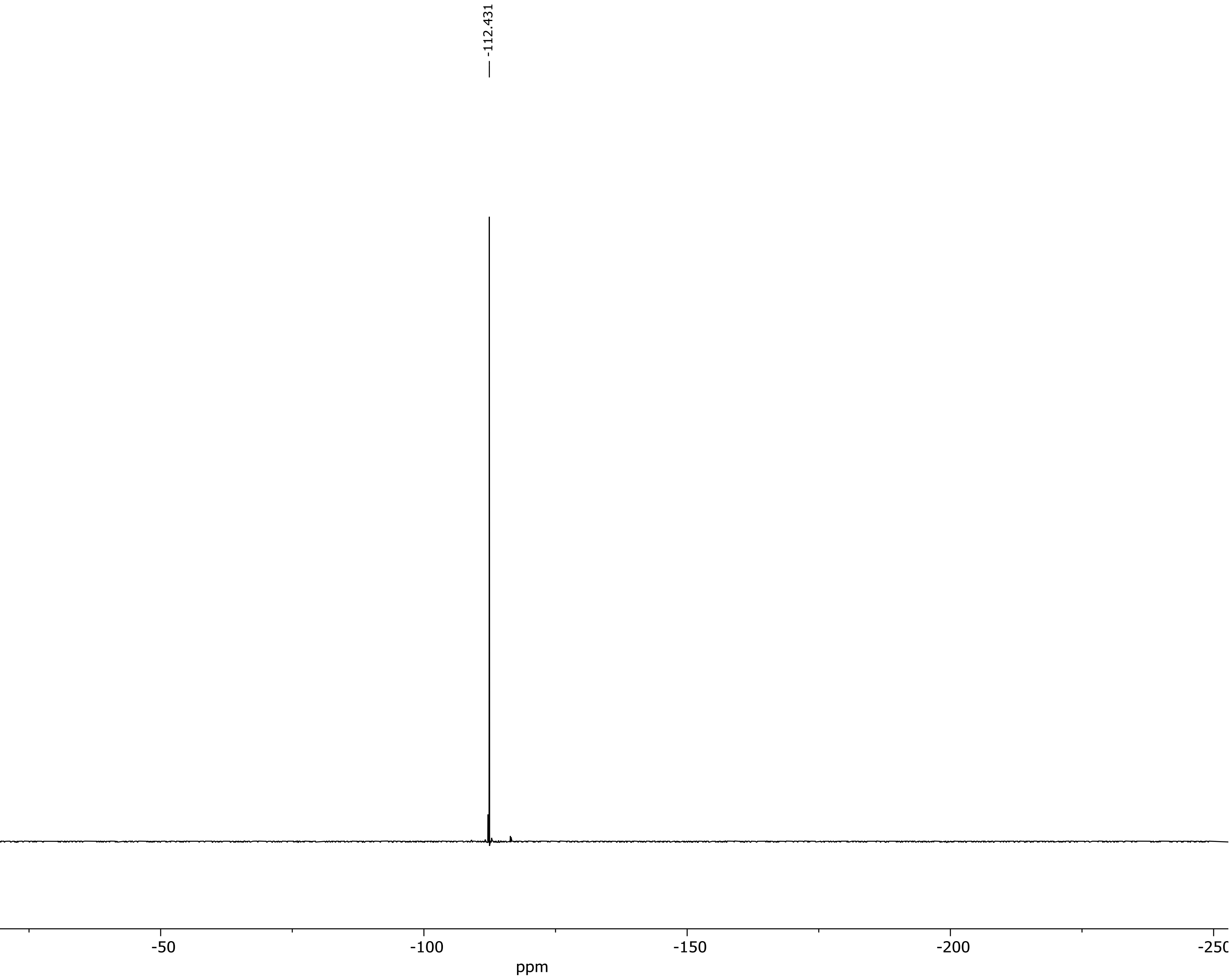

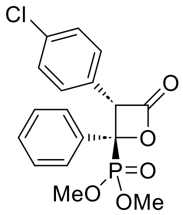

anti-**51**

^{1}H, $CDCl_3$, 400 MHz

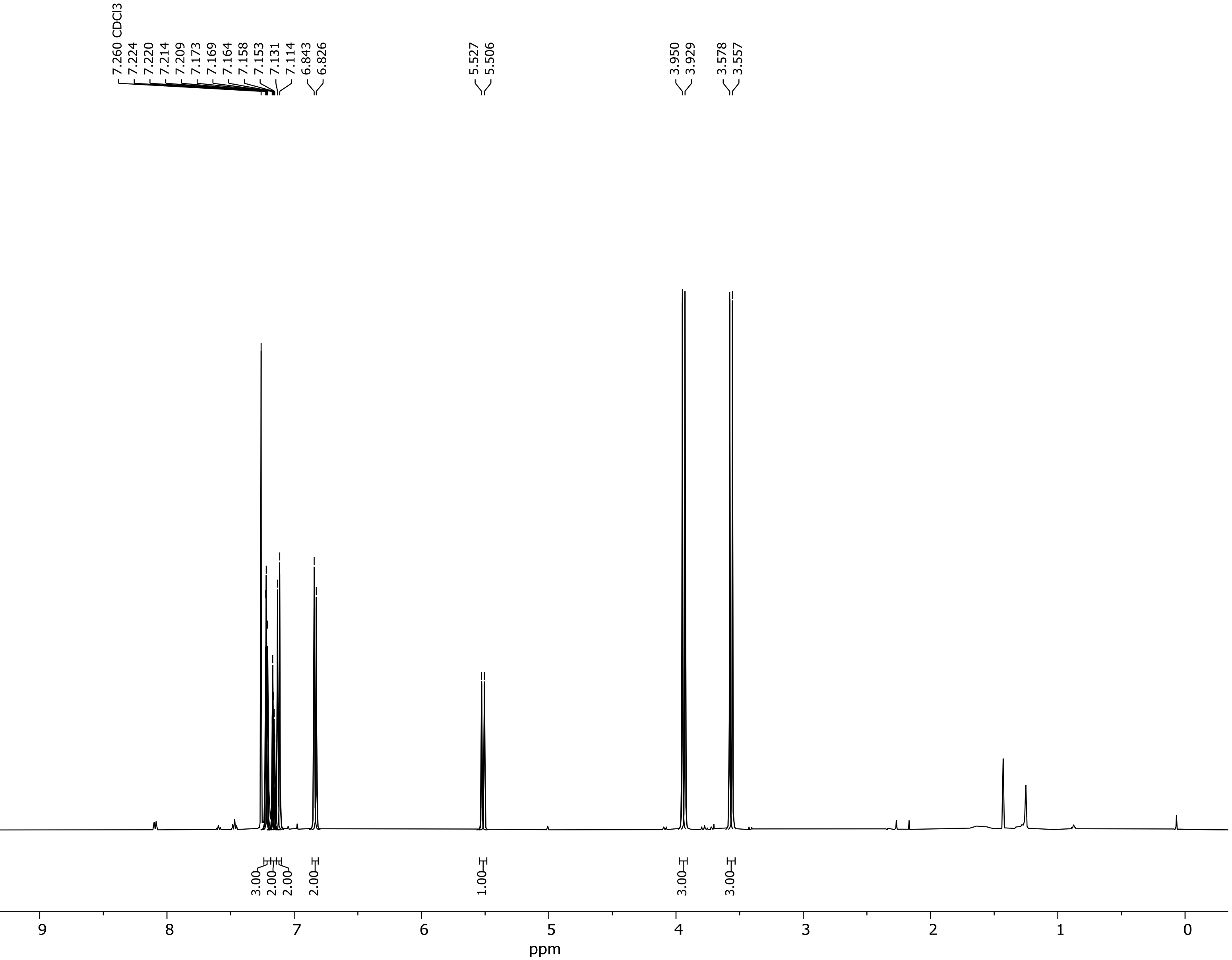

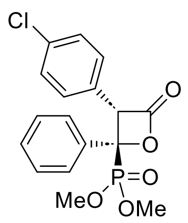

anti-**51**

^{13}C, $CDCl_3$, 101 MHz

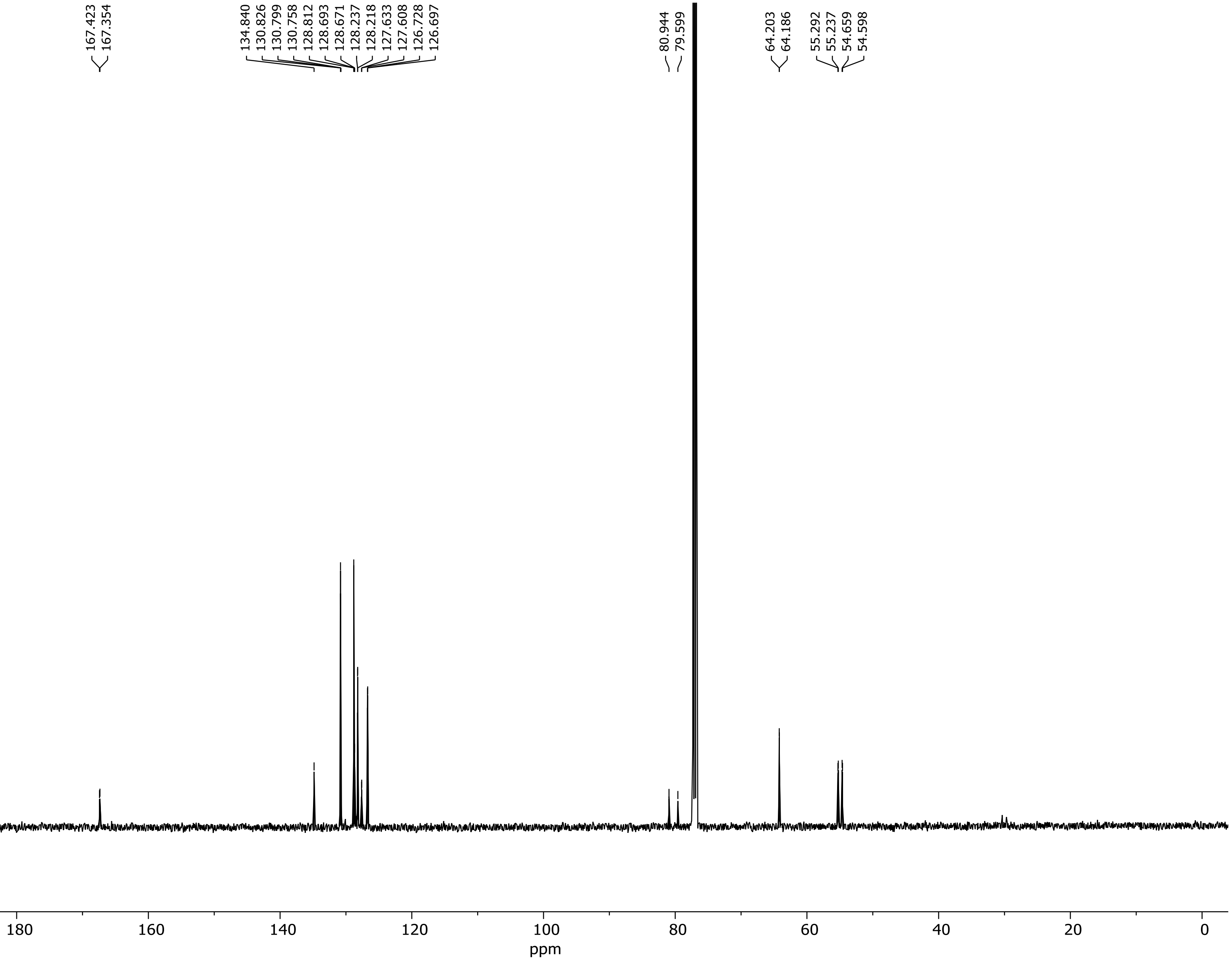

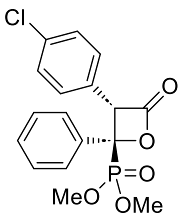

anti-**51**

^{31}P, $CDCl_3$, 162 MHz

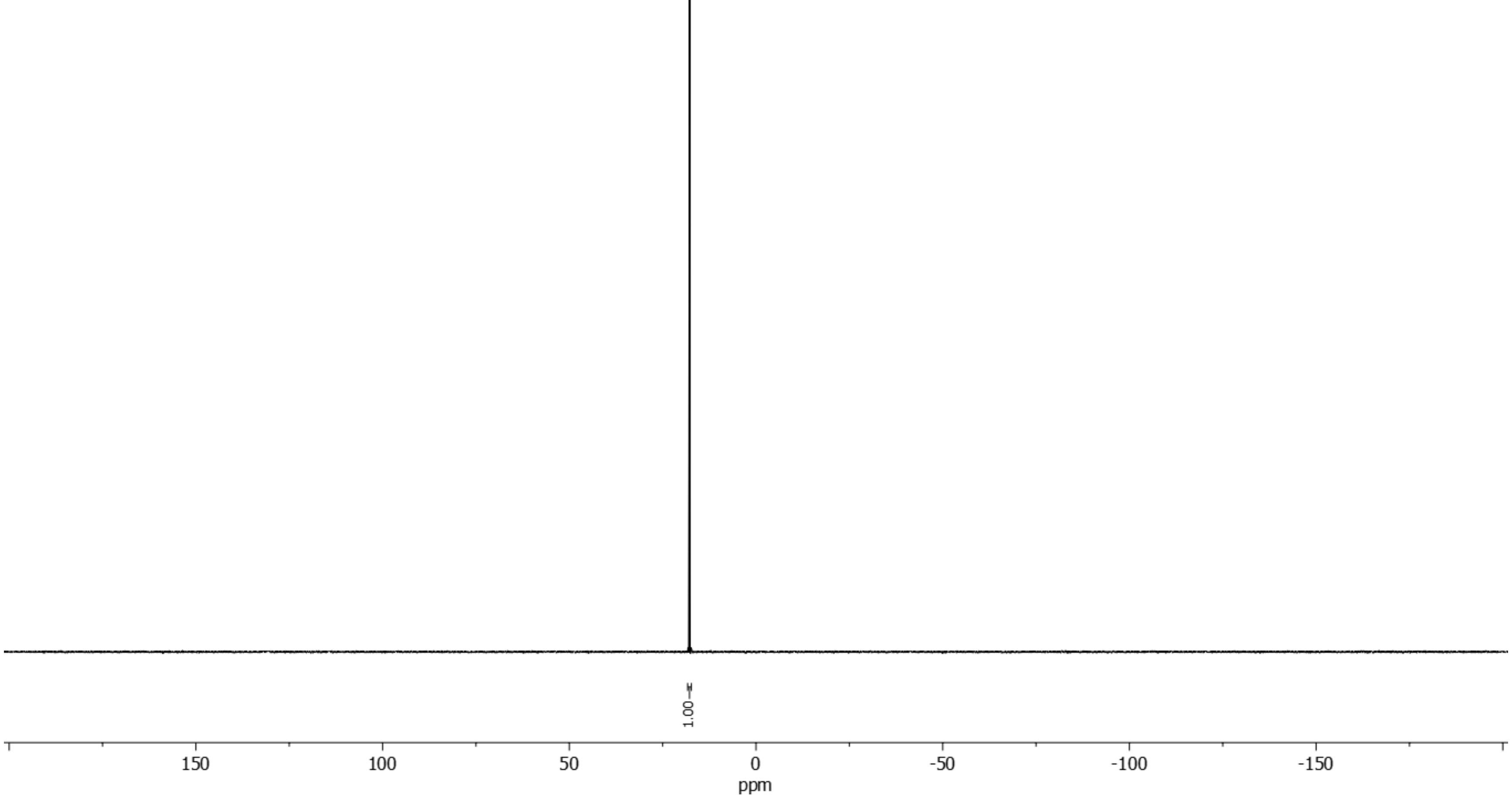

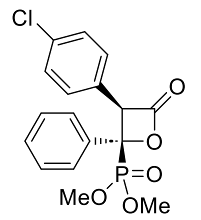

syn-**51**

^{1}H, $CDCl_3$, 400 MHz

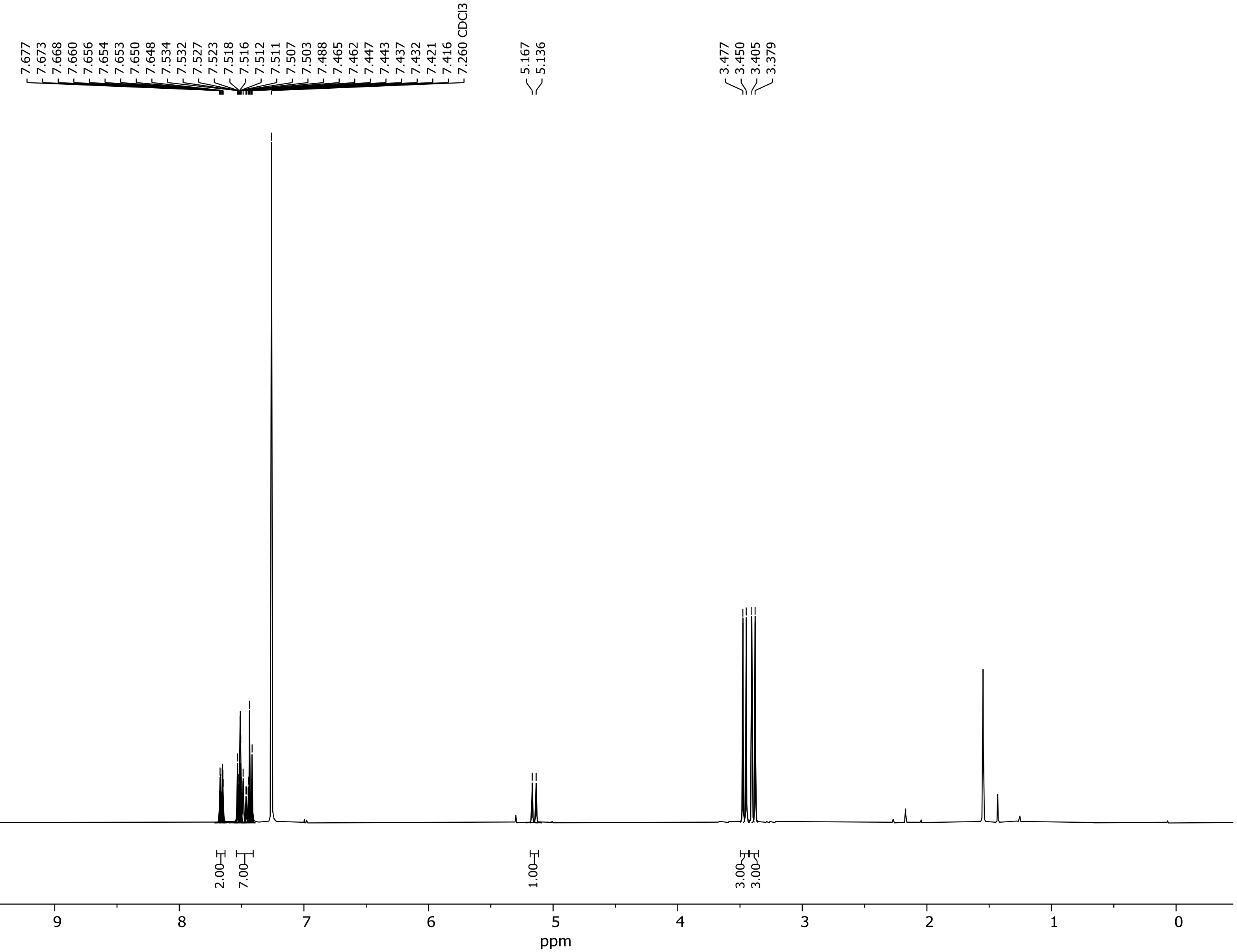

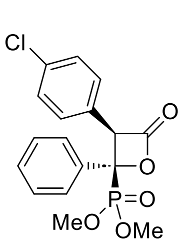

syn-**51**

^{13}C, $CDCl_3$, 101 MHz

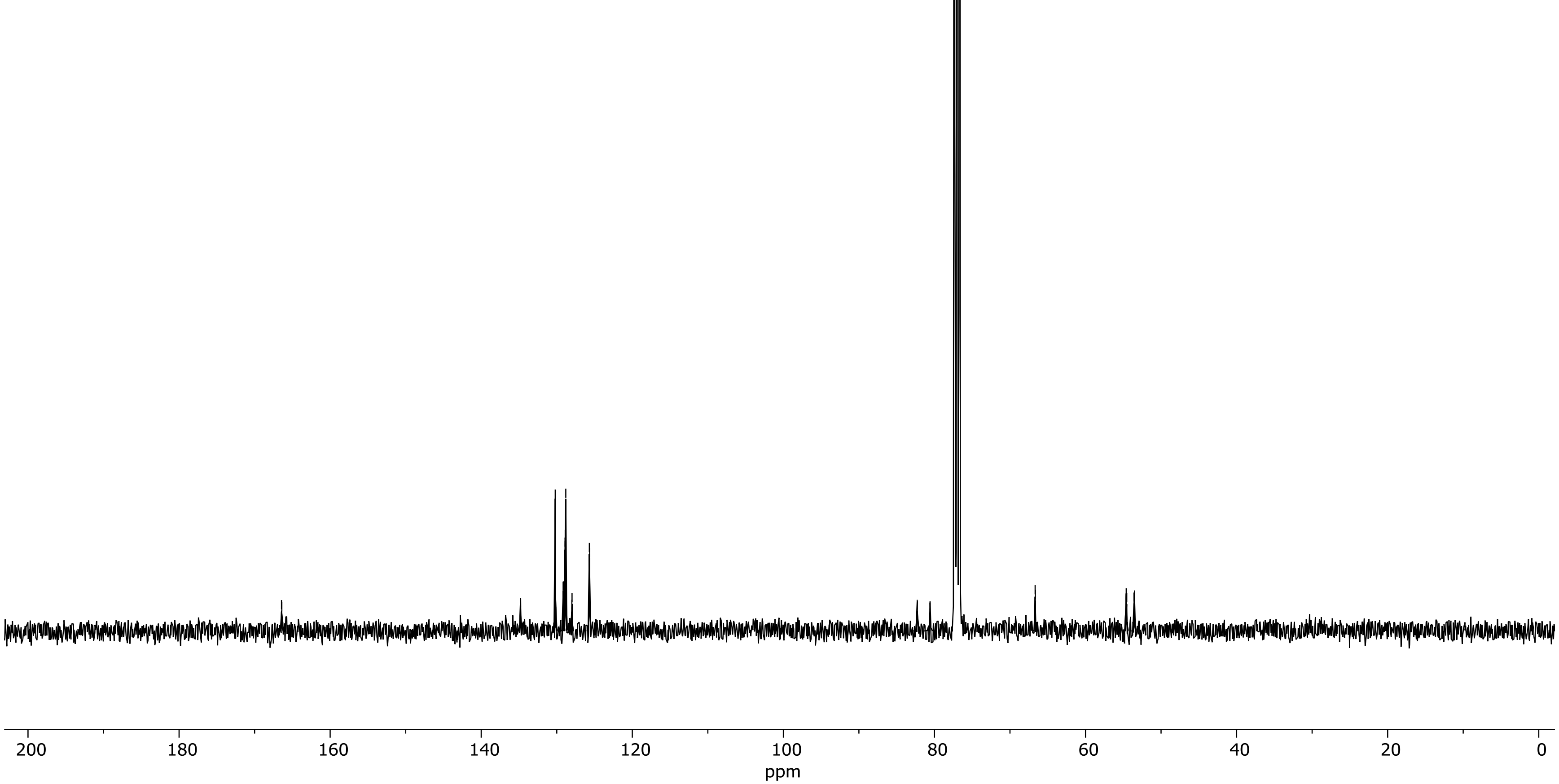

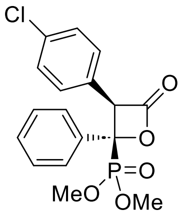

syn-**51**

^{31}P, $CDCl_3$, 162 MHz

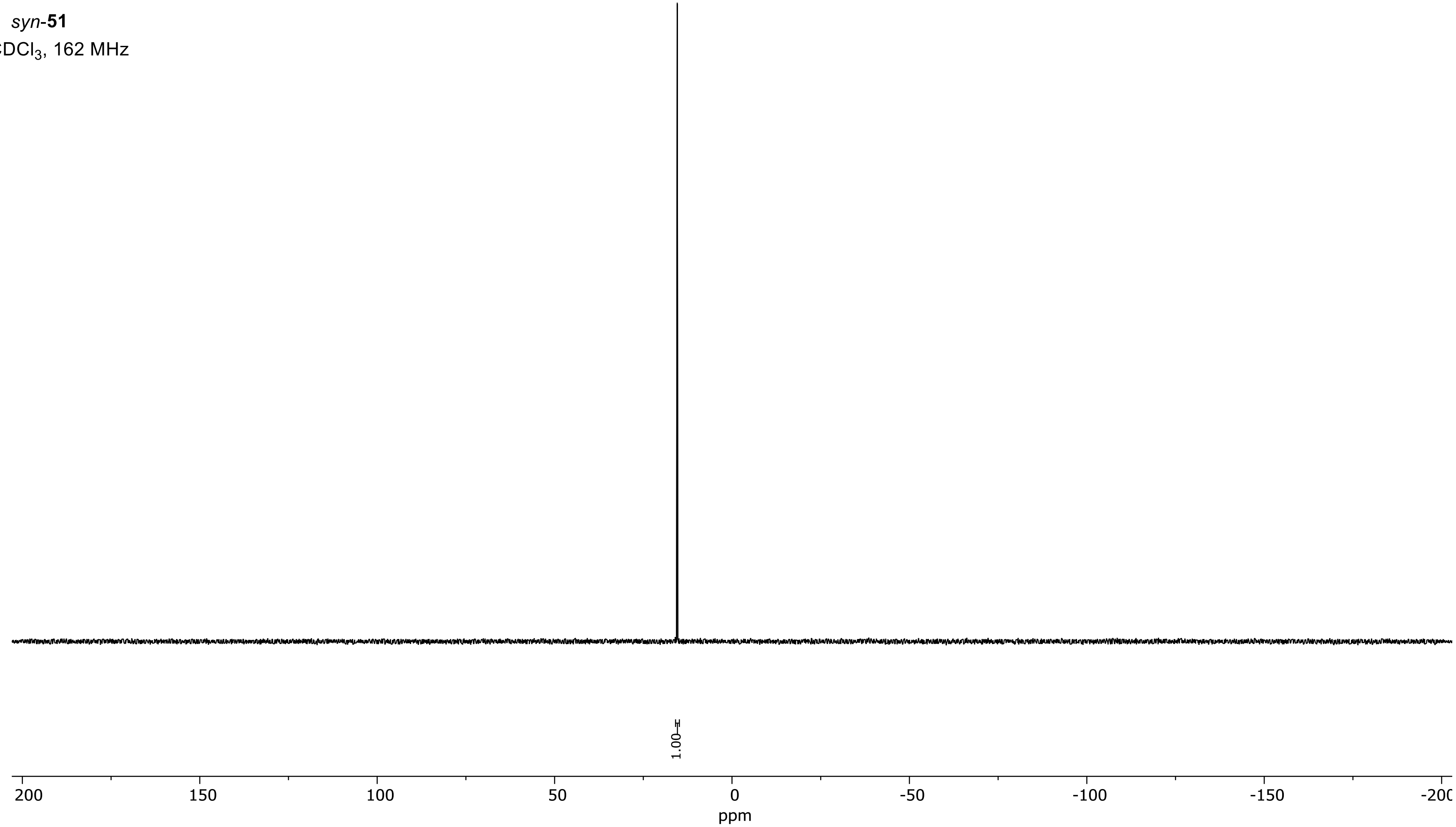

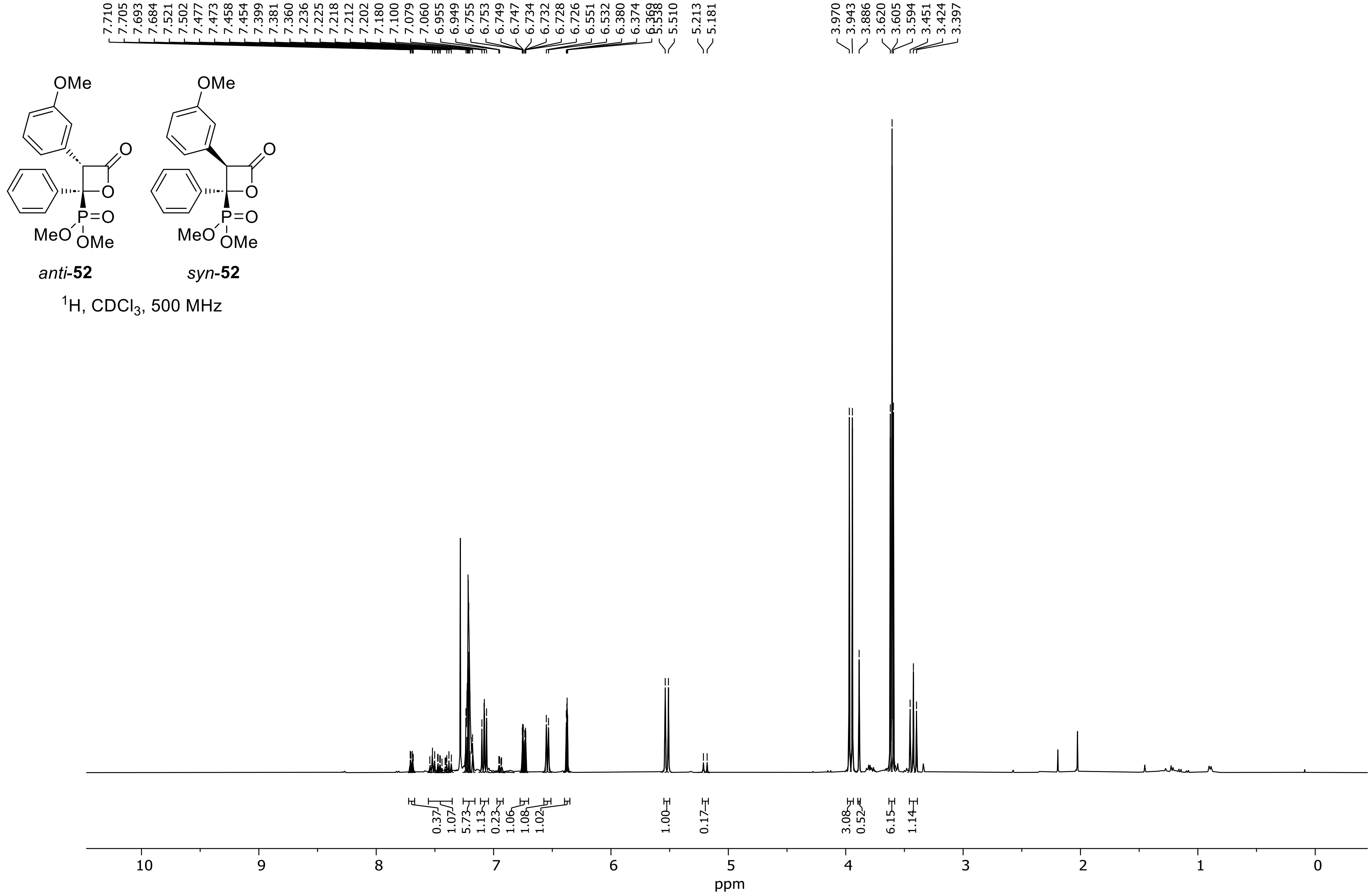
OMe
OMe
O
O
P=O
MeO
OMe
anti-52
syn-52
¹H, CDCl₃, 500 MHz
ppm

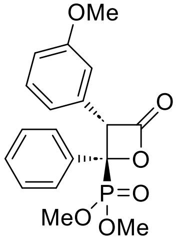

anti-**52**

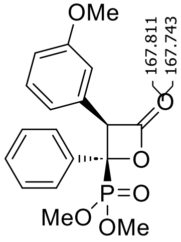

syn-**52**

^{13}C, $CDCl_3$, 126 MHz

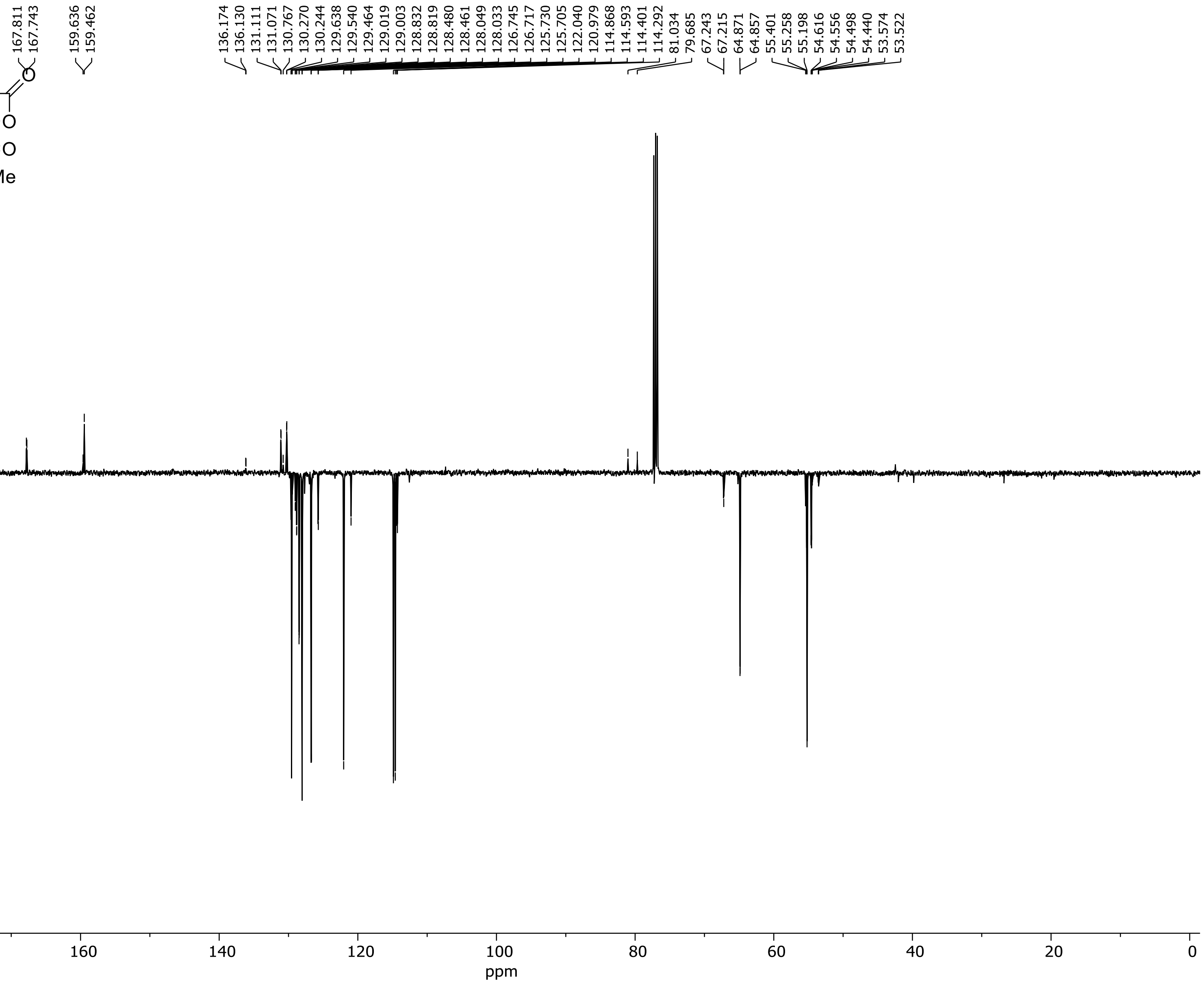

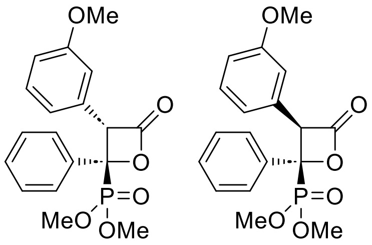

anti-**52** *syn*-**52**

^{31}P, $CDCl_3$, 202 MHz

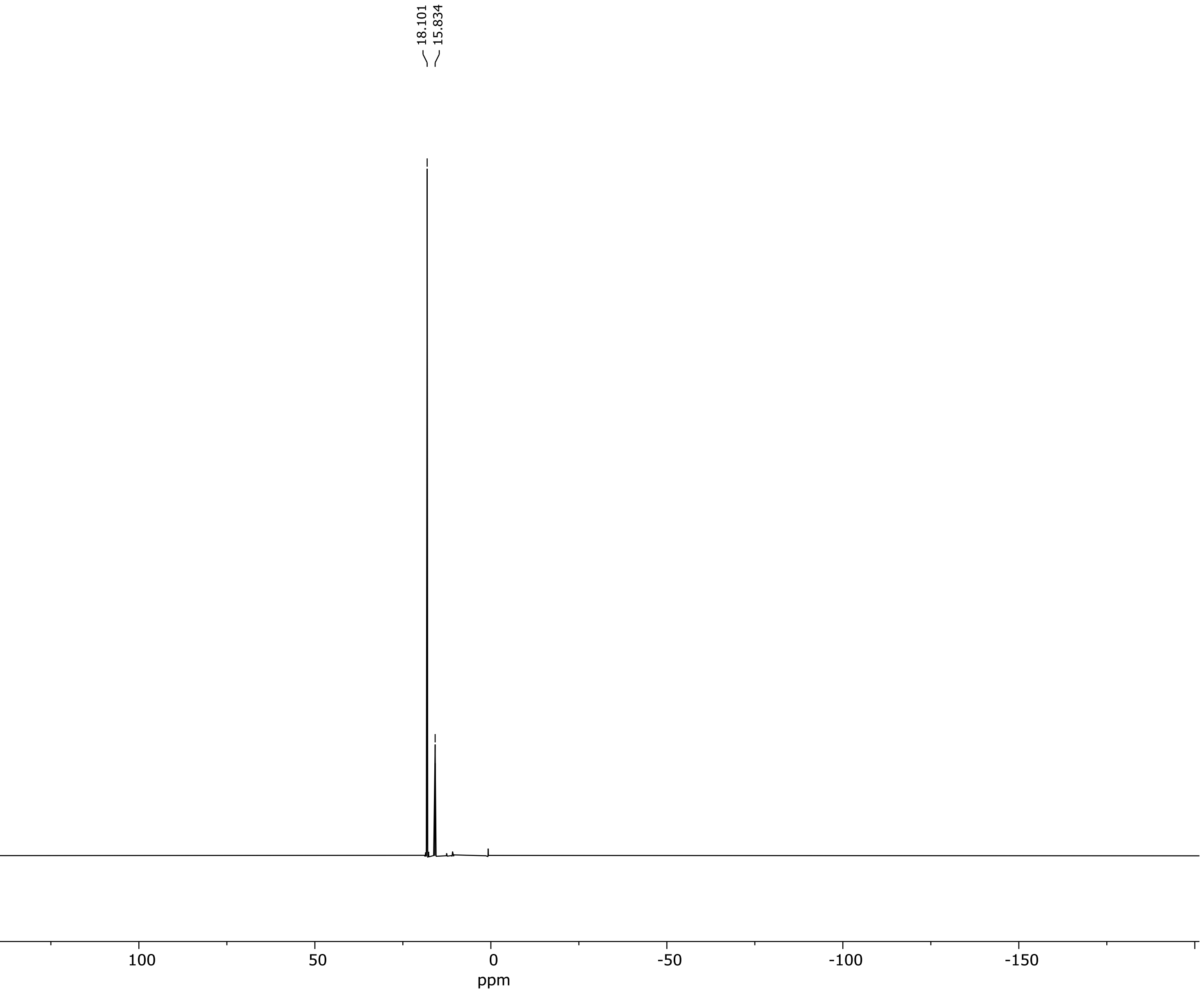

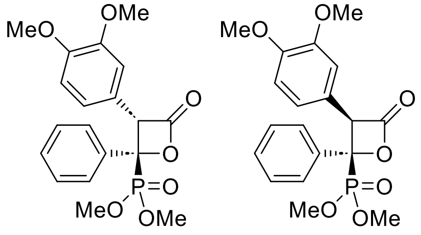

anti-**53** *syn*-**53**

^{1}H, $CDCl_3$, 400 MHz

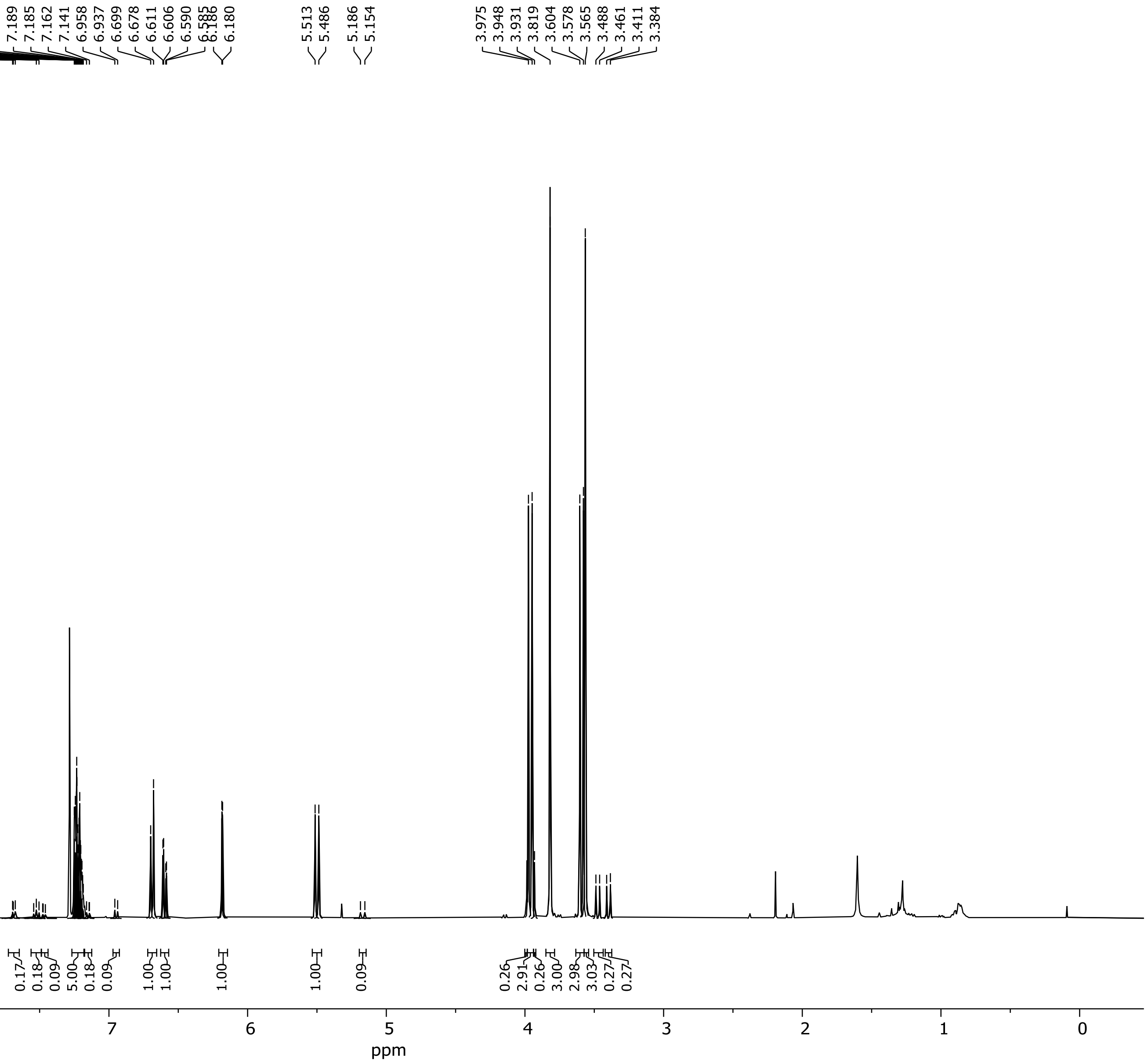

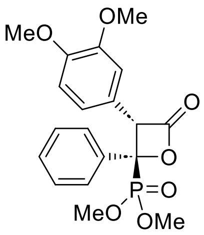

anti-**53**

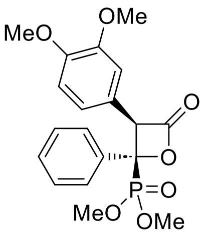

syn-**53**

^{13}C, $CDCl_3$, 101 MHz

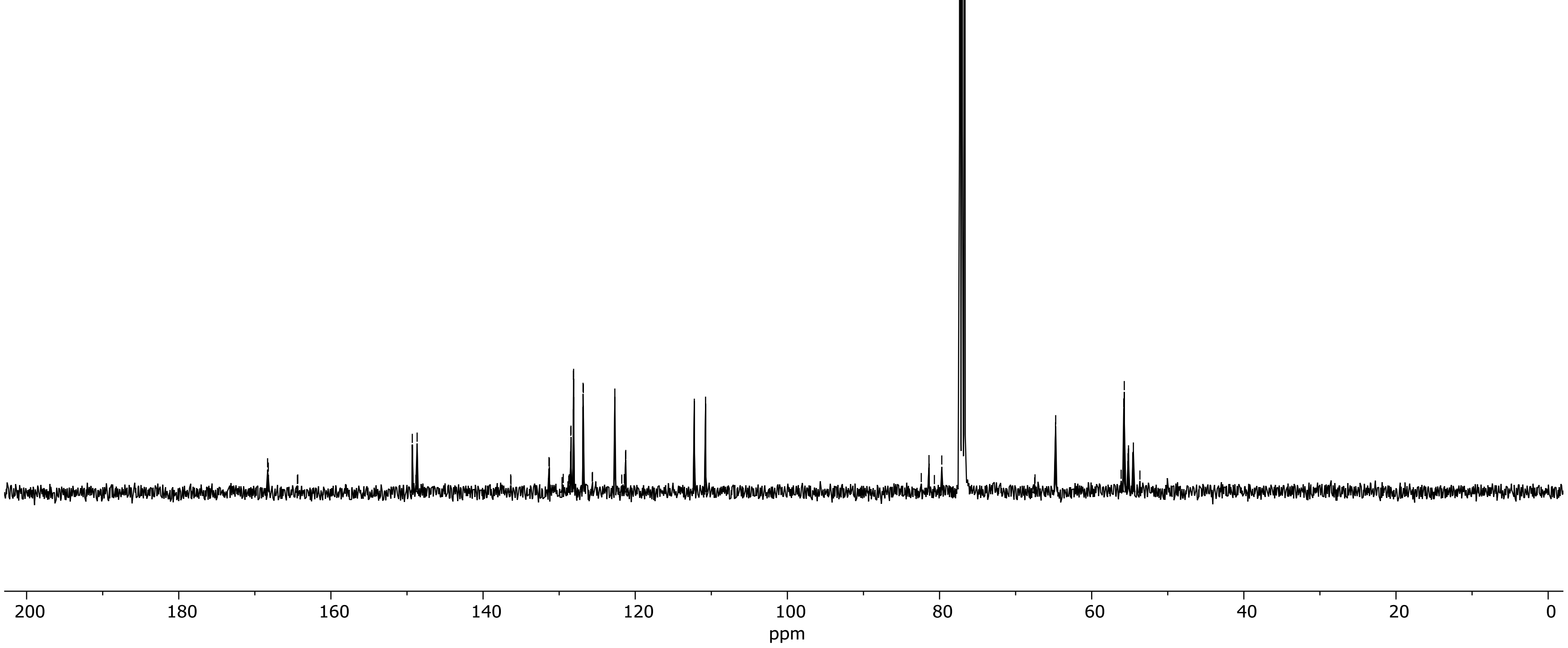

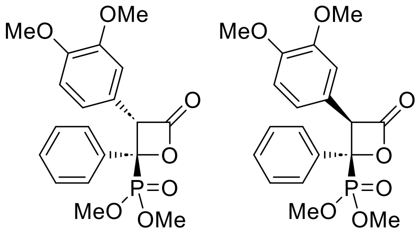

anti-**53** *syn*-**53**

^{31}P, $CDCl_3$, 162 MHz

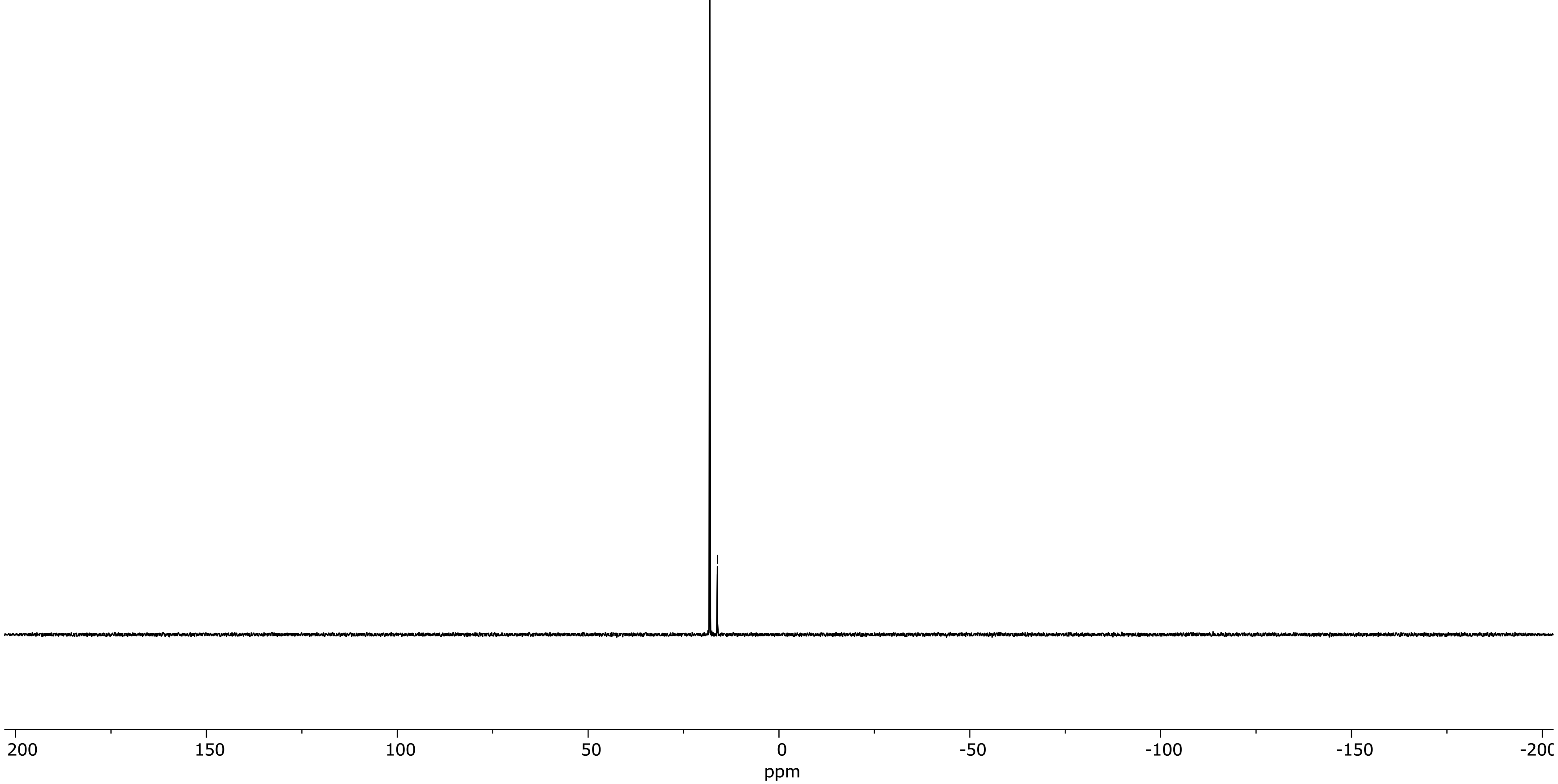

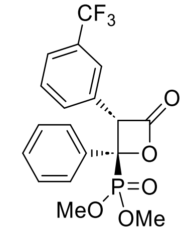

anti-**54**

^{1}H, $CDCl_3$, 400 MHz

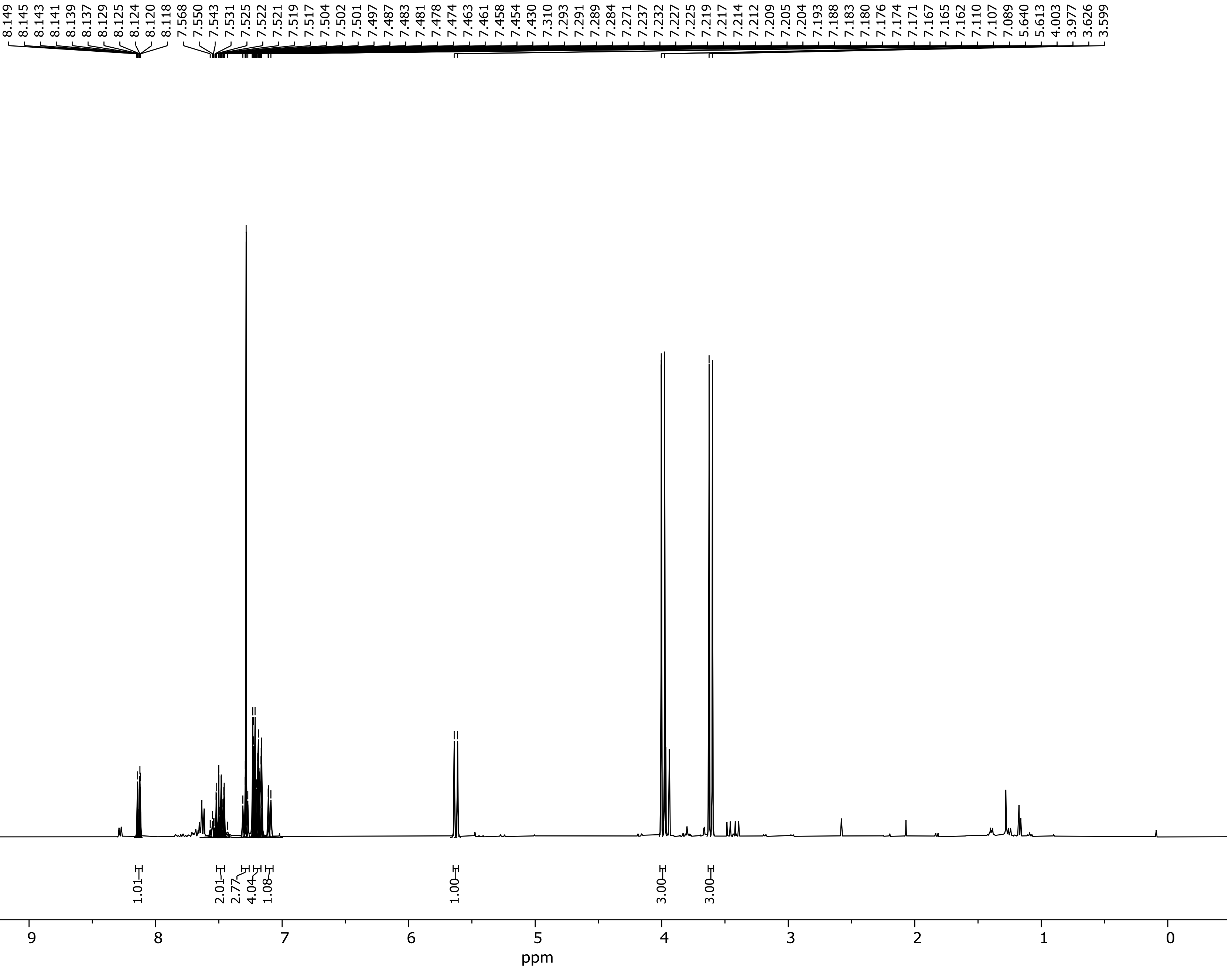

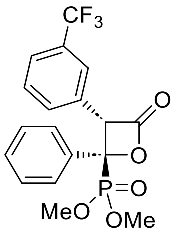

anti-**54**

^{13}C, $CDCl_3$, 101 MHz

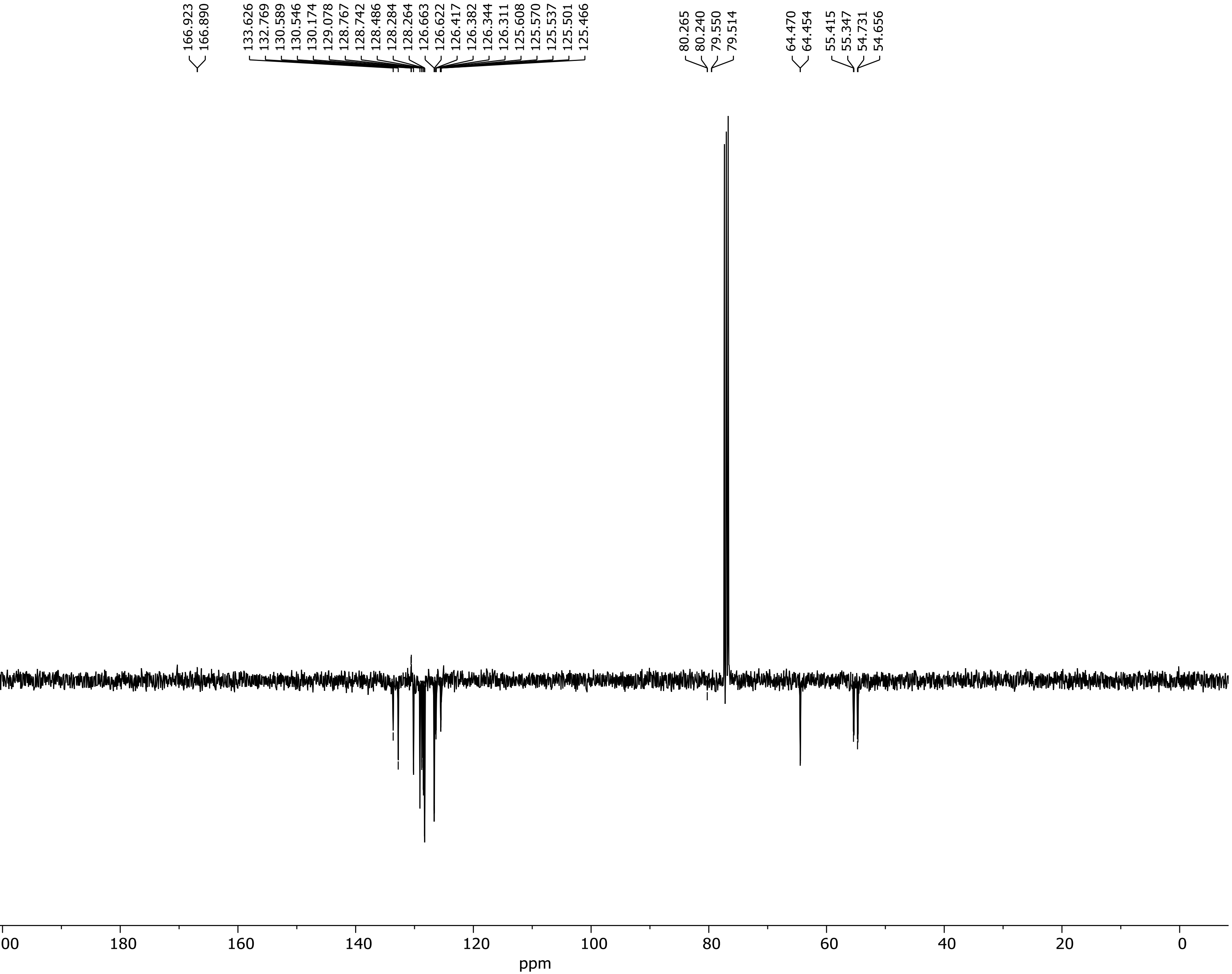

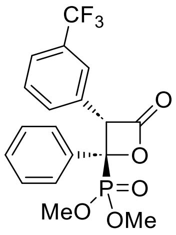

anti-**54**

^{31}P, $CDCl_3$, 162 MHz

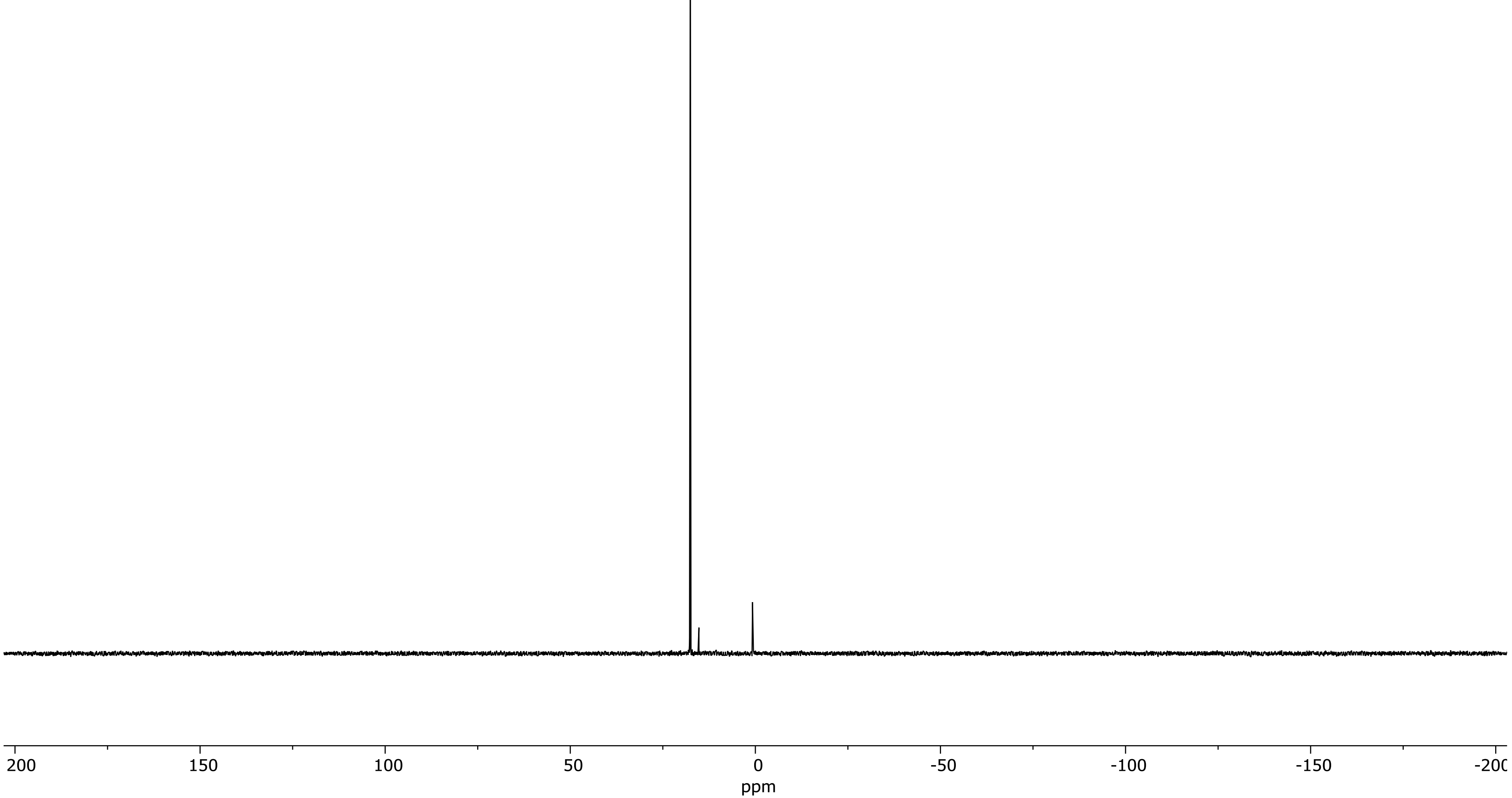

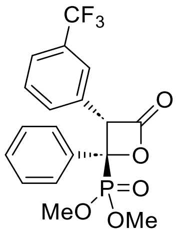

anti-**54**

^{19}F, $CDCl_3$, 376 MHz

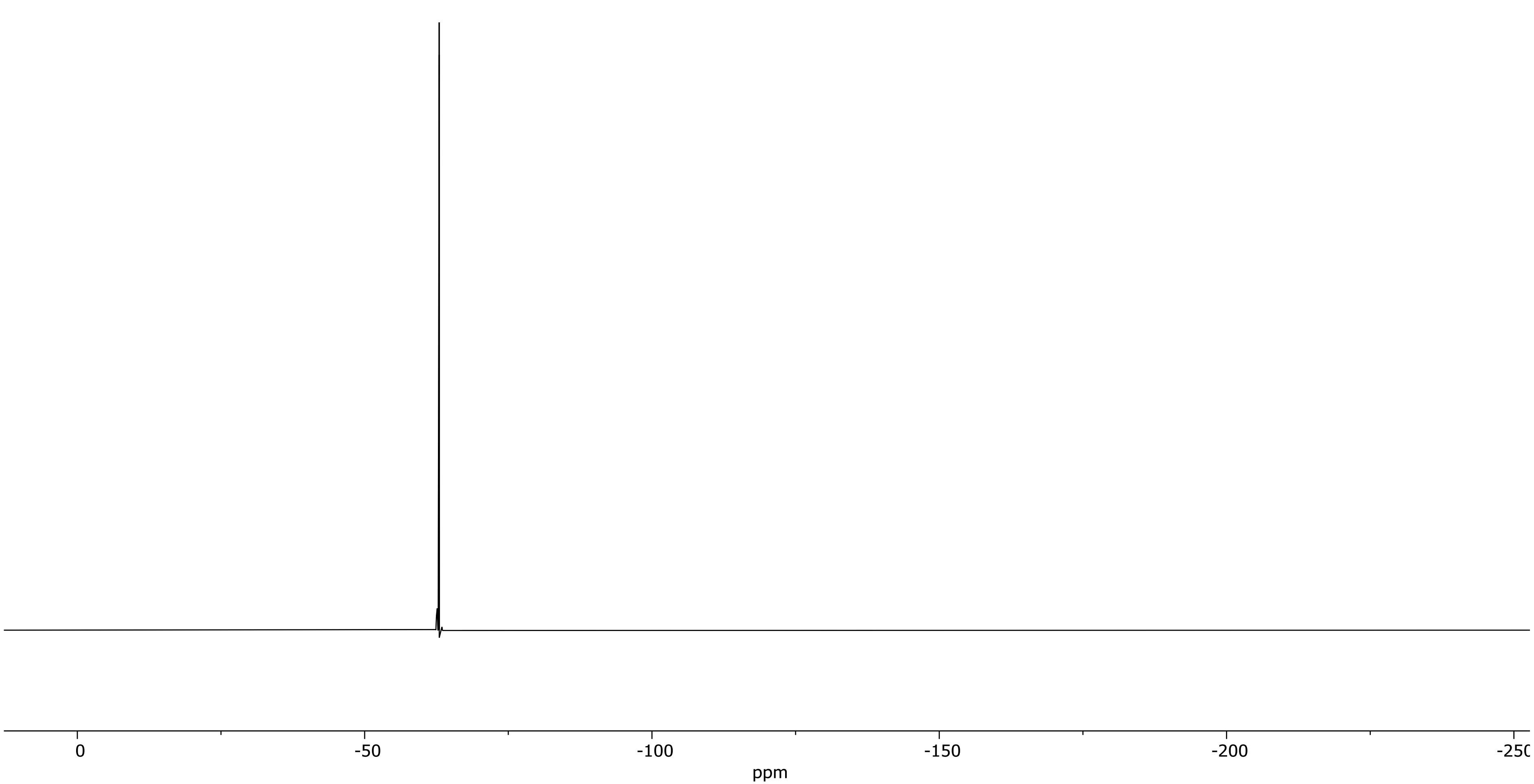

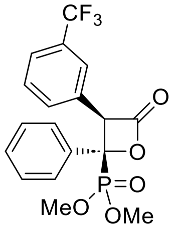

syn-**54**

^1H, $CDCl_3$, 400 MHz

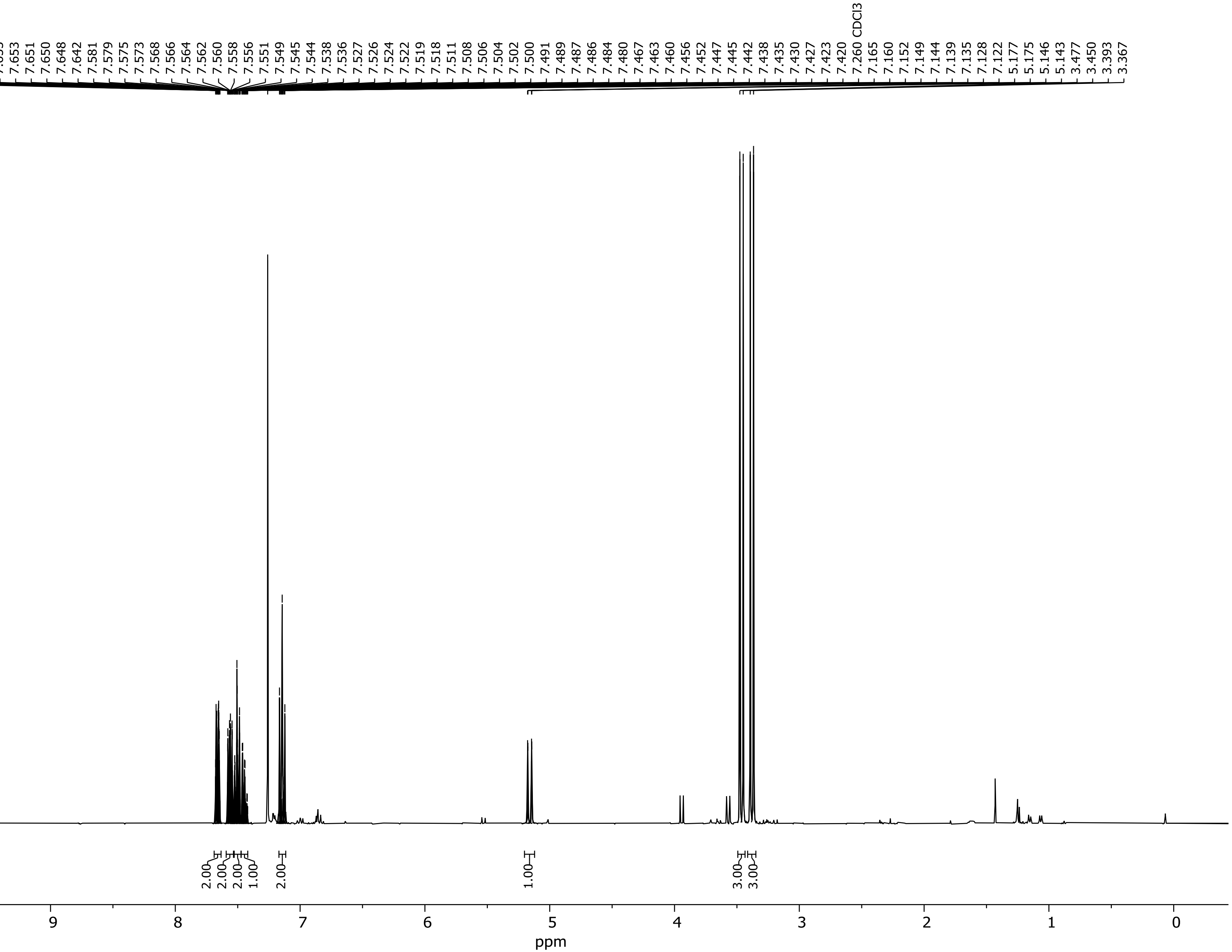

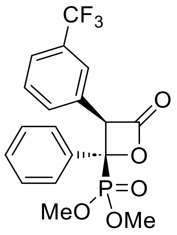

syn-**54**

^{13}C, $CDCl_3$, 101 MHz

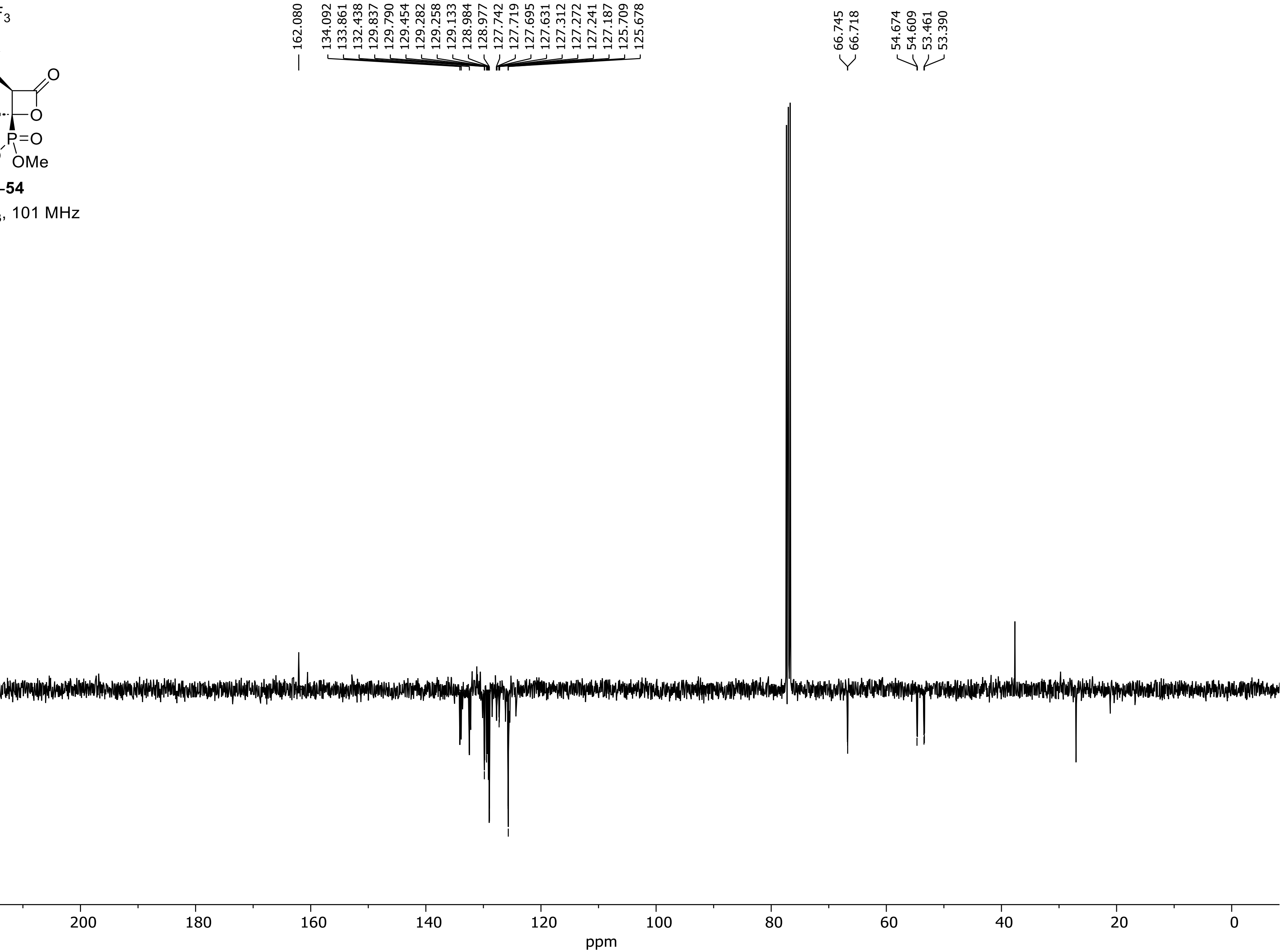

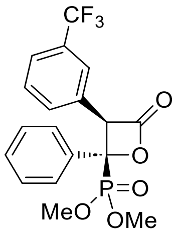

syn-**54**

^{31}P, $CDCl_3$, 162 MHz

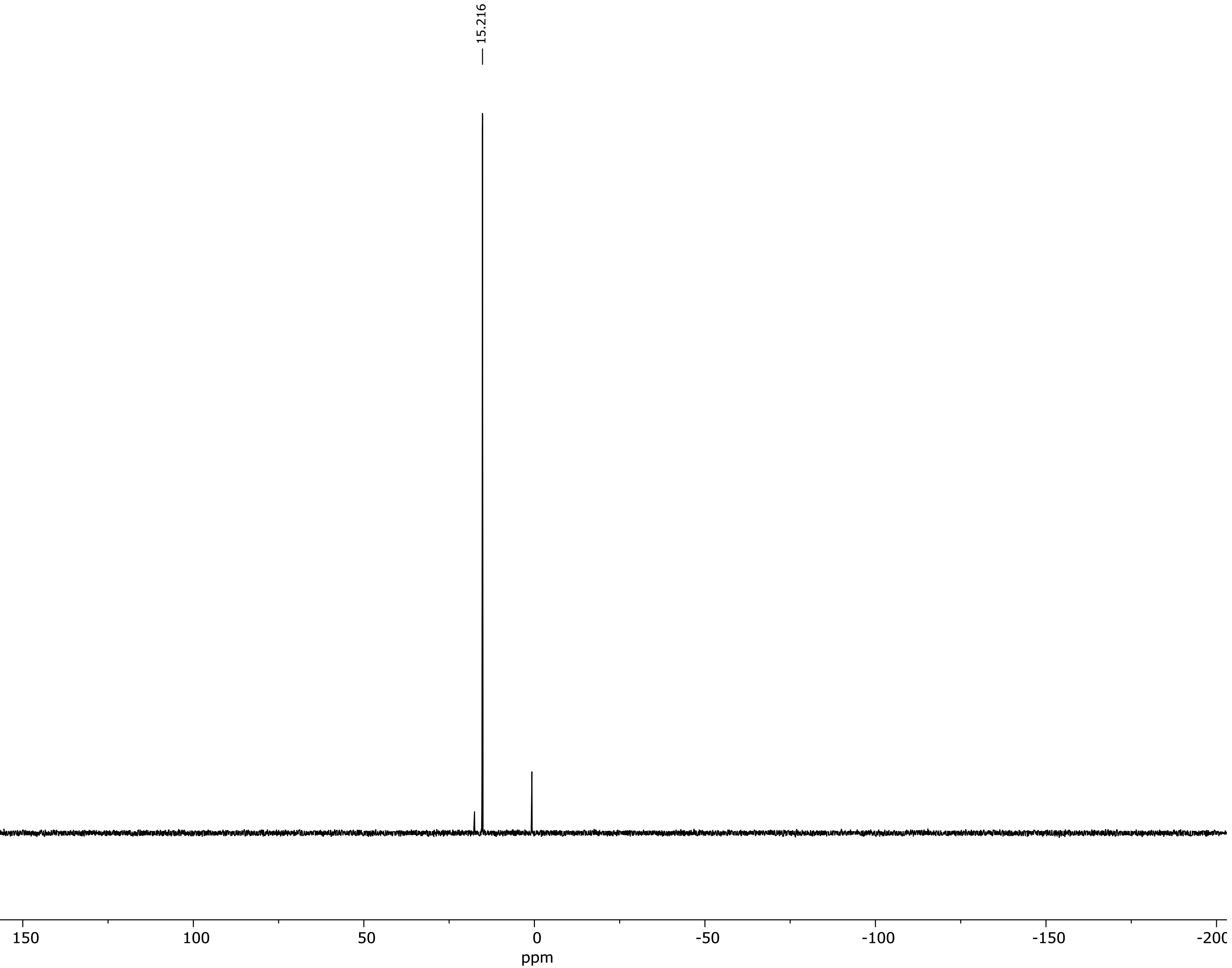

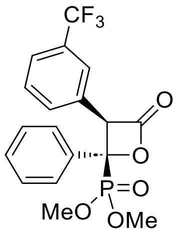

syn-**54**

^{19}F, $CDCl_3$, 376 MHz

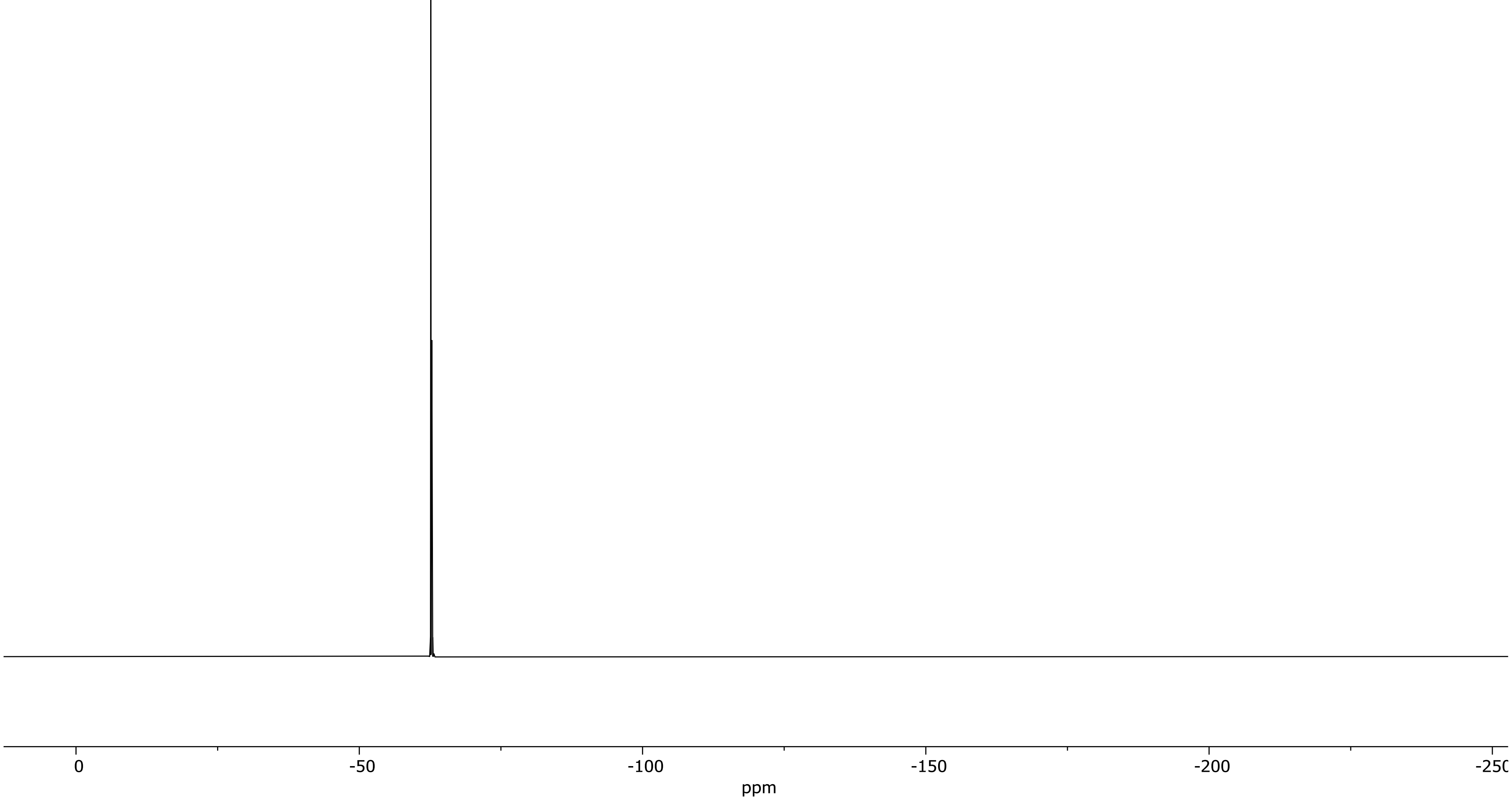

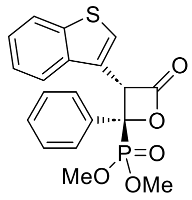

anti-**55**

^{1}H, $CDCl_3$, 400 MHz

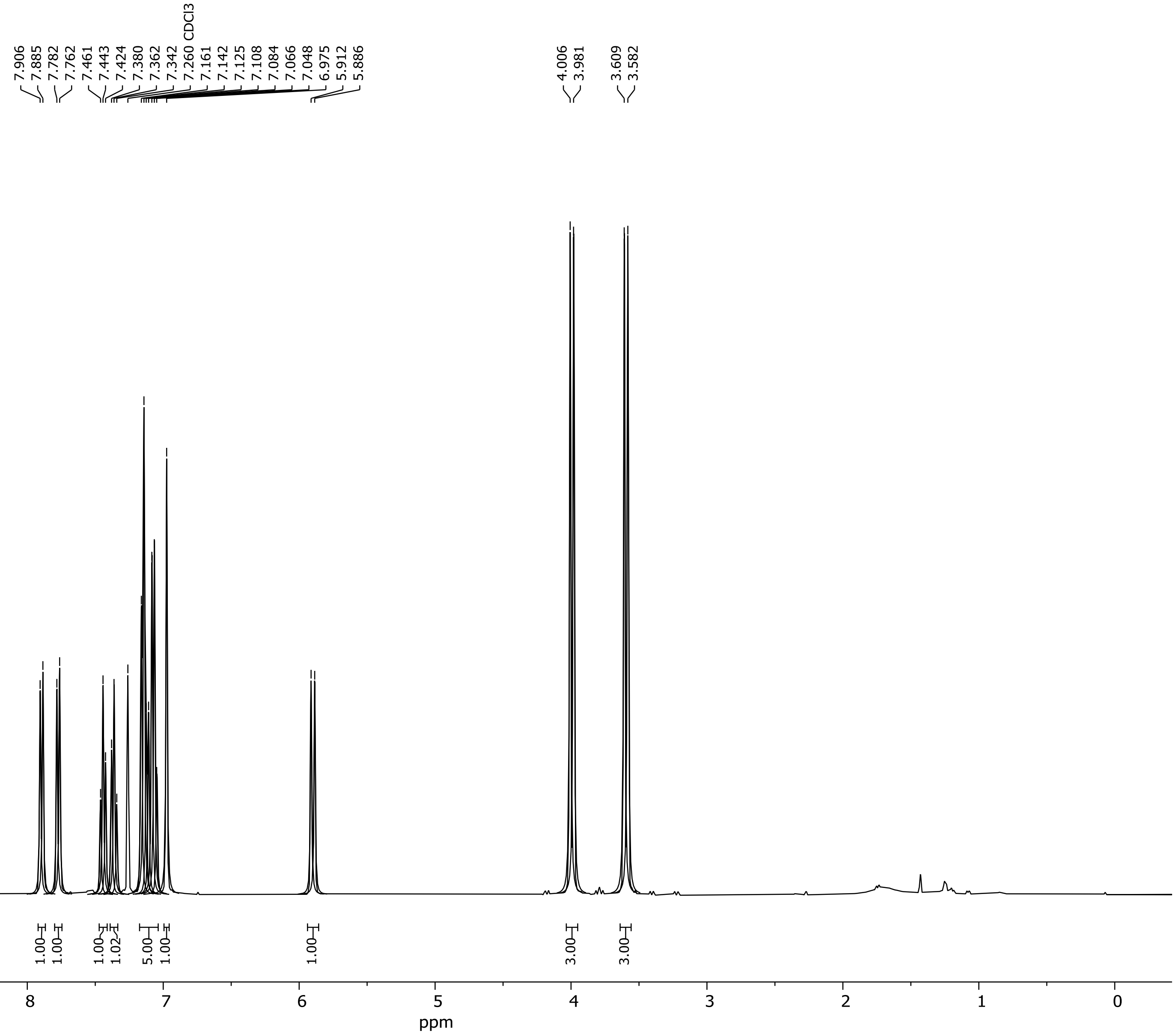

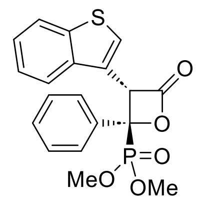

anti-**55**

^{13}C, $CDCl_3$, 101 MHz

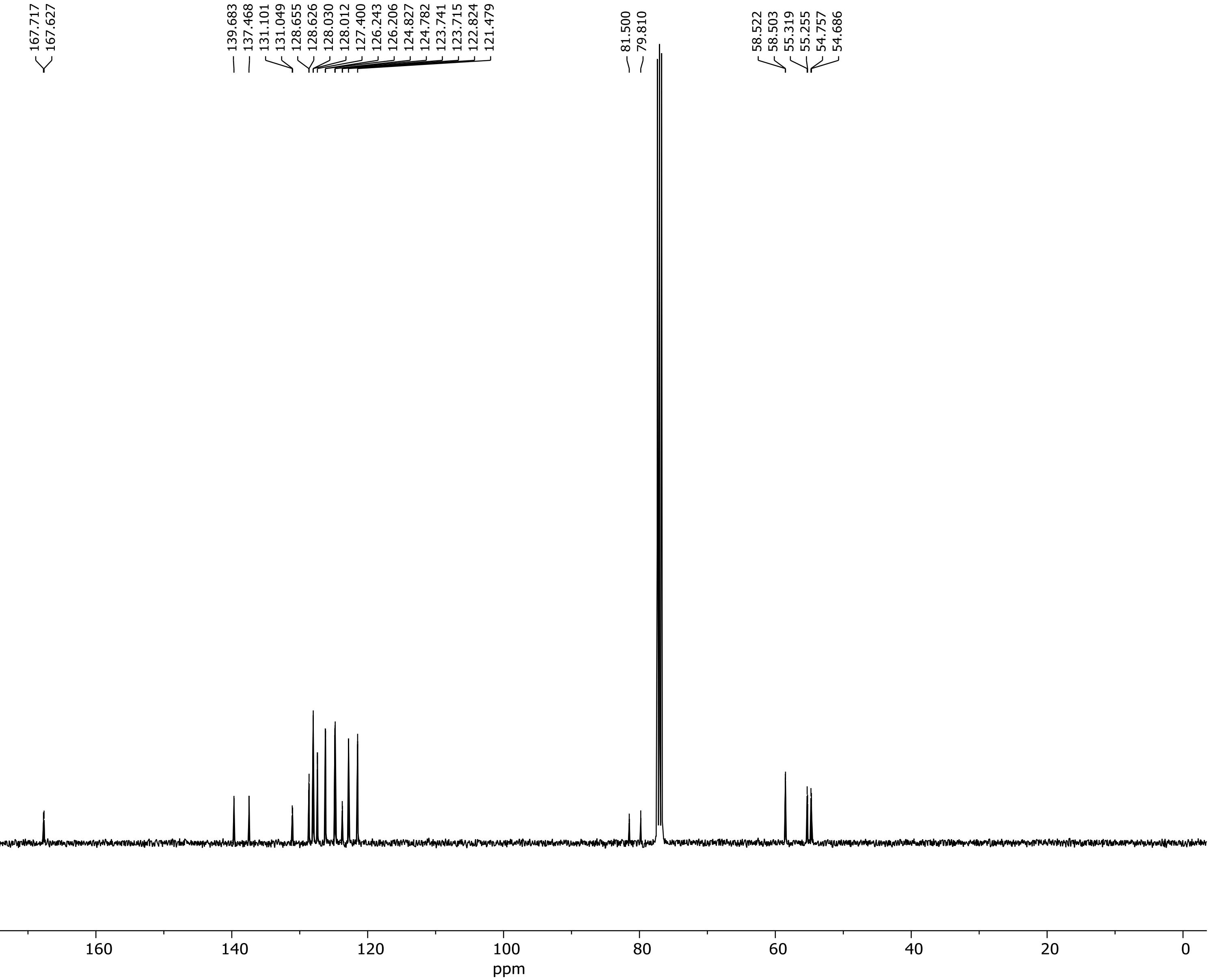

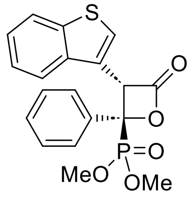

anti-**55**

^{31}P, $CDCl_3$, 162 MHz

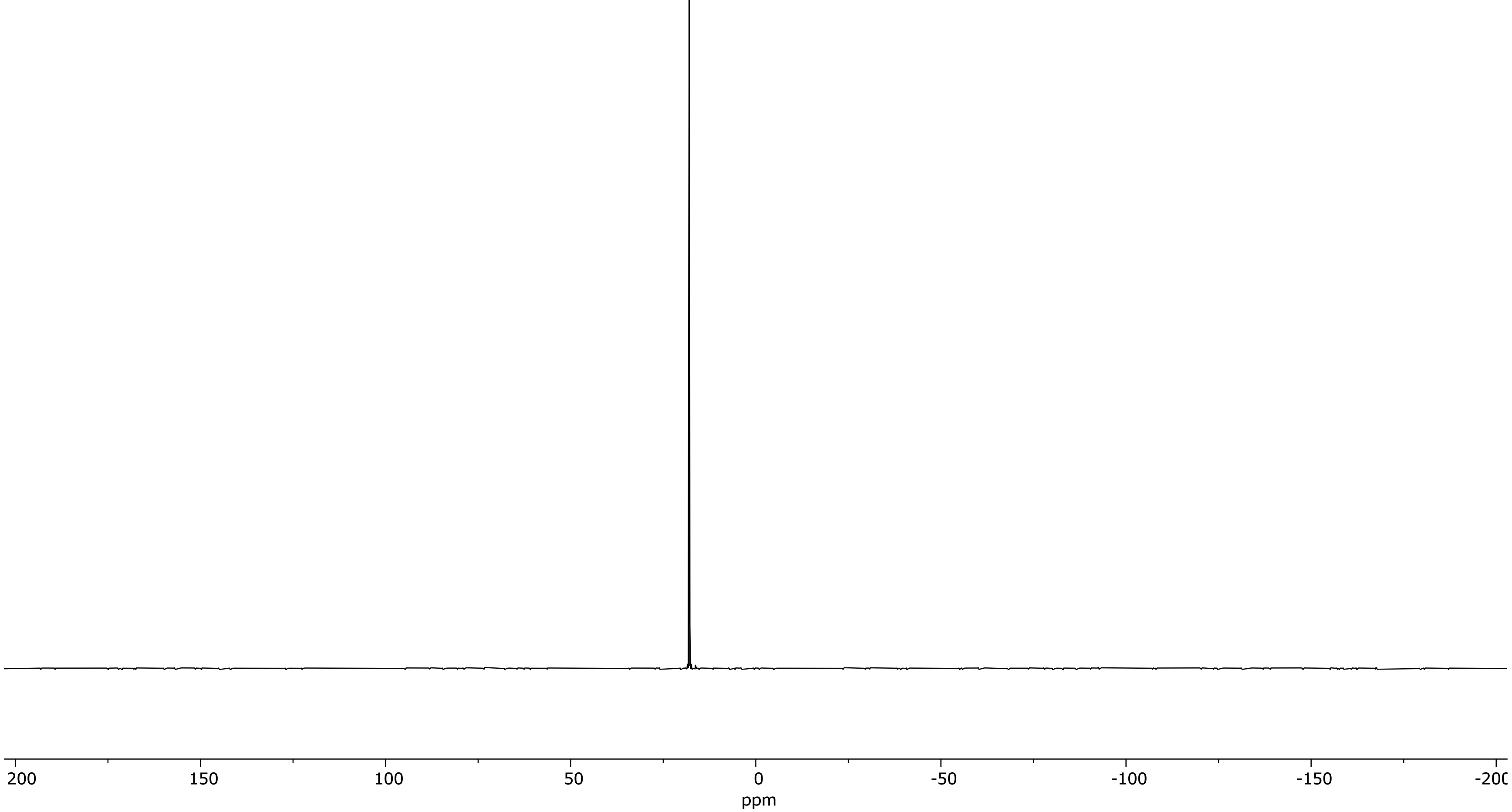

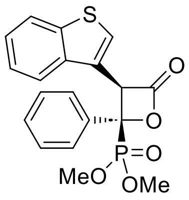

syn-**55**

^{1}H, $CDCl_3$, 400 MHz

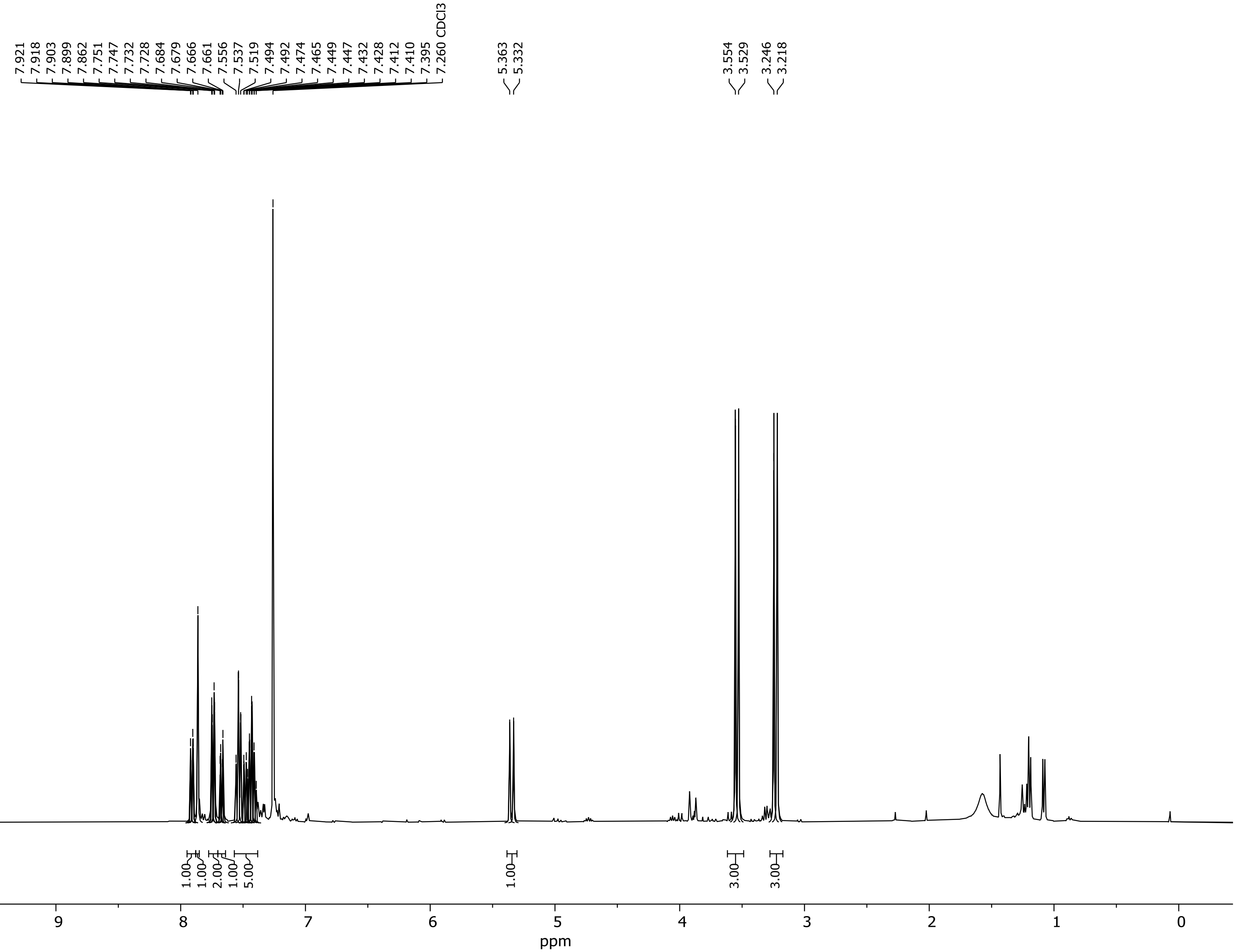

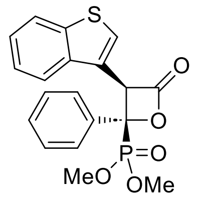

syn-**55**

^{13}C, $CDCl_3$, 101 MHz

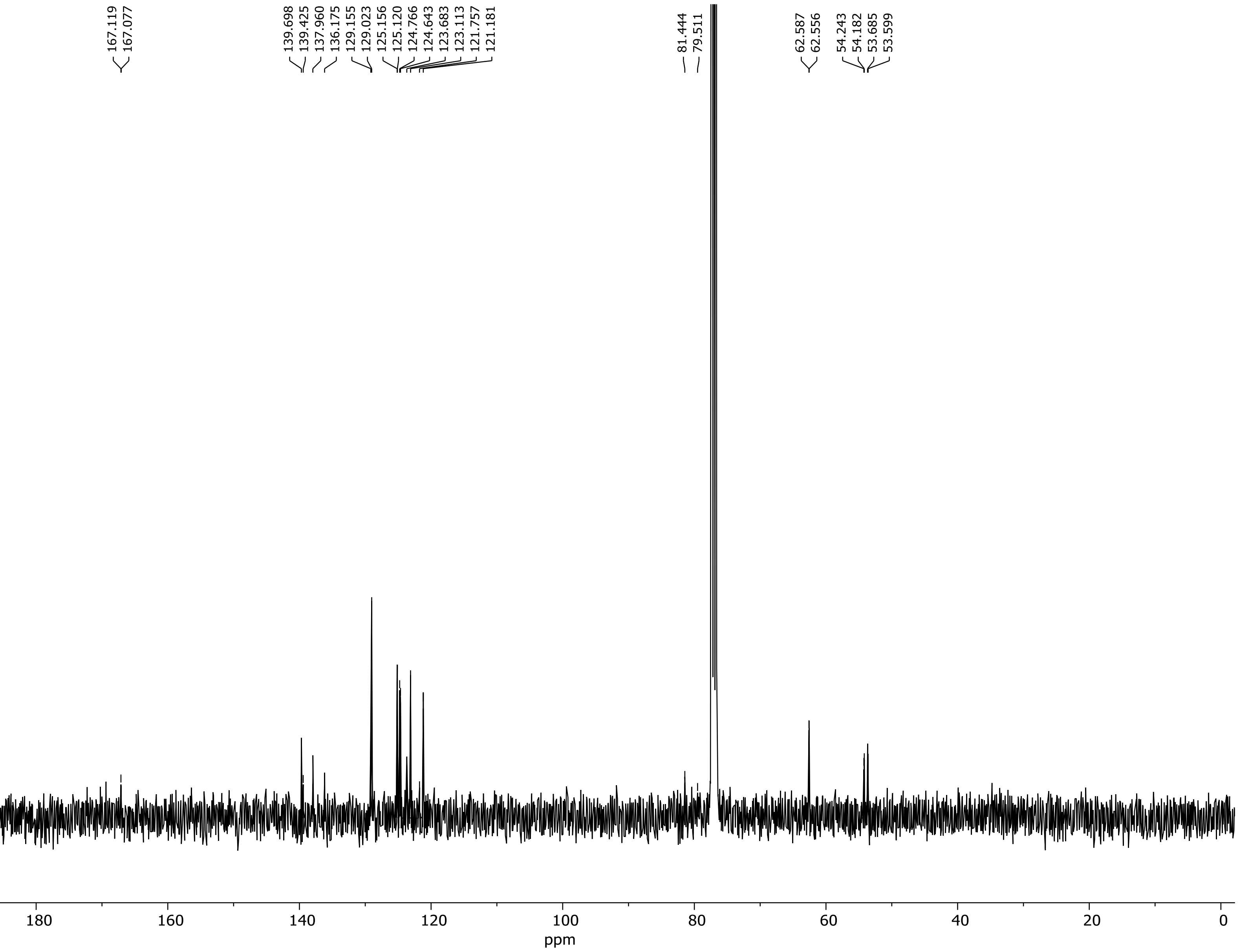

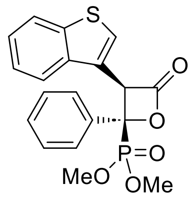

syn-**55**

^{31}P, $CDCl_3$, 162 MHz

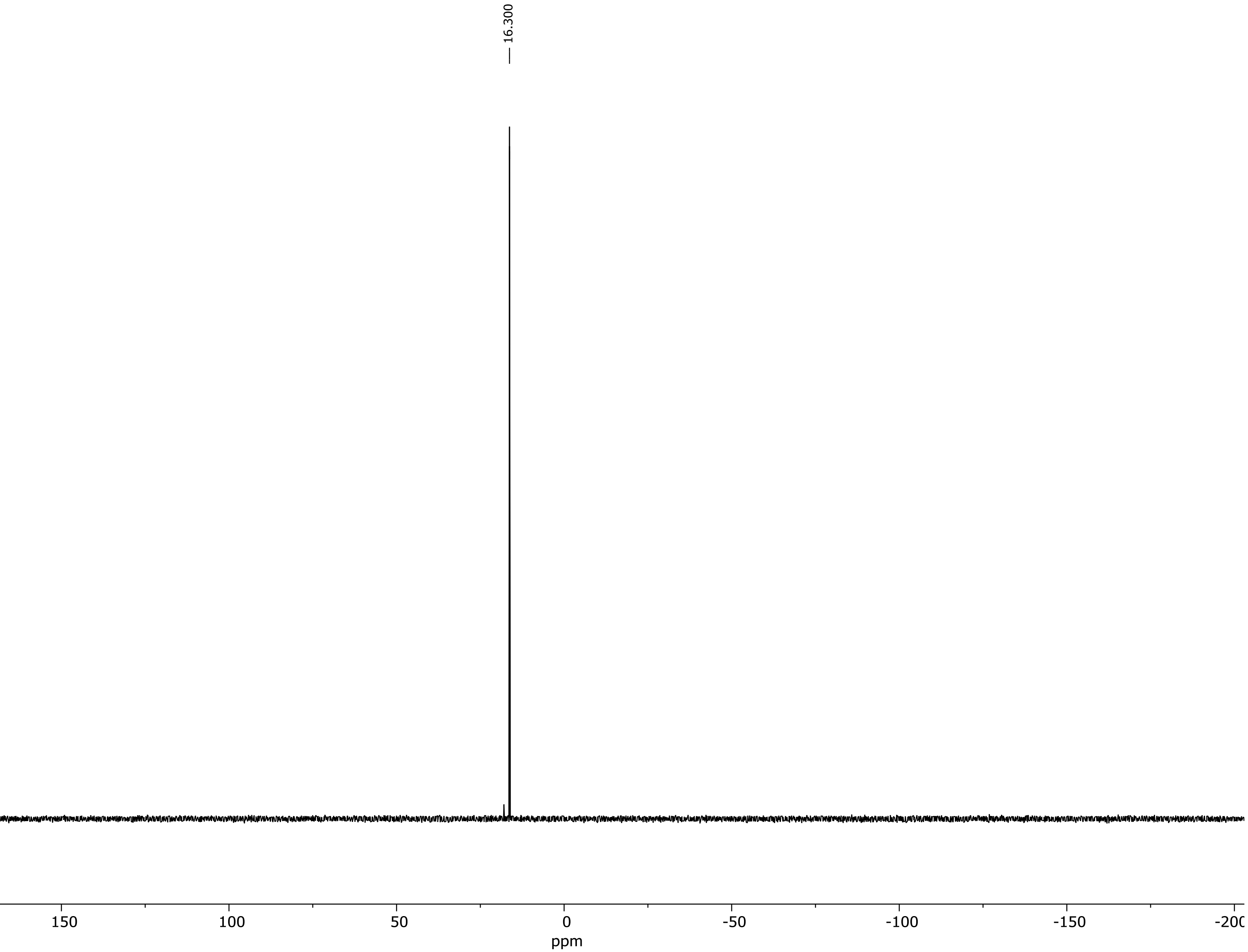

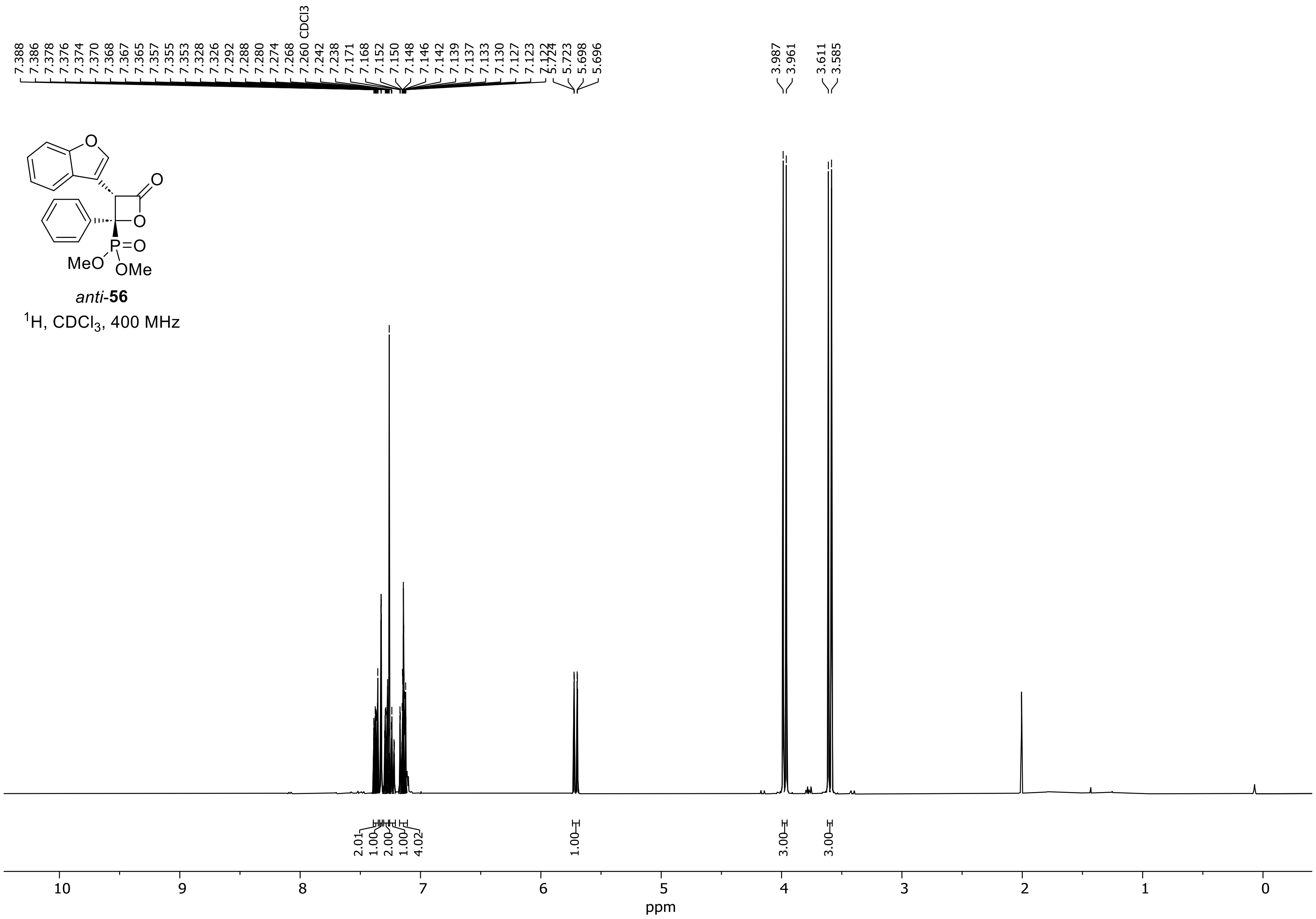

anti-**56**

^{1}H, $CDCl_3$, 400 MHz

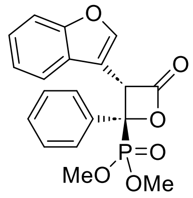

anti-**56**

^{13}C, $CDCl_3$, 101 MHz

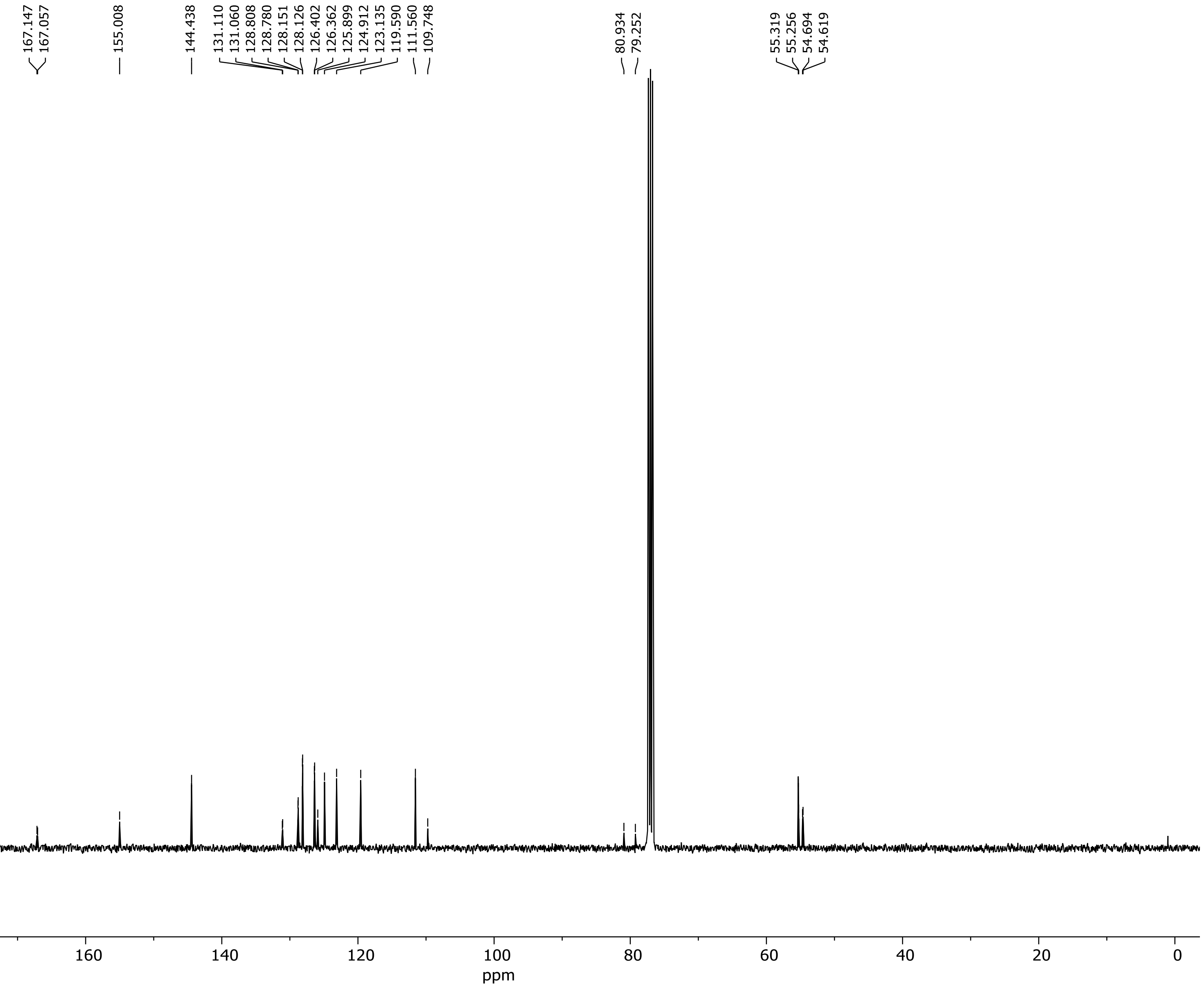

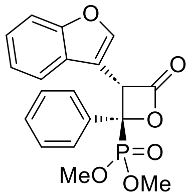

anti-**56**

^{31}P, $CDCl_3$, 162 MHz

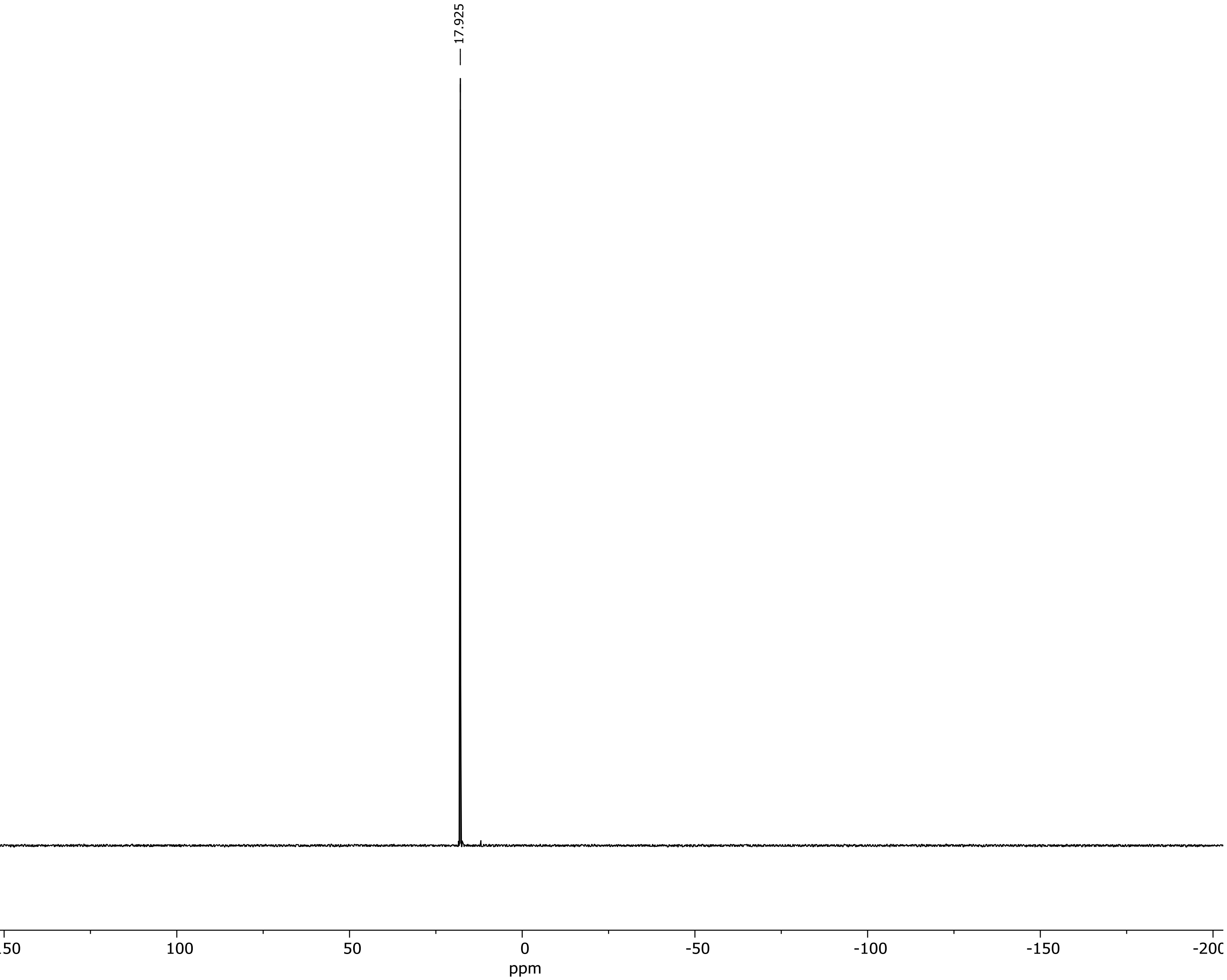

syn-**56**

^{1}H, $CDCl_3$, 400 MHz

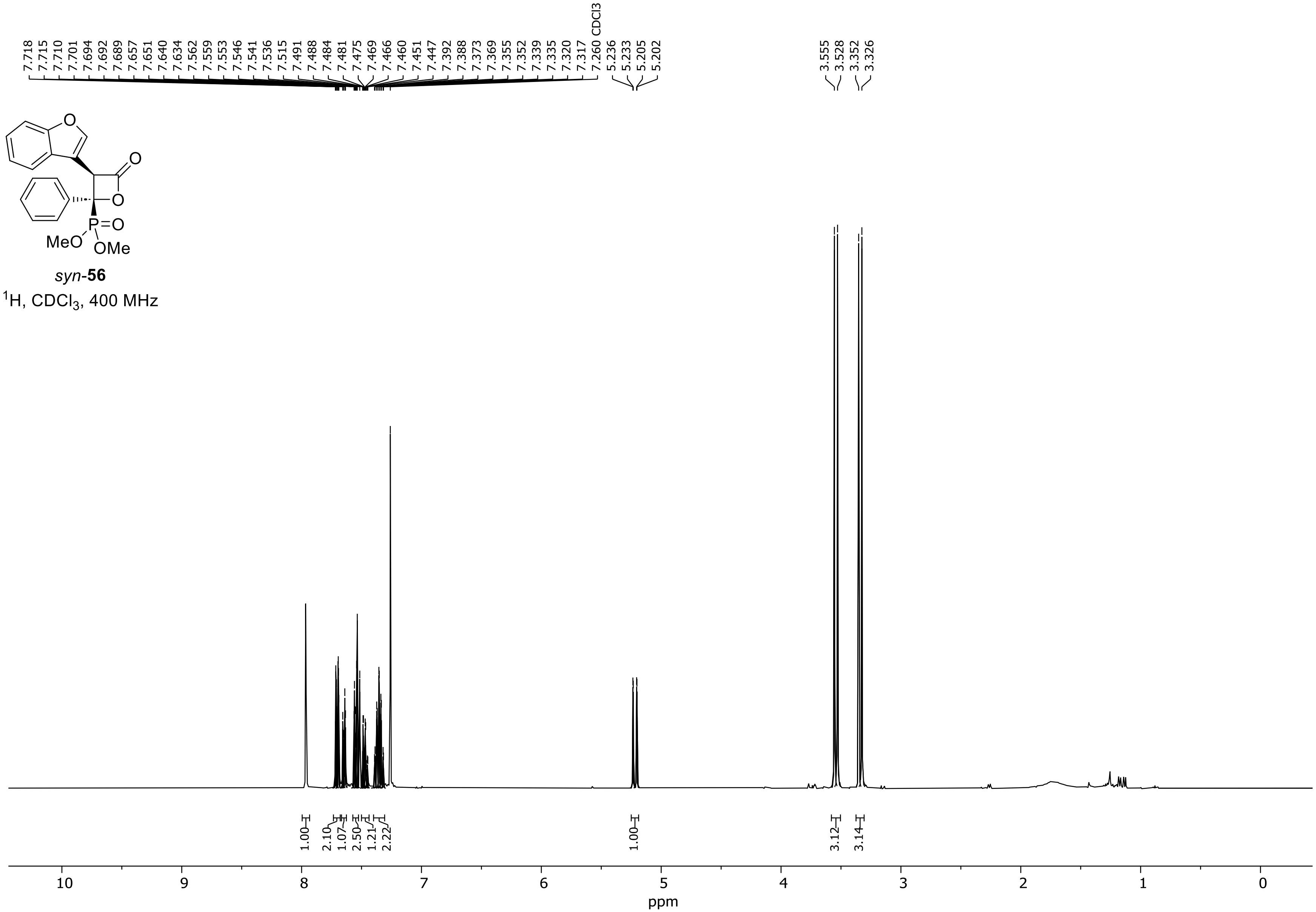

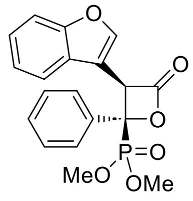

syn-**56**

^{13}C, $CDCl_3$, 101 MHz

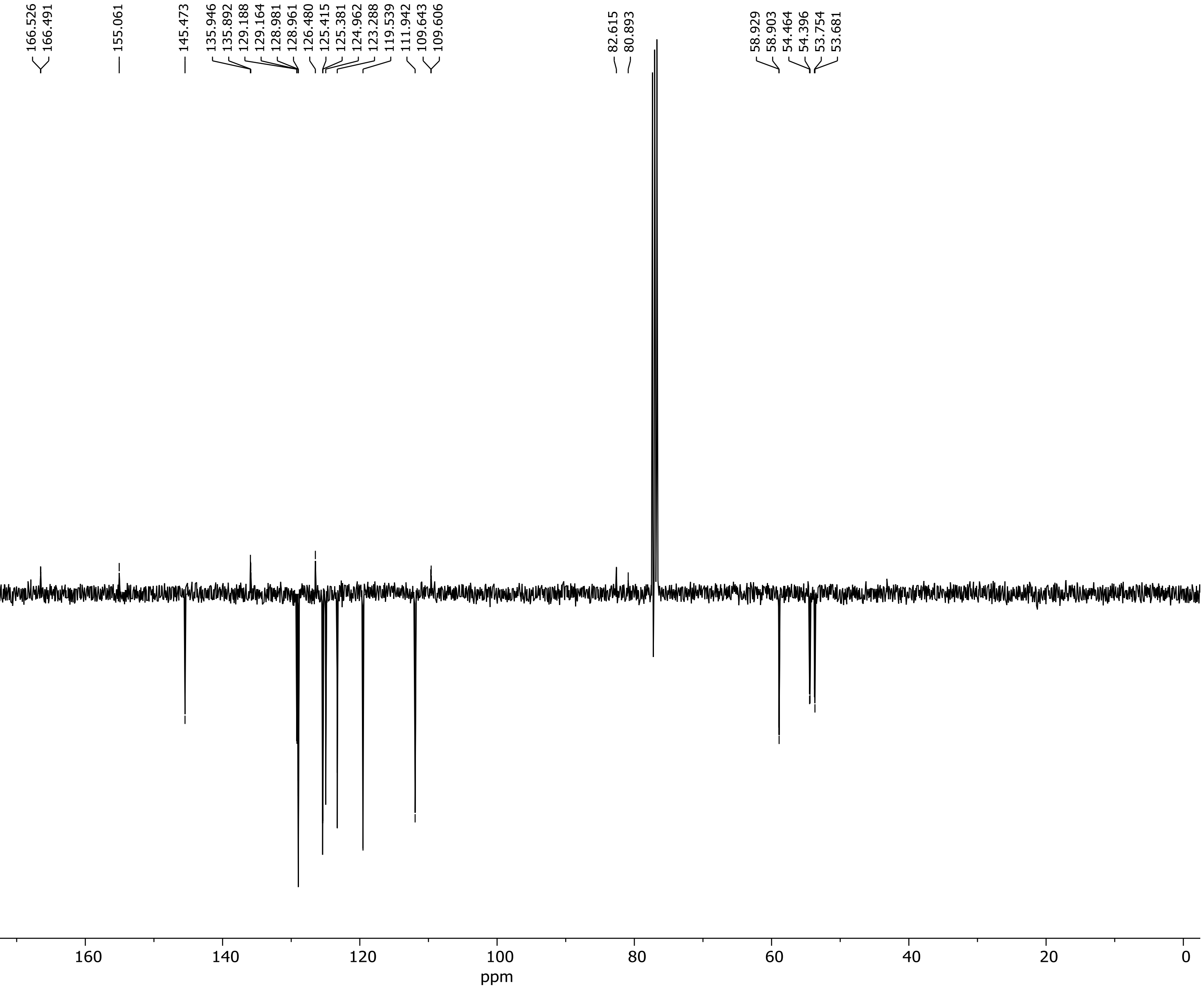

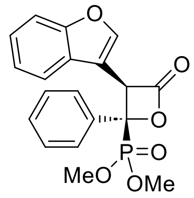

syn-**56**

^{31}P, $CDCl_3$, 162 MHz

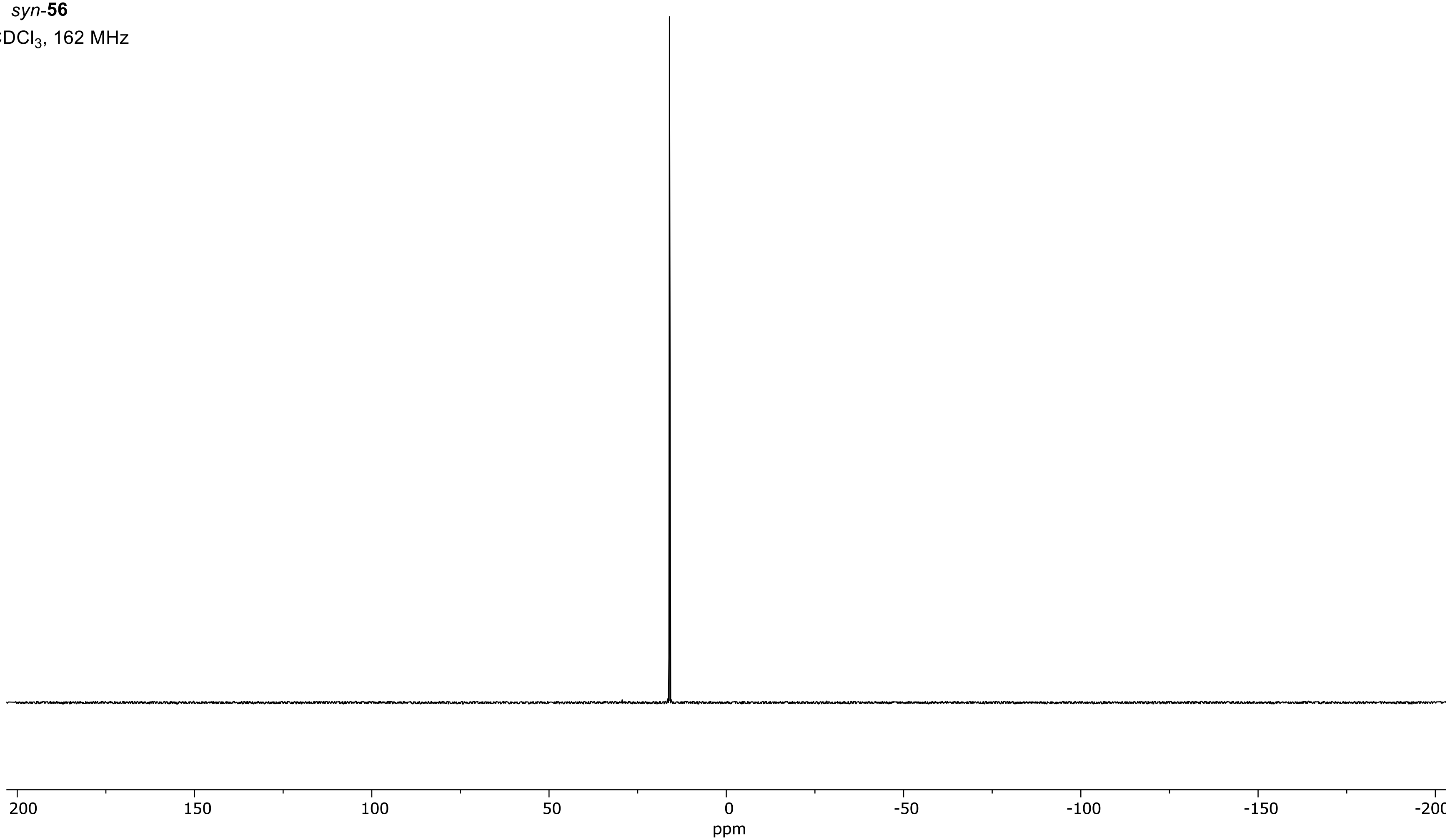

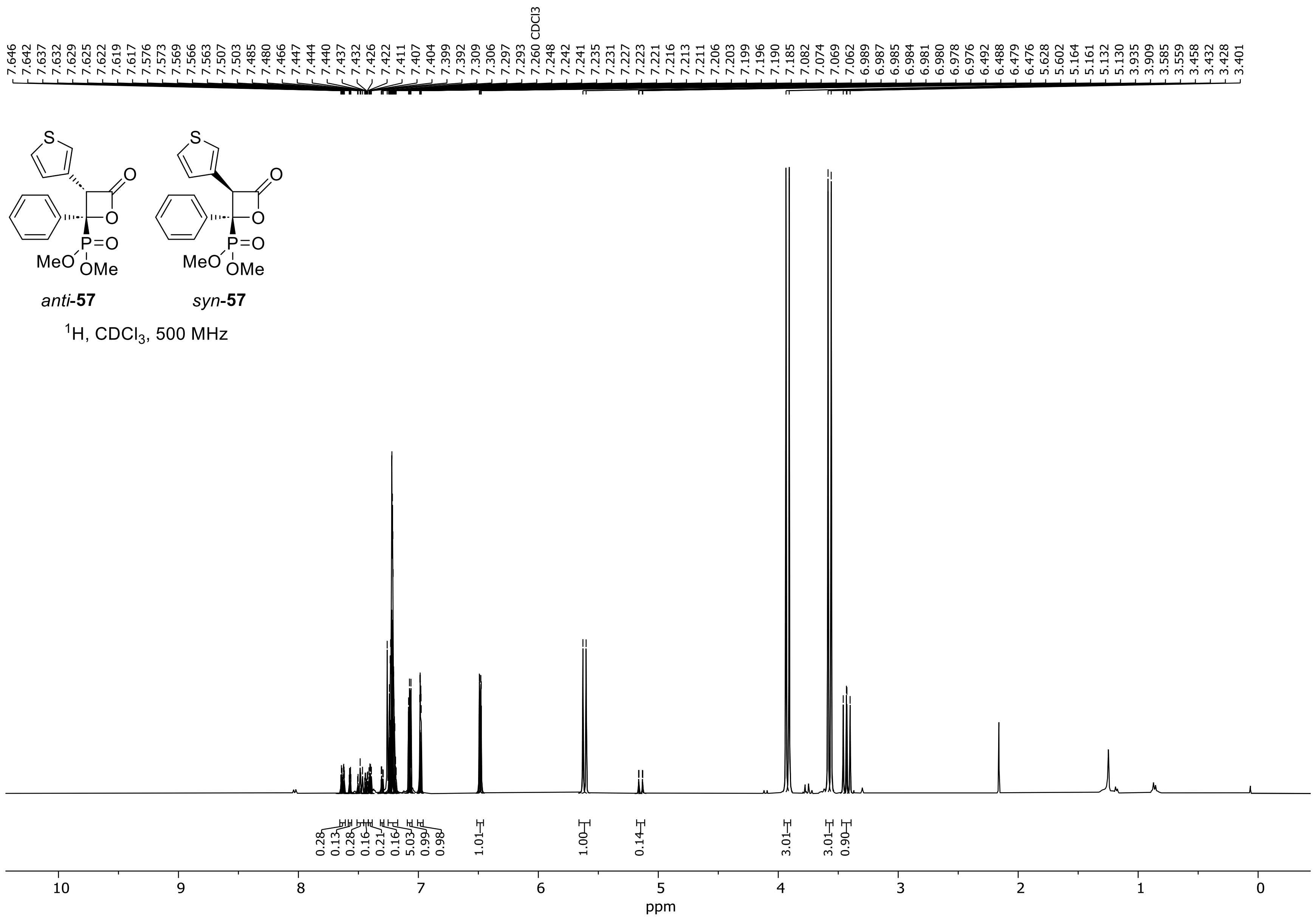
anti-57
syn-57
1H, CDCl3, 500 MHz
ppm

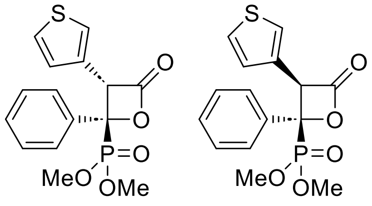

anti-**57** *syn*-**57**

^{13}C, $CDCl_3$, 101 MHz

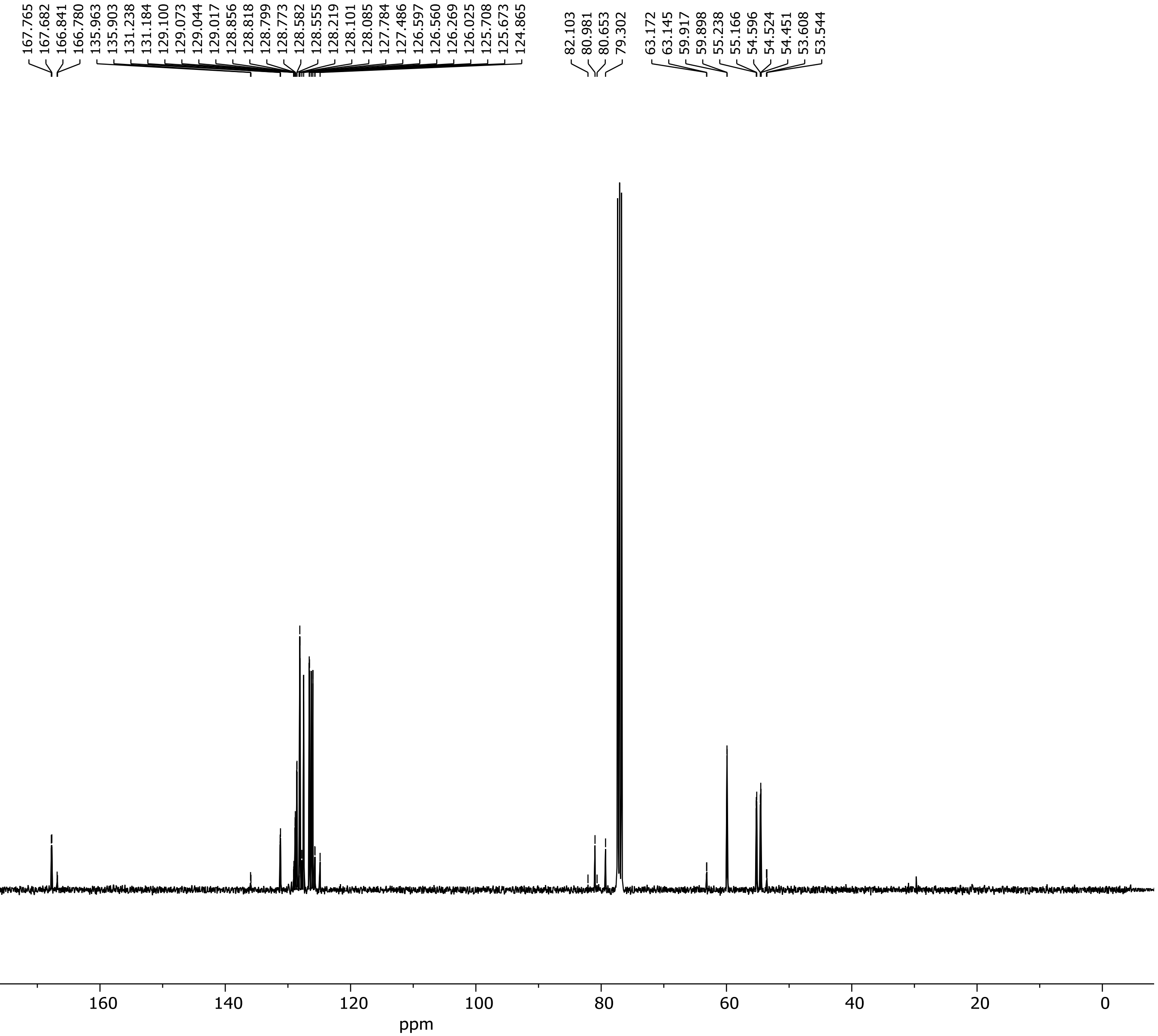

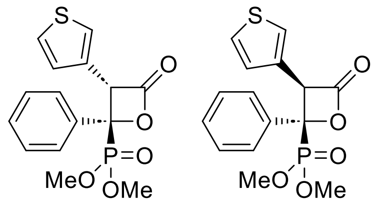

^{31}P, $CDCl_3$, 162 MHz

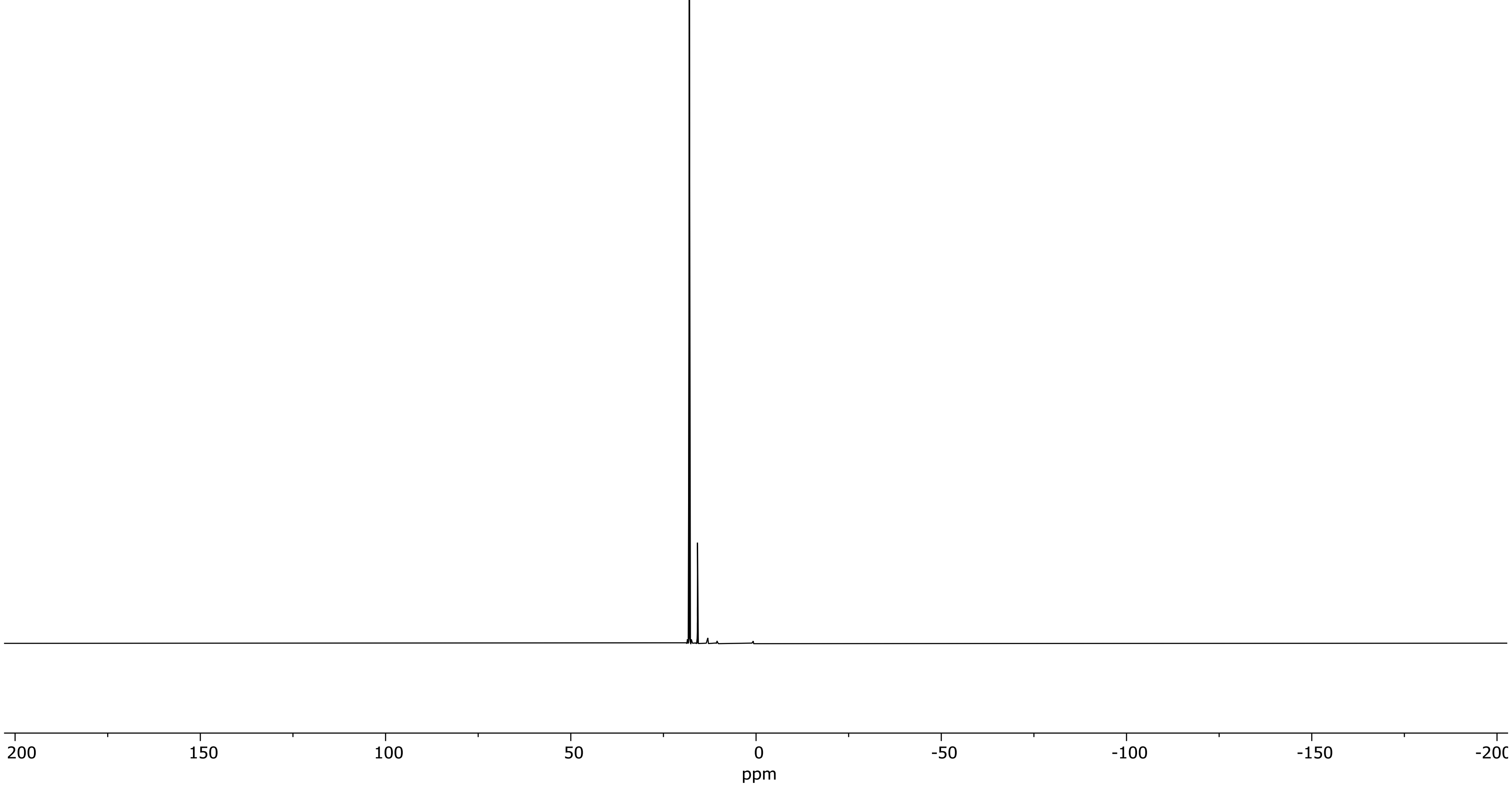

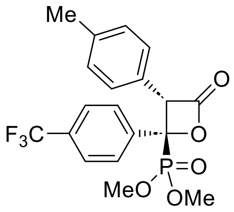

anti-**58**

^{1}H, $CDCl_3$, 400 MHz

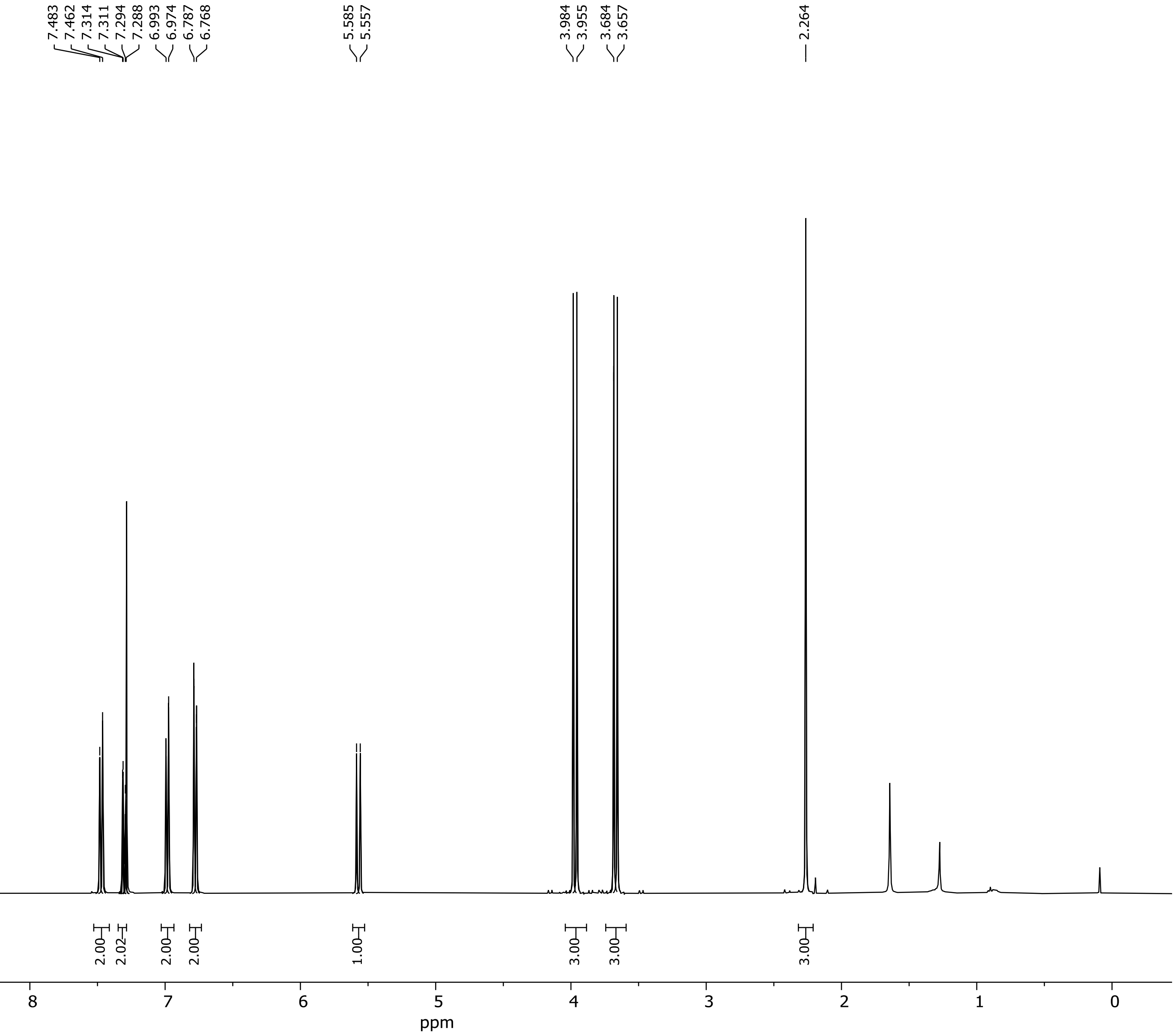

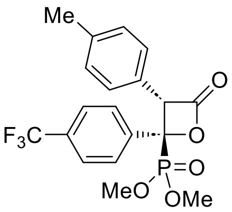

anti-**58**

^{13}C, $CDCl_3$, 101 MHz

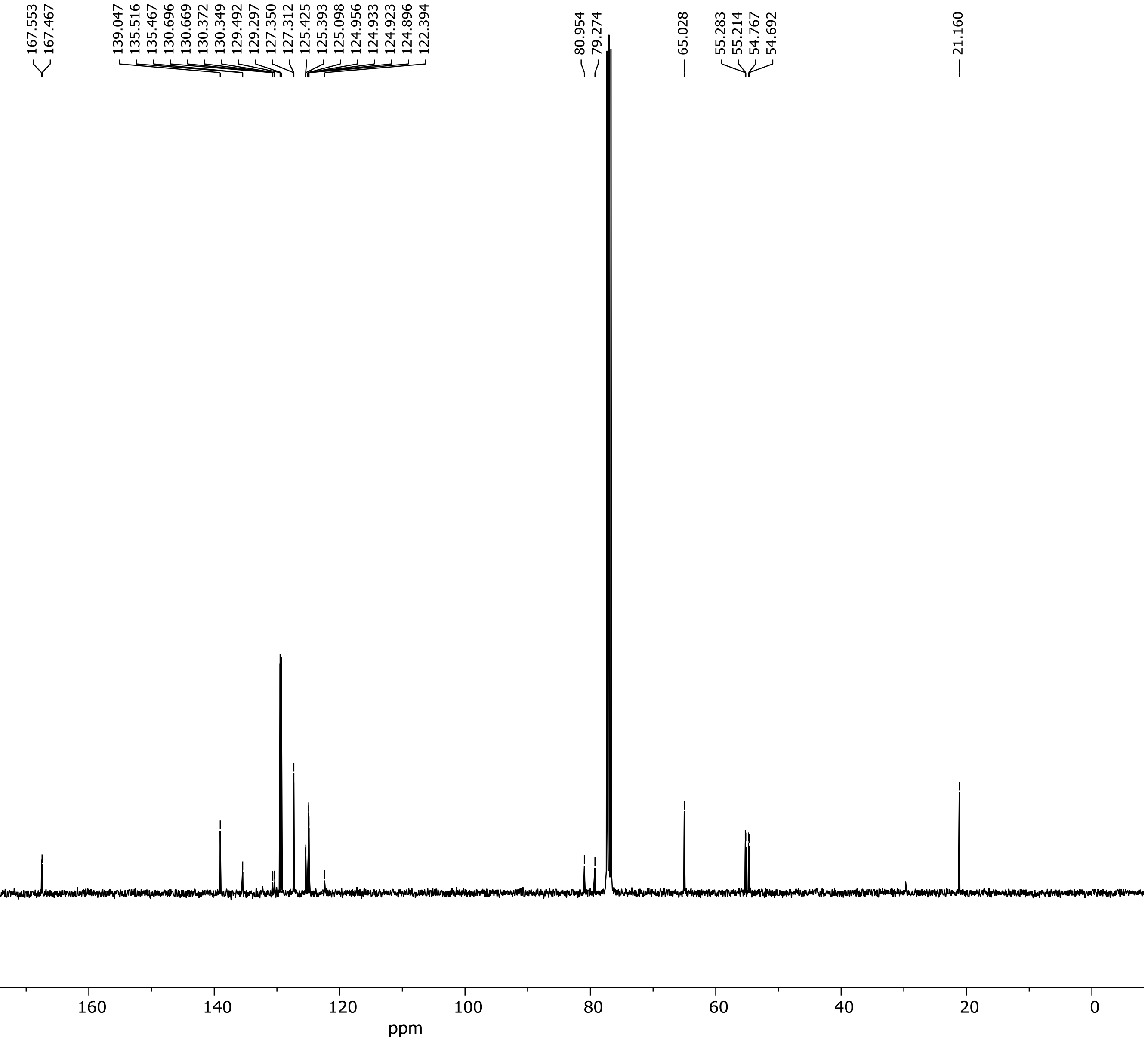

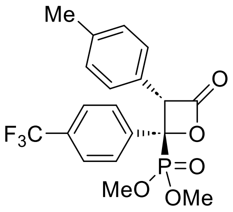

anti-**58**

^{31}P, $CDCl_3$, 162 MHz

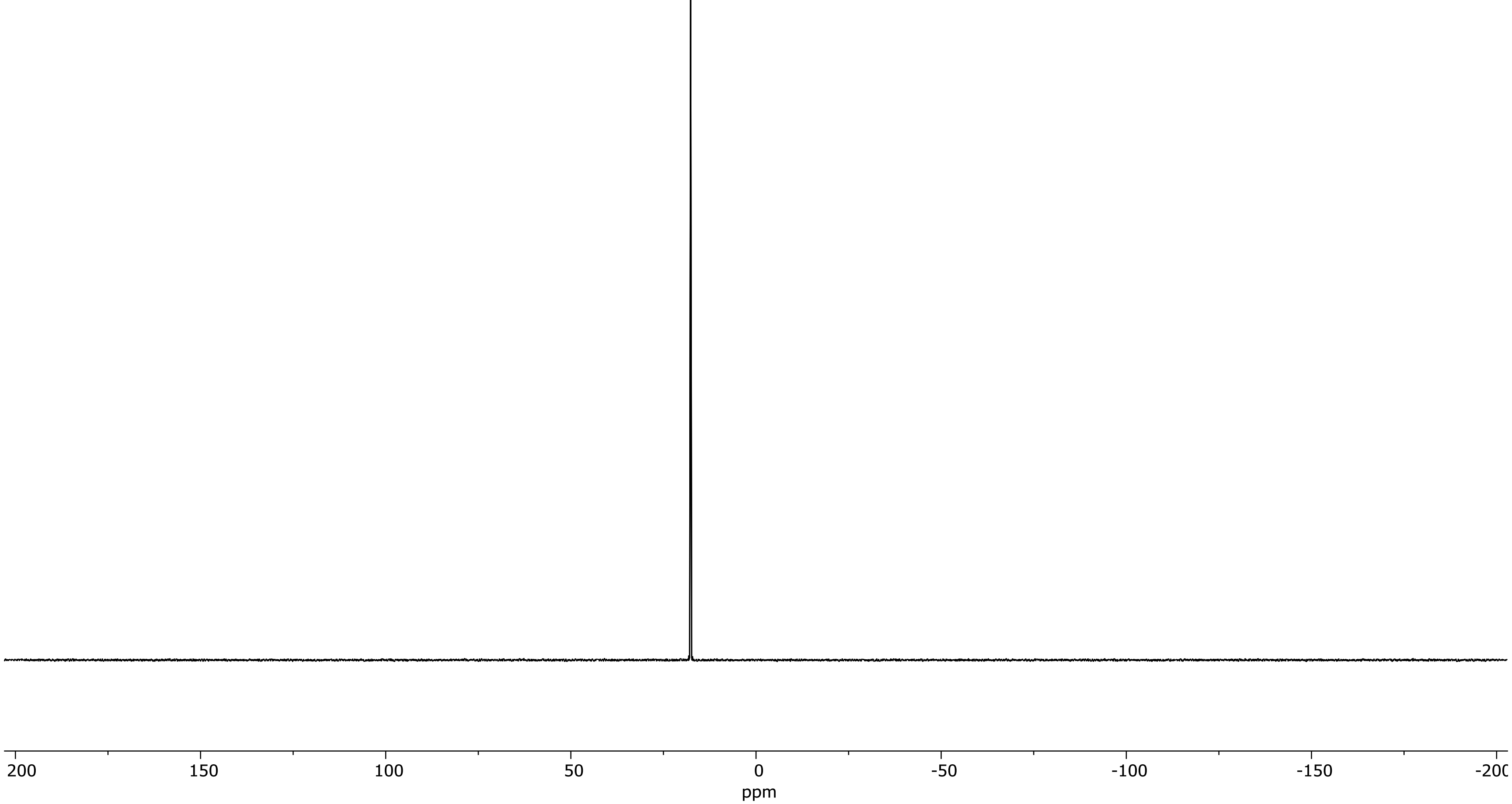

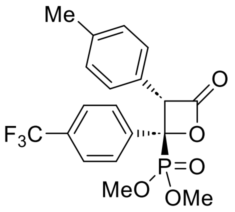

anti-**58**

^{19}F, $CDCl_3$, 376 MHz

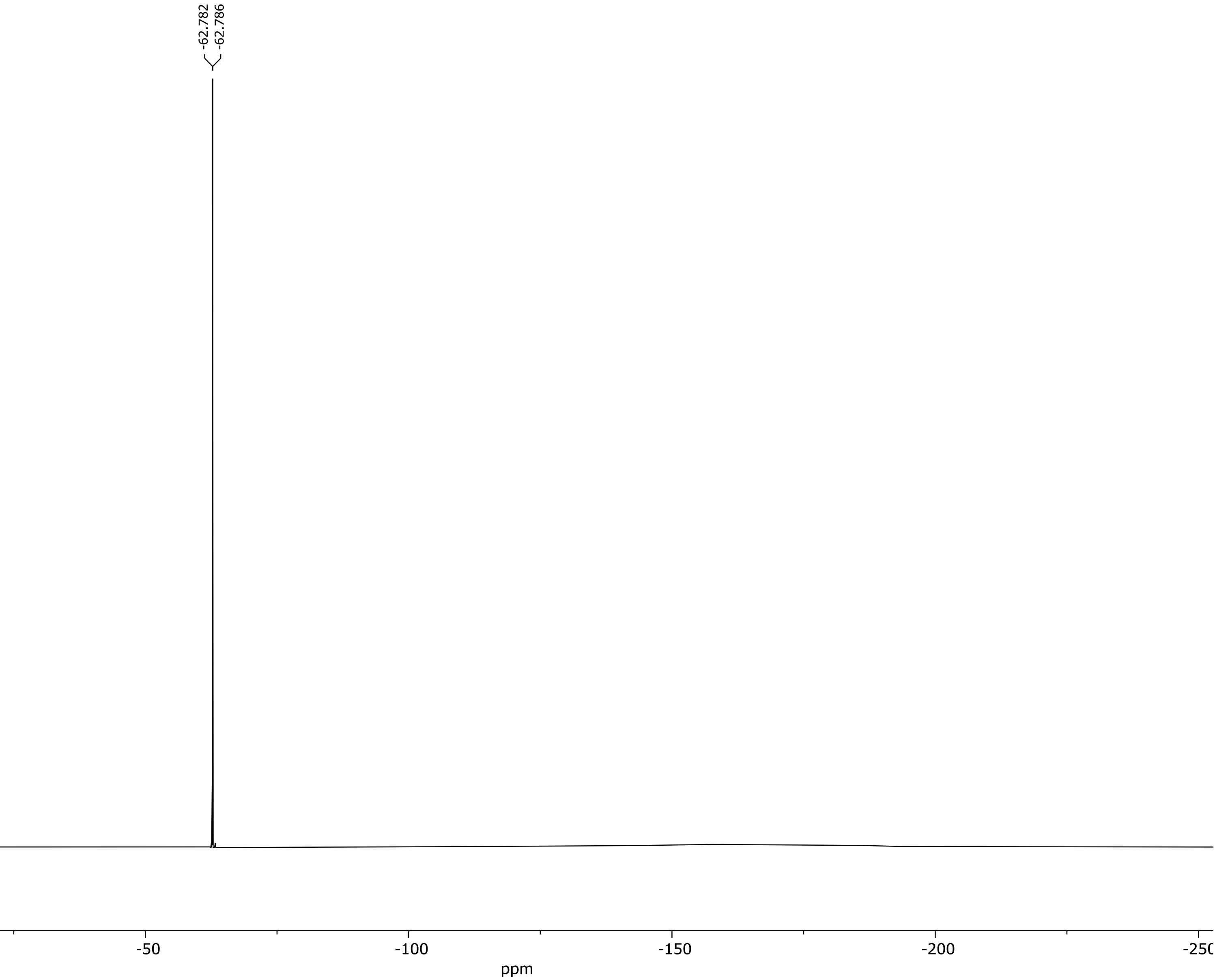

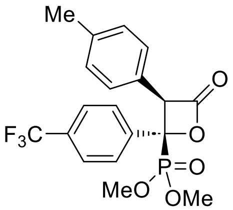

syn-**58**

^1H, $CDCl_3$, 400 MHz

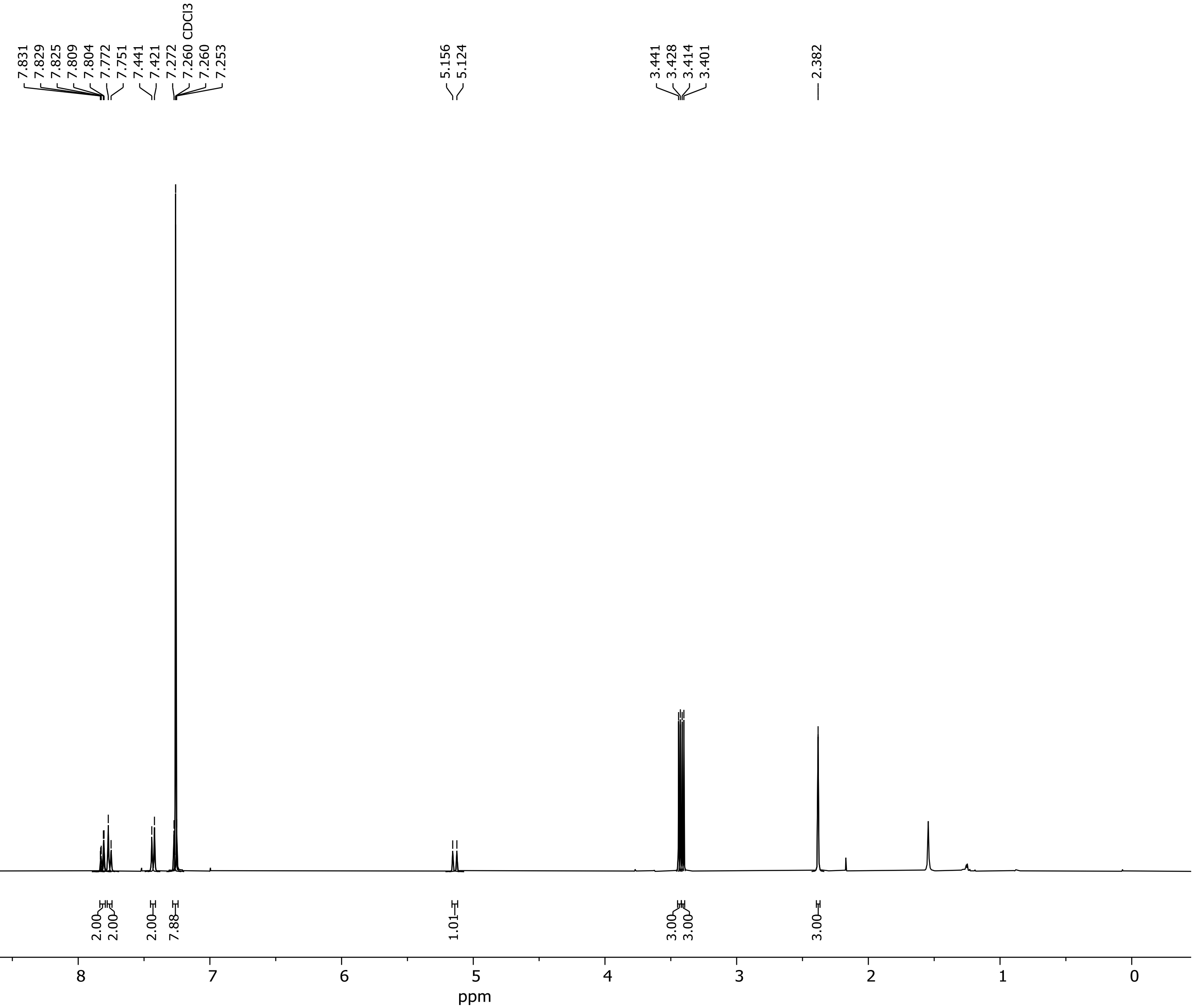

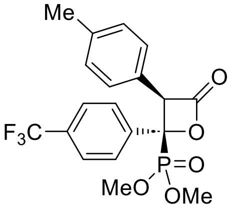

syn-**58**

^{13}C, $CDCl_3$, 101 MHz

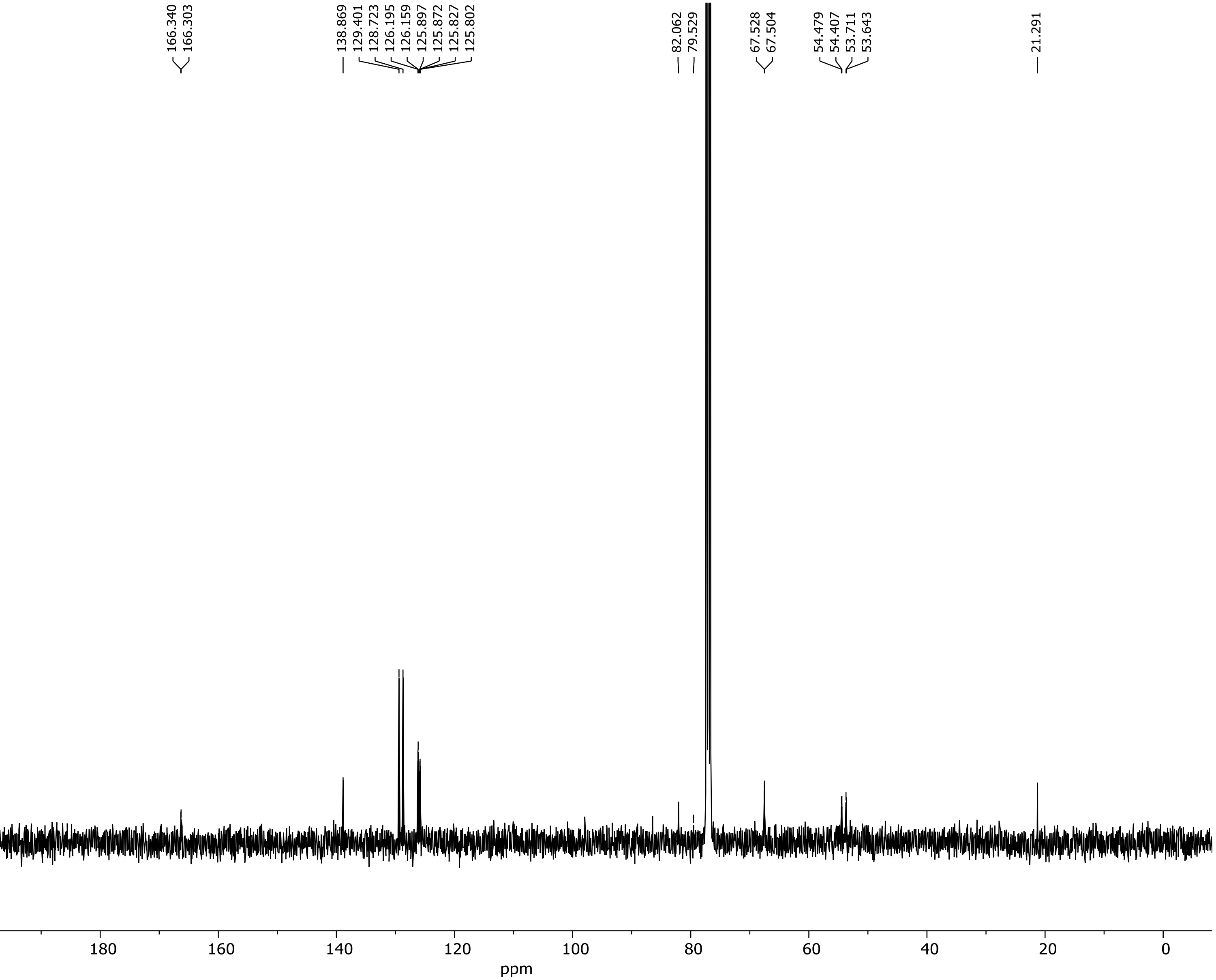

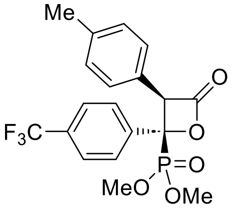

syn-**58**

^{31}P, $CDCl_3$, 162 MHz

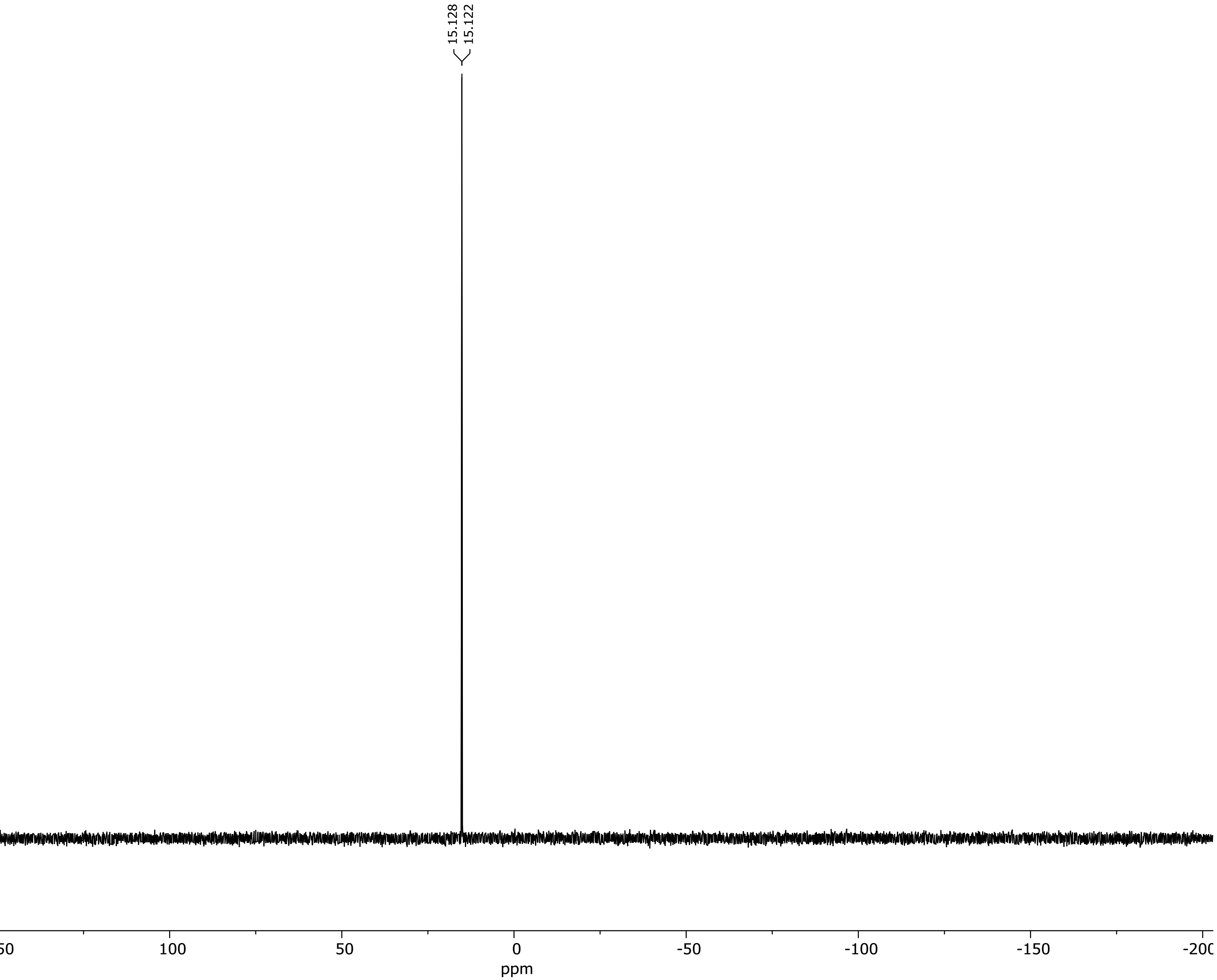

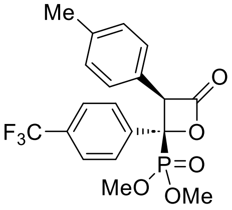

syn-**58**

^{19}F, $CDCl_3$, 376 MHz

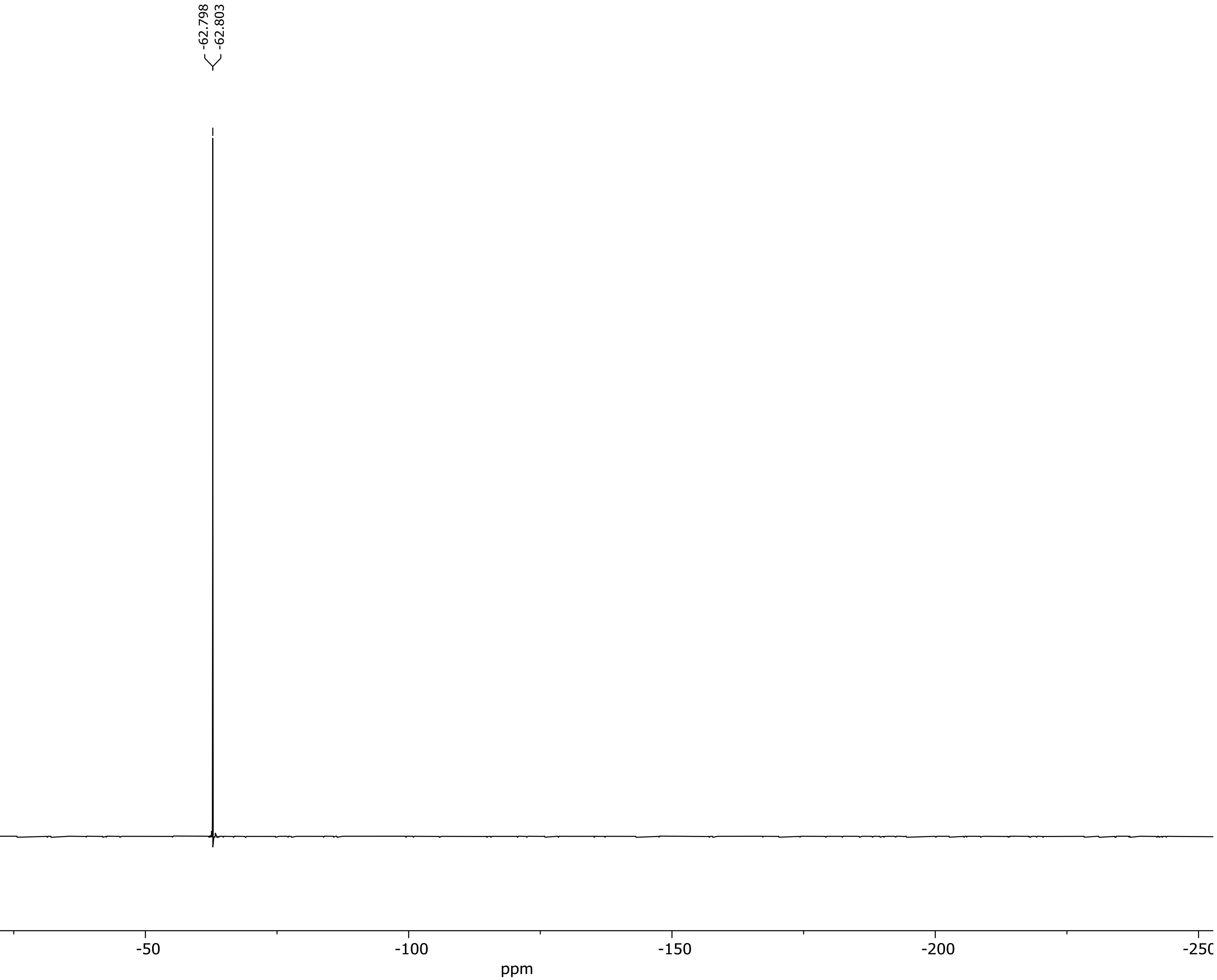

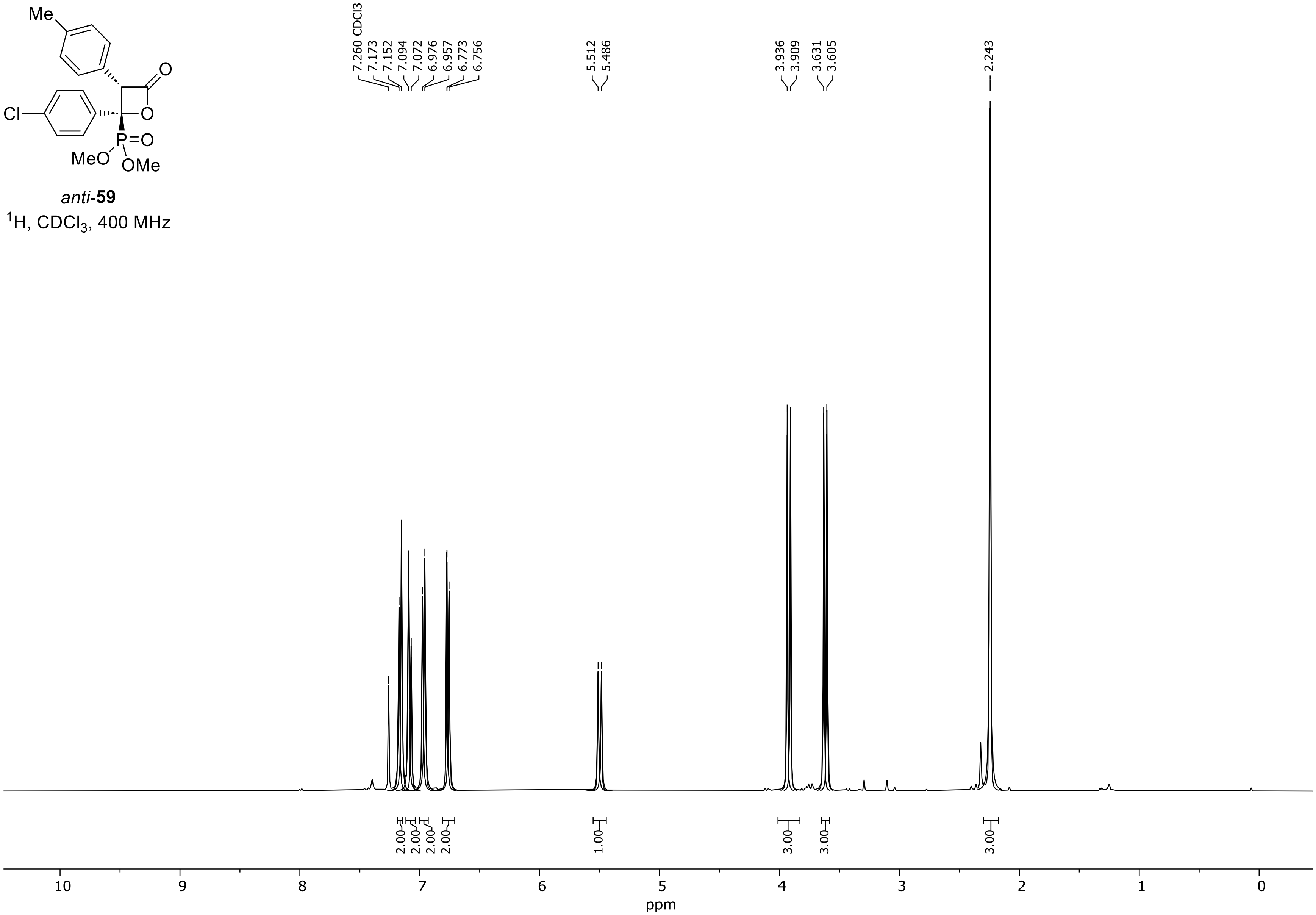

anti-**59**

^{1}H, $CDCl_3$, 400 MHz

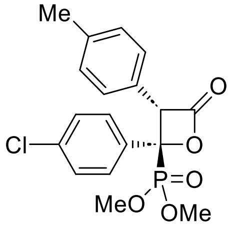

anti-**59**

^{13}C, $CDCl_3$, 101 MHz

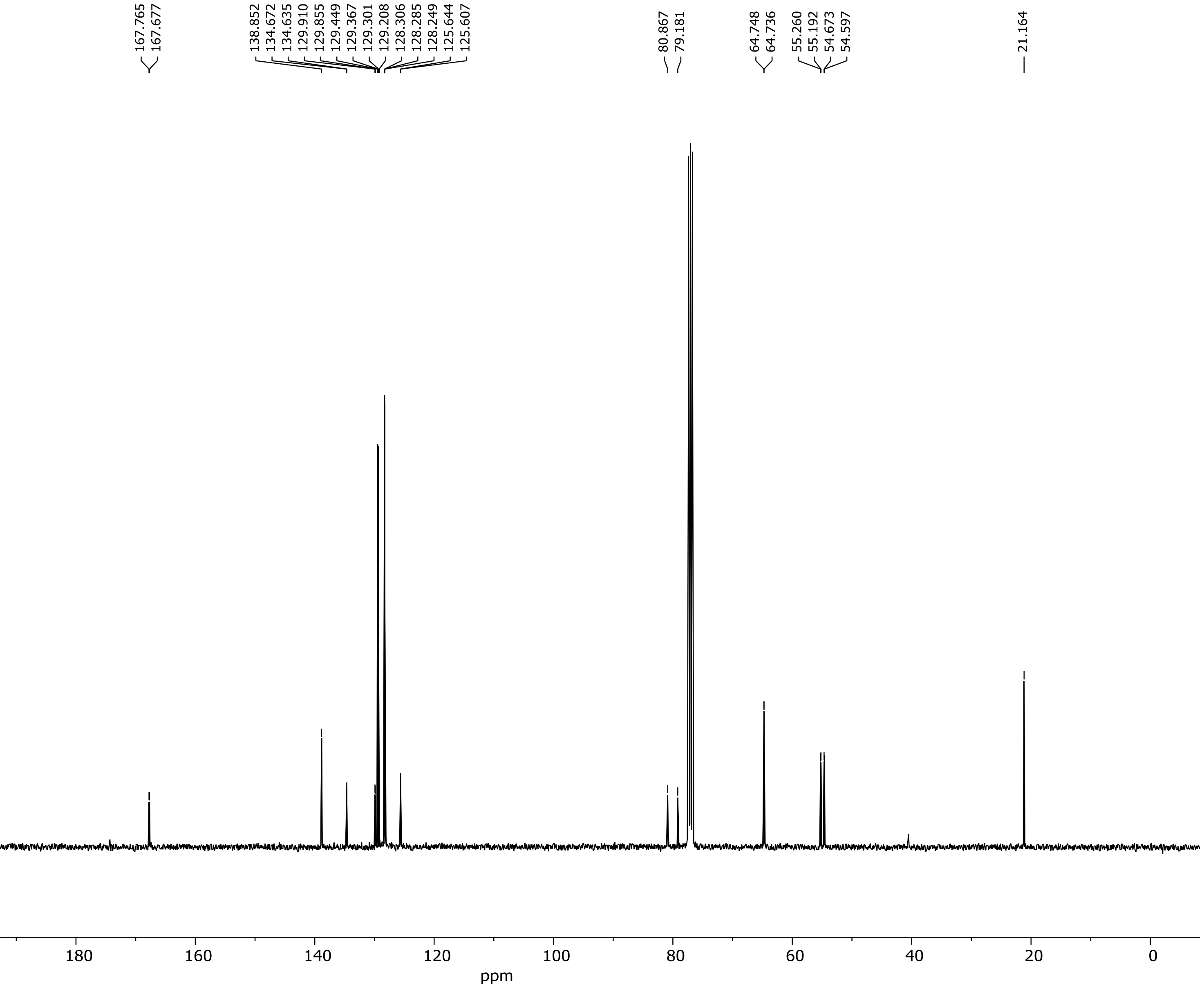

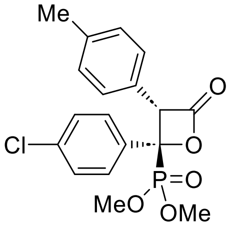

anti-**59**

^{31}P, $CDCl_3$, 162 MHz

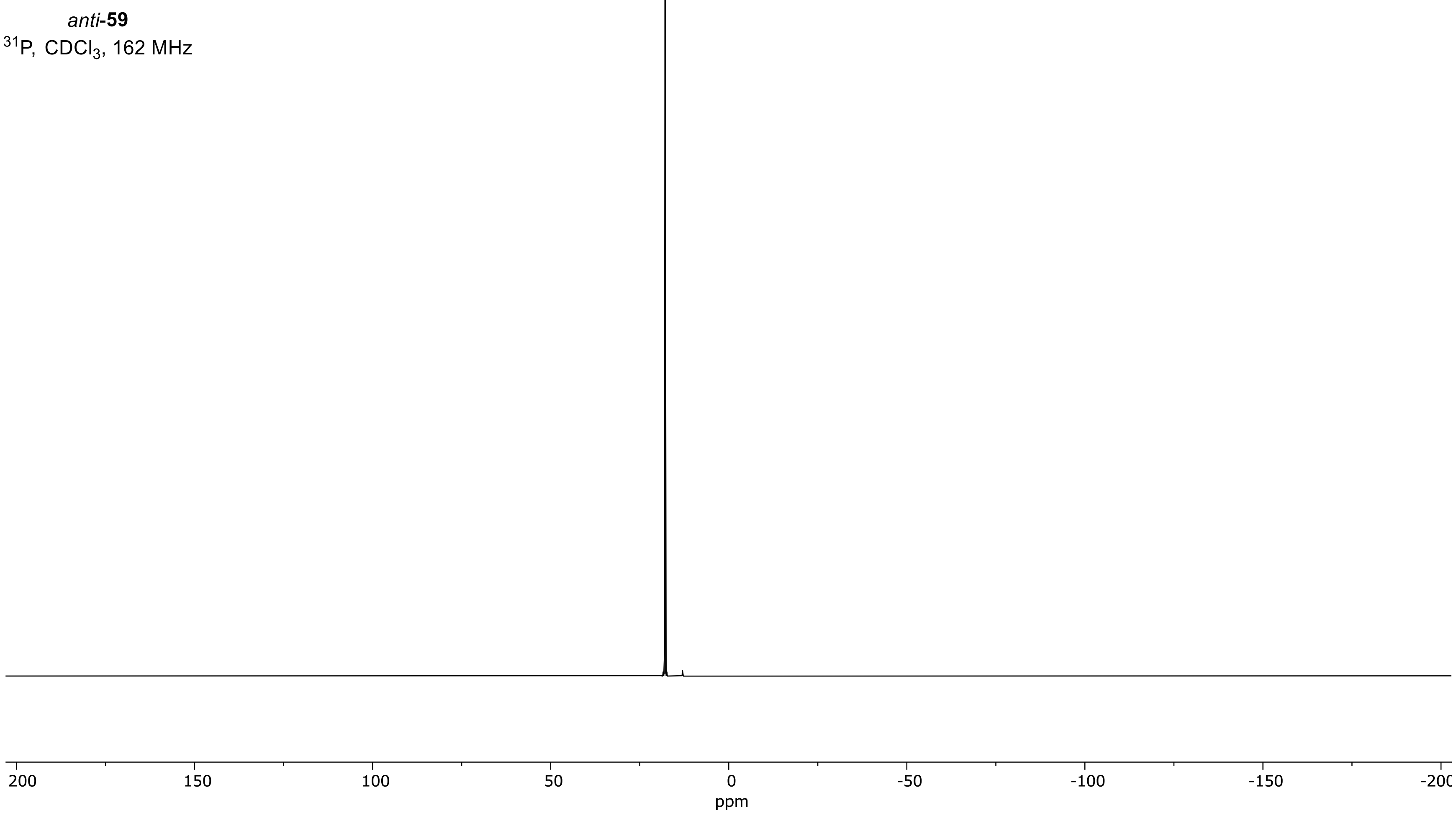

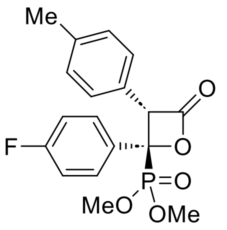

anti-**60**

^{1}H, $CDCl_3$, 400 MHz

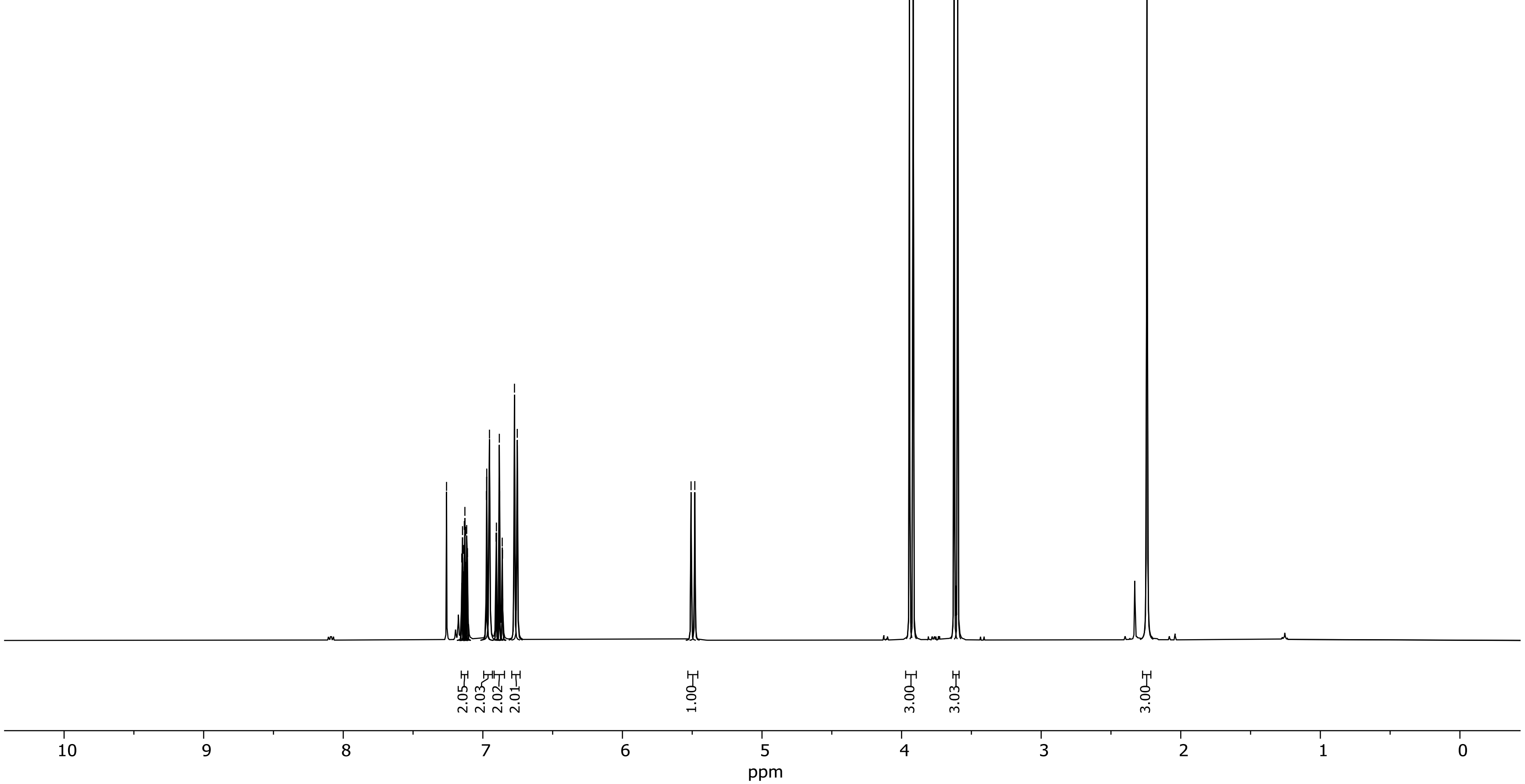

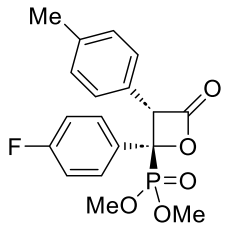

anti-**60**

^{13}C, $CDCl_3$, 101 MHz

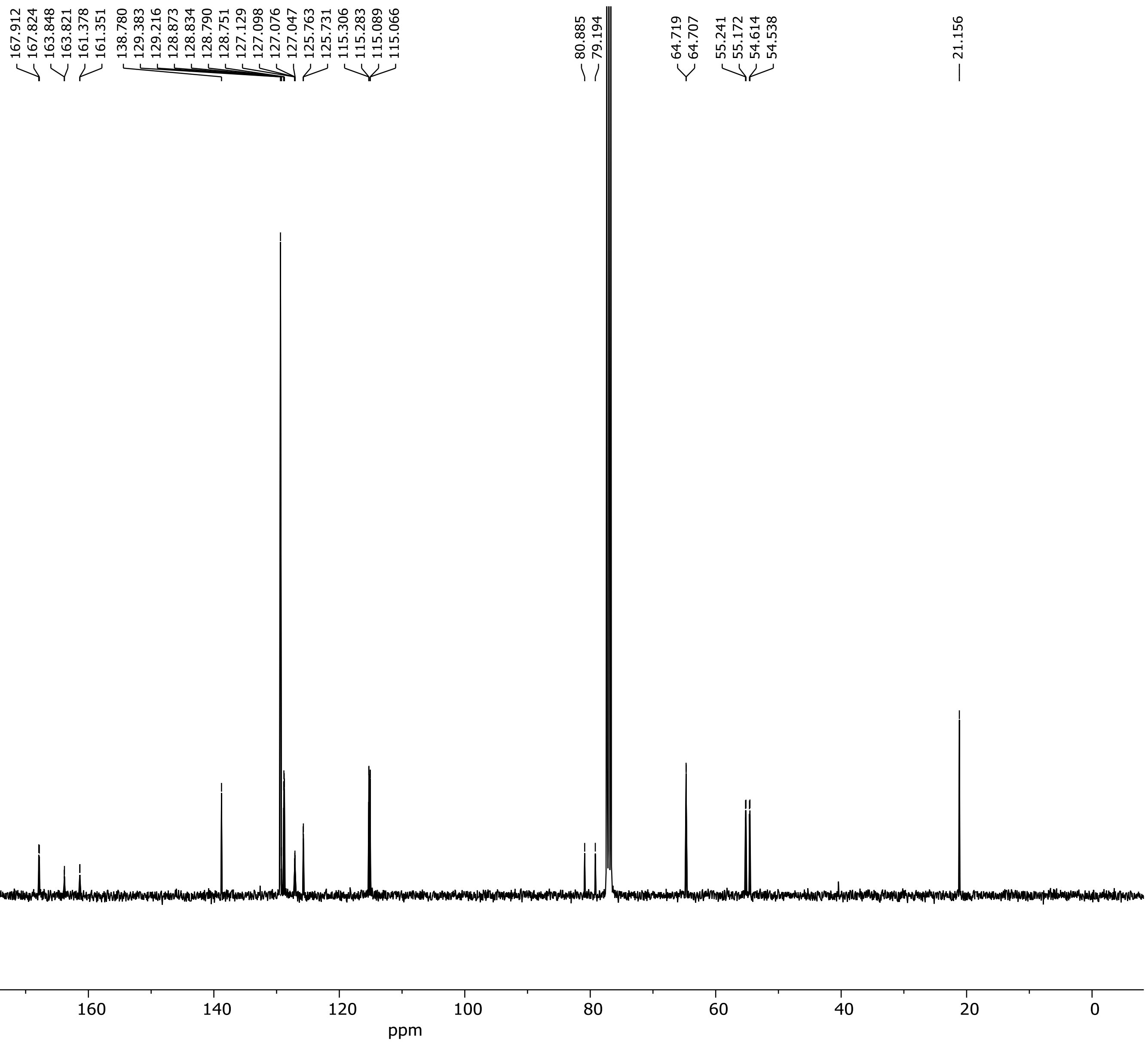

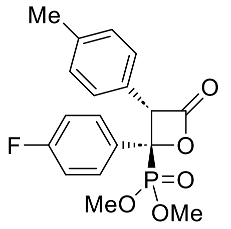

anti-**60**

^{31}P, $CDCl_3$, 162 MHz

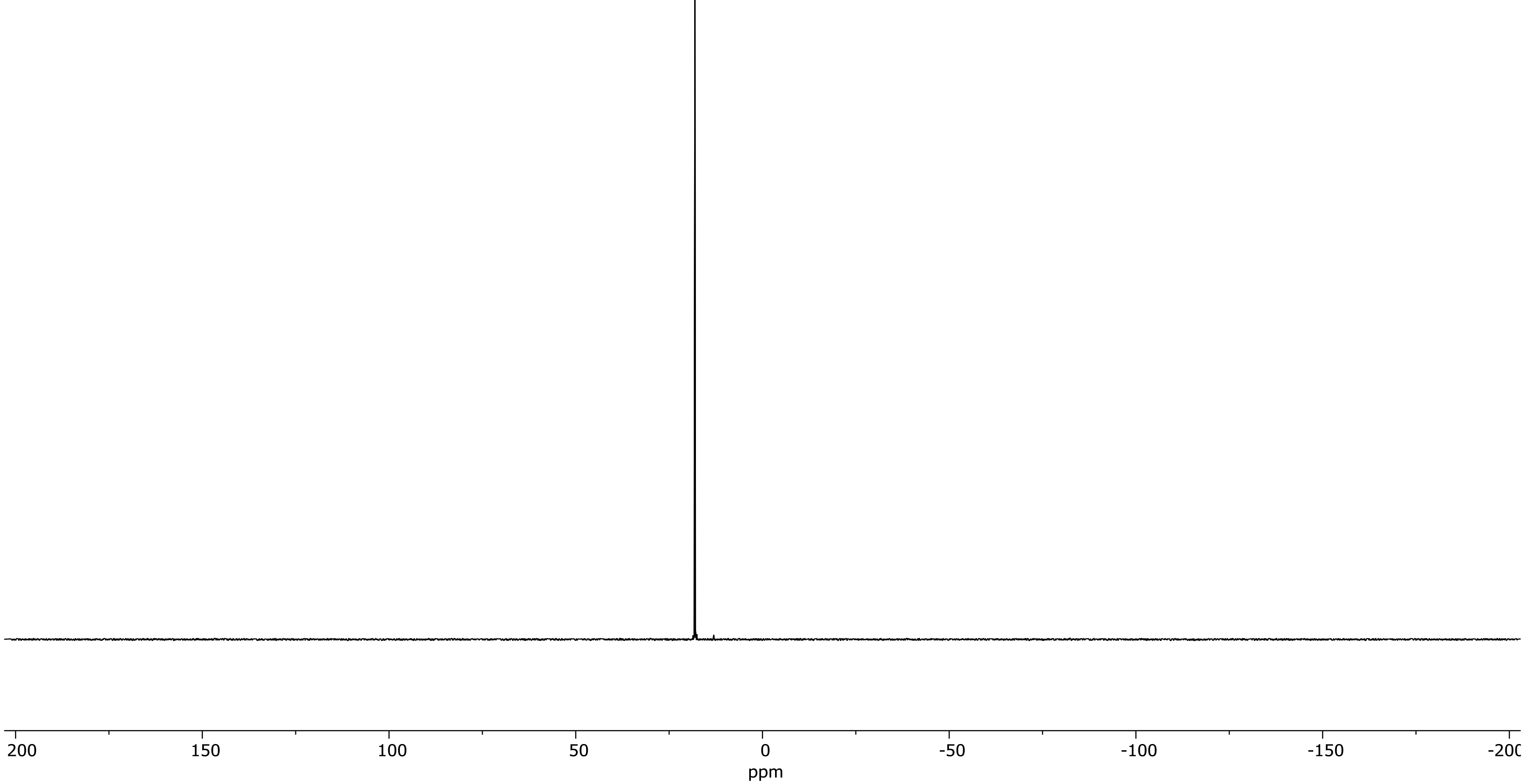

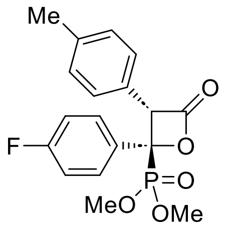

anti-**60**

^{19}F, $CDCl_3$, 376 MHz

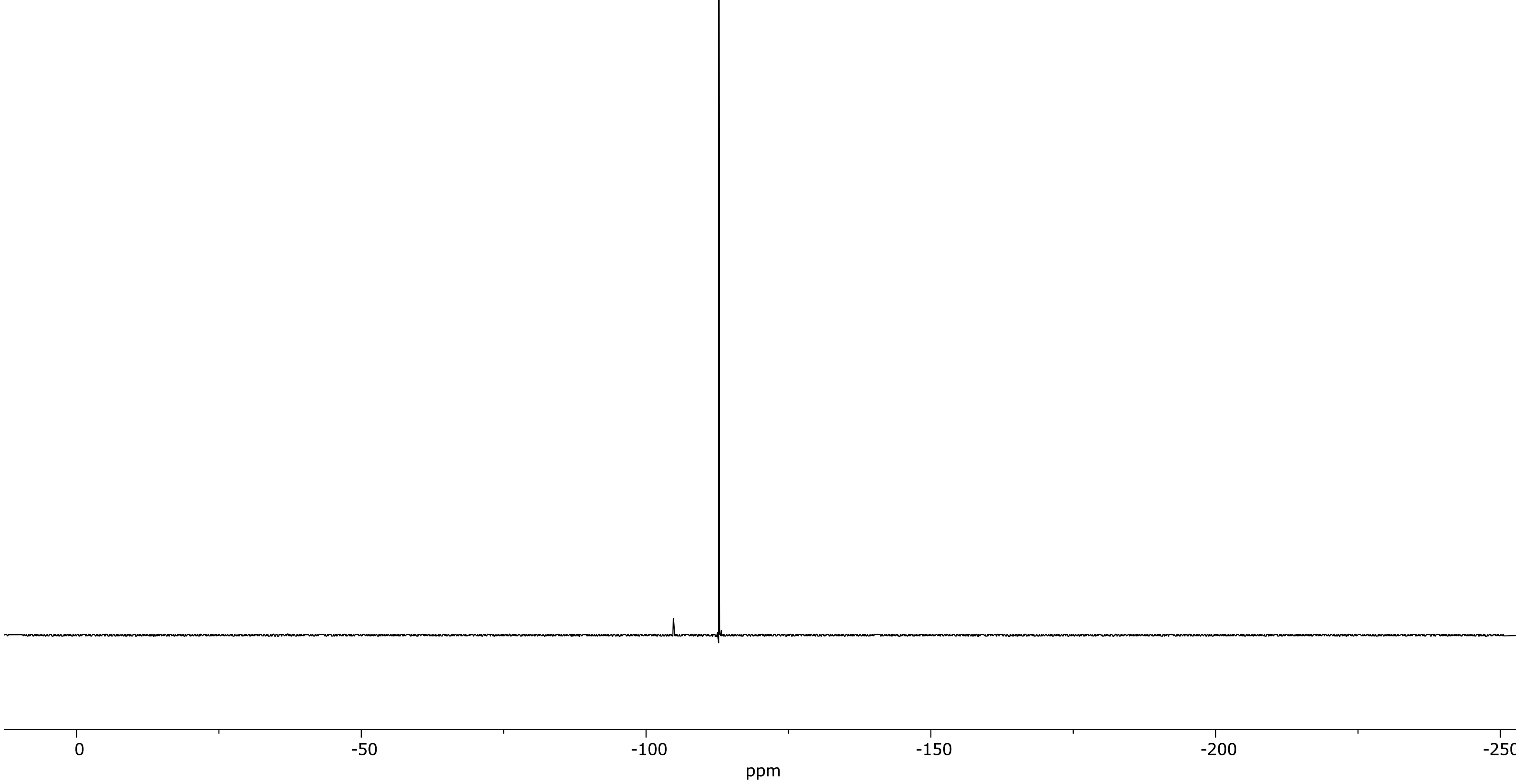

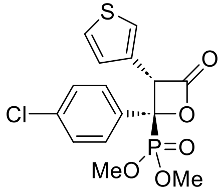

anti-**61**

^{1}H, $CDCl_3$, 400 MHz

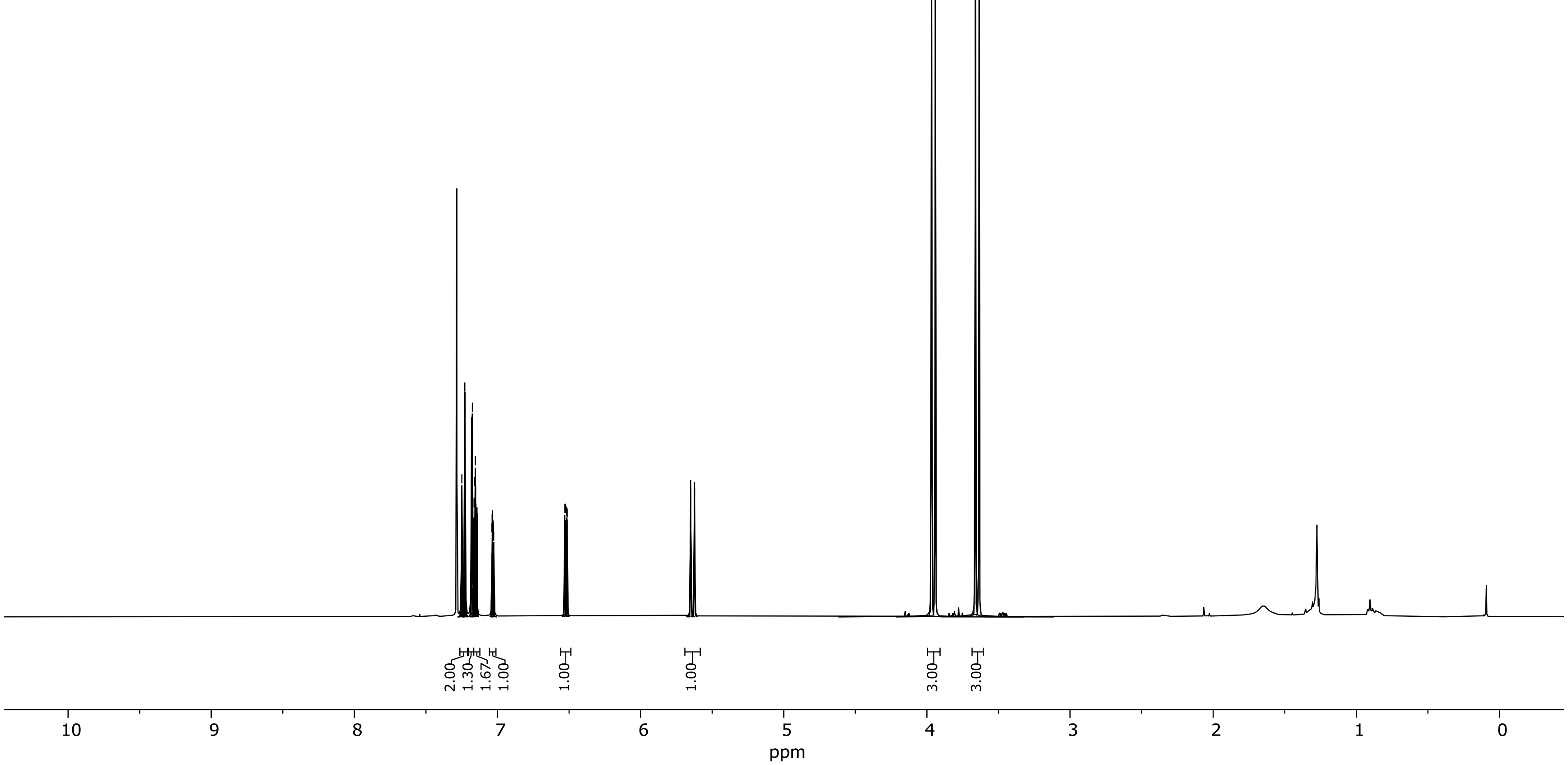

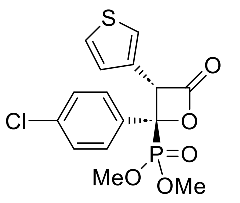

anti-**61**

^{13}C, $CDCl_3$, 101 MHz

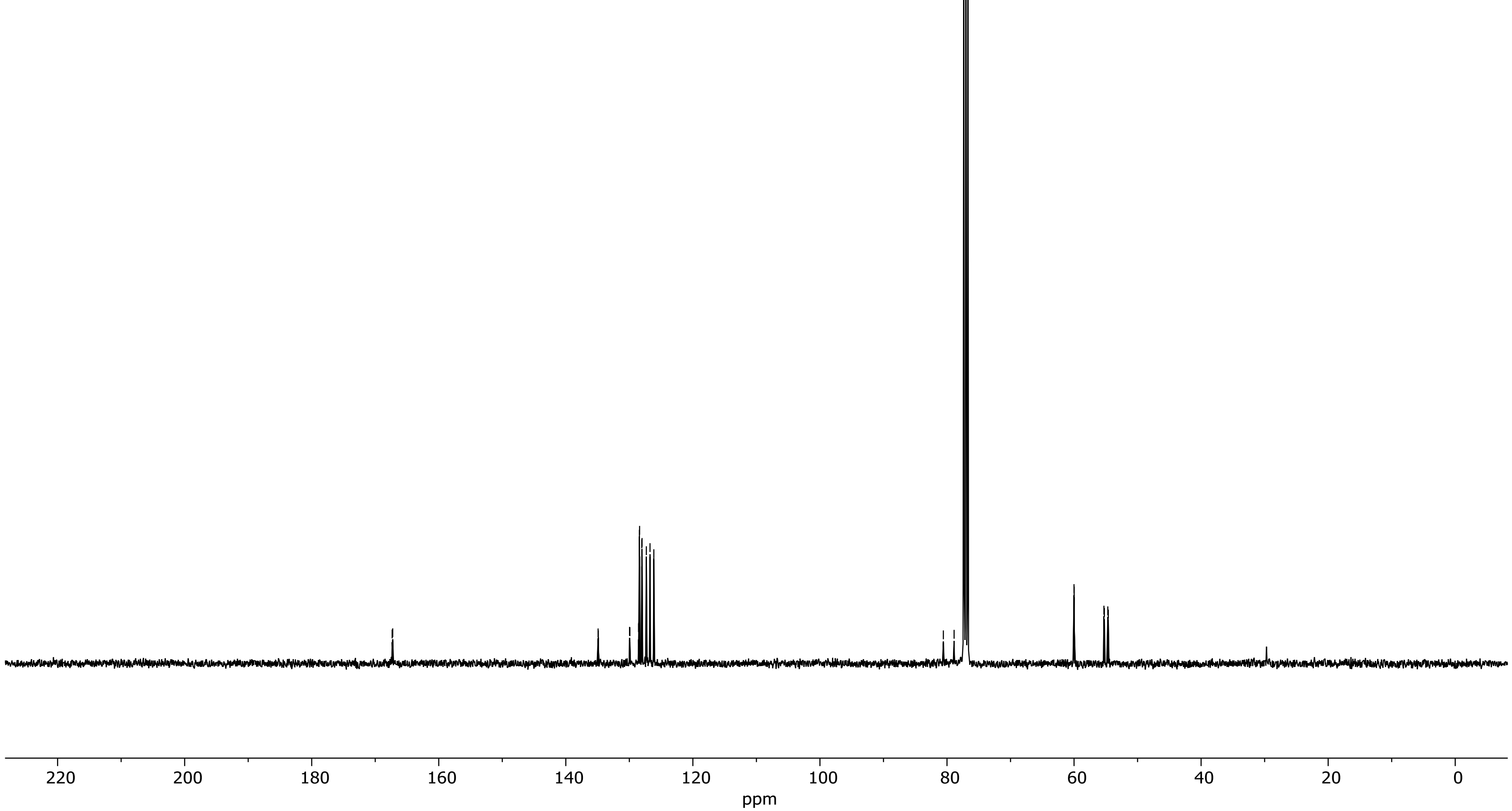

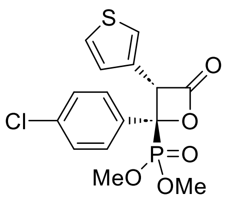

anti-**61**

^{31}P, $CDCl_3$, 162 MHz

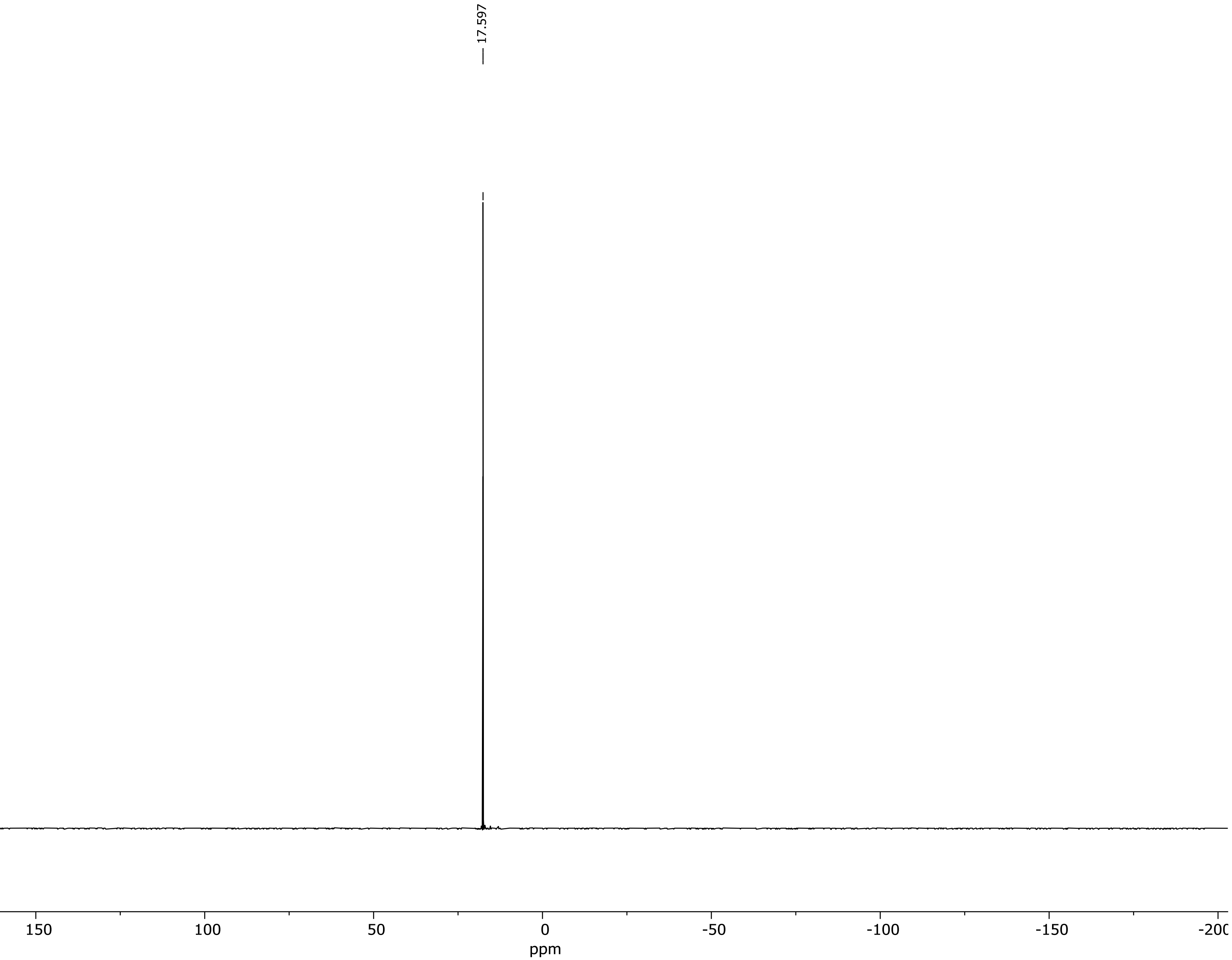

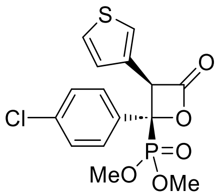

syn-**61**

^1H, $CDCl_3$, 400 MHz

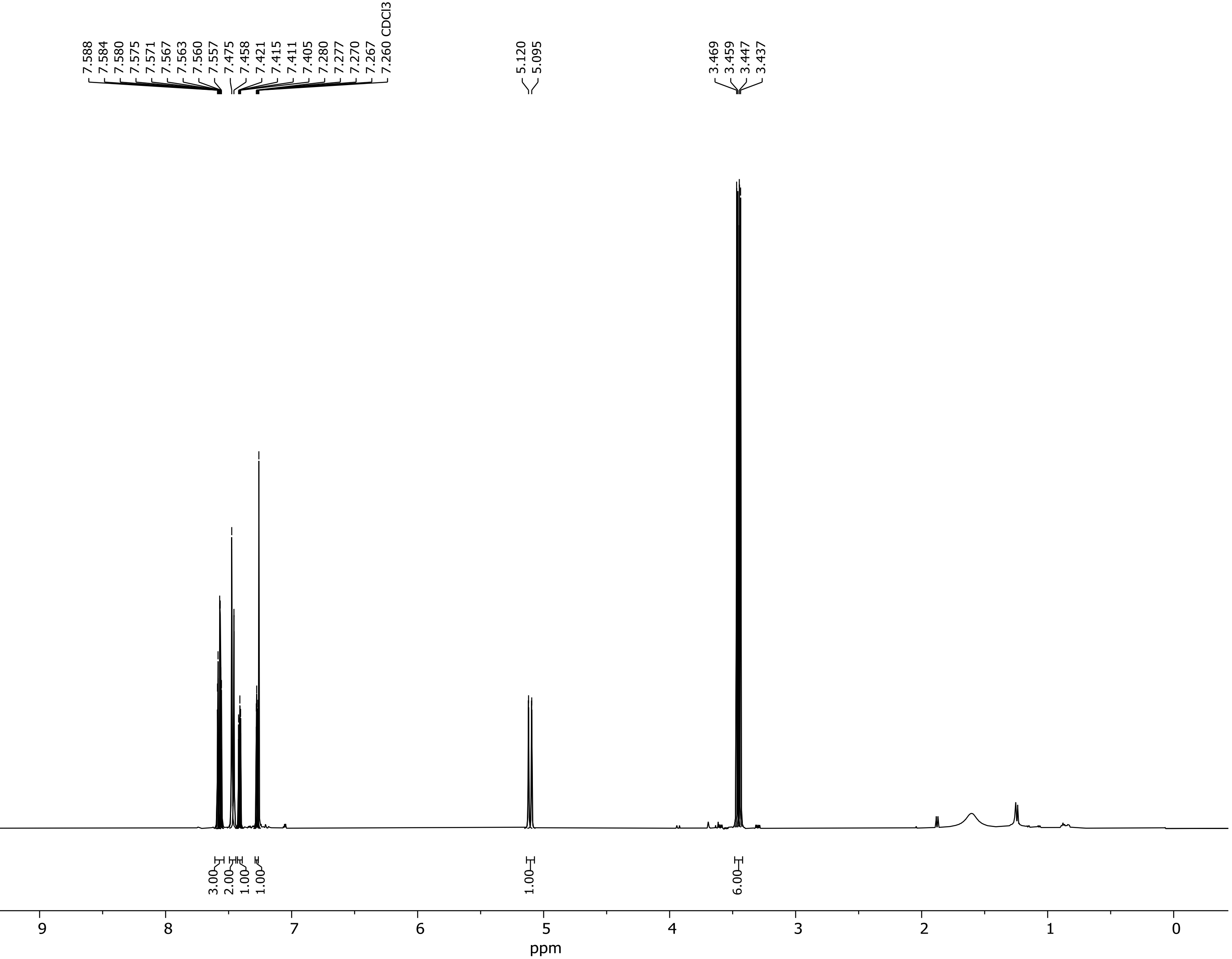

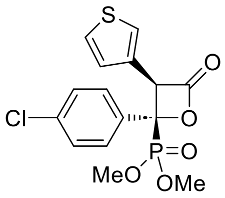

syn-**61**

^{13}C, $CDCl_3$, 101 MHz

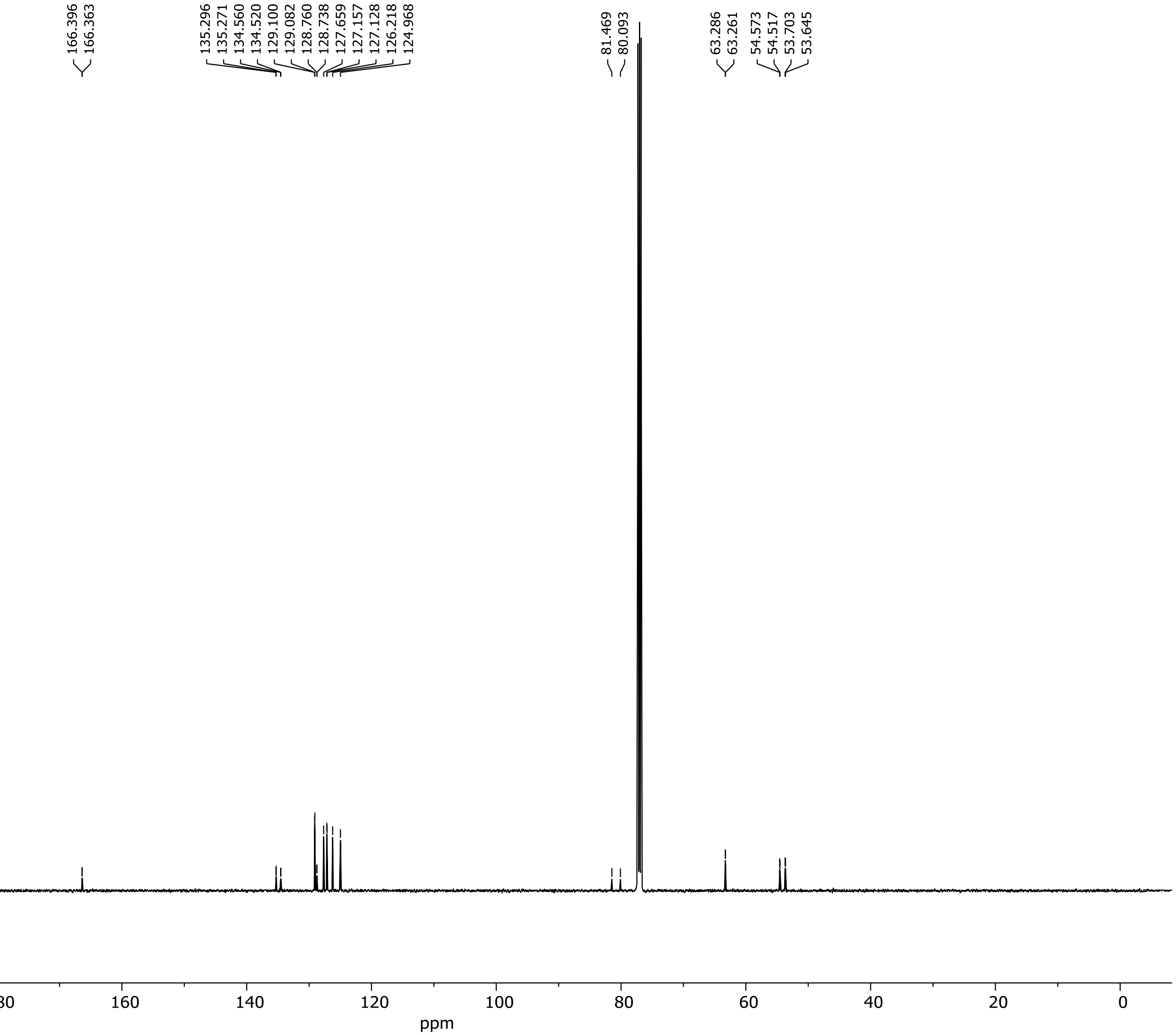

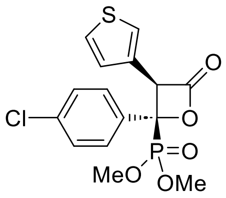

syn-**61**

^{31}P, $CDCl_3$, 162 MHz

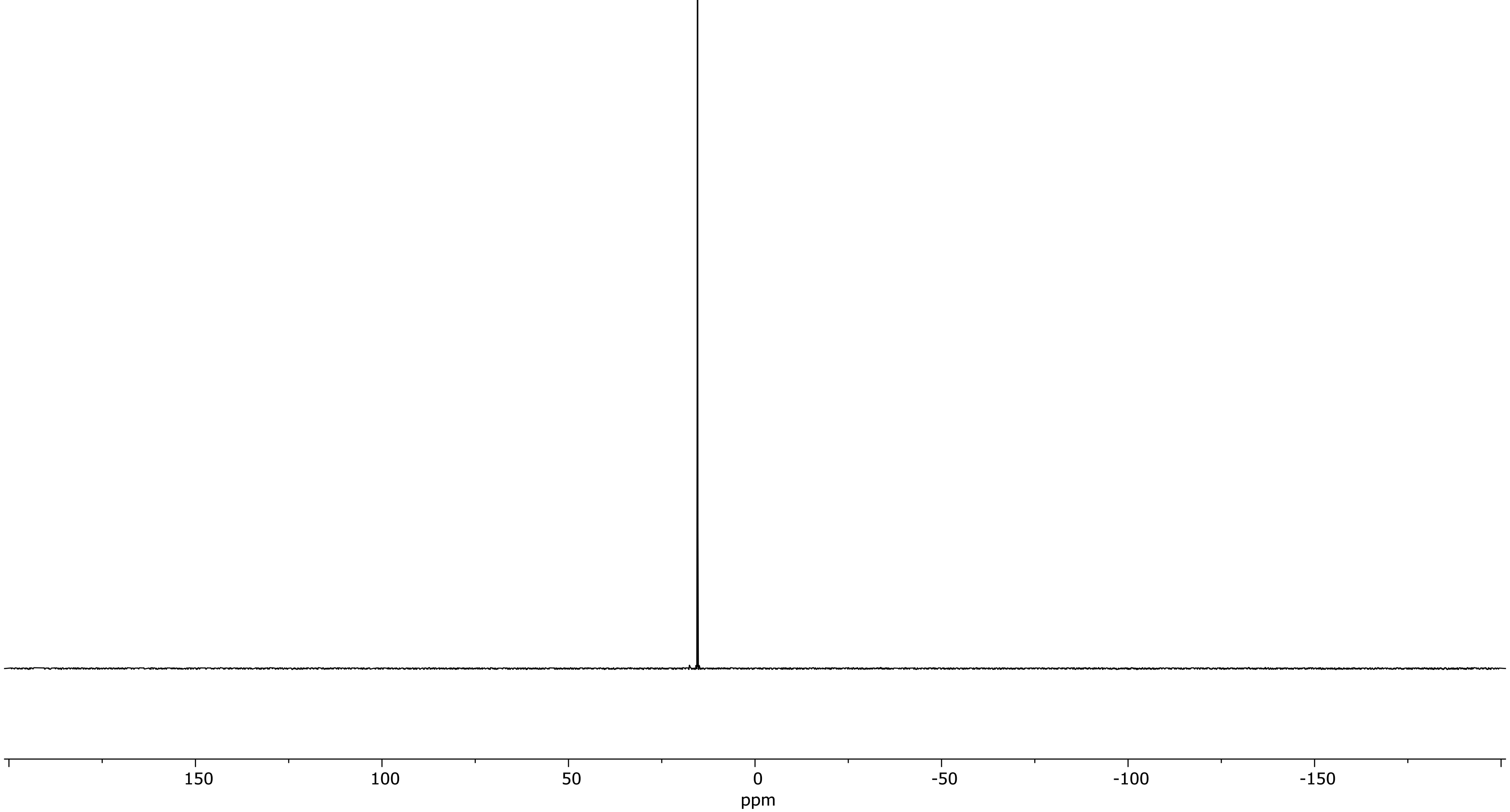

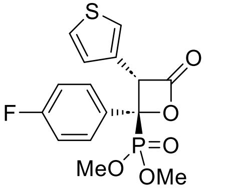

anti-**62**

^1H, $CDCl_3$, 400 MHz

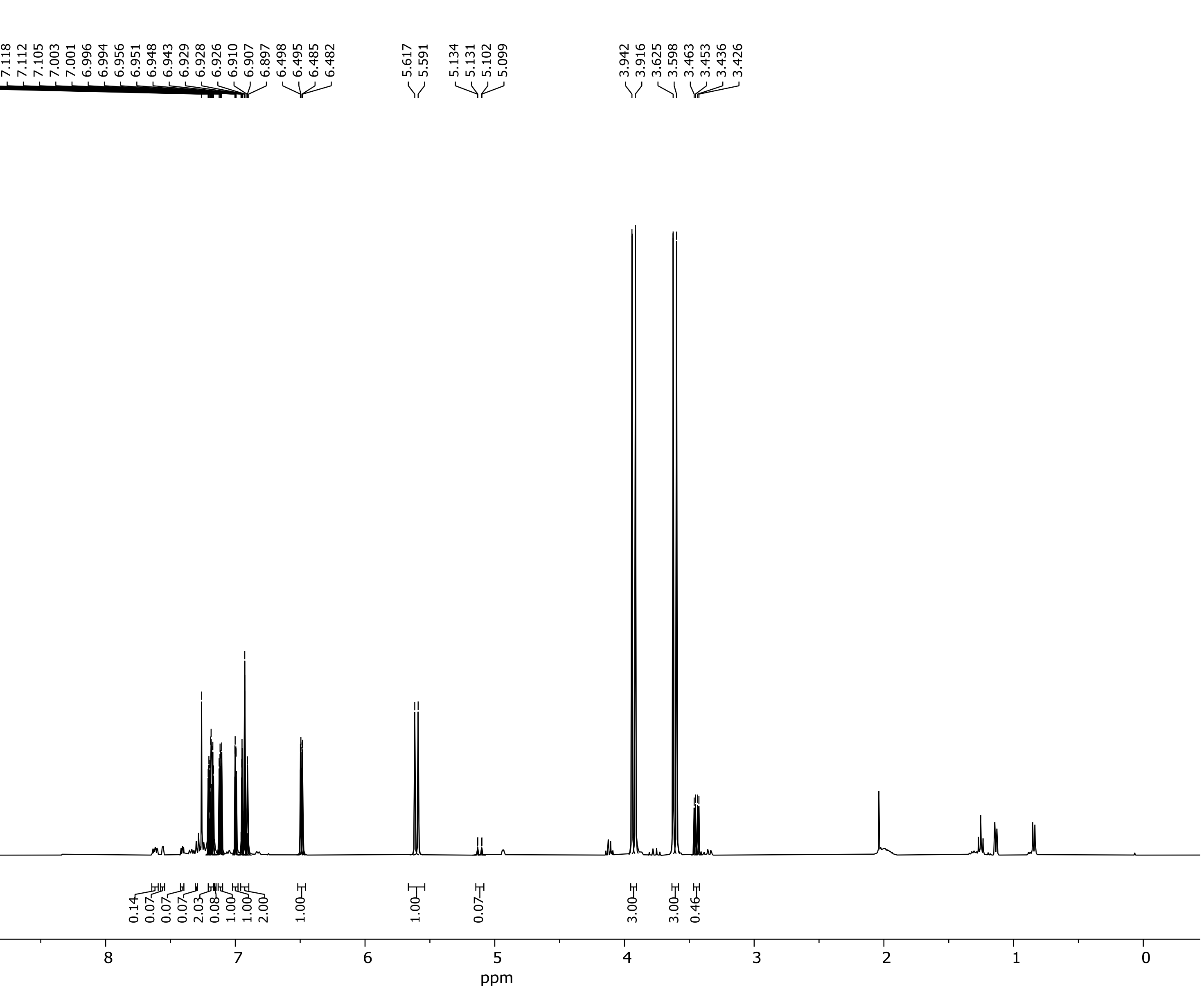

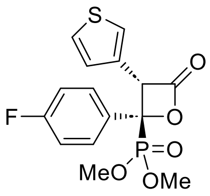

anti-**62**

^{13}C, $CDCl_3$, 101 MHz

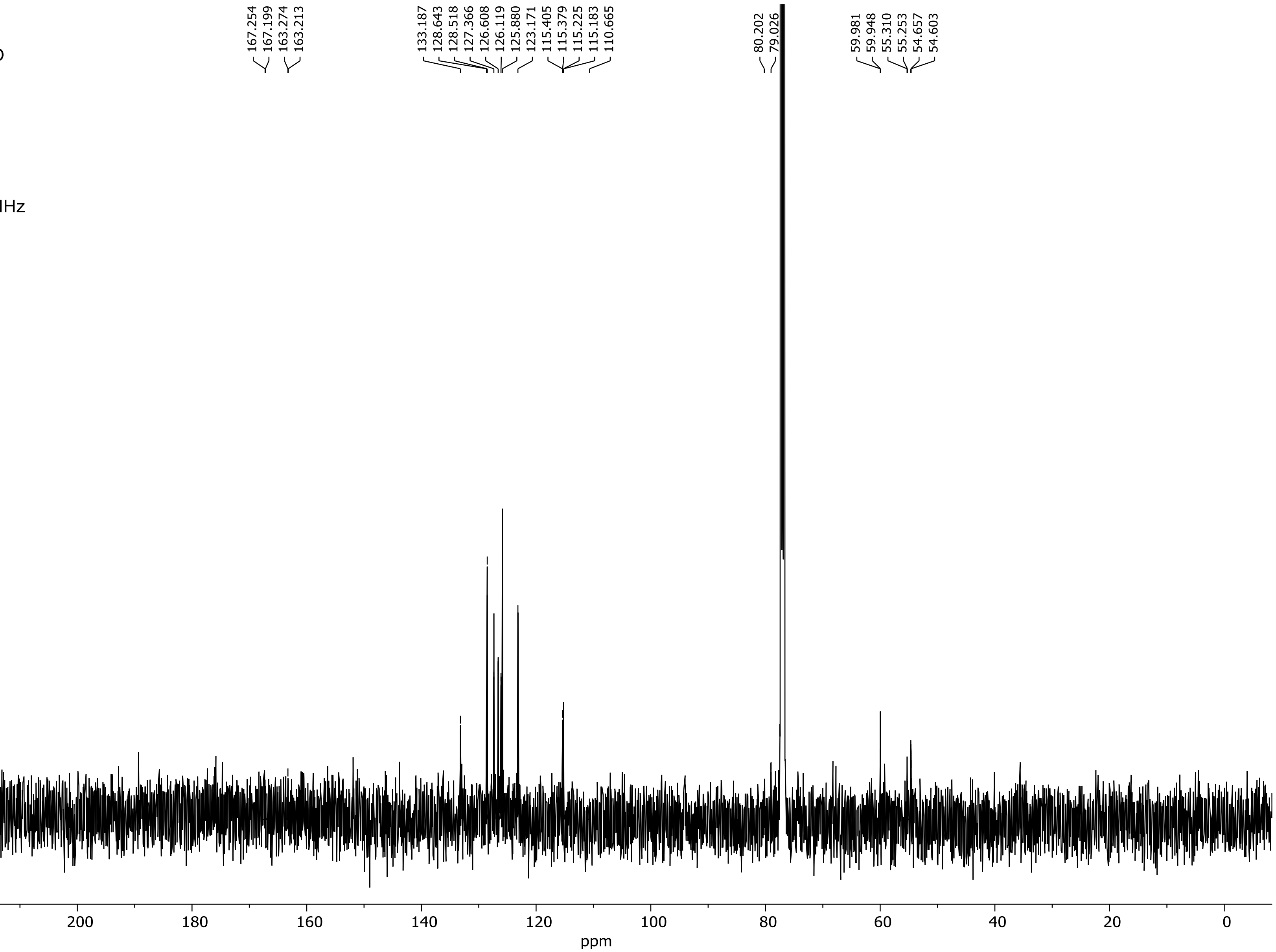

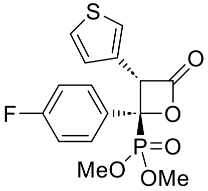

anti-**62**

^{31}P, $CDCl_3$, 162 MHz

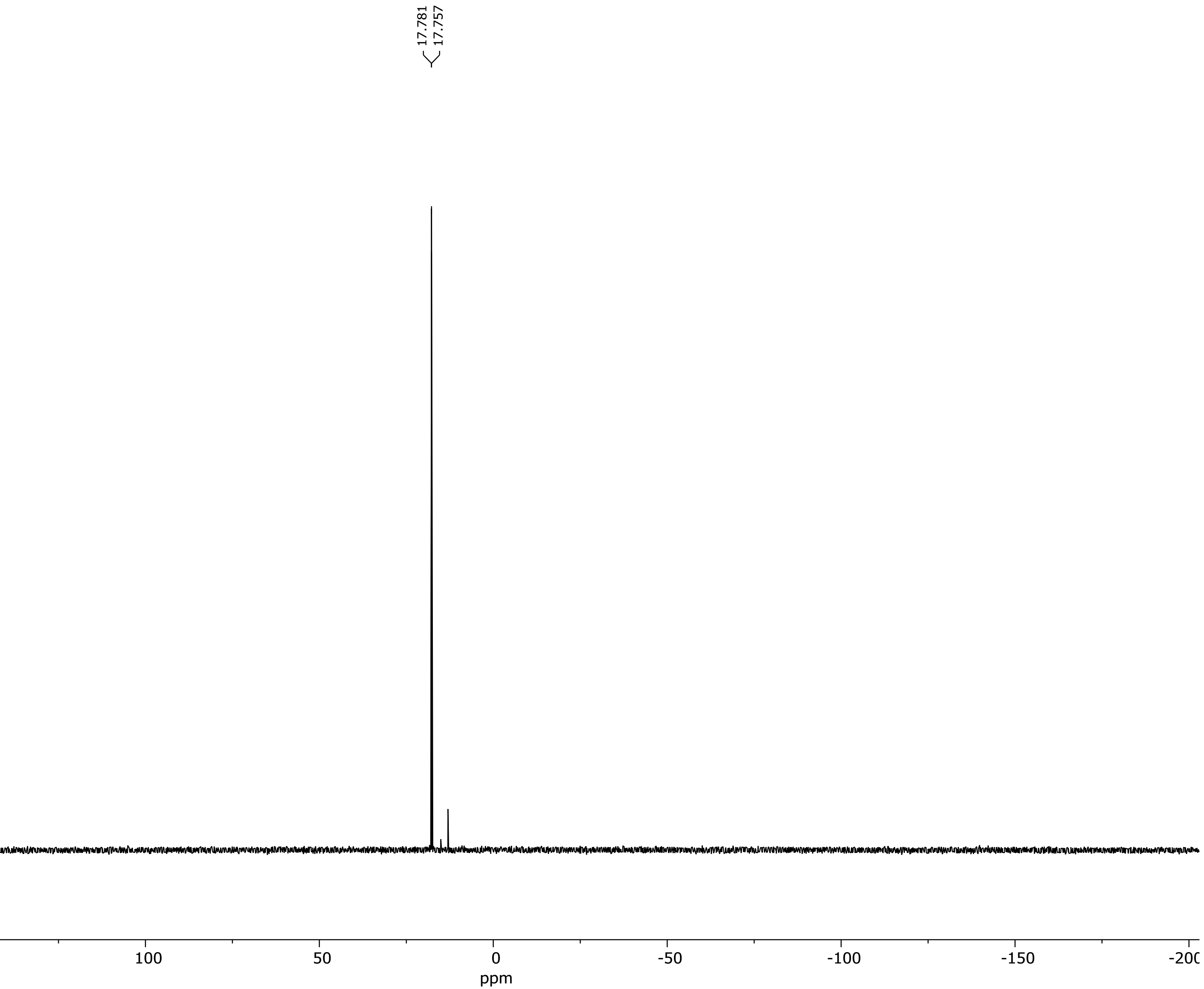

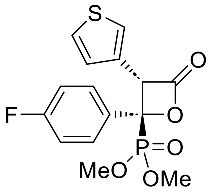

anti-**62**

^{19}F, $CDCl_3$, 376 MHz

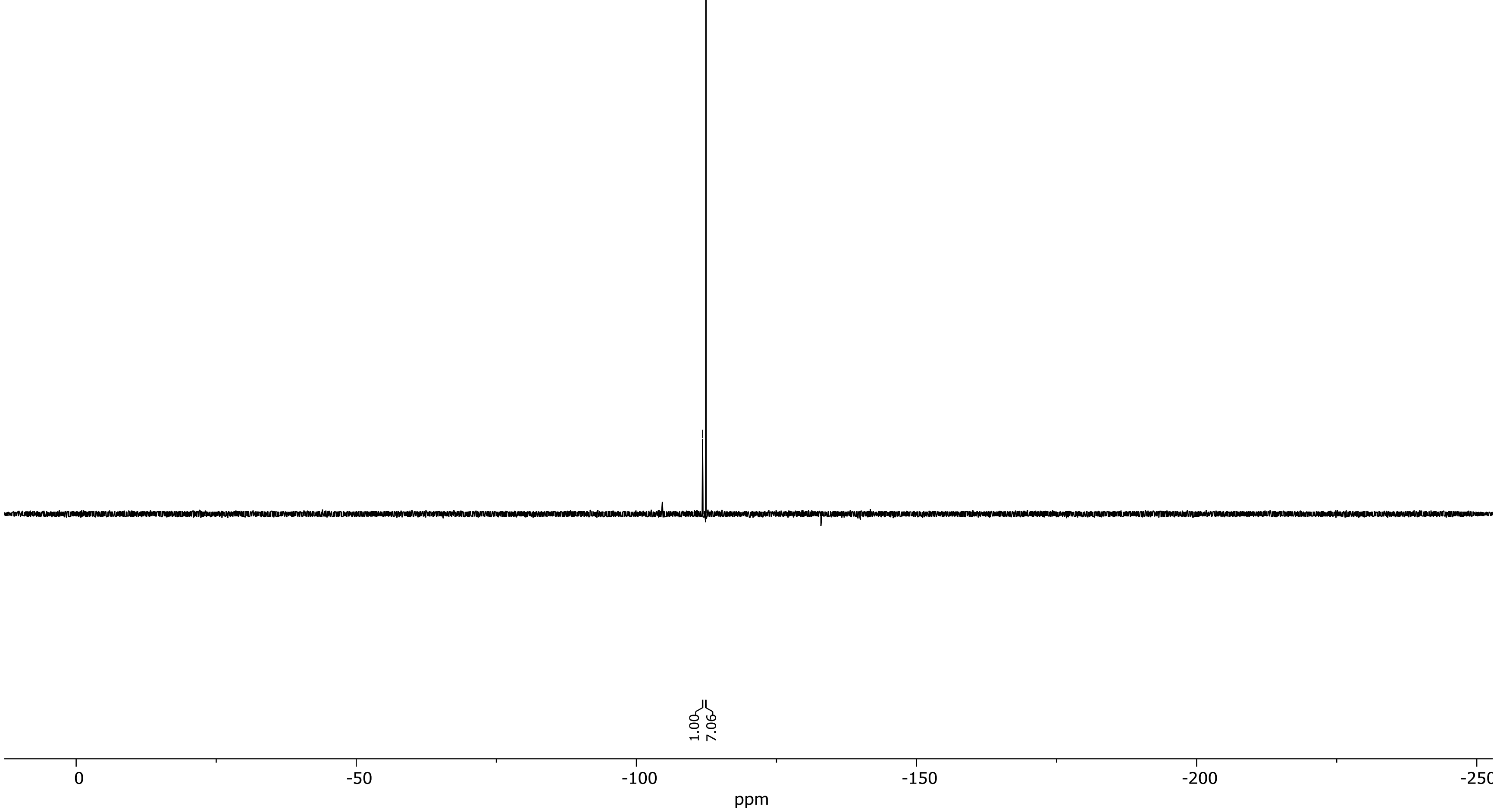

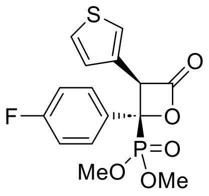

syn-**62**

^{1}H, $CDCl_3$, 400 MHz

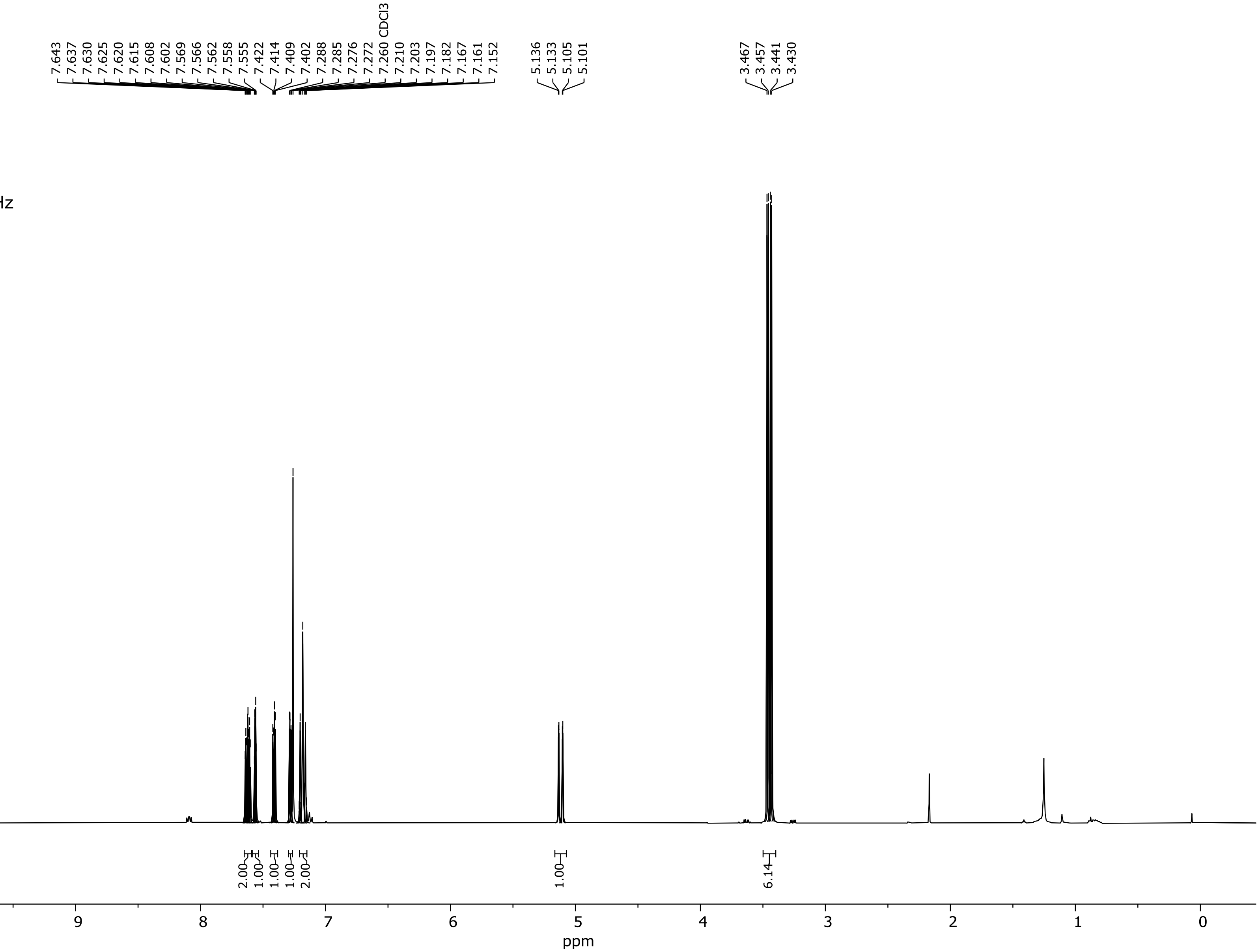

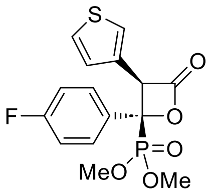

syn-**62**

^{13}C, $CDCl_3$, 101 MHz

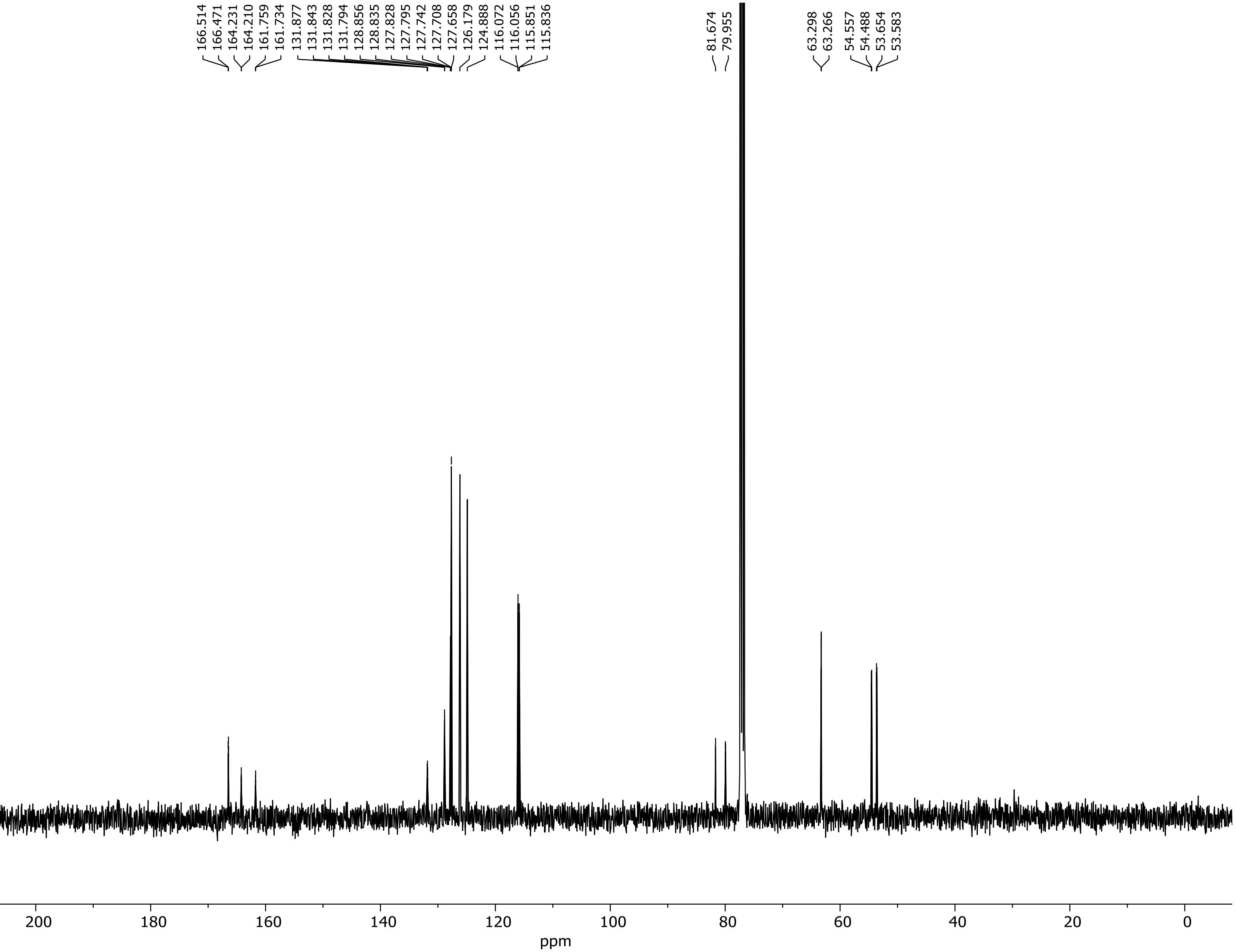

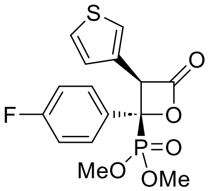

syn-**62**

^{31}P, $CDCl_3$, 162 MHz

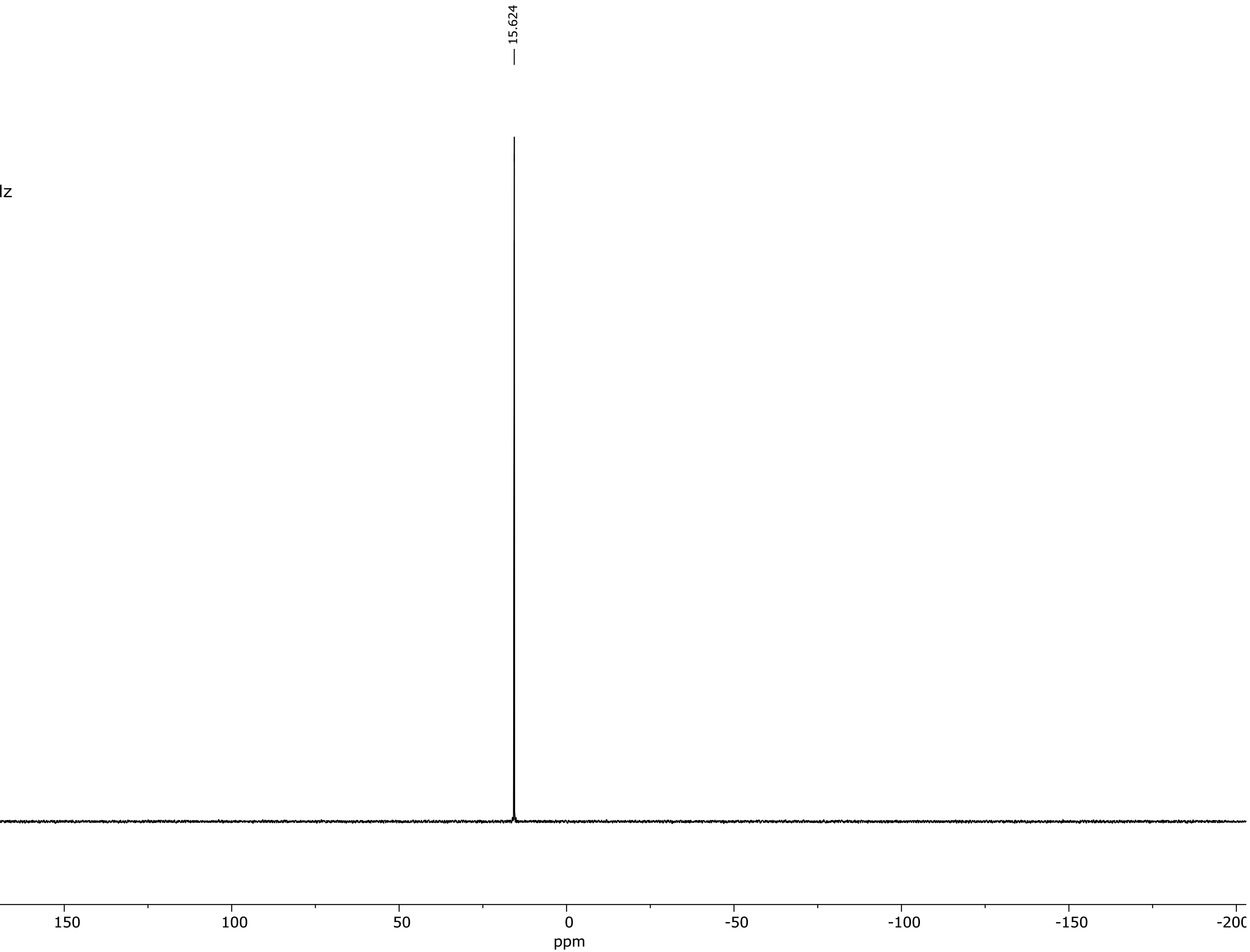

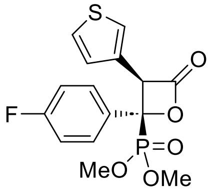

syn-**62**

^{19}F, $CDCl_3$, 376 MHz

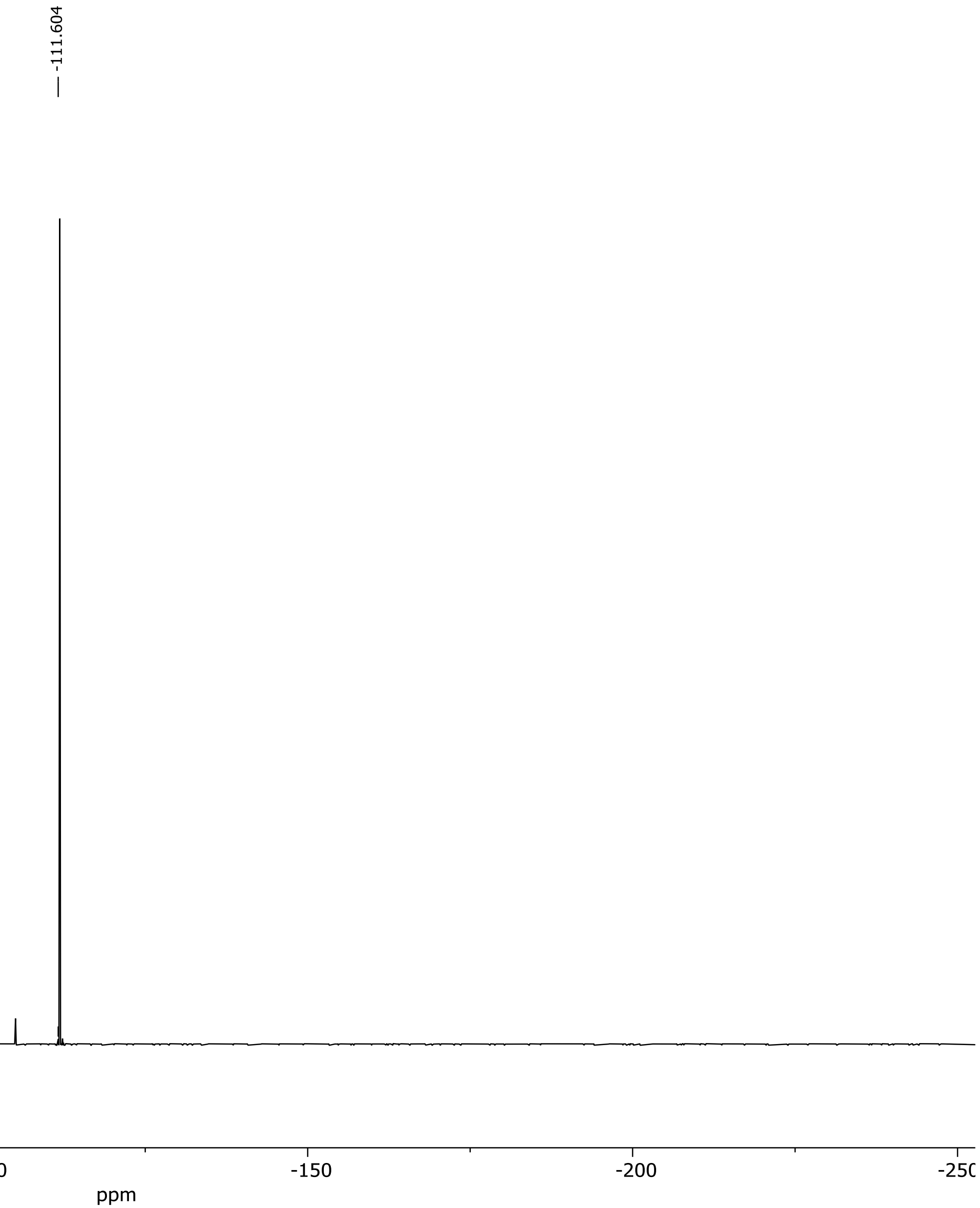

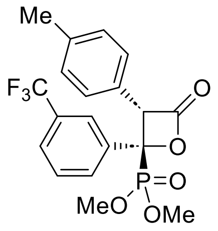

anti-**63**

^{1}H, $CDCl_3$, 400 MHz

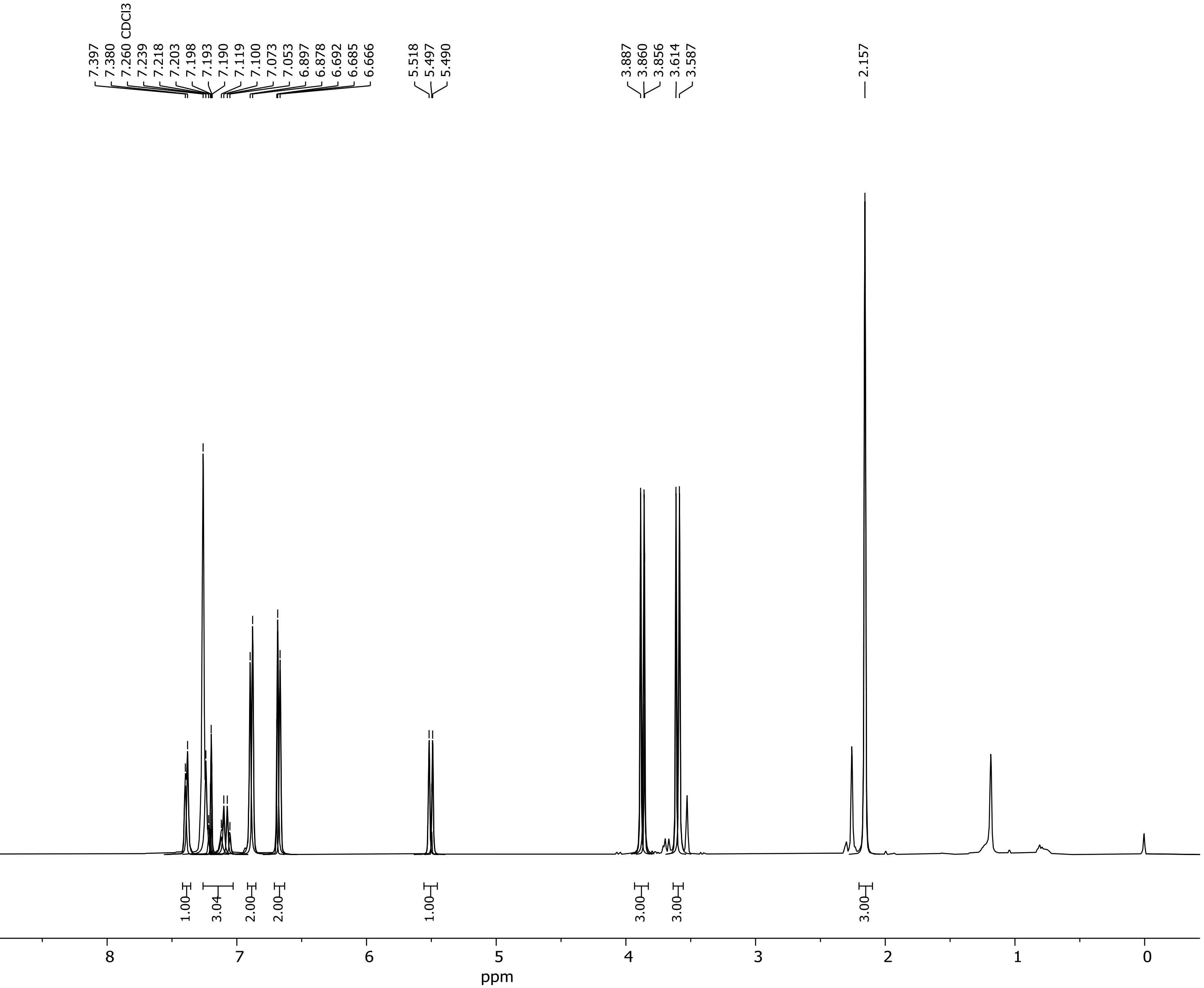

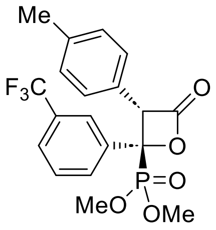

anti-**63**

^{13}C, $CDCl_3$, 101 MHz

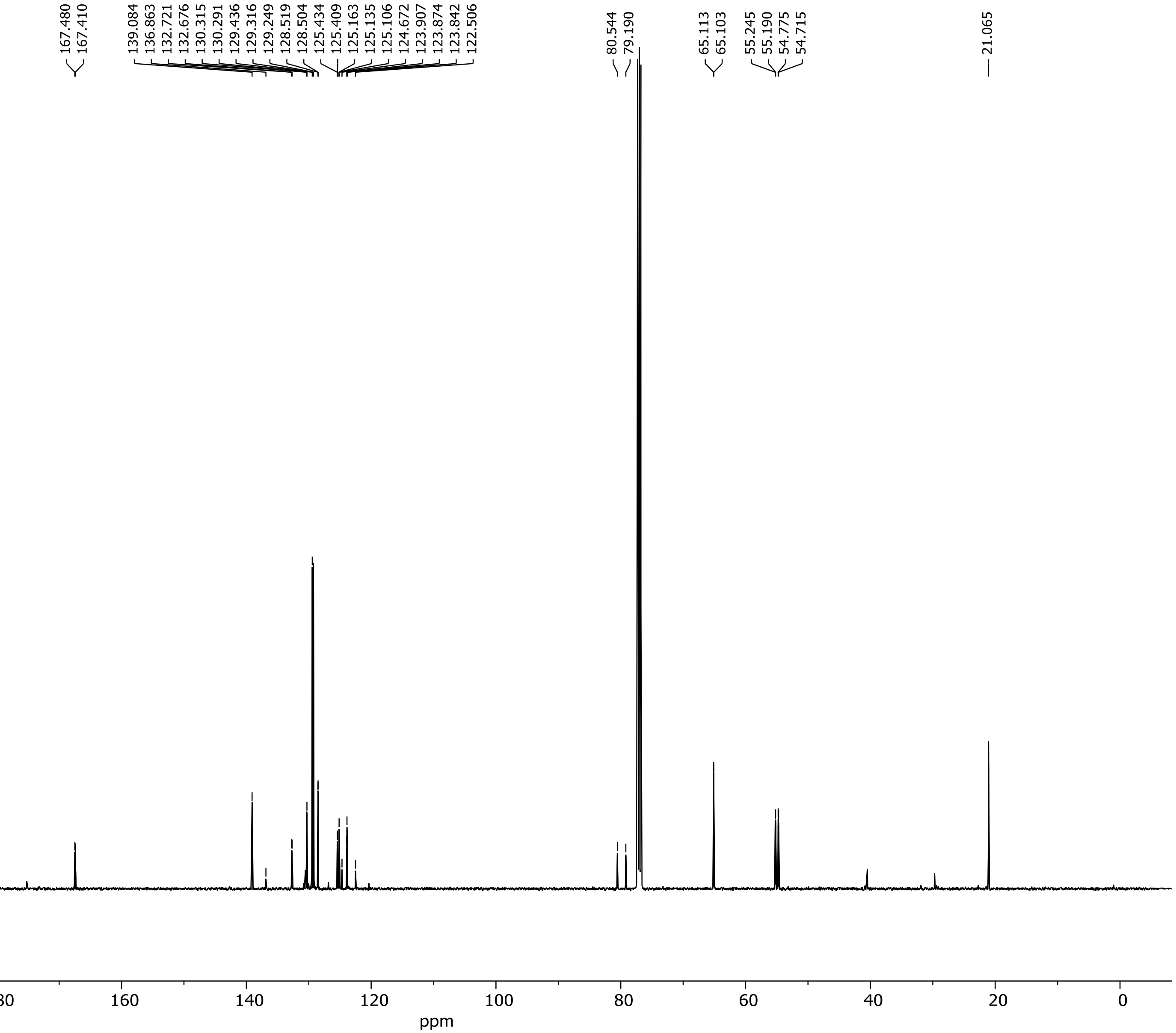

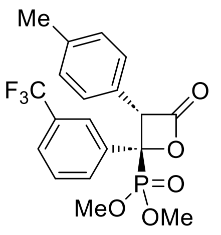

anti-**63**

^{19}F, $CDCl_3$, 376 MHz

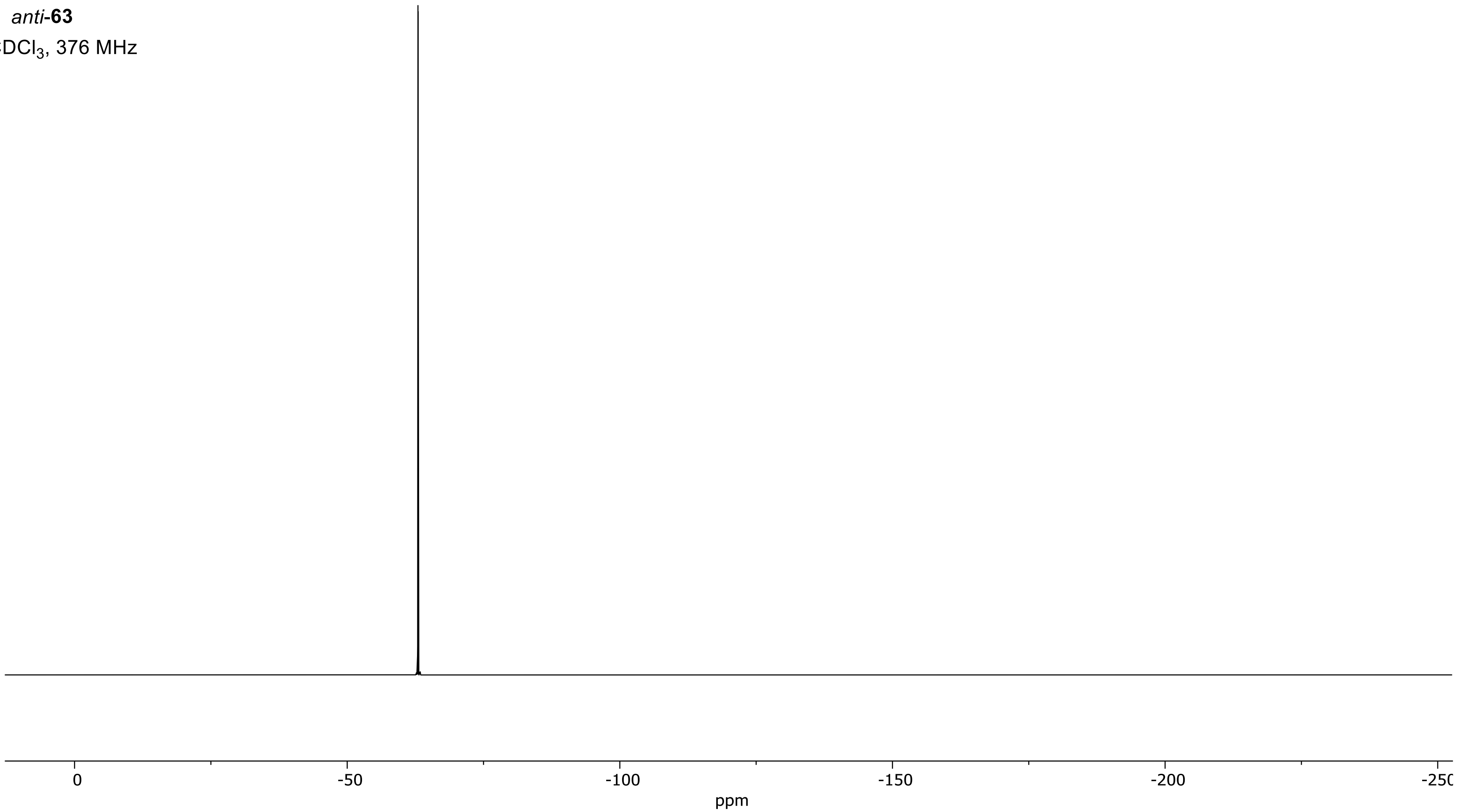

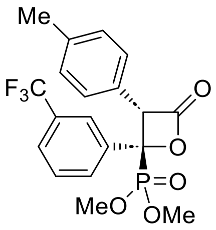

anti-**63**

^{31}P, $CDCl_3$, 162 MHz

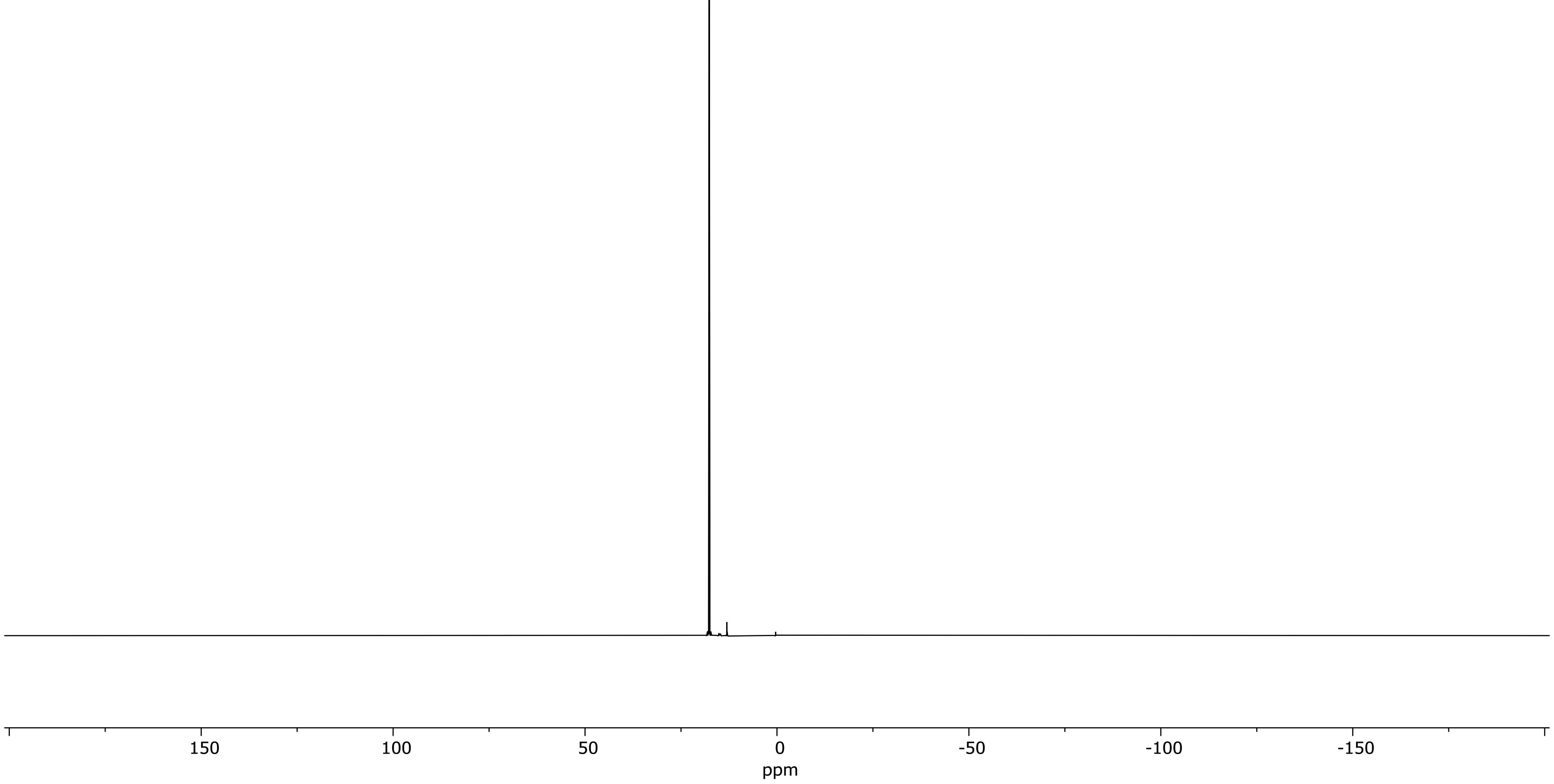

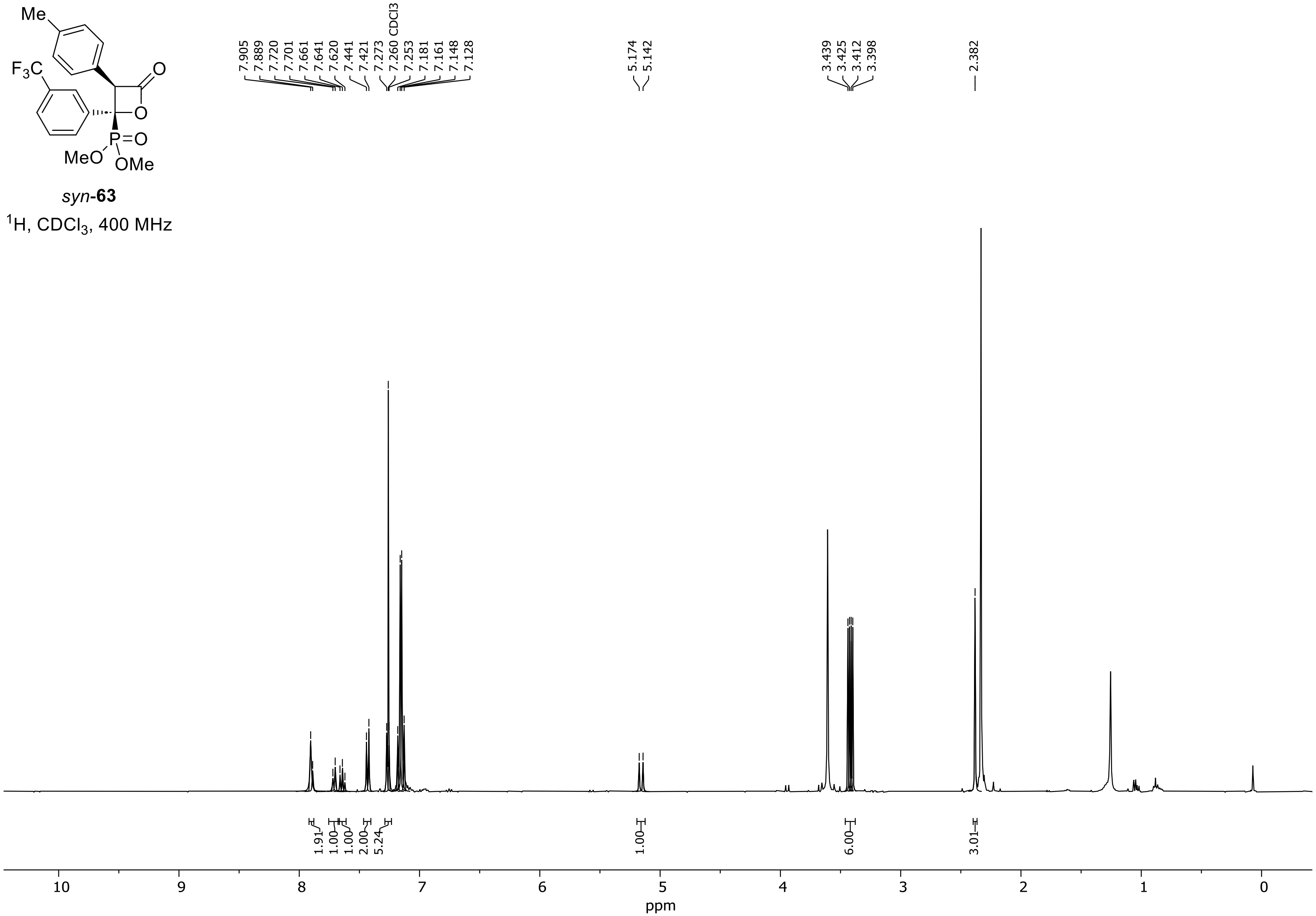

syn-**63**

^1H, $CDCl_3$, 400 MHz

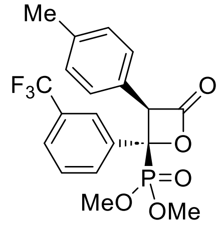

syn-**63**

^{13}C, $CDCl_3$, 101 MHz

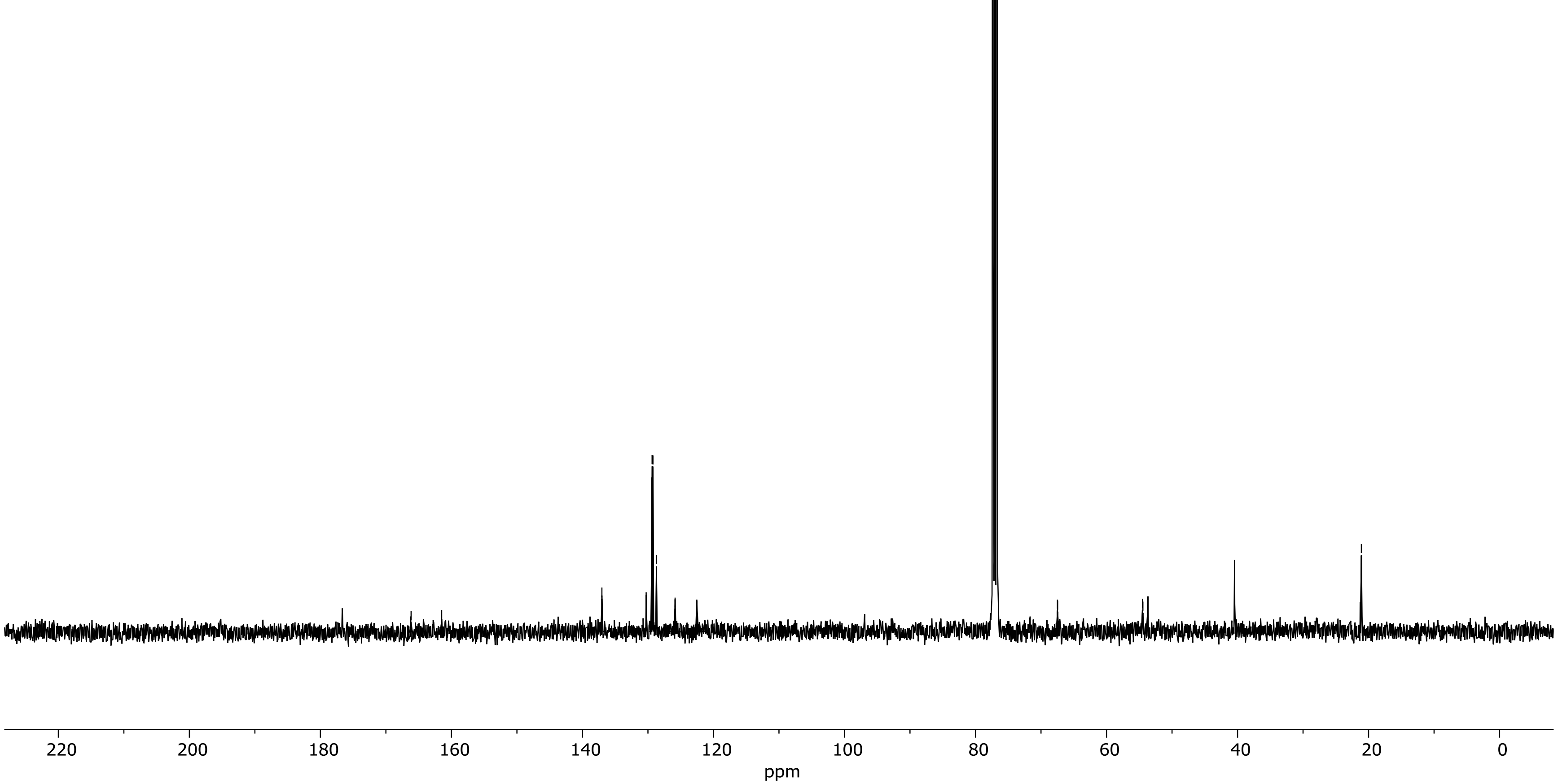

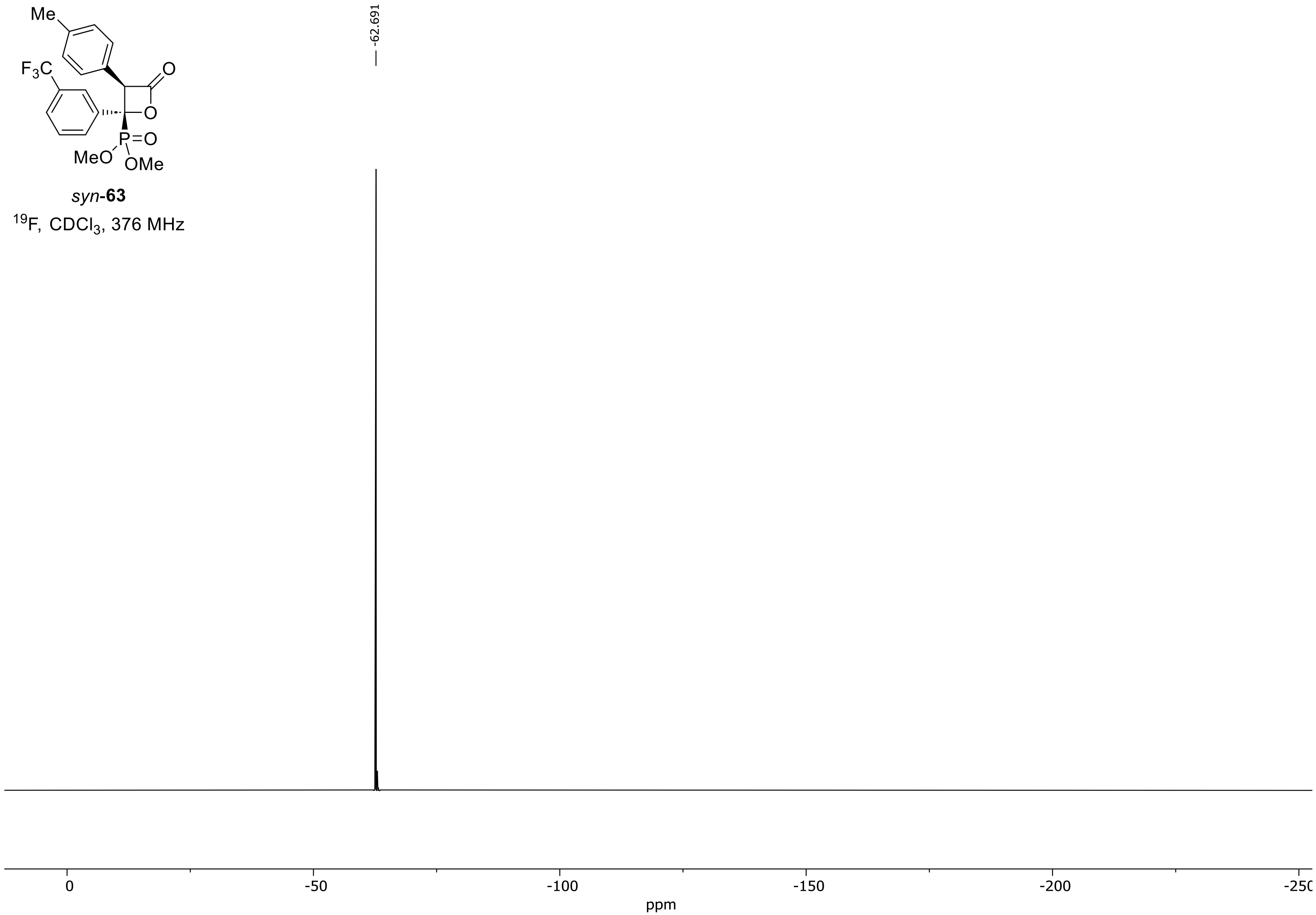

syn-**63**

^{19}F, $CDCl_3$, 376 MHz

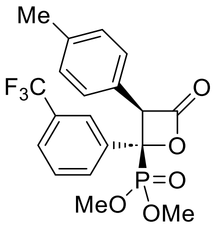

syn-**63**

^{31}P, $CDCl_3$, 162 MHz

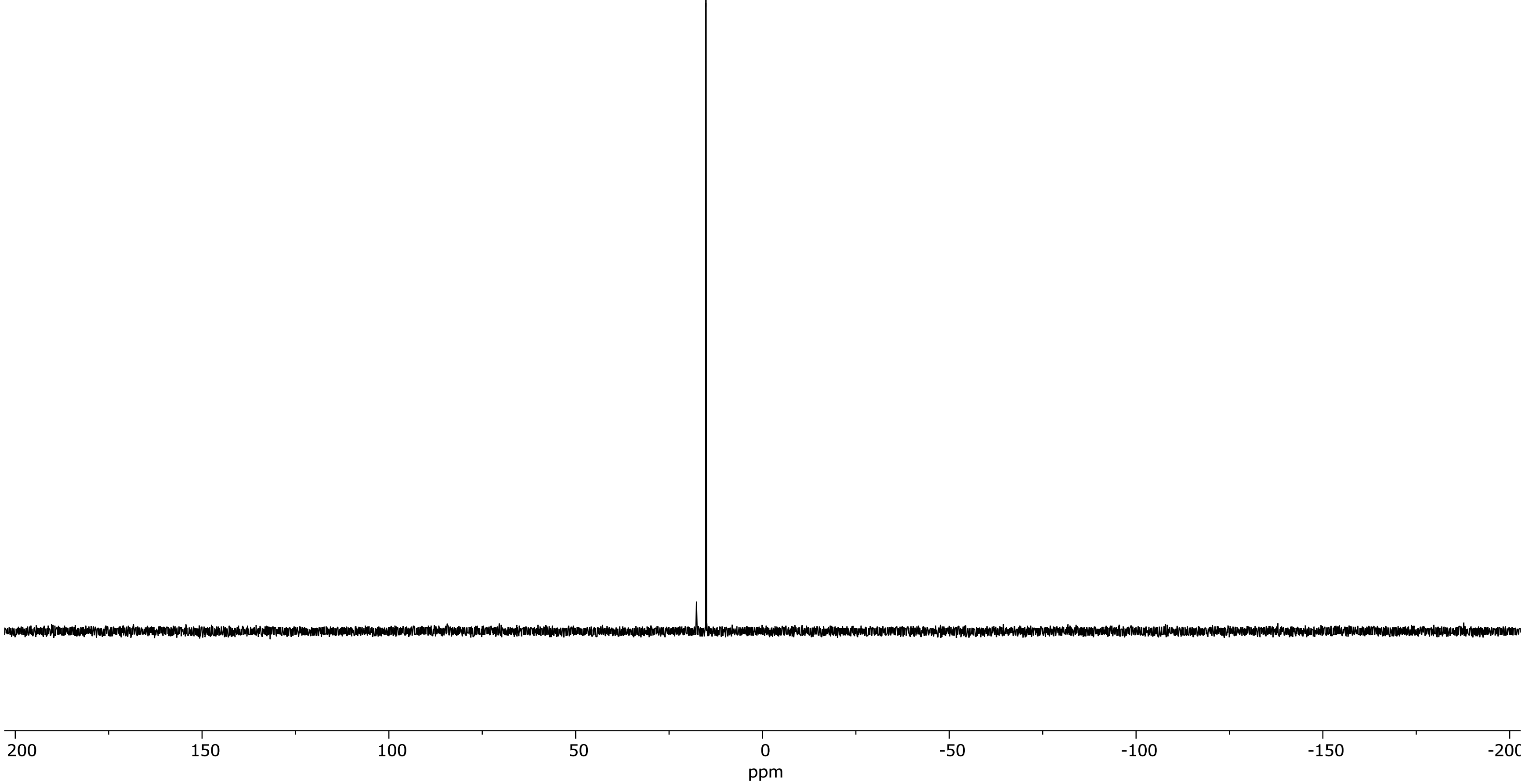

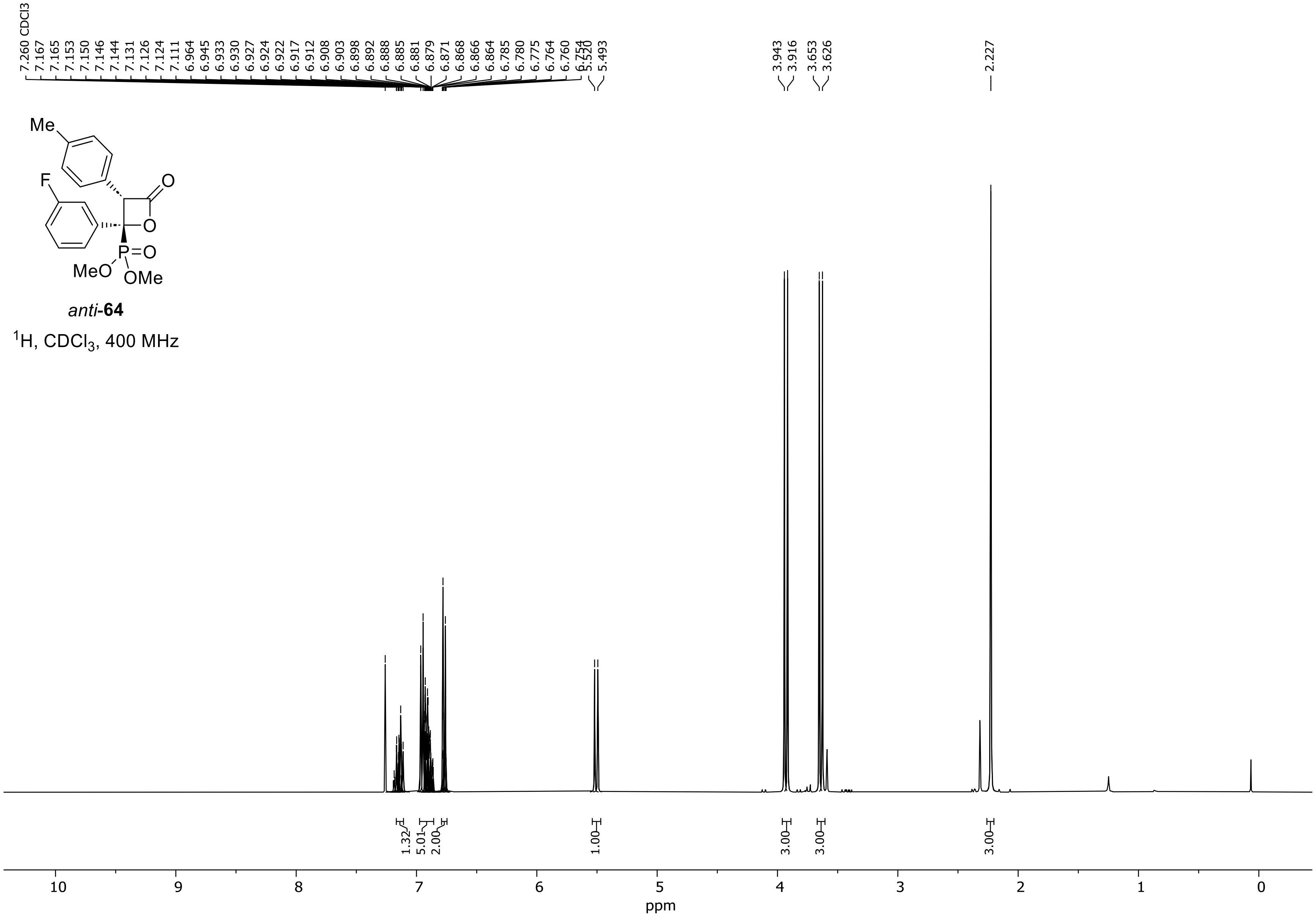

anti-**64**

^{1}H, $CDCl_3$, 400 MHz

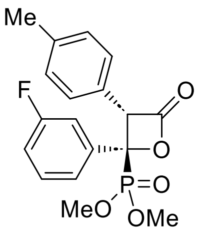

anti-**64**

^{13}C, $CDCl_3$, 101 MHz

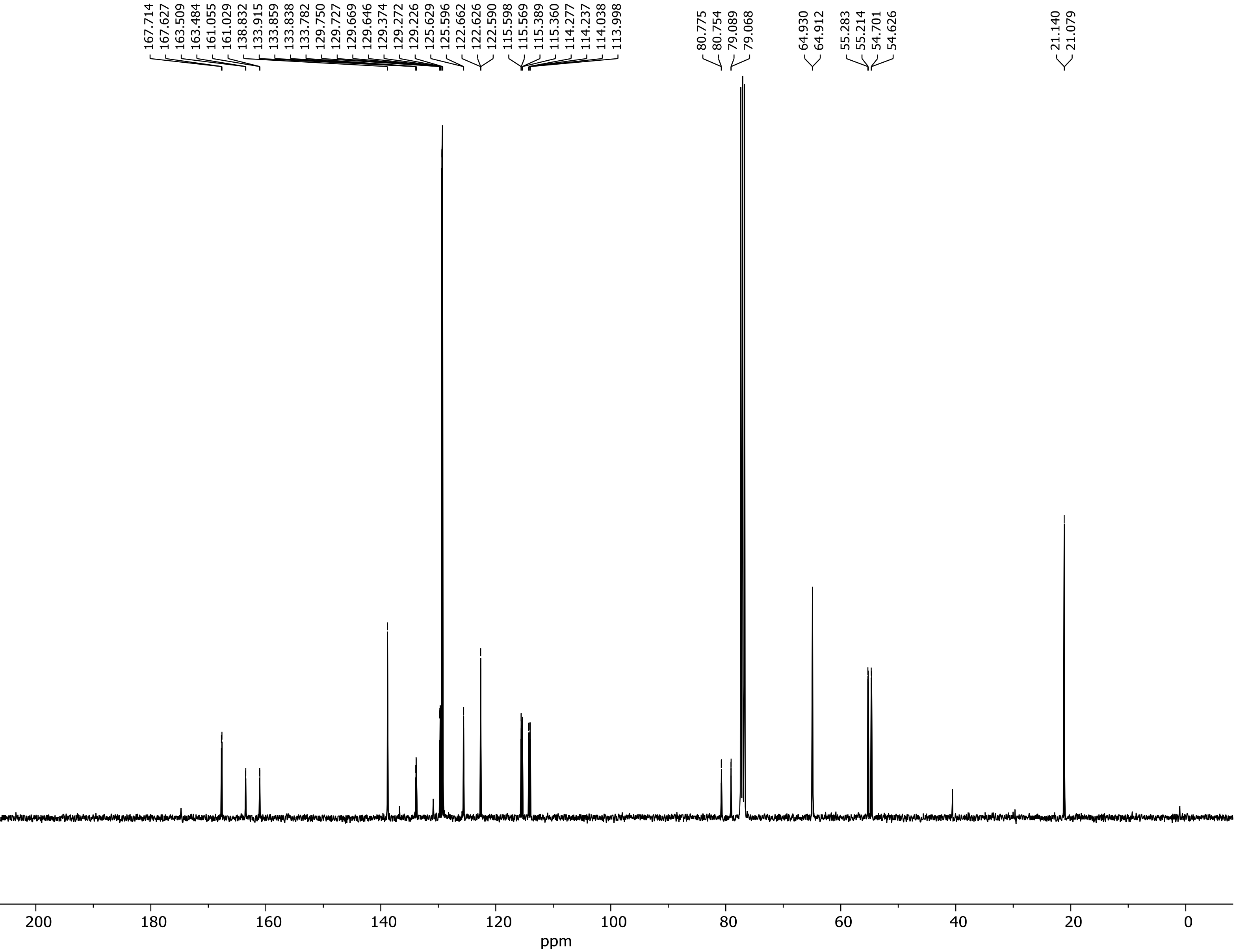

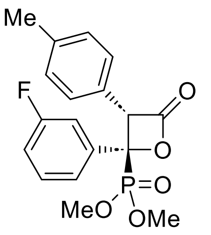

anti-**64**

^{19}F, $CDCl_3$, 376 MHz

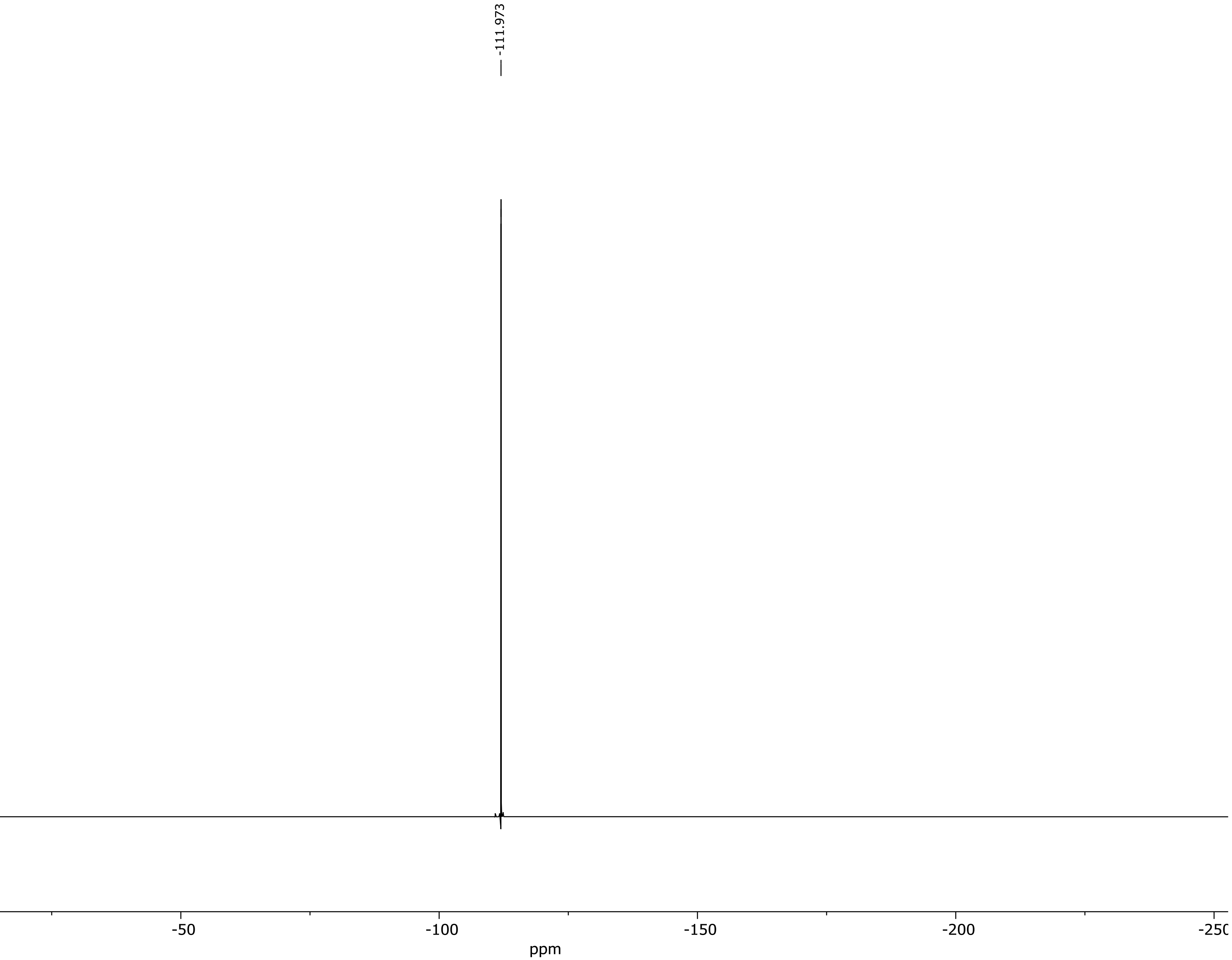

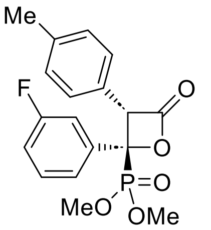

anti-**64**

^{31}P, $CDCl_3$, 162 MHz

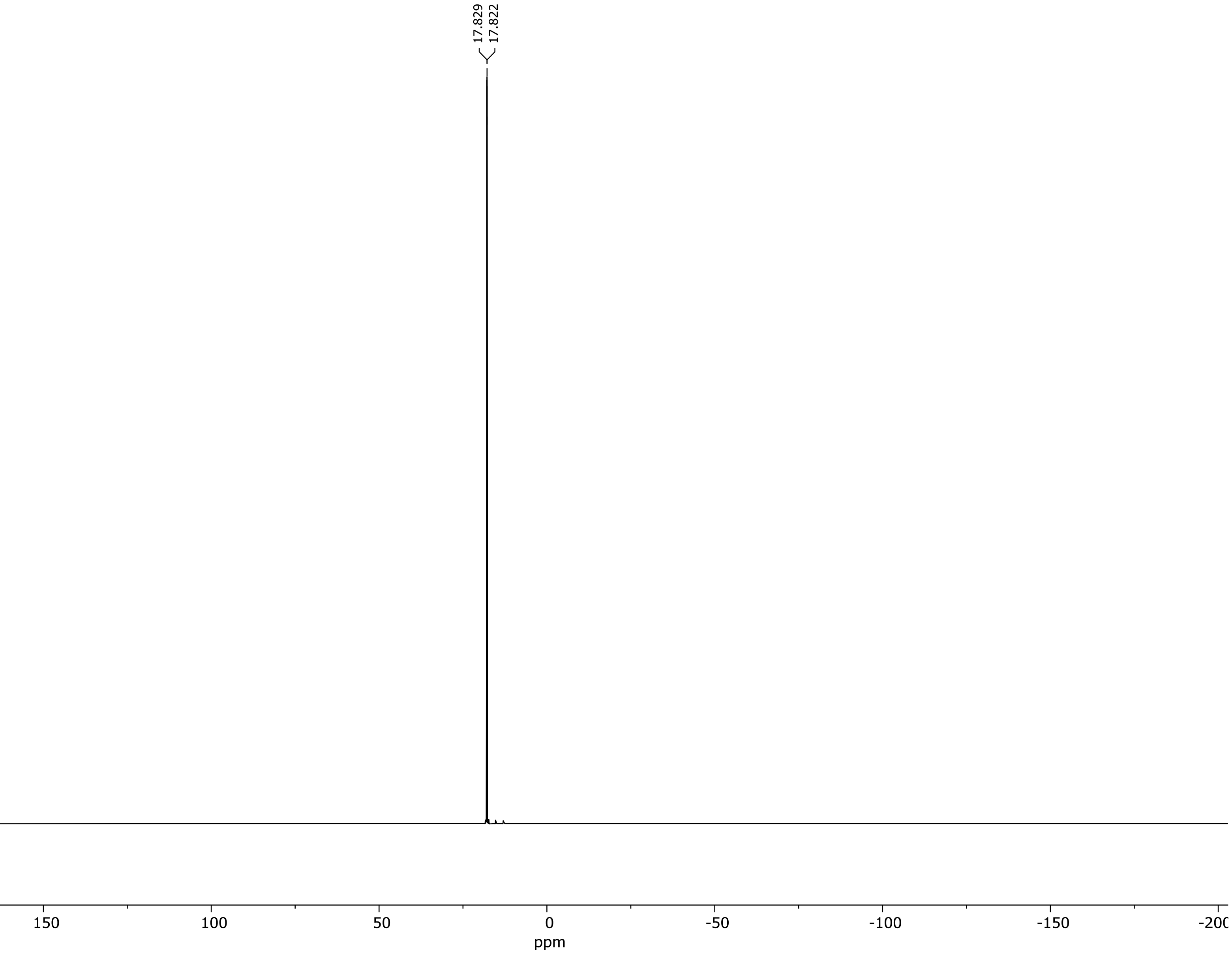

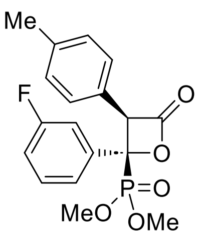

syn-**64**
^{1}H, $CDCl_3$, 400 MHz

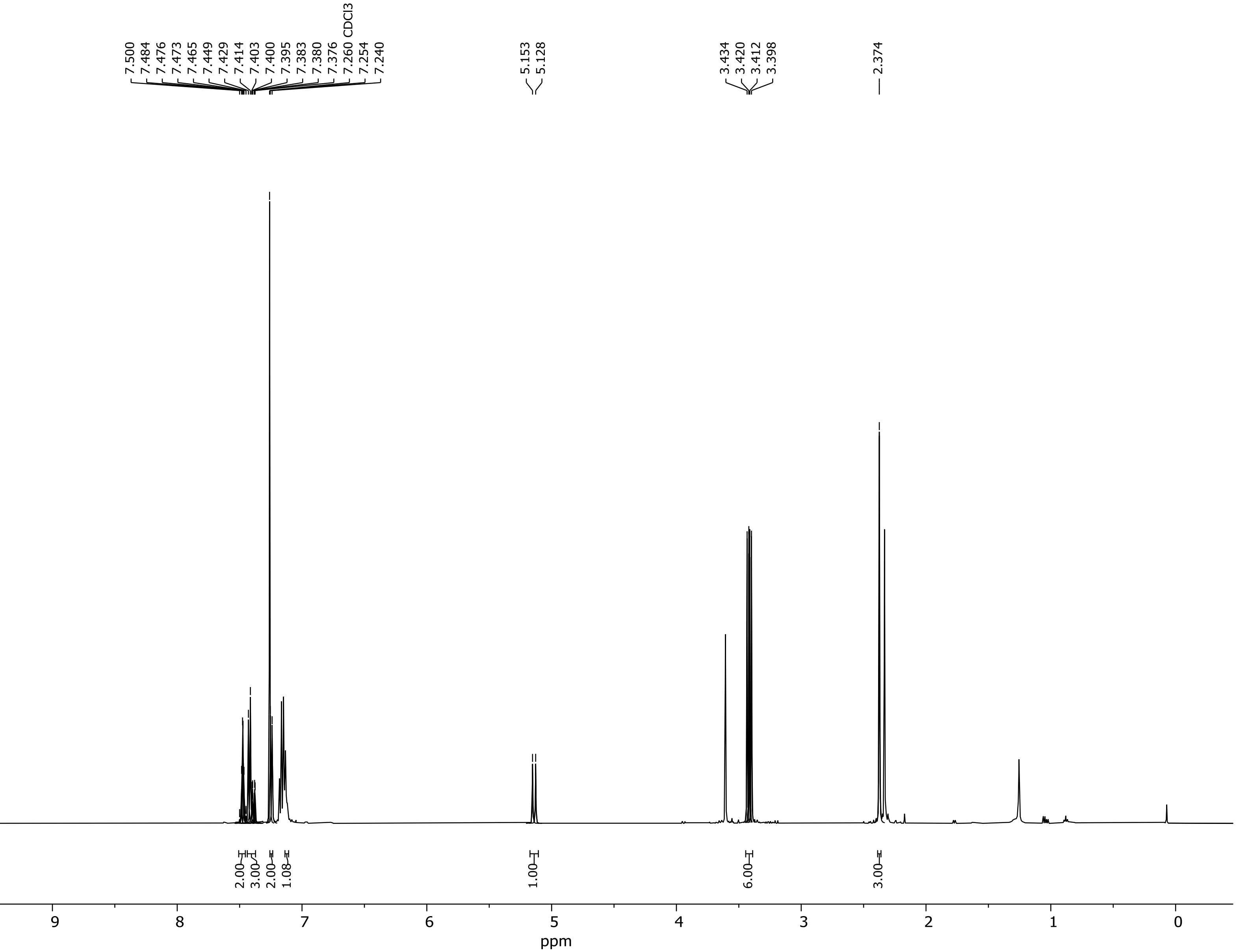

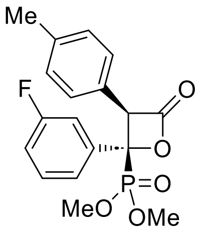

syn-**64**

^{13}C, $CDCl_3$, 101 MHz

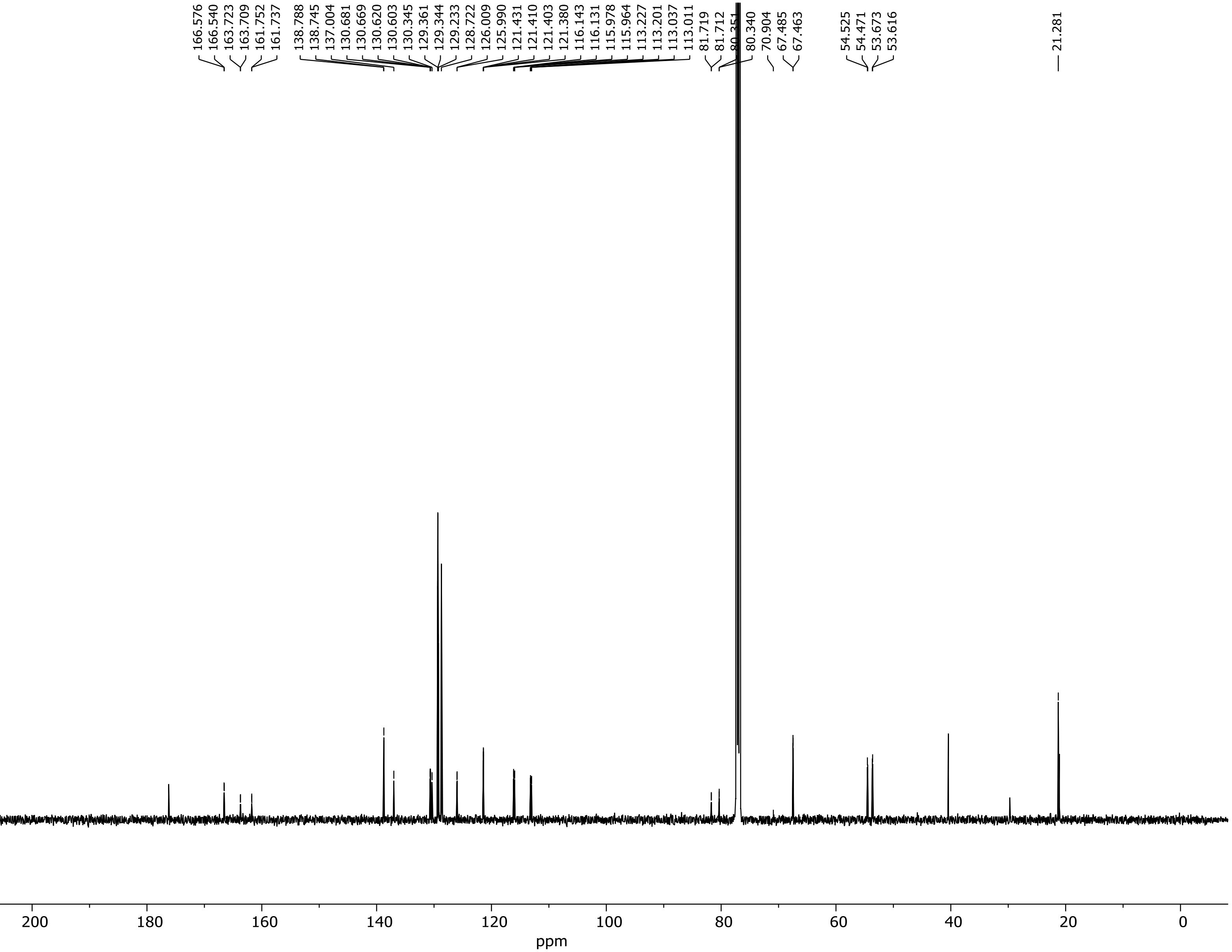

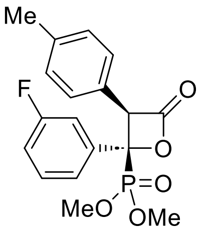

syn-**64**

^{19}F, $CDCl_3$, 376 MHz

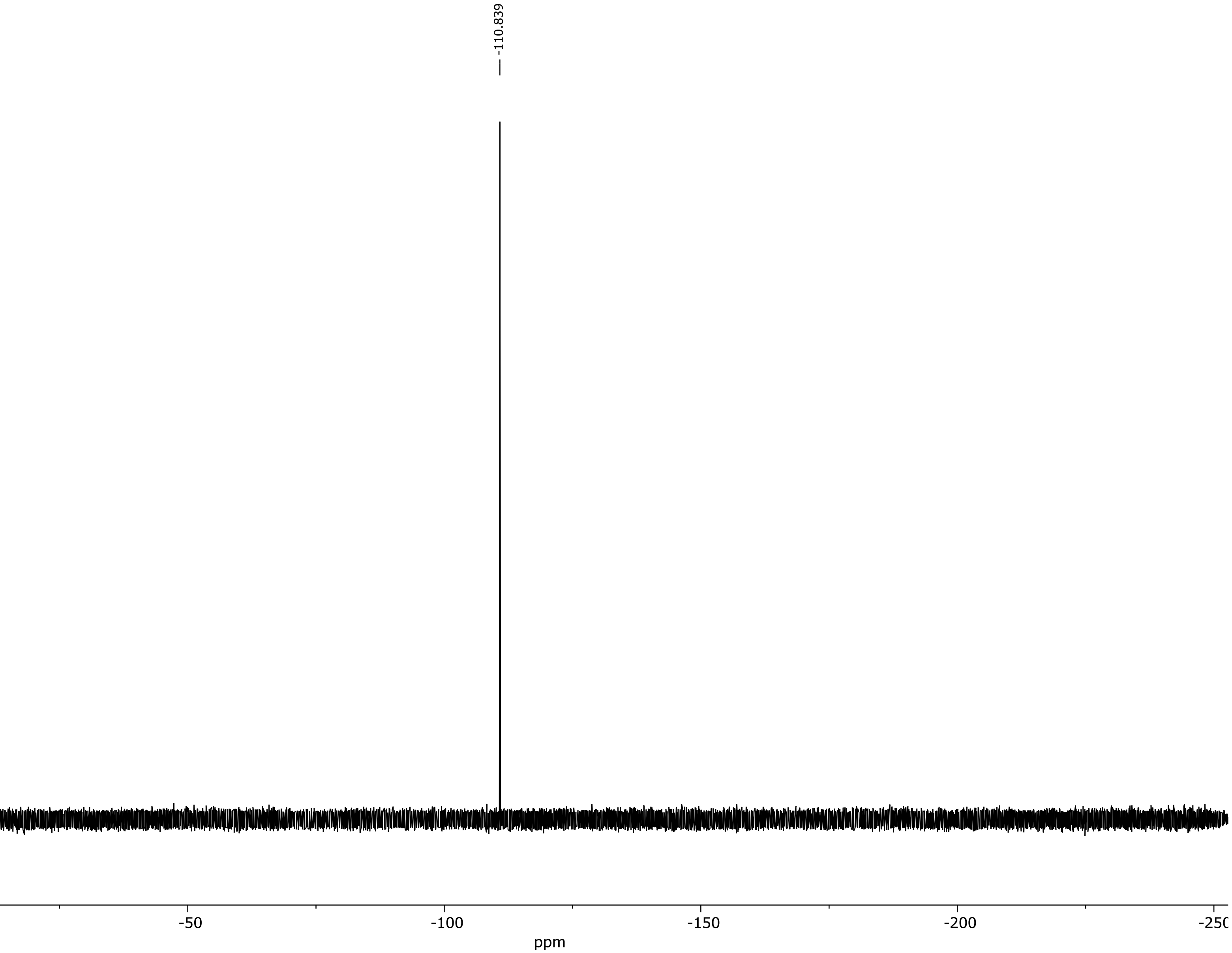

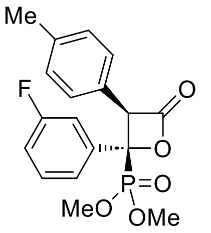

syn-**64**

^{31}P, $CDCl_3$, 162 MHz

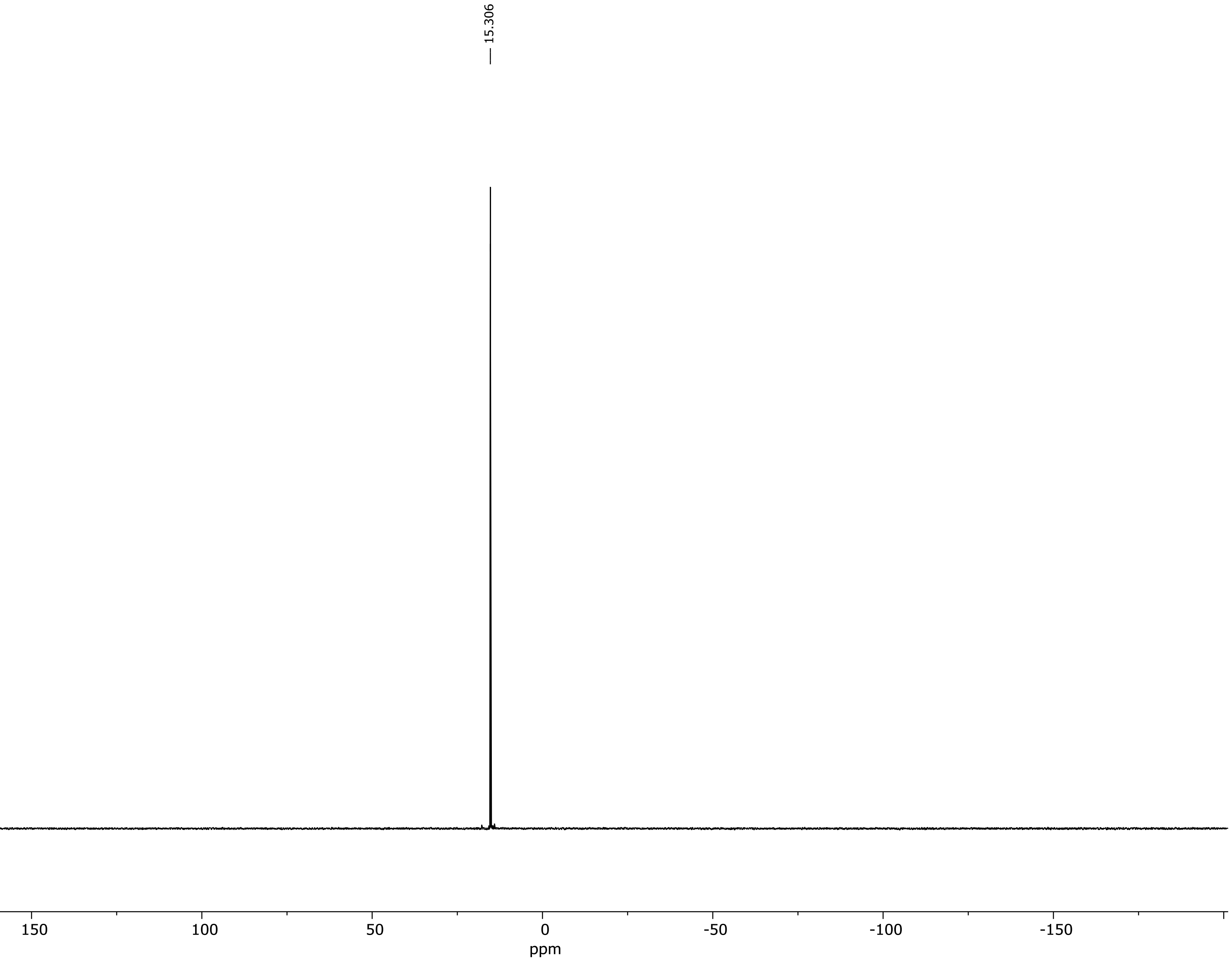

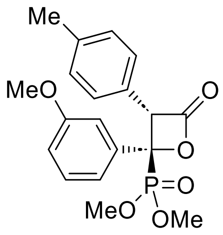

anti-**65**

^1H, $CDCl_3$, 400 MHz

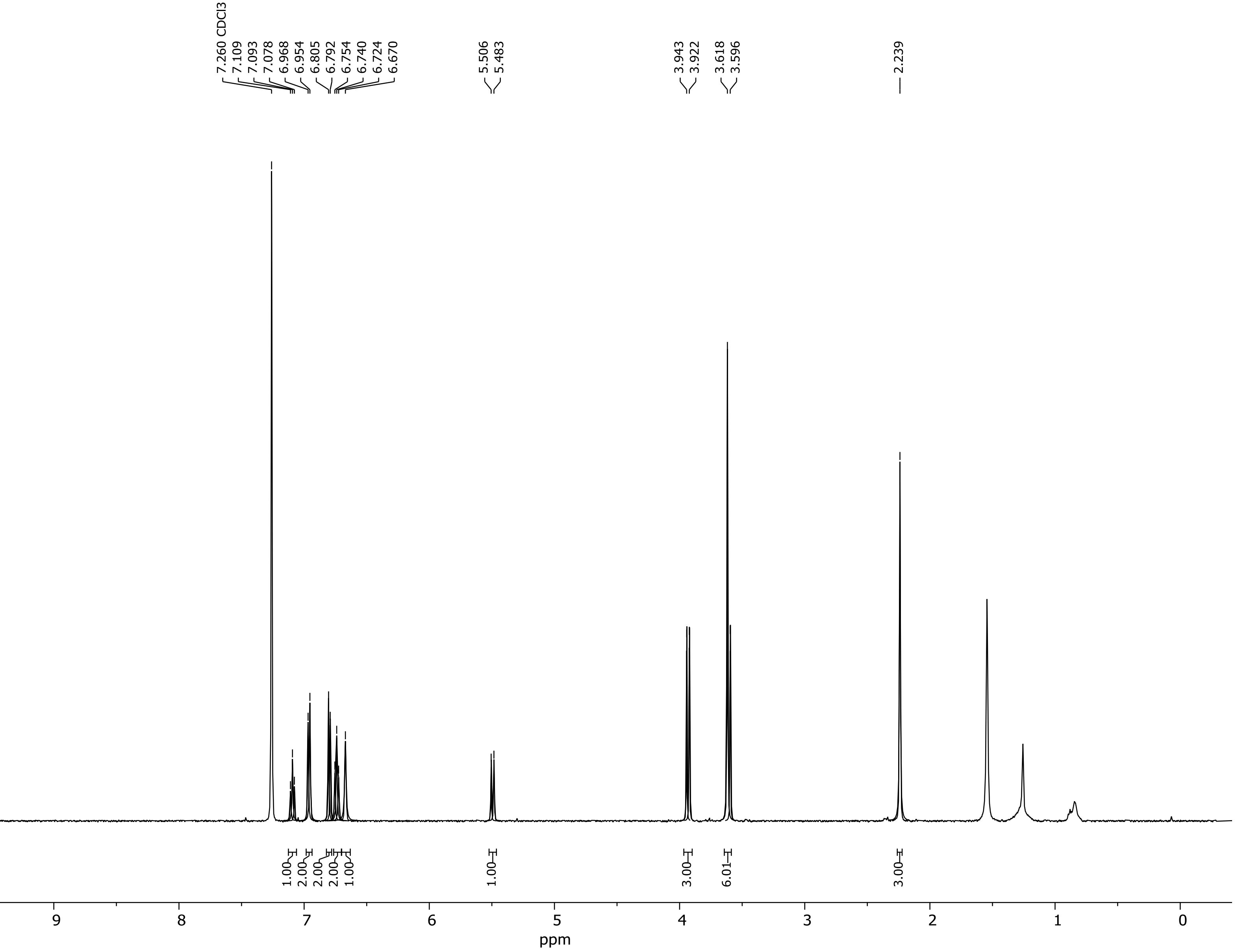

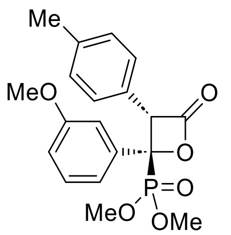

anti-**65**

^{13}C, $CDCl_3$, 101 MHz

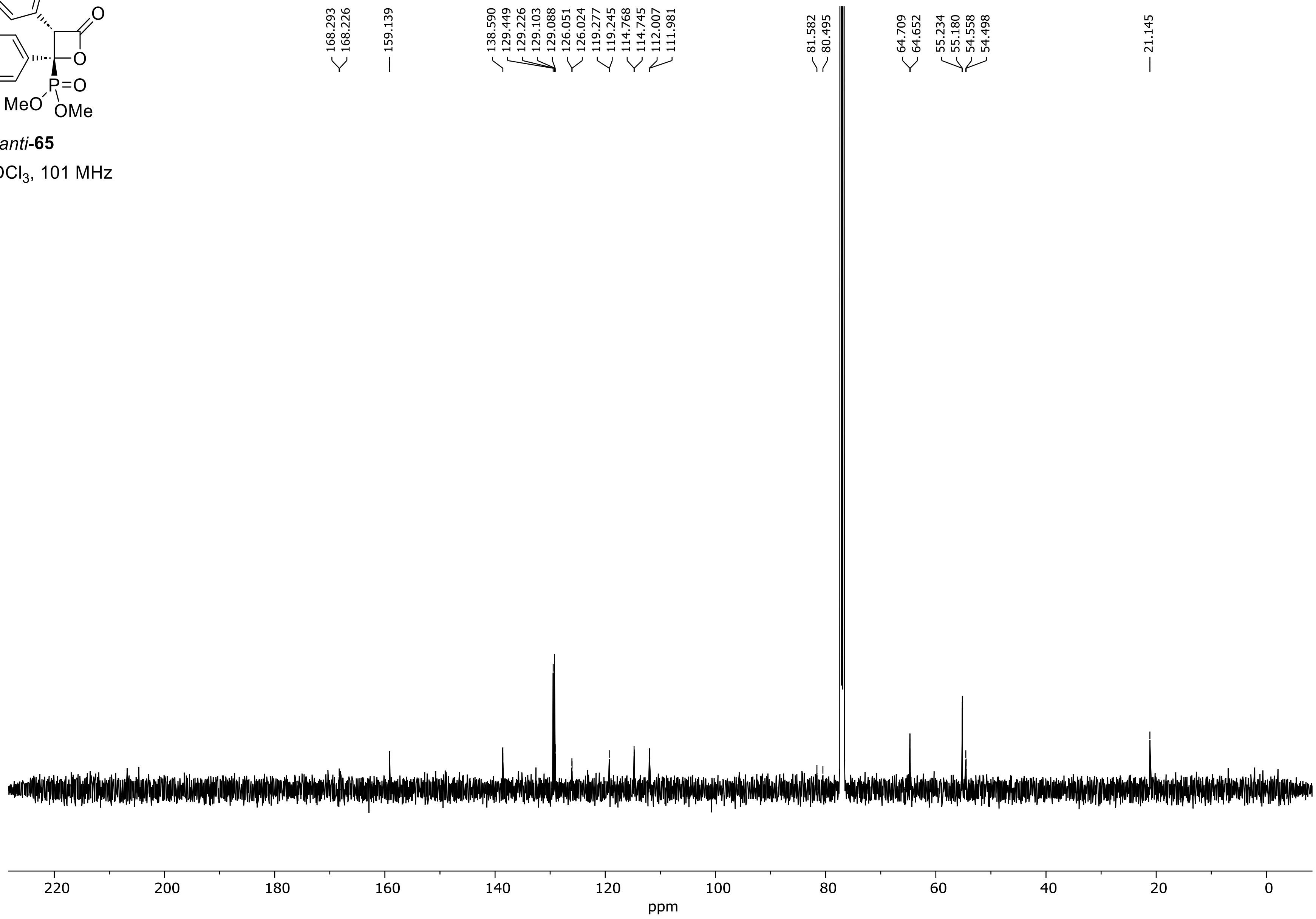

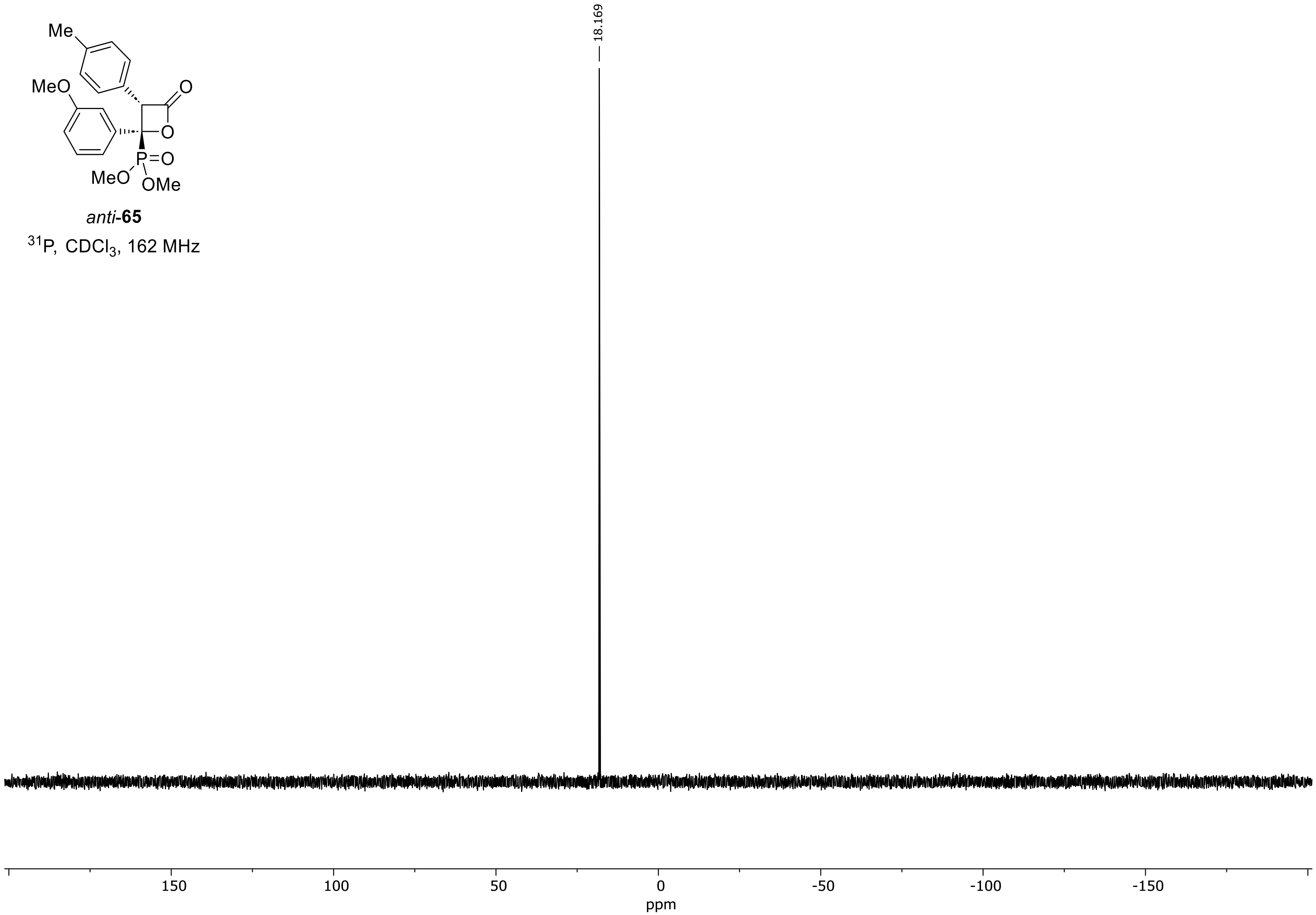
Me
MeO
O
O
P=O
MeO
OMe
anti-65
^{31}P, CDCl$_3$, 162 MHz
18.169
150
100
50
0
-50
-100
-150
ppm

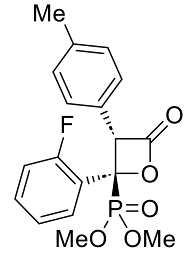

anti-**66**

^{1}H NMR, 400 MHz

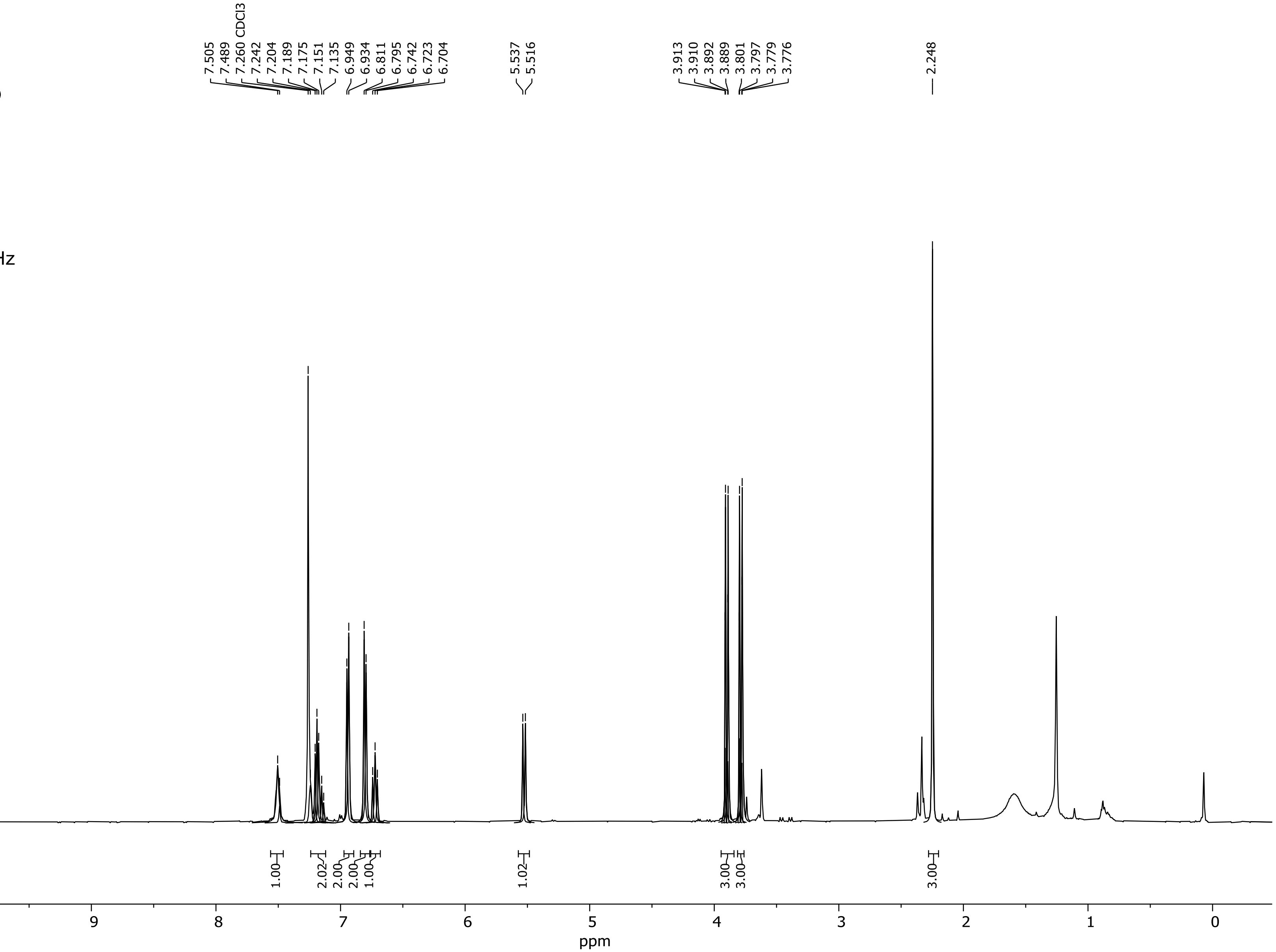

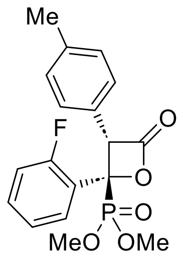

anti-**66**

^{13}C NMR, 102 MHz

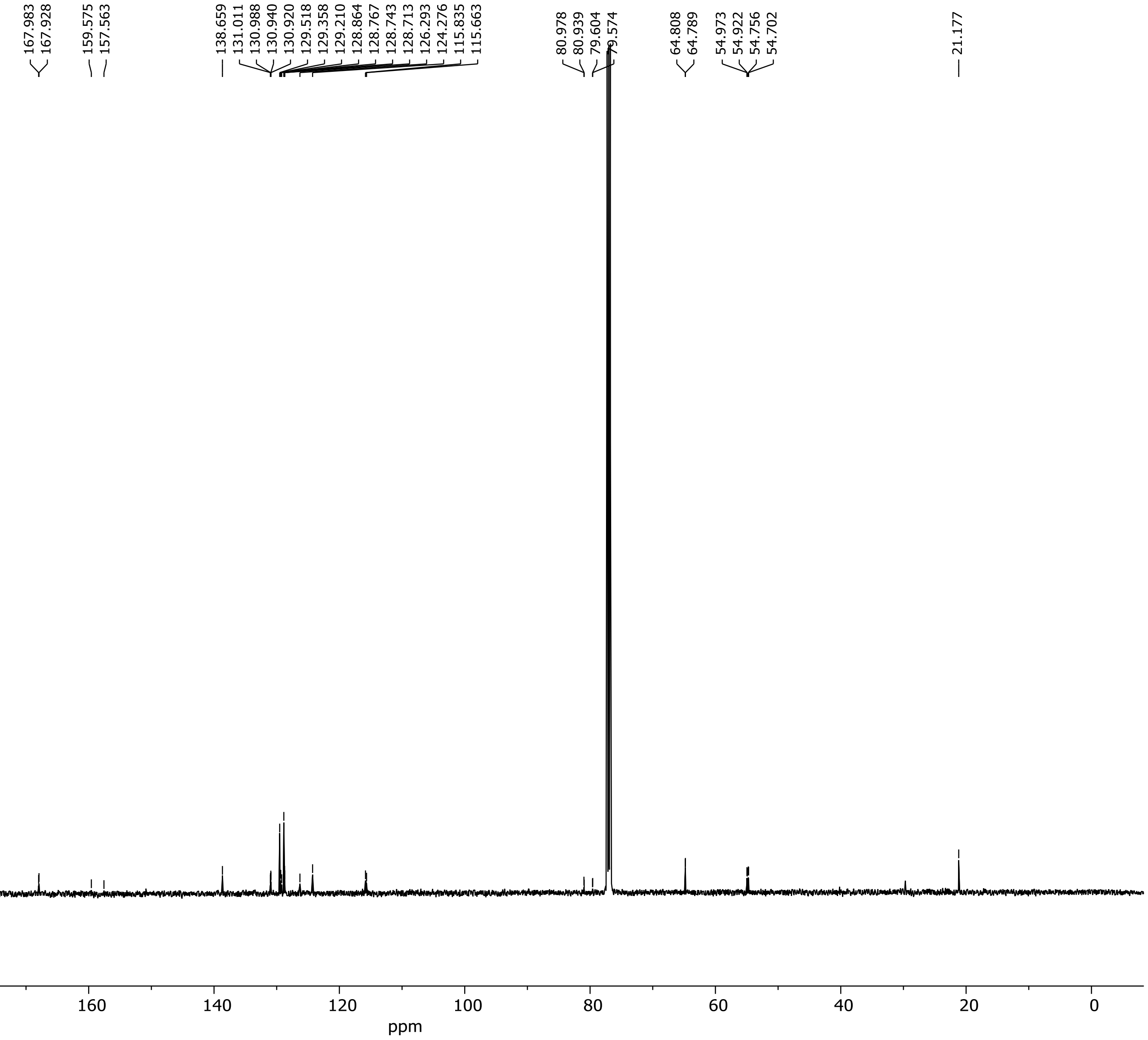

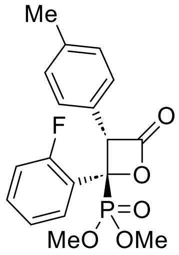

anti-**66**

^{31}P NMR, 162 MHz

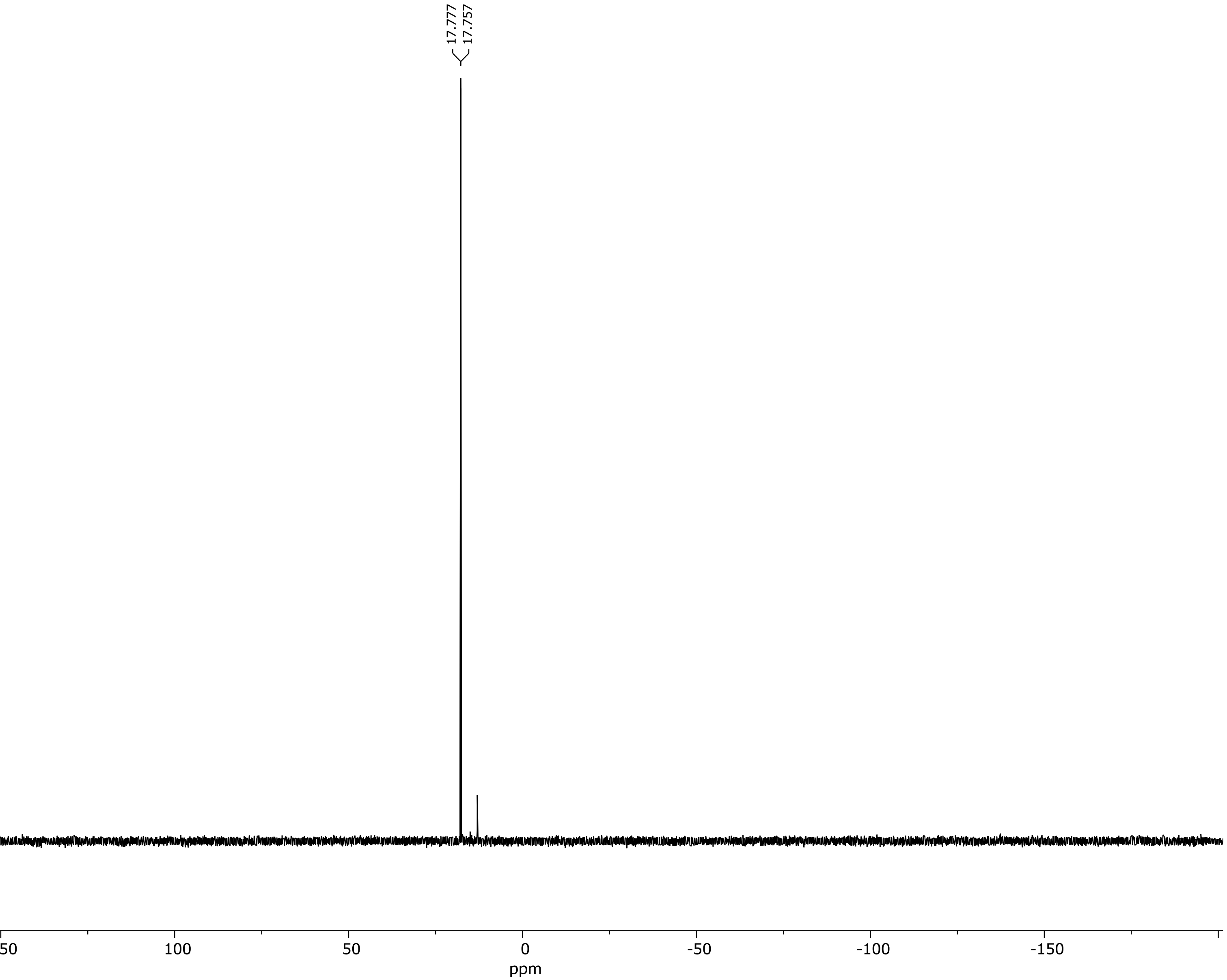

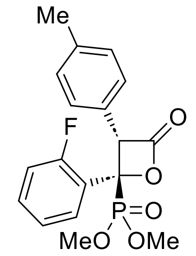

anti-**66**

^{19}F NMR, 376 MHz

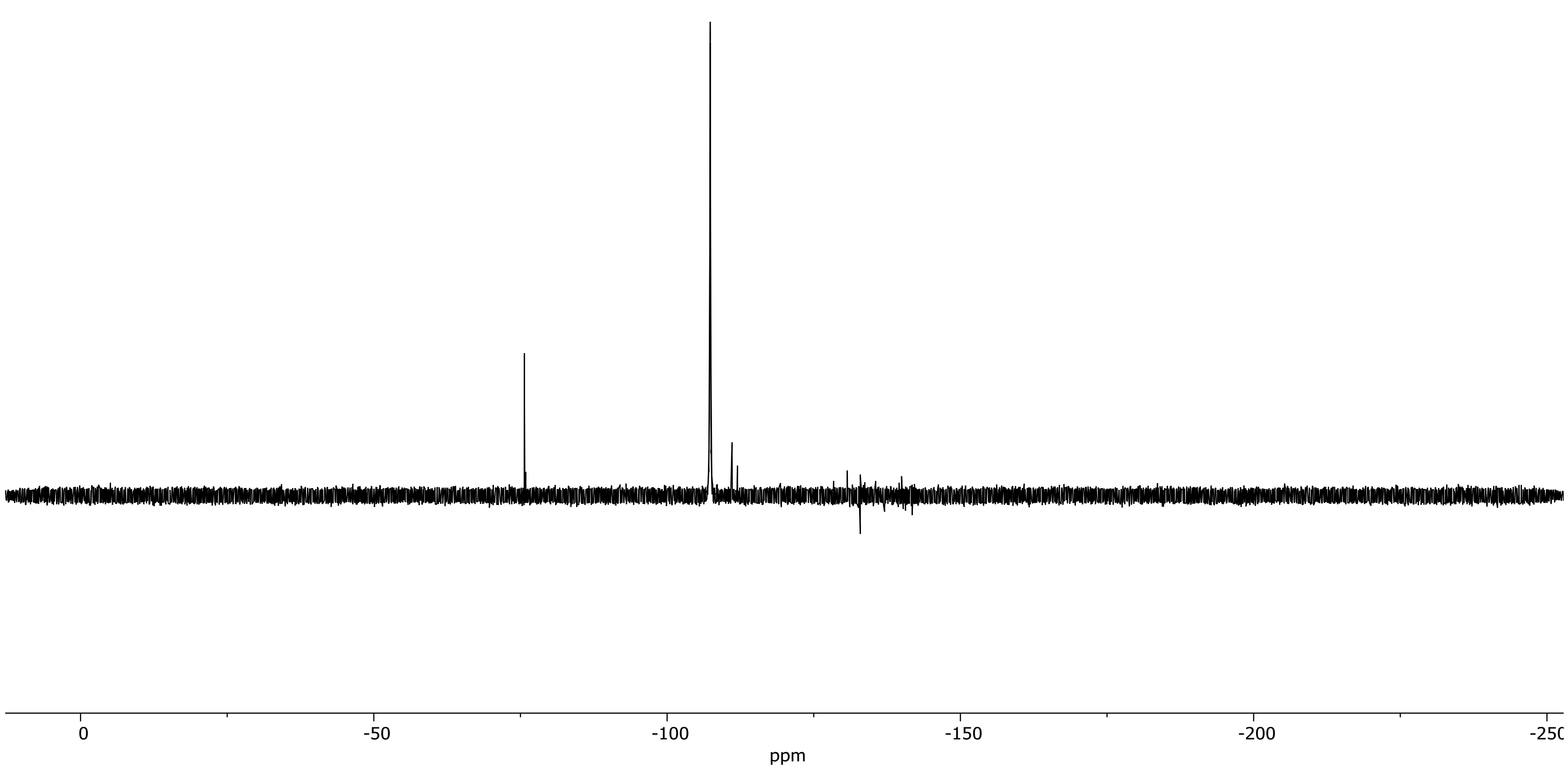

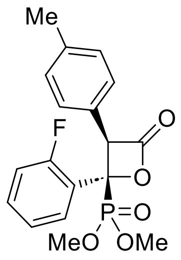

syn-**66**

^{1}H NMR, 400 MHz

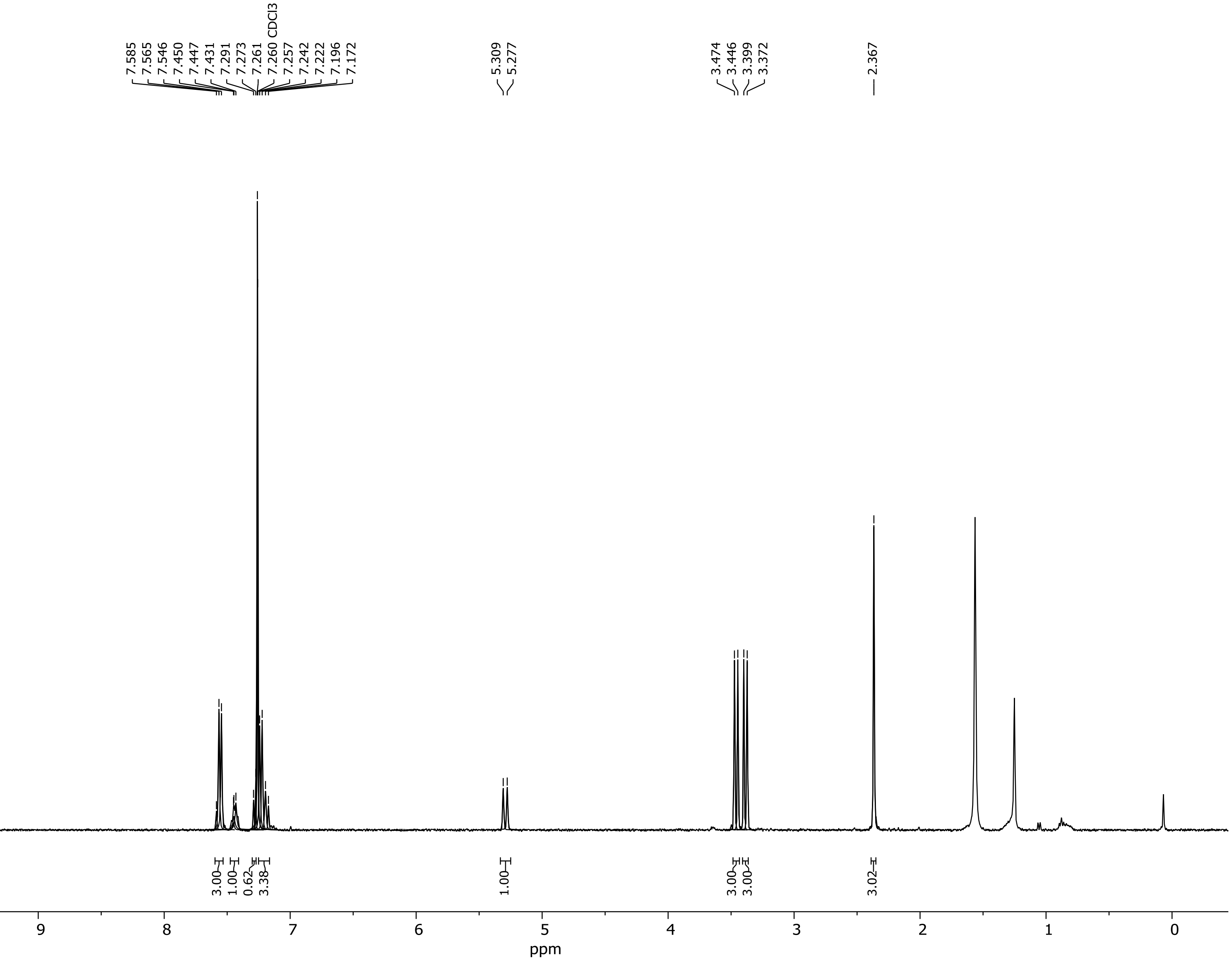

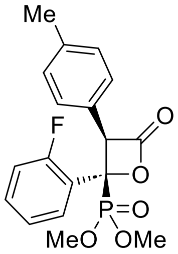

syn-**66**

^{13}C NMR, 102 MHz

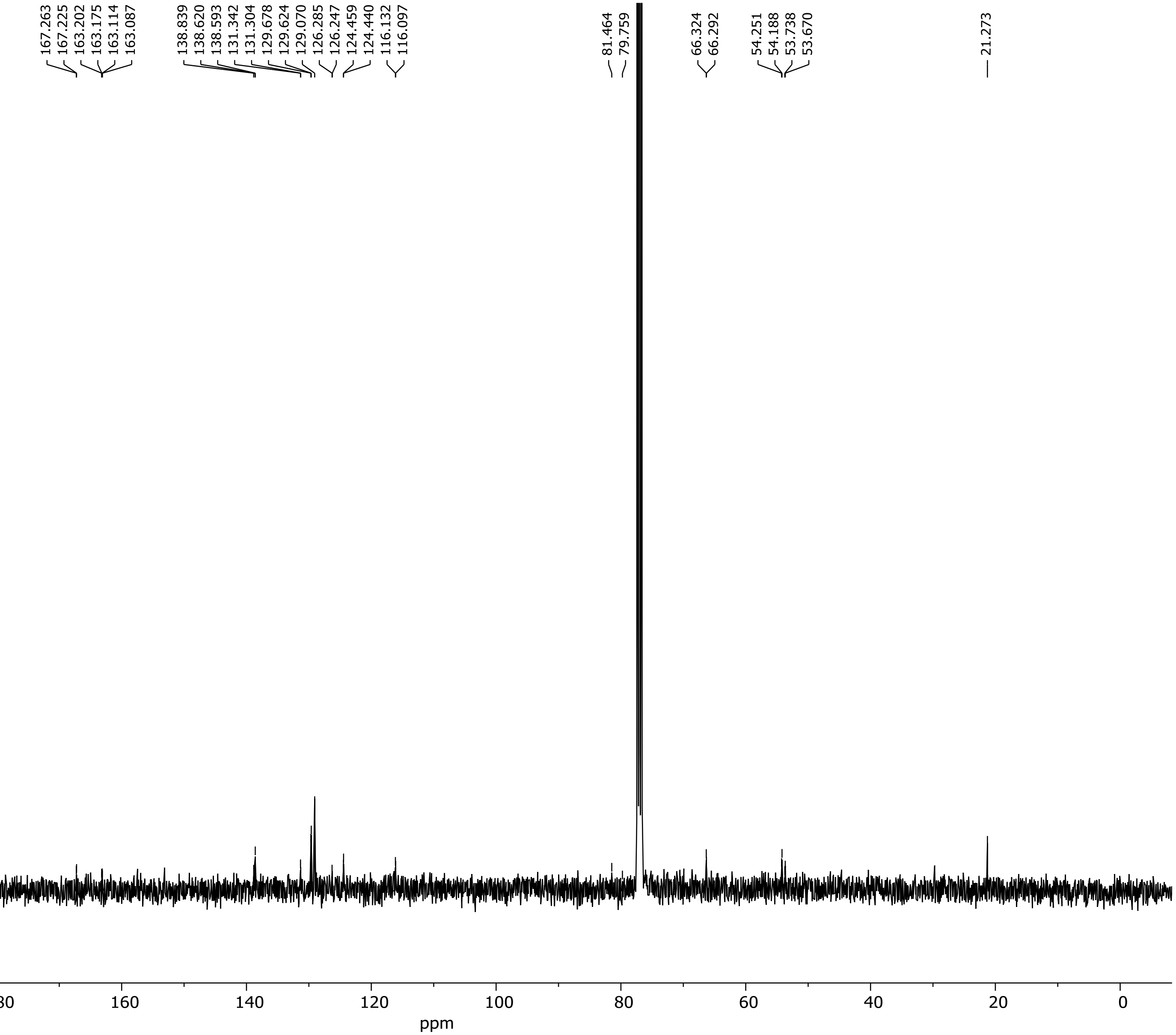

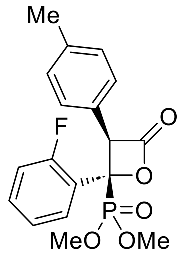

syn-**66**

^{31}P NMR, 162 MHz

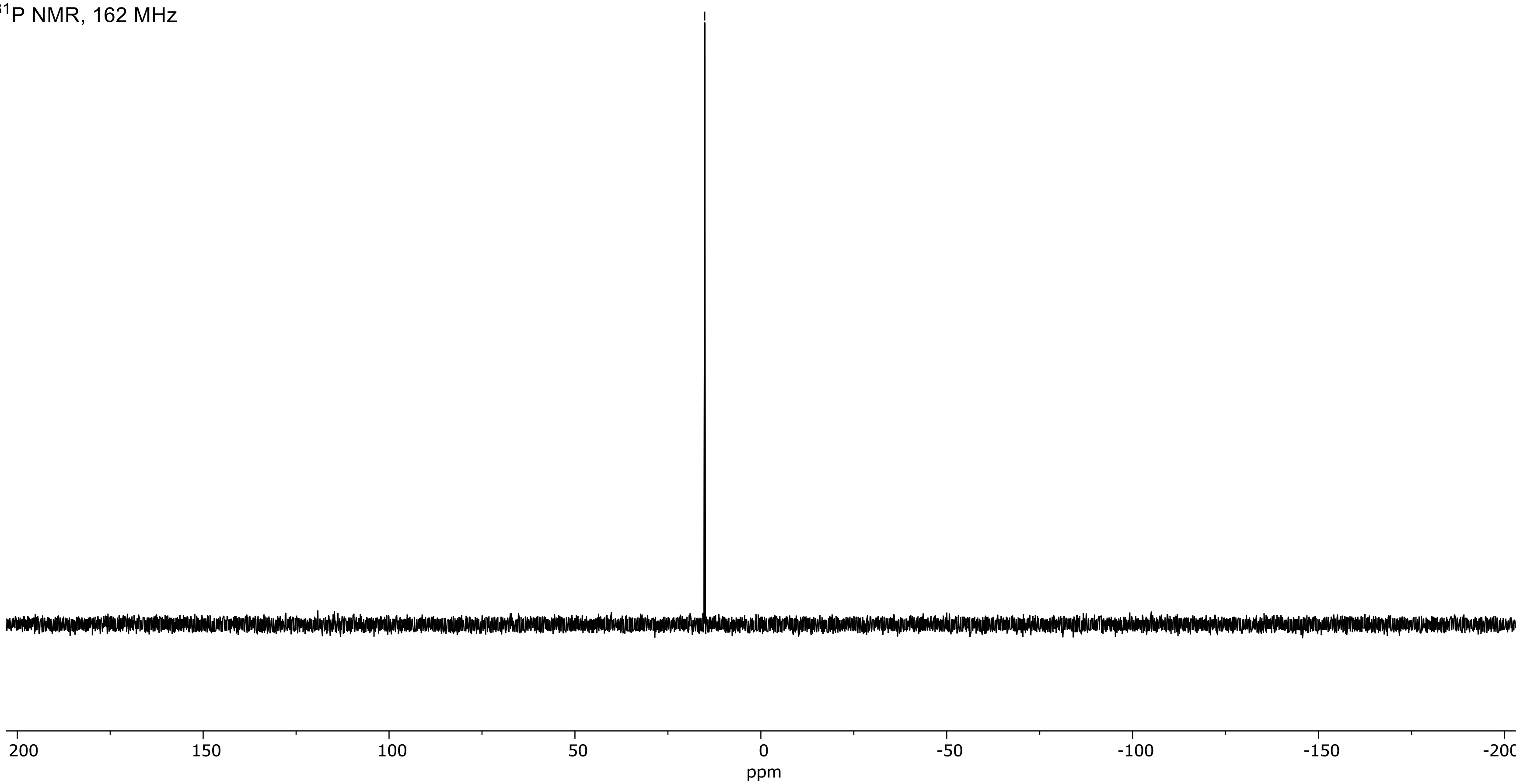

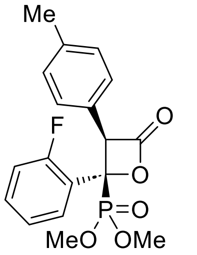

syn-**66**

^{19}F NMR, 376 MHz

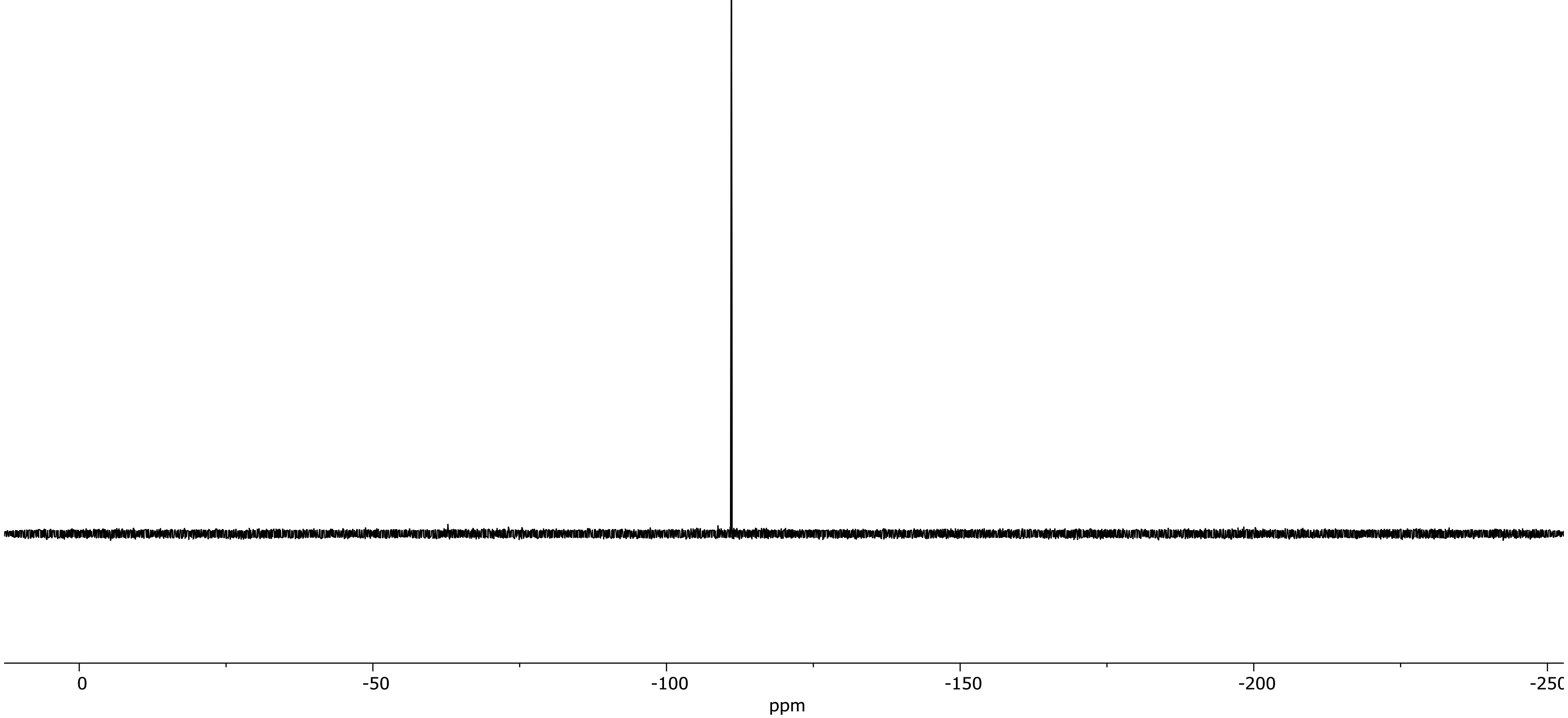

Appendix II. HPLC Traces

HPLC Data for **5**: Chiralcel OJ-H (90:10 hexane:IPA, flow rate 1.0 mLmin^{-1}, 211 nm, 30 ℃), t_R(2*S*,3*S*): 18.8 min, t_R(2*R*,3*R*): 38.6 min, >99:1 er.

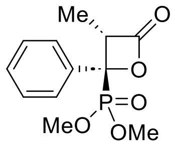

5

PDA Ch1 211nm

Peak#	Ret. Time	Area%
1	18.807	50.440
2	38.614	49.560
Total		100.000

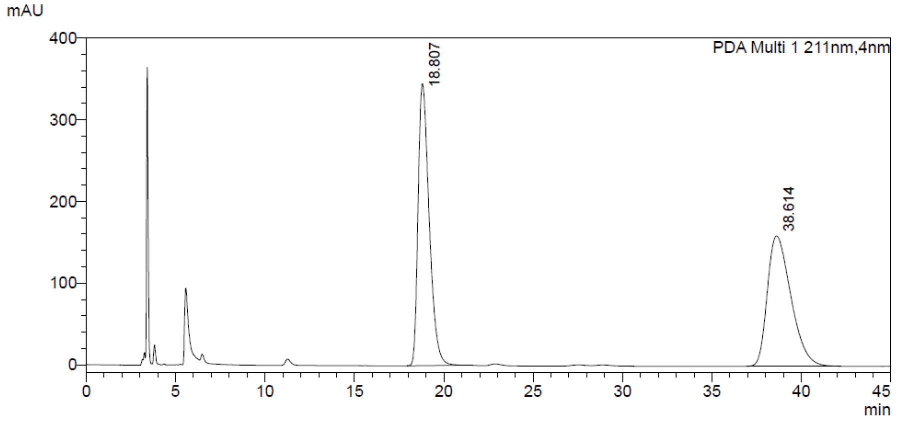

PDA Ch1 211nm

Peak#	Ret. Time	Area%
1	39.145	100.000
Total		100.000

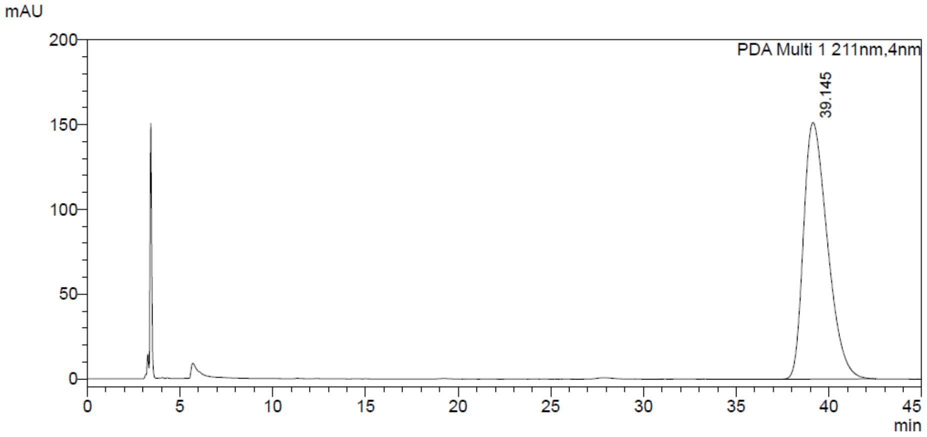

HPLC Data for **11**: Chiralcel OJ-H (95:5 hexane:IPA, flow rate 1.0 mLmin^{-1}, 211 nm, 30 ℃), t_R(2*S*,3*S*): 9.0 min, t_R(2*R*,3*R*): 17.8 min, >99:1 er.

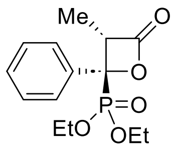

11

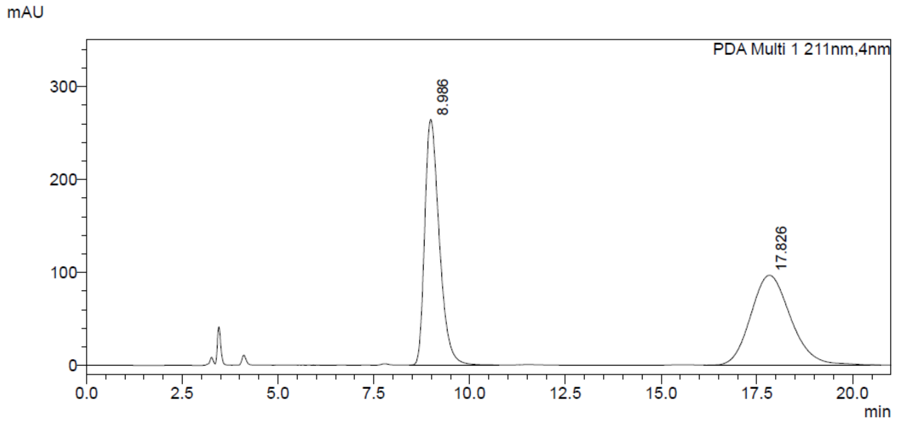

<Peak Table>

PDA Ch1 211nm

Peak#	Ret. Time	Area%
1	8.986	49.857
2	17.826	50.143
Total		100.000

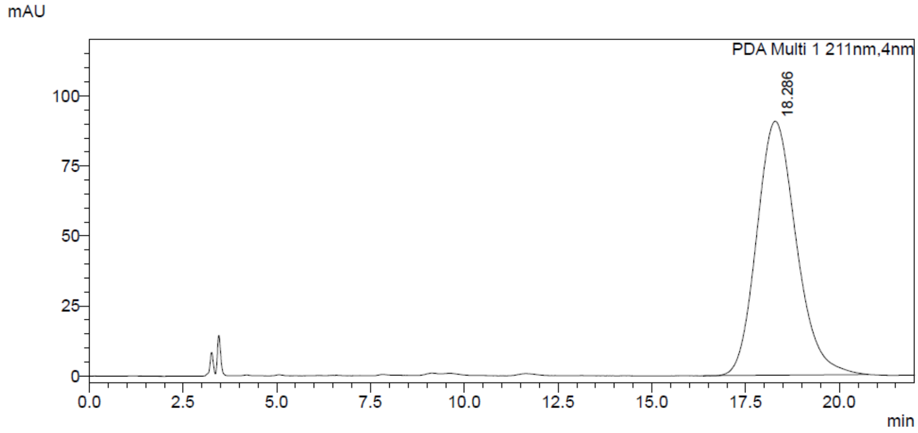

<Peak Table>

PDA Ch1 211nm

Peak#	Ret. Time	Area%
1	18.286	100.000
Total		100.000

HPLC Data for **12**: Chiralcel OJ-H (98:2 hexane:IPA, flow rate 1.0 mLmin^{-1}, 211 nm, 30 ℃), t_R(2*S*,3*S*): 7.4 min, t_R(2*R*,3*R*): 11.3 min, >99:1 er.

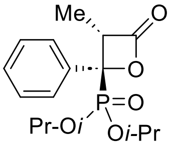

12

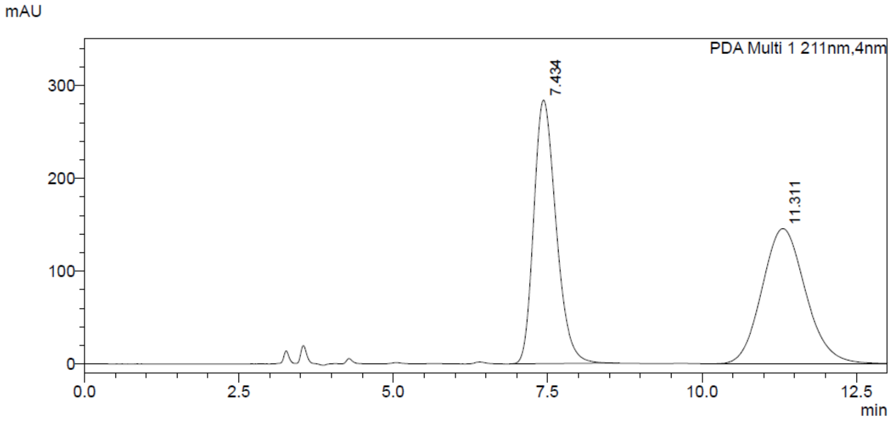

<Peak Table>

PDA Ch1 211nm

Peak#	Ret. Time	Area%
1	7.434	49.831
2	11.311	50.169
Total		100.000

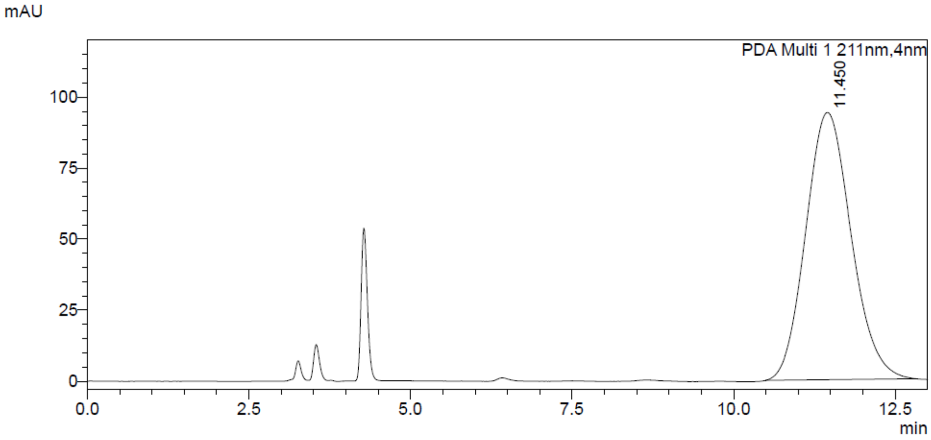

<Peak Table>

PDA Ch1 211nm

Peak#	Ret. Time	Area%
1	11.450	100.000
Total		100.000

HPLC Data for **13**: Chiralcel OJ-H (90:10 hexane:IPA, flow rate 1.0 mLmin^{-1}, 211 nm, 30 ℃), t_R(2*R*,3*R*): 33.1 min, t_R(2*S*,3*S*): 38.5 min, 87:13 er.

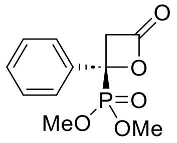

13

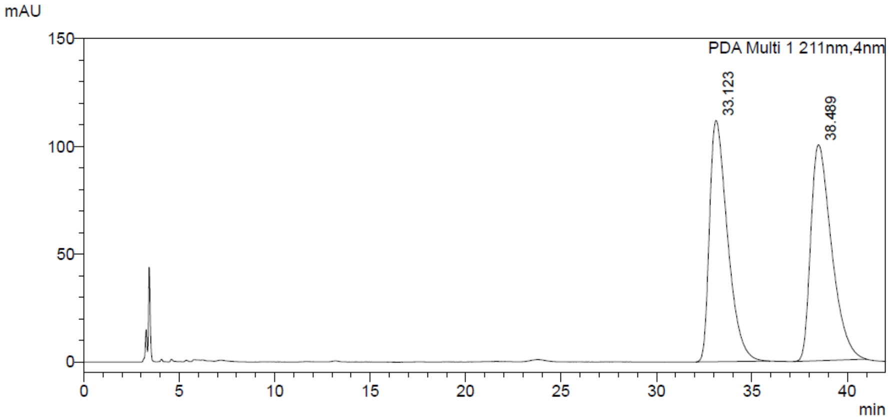

<Peak Table>

PDA Ch1 211nm

Peak#	Ret. Time	Area%
1	33.123	49.130
2	38.489	50.870
Total		100.000

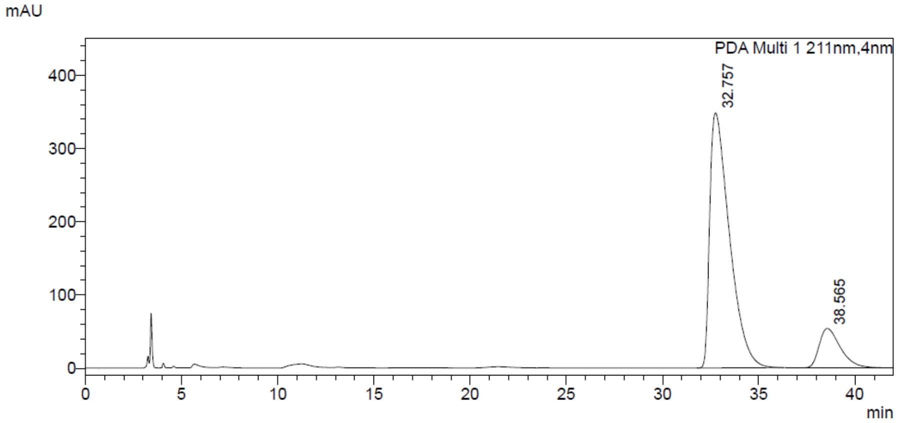

<Peak Table>

PDA Ch1 211nm

Peak#	Ret. Time	Area%
1	32.757	85.754
2	38.565	14.246
Total		100.000

HPLC Data for **14**: Chiralpak AS-H (95:5 hexane:IPA, flow rate 1.0 mLmin^{-1}, 211 nm, 30 ℃), t_R(2*R*,3*R*): 8.7 min, t_R(2*S*,3*S*): 10.8 min, 88:12 er.

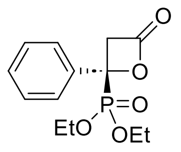

14

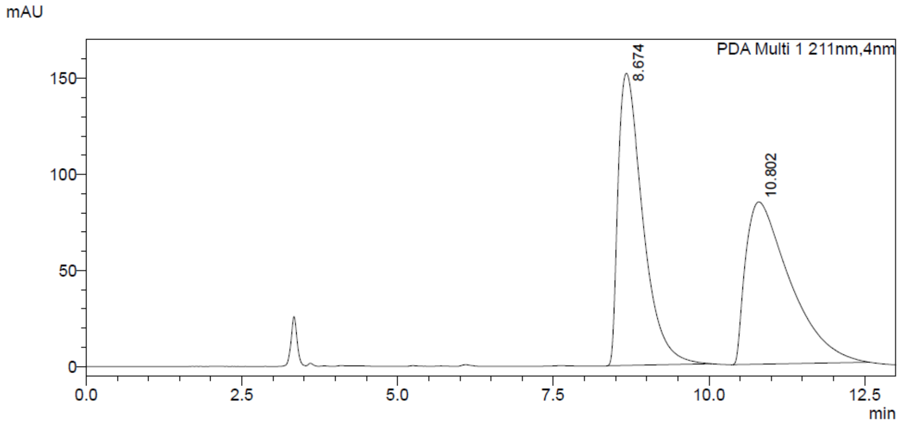

<Peak Table>

PDA Ch1 211nm

Peak#	Ret. Time	Area%
1	8.674	50.034
2	10.802	49.966
Total		100.000

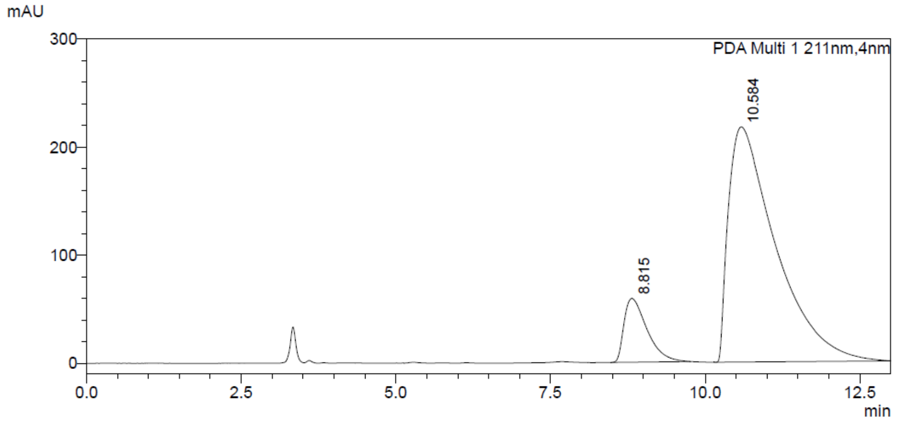

<Peak Table>

PDA Ch1 211nm

Peak#	Ret. Time	Area%
1	8.815	11.653
2	10.584	88.347
Total		100.000

HPLC Data for **15**: Chiralcel OD-H (95:5 hexane:IPA, flow rate 1.0 mLmin^{-1}, 211 nm, 30 ℃), t_R(2*S*,3*S*): 7.0 min, t_R(2*R*,3*R*): 7.7 min, 89:11 er.

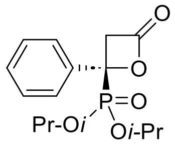

15

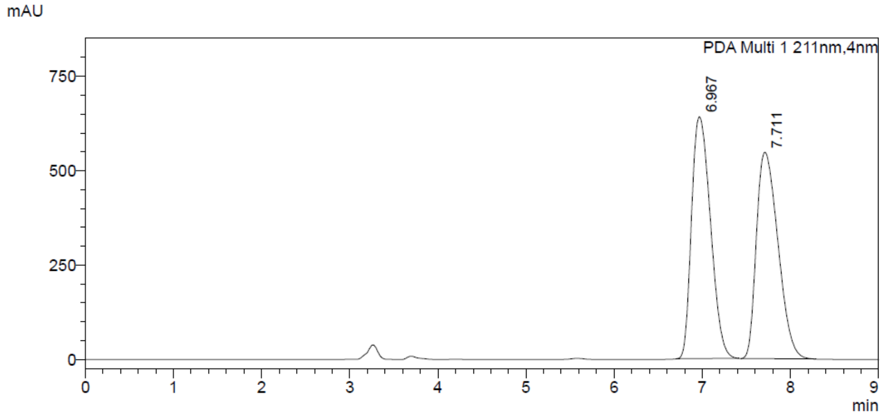

<Peak Table>

PDA Ch1 211nm

Peak#	Ret. Time	Area%
1	6.967	50.698
2	7.711	49.302
Total		100.000

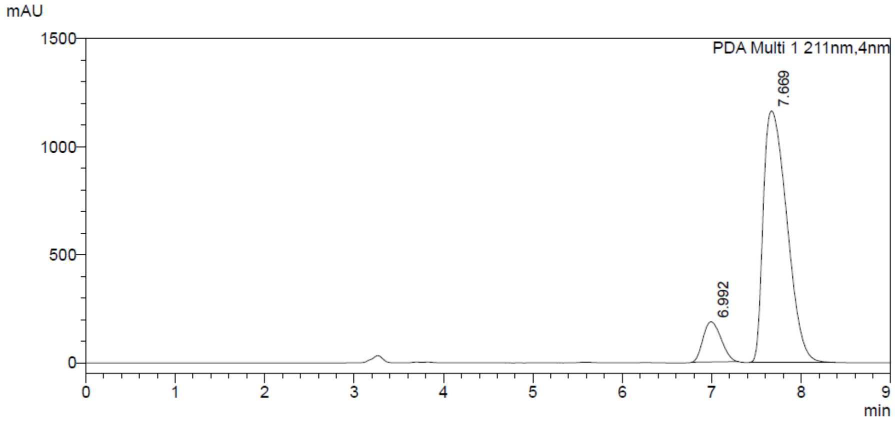

<Peak Table>

PDA Ch1 211nm

Peak#	Ret. Time	Area%
1	6.992	10.919
2	7.669	89.081
Total		100.000

HPLC Data for **16**: Chiralcel OJ-H (90:10 hexane:IPA, flow rate 1.0 mLmin^{-1}, 211 nm, 30 ℃), t_R(2*S*,3*S*): 8.8 min, t_R(2*R*,3*R*): 10.0 min, >99:1 er.

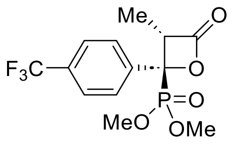

16

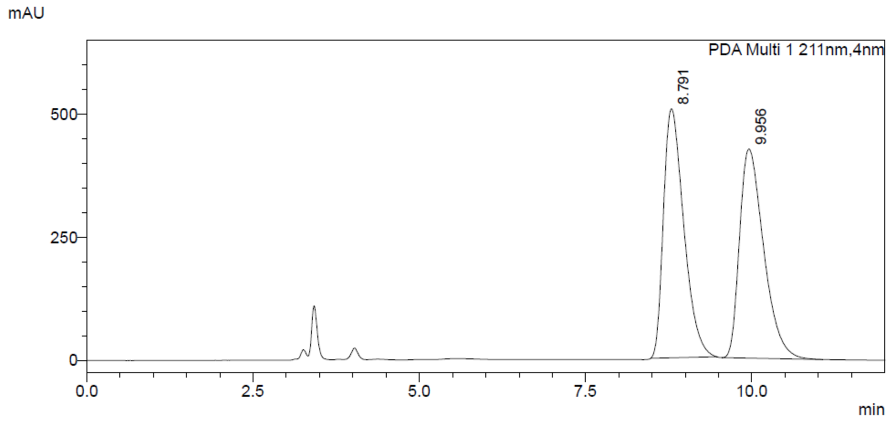

<Peak Table>

PDA Ch1 211nm

Peak#	Ret. Time	Area%
1	8.791	50.045
2	9.956	49.955
Total		100.000

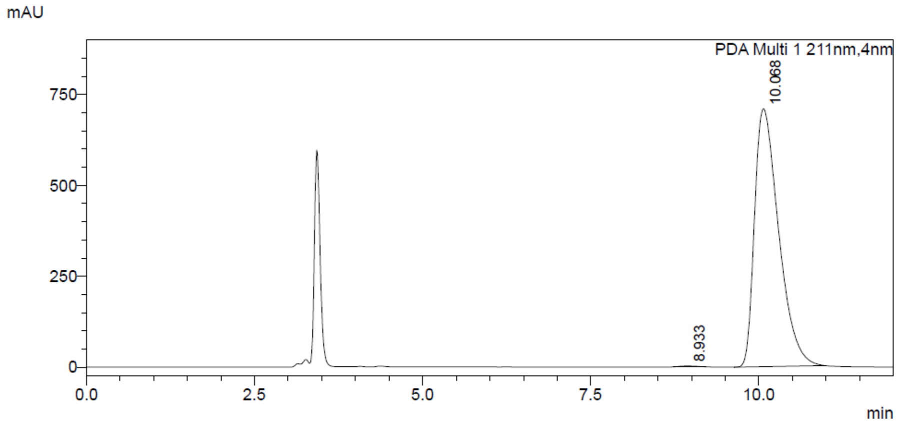

<Peak Table>

PDA Ch1 211nm

Peak#	Ret. Time	Area%
1	8.933	0.260
2	10.068	99.740
Total		100.000

HPLC Data for **17**: Chiralcel OJ-H (90:10 hexane:IPA, flow rate 1.0 mLmin^{-1}, 211 nm, 30 ℃), t_R(2*S*,3*S*): 8.5 min, t_R(2*R*,3*R*): 11.0 min, >99:1 er.

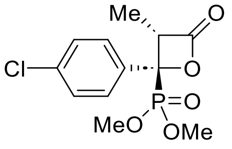

17

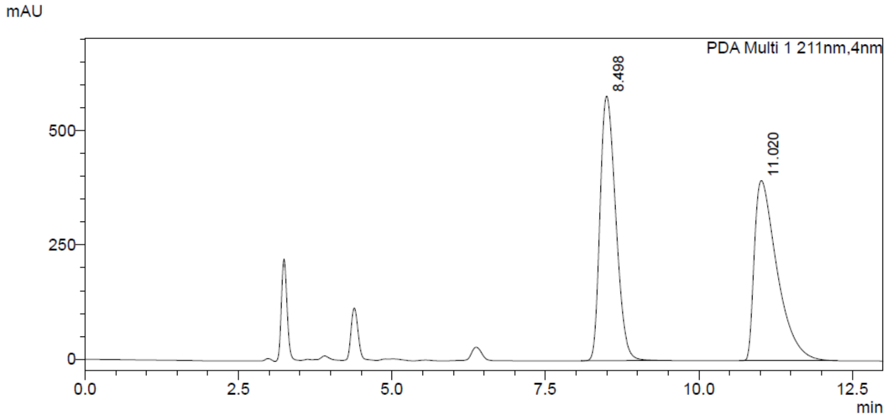

<Peak Table>

PDA Ch1 211nm

Peak#	Ret. Time	Area%
1	8.498	50.401
2	11.020	49.599
Total		100.000

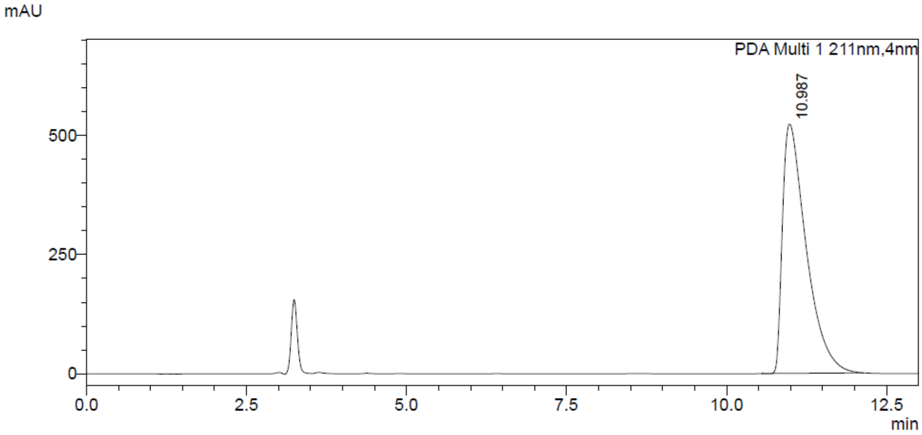

<Peak Table>

PDA Ch1 211nm

Peak#	Ret. Time	Area%
1	10.987	100.000
Total		100.000

HPLC Data for **18**: Chiralcel OD-H (90:10 hexane:IPA, flow rate 1.0 mLmin^{-1}, 211 nm, 30 ℃), t_R(2*S*,3*S*): 13.6 min, t_R(2*R*,3*R*): 16.0 min, >99:1 er.

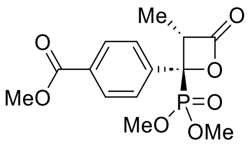

18

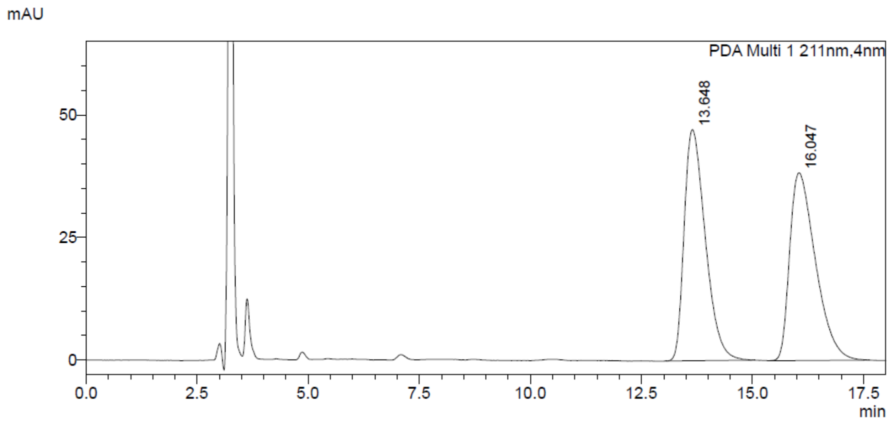

<Peak Table>

PDA Ch1 211nm

Peak#	Ret. Time	Area%
1	13.648	50.280
2	16.047	49.720
Total		100.000

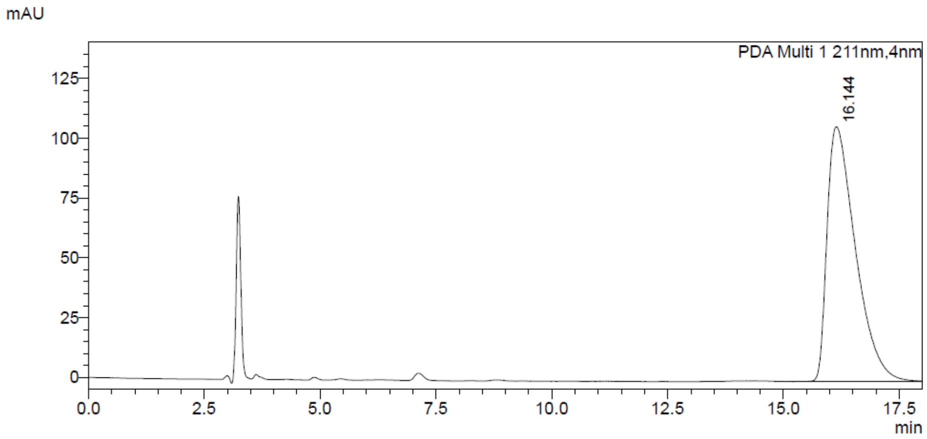

<Peak Table>

PDA Ch1 211nm

Peak#	Ret. Time	Area%
1	16.144	100.000
Total		100.000

HPLC Data for **19**: Chiralcel OJ-H (90:10 hexane:IPA, flow rate 1.0 mLmin^{-1}, 211 nm, 30 ℃), t_R(2*S*,3*S*): 15.0 min, t_R(2*R*,3*R*): 23.5 min, >99:1 er.

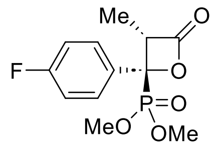

19

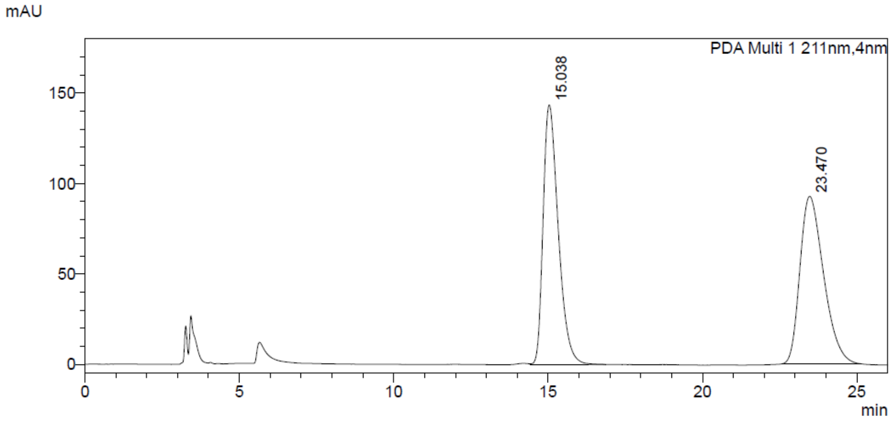

<Peak Table>

PDA Ch1 211nm

Peak#	Ret. Time	Area%
1	15.038	49.686
2	23.470	50.314
Total		100.000

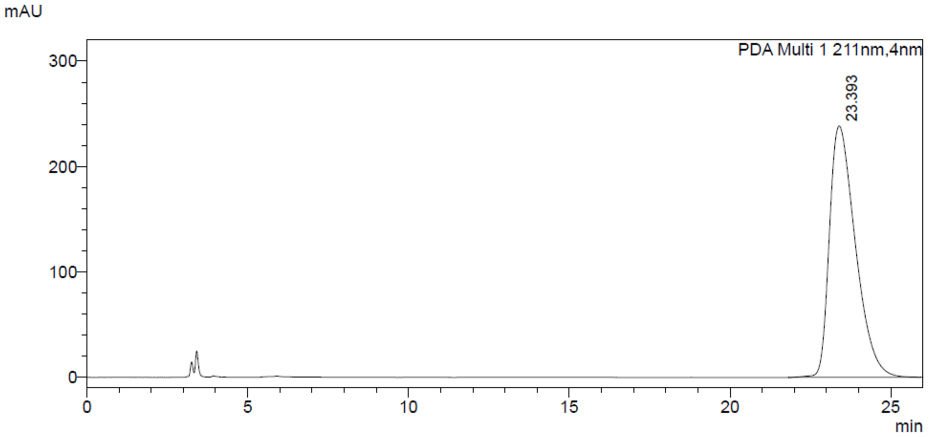

<Peak Table>

PDA Ch1 211nm

Peak#	Ret. Time	Area%
1	23.393	100.000
Total		100.000

HPLC Data for **20**: Chiralcel OD-H (90:10 hexane:IPA, flow rate 1.0 mLmin^{-1}, 211 nm, 30 ℃), t_R(2*S*,3*S*): 7.5 min, t_R(2*R*,3*R*): 9.5 min, >99:1 er.

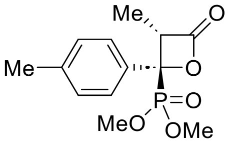

20

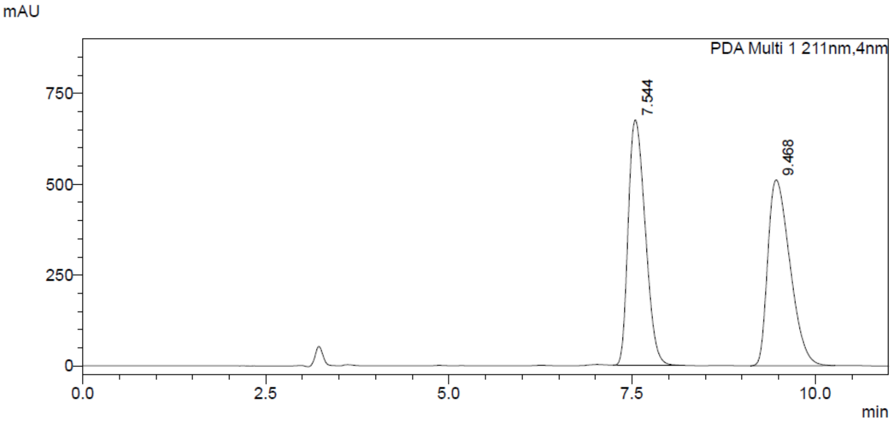

<Peak Table>

PDA Ch1 211nm

Peak#	Ret. Time	Area%
1	7.544	50.275
2	9.468	49.725
Total		100.000

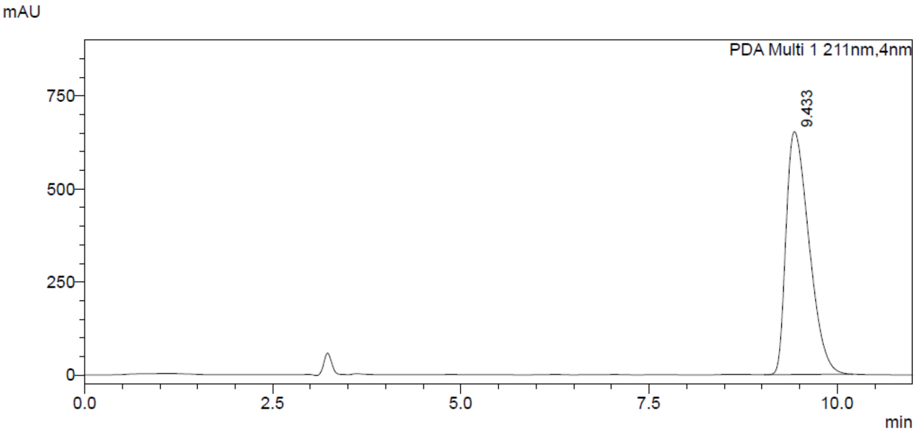

<Peak Table>

PDA Ch1 211nm

Peak#	Ret. Time	Area%
1	9.433	100.000
Total		100.000

HPLC Data for **21**: Chiralcel OD-H (90:10 hexane:IPA, flow rate 1.0 mLmin^{-1}, 211 nm, 30 ℃), t_R(2*S*,3*S*): 11.2 min, t_R(2*R*,3*R*): 14.3 min, >99:1 er.

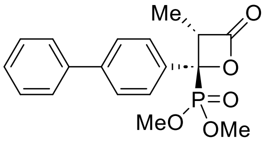

21

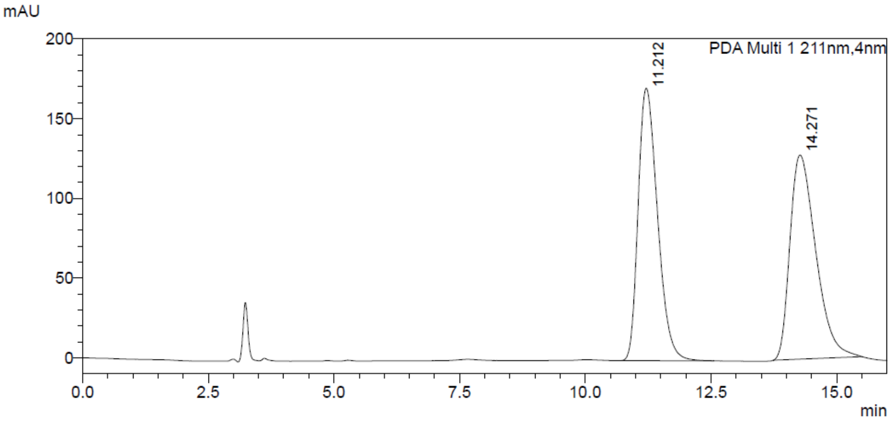

<Peak Table>

PDA Ch1 211nm

Peak#	Ret. Time	Area%
1	11.212	50.077
2	14.271	49.923
Total		100.000

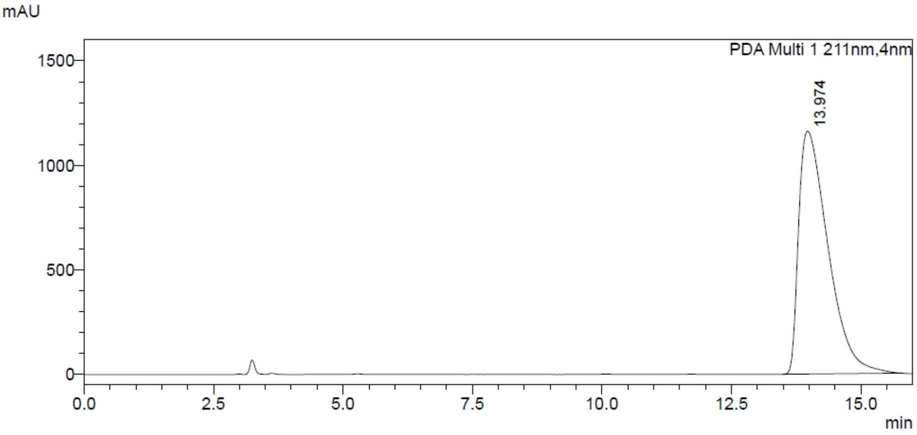

<Peak Table>

PDA Ch1 211nm

Peak#	Ret. Time	Area%
1	13.974	100.000
Total		100.000

HPLC Data for **22**: Chiralcel OD-H (90:10 hexane:IPA, flow rate 1.0 mLmin^{-1}, 211 nm, 30 ℃), t_R(2*S*,3*S*): 9.0 min, t_R(2*R*,3*R*): 11.0 min, >99:1 er.

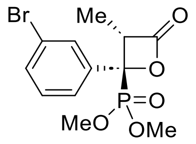

22

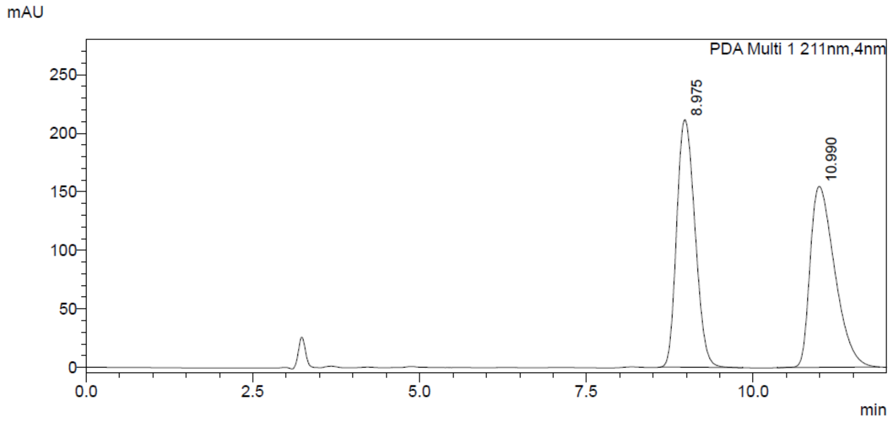

<Peak Table>

PDA Ch1 211nm

Peak#	Ret. Time	Area%
1	8.975	50.382
2	10.990	49.618
Total		100.000

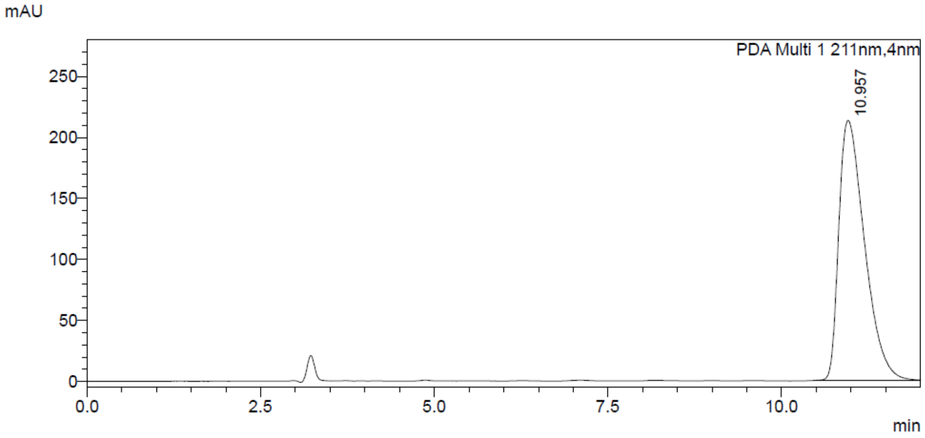

<Peak Table>

PDA Ch1 211nm

Peak#	Ret. Time	Area%
1	10.957	100.000
Total		100.000

HPLC Data for **23**: Chiralcel OD-H (90:10 hexane:IPA, flow rate 1.0 mLmin^{-1}, 211 nm, 30 ℃), t_R(2*S*,3*S*): 7.3 min, t_R(2*R*,3*R*): 8.7 min, >99:1 er.

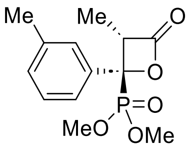

23

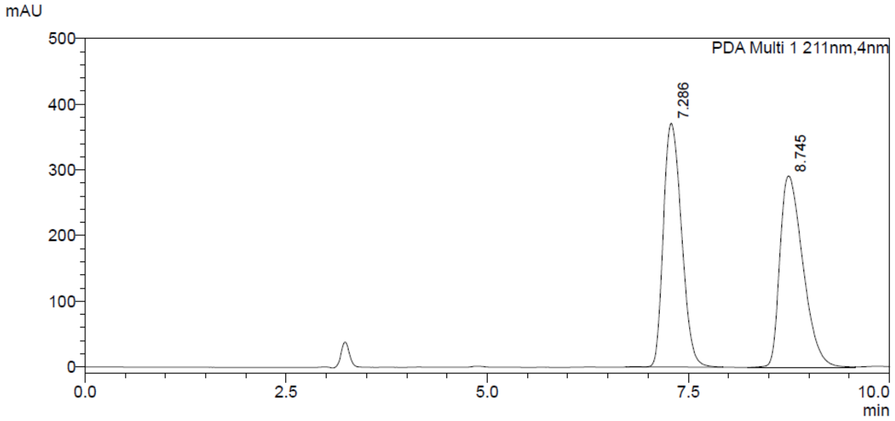

<Peak Table>

PDA Ch1 211nm

Peak#	Ret. Time	Area%
1	7.286	49.865
2	8.745	50.135
Total		100.000

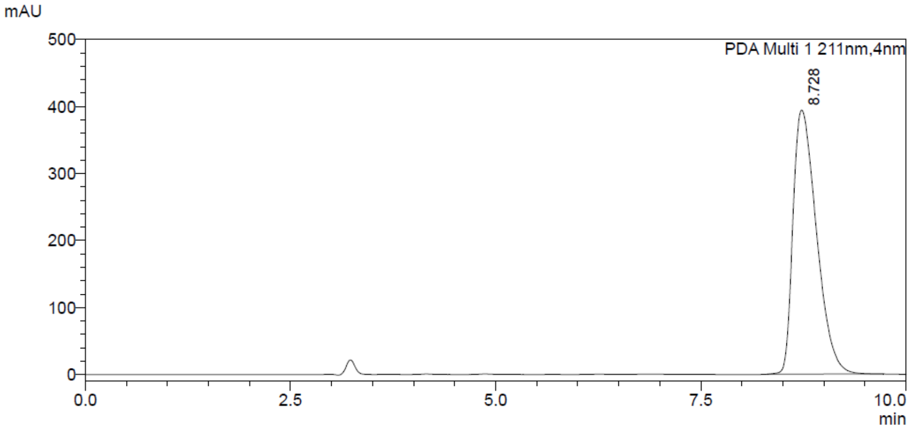

<Peak Table>

PDA Ch1 211nm

Peak#	Ret. Time	Area%
1	8.728	100.000
Total		100.000

HPLC Data for **24**: Chiralcel OJ-H (90:10 hexane:IPA, flow rate 1.0 mLmin^{-1}, 211nm, 30 ℃), t_R(2*S*,3*S*): 20.9 min, t_R(2*R*,3*R*): 27.1 min, >99:1 er.

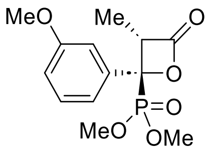

24

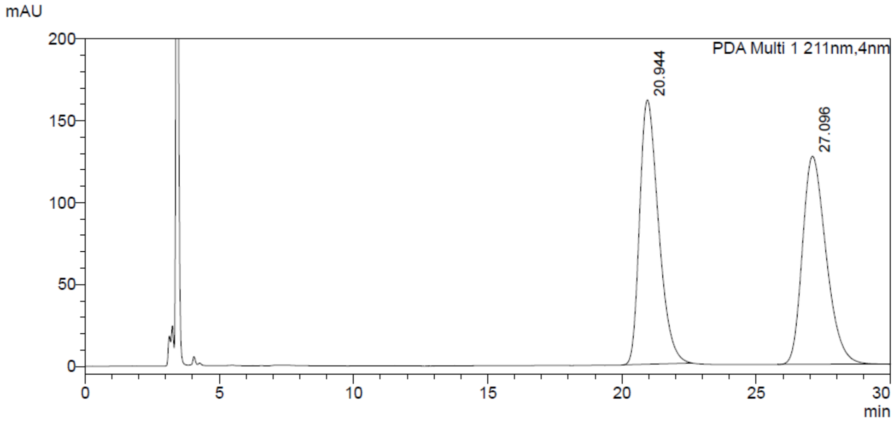

<Peak Table>

PDA Ch1 211nm

Peak#	Ret. Time	Area%
1	20.944	50.323
2	27.096	49.677
Total		100.000

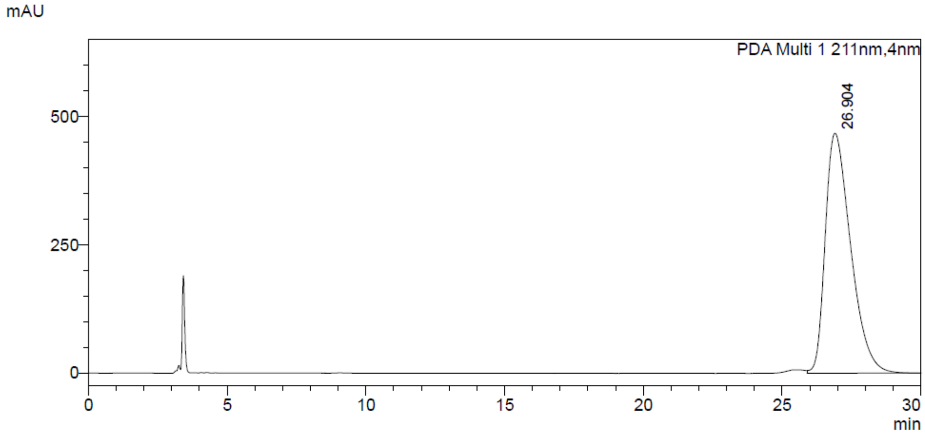

<Peak Table>

PDA Ch1 211nm

Peak#	Ret. Time	Area%
1	26.904	100.000
Total		100.000

HPLC Data for **25**: Chiralcel OD-H (90:10 hexane:IPA, flow rate 1.0 mLmin^{-1}, 211nm, 30 ℃), t_R(2*S*,3*S*): 10.6 min, t_R(2*R*,3*R*): 14.6 min, >99:1 er.

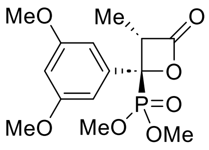

25

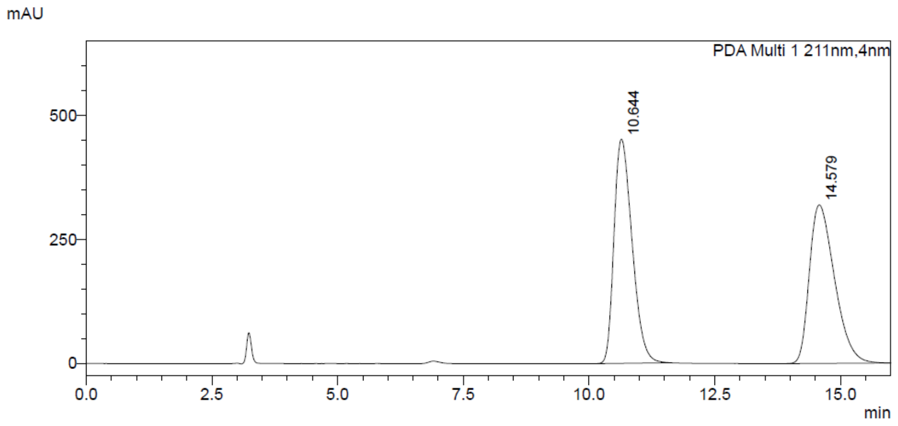

<Peak Table>

PDA Ch1 211nm

Peak#	Ret. Time	Area%
1	10.644	50.348
2	14.579	49.652
Total		100.000

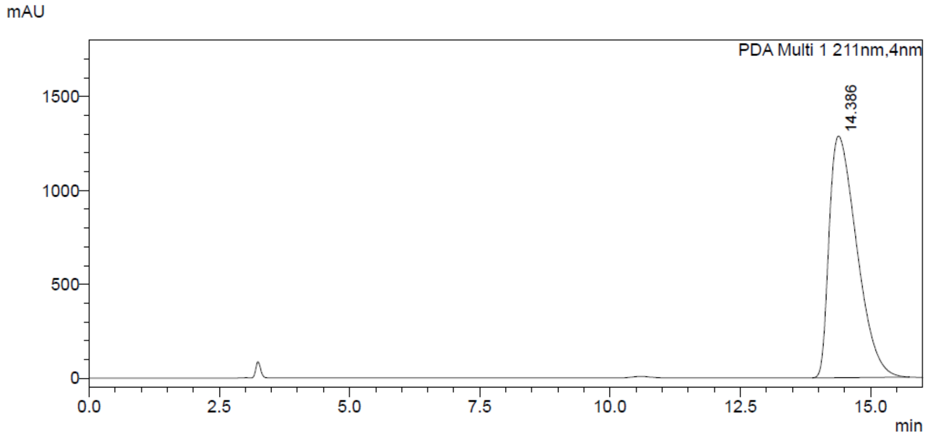

<Peak Table>

PDA Ch1 211nm

Peak#	Ret. Time	Area%
1	14.386	100.000
Total		100.000

HPLC Data for **26**: Chiralcel OD-H (90:10 hexane:IPA, flow rate 1.0 mLmin^{-1}, 211 nm, 30 ℃), t_R(2*S*,3*S*): 10.0 min, t_R(2*R*,3*R*): 13.7 min, >99:1 er.

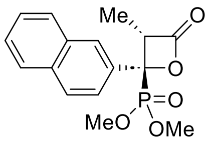

26

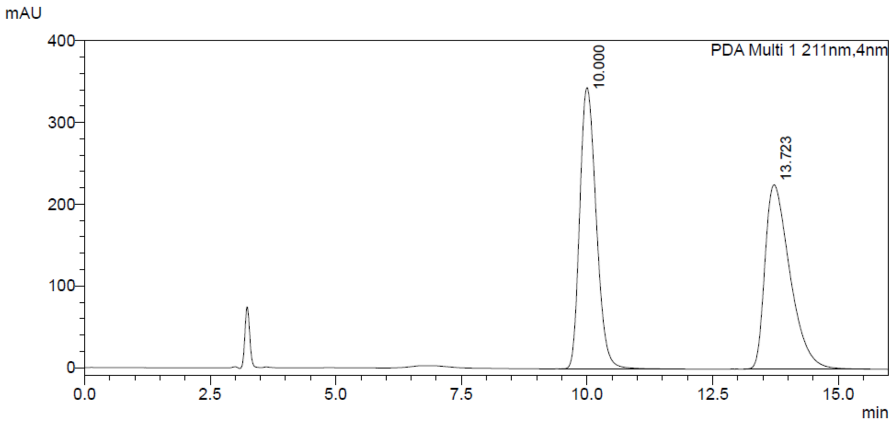

<Peak Table>

PDA Ch1 211nm

Peak#	Ret. Time	Area%
1	10.000	50.089
2	13.723	49.911
Total		100.000

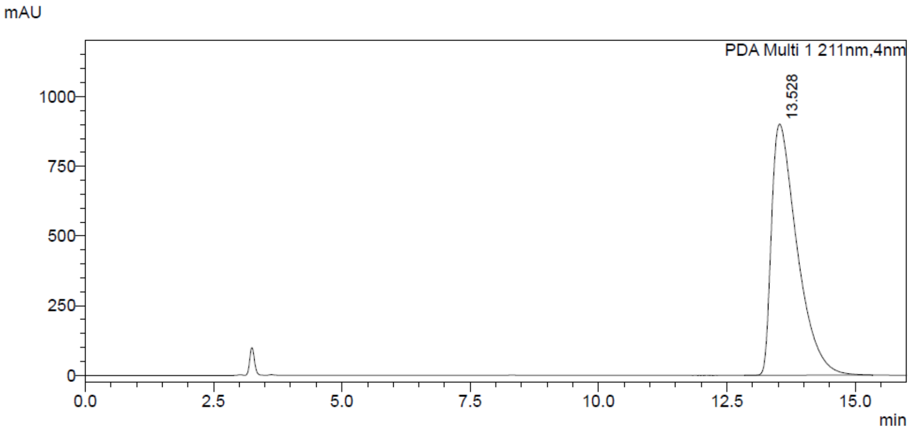

<Peak Table>

PDA Ch1 211nm

Peak#	Ret. Time	Area%
1	13.528	100.000
Total		100.000

HPLC Data for **27**: Chiralcel OJ-H (90:10 hexane:IPA, flow rate 1.0 mLmin^{-1}, 211 nm, 30 ℃), t_R(2*S*,3*S*): 15.3 min, t_R(2*R*,3*R*): 17.1 min, >99:1 er.

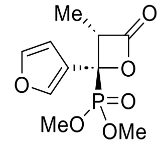

27

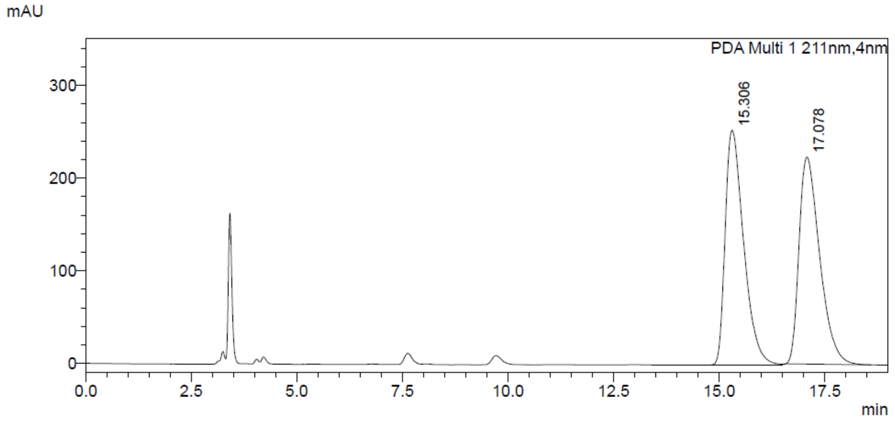

<Peak Table>

PDA Ch1 211nm

Peak#	Ret. Time	Area%
1	15.306	50.390
2	17.078	49.610
Total		100.000

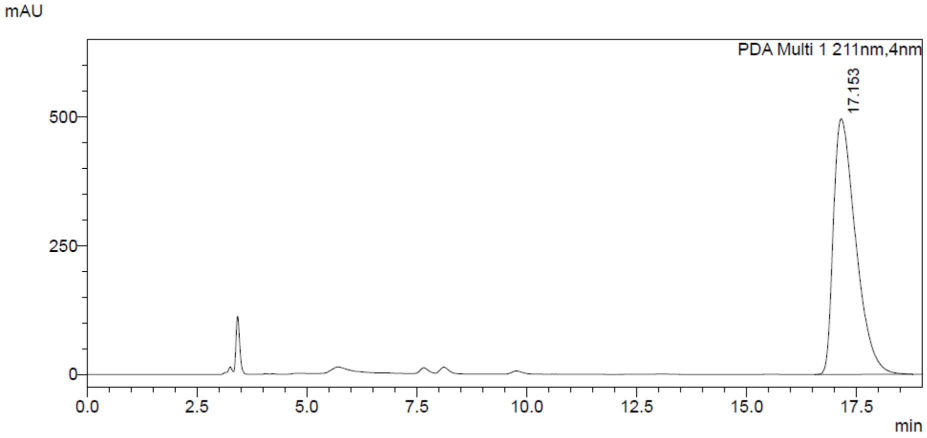

<Peak Table>

PDA Ch1 211nm

Peak#	Ret. Time	Area%
1	17.153	100.000
Total		100.000

HPLC Data for **28**: Chiralcel OD-H (90:10 hexane:IPA, flow rate 1.0 mLmin^{-1}, 211 nm, 30 ℃), t_R(2*S*,3*S*): 10.6 min, t_R(2*R*,3*R*): 14.6 min, >99:1 er.

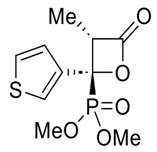

28

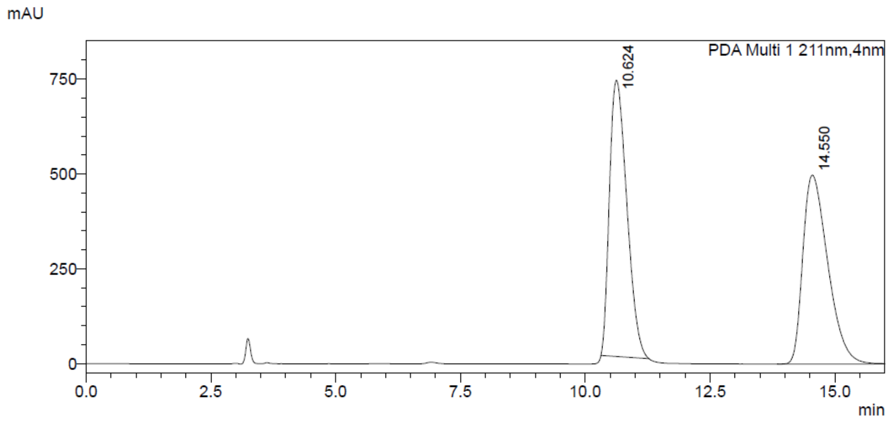

<Peak Table>

PDA Ch1 211nm

Peak#	Ret. Time	Area%
1	10.624	50.342
2	14.550	49.658
Total		100.000

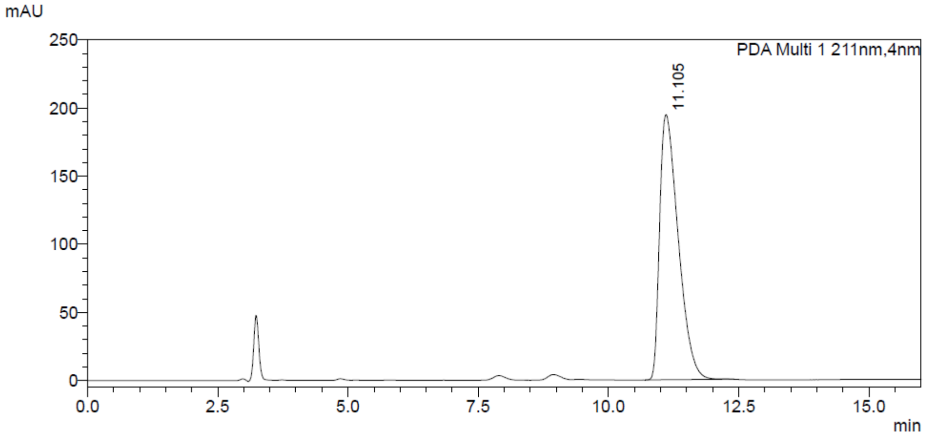

<Peak Table>

PDA Ch1 211nm

Peak#	Ret. Time	Area%
1	11.105	100.000
Total		100.000

GC Data for **29**: Rt-βDEXsm Column (110 ℃ hold for 60 min, 1.0 ℃min^{-1}, 110 – 130 ℃, 0.5 ℃min^{-1}), t_R(2*R*,3*R*): 115.2 min, t_R(2*S*,3*S*): 116.9 min, >99:1 er.

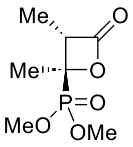

29

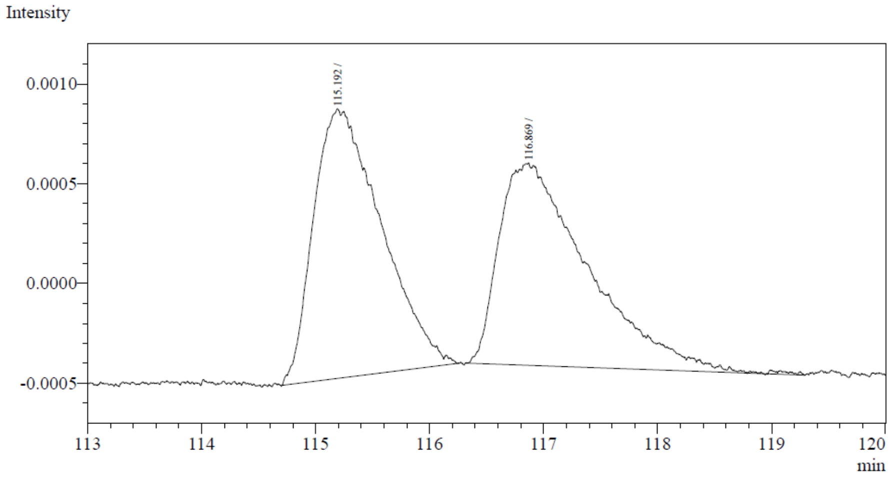

FID1

Peak#	Ret.Time	Area	Height	Conc.	Unit	Mark	ID#	Cmpd Name
1	115.192	55839	1352	50.430		M		
2	116.869	54886	1015	49.570		M		
Total		110725	2367					

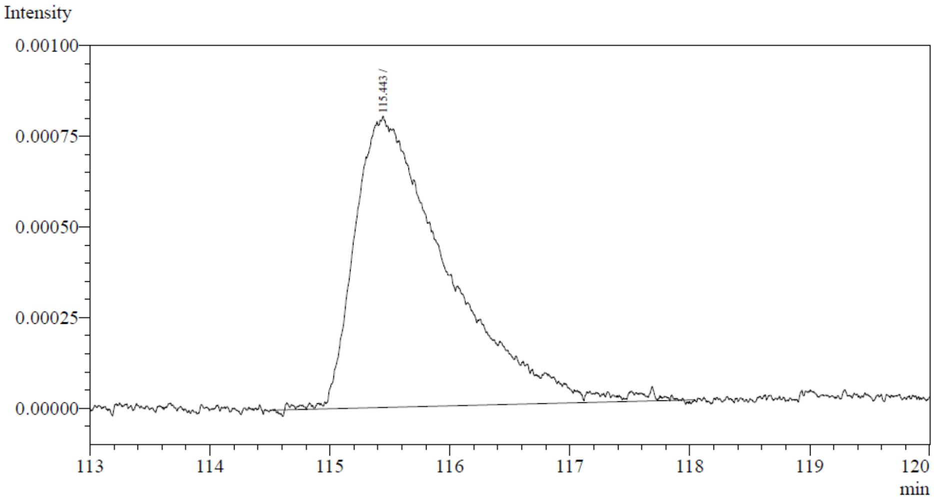

FID1

Peak#	Ret.Time	Area	Height	Conc.	Unit	Mark	ID#	Cmpd Name
1	115.443	43301	804	100.000		M		
Total		43301	804					

HPLC Data for **30**: Chiralcel OD-H (90:10 hexane:IPA, flow rate 1.0 mLmin^{-1}, 211 nm, 30 ℃), t_R(2*S*,3*S*): 8.8 min, t_R(2*R*,3*R*): 11.6 min, >99:1 er.

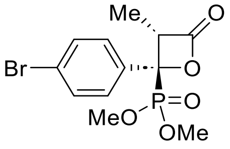

30

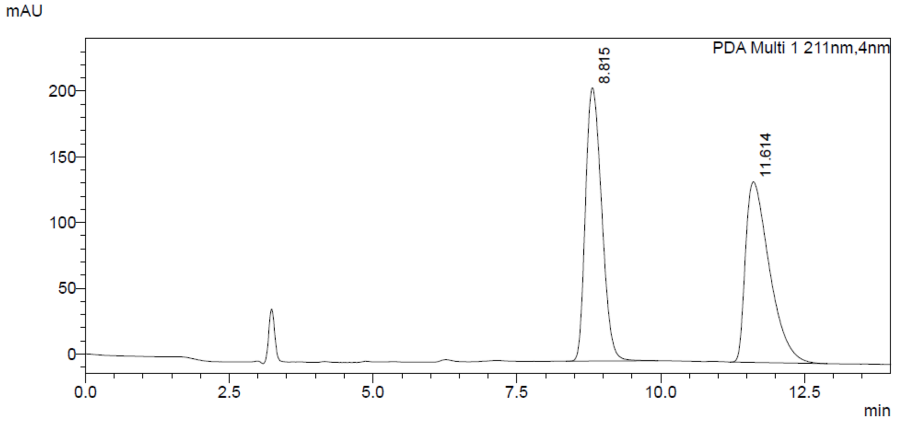

<Peak Table>

PDA Ch1 211nm

Peak#	Ret. Time	Area%
1	8.815	50.527
2	11.614	49.473
Total		100.000

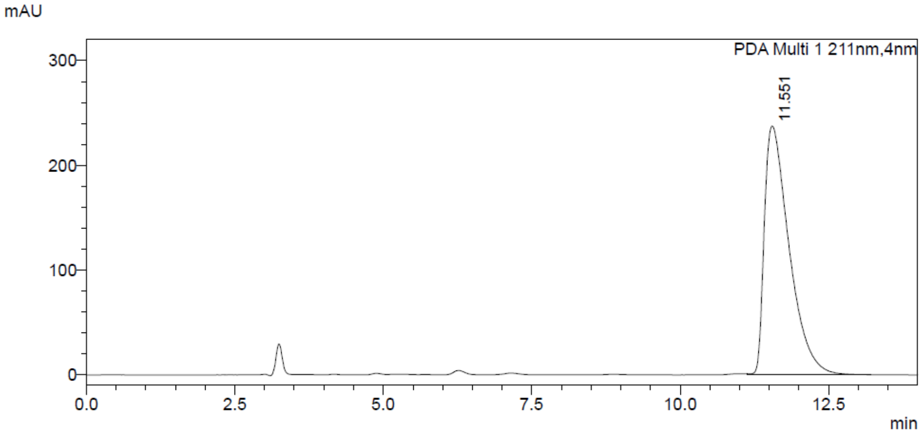

<Peak Table>

PDA Ch1 211nm

Peak#	Ret. Time	Area%
1	11.551	100.000
Total		100.000

HPLC Data for **31**: Chiralcel OD-H (95:5 hexane:IPA, flow rate 0.5 mLmin^{-1}, 211 nm, 30 ℃), t_R(2*S*,3*S*): 34.4 min, t_R(2*R*,3*R*): 38.7 min, >99:1 er.

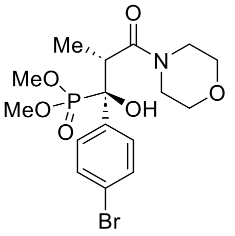

31

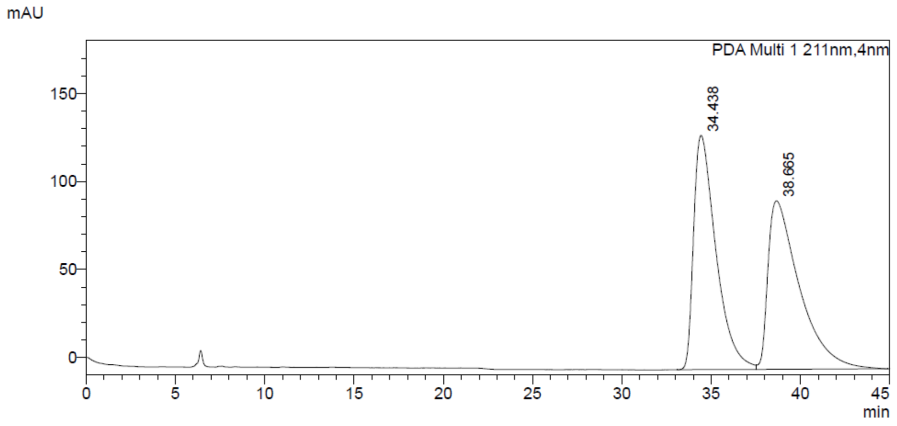

<Peak Table>

PDA Ch1 211nm

Peak#	Ret. Time	Area%
1	34.438	49.692
2	38.665	50.308
Total		100.000

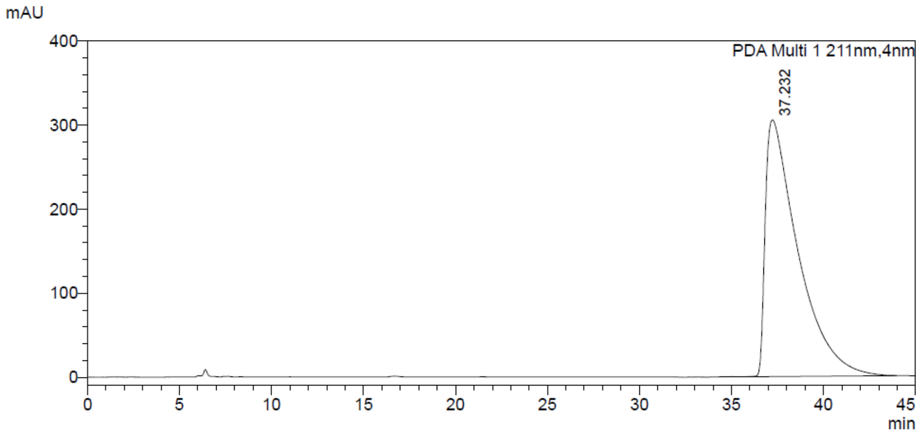

<Peak Table>

PDA Ch1 211nm

Peak#	Ret. Time	Area%
1	37.232	100.000
Total		100.000

HPLC Data for **36**: Chiralcel OD-H (90:10 hexane:IPA, flow rate 1.0 mLmin^{-1}, 211 nm, 30 ℃), t_R(2*S*,3*S*): 7.4 min, t_R(2*R*,3*R*): 8.2 min, >99:1 er.

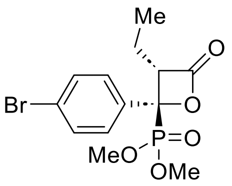

36

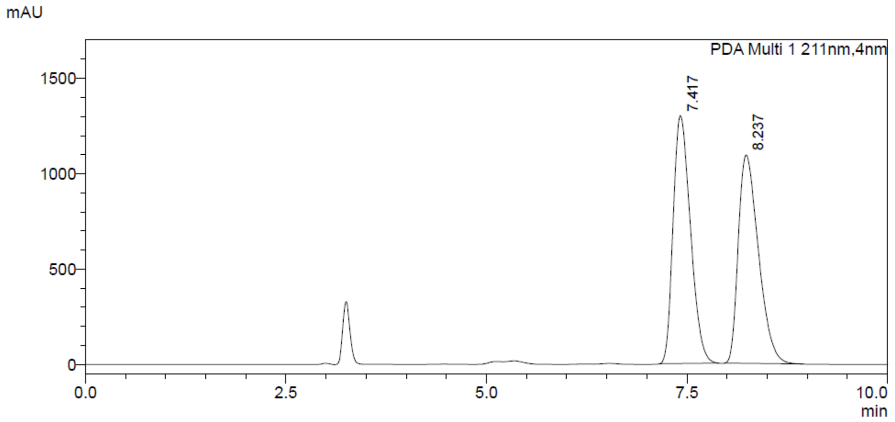

<Peak Table>

PDA Ch1 211nm

Peak#	Ret. Time	Area%
1	7.417	50.539
2	8.237	49.461
Total		100.000

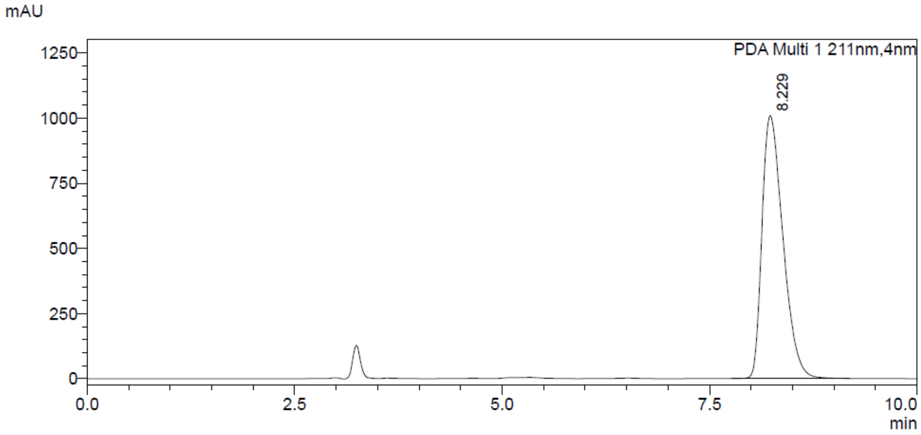

<Peak Table>

PDA Ch1 211nm

Peak#	Ret. Time	Area%
1	8.229	100.000
Total		100.000

HPLC Data for **37**: Chiralcel OJ-H (95:5 hexane:IPA, flow rate 1.0 mLmin^{-1}, 211 nm, 30 ℃), t_R(2*S*,3*S*): 10.2 min, t_R(2*R*,3*R*): 11.9 min, >99:1 er.

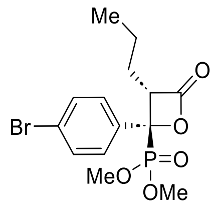

37

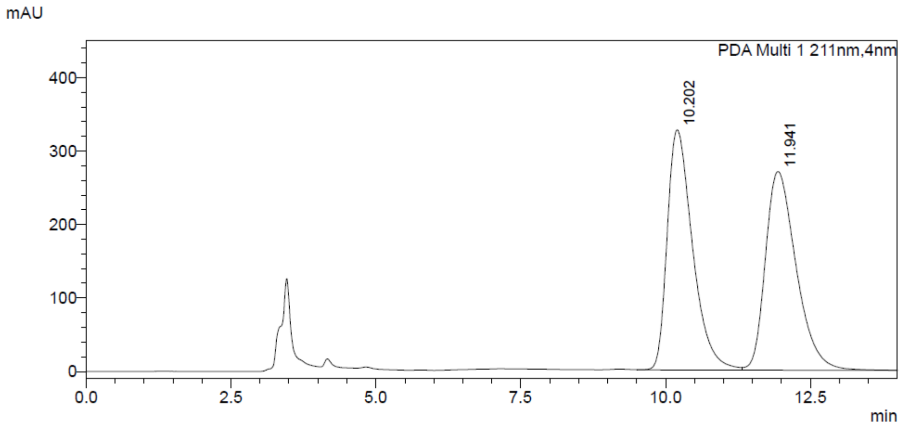

<Peak Table>

PDA Ch1 211nm

Peak#	Ret. Time	Area%
1	10.202	49.902
2	11.941	50.098
Total		100.000

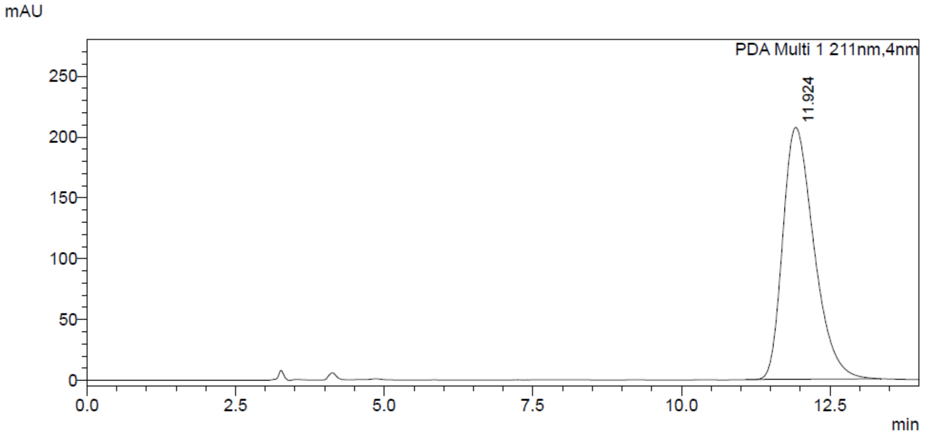

<Peak Table>

PDA Ch1 211nm

Peak#	Ret. Time	Area%
1	11.924	100.000
Total		100.000

HPLC Data for **38**: Chiralcel OJ-H (90:10 hexane:IPA, flow rate 1.0 mLmin^{-1}, 211 nm, 30 ℃), t_R(2*S*,3*S*): 9.7 min, t_R(2*R*,3*R*): 13.1 min, >99:1 er.

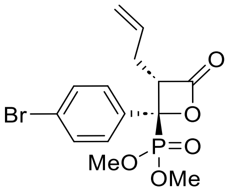

38

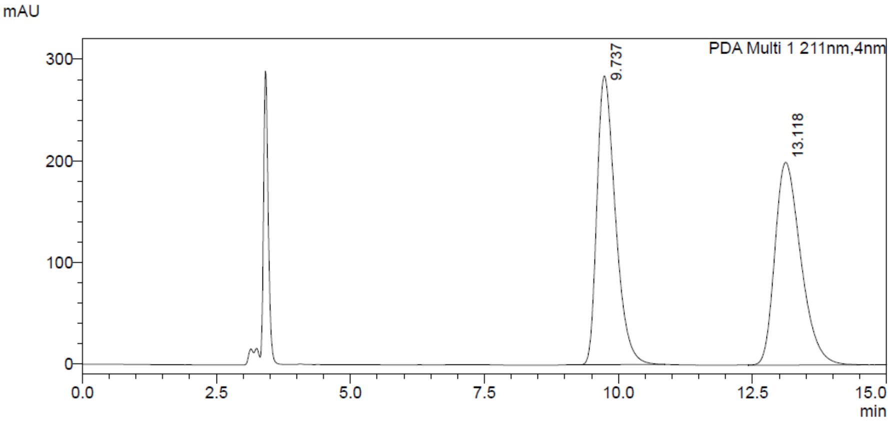

<Peak Table>

PDA Ch1 211nm

Peak#	Ret. Time	Area%
1	9.737	50.229
2	13.118	49.771
Total		100.000

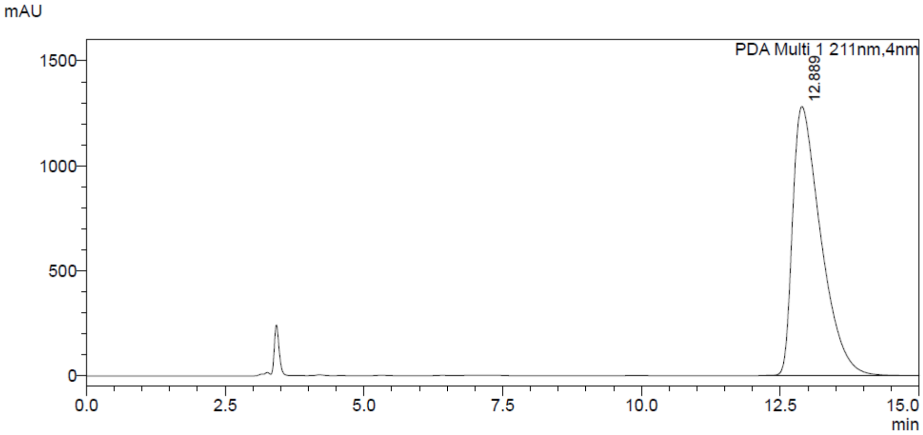

<Peak Table>

PDA Ch1 211nm

Peak#	Ret. Time	Area%
1	12.889	100.000
Total		100.000

HPLC Data for **39**: Chiralcel OJ-H (90:10 hexane:IPA, flow rate 1.0 mL min^{-1}, 211 nm, 30 ℃), t_R(2*S*,3*S*): 20.2 min, t_R(2*R*,3*R*): 30.2 min, >99:1 er.

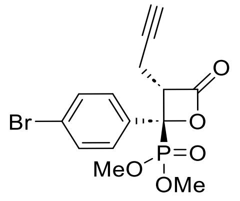

39

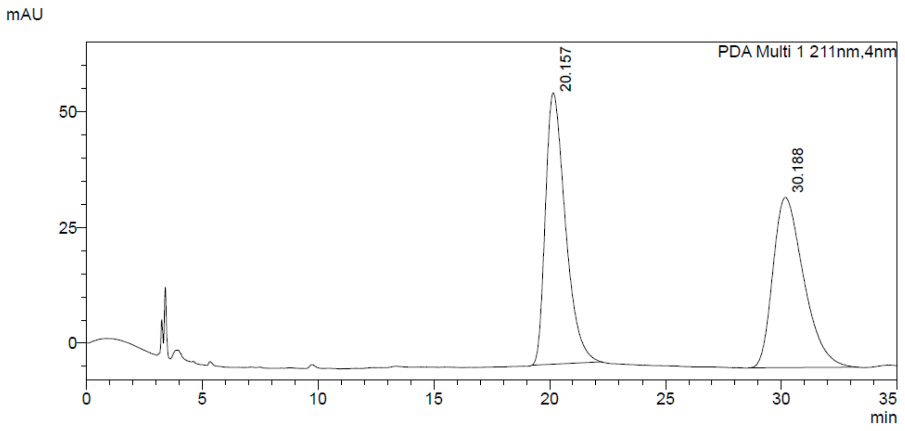

<Peak Table>

PDA Ch1 211nm

Peak#	Ret. Time	Area%
1	20.157	50.825
2	30.188	49.175
Total		100.000

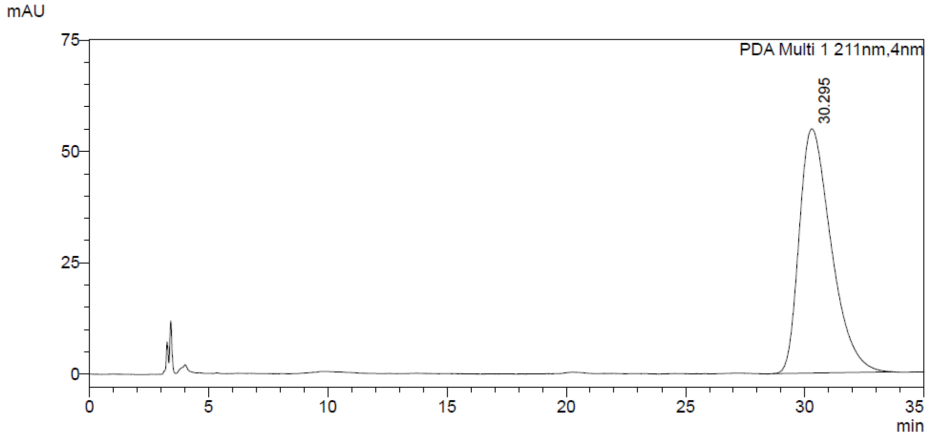

<Peak Table>

PDA Ch1 211nm

Peak#	Ret. Time	Area%
1	30.295	100.000
Total		100.000

HPLC Data for **40**: Chiralcel OJ-H (90:10 hexane:IPA, flow rate 1.0 mLmin^{-1}, 211 nm, 30 ℃), t_R(2*S*,3*S*): 20.1 min, t_R(2*R*,3*R*): 30.9 min, >99:1 er.

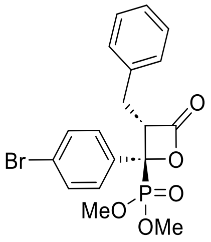

40

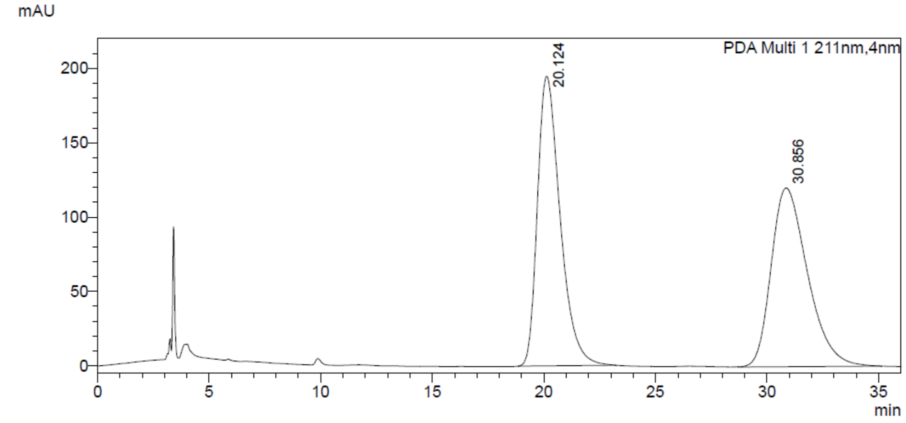

<Peak Table>

PDA Ch1 211nm

Peak#	Ret. Time	Area%
1	20.124	50.322
2	30.856	49.678
Total		100.000

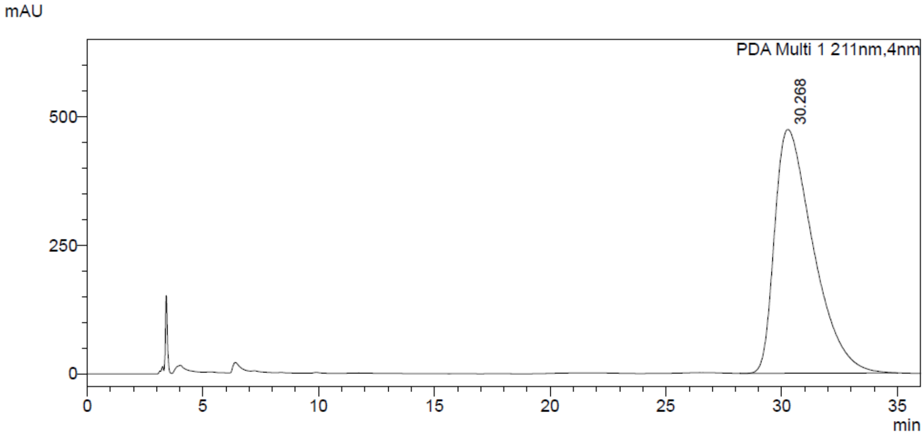

<Peak Table>

PDA Ch1 211nm

Peak#	Ret. Time	Area%
1	30.268	100.000
Total		100.000

HPLC Data for **41**: Chiralcel OJ-H (90:10 hexane:IPA, flow rate 1.0 mLmin^{-1}, 211 nm, 30 ℃), t_R(2*S*,3*S*): 14.4 min, t_R(2*R*,3*R*): 25.3 min, >99:1 er.

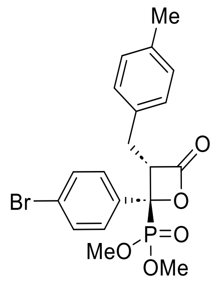

41

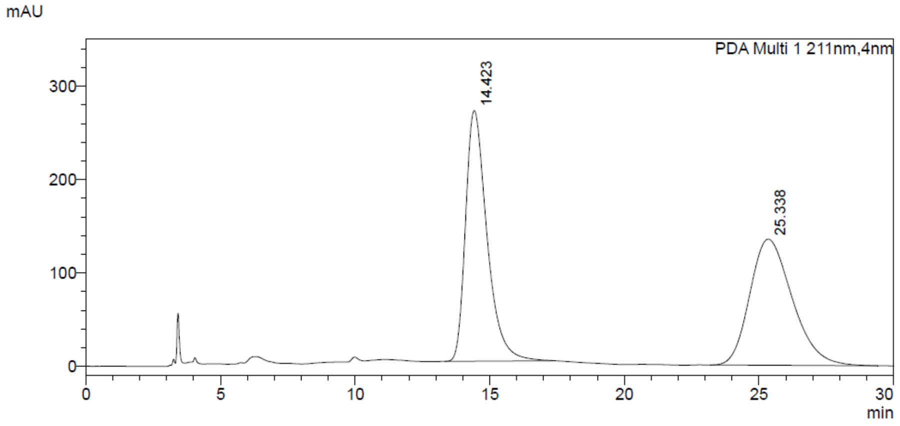

<Peak Table>

PDA Ch1 211nm

Peak#	Ret. Time	Area%
1	14.423	50.171
2	25.338	49.829
Total		100.000

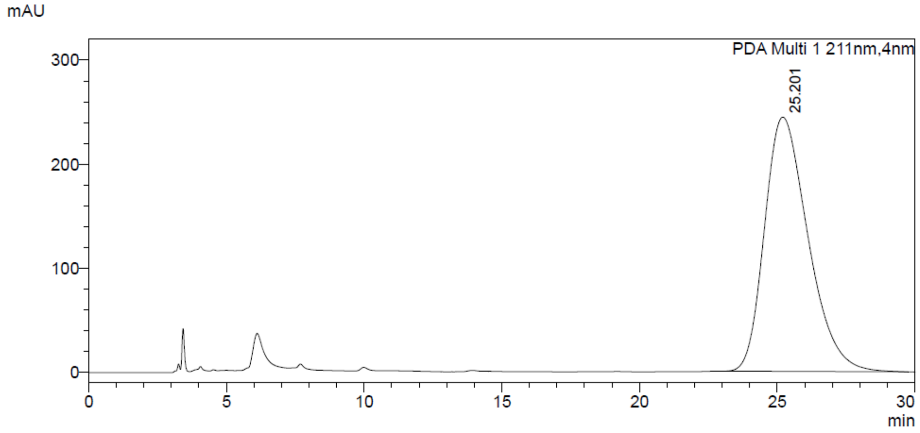

<Peak Table>

PDA Ch1 211nm

Peak#	Ret. Time	Area%
1	25.201	100.000
Total		100.000

HPLC Data for **42**: Chiralcel OJ-H (90:10 hexane:IPA, flow rate 1.0 mLmin^{-1}, 211 nm, 30 ℃), t_R(2*S*,3*S*): 19.0 min, t_R(2*R*,3*R*): 36.7 min, >99:1 er.

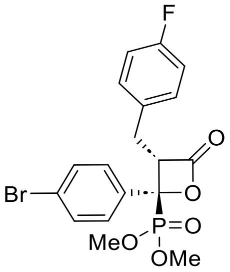

42

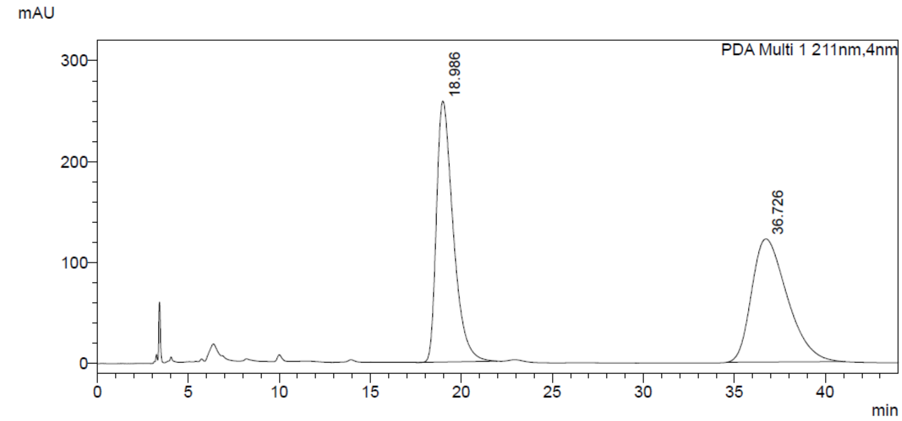

<Peak Table>

PDA Ch1 211nm

Peak#	Ret. Time	Area%
1	18.986	49.867
2	36.726	50.133
Total		100.000

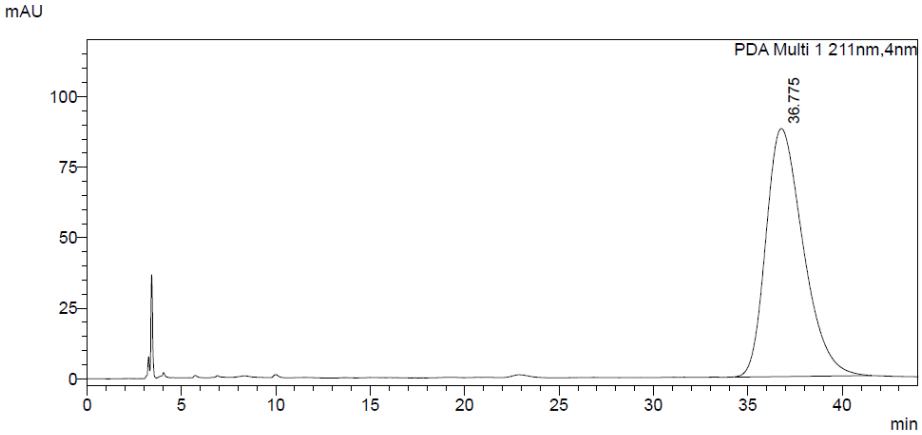

<Peak Table>

PDA Ch1 211nm

Peak#	Ret. Time	Area%
1	36.775	100.000
Total		100.000

HPLC Data for **43**: Chiralcel OD-H (95:5 hexane:IPA, flow rate 0.5 mLmin^{-1}, 211 nm, 30 ℃), t_R(2*S*,3*S*): 60.7 min, t_R(2*R*,3*R*): 66.7 min, >99:1 er.

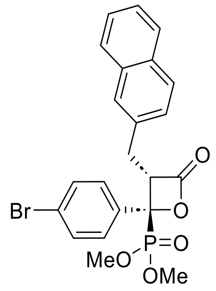

43

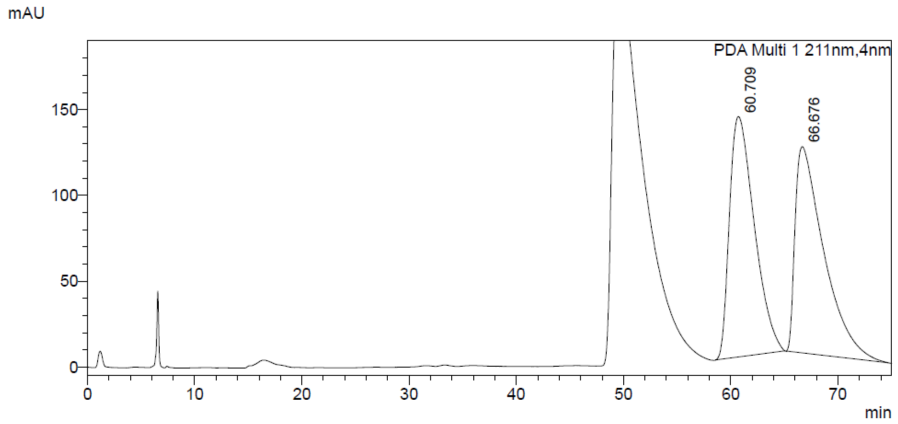

<Peak Table>

PDA Ch1 211nm

Peak#	Ret. Time	Area%
1	60.709	50.249
2	66.676	49.751
Total		100.000

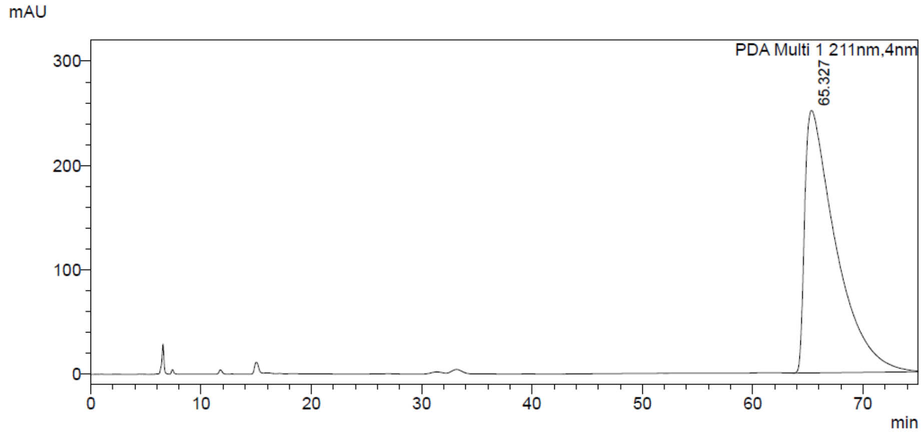

<Peak Table>

PDA Ch1 211nm

Peak#	Ret. Time	Area%
1	65.327	100.000
Total		100.000

HPLC Data for **44**: Chiralpak ID (90:10 hexane:IPA, flow rate 1.0 mLmin^{-1}, 211 nm, 30 ℃), t_R (2*S*,3*S*): 19.7 min, t_R (2*R*,3*R*): 21.4 min, 99:1 er.

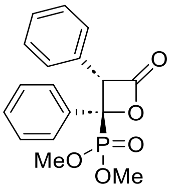

44

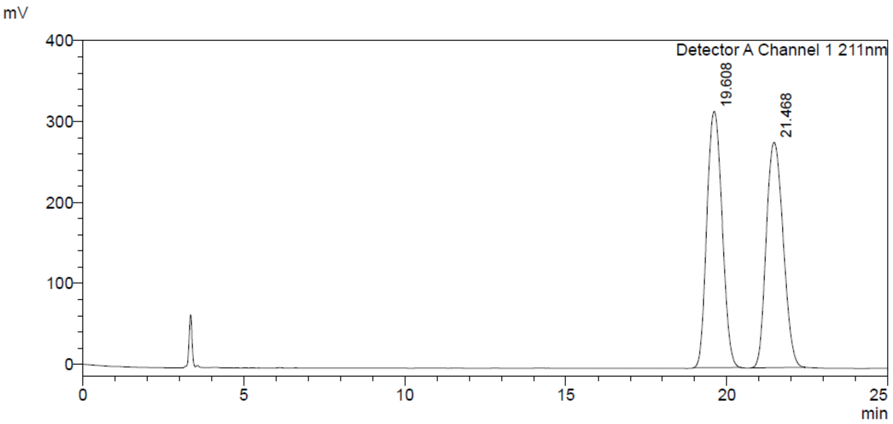

<Peak Table>

Detector A Channel 1 211nm

Peak#	Ret. Time	Area%
1	19.608	50.557
2	21.468	49.443
Total		100.000

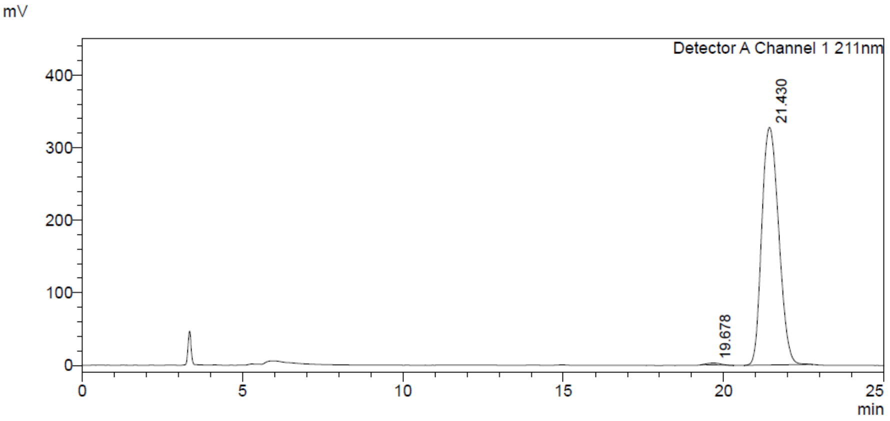

<Peak Table>

Detector A Channel 1 211nm

Peak#	Ret. Time	Area%
1	19.678	0.579
2	21.430	99.421
Total		100.000

HPLC Data for *anti*-**44** and *syn*-**44**: Chiralpak AD-H (95:5 hexane : IPA, flow rate 2.0 mLmin^{-1}, 220 nm, 30 ℃) t_R(2*S*,3*S*): 8.5 min, t_R(2*R*,3*R*): 10.7 min, t_R(2*S*,3*R*): 12.9 min, t_R(2*R*,3*S*): 17.1 min, 96:4 er$_{syn}$, 96:4 er$_{anti}$.

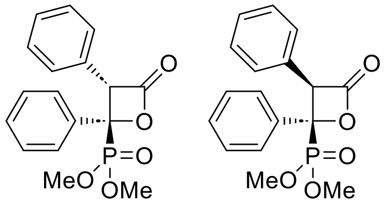

anti-**44** *syn*-**44**

PDA Ch2 220nm

Peak#	Ret. Time	Area%
1	8.461	34.044
2	10.702	33.854
3	12.937	15.457
4	17.120	16.645
Total		100.000

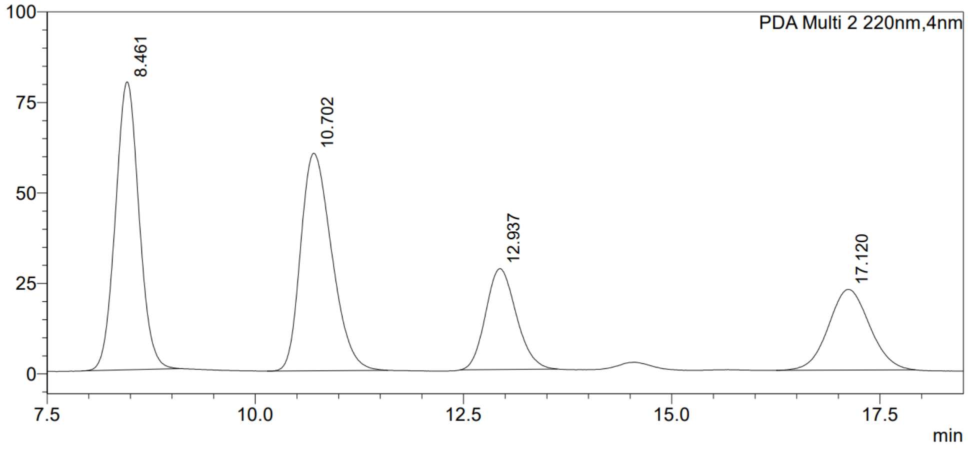

PDA Ch1 211nm

Peak#	Ret. Time	Area%
1	8.470	3.432
2	10.545	78.624
3	12.881	0.794
4	16.977	17.150
Total		100.000

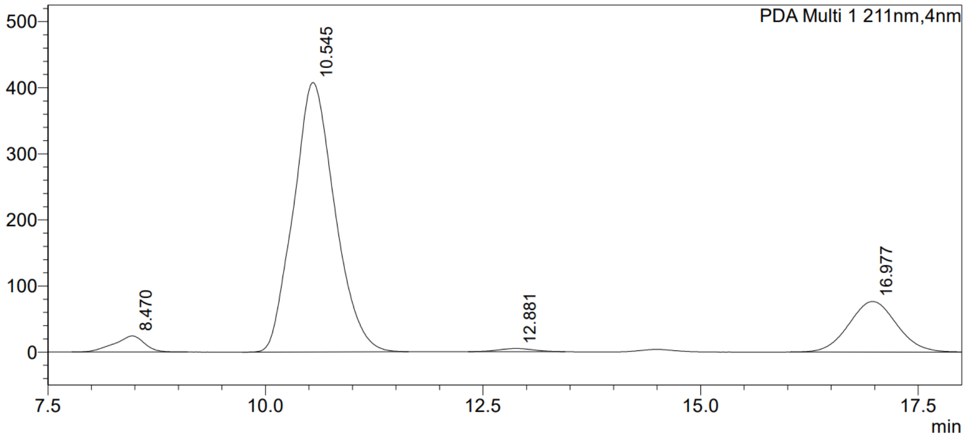

HPLC Data for *anti*-**48** and *syn*-**48**: Chiralpak AD-H (95:5 hexane : IPA, flow rate 2.0 mLmin^{-1}, 220 nm, 30 ℃) t_R(2*S*,3*S*): 13.8 min, t_R(2*R*,3*R*): 15.8 min, t_R(2*S*,3*R*): 20.9 min, t_R(2*R*,3*S*): 30.0 min, 95:5 er$_{syn}$, 88:12 er$_{anti}$.

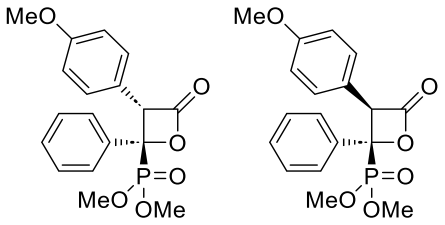

anti-**48** *syn*-**48**

PDA Ch1 211nm

Peak#	Ret. Time	Area%
1	13.784	41.670
2	15.804	43.178
3	20.896	6.756
4	30.067	8.397
Total		100.000

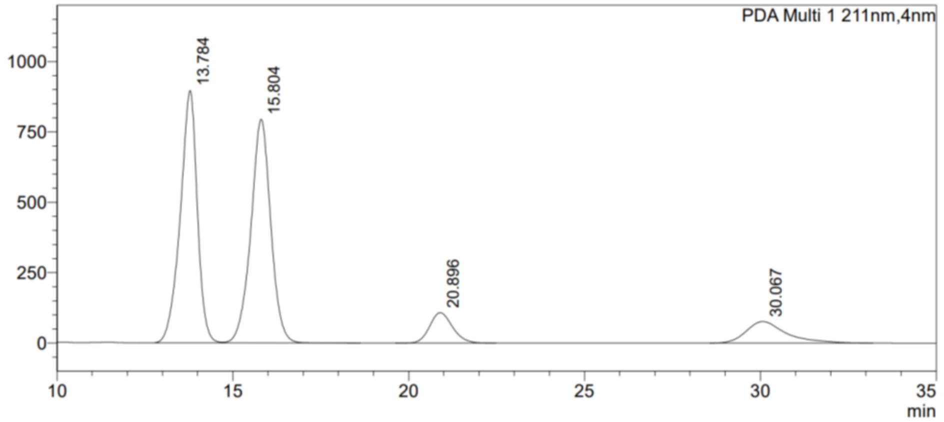

PDA Ch1 211nm

Peak#	Ret. Time	Area%
1	13.756	4.607
2	15.768	81.194
3	20.855	12.488
4	29.995	1.711
Total		100.000

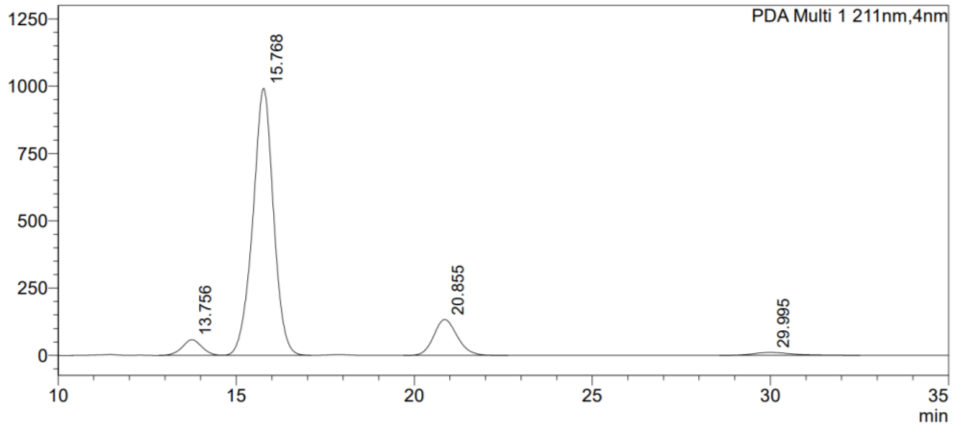

HPLC Data for *anti*-**49**: Chiralpak AD-H (97:3 hexane : IPA, flow rate 1.5 mLmin^{-1}, 220 nm, 30 °C) t_R(2*S*,3*S*): 20.3 min, t_R(2*R*,3*R*): 22.8 min, 95:5 er$_{anti}$.

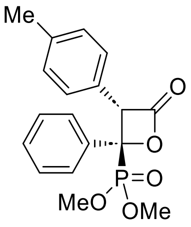

anti-**49**

PDA Ch2 220nm

Peak#	Ret. Time	Area%
1	20.598	49.576
2	22.848	50.424
Total		100.000

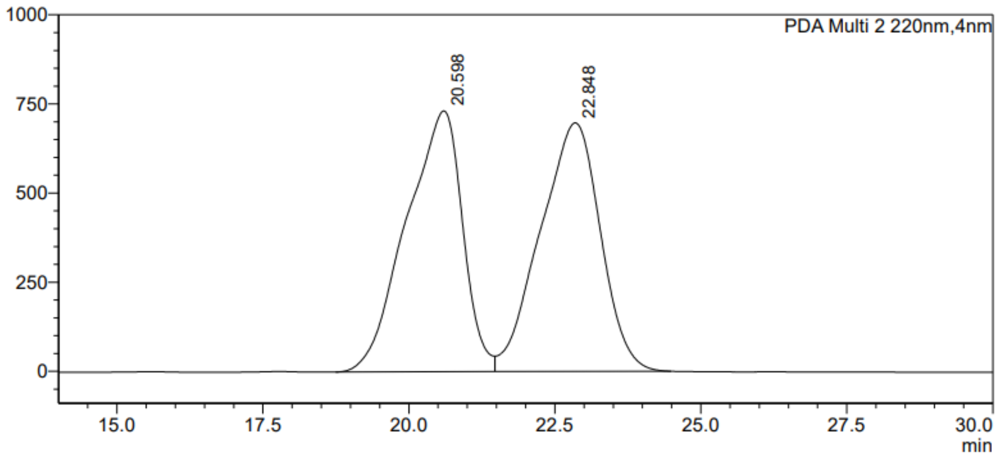

PDA Ch2 220nm

Peak#	Ret. Time	Area%
1	20.346	4.504
2	22.766	95.496
Total		100.000

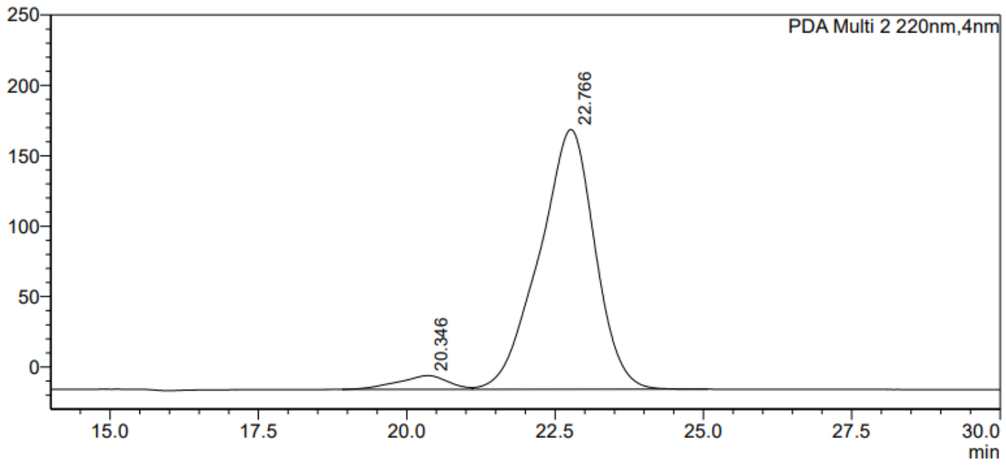

HPLC Data for *anti*-**50**: Chiralpak AD-H (95:5 hexane : IPA, flow rate 1.0 mLmin^{-1}, 211 nm, 30 °C) t_R(2*S*,3*S*): 16.5 min, t_R(2*R*,3*R*): 20.0 min, 95:5 er$_{anti}$.

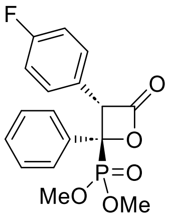

anti-**50**

Detector A Channel 1 211nm

Peak#	Ret. Time	Area%
1	16.505	49.798
2	20.065	50.202
Total		100.000

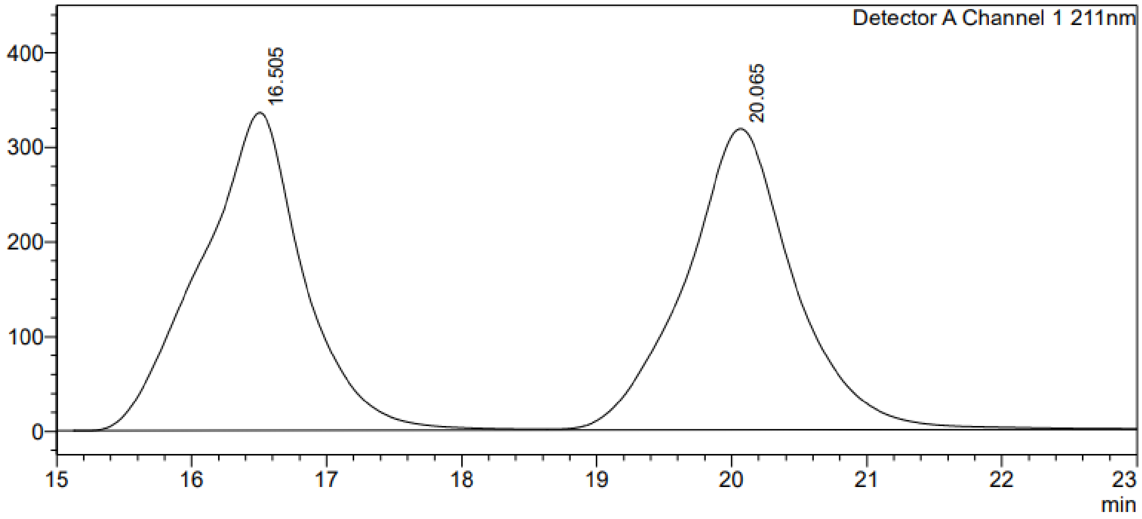

Peak#	Ret. Time	Area%
1	16.498	5.363
2	20.009	94.637
Total		100.000

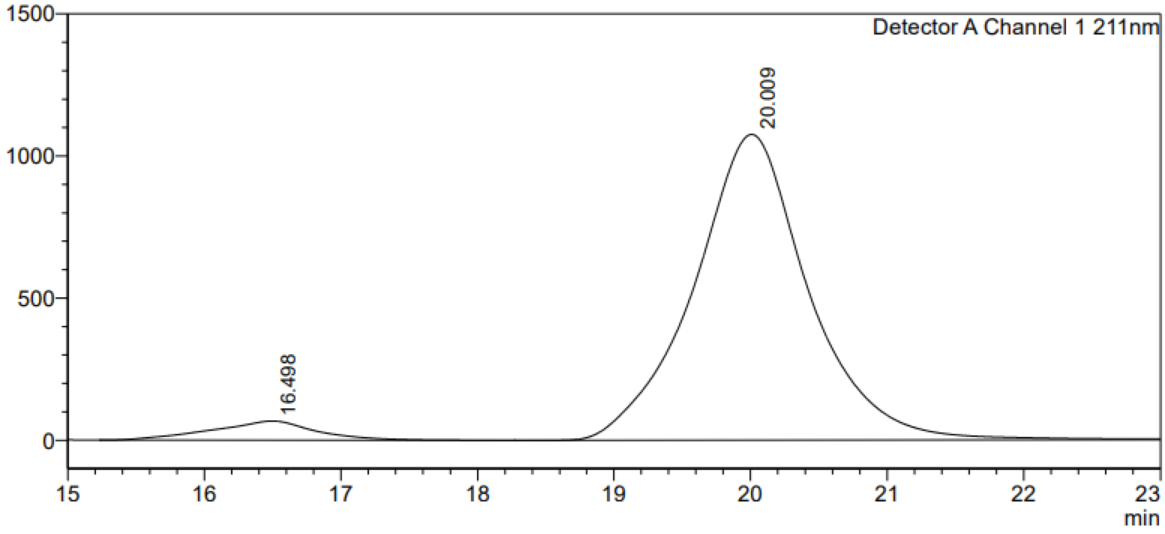

HPLC Data for *syn*-**50**: Chiralpak AD-H (95:5 hexane : IPA, flow rate 1.0 mLmin^{-1}, 211 nm, 30 ℃) t_R(2*R*,3*S*): 27.8 min, t_R(2*S*,3*R*): 30.1 min, 94:6 er$_{syn.}$

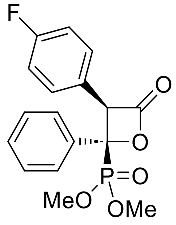

syn-**50**

Detector A Channel 1 211nm

Peak#	Ret. Time	Area%
1	27.890	49.173
2	30.345	50.827
Total		100.000

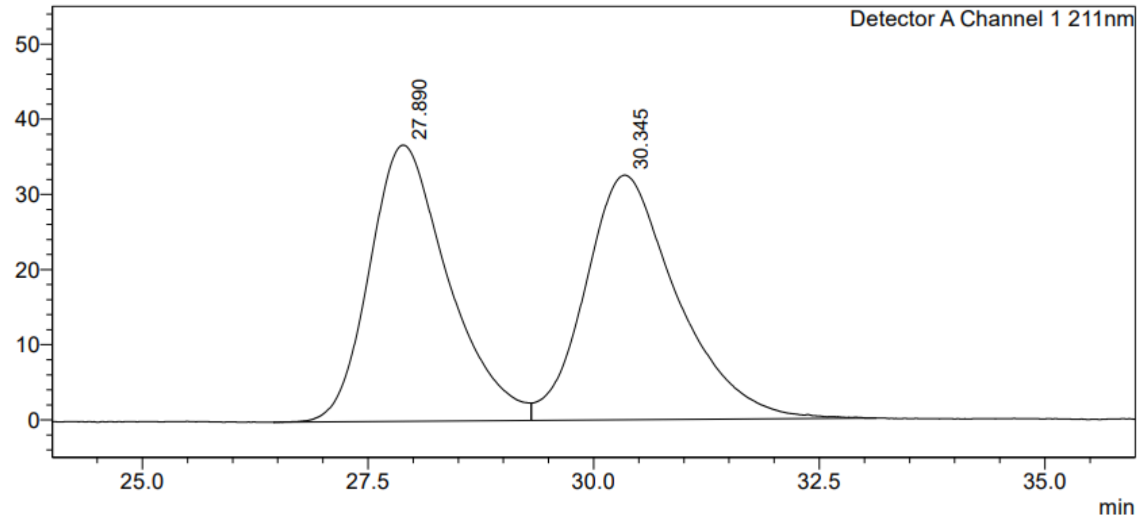

Detector A Channel 1 211nm

Peak#	Ret. Time	Area%
1	27.827	93.931
2	30.062	6.069
Total		100.000

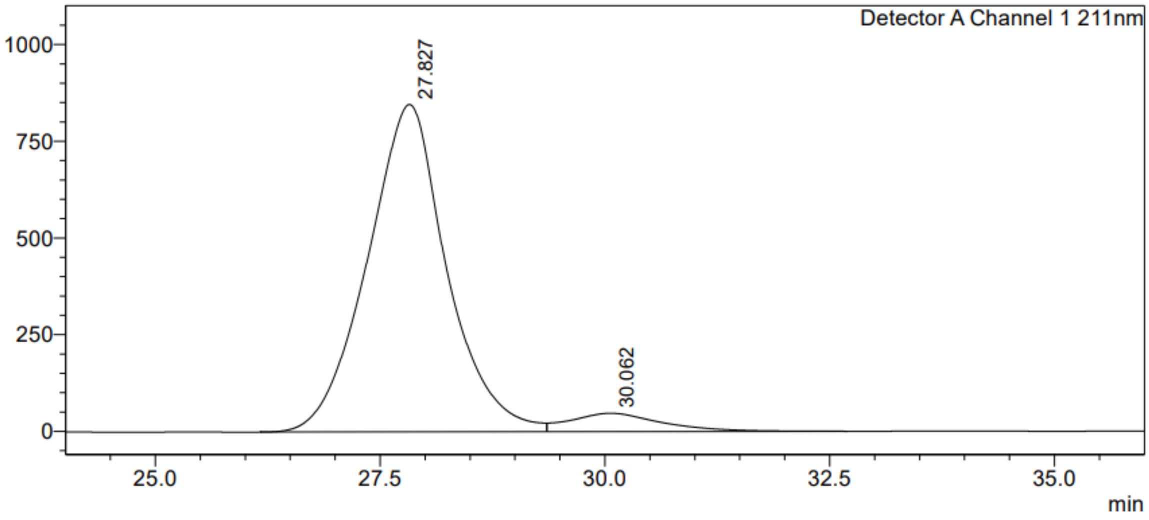

HPLC Data for *anti*-**51** and *syn*-**51**: Chiralpak AD-H (95:5 hexane : IPA, flow rate 1.0 mLmin^{-1}, 211 nm, 30 ℃) t_R(2*S*,3*S*): 20.3 min, t_R(2*R*,3*R*): 23.0 min, t_R(2*R*,3*S*): 29.8 min, t_R(2*S*,3*R*): 35.2 min, 95:5 er$_{ant,,}$ 93:7 er$_{syn..}$

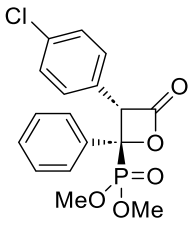

anti-**51**

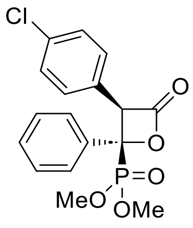

syn-**51**

Detector A Channel 1 211nm

Peak#	Ret. Time	Area%
1	20.205	41.664
2	22.894	41.639
3	29.724	8.323
4	35.063	8.374
Total		100.000

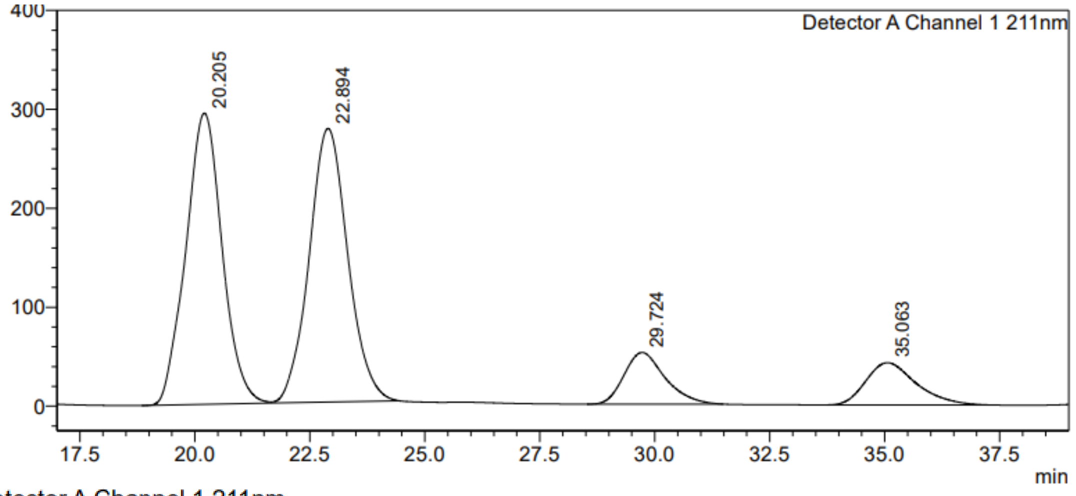

Detector A Channel 1 211nm

Peak#	Ret. Time	Area%
1	20.306	4.368
2	22.986	79.365
3	29.808	15.162
4	35.169	1.105
Total		100.000

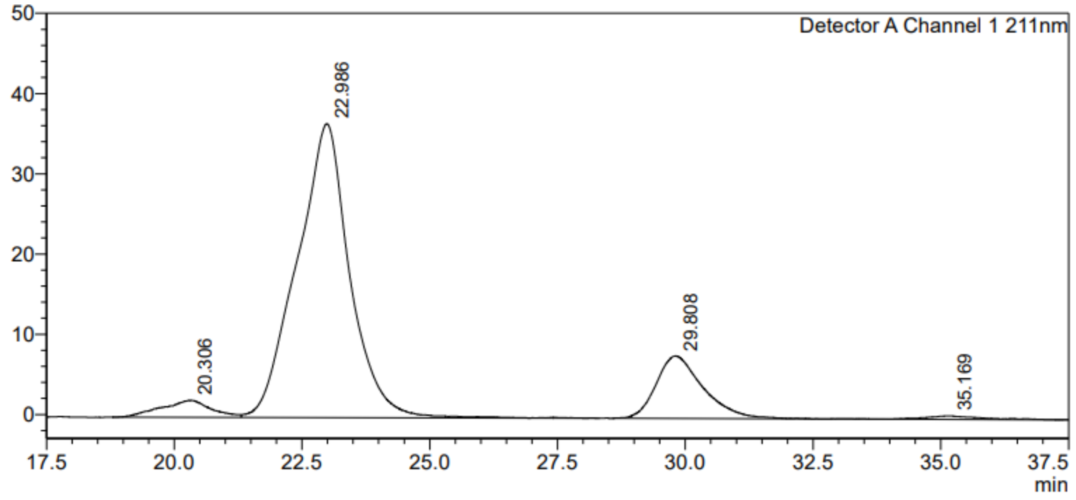

HPLC Data for *anti*-**52** and *syn*-**52**: Chiralcel OD-H (98:2 hexane : IPA, flow rate 1.0 mLmin^{-1}, 211 nm, 40 ℃) t_R(2*S*,3*S*): 32.0 min, t_R(2*R*,3*R*): 36.8 min, t_R(2*R*,3*S*): 49.0 min, t_R(2*S*,3*R*): 53.6 min, 95:5 er$_{anti}$, 82:18 er$_{syn}$.

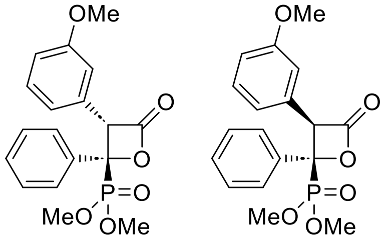

anti-**52** *syn*-**52**

PDA Ch1 211nm

Peak#	Ret. Time	Area%
1	31.801	43.251
2	37.265	44.536
3	49.174	6.046
4	53.819	6.167
Total		100.000

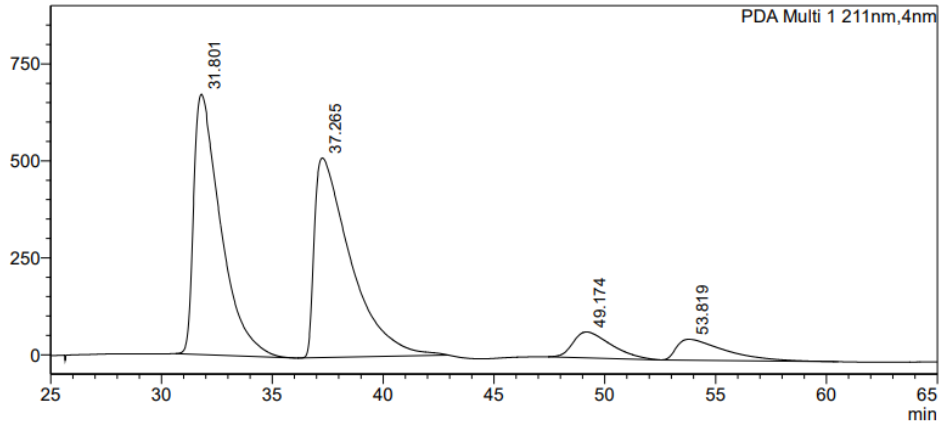

PDA Ch1 211nm

Peak#	Ret. Time	Area%
1	32.262	4.270
2	36.331	80.841
3	48.831	2.703
4	53.315	12.185
Total		100.000

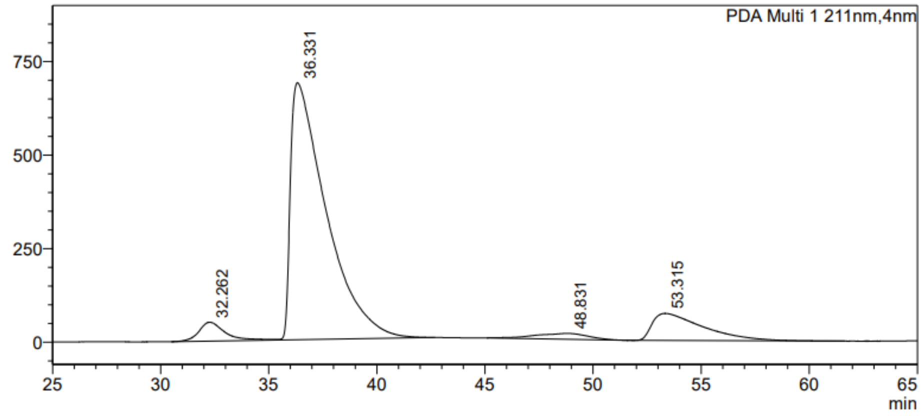

HPLC data for *anti*-**53** and *syn*-**53**: Chiralcel OD-H (95:5 hexane : IPA, flow rate 1.0 mLmin^{-1}, 220 nm, 40 ℃) t_R(2*S*,3*S*): 28.1 min, t_R(2*R*,3*R*): 33.4 min, t_R(2*S*,3*R*): 43.5 min, t_R(2*R*,3*S*): 51.5 min, 90:10 er$_{anti}$, 84:16 er$_{syn}$.

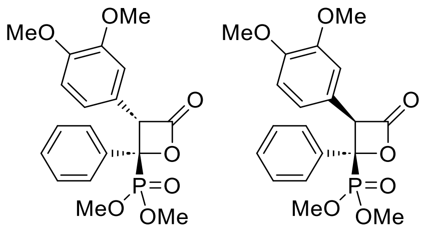

anti-**53** *syn*-**53**

PDA Ch2 220nm

Peak#	Ret. Time	Area%
1	28.057	44.413
2	33.424	44.351
3	43.517	5.651
4	51.521	5.586
Total		100.000

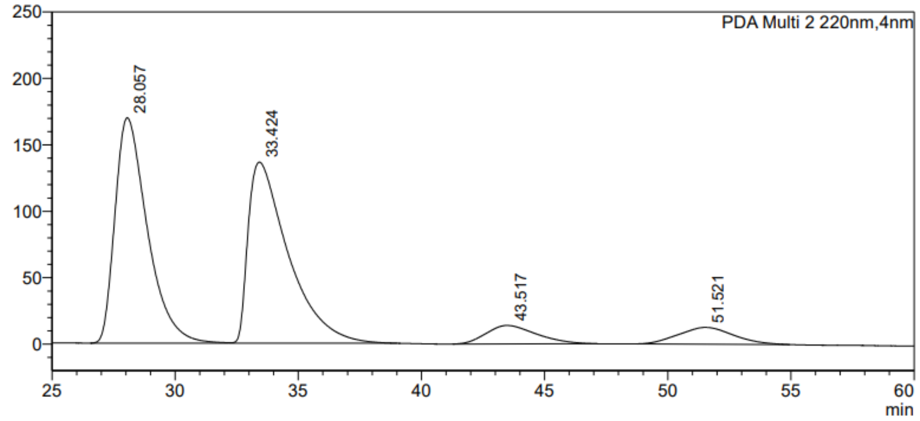

PDA Ch2 220nm

Peak#	Ret. Time	Area%
1	28.501	9.101
2	32.849	79.693
3	43.079	9.415
4	51.875	1.790
Total		100.000

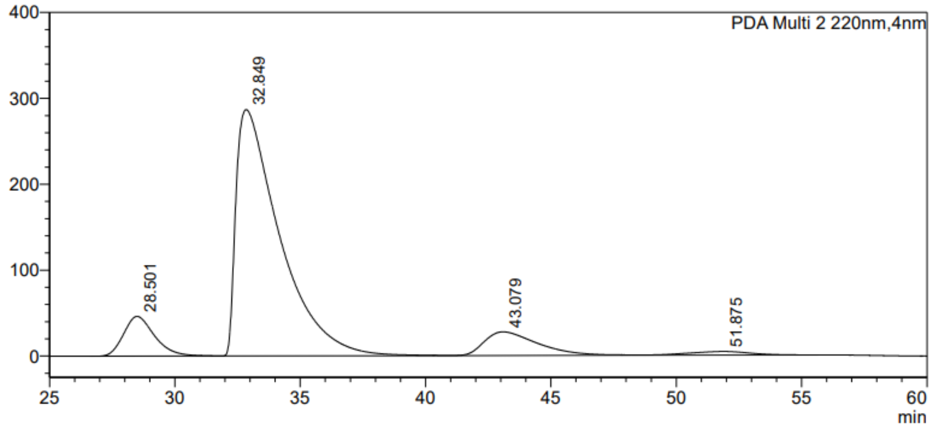

HPLC Data for *anti*-**54**: Chiralpak AD-H (97:3 hexane : IPA, flow rate 1.0 mLmin^{-1}, 220 nm, 30 °C) t_R(2*S*,3*S*): 16.6 min, t_R(2*R*,3*R*): 20.0 min, 95:5 er$_{anti.}$

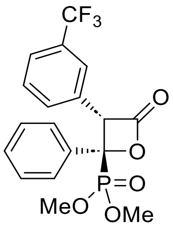

anti-**54**

PDA Ch2 220nm

Peak#	Ret. Time	Area%
1	16.623	29.992
2	19.981	30.816
3	23.586	19.897
4	25.703	19.295
Total		100.000

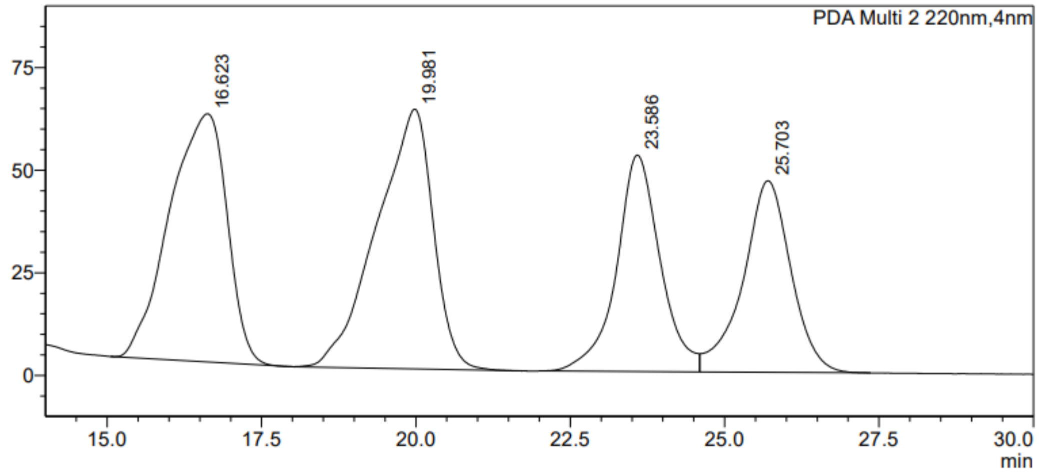

PDA Ch2 220nm

Peak#	Ret. Time	Area%
1	16.216	5.290
2	19.769	73.762
3	23.760	19.644
4	25.649	1.304
Total		100.000

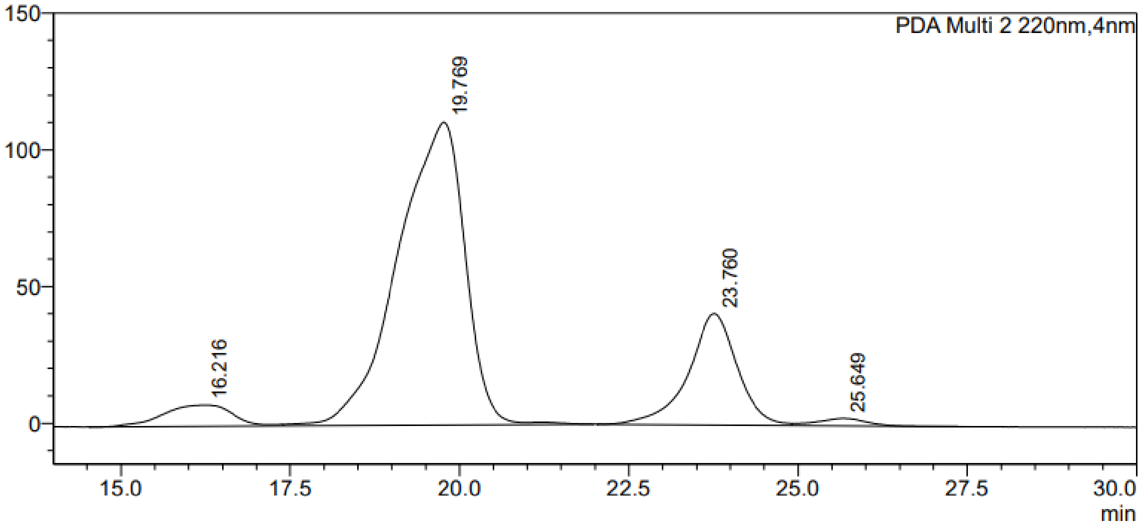

HPLC Data for *syn*-**54**: Chiralpak AD-H (97:3 hexane : IPA, flow rate 1.0 mLmin^{-1}, 220 nm, 30 °C) t_R(2*R*,3*S*): 23.6 min, t_R(2*S*,3*R*): 25.7 min, 93:7 er$_{syn.}$

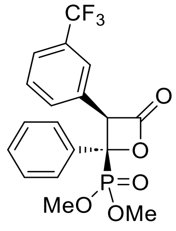

anti-**54**

PDA Ch2 220nm

Peak#	Ret. Time	Area%
1	16.623	29.992
2	19.981	30.816
3	23.586	19.897
4	25.703	19.295
Total		100.000

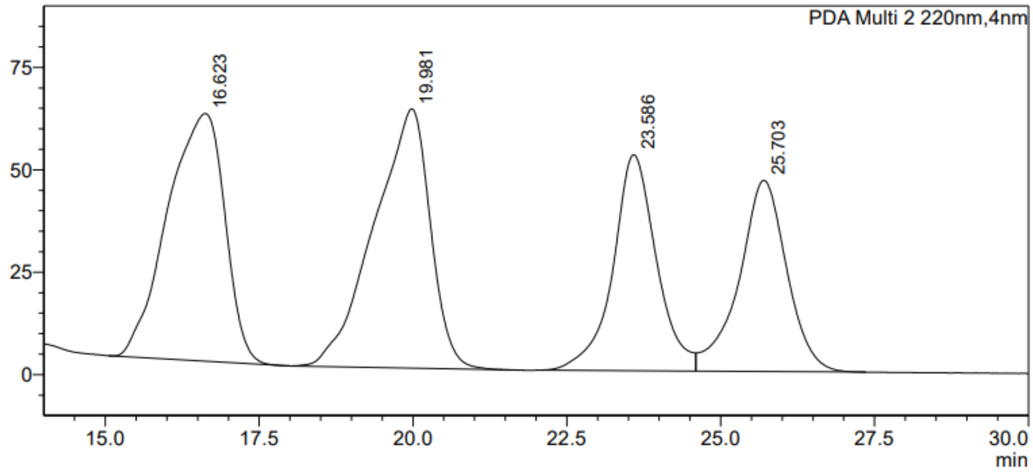

PDA Ch2 220nm

Peak#	Ret. Time	Area%
1	22.642	93.310
2	24.885	6.690
Total		100.000

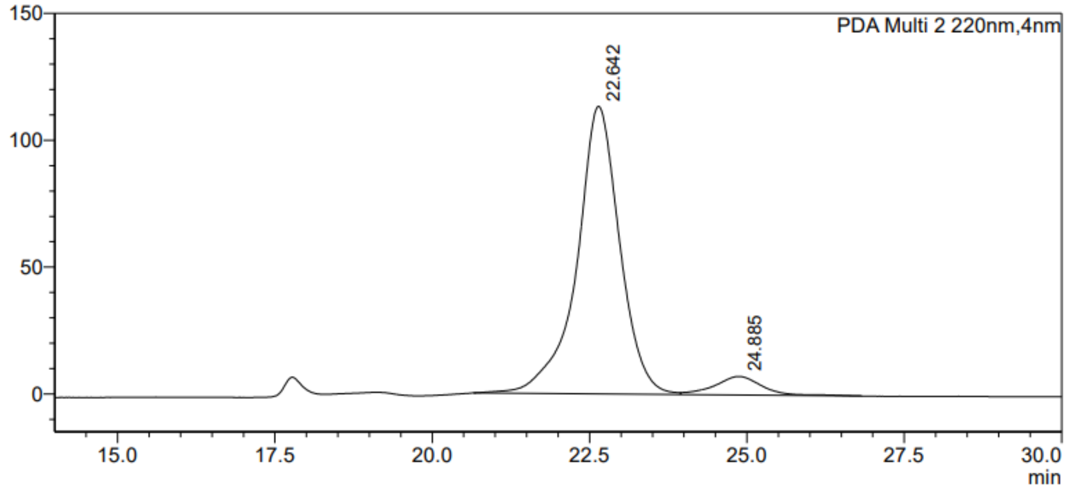

HPLC Data for *anti*-**55**: Chiralpak AD-H (95:5 hexane : IPA, flow rate 1.0 mLmin^{-1}, 211 nm, 30 °C) t_R(2*S*,3*S*): 18.1 min, t_R(2*R*,3*R*): 30.3 min, 98:2 er$_{anti}$.

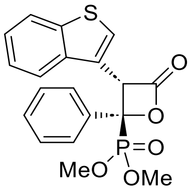

anti-**55**

Detector A Channel 1 211nm

Peak#	Ret. Time	Area%
1	18.132	49.872
2	30.401	50.128
Total		100.000

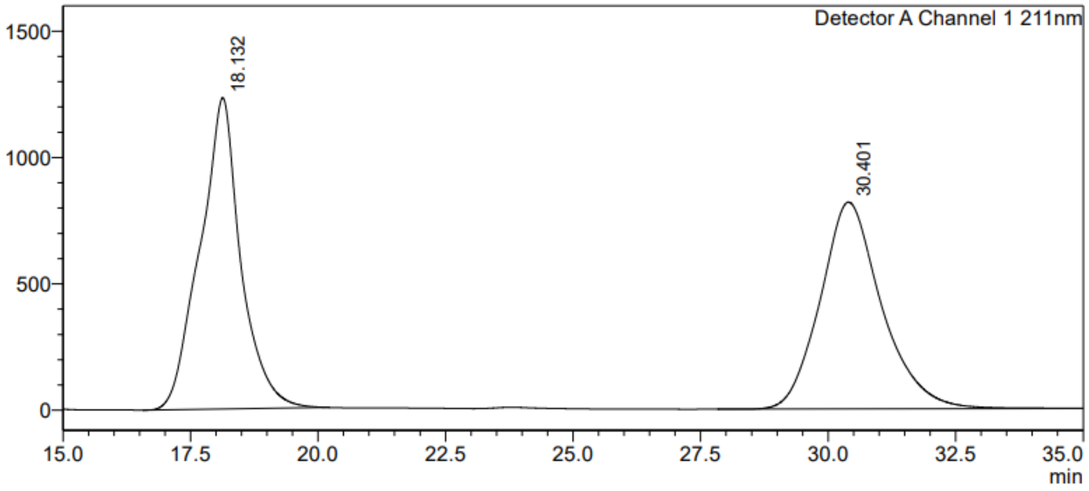

Detector A Channel 1 211nm

Peak#	Ret. Time	Area%
1	18.122	2.116
2	30.347	97.884
Total		100.000

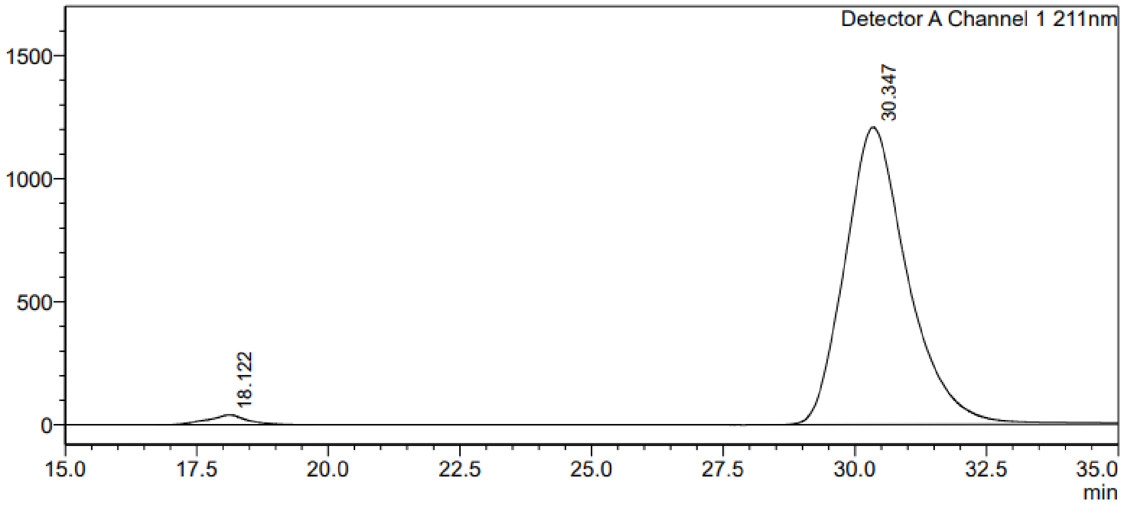

HPLC Data for *syn*-**55**: Chiralpak AS-H (97:3 hexane : IPA, flow rate 1.0 mLmin^{-1}, 211 nm, 40 ℃) t_R(2*S*,3*R*): 16.7 min, t_R(2*R*,3*S*): 21.6 min, 98:2 er$_{syn}$.

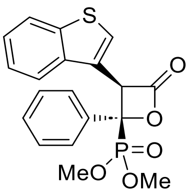

syn-**55**

Detector A Channel 1 211nm

Peak#	Ret. Time	Area%
1	16.544	50.537
2	21.693	49.463
Total		100.000

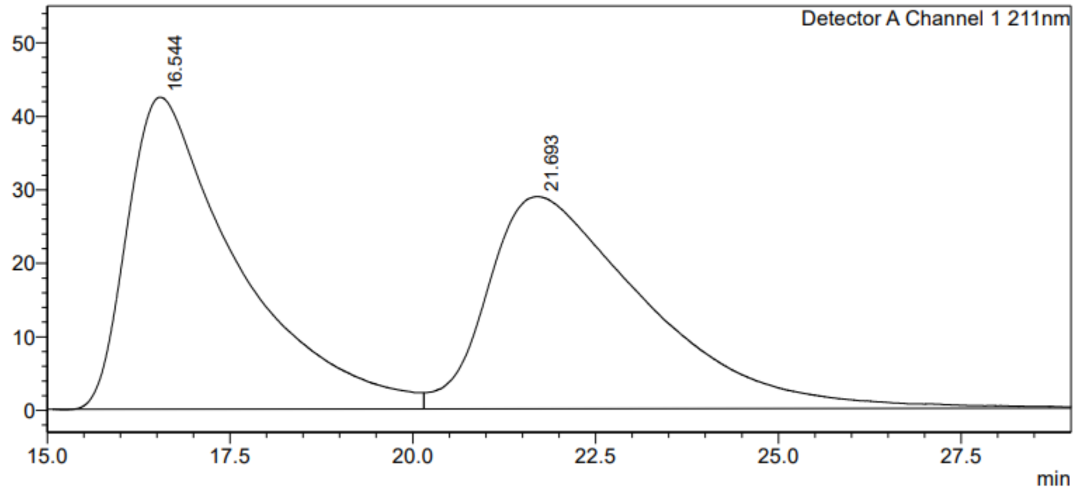

Detector A Channel 1 211nm

Peak#	Ret. Time	Area%
1	16.663	2.361
2	21.600	97.639
Total		100.000

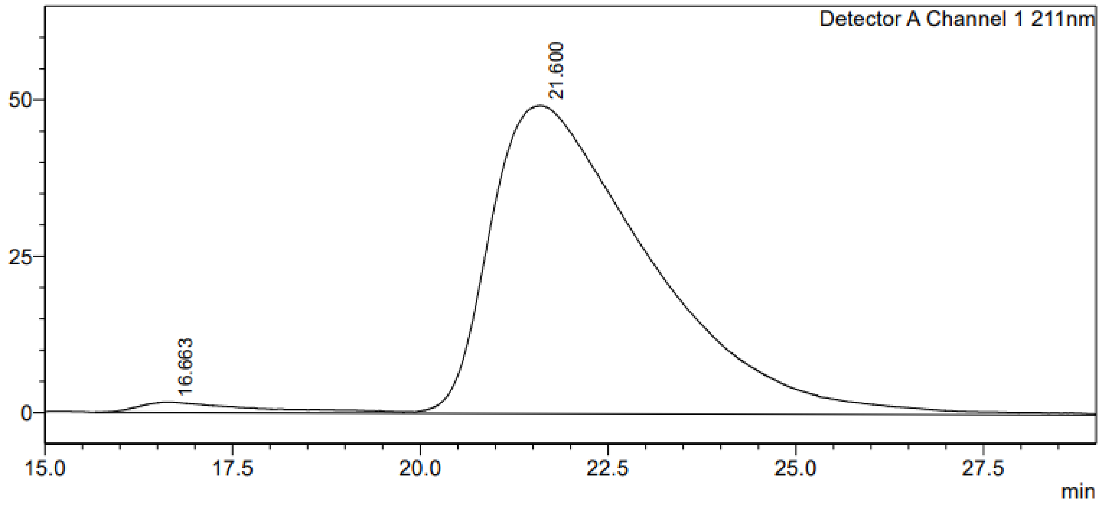

HPLC Data for *anti*-**56**: Chiralcel OD-H (95:5 hexane : IPA, flow rate 1.0 mLmin^{-1}, 211 nm, 30 ℃) t_R(2*S*,3*S*): 20.1 min, t_R(2*R*,3*R*): 26.8 min, 90:10 er$_{anti}$.

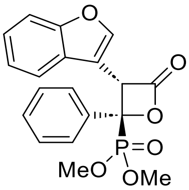

anti-**56**

Detector A Channel 1 211nm

Peak#	Ret. Time	Area%
1	19.860	49.898
2	26.602	50.102
Total		100.000

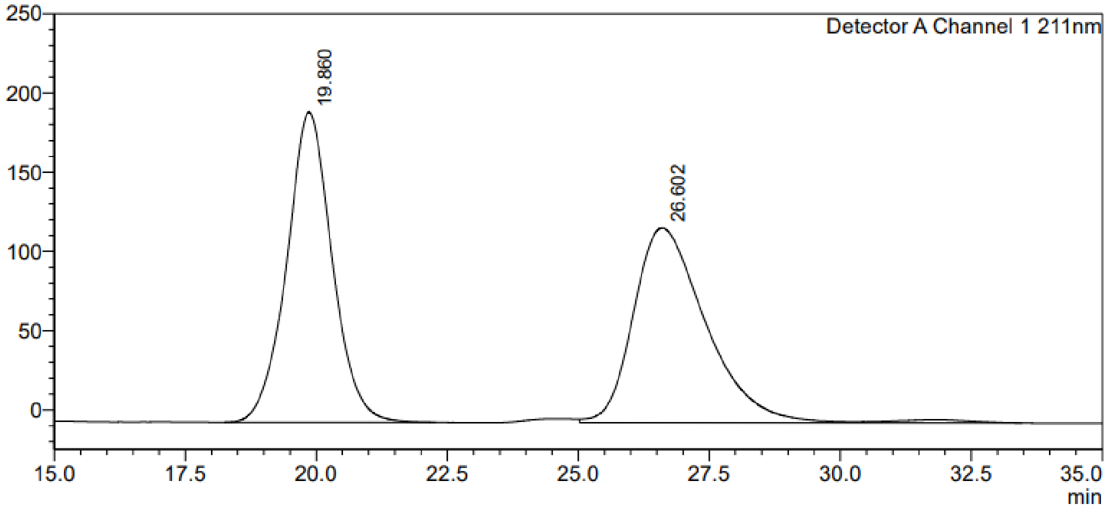

Detector A Channel 1 211nm

Peak#	Ret. Time	Area%
1	20.103	10.274
2	26.813	89.726
Total		100.000

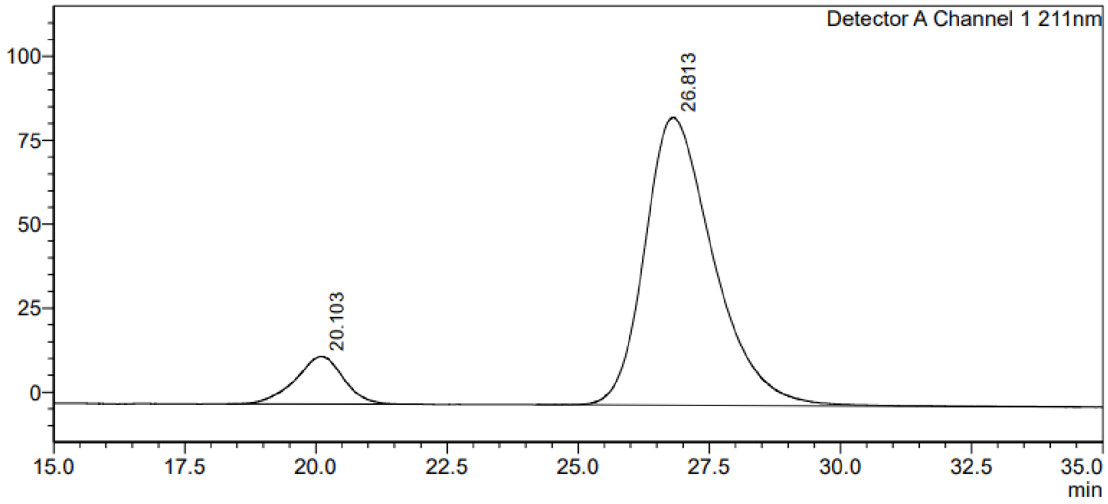

HPLC Data for *syn*-**56**: Chiralcel OD-H (95:5 hexane : IPA, flow rate 1.0 mLmin^{-1}, 211 nm, 30 ℃) t_R(2*R*,3*S*): 24.7 min, t_R(2*S*,3*R*): 31.3 min, 90:10 er$_{syn}$.

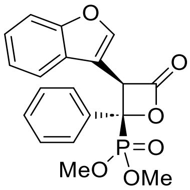

syn-**56**

Detector A Channel 1 211nm

Peak#	Ret. Time	Area%
1	24.509	49.694
2	31.350	50.306
Total		100.000

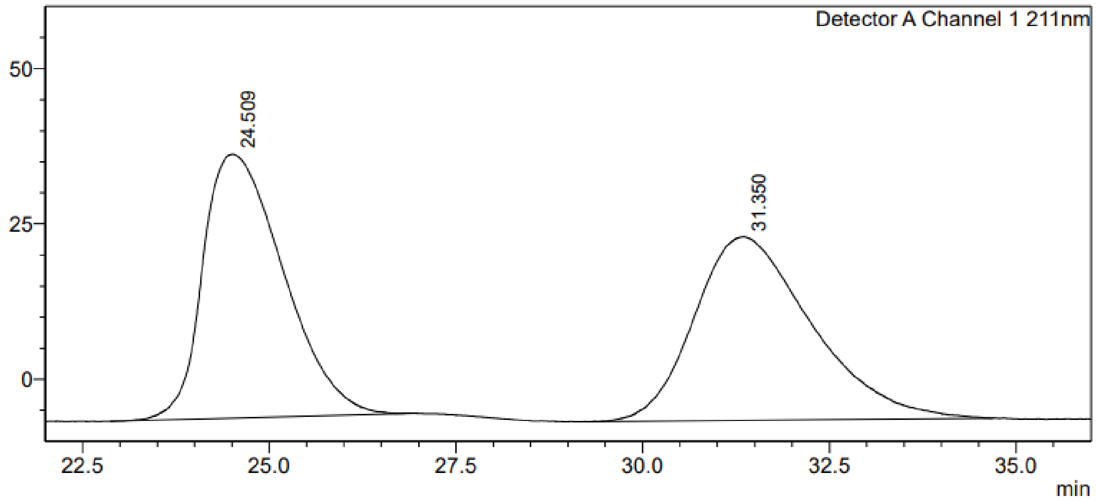

Detector A Channel 1 211nm

Peak#	Ret. Time	Area%
1	24.721	90.184
2	31.325	9.816
Total		100.000

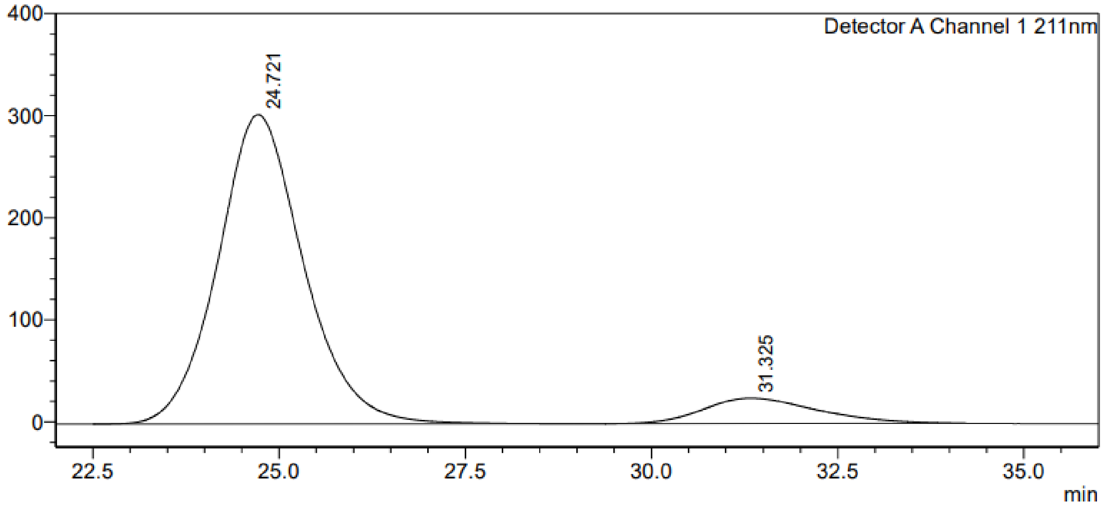

HPLC Data for *anti*-**57**: Chiralcel OJ-H (97:3 hexane : IPA, flow rate 1.5 mLmin^{-1}, 211 nm, 30 °C) t_R(2*S*,3*S*): 28.0 min, t_R(2*R*,3*R*): 33.1 min, 97:3 er$_{anti}$.

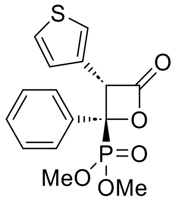

anti-**57**

Peak#	Ret. Time	Area%
1	27.448	49.271
2	33.189	50.729
Total		100.000

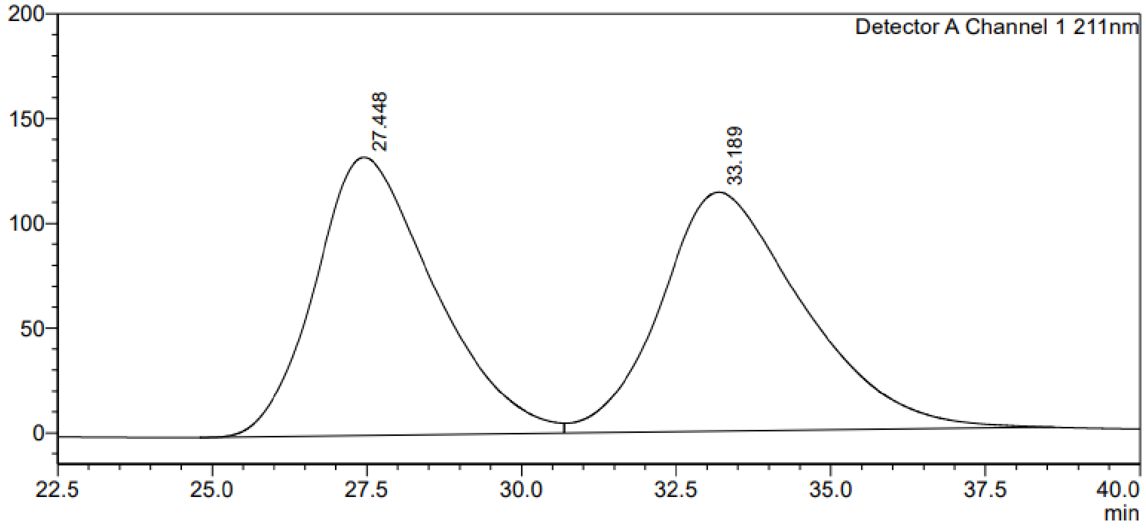

Detector A Channel 1 211nm

Peak#	Ret. Time	Area%
1	28.005	2.825
2	33.143	97.175
Total		100.000

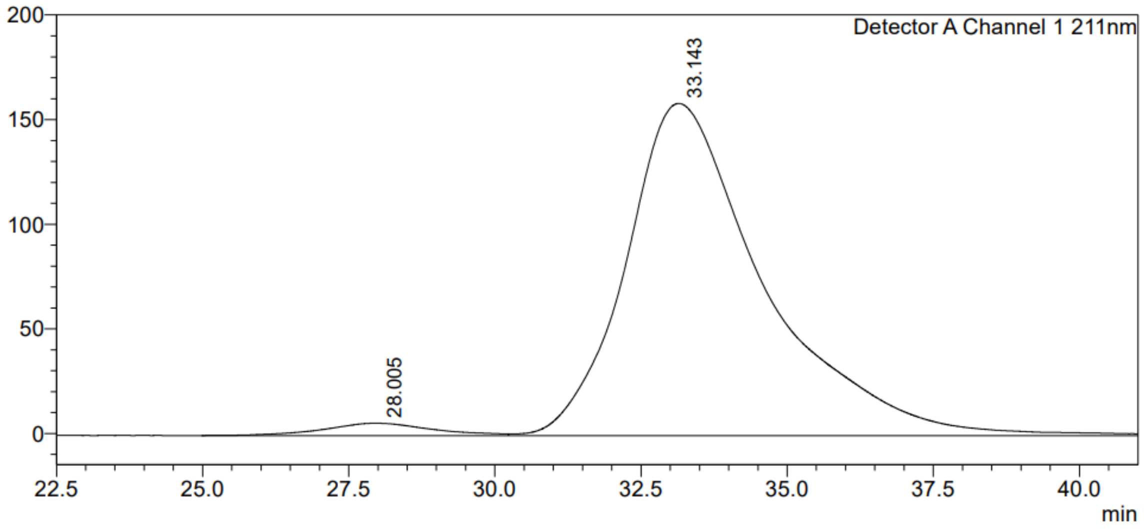

HPLC Data for *syn*-**57**: Chiralpak AD-H (95:5 hexane : IPA, flow rate 1.0 mLmin^{-1}, 211 nm, 30 °C) t_R(2*S*,3*R*): 31.4 min, t_R(2*R*,3*S*): 41.6 min, 97:3 er$_{syn}$.

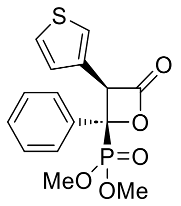

syn-**57**

Detector A Channel 1 211nm

Peak#	Ret. Time	Area%
1	30.758	49.375
2	41.121	50.625
Total		100.000

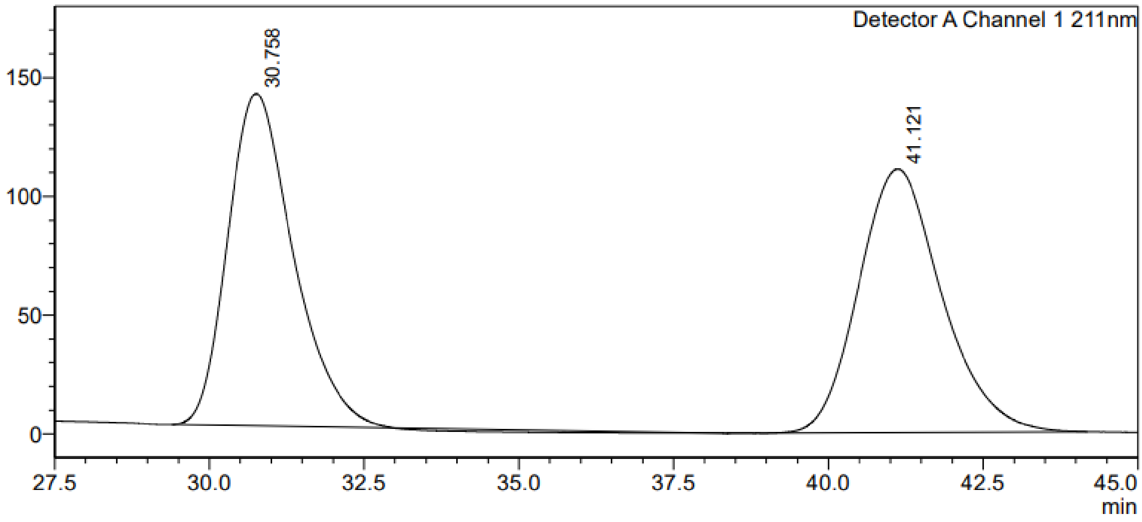

Detector A Channel 1 211nm

Peak#	Ret. Time	Area%
1	31.355	2.880
2	41.584	97.120
Total		100.000

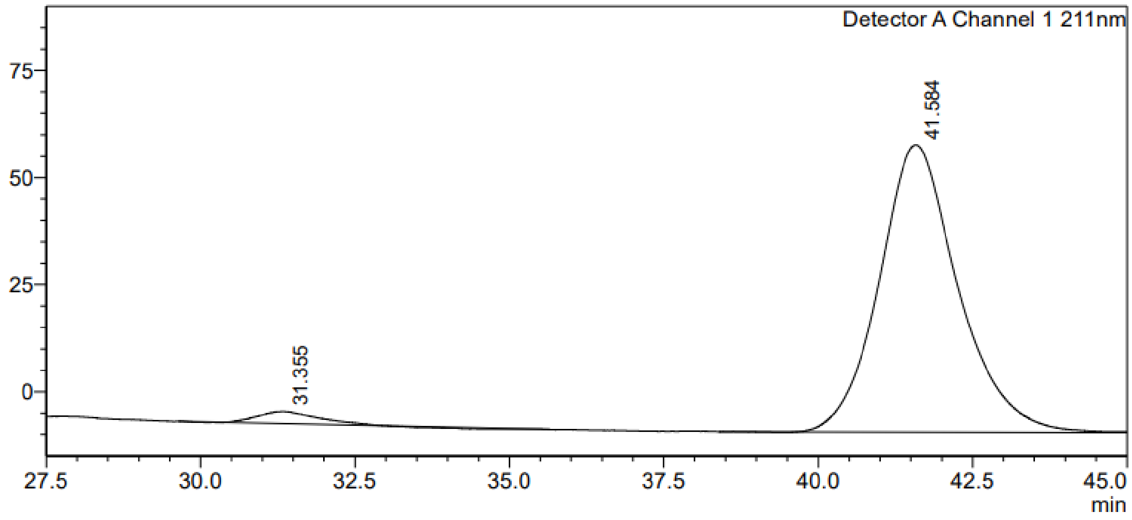

HPLC Data for *anti*-**58**: Chiralpak AD-H (95:5 hexane : IPA, flow rate 1.0 mLmin^{-1}, 211 nm, 30 °C) t_R(2*S*,3*S*): 17.1 min, t_R(2*R*,3*R*): 19.6 min, 99:1 er$_{anti}$.

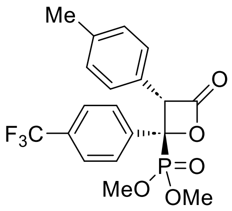

anti-**58**

Detector A Channel 1 211nm

Peak#	Ret. Time	Area%
1	18.167	50.115
2	20.198	49.885
Total		100.000

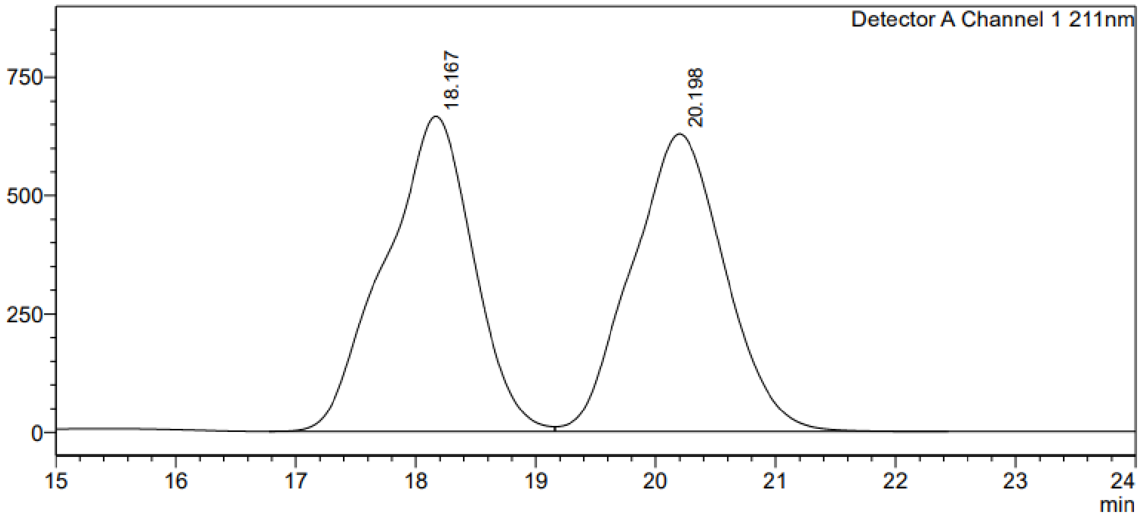

Detector A Channel 1 211nm

Peak#	Ret. Time	Area%
1	17.808	1.219
2	19.554	98.781
Total		100.000

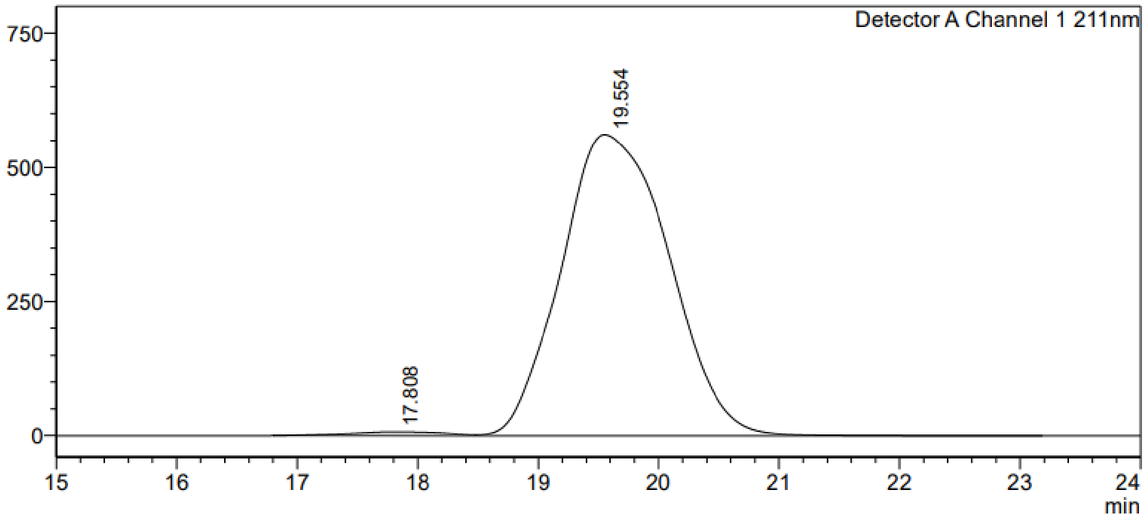

HPLC Data for *syn*-**58**: Chiralpak AD-H (95:5 hexane : IPA, flow rate 1.0 mLmin^{-1}, 211 nm, 30 ℃) t_R(2*S*,3*S*): 17.9 min, t_R(2*R*,3*R*): 28.6 min, 98:2 er$_{anti}$.

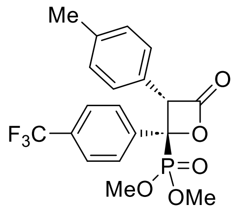

syn-**58**

Detector A Channel 1 211nm

Peak#	Ret. Time	Area%
1	17.721	50.081
2	28.608	49.919
Total		100.000

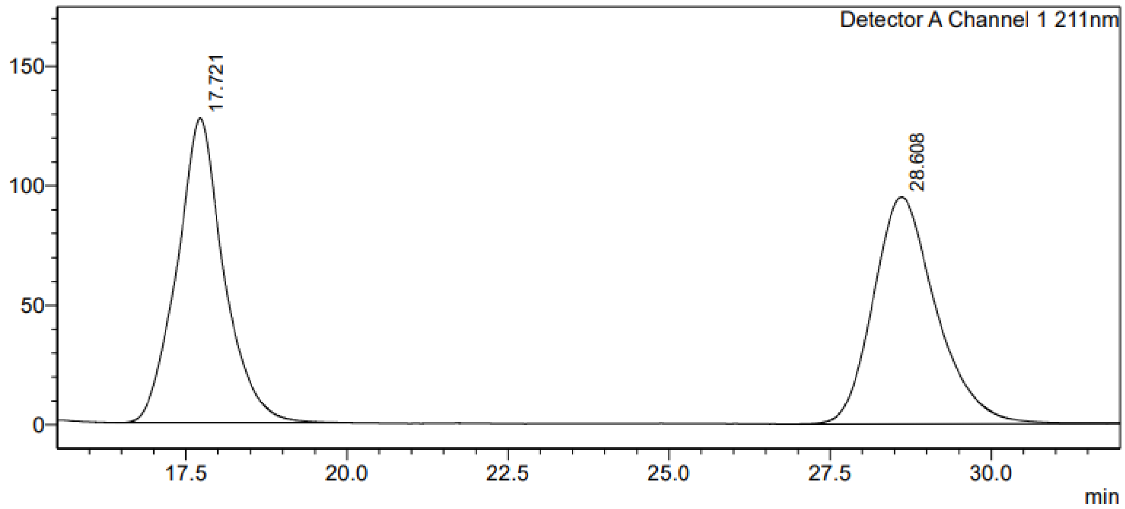

Detector A Channel 1 211nm

Peak#	Ret. Time	Area%
1	17.875	1.964
2	28.609	98.036
Total		100.000

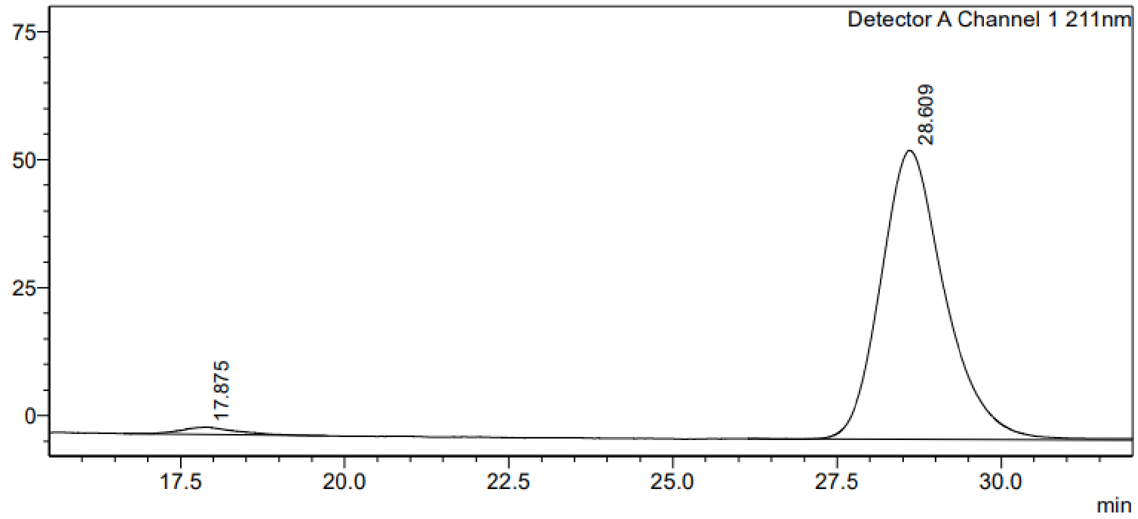

HPLC Data for *anti*-**59**: Chiralpak AD-H (95:5 hexane : IPA, flow rate 1.0 mLmin^{-1}, 220 nm, 30 ℃) t_R(2*S*,3*S*): 17.5 min, t_R(2*R*,3*R*): 22.5 min, 98:2 er$_{anti}$.

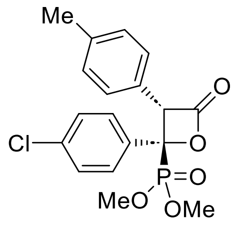

anti-**59**

PDA Ch2 220nm

Peak#	Ret. Time	Area%
1	17.553	50.041
2	22.536	49.959
Total		100.000

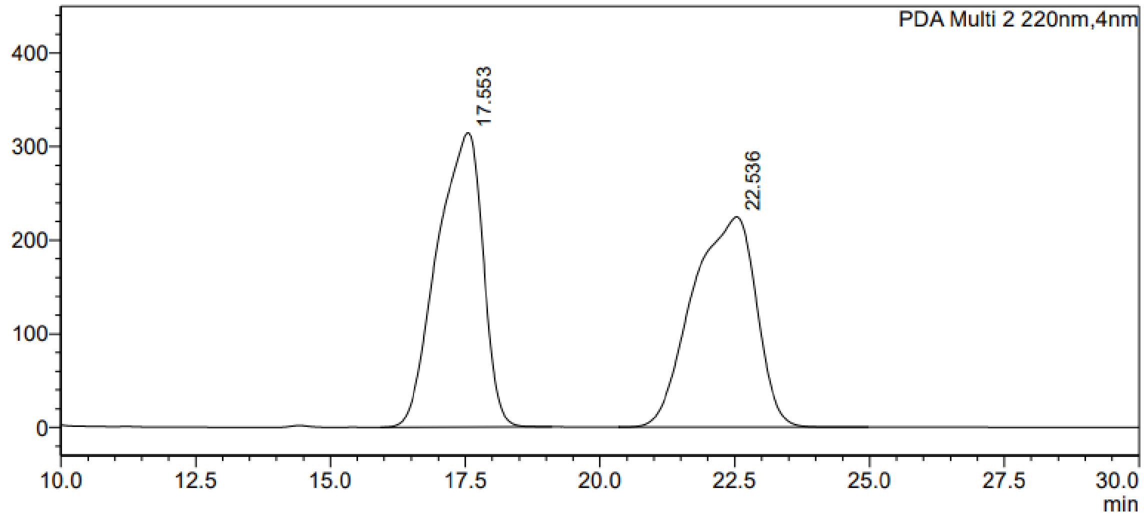

PDA Ch2 220nm

Peak#	Ret. Time	Area%
1	17.504	1.969
2	22.512	98.031
Total		100.000

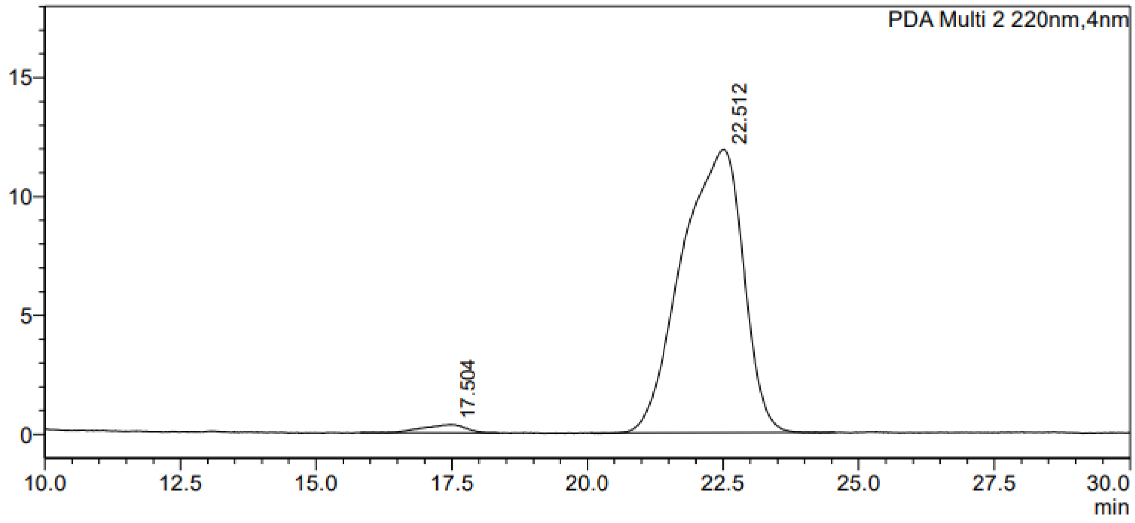

HPLC Data for *syn*-**60** : Chiralcel OD-H (95:5 hexane : IPA, flow rate 1.0 mLmin^{-1}, 211 nm, 30 °C) t_R(2*S*,3*S*): 15.4 min, t_R(2*R*,3*R*): 20.3 min, 98:2 er$_{anti}$.

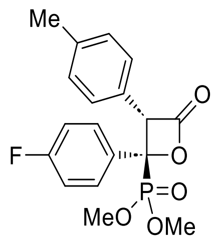

anti-**60**

Detector A Channel 1 211nm

Peak#	Ret. Time	Area%
1	15.125	49.701
2	20.154	50.299
Total		100.000

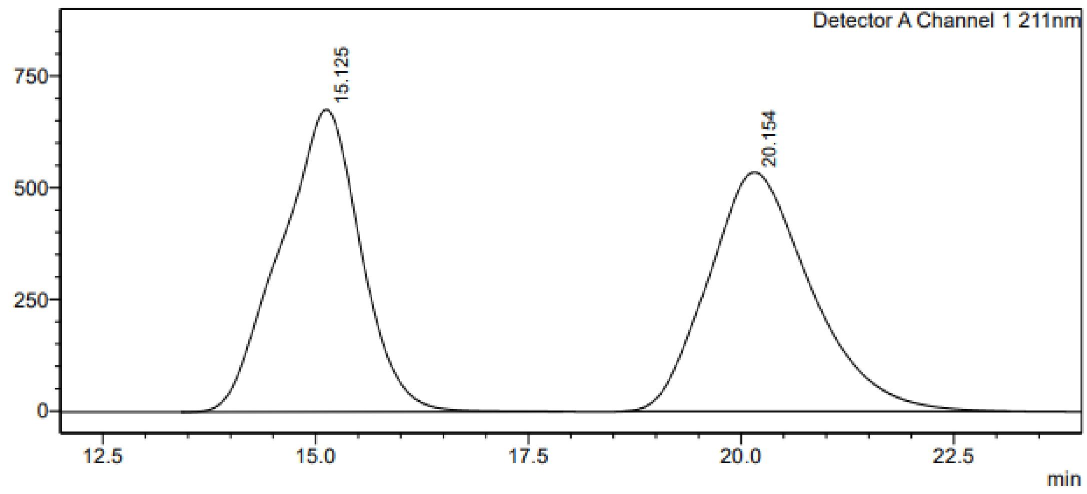

Detector A Channel 1 211nm

Peak#	Ret. Time	Area%
1	15.414	1.574
2	20.328	98.426
Total		100.000

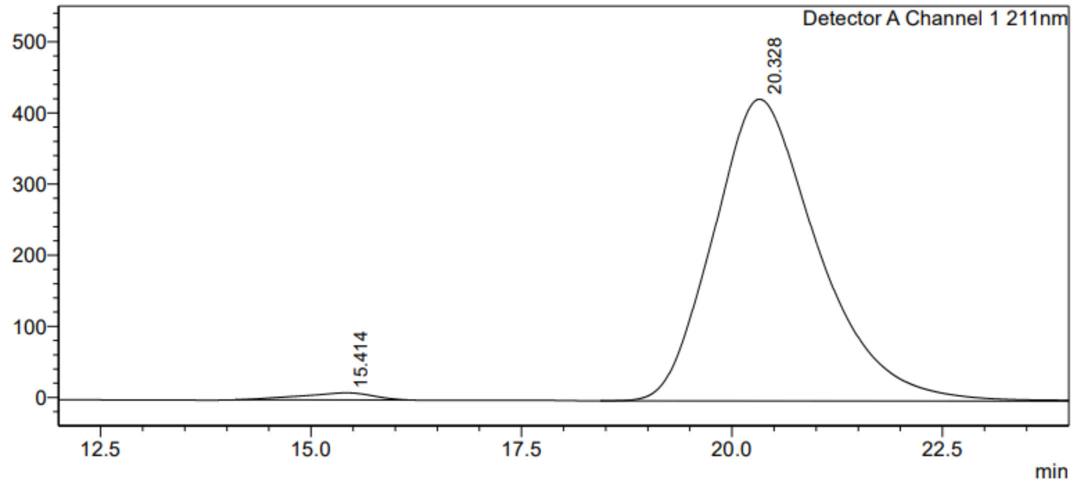

HPLC Data for *anti*-**61**: Chiralpak AD-H (95:5 hexane : IPA, flow rate 1.0 mLmin^{-1}, 220 nm, 30 °C) t_R(2*S*,3*S*): 18.8 min, t_R(2*R*,3*R*): 23.3 min, 94:6 er$_{anti}$.

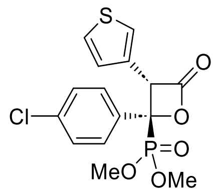

anti-**61**

PDA Ch2 220nm

Peak#	Ret. Time	Area%
1	18.809	49.143
2	23.447	50.857
Total		100.000

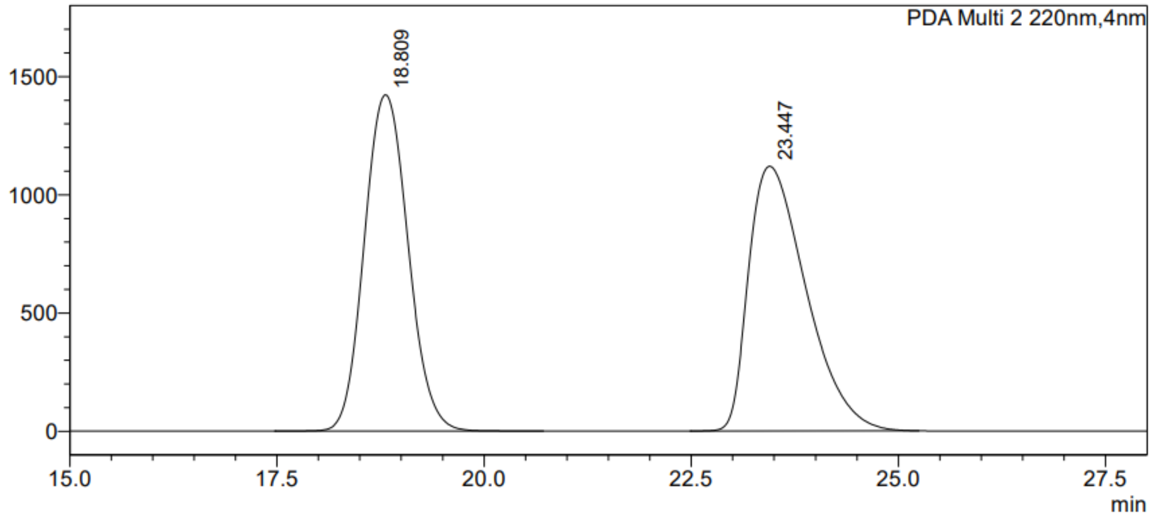

PDA Ch2 220nm

Peak#	Ret. Time	Area%
1	18.767	5.346
2	23.306	94.654
Total		100.000

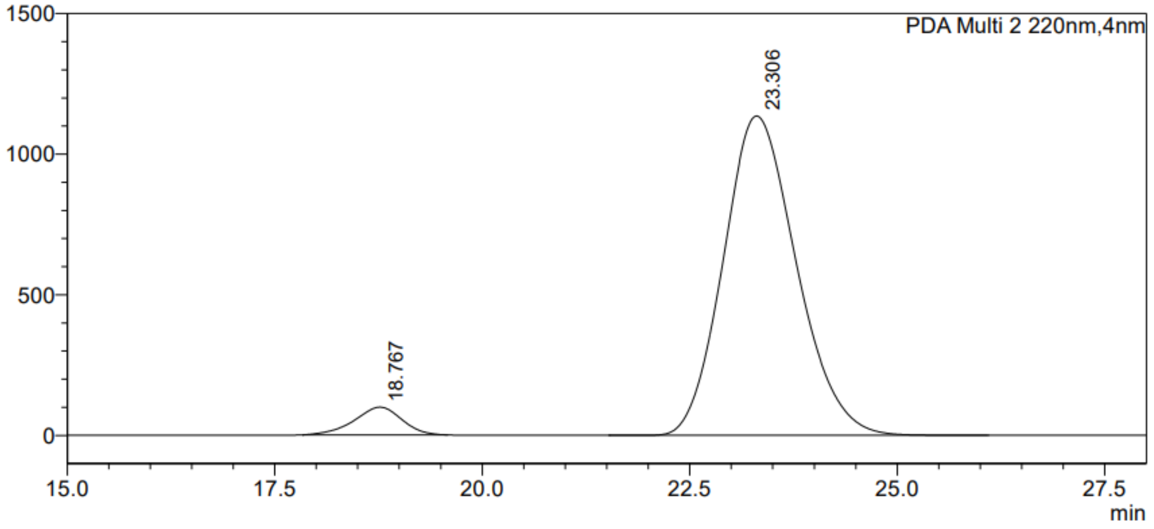

HPLC Data for *syn*-**61**: Chiralpak IA (95:5 hexane : IPA, flow rate 1.0 mLmin^{-1}, 220 nm, 30 ℃) t_R(2*R*,3*S*): 31.5 min, t_R(2*S*,3*R*): 47.1 min, 94:6 er$_{syn}$.

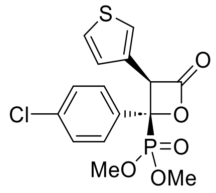

syn-**61**

PDA Ch2 220nm

Peak#	Ret. Time	Area%
1	31.138	50.082
2	46.983	49.918
Total		100.000

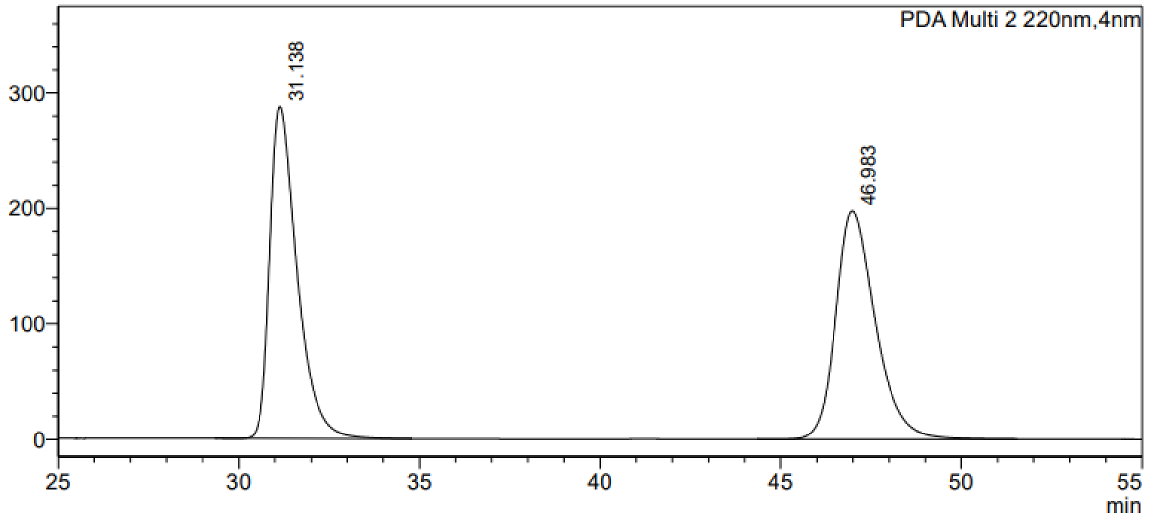

PDA Ch2 220nm

Peak#	Ret. Time	Area%
1	31.532	5.536
2	47.071	94.464
Total		100.000

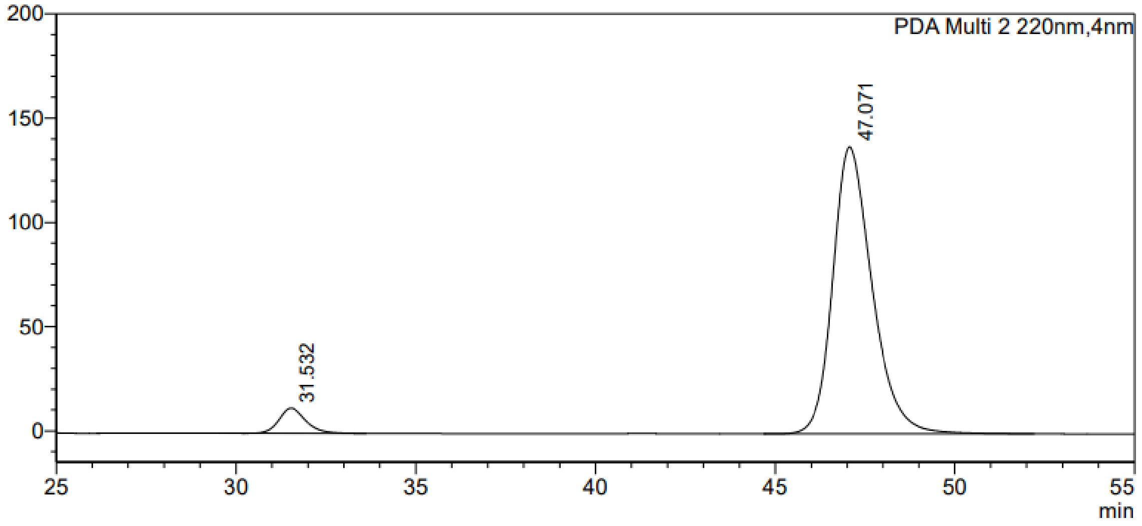

HPLC Data for *anti*-**62**: Chiralpak AS-H (95:5 hexane : IPA, flow rate 1.0 mLmin^{-1}, 211 nm, 30 ℃) t_R(2*S*,3*S*): 18.3 min, t_R(2*R*,3*R*): 20.7 min, 97:3 er$_{anti}$.

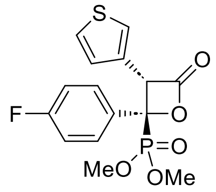

anti-**62**

PDA Ch1 211nm

Peak#	Ret. Time	Area%
1	18.263	50.344
2	20.804	49.656
Total		100.000

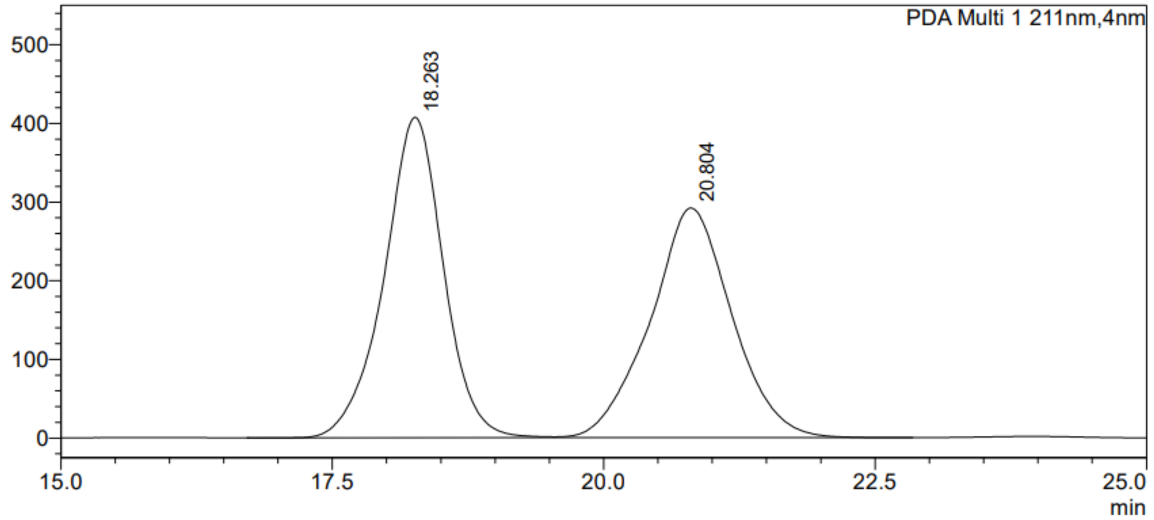

PDA Ch1 211nm

Peak#	Ret. Time	Area%
1	18.311	3.008
2	20.708	96.992
Total		100.000

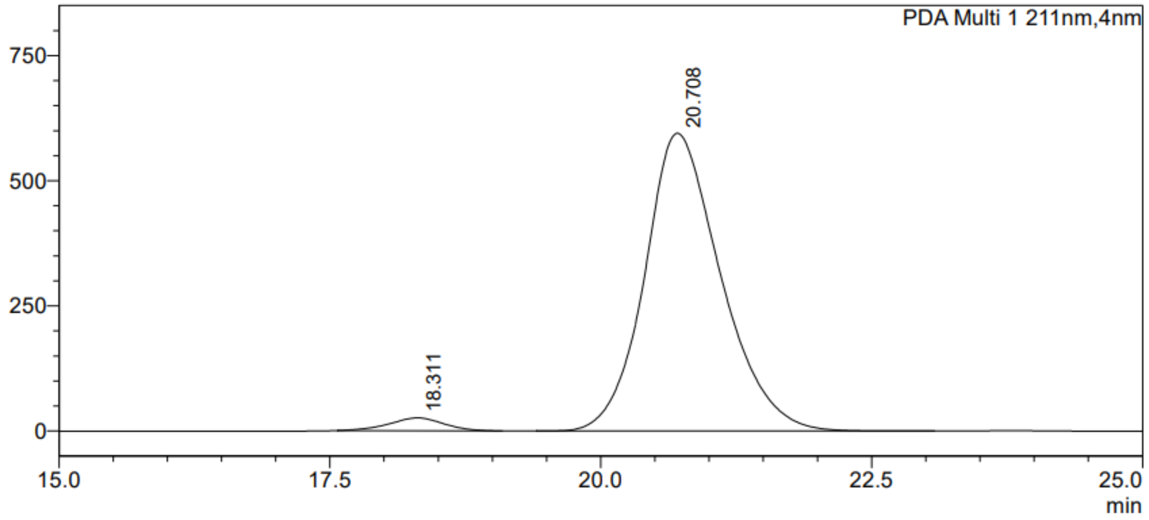

HPLC Data for *syn*-**62**: Chiralpak AD-H (94:6 hexane : IPA, flow rate 1.0 mLmin^{-1}, 220 nm, 30 ℃) t_R(2*S*,3*R*): 24.5 min, t_R(2*R*,3*S*): 41.2 min, 96:3 er$_{syn}$.

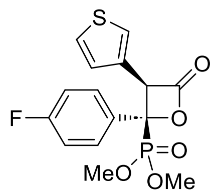

syn-**62**

PDA Ch2 220nm

Peak#	Ret. Time	Area%
1	24.520	49.988
2	41.159	50.012
Total		100.000

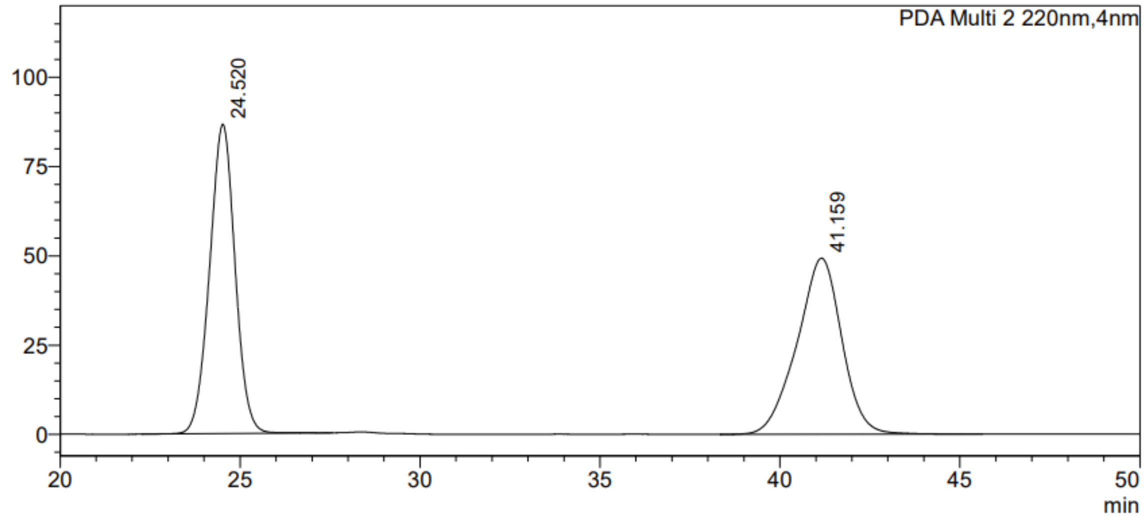

PDA Ch2 220nm

Peak#	Ret. Time	Area%
1	24.518	3.670
2	41.167	96.330
Total		100.000

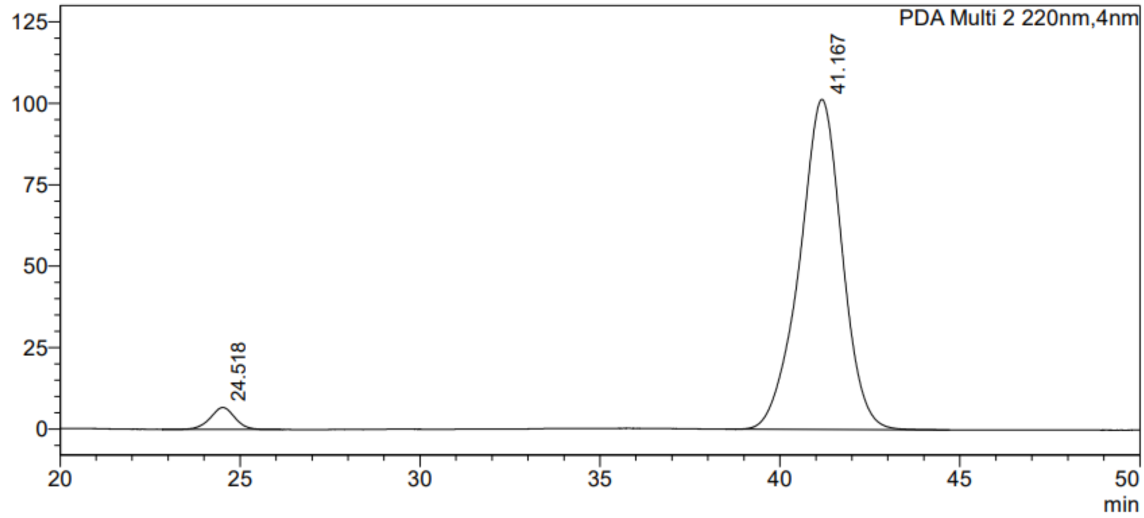

HPLC Data for *anti*-**63**: Chiralpak IA (97:3 hexane : IPA, flow rate 1.0 mLmin^{-1}, 211 nm, 30 ℃) t_R(2*R*,3*R*): 17.1 min, t_R(2*S*,3*S*): 18.6 min, 99:1 er$_{anti}$.

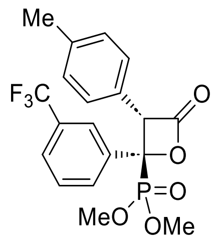

anti-**63**

PDA Ch1 211nm

Peak#	Ret. Time	Area%
1	17.266	49.722
2	18.713	50.278
Total		100.000

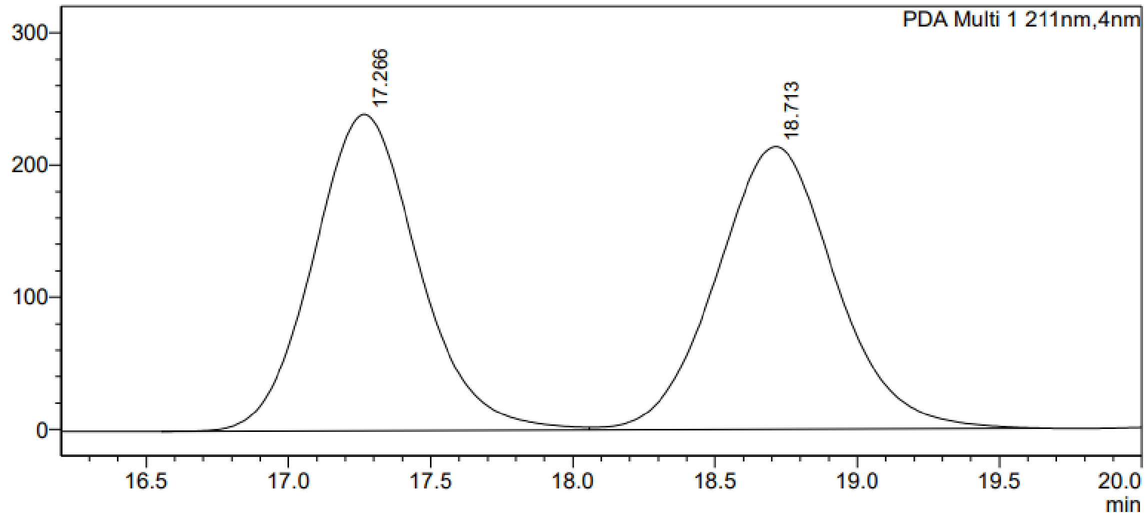

PDA Ch1 211nm

Peak#	Ret. Time	Area%
1	17.070	99.066
2	18.559	0.934
Total		100.000

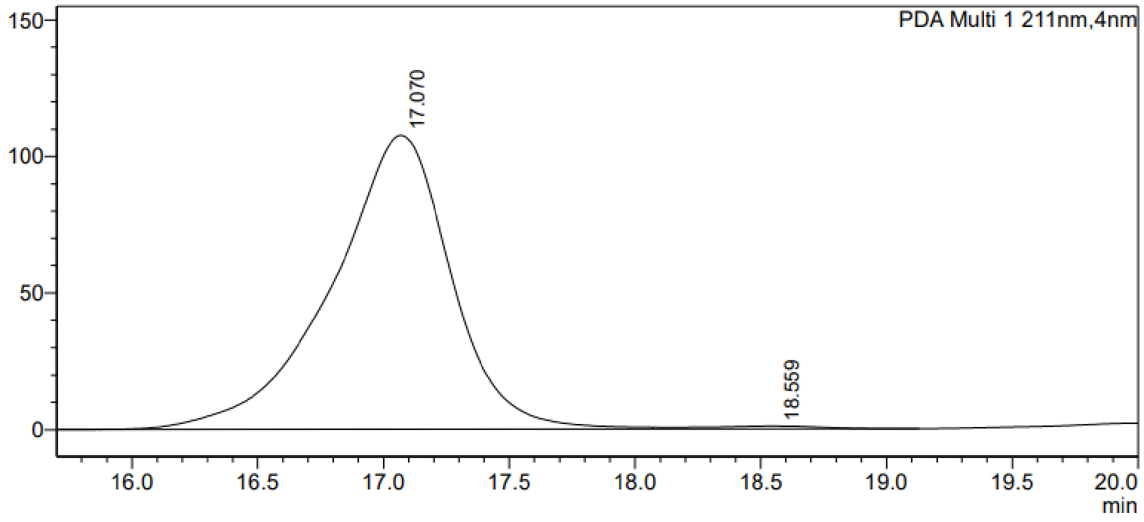

HPLC Data for *syn*-**63**: Chiralpak AD-H (96:4 hexane : IPA, flow rate 1.0 mLmin^{-1}, 211 nm, 30 ℃) t_R(2*R*,3*S*): 16.5 min, t_R(2*S*,3*R*): 19.1 min, >99:1 er$_{syn}$.

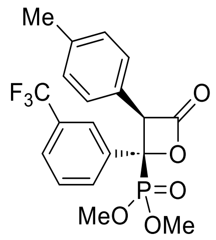

syn-**63**

Detector A Channel 1 211nm

Peak#	Ret. Time	Area%
1	16.962	49.991
2	19.727	50.009
Total		100.000

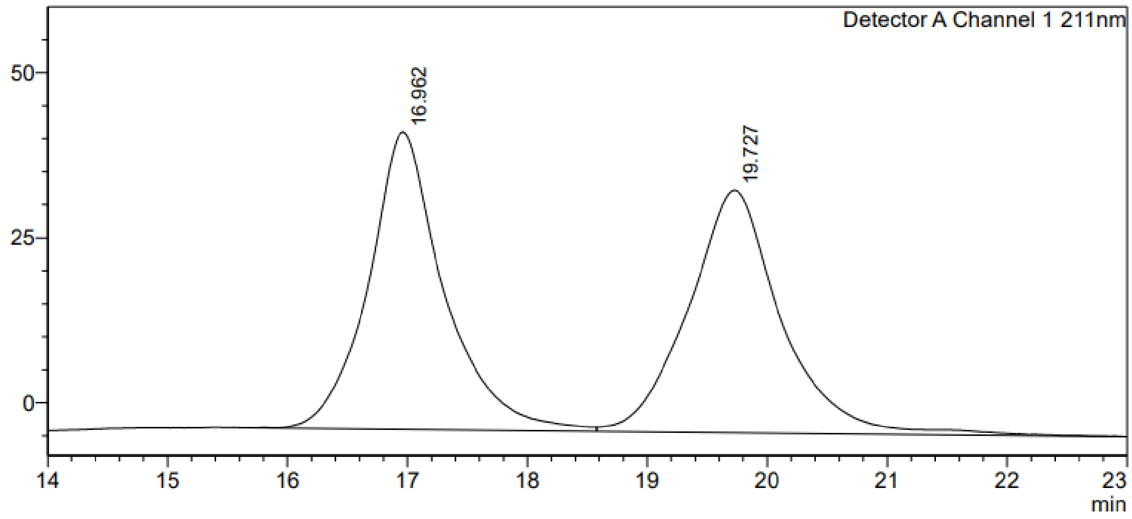

Detector A Channel 1 211nm

Peak#	Ret. Time	Area%
1	16.510	-0.141
2	19.141	100.141
Total		100.000

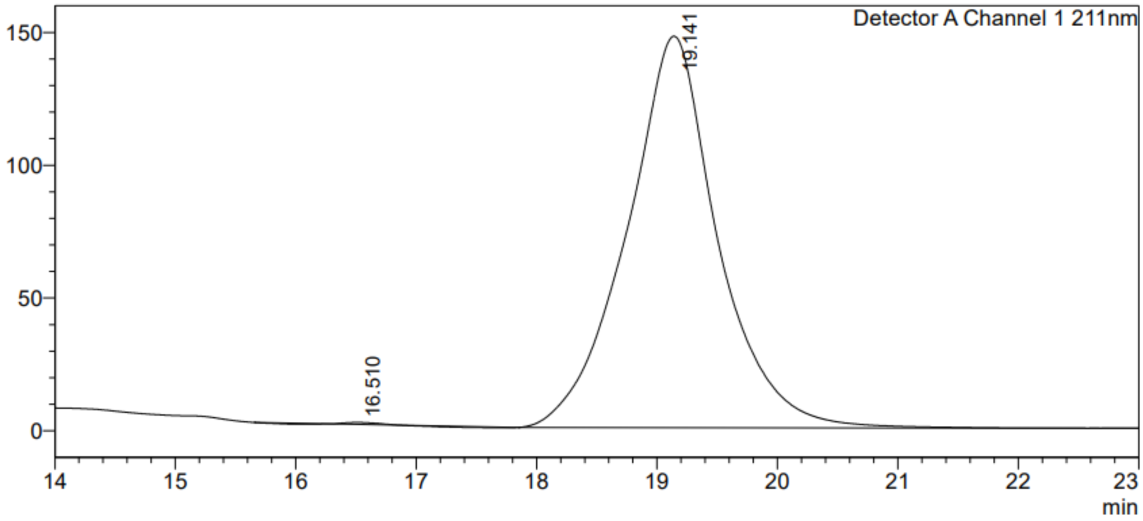

HPLC Data for *anti*-**64**: Chiralcel OD-H (95:5 hexane : IPA, flow rate 1.0 mLmin^{-1}, 211 nm, 30 °C) t_R(2*S*,3*S*): 14.7 min, t_R(2*R*,3*R*): 16.9 min, 98:2 er$_{anti}$.

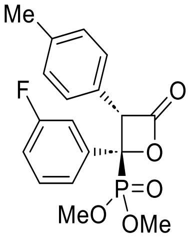

anti-**64**

Detector A Channel 1 211nm

Peak#	Ret. Time	Area%
1	14.559	49.743
2	16.998	50.257
Total		100.000

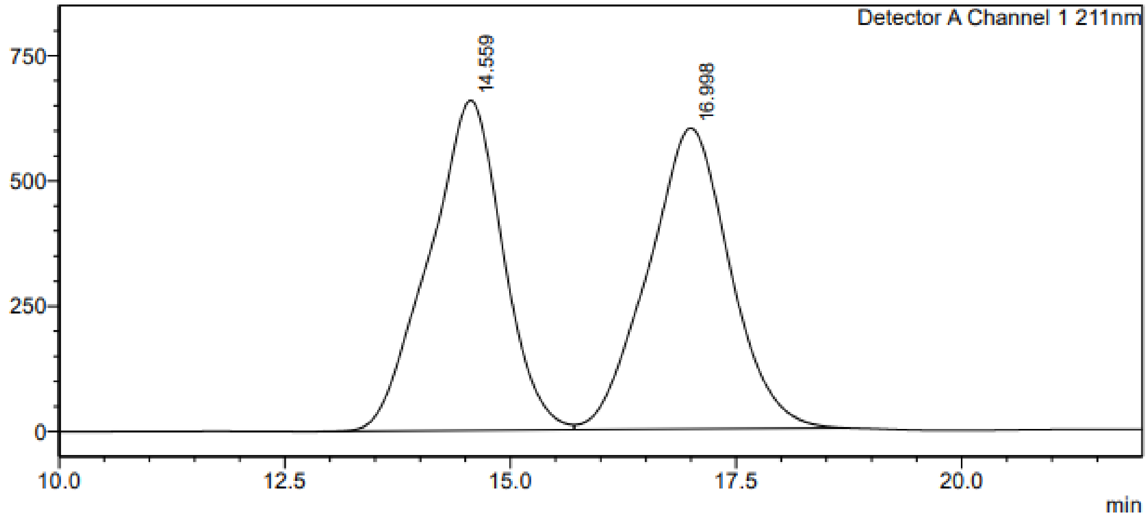

Detector A Channel 1 211nm

Peak#	Ret. Time	Area%
1	14.722	1.724
2	16.909	98.276
Total		100.000

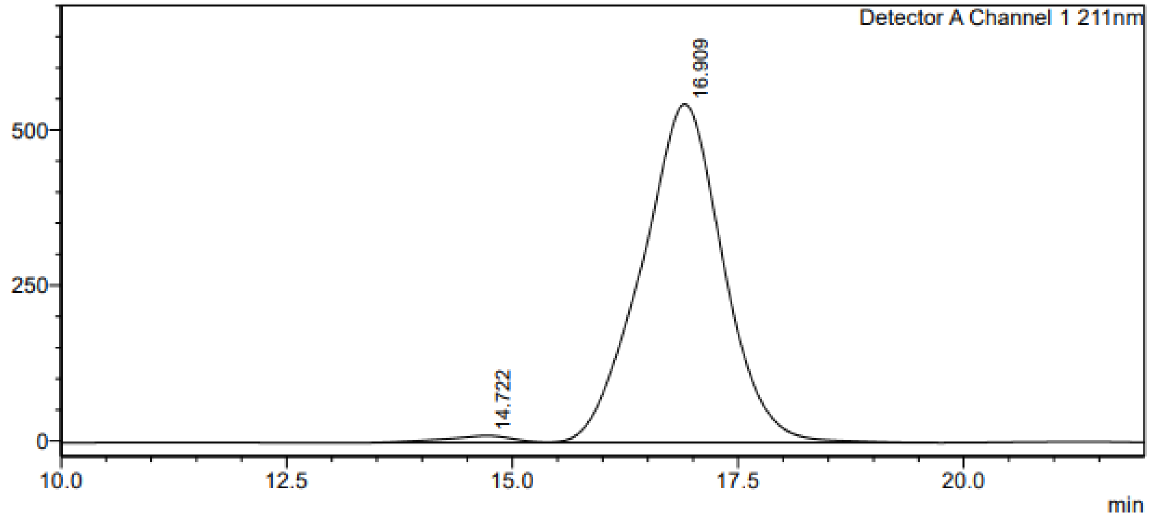

HPLC Data for *syn*-**64**: Chiralpak IA (95:5 hexane : IPA, flow rate 1.0 mLmin^{-1}, 211 nm, 30 ℃) t_R(2*R*,3*S*): 20.9 min, t_R(2*S*,3*R*): 28.1 min, 97:3 er$_{syn}$.

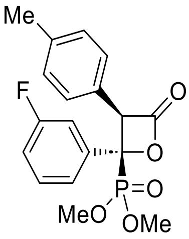

syn-**64**

PDA Ch1 211nm

Peak#	Ret. Time	Area%
1	21.246	50.303
2	28.339	49.697
Total		100.000

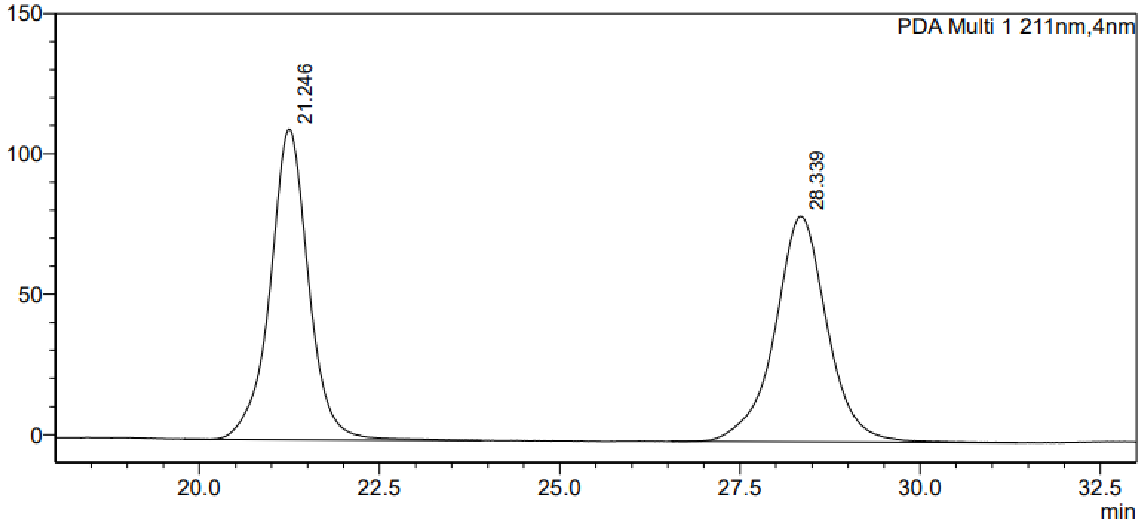

PDA Ch1 211nm

Peak#	Ret. Time	Area%
1	20.908	96.820
2	28.061	3.180
Total		100.000

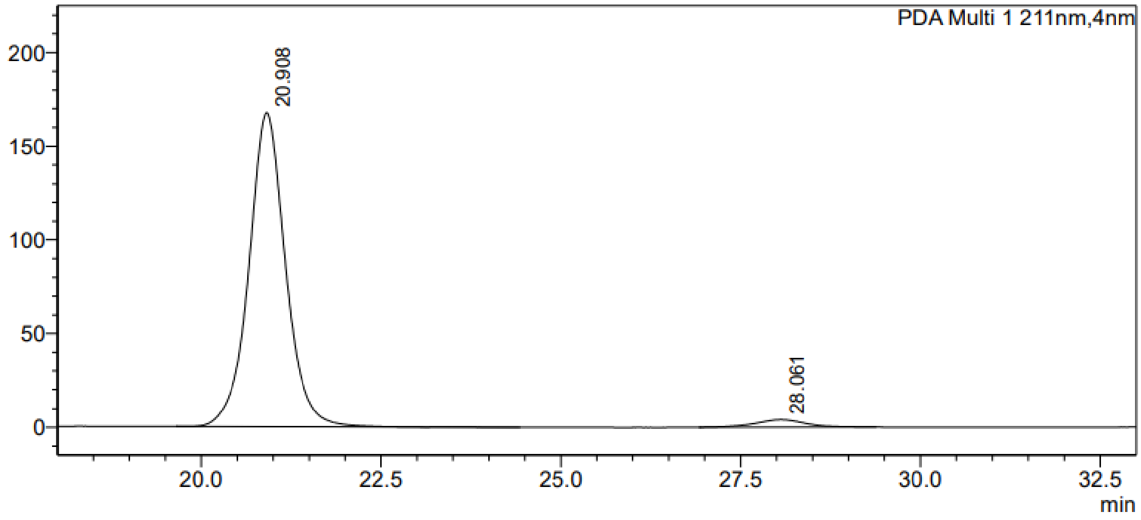

HPLC Data for *anti*-**65**: Chiralpak ID (95:5 hexane : IPA, flow rate 1.5 mLmin^{-1}, 211 nm, 30 ℃) t_R(2*S*,3*S*): 31.0 min, t_R(2*R*,3*R*): 37.1 min, 95:5 er$_{anti}$.

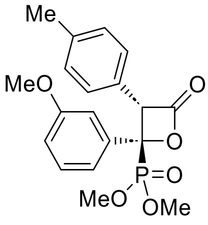

anti-**65**

Detector A Channel 1 211nm

Peak#	Ret. Time	Area%
1	37.702	49.383
2	44.859	50.617
Total		100.000

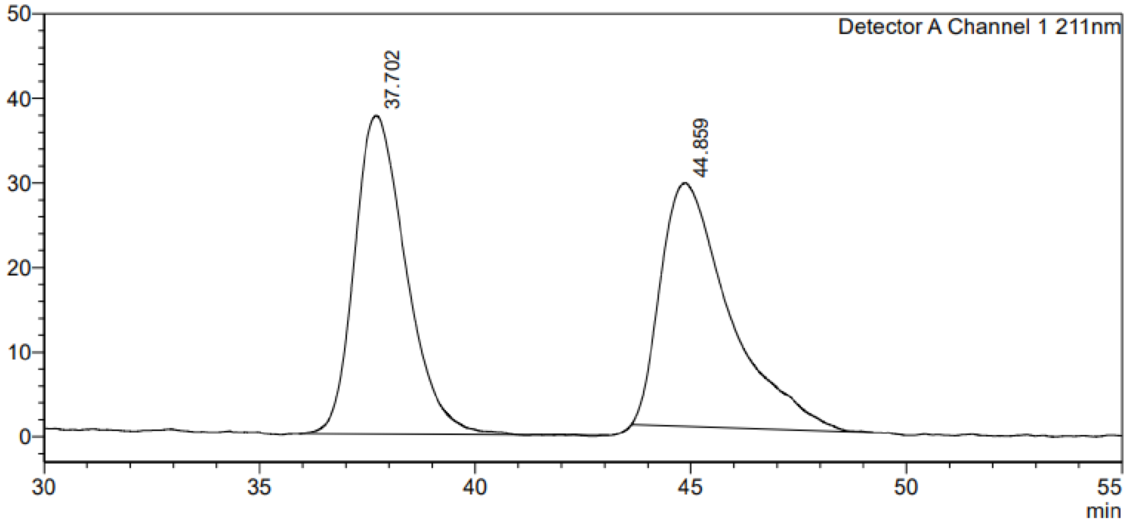

Detector A Channel 1 211nm

Peak#	Ret. Time	Area%
1	31.028	5.199
2	37.097	94.801
Total		100.000

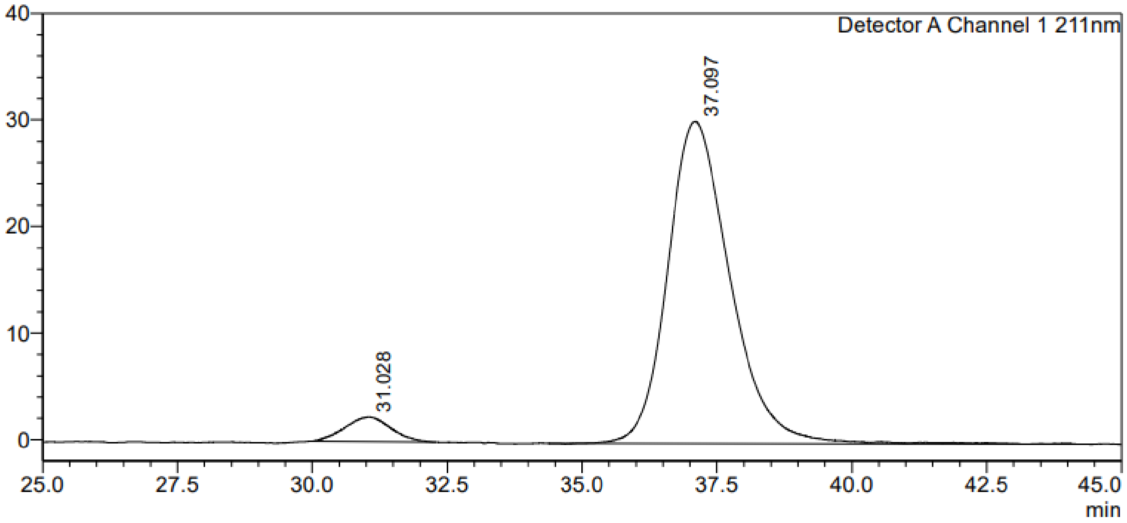

HPLC Data for *anti*-**66**: Chiralpak AD-H (95:5 hexane : IPA, flow rate 1.0 mLmin^{-1}, 211 nm, 30 °C) t_R(2*R*,3*R*): 20.7 min, t_R(2*S*,3*S*): 25.8 min, 96:4 er$_{anti}$.

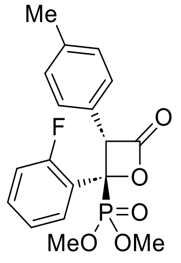

anti-**66**

Detector A Channel 1 211nm

Peak#	Ret. Time	Area%
1	21.173	49.876
2	26.471	50.124
Total		100.000

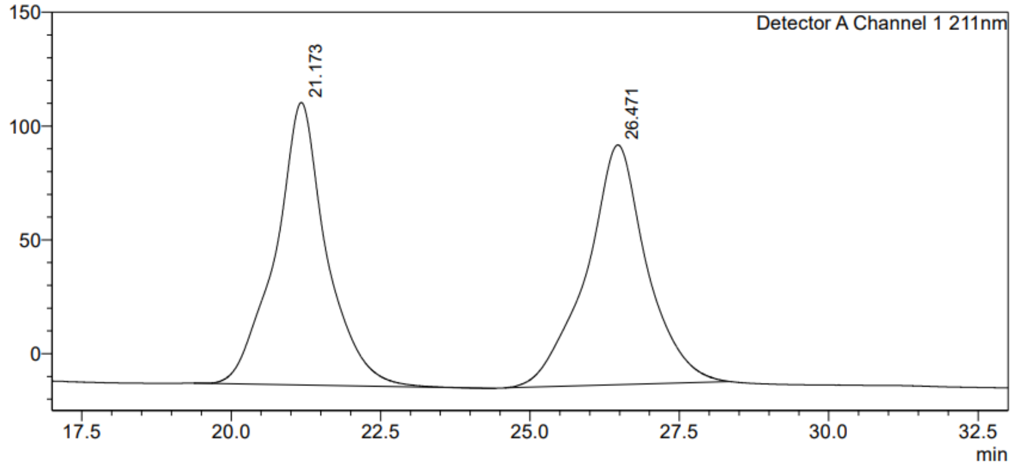

Detector A Channel 1 211nm

Peak#	Ret. Time	Area%
1	20.658	95.722
2	25.753	4.278
Total		100.000

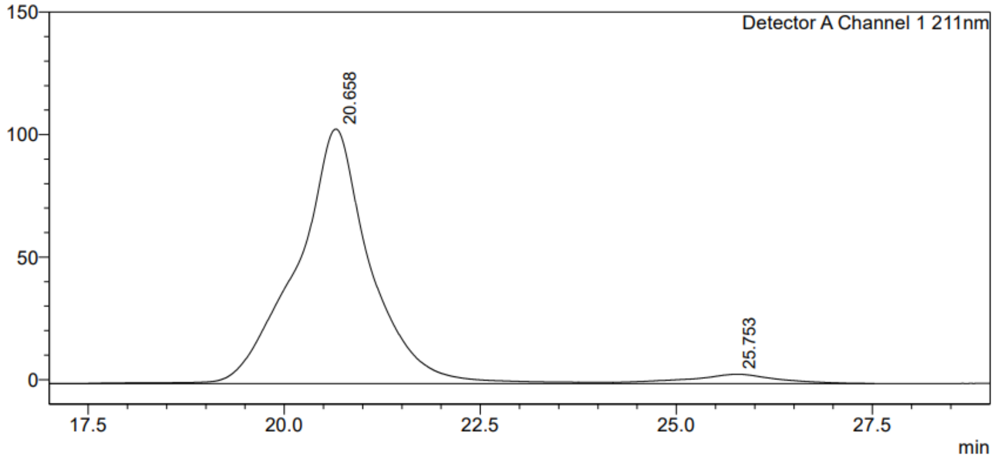

HPLC Data for *syn*-**66**: Chiralpak IA (96:4 hexane : IPA, flow rate 1.5 mLmin^{-1}, 211 nm, 40 ℃) t_R(2*R*,3*S*): 17.2 min, t_R(2*S*,3*R*): 18.4 min, 75:25 er$_{syn}$.

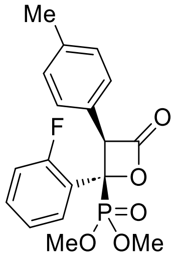

syn-**66**

PDA Ch1 211nm

Peak#	Ret. Time	Area%
1	17.085	49.730
2	18.283	50.270
Total		100.000

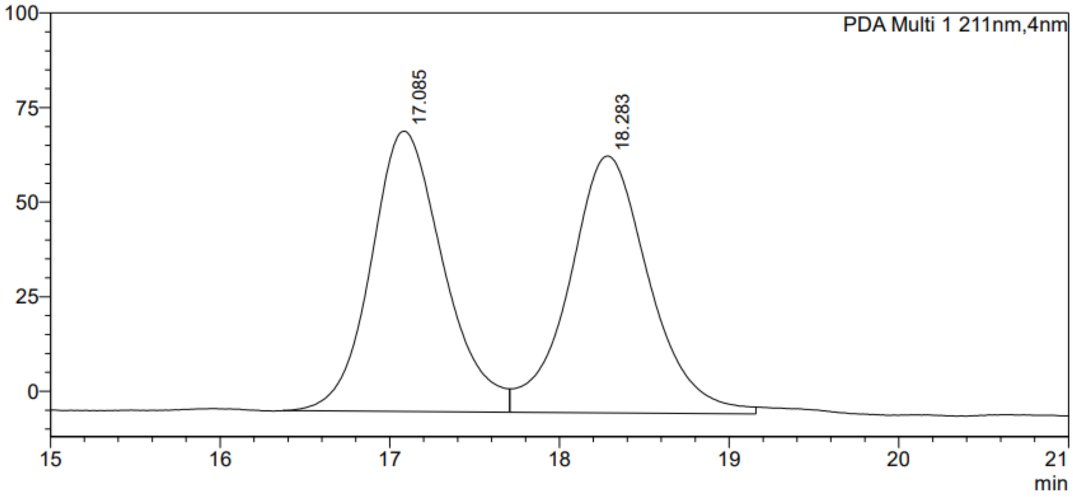

PDA Ch1 211nm

Peak#	Ret. Time	Area%
1	17.158	75.430
2	18.353	24.570
Total		100.000

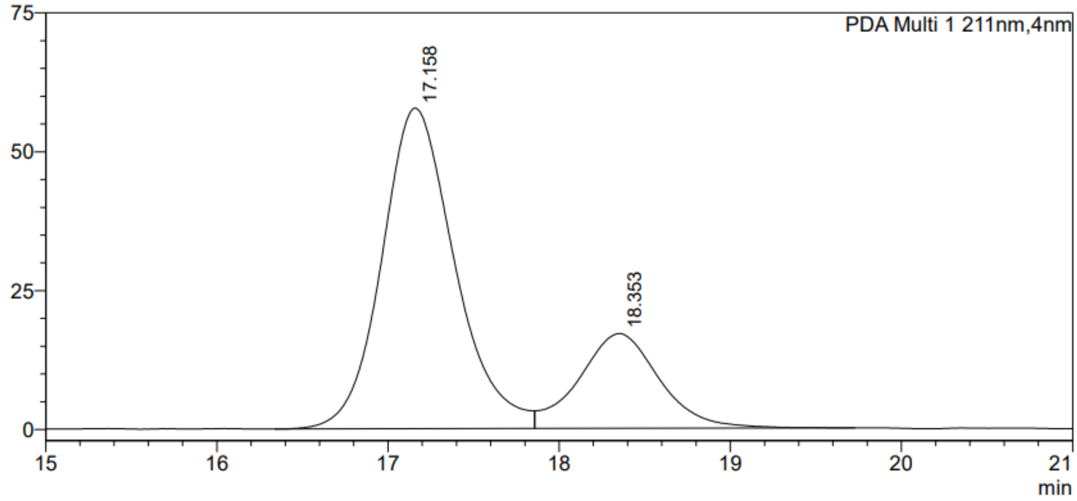

Supplement: SC-016-D5SC00322A-s001 [file SC-016-D5SC00322A-s001.pdf]
